# Long-Term Biological and Behavioural Impact of an Adolescent Sexual Health Intervention in Tanzania: Follow-up Survey of the Community-Based MEMA kwa Vijana Trial

**DOI:** 10.1371/journal.pmed.1000287

**Published:** 2010-06-08

**Authors:** Aoife M. Doyle, David A. Ross, Kaballa Maganja, Kathy Baisley, Clemens Masesa, Aura Andreasen, Mary L. Plummer, Angela I. N. Obasi, Helen A. Weiss, Saidi Kapiga, Deborah Watson-Jones, John Changalucha, Richard J. Hayes, for the MEMA kwa Vijana Trial Study Group

**Affiliations:** 1 London School of Hygiene & Tropical Medicine, London, United Kingdom; 2 National Institute for Medical Research, Mwanza Centre, Tanzania; 3 AMREF (African Medical & Research Foundation), Mwanza, Tanzania; 4 Liverpool School of Tropical Medicine, Liverpool, United Kingdom; University of Western Sydney, Australia

## Abstract

**Background:**

The ability of specific behaviour-change interventions to reduce HIV infection in young people remains questionable. Since January 1999, an adolescent sexual and reproductive health (SRH) intervention has been implemented in ten randomly chosen intervention communities in rural Tanzania, within a community randomised trial (see below; NCT00248469). The intervention consisted of teacher-led, peer-assisted in-school education, youth-friendly health services, community activities, and youth condom promotion and distribution. Process evaluation in 1999–2002 showed high intervention quality and coverage. A 2001/2 intervention impact evaluation showed no impact on the primary outcomes of HIV seroincidence and herpes simplex virus type 2 (HSV-2) seroprevalence but found substantial improvements in SRH knowledge, reported attitudes, and some reported sexual behaviours. It was postulated that the impact on “upstream” knowledge, attitude, and reported behaviour outcomes seen at the 3-year follow-up would, in the longer term, lead to a reduction in HIV and HSV-2 infection rates and other biological outcomes. A further impact evaluation survey in 2007/8 (∼9 years post-intervention) tested this hypothesis.

**Methods and Findings:**

This is a cross-sectional survey (June 2007 through July 2008) of 13,814 young people aged 15–30 y who had attended trial schools during the first phase of the MEMA kwa Vijana intervention trial (1999–2002). Prevalences of the primary outcomes HIV and HSV-2 were 1.8% and 25.9% in males and 4.0% and 41.4% in females, respectively. The intervention did not significantly reduce risk of HIV (males adjusted prevalence ratio [aPR] 0.91, 95%CI 0.50–1.65; females aPR 1.07, 95%CI 0.68–1.67) or HSV-2 (males aPR 0.94, 95%CI 0.77–1.15; females aPR 0.96, 95%CI 0.87–1.06). The intervention was associated with a reduction in the proportion of males reporting more than four sexual partners in their lifetime (aPR 0.87, 95%CI 0.78–0.97) and an increase in reported condom use at last sex with a non-regular partner among females (aPR 1.34, 95%CI 1.07–1.69). There was a clear and consistent beneficial impact on knowledge, but no significant impact on reported attitudes to sexual risk, reported pregnancies, or other reported sexual behaviours. The study population was likely to have been, on average, at lower risk of HIV and other sexually transmitted infections compared to other rural populations, as only youth who had reached year five of primary school were eligible.

**Conclusions:**

SRH knowledge can be improved and retained long-term, but this intervention had only a limited effect on reported behaviour and no significant effect on HIV/STI prevalence. Youth interventions integrated within intensive, community-wide risk reduction programmes may be more successful and should be evaluated.

**Trial Registration:**

ClinicalTrials.gov NCT00248469

*Please see later in the article for the Editors' Summary*

## Abstract 2

David Ross and colleagues conduct a follow-up survey of the community-based MEMA kwa Vijana (“Good things for young people”) trial in rural Tanzania to assess the long-term behavioral and biological impact of an adolescent sexual health intervention.

## Editors' Summary

**Background:**

Every year, about 2.5 million people become infected with the human immunodeficiency virus (HIV), the virus that causes AIDS. HIV is most often spread through unprotected sex with an infected partner, so individuals can reduce their risk of HIV infection by abstaining from sex, by delaying first sex, by having few partners, and by always using a condom. And, because nearly half of new HIV infections occur among youths (15- to 24-year-olds), programs targeted at adolescents that encourage these protective behaviors could have a substantial impact on the HIV epidemic. One such program is the MEMA kwa Vijana (“Good things for young people”) program in rural Tanzania. This program includes in-school sexual and reproductive health (SRH) education for pupils in their last three years of primary education (12- to 15-year-olds) that provides them with the knowledge and skills needed to delay sexual debut and to reduce sexual risk taking. Between 1999 and 2002, the program was trialed in ten randomly chosen rural communities in the Mwanza Region of Tanzania; ten similar communities that did not receive the intervention acted as controls. Since 2004, the program has been scaled up to cover more communities.

**Why Was This Study Done?:**

Although the quality and coverage of the MEMA kwa Vijana program was good, a 2001/2002 evaluation found no evidence that the intervention had reduced the incidence of HIV (the proportion of the young people in the trial who became HIV positive during the follow-up period) or the prevalence (the proportion of the young people in the trial who were HIV positive at the end of the follow-up period) of herpes simplex virus 2 (HSV-2, another sexually transmitted virus). However, the evaluation found improvements in SRH knowledge, in reported sexual attitudes, and in some reported sexual behaviors. Evaluations of other HIV prevention programs in other developing countries have also failed to provide strong evidence that such programs decrease the risk of HIV infection or other biological outcomes such as the frequency of other sexually transmitted infections or pregnancies, even when SRH knowledge improves. One possibility is that it takes some time for improved SRH knowledge to be reflected in true changes in sexual behavior and in HIV prevalence. In this follow-up study, therefore, researchers investigate the long-term impact of the MEMA kwa Vijana program on HIV and HSV-2 prevalence and ask whether the improvement in knowledge, reported attitudes and sexual risk behaviours seen at the 3-year follow up has persisted.

**What Did the Researchers Do and Find?:**

In 2007/8, the researchers surveyed nearly 14,000 young people who had attended the trial schools between 1999 and 2002. Each participant had their HIV and HSV-2 status determined and answered questions (for example, “can HIV be caught by sexual intercourse (making love) with someone,” and “if a girl accepts a gift from a boy, must she agree to have sexual intercourse (make love) with him?”) to provide three composite sexual knowledge scores and one composite attitude score. 1.8% of the male and 4.0% of the female participants were HIV positive; 25.9% and 41.4% of the male and female participants, respectively, were HSV-2 positive. The prevalences were similar among the young people whose trial communities had been randomly allocated to receive the MEMA kwa Vijana Program and those whose communities had not received it, indicating that the MEMA kwa Vijana intervention program had not reduced the risk of HIV or HSV-2. The intervention program was associated, however, with a reduction in the proportion of men reporting more than four sexual partners in their lifetime and with an increase in reported condom use at last sex with a non-regular partner among women. Finally, although the intervention had still increased SRH knowledge, it now had had no impact on reported attitudes to sexual risk, reported pregnancies, or other reported risky sexual behaviors beyond what might have happened due to chance.

**What Do These Findings Mean?:**

These findings indicate that, in the MEMA kwa Vijana trial, SRH knowledge improved and that this improved knowledge was retained for many years. Disappointingly, however, this intervention program had only a limited effect on reported sexual behaviors and no effect on HIV and HSV-2 prevalence at the 9-year follow-up. Although these findings may not be generalizable to other adolescent populations, they suggest that intervention programs that target only adolescents might not be particularly effective. Young people might find it hard to put their improved skills and knowledge into action when challenged, for example, by widespread community attitudes such as acceptance of older male–younger female relationships. Thus, the researchers suggest that the integration of youth HIV prevention programs within risk reduction programs that tackle sexual norms and expectations in all age groups might be a more successful approach and should be evaluated.

**Additional Information:**

Please access these Web sites via the online version of this summary at http://dx.doi.org/10.1371/journal.pmed.1000287.

- This study is further discussed in a *PLoS Medicine* Perspective by Rachel Jewkes
- More information about the MEMA kwa Vijana program is available at their Web site
- Information is available from the Programme for Research and Capacity Building in Sexual and Reproductive Health and HIV in Developing Countries on recent and ongoing research on HIV infection and other STIs
- Information is available from the World Health Organization on HIV and on the health of young people
- Information on HIV is available from UNAIDS
- Information on HIV in children and adolescents is available from UNICEF
- Information on HIV prevention interventions in the education sector is available from UNESCO
- Information on HIV infection and AIDS is available from the US National Institute of Allergy and Infectious Diseases
- The US Centers for Disease Control and Prevention provide information on HIV/AIDS and on HIV/AIDS among youth  (in English and Spanish)
- HIV InSitehas comprehensive information on all aspects of HIV/AIDS, including links to information on the prevention of HIV/AIDS
- Information is available from Avert, an international AIDS charity, on many aspects of HIV/AIDS, including information on HIV and AIDS prevention and AIDS and sex education (in English and Spanish)

## Introduction

In 2007, 45% of new HIV infections worldwide were among youth (15–24 y) [1], and several studies have demonstrated high rates of other sexually transmitted infections (STIs) and pregnancy in this age group [2],[3]. Effective HIV prevention interventions focusing on adolescents should therefore have a substantial impact on the HIV epidemic. Behavioural interventions [4]–[6] and male circumcision [7],[8] are advocated as the most effective HIV control strategies. However, despite a very wide range of different approaches and specific interventions that might be used to try to induce behaviour change, empirical evidence on the efficacy of behavioural interventions to prevent HIV is weak and contradictory [9]–[14]. While most programme evaluations in developing countries have shown an improvement in knowledge, reported communication about sexual matters, and attitudes, at least in the short-term about one-third of programme evaluations showed no changes in reported sexual behaviours, and many other studies found reported sexual behaviour change in only some subgroups [11],[15].

Few previous trials to assess the efficacy of behavioural interventions have measured biomedical endpoints [9],[12]–[14],[16]. The inclusion of such outcomes is critically important (1) because of the limited validity of reported sexual behaviour, particularly in young people [17]–[20]; (2) because of the potential for social desirability bias; and (3) because reductions in HIV, STI, and pregnancy are usually the ultimate objectives for these interventions.

**Figure 1:**
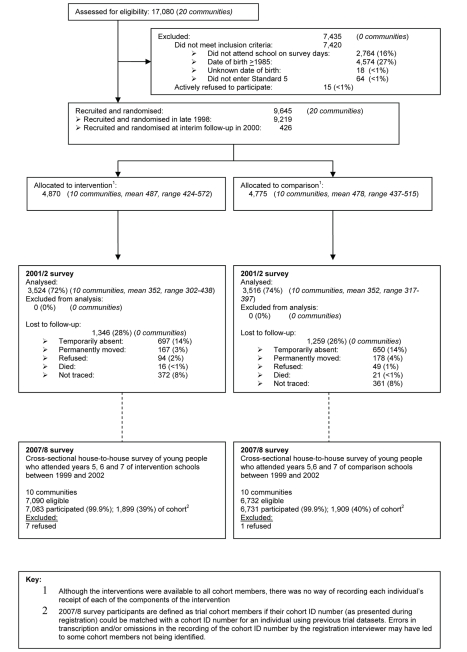
The MEMA kwa Vijana Community Randomised Controlled Trial (1998–2008).

We report results of a long-term (>8 y) community-randomised trial to evaluate the impact of the MEMA kwa Vijana (“Good Things for Young People”) intervention in rural Tanzania on the prevalence of HIV, other STIs, and pregnancy, and on sexual health knowledge, attitudes, and reported sexual behaviour (Figure 1).

**Figure 2:**
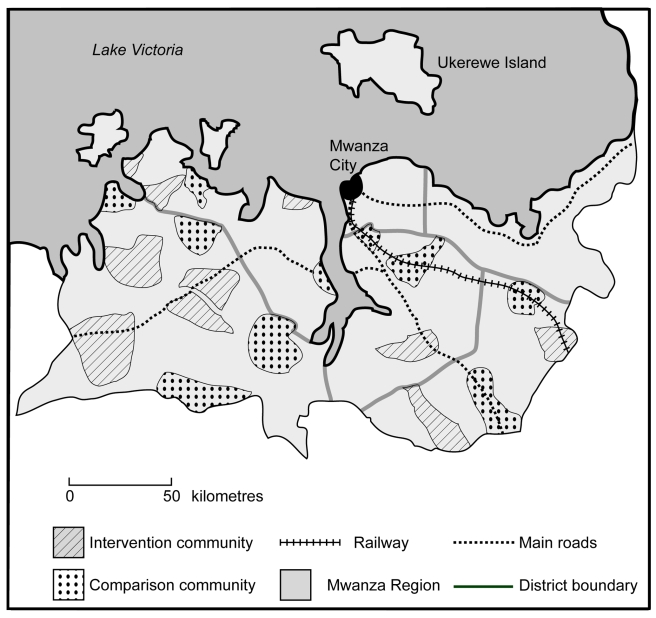
Map of Mwanza Region, Tanzania, showing intervention and comparison communities.

The design of the trial [21] and intervention [22], and the results of the 2001/2 impact evaluation [23] are described in detail elsewhere. In summary, the trial was conducted in 20 distinct rural communities in Mwanza Region, Tanzania (Figure 2). The study communities were grouped into three risk strata using data from a prior population-based survey [3]. Restricted randomisation was used to balance HIV and *Chlamydia trachomatis* prevalence between the two trial arms [21]. Ten communities (58 primary schools, 18 health facilities) were randomised to receive the intervention from 1999 onwards; the other ten (63 primary schools, 21 health facilities) were comparison communities.

The intervention was based on the principles of social learning theory, and its main aims were to reduce the incidence of HIV/STI and unintended pregnancies by providing young people with the knowledge and skills to enable them to delay sexual debut, reduce sexual risk-taking (including reducing numbers of sexual partners and promoting condom use), and increase their appropriate use of sexual health services (e.g., STI treatment, family planning). To encourage sustainability and replicability, the intervention was delivered through existing structures and supervision systems by government workers, trained and supported by eight staff members from the African Medical and Research Foundation (AMREF).

This adolescent sexual and reproductive health (ASRH) intervention had four components [22]: (1) a participatory, teacher-led, peer-assisted, in-school programme, comprising an average of 12 forty-minute school sessions per year, in primary school years 5–7 (Box 1); (2) the provision of youth-friendly health services with quarterly supervision; (3) community-based condom promotion and distribution by and for youth, which was introduced in early 2000 in response to a process evaluation that found that young people had difficulty accessing condoms; and (4) limited community-wide activities including an initial mobilisation week in each community and annual youth health weeks.

**Box 1. Topics Covered during the MEMA kwa Vijana In-School, Teacher-Led Peer-Assisted Sessions (approximately 12 forty-minute sessions per school year)**

**Year 5**

- What is reproductive health and why is it important?
- Leaving childhood: Puberty
- What are HIV and AIDS?
- The facts about AIDS
- The facts about sexually transmitted diseases
- Girls and Boys have equal abilities
- Misconceptions about sex
- Refusing temptations
- Saying No to sex
- Sexually Transmitted Diseases: Going to the clinic

**Year 6**

- Review of last years' learning
- How HIV infection causes AIDS
- How Sexually Transmitted Diseases are spread
- The relationship between HIV and Sexually Transmitted Diseases
- Reproductive organs and their functions
- Pregnancy and menstruation
- Respecting other people's decisions
- Recognising and avoiding temptations
- Protecting yourselves: What are condoms?
- Revision

**Year 7**

- Review of previous years' learning
- How to avoid HIV infection and AIDS
- Sexually Transmitted Diseases and their consequences
- Making good decisions
- Practising saying ‘No’
- Being faithful
- Achieving your future expectations
- Planning for your future
- Protecting yourself: Correct use of condoms & the truth about condoms
- Revision

Source: Teachers' guides accessible at http://www.memakwavijana.org.

Surveys in the trial communities between 1999 and 2001 showed that sexual health programmes in the comparison communities were very limited. Results of internal [22],[24] and external evaluations by international and national experts [25]–[27] conducted in 1999–2002 found that the multi-component intervention was implemented well and achieved high coverage.

The first two intervention components have continued in the intervention communities since 2002 and were started in the comparison communities between May 2005 and July 2007 as part of the scale-up and operations research component of the MEMA kwa Vijana Programme [28],[29].

The impact of the intervention was evaluated in 2001/2, approximately three years after recruitment, in 7,040 (73%) out of a cohort of 9,645 adolescents recruited into that phase of the trial (Figure 1). The 2001/2 impact evaluation showed that the intervention had resulted in substantial and statistically significant improvements in knowledge and reported attitudes, with adjusted relative risks ranging from 1.3 to 1.8 [23]. Amongst males, the intervention had also delayed reported sexual debut, reduced the reported number of sexual partners in the past 12 months, and increased reported condom use. Females reported an increase in first use of condoms during the 3-year follow-up period [23]. The results suggested that the impact of the intervention on knowledge, reported attitudes, and reported behaviours was greater in males than females, and in those who had received more years of the in-school component of the intervention. However, there was no consistent impact of the intervention on biological indicators of HIV, other STIs, and pregnancy rates. It was postulated that the duration of follow-up (3 years) may have been too short to see the impact of improvement in young men's risk-taking on biological outcomes in young women, due to the difference in the average age of sexual partners [30]. Also, exposure of more school years of adolescents to the intervention may have been needed to effect a significant change in the norms of young people as a whole. Furthermore, by the time of the 2001/2 survey, 40% of the evaluation cohort had only received 1 year of the in-school intervention, and the highest risk group (year 6 at recruitment) had had the least exposure to the in-school intervention. This paper reports the findings of a long-term evaluation carried out to evaluate this hypothesis.

## Methods

### Ethics Statement

The trial protocol received ethical and research clearance from the Tanzanian Medical Research Coordinating Committee and the Ethics Committee of the London School of Hygiene & Tropical Medicine. Signed informed consent was obtained from each participant on the day of the survey round. Additional written consent from parents was obtained for participants under the age of 18 y.

Between June 2007 and July 2008, a cross-sectional survey was conducted in the 20 MEMA kwa Vijana trial communities to evaluate the long-term impact of the intervention. By then, nine consecutive school year groups had participated in the in-school component of the intervention and the health services intervention had also been in place for 8–9 y. As no external evaluations of the coverage and quality of the intervention had taken place since 2002, the long-term evaluation survey was restricted to young people who had attended at least one of school years 5–7 in the trial communities within the period 1999–2002 inclusive. Primary outcomes were HIV seroprevalence and HSV-2 seroprevalence. Secondary biological, knowledge, reported attitude, and reported behaviour outcomes were similar but not always identical to those used in the 2001/2 evaluation. Each of the attitudinal and knowledge outcomes were based on the answers to three questions (Table 1) [23].

**Table 1:** Questions used in the composite knowledge and attitudes scores.

Question	Correct Answer
**1. Knowledge on acquisition of HIV**	
1.1. Can HIV be caught by sexual intercourse (making love) with someone?	Yes
1.2. Can you catch HIV by sharing a plate of food with an HIV-positive person?	No
1.3. Can a person who looks strong and healthy have HIV?	Yes
**2. Knowledge on acquisition of sexually transmitted diseases**	
2.1. Can pus or abnormal fluids coming out of the private parts be caught by sexual intercourse (making love) with someone?	Yes
2.2. Can schistosomiasis be caught by sexual intercourse (making love) with someone?	No
2.3. Can an ulcer on the private parts be caught by sexual intercourse (making love) with someone?	Yes
**3. Knowledge on pregnancy prevention**	
3.1. Is it possible for a girl to become pregnant the first time she makes love?	Yes
3.2. Is it possible for a person to prevent pregnancy by using a condom while having sexual intercourse (making love)?	Yes
3.3. Is it possible for a person to prevent pregnancy by not having sexual intercourse (making love) at all?	Yes
**4. Sexual attitudes**	
4.1. If a man or youth wants to have sexual intercourse (make love) with a girl, can she refuse to have sexual intercourse (make love) with him if he is older than her?	Yes
4.2. If a man or youth wants to have sexual intercourse (make love) with a girl, can she refuse to have sexual intercourse (make love) with him if he is her lover?	Yes
4.3. If a girl accepts a gift from a boy, must she agree to have sexual intercourse (make love) with him?	No

Between June 2007 and May 2008 a household census in the 20 trial communities identified potentially eligible young people and invited them to attend the survey at a central location in the village two or three days later. Some additional young people who had not specifically been invited by the census interviewers but who heard about the survey and thought that they might be eligible also attended. At the survey, detailed checks identified those who had attended years 5, 6, and/or 7 in a trial school between 1999 and 2002. Eligible attendees who gave informed consent were interviewed about their knowledge, reported attitudes, and reported sexual behaviour. Blood and urine specimens were collected. A clinician asked about STI symptoms (males and females) and examined males for signs of STIs. HIV counselling and testing was offered using parallel HIV rapid tests (SD Bioline HIV-1/2 3.0 [Standard Diagnostics] and Determine HIV1/2 [Abbott Laboratories]). In order to include additional eligible young people, all 20 trial communities, nearby secondary schools, and major migration points within the Lake Zone of Tanzania were revisited in June and July 2008.

### Laboratory Methods

Sera were tested for HIV-1 and HIV-2 in parallel, using third-generation Murex HIV 1.2.0 ELISA (Abbott-Murex, Dartford, UK) and third-generation Vironostika HIV UNIFORM II plus O (Biomeriux, Boxtel, Netherlands). Sera with discordant ELISA results were retested up to two more times on both ELISAs. Persistently discordant samples were tested for p24 antigen using Bio-Rad Genetic System HIV1 Ag EIA (Bio-Rad, Lacoquette, France), and p24-negative samples were tested with Inno-Lia HIV1/2 score Assay (Inno-Genetics NV, Gent, Belgium). INNO-LIA-indeterminate specimens were classified as negative.

Sera were tested for antibodies to HSV-2 using KALON HSV Type 2 IgG ELISA (KALON Biologicals, Guildford, UK) following the manufacturer's instructions. KALON ELISA-indeterminate samples were retested. Persistently indeterminate specimens were classified as negative. Lifetime exposure to syphilis was examined using the Serodia *Treponema pallidum* particle agglutination (TPPA) test (Fujirebio, Japan). Those positive on TPPA were further tested for active syphilis using the Immutrep carbon antigen rapid plasma reagin (RPR) test (Omega Diagnostics, Hillfoot, UK).

Urine specimens were tested for *Chlamydia trachomatis* (CT) and *Neisseria gonorrhoeae* (NG) by Amplicor PCR (Roche Diagnostics, Branchburg, USA) according to the manufacturer's instructions. PCR-positive samples were retested up to twice and classified using a “two out of three strategy.” NG samples that remained positive on repeated Amplicor PCR testing were confirmed with an in-house 16S rDNA PCR using primers NG01: 5′-GACGGCAGCACAGGGAAGCTTGCTTCTCGG-3′ and NG03M: 5′-TCGGCCGCCGATATTGGCAA-3′ [31],[32]. Only samples with positive 16S PCR results were reported as positive for NG.

### Statistical Methods

Allocation to the arm of the trial for the primary analysis was based on the community where a young person had first attended one of school years 5–7 in a trial school between 1999 and 2002. Prior to the survey, it was estimated that 14,520 (363 males and females, respectively, in each community) might be found, based on the estimated number of eligible young people who might still be living in trial communities and would agree to participate. Prevalence and incidence estimates from previous studies in Mwanza Region were used to predict an HIV seroprevalence and HSV-2 seroprevalence of, respectively, 2% and 25% in males and 5% and 35% in females [23],[33],[34]. With ten communities per arm and assuming a between-community coefficient of variation of 0.2, 14,520 participants would provide 85% power to detect a 50% reduction in HIV prevalence in males and 79% power to detect a 35% reduction in females. The power to detect a 25% reduction in HSV-2 prevalence would be 77% for males and 80% for females.

The data were analysed as described for stratified cluster-randomised trials in Hayes and Moulton [35]. The number of individuals differed slightly for each analysis because of missing results. Impact was measured using prevalence ratios. The unadjusted prevalence ratio was calculated as the ratio of the geometric mean prevalence for the ten communities in each arm or the ratio of arithmetic mean prevalences if an outcome had zero cases in at least one community. The 95% confidence interval (CI) for the prevalence ratio was calculated using a stratified t-test with 14 degrees of freedom, with variance estimated from the residual mean square from a two-way analysis of variance (ANOVA) of community log-prevalence on stratum and study arm.

Adjusted prevalence ratios (aPRs) were calculated using logistic regression to adjust for individual level covariates. The regression model included terms for the adjustment factors (age group, stratum, and ethnic group [Sukuma/non-Sukuma]), but not study arm. For each community, the fitted model was used to compute the ratio of observed to expected events (O/E). The adjusted prevalence ratio was obtained as the ratio of the geometric mean of these O/E estimates for the two study arms, and variances and CIs were obtained from an ANOVA of log(O/E) on stratum and study arm.

## Results

**Figure 3:**
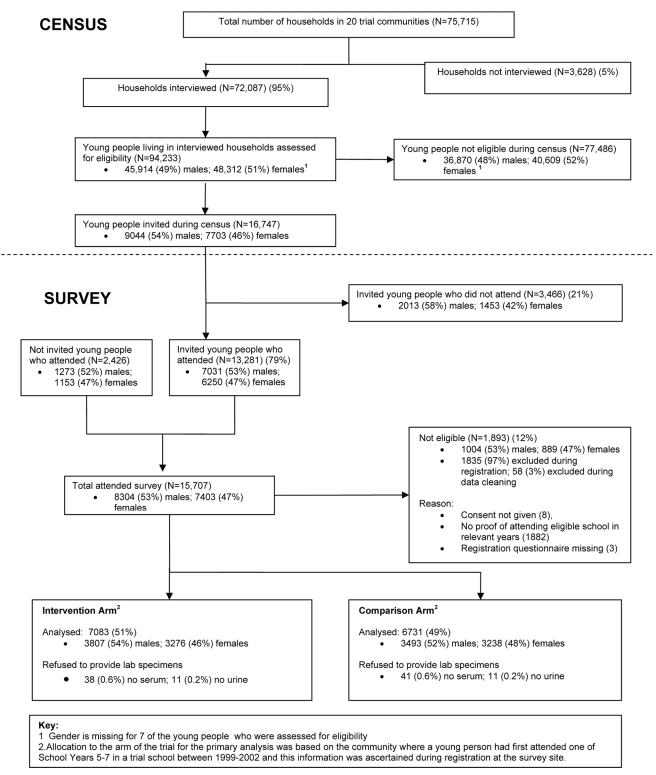
Long-term evaluation of the MEMA kwa Vijana intervention, 2007–2008.

Overall 72,087 (95%) of an estimated 75,715 households in the survey areas were visited during the census. 449,298 individuals were reported to be living in the visited households. At the census, 16,747 young people were invited to attend the survey; 13,281 (79%) of these actually attended along with an additional 2,426 young people (Figure 3).

At the survey, 88% (13,814/15,707) of attendees met the eligibility criteria and were enrolled; 7,083 (51%) from intervention and 6,731 (49%) from comparison communities (Figure 3). Preliminary estimates based on data from the cohort recruitment survey in 1998 suggested that there would be an average of 720 men and 720 women from each community who had, between 1999 and 2002, completed at least one of the final three years of primary school in that community. We were able to survey an average of 365 males and 326 females in the 2007/8 survey.

Participants' median age was 22 y for males (interquartile range [IQR] 20–24 y) and 21 years for females (IQR 19–23 y) (Table 2). The majority of participants (80%) were from the Sukuma ethnic group and over 80% were Christian. Relatively few (10%–20%) had higher than primary level education. Over half of females and one third of males were married, and 92% reported ever having had sex. Males were on average 2.4 y older and females 4 y younger than their most recent sexual partner. 41% of males were circumcised on clinical inspection. 75% of participants opted to know and therefore received their HIV result.

**Table 2:** Characteristics of the 13,814 long-term evaluation (2007/8) participants, by sex and trial arm.

Variable	Males (n = 7,300)		Females (n = 6,514)	
	Intervention (n = 3,807)	Comparison (n = 3,493)	Intervention (n = 3,276)	Comparison (n = 3,238)
**Age, n (%)**				
<21 y	1,150 (30%)	896 (26%)	1,357 (41%)	1,284 (40%)
21–22 y	990 (26%)	987 (28%)	898 (27%)	966 (30%)
23–24 y	976 (26%)	938 (27%)	763 (23%)	735 (23%)
≥25 y	690 (18%)	672 (19%)	257 (8%)	252 (8%)
**Median age and IQR, y**	22 (20–24)	22 (20–24)	21 (19–23)	21 (20–23)
**Sukuma ethnic group, n (%)**	2,882 (76%)	2,834 (81%)	2,549 (78%)	2,747 (85%)
**Religion, n (%)**				
Christian	3,099 (81%)	2,784 (80%)	2,860 (87%)	2,905 (90%)
Muslim	143 (4%)	187 (5%)	142 (4%)	136 (4%)
Other religion	20 (0.5%)	38 (1%)	7 (0.2%)	2 (0.1%)
None	542 (14%)	476 (14%)	260 (8%)	187 (6%)
**Currently married, n (%)**	1,242 (33%)	1,202 (34%)	1,806 (55%)	1,858 (57%)
**Ever married, n (%)**	1,346 (35%)	1,327 (38%)	2,121 (65%)	2,168 (67%)
**Highest level of education, n (%)**				
Secondary school or higher	864 (23%)	678 (19%)	472 (14%)	411 (13%)
**Male circumcision (clinical examination), n (%)**	1,596 (43%)	1,315 (38%)	NA	NA
**Median reported age at sexual debut, y**	18	17	17	17
**Blood transfusion in the previous 5 y, n (%)**	30 (1%)	29 (1%)	82 (3%)	80 (3%)
**Number of injections in the previous 12 mo, n (%)**				
0	2,949 (78%)	2,700 (78%)	1,821 (56%)	1,703 (53%)
1	265 (7%)	236 (7%)	406 (13%)	423 (13%)
≥2	579 (15%)	525 (15%)	1,008 (31%)	1,064 (33%)
**Years of exposure to in-school component of MEMA kwa Vijana between 1999 and 2004** a **, n (%)**				
1	629 (17%)	576 (16%)	515 (16%)	517 (16%)
2	616 (16%)	647 (19%)	555 (17%)	518 (16%)
≥3	2,562 (67%)	2,270 (65%)	2,206 (67%)	2,203 (68%)
**Years since last exposure to in-school intervention (or comparison), n (%)**				
3	711 (19%)	551 (16%)	604 (18%)	619 (19%)
4	715 (19%)	566 (16%)	604 (18%)	525 (16%)
5	623 (16%)	602 (17%)	521 (16%)	574 (18%)
6	622 (16%)	632 (18%)	576 (18%)	555 (17%)
7	543 (14%)	594 (17%)	489 (15%)	466 (14%)
8	593 (16%)	548 (16%)	482 (15%)	499 (15%)
**Mean number of years**	5.4	5.5	5.4	5.4

a Or exposure to equivalent years in comparison school.

Two-thirds of participants had had the opportunity to receive 3 y of the in-school intervention. A high proportion (91%) of both males and females from the intervention communities stated that they had attended at least one MEMA kwa Vijana session while in primary school. On average, participants had last been exposed to the in-school intervention 5.4 y prior to the survey. Of the original MEMA kwa Vijana trial cohort identified in 1998, 3,808 (40%) were interviewed during the 2007/8 survey (Figure 1).

Correct knowledge and desirable reported attitudes were higher in intervention communities. There was evidence of an association for each outcome (adjusted prevalence ratio [aPR] from 1.11–1.31 for males and 1.11–1.24 for females), except for the “attitudes to sex” in females (Table 3).

**Table 3:** Impact of intervention on knowledge, reported attitudes, and reported behaviours by sex in 2007/8.

Outcome	Males			Females		
	Prevalence^a^		aPR^b^ (CI)	Prevalence^a^		aPR^b^ (CI)
	Intervention (n = 3807), n (%)	Comparison (n = 3493), n (%)		Intervention (n = 3276), n (%)	Comparison (n = 3238), n (%)	
**Knowledge** c						
HIV acquisition	2,773 (73%)	2,295 (66%)	1.11 (0.99,1.23)	2,233 (68%)	1,952 (61%)	1.11 (1.00,1.24)
STD acquisition	2,056 (54%)	1,591 (46%)	1.18 (1.04,1.34)	1,253 (38%)	974 (30%)	1.24 (0.97,1.58)
Pregnancy prevention	3,133 (83%)	2,410 (69%)	1.19 (1.12,1.26)	2,304 (71%)	1,934 (60%)	1.17 (1.06,1.30)
**Reported attitudes** c						
Attitudes to sex	1,053(28%)	759 (22%)	1.31 (0.97,1.77)	359 (11%)	332 (10%)	1.09 (0.67,1.77)
**Reported sexual behaviour**						
Age at first sex <16 y	954 (25%)	956 (28%)	0.91 (0.80,1.05)	903 (28%)	865 (27%)	1.01 (0.80,1.28)
>2 (female) or >4 (male) lifetime sexual partners	1,412 (37%)	1,531 (44%)	0.87 (0.78,0.97)	1,096 (34%)	1,191 (37%)	0.89 (0.75,1.05)
>1 partner in last 12 months	1,542 (41%)	1,557 (45%)	0.92 (0.79,1.08)	333 (10%)	325 (10%)	0.97 (0.76,1.23)
Used condom at last sex in past 12 mo^d^	1,021/2,988 (34%)	795/2,776 (29%)	1.19 (0.91,1.54)	541/2,832 (19%)	407/2,775 (15%)	1.27 (0.97,1.67)
Used condom at last sex in past 12 mo with non-regular partner^e^	903/1,821 (50%)	760/1,746 (44%)	1.15 (0.97,1.36)	189/427 (45%)	136/434 (31%)	1.34 (1.07,1.69)
Ever used modern contraceptive^f^	2,232 (59%)	1,911 (55%)	1.09 (0.94,1.26)	1,561 (48%)	1,371 (42%)	1.11 (0.95,1.30)
Used modern contraceptive at last sex^d^,^f^	1,040/2,992 (35%)	803/2,781 (29%)	1.21 (0.92,1.58)	632/2,841 (22%)	538/2,796 (18%)	1.16 (0.91,1.47)
>1 partner in same time period in past 12 mo	1,087 (29%)	1,132 (32%)	0.90 (0.76,1.06)	209 (6%)	219 (7%)	0.87 (0.63,1.20)
>1 partner in past 4 wk	435 (11%)	464 (13%)	0.87 (0.65,1.15)	57 (2%)	53 (2%)	1.04 (0.66,1.66)

a Denominators vary depending on missing values and unless specified have the following ranges: Male intervention 3,786–3,807; male comparison 3,473–3,493; female intervention 3,256–3,276; female comparison 3,220–3,238.

b Adjusted for: Age group (<21, 21–22, 23–24, ≥25 y), stratum, ethnic group (Sukuma versus non-Sukuma).

c % with all three responses “correct.”

d Among those who reported having had sex in past 12 mo.

e Among those who reported having ever had sex with a non-regular partner in past 12 mo.

f Modern contraceptive  =  condom, oral contraceptive pill, injectable contraceptives.

The median reported age at sexual debut was 18 and 17 y in males, and 17 y in females in the intervention and comparison communities, respectively (Table 2). Overall, 37% of males in intervention communities reported >4 lifetime sexual partners compared to 44% males in the comparison communities (aPR 0.87, 95%CI 0.78–0.97), but similar prevalences by trial arm were seen for other measures of reported partner change and concurrency (Table 3). There was little evidence of an increase in reported condom or modern contraceptive use among men in the intervention communities, but stronger evidence that reported condom use with the most recent non-regular partner was higher among females in intervention communities (aPR 1.34, 95%CI 1.07–1.69) (Table 3).

Genital ulcers were reported less frequently by both sexes in the intervention communities (males aPR 0.76, 95%CI 0.59–−0.99; females aPR 0.69, 95%CI 0.47–1.01) (Table 4). In respondents who reported having STI symptoms within the last 12 mo, there was no significant difference by trial arm in reported use of health facilities for their most recent STI symptoms. There was no evidence of differences between arms in the outcomes related to pregnancy (Table 4).

**Table 4:** Impact of intervention on clinical and biological outcomes by sex in 2007/8.

Outcome	Males			Females		
	Prevalence^a^		aPR^b^ (CI)	Prevalence^a^		aPR^b^ (CI)
	Intervention (N = 3807) n (%)	Comparison (N = 3493) n (%)		Intervention (N = 3276) n (%)	Comparison (N = 3238) n (%)	
**Reported clinical/biological outcomes**						
Genital discharge (last 12 mo)	288 (8%)	320 (9%)	0.83 (0.63,1.09)	122 (4%)	178 (6%)	0.70 (0.45,1.09)
Genital ulcer (last 12 mo)	193 (5%)	245 (7%)	0.76 (0.59,0.99)	149 (5%)	216 (7%)	0.69 (0.47,1.01)
Went to health facility for most recent STI symptoms within past 12 mo^c^	192/401 (48%)	195/451 (43%)	1.19 (0.91,1.56)	102/216 (47%)	154/326 (47%)	1.02 (0.77,1.37)
>2 reported pregnancy (lifetime)^d^	207 (5%)	220 (6%)	0.95 (0.70,1.29)	587 (18%)	605 (19%)	0.96 (0.80,1.15)
Reported pregnancy while in primary school^d^	113 (3%)	132 (4%)	0.84 (0.57, 1.23)	102 (3%)	91 (3%)	1.16 (0.68,1.97)
Reported ≥1 unplanned pregnancy^d^	675 (39%)	782 (47%)	0.87 (0.69,1.10)	792 (25%)	759 (24%)	1.03 (0.83,1.26)
**Primary biological outcomes**						
HIV prevalence	74 (2.0%)	59 (1.7%)	0.91 (0.50,1.65)	126 (3.9%)	136 (4.2%)	1.07 (0.68,1.67)
HSV-2 prevalence	948 (25.0%)	928 (26.7%)	0.94 (0.77,1.15)	1,313 (40.3%)	1,369 (42.5%)	0.96 (0.87,1.06)
**Secondary biological outcomes**						
Syphilis seroprevalence (TPPA+)	218 (5.8%)	183 (5.3%)	1.06 (0.74,1.52)	206 (6.3%)	241 (7.5%)	0.86 (0.62,1.21)
Active syphilis prevalence (TPPA+, RPR+)	144 (3.8%)	113 (3.3%)	1.11 (0.72,1.72)	147 (4.5%)	167 (5.2%)	0.91 (0.65,1.28)
Chlamydia prevalence	80 (2.1%)	73 (2.1%)	1.24 (0.66,2.33)	85 (2.6%)	69 (2.1%)	1.27 (0.87,1.86)
Gonorrhoea prevalence	13 (0.3%)	15 (0.4%)	0.71 (0.21,2.41)	11 (0.3%)	12 (0.4%)	0.73 (0.20,2.63)

a Denominators vary depending on missing values and unless specified have the following ranges: Male intervention 3,786–3,807; male comparison 3,473–3,493; female intervention 3,256–3,276; female comparison 3,220–3,238.

b Adjusted for: Age group (<21, 21–22, 23–24, ≥25 y), stratum, ethnic group (Sukuma versus non-Sukuma).

c Among those reporting STI symptoms (genital discharge or genital ulcer) within past 12 mo.

d For males, reported times they had made a girl or woman pregnant, pregnant in primary school, or have an unplanned pregnancy.

**Figure 4:**
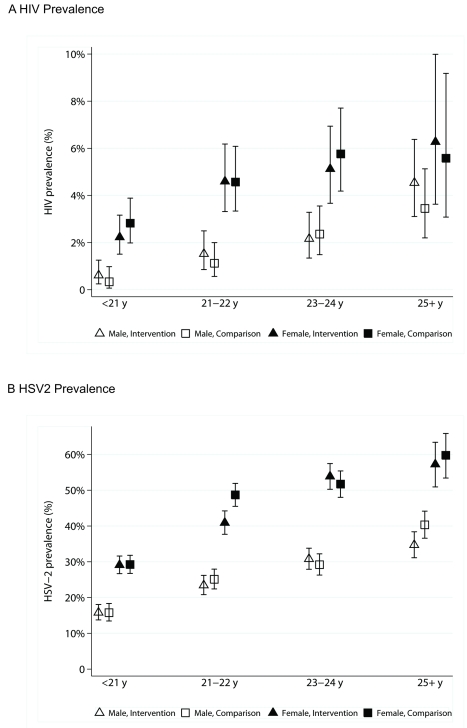
HIV and HSV-2 prevalence and 95% confidence intervals, by sex, age group, and arm of trial.

The prevalence of the primary trial outcomes, HIV and HSV-2, in the comparison communities were similar to those predicted at 1.7% and 26.7%, respectively, in males, and 4.2% and 42.5%, respectively, in females (Table 4). Figure 4 shows HIV and HSV-2 prevalence by age, sex, and trial arm. There was no significant difference in prevalence by trial arm either for HIV (males aPR 0.91, 95%CI 0.50–1.65; females aPR 1.07, 95%CI 0.68–1.67) or for HSV-2 (males aPR 0.94, 95%CI 0.77–1.15; females aPR 0.96, 95%CI 0.87–1.06). Similarly, prevalences of the secondary biological outcomes—syphilis, chlamydia, and gonorrhoea—were similar in the two arms (Table 4).

## Discussion

The trial results demonstrate that the MEMA kwa Vijana intervention led to a sustained improvement in young people's sexual and reproductive health (SRH) knowledge and some reported sexual behaviours. The lack of any significant impact on the prevalence of HIV and other STIs either after 3 years or after more than 8 years of interventions being in place, indicates that skills-based, in-school education, linked to more youth-friendly health services and limited supportive community activities, while important in improving young people's knowledge of how to reduce their sexual risk, may not be sufficient to reduce HIV incidence and other biological outcomes among young people in this setting.

The 2007/8 survey was carried out in communities in which nine consecutive cohorts of young people had been exposed to the MEMA kwa Vijana intervention, thus increasing the chances for the interventions to have influenced community norms among young people. The inclusion of young people from six of these year groups in the survey (those who had been in years 5–7 between 1999 and 2002 when the interventions were known to have been implemented with high coverage and fidelity) increased the chances that both male and female sexual partners could have been exposed to the intervention. 67% of the young people interviewed in the intervention communities in the 2007/8 survey had had a chance of being exposed to the full 3 years of the in-school component of the intervention, compared with only 26% during the 2001/2 survey.

The intervention was associated with higher levels of SRH knowledge, although the relative risk was not as strong as that observed in 2001/2 (Table 5). An increase in knowledge was observed in both trial arms between 2001/2 and 2007/8. Improvements in HIV knowledge in the comparison communities may have resulted from exposure to national media campaigns, including recent campaigns encouraging voluntary counselling and testing for HIV, exposure to HIV information at antenatal care or marriage preparation, and the roll-out of antiretroviral treatment. The increases in knowledge about pregnancy prevention and STIs may be due to the older age of the respondents and their personal experiences with pregnancy and/or STIs. Improvements in knowledge in the young people in the comparison communities will have decreased the chances of finding differences in knowledge by trial arm, making it even more impressive that such differences were still observed an average of 5.4 y after the young people had left primary school.

**Table 5:** Impact of intervention on selected outcomes by sex in 2001/2 and 2007/8.

Outcome	Males						Females					
	2001/2			2007/8			2001/2			2007/8		
	I	C	aRR^a^ (CI)	I	C	aPR^a^ (CI)	I	C	aRR^a^ (CI)	I	C	aPR^a^ (CI)
**Knowledge** b												
HIV acquisition	65%	45%	1.44 (1.25,1.67)	73%	66%	1.11 (0.99,1.23)	58%	40%	1.41 (1.14,1.75)	68%	61%	1.11 (1.00,1.24)
STD acquisition	52%	40%	1.28 (1.07,0.54)	54%	46%	1.18 (1.04,1.34)	36%	25%	1.41 (1.06,1.88)	38%	30%	1.24 (0.97,1.58)
Pregnancy prevention	84%	50%	1.66 (1.55,1.78)	83%	69%	1.19 (1.12,1.26)	72%	46%	1.58 (1.26,1.99)	71%	60%	1.17 (1.06,1.30)
**Reported attitudes** b												
Attitudes to sex	22%	12%	1.77 (1.42,2.22)	28%	22%	1.31 (0.97,1.77)	27%	19%	1.42 (1.11,1.81)	11%	10%	1.09 (0.67,1.77)
**Reported sexual behaviour**												
Sexual debut during follow-up^c^	60%	72%	0.84 (0.71,1.01)			—	68%	67%	1.03 (0.91,1.16)			—
Age at first sex <16 y			—	25%	28%	0.91 (0.80,1.05)			—	28%	27%	1.01 (0.80,1.28)
>1 partner in last 12 mo	19%	28%	0.69 (0.49,0.95)	41%	45%	0.92 (0.79,1.08)	9%	8%	1.04 (0.58,1.89)	10%	10%	0.97 (0.76,1.23)
Used condom at last sex^d^	29%	20%	1.47 (1.12,1.93)			—	27%	22%	1.12 (0.85,1.48)			—
Used condom at last sex in past 12 mo^e^			—	34%	29%	1.19 (0.91,1.54)			—	19%	15%	1.27 (0.97,1.67)
Went to health facility for most recent STI symptoms within past 12 mo^f^	29%	35%	0.84 (0.50,1.41)	48%	43%	1.19 (0.91,1.56)	36%	34%	1.02 (0.62,1.70)	47%	47%	1.02 (0.77,1.37)
**Primary biological outcomes**												
HIV incidence (/1,000py)	0.43	0.3	NA			—	3.2	4.7	0.75 (0.34,1.66)			—
HIV prevalence			—	2.0%	1.7%	0.91 (0.50,1.65)			—	3.9%	4.2%	1.07 (0.68,1.67)
HSV-2 prevalence	11.3%	12.5%	0.92 (0.69,1.22)	25.0%	26.7%	0.94 (0.77,1.15)	21.3%	20.8%	1.05 (0.83,1.32)	40.3%	42.5%	0.96 (0.87,1.06)

a Adjusted for: Age group (2001/2: ≤17, 18, ≥19 y at 2001/2 survey; 2007/8: <21, 21-22, 23–24, ≥25 y at 2007/8 survey), stratum, ethnic group (Sukuma vs non-Sukuma). 2001/2 also adjusted for number of lifetime partners at baseline (0, 1, 2, ≥3).

b % with all 3 responses “correct.”

c Among those who reported never having had sex at recruitment in 1998.

d Among those who reported having had sex at the 2001/2 survey.

e Among those who reported having had sex in past 12 mo.

f Among those reporting STI symptoms (genital discharge or genital ulcer) within past 12 mo.

C, comparison; I, intervention; NA, Number of cases too small to justify comparison (<10 in each group); —, not measured.

In both surveys, the proportion of young people answering all three attitudinal questions desirably was <30% in both sexes and both trial arms. These questions focused mainly on gender norms, suggesting that the intervention did not have a major impact on such norms.

In terms of sexual behaviour, strong evidence of intervention impact was seen only on the number of sexual partners among males, and condom use among sexually active females with their last non-regular partner. Overall, the intervention appears to have had less impact on reported sexual behaviour in the 2007/8 survey than in the 2001/2 survey. One potential explanation may be that the length of time since exposure to the in-school intervention led to an attenuation of intervention effect. Another is that when young people are older and/or have left primary school their sexual behaviour is more influenced by community norms. Alternatively, as the young people interviewed in 2007/8 were older and exposed to the intervention many years previously, responses may have been more honest and less subject to differential reporting bias by trial arm.

The lack of impact, in either direction, on biological outcomes an average of 8.9 y after the start of the intervention tends to contradict the frequently held belief that positive changes in knowledge, reported attitudes, and reported behaviours will eventually lead to a reduction in HIV, STIs, and unwanted pregnancies. A direct comparison between overall prevalences in the various survey rounds is not appropriate because the ages of the young people included differed, the median ages in the 1998, 2001/02, and 2007/08 surveys being 15, 18, and 22 y, respectively.

One explanation for the lack of impact could have been weaknesses in the design or implementation of the intervention itself. However, external evaluations of the intervention design and materials concluded that it was theoretically sound and of high quality. Also, internal and external process evaluations conducted between 1999 and 2002 demonstrated that the interventions were delivered to a high standard and that coverage was high.

The rural communities included in the trial were geographically separated from each other. Migration in the area is usually to larger towns, often to seek work, or to neighbouring villages, such as when a woman gets married. It was, therefore, unlikely that there was significant spill-over of the intervention into the comparison communities. Qualitative data collected in 1999–2002 and more recently in 2007/8 suggest that there was little other governmental or non-governmental organisation SRH intervention activity in the trial communities. Similarly, between 1999 and 2005 there was only a minimal amount of SRH education included in the national curriculum for primary schools in the comparison communities [36]. It is unlikely that the introduction of interventions into primary schools and health facilities in comparison communities between 2005 and 2007 had any important effect on the sexual and reproductive health of survey respondents who had all left primary school by that time.

Three other African [12],[13],[16] studies have measured the impact of ASRH interventions on biological outcomes and generally their findings have not been promising. This present study is a valuable complement to these three studies. The MEMA kwa Vijana trial evaluated the long-term impact of an intervention that had been subjected to careful, theory-based design and pilot testing, and for which process evaluations had shown that it had been implemented with high coverage and good fidelity [23]. The cluster randomised trial design means that significant differences in the outcomes between trial arms were likely due to the intervention effects. This study is unique in having such a long follow-up period and as such should have been able to detect change in behaviours resulting from exposure of consecutive cohorts of young people, such as changes within age-mixed relationships.

The evaluation of the trial had several limitations. The study population was likely to have been, on average, at lower risk of HIV and other STIs compared to other rural populations for two main reasons. First, it was restricted to young people who had reached at least year 5 of primary school. A preliminary, population-based survey in the trial communities showed that HIV was more prevalent in 15- to 19-y-olds who had never been to school or who had left school before school year 5 [3]. Second, despite repeat visits to the trial communities and tracing of young people to major migration points and local secondary schools, we are likely to have missed many of those attending secondary school outside the trial communities, those who migrated outside the study area for employment or marriage, and mobile groups such as fishermen, miners, or traders. As elsewhere, studies in the Mwanza Region have shown that mobile young people are at increased risk of HIV and other STIs [37]. On the other hand, the study population might have been more amenable to behaviour change because of their better education. Although it would have been ideal to measure HIV incidence as a primary outcome in the 2007/8 survey, this was not possible as no baseline data were available on several of the school year-groups included.

Exposure to the MEMA kwa Vijana intervention did not increase risk-taking among youth, and significant differences in ASRH knowledge persisted in the 2007/8 survey when the young people had last been exposed to the in-school intervention an average of 5.4 y previously. The results of this trial show that such skills-based sexual health education interventions and youth-friendly health services can make a valuable contribution towards the UN General Assembly Special Session on HIV/AIDS goal [38] of increasing young people's access to the information, skills, and services they need to reduce their vulnerability to HIV. However, these results imply that such interventions, on their own, will not be sufficient to reduce HIV and other STIs among young people in sub-Saharan Africa. Qualitative work carried out in the trial communities in 1999–2002 found that many young people were not always in a position to use the knowledge and skills obtained through MEMA kwa Vijana [24],[39],[40]. Peer pressure to be sexually active, and widespread attitudes and practices in the broader community such as negative attitudes to condoms, material exchange for sex, and older male–younger female relationships, may have posed too great a challenge for youth who wanted to reduce their risk behaviours. This suggests that additional interventions are needed to address broader sexual norms and expectations. Efforts to design, implement, and rigorously evaluate behaviour change interventions among adults as well as young people, with strong support from political leaders, are urgently needed.

## Supporting Information

Text S1 Protocol for the long term evaluation of the MEMA kwa Vijana intervention.
(3.76 MB PDF)

Text S2 CONSORT checklist.
(0.09 MB PDF)

## Abbreviations

aPR: adjusted prevalence ratio
IQR: interquartile range
[A]SRH: [adolescent] sexual and reproductive health
STI: sexually transmitted infection

## References

[1] UNAIDS (2008). Report on the global HIV/AIDS epidemic 2008..

[2] Temmerman M (1994). Sexually transmitted diseases and reproductive health.. Sex Transm Dis.

[3] Obasi AI, Balira R, Todd J, Ross DA, Changalucha J (2001). Prevalence of HIV and Chlamydia trachomatis infection in 15-19-year olds in rural Tanzania.. Trop Med Int Health.

[4] Pequegnat W, Stover E (2000). Behavioral prevention is today's AIDS vaccine!. AIDS.

[5] UNICEF (2002). Young people and HIV/AIDS: Opportunity in crisis..

[6] UNAIDS (2007). Practical Guidelines for Intensifying HIV Prevention: Towards Universal Access..

[7] WHOUNAIDS (2007). New data on male circumcision and HIV prevention: policy and programme implications: WHO/UNAIDS Technical Consultation, Male Circumcision and HIV Prevention: Research Implications for Policy and Programming, Montreux, 6–7 March 2007:conclusions and recommendations..

[8] Weiss HA, Halperin D, Bailey RC, Hayes RJ, Schmid G (2008). Male circumcision for HIV prevention: from evidence to action?. AIDS.

[9] Henderson M, Wight D, Raab GM, Abraham C, Parkes A (2007). Impact of a theoretically based sex education programme (SHARE) delivered by teachers on NHS registered conceptions and terminations: final results of cluster randomised trial.. BMJ.

[10] DiCenso A, Guyatt G, Willan A, Griffith L (2002). Interventions to reduce unintended pregnancies among adolescents: systematic review of randomised controlled trials.. BMJ.

[11] Gallant M, Maticka-Tyndale E (2004). School-based HIV prevention programmes for African youth.. Soc Sci Med.

[12] Pronyk PM, Hargreaves JR, Kim JC, Morison LA, Phetla G (2006). Effect of a structural intervention for the prevention of intimate-partner violence and HIV in rural South Africa: a cluster randomised trial.. Lancet.

[13] Jewkes R, Nduna M, Levin J, Jama N, Dunkle K (2008). Impact of stepping stones on incidence of HIV and HSV-2 and sexual behaviour in rural South Africa: cluster randomised controlled trial.. BMJ.

[14] Stephenson J, Strange V, Allen E, Copas A, Johnson A (2008). The long-term effects of a peer-led sex education programme (RIPPLE): a cluster randomised trial in schools in England.. PLoS Med.

[15] Kirby D, Obasi A, Laris BA, Ross DA, Dick B, Ferguson J (2006). The effectiveness of sex education and HIV education interventions in schools in developing countries..

[16] Cowan FM, Pascoe SJ, Langhaug LF, Dirawo J, Chidiya S (2008). The Regai Dzive Shiri Project: a cluster randomised controlled trial to determine the effectiveness of a multi-component community-based HIV prevention intervention for rural youth in Zimbabwe–study design and baseline results.. Trop Med Int Health.

[17] Catania JA, Gibson DR, Chitwood DD, Coates TJ (1990). Methodological problems in AIDS behavioral research: influences on measurement error and participation bias in studies of sexual behavior.. Psychol Bull.

[18] McFarlane M, St Lawrence JS (1999). Adolescents' recall of sexual behavior: consistency of self-report and effect of variations in recall duration.. J Adolesc Health.

[19] Wight D, West P (1999). Poor recall, misunderstandings and embarrassment: interpreting discrepancies in young men's reported heterosexual behaviour.. Cult Health Sex.

[20] Plummer ML, Ross DA, Wight D, Changalucha J, Mshana G (2004). “A bit more truthful”: the validity of adolescent sexual behaviour data collected in rural northern Tanzania using five methods.. Sex Transm Infect.

[21] Hayes RJ, Changalucha J, Ross DA, Gavyole A, Todd J (2005). The MEMA kwa Vijana project: design of a community randomised trial of an innovative adolescent sexual health intervention in rural Tanzania.. Contemp Clin Trials.

[22] Obasi AI, Cleophas B, Ross DA, Chima KL, Mmassy G (2006). Rationale and design of the MEMA kwa Vijana adolescent sexual and reproductive health intervention in Mwanza Region, Tanzania.. AIDS Care.

[23] Ross DA, Changalucha J, Obasi AI, Todd J, Plummer ML (2007). Biological and behavioural impact of an adolescent sexual health intervention in Tanzania: a community-randomized trial.. AIDS.

[24] Plummer ML, Wight D, Obasi AIN, Wamoyi J, Mshana G (2007). A process evaluation of a school-based adolescent sexual health intervention in rural Tanzania: the MEMA kwa Vijana programme.. Health Educ Res.

[25] Guyon A, Lugoe WL, Ferguson J (2000). Evaluation report of HIV/AIDS peer education in the MEMA kwa Vijana project, Mwanza, Tanzania.. AMREF, NIMR and LSHTM Collaborative Research Projects.

[26] Kirby D (2001). The Mema kwa Vijana Curriculum: A Review..

[27] Lugoe WL (2001). Evaluation of Teacher-training sessions for MkV teacher led component..

[28] Renju J, Nyalali K (2008). Scaling up ASRH education through widespread teacher training: training evaluation report.. National Institute for Medical Research, Mwanza, Tanzania and Liverpool School of Tropical Medicine, UK.

[29] Renju J, Nyalali K (2008). Scaling up Youth Friendly health services through widespread health worker training; training evaluation report.. National Institute for Medical Research, Mwanza, Tanzania and Liverpool School of Tropical Medicine, UK.

[30] Boerma JT, Urassa M, Nnko S, Ng'weshemi J, Isingo R (2002). Sociodemographic context of the AIDS epidemic in a rural area in Tanzania with a focus on people's mobility and marriage.. Sex Transm Infect.

[31] Rossau R, Heyndrickx L, Van Heuverswyn H (1988). Nucleotide sequence of a 16S ribosomal RNA gene from Neisseria gonorrhoeae.. Nucleic Acids Res.

[32] Roymans R, Onland G, Jansz A, Quint W, Boel E (1999). Evaluation of an in-house polymerase chain reaction for detection of Neisseria gonorrhoeae in urogenital samples.. J Clin Pathol.

[33] Grosskurth H, Mosha F, Todd J, Mwijarubi E, Klokke A (1995). Impact of improved treatment of sexually transmitted diseases on HIV infection in rural Tanzania: randomised controlled trial.. Lancet.

[34] del Mar Pujades Rodriguez M, Obasi A, Mosha F, Todd J, Brown D (2002). Herpes simplex virus type 2 infection increases HIV incidence: a prospective study in rural Tanzania.. AIDS.

[35] Hayes RJ, Moulton LH (2009). Cluster Randomised Trials..

[36] Makokha M (2008). What MEMA kwa Vijana has to offer the education sector AIDS response in Tanzania: A comparative review.. National Institute for Medical Research, Medical Research Council, UK and Liverpool School of Tropical Medicine, UK.

[37] Mwita W, White R, Chilongani J, Orroth K, Zaba B (2002). The association between population mobility and HIV prevalence in rural villages in Mwanza region, Tanzania.. Trans R Soc Trop Med Hyg.

[38] United Nations (2001). UNGASS Goals..

[39] Wight D, Plummer ML, Mshana G, Wamoyi J, Shigongo ZS (2006). Contradictory sexual norms and expectations for young people in rural Northern Tanzania.. Soc Sci Med.

[40] Plummer ML, Wight D, Wamoyi J, Mshana G, Hayes RJ (2006). Farming with your hoe in a sack: condom attitudes, access, and use in rural Tanzania.. Stud Fam Plann.

