# Supplementary material for: Long-Term Biological and Behavioural Impact of an Adolescent Sexual Health Intervention in Tanzania: Follow-up Survey of the Community-Based MEMA kwa Vijana Trial
Source: PLoS Med. 2010 Jun 8;7(6):e1000287. doi: 10.1371/journal.pmed.1000287 (PMC2882431; doi:10.1371/journal.pmed.1000287)
Supplement: Text S1 — Protocol for the long term evaluation of the MEMA kwa Vijana intervention. (3.76 MB PDF) [file pmed.1000287.s001.pdf]

MkV1FS_Protocol_Final.doc

Protocol for MEMA kwa Vijana Trial Further Survey

Table of Contents

List of Abbreviations ... 3
List of Definitions ... 3
Summary ... 4
1. Background ... 4
1.1 Introduction ... 4
1.2 MEMA kwa Vijana Intervention ... 5
1.3 MEMA kwa Vijana Community Randomised Trial ... 5
1.4 Developments since the end of the trial ... 6
1.5 Rationale for further follow-up survey ... 6
1.6 Aim ... 7
1.7 Hypothesis to be tested ... 7
1.8 Trial outcomes ... 7
1.9 Study Design ... 8
Figure 1.1. Cohort diagram showing those eligible for the survey (2007/2008)- age distribution ... 9
1.10 Sample size and Power ... 10
Box 1.1 Sample Size Calculation ... 10
Table 1.1 Power to detect true sizes of effect for various outcomes ... 11
2. Preparations at the National Institute for Medical Research ... 12
2.1 Memorandum of Understanding ... 12
2.2 Official permissions and clearances ... 12
2.3 Procurement/ Rental of vehicles ... 12
2.4 Staff recruitment ... 12
2.5 Standard Operating Procedures ... 13
2.6 Training of Field Staff ... 13
2.7 Pilot study ... 13
2.8 Additional materials to be prepared at NIMR prior to the survey ... 13
2.8.1 Lists for Registration Interviewer ... 13
2.8.2 Maps of study area and study communities ... 14
2.8.3 Survey timetables ... 14
Table 2.1 Order of Communities for MkV1 FS ... 15
2.8.4 Questionnaires and Forms ... 15
2.8.5 Additional field materials ... 16
3. Study Procedures ... 16
3.1 Mobilisation ... 16
3.1.1 Regional Level ... 16
3.1.2 District Level ... 16
3.1.3 Ward level ... 17
3.1.4 Village level ... 18
3.1.5 Mobilisation report and supervision ... 19
3.2 Census ... 20
3.2.1 Pre-census preparations ... 20

3.2.2 Census 21
Table 3.1 Estimated time taken to carry out census 21
3.2.3 Generation of lists for survey team 22
3.3 Survey 23
3.3.1 Pre-survey preparations 23
Table 3.2 Estimated time taken to carry out survey 24
3.3.2 Registration 25
3.3.3 Face-to-face interview 26
3.3.4 Laboratory 26
3.3.5 Clinician 26
3.3.6 Voluntary counselling and testing for HIV (VCT) 27
3.3.7 Checking of questionnaire 28
3.3.8 Survey Venue Flowchart 28
Figure 3.1: Survey flowchart 29
3.3.9 Participant Incentives 30
Table 3.3: Incentives for Survey Attendees 30
3.3.10 Tracing 30
3.3.11 Team Debrief 30
3.4 Census and Survey reporting and Field staff supervision 31
3.5 Other field activities 32
3.5.1 Specimen collection 32
3.5.2 Data collection 32
3.5.3 Post-survey mopping up 32
3.5.4 Post-survey STI treatment 32
3.6 Logistics 33
3.6.1 Accommodation 33
3.6.2 Personnel Identification 33
3.6.3 Project Vehicles 33
3.6.4 Money for fieldwork 33
3.6.5 Communications 33
3.6.6 Timeline 33
4. Data management 34
5. Laboratory analysis 34
7. Communication and Dissemination 34
8. References 35
9. Annexes .... **Error! Bookmark not defined.**

MkV1FS_Protocol_Final.doc

List of Abbreviations

ASRH	Adolescent Sexual and Reproductive Health
CDE- Inspector	Chief District Education Inspector
CT	Chlamydia trachomatis
DC	District Commissioner
DAC	District AIDS Coordinator
DAS	District Administrative Secretary
DCDO	District Community Development Officer
DCI	Development Co-operation Ireland (now called Irish Aid)
DED	District Executive Director
DEO	District Education Officer
DFID	UK Department for International Development
DPLO	District Planning Officer
EC	European Commission
GPS	Geographical Positioning System
HIV	Human Immunodeficiency Virus
HSV2	Herpes Simplex Virus 2
IA	Irish Aid
MDG	Millennium Development Goals
MEMA	Mpango wa Elimu na Maadili ya Afya kwa Vijana
MkV1	MEMA kwa Vijana Trial
MkV1 FS	MEMA kwa Vijana Trial Further Survey
MkV2	MEMA kwa Vijana larger-scale implementation and operations research
MITU	Mwanza Intervention Trials Unit
MRC	Medical Research Council
NG	Neisseria gonorrhoeae
NIMR	National Institute for Medical Research
PDA	Personal Data Assisistant
RAS	Regional Administrative Secretary
RCT	Randomised Controlled Trial
SRH	Sexual and Reproductive Health
STI	Sexually Transmitted Infection
TA	Technical Advisor
UNAIDS	Joint United Nations Programme on HIV/AIDS
UNGASS	United Nations General Assembly Special Session
VCT	Voluntary Counselling and Testing for HIV
WEC	Ward Education Coordinator
WEO	Ward Executive Officer

List of Definitions

Attendee	Young person who attends survey (eligible or non-eligible)
Participant	Young person who is eligible and who participates in the survey
Invited young person	Potentially eligible young person who has been given an invitation to attend the survey

3

MkV1FS_Protocol_Final.doc

Summary

The overall purpose of the MEMA kwa Vijana 1 Trial Further Survey (MkV1 FS) is to evaluate the impact of the MkV 1 intervention on the sexual and reproductive health of young people.

The MkV1 FS will be split into 3 phases:

- ***Mobilisation phase***: The MkV1 Further Survey (Reasons for survey, aims and procedures) will be presented to National, Regional, District and Ward officials. Mobilisation will also involve visits to each of the survey villages. During these visits community members will have the opportunity to hear about the survey activities and to ask questions.
- ***Census phase***: During a household census, young people who are eligible to participate in the survey will be identified and invited to participate in the survey.
- ***Survey phase***: Eligible young people will be interviewed about their sexual and reproductive health using a face-to-face questionnaire and will be asked to provide blood and urine samples for testing for HIV and other sexually transmitted infections (STIs). Participants will also be offered syndromic treatment of STIs and Voluntary Counselling and Testing (VCT) for HIV.

1. Background

1.1 Introduction

UNAIDS estimate that, in 2004[1] , over 60% of all new HIV infections occurred in sub-Saharan Africa. About half of these new infections were in youth aged 15-24 years, in whom rates of STI, and unintended pregnancies are also very high. In the absence of a vaccine or cure, behavioural interventions are the main strategy for HIV control, especially amongst youth. Even in the worst affected countries, the prevalence of both HIV and STIs is very low in 15 year olds, but rise steeply after that. If effective interventions that focus on adolescents can be found, they will make a very substantial impact on the HIV epidemic. However, the limited evidence of the effectiveness of behavioural interventions is mainly from developed countries, and is contradictory.

Very few interventions have been rigorously evaluated in the developing world, where the need is greatest. For example, a review carried out in 2004 could only identify 11 school-based HIV prevention programmes in sub-Saharan Africa that had been evaluated using experimental or quasi-experimental designs[2]. Almost all of the programmes had been able to demonstrate an improvement in knowledge about sexual health issues; most showed an improvement in some reported attitudes; but most studies either reported no changes in reported sexual behaviours or changes that were only present in sub-groups. None had evaluated intervention impact on biomedical outcomes, and none had evaluated impact beyond 24 months follow-up, with most (8/11) having their final evaluation within 6 months. Yet evaluation of biological outcomes is critically important because of the limited validity of reported sexual behaviour among young people [3-5], and because of the considerable potential for interventions to differentially bias reported behaviour towards more “desirable” behaviours in the intervention arm. It is also crucially important to know whether interventions will only have transient effects, or affect behaviour and hence HIV incidence for many years. The recent review of the evidence on the effectiveness of interventions to reduce HIV and to meet the HIV-related UNGASS and Millennium

Development Goals related to young people, 'Steady, Ready, Go', has highlighted the lack of such rigorous studies with biological outcomes [6] Governments and donor agencies are therefore forced to invest in "best guess" interventions, without the benefit of evidence of their effectiveness to reduce HIV incidence.

1.2 MEMA kwa Vijana Intervention

The MEMA kwa Vijana ("Good things for young people") intervention is a sexual and reproductive health (SRH) programme for young people in rural Mwanza, Tanzania.

The intervention is implemented by AMREF and the Ministries of Health and of Education and Culture through existing government structures, and was specifically designed to be sustainable and replicable in resource-constrained settings. During the period 1999-2002 (MkV1) it had four major components:

(i)In-school sexual and reproductive health education in years (standards) 5, 6 and 7 of primary schools through a teacher-led, peer-assisted programme of participatory lessons that include the use of drama, stories and games. The curriculum includes the key characteristics that have been found to be important for effective in-school programmes in terms of behaviour change in previous studies [7] .

(ii) Youth-friendly sexual and reproductive health services, through training of the health workers in government health facilities on how to provide attractive and effective sexual & reproductive health services for youth.

(iii) Community-based condom promotion and distribution, for and by youth (only in 2000-2001 of MkV1).

(iv) Community-wide activities to create a supportive environment for the adolescent sexual health interventions; these included a one-week initial community mobilisation, the creation of a ward-level committee to support and supervise the project activities, an annual youth health week and twice-yearly youth health days at the government health facilities.

1.3 MEMA kwa Vijana Community Randomised Trial

The MEMA kwa Vijana trial (MkV1) is the first randomised controlled trial (RCT) anywhere in the world that has measured biological outcomes to assess the effectiveness of an adolescent sexual and reproductive health (ASRH) intervention in preventing HIV and other STIs. The MkV1 trial was funded by the European Commission (EC), Development Cooperation Ireland (DCI), and the UK Medical Research Council (MRC), with additional support from UNAIDS and UK DFID. Starting in January 1999, 20 communities (each roughly equivalent to an administrative "ward") scattered across Sengerema, Geita, Kwimba and Missungwi districts of Mwanza Region were randomly allocated either to receive (10 intervention communities with a total of 58 primary schools and 18 health facilities) or not to receive (10 comparison communities with a total of 63 primary schools and 21 health facilities) the MkV intervention.

During the period from January 1999 to March 2002, the intervention's impact on HIV, other STIs, unintended pregnancies, reported attitudes and sexual behaviour, and on

SRH knowledge was evaluated within a cohort of 9,645 adolescents. At enrolment in late 1998, the cohort were all aged at least 14 years (>95% aged between 14 and 18 years) and were in years 4-6 of primary school. A **baseline survey** was carried out at cohort recruitment in 1998, with an **interim survey** in 2000, and **3-year follow-up survey** in Oct 2001- April 2002.

The results of the 3-year evaluation showed that the MkV1 intervention had led to a highly significant improvement in knowledge, reported attitudes and some, but not all, reported behavioural outcomes, especially among males. The results of the evaluation of biological outcomes were inconclusive, at least partly because, by that time, the trial lacked the statistical power to detect effects of public health importance on HIV and some of the other STIs measured. There were only 5 HIV seroconversions in males and 40 in females, in whom the adjusted rate ratio was 0.75 (95% CI: 0.34, 1.66). Overall HSV2 prevalence at 3-year follow-up was 11.9% in males and 21.1% in females, with adjusted prevalence ratios of 0.92 (95% CI: 0.69, 1.22) and 1.05 (95% CI: 0.83, 1.32), respectively. There was no consistent impact on other biological outcomes, though there was a significantly higher prevalence of Neisseria gonorrhoeae (NG) in females in intervention communities (***Annex 2: Results of 3-year MkV1 evaluation survey***).

1.4 Developments since the end of the trial

Between 2002 and 2005 the in-school sexual and reproductive health education, support and supervision for the youth-friendly sexual and reproductive health services, and the community-wide activities have been maintained in the 10 intervention communities only, with support from Irish Aid (formerly DCI). In 2006, MEMA kwa Vijana Phase 2 intervention started in the comparison communities as part of an Irish Aid (IA)-supported district-wide expansion of the programme throughout the four project districts (MkV2). The MkV2 intervention is very similar to MkV1 intervention though it does not include the condom promotion component. This expansion has been accompanied by operations research, policy research and formative research in order to develop additional community components to the intervention. The MkV2 intervention primarily targets those adolescents (mainly aged between 13 and 16 years of age) who are in the last three years of primary school.

1.5 Rationale for further follow-up survey

One of the potential explanations for the lack of any consistent impact on the biological outcomes during the first 3-years of the MkV1 trial, despite the substantial differences in knowledge, reported attitudes and reported sexual behaviours, is that the interventions may have needed more time to work. The follow-up period of three years that was available for the original phase of the trial led to the following unavoidable limitations:

1. The highest risk group (School Year 6 at recruitment) represented 40% of the trial cohort, but received only one year of the in-school component.

2. There is a substantial average difference in the age of males and females in sexual partnerships in this population. The older male sexual partners of the young women in the cohort had not been exposed to the in-school component and, conversely, some of the sexual partners of the young men in the cohort may have been too young to have been exposed to the in-school component. Given the power differentials between men and women, it may be the case that both

partners, or at least the male partner, need to have been exposed to the interventions before substantial behavioural changes take place.

3. Relatively low HIV incidence in the school-going trial cohort resulted in lower than projected power to detect an impact of the interventions on HIV incidence.

A further follow-up survey in 2007/8 will take place in order to evaluate the longer-term impact of the MkV1 intervention. The **MkV1 Further Survey (MkV1 FS)** has several advantages relative to the earlier phase of the trial. Firstly, participants in five of the six school year groups in the intervention communities will have had the opportunity to receive at least 2 years of in-school intervention. Secondly, many of the male partners of the young women in the survey will have previously received the MEMA kwa Vijana in-school component, and this may be translated into stronger effects in these women. The mean HIV prevalence in this older population will be higher and the study will, therefore, have a greatly enhanced power to detect differences in HIV prevalence. Members of the original trial cohort will be included, and so the long-term effects on knowledge, attitudes and other frequent outcomes can be measured in this subgroup.

Those eligible to participate in the MkV1 further survey will have left primary school by the end of 2004 (NB if repeated std 5, 6 or 7 then may have left primary school after 2004) (Figure 1.1). Young people in the comparison communities who have been exposed to MkV2 in-school component of the intervention will not be included.

This survey will be carried out 8 years after the introduction of the MkV1 intervention and will be able to examine the impact of this intervention in the long-term, when it has had the chance to have affected several consecutive cohorts of young people. The survey will, however, retain the advantages of the original community randomised controlled trial design. This proposal exploits a unique window of opportunity to directly evaluate the longer-term effectiveness of behavioural interventions on HIV and STIs and builds on the considerable knowledge and experience that has been acquired during the earlier phases of the trial.

1.6 Aim

To investigate whether there is a significant long-term impact of the MEMA kwa Vijana (MkV1) intervention on the sexual health of young people.

1.7 Hypothesis to be tested

That in the longer-term the MEMA kwa Vijana (MkV1) Intervention leads to an improvement in sexual and reproductive health.

1.8 Trial outcomes

Primary outcomes will be:

- HIV prevalence and HSV2 antibody prevalence

Secondary outcomes will be:

- Prevalence of Syphilis, Chlamydia trachomatis (CT) and Neisseria gonorrhoeae (NG)

- Three sexual health knowledge scores and one sexual health attitudes score (each score based on 3 questions, as used in the previous MkV surveys)
- Reported sexual behaviours, including: sexual debut, lifetime number of sexual partners, no. different sexual partners in past 12 months, condom use with last non-regular partner, use of other contraceptives at last sexual intercourse.
- Reported lifetime number of pregnancies

1.9 Study Design

A cross-sectional survey of young people living in the 20 MkV1 trial communities (10 intervention, 10 comparison).

The specific groups who will be invited to participate in the survey are shown in **Figure 1.1**. In summary, the inclusion criteria will be:

- Currently considered to be *de jure* member of a household within one of the 20 trial communities (allocation to Intervention and comparison arms described in Annex 3). A de jure member of a household is someone who currently sleeps or has previously slept on a regular basis in the household (‘Kaya’).

- Attended standard 5,6 or 7 in a primary school within a trial community for at least one year between 1999 and 2002 inclusive (the period when the intervention was implemented most intensively and with closest supervision)

- Willing to give informed consent to all the study procedures

Figure 1.1. Cohort diagram showing those eligible for the survey (2007/2008)- age distribution

- The potential **number of years of exposure** to the MkV in-school component of intervention, by the end of the calendar year, for those in the 10 intervention communities is represented by the number in each cell. The number of years of exposure will be '0' for the young people who will be invited to participate in the 10 comparison communities (not shown).
- The **school year groups** which include those eligible for the original MkV1 **trial cohorts** are highlighted in yellow. The other school year groups who had the potential to receive at least one year of the in-school intervention during the period when this was being implemented most intensively (ie. 1999-2002 inclusive) are shown in light blue. The further follow-up survey will be carried out in the second half of 2007 and will include both these school year groups (cross-hatched in row for the end of 2007).
- The mean and age ranges are based on an extrapolation from the baseline data (1998) for std 4, 5 and 6 at that time- excluded ages where <1% of school year group had that age.
- The school year groups that have been exposed to the MkV2 intervention in both the intervention AND comparison communities are indicated in pink. MkV2 intervention started in 25% of the schools in the 4 trial districts mid-2005. A further 50% of the schools started in Oct 2006 and the last 25% of schools are due to start MkV2 interventions in May 2007.

**Activity**		**Primary School**				**Years since left school**								
	**Year**	**Std 4**	**Std 5**	**Std 6**	**Std7**	**+1**	**+2**	**+3**	**+4**	**+5**	**+6**	**+7**	**+8**	**+9**
**Recruitment survey (Aug-Dec '98)**	**Dec 1998**	0	0	0										
Intervention started Jan '99	**Dec 1999**		1	1	1									
	**Dec 2000**		1	2	2	1								
**3-year evaluation Survey**	**Dec 2001**		1	2	3	2	1							
	**Dec 2002**		1	2	3	3	2	1						
	**Dec 2003**		1	2	3	3	3	2	1					
	**Dec 2004**		1	2	3	3	3	3	2	1				
	**Dec 2005**		1	2	3	3	3	3	3	2	1			
	**Dec 2006**		1	2	3	3	3	3	3	3	2	1		
**8-year evaluation Survey**	**Dec 2007**		1	2	3	3	3	3	3	3	3	2	1	
**Mean age in late 2007 (range) for each schl yrcohort (yrs)- FEMALE**		13 (10,16)	14 (11,17)	15 (12,18)	16 (13,19)	17 (14,20)	18 (15,21)	19 (16,22)	20 (17,23)	21 (18,24)	22 (19, 25)	23 (20, 26)	24 (21, 27)	25 (22, 28)
**Mean age in late 2007 (range) for each schl yr cohort (yrs)- MALES**		14 (11,18)	15 (12,19)	16 (13,20)	17 (14,21)	18 (15,22)	19 (16,23)	20 (17,24)	21 (18,25)	22 (19,26)	23 (20, 27)	24 (21, 28)	25 (22, 29)	26 (23, 30)

1.10 Sample size and Power

The population who are eligible for this survey is limited (ie must have attended the trial primary schools during the selected time period) and so the sample size of 14, 520 was calculated (Box 1.1) based on the number of eligible young people who:

(i) Are aged 17-25 years (See NOTE below)
(ii) Can be traced during the census and attend the survey
(iii) Agree to participate

Box 1.1 Sample Size Calculation

We estimate that there will be an average of 720 men and 720 women from each community who had, between 1999 and 2002, completed at least 1 of the final 3 years of primary school in that community. Based on data from the original enrolment survey for the trial, we predict that 90% of these young people will be aged 17-25 years at the end of 2006. We also estimate that 70% of these young adults will still be living in those communities and will be registered during the census, and that 80% will agree to participate in the further follow-up survey. The total sample size will therefore be **14,520** (726 interviewed per community x 20 communities).

The power of the study to detect true reduction in the prevalence of biological outcomes was calculated separately for men and women assuming 365 men and 365 women per community, 10 communities per arm,and k=0.2, where k is the coefficient of variation between communities for that outcome (***Table 1.1***). The prevalences of all the other endpoints are expected to be greater than 25%, so the study will have greater power for these endpoints.

NOTE: The inclusion/exclusion criteria have now been revised and there is no longer an age restriction i.e. eligible young people of all ages can participate in the survey.

We had previously estimated that 10% of the young people who attended the appropriate years in trial schools would be excluded on age and by removing the age restriction we would expect an increase in the size of the eligible population. However, given the uncertainty around the estimates of the number of eligible young people who can be traced during the census and/or the number who will agree to participate we have made no change to the sample-size.

Despite previous work, the greatest uncertainty is in the HIV prevalence. Using our best estimates, we predict that the study will have adequate power (79%) to detect a 35% reduction in HIV prevalence in females, but only a moderate power (64%) to detect a 40% reduction in males. Similarly, the study will have good power to detect differences of 35% in syphilis and of 25% in HSV2, in each sex. If similar effects are found in the two sexes, it will be possible to combine the results from the two sexes to give greater power. Subgroup analyses for the commoner outcomes such as HSV2, knowledge, attitudes and behaviours will include the impact by age group, number of years of in-school intervention received, and among those who were original MkV cohort members.

Table 1.1 Power to detect true sizes of effect for various outcomes

Outcome	Sex	Prevalence (%) in comparison community[1]	Size of effect[2]			
			40%	35%	30%	25%
HIV	Males	1.0	0.40	0.31	0.24	0.17
		2.0	0.64	0.51	0.39	0.28
		2.3	0.69	0.56	0.43	0.31
	Females	3.0	0.77	0.64	0.50	0.36
		4.0	0.85	0.73	0.58	0.43
		4.5	0.88	0.76	0.62	0.46
		5.0	0.90	0.79	0.65	0.48
		6.0	0.93	0.83	0.69	0.52
		7.0	0.95	0.86	0.73	0.56
Syphilis	Males	6.5	0.94	0.85	0.71	0.54
	Females	10.0	0.97	0.92	0.80	0.63
HSV2	Males	25.0	1.00	0.98	0.91	0.77
	Females	35.0	1.00	0.98	0.93	0.80

Key:
[1] Estimated using prevalence and incidence estimates from other studies in Mwanza Region
[2] Difference in prevalence between intervention and comparison communities
□ Best estimates of the prevalence of each outcome.

2. Preparations at the National Institute for Medical Research

2.1 Memorandum of Understanding

A Memorandum of Understanding (MoU) will be signed between London School of Hygiene & Tropical Medicine (LSHTM) and Mwanza Intervention Trials Unit (MITU) /National Institute for Medical Research (NIMR), Mwanza. Office and storage space at NIMR, Mwanza will be allocated to the survey.

2.2 Official permissions and clearances

Research and Ethics Clearance for the MkV1 FS has already been obtained from the Government of the United Republic of Tanzania through the Medical Research Coordination Committee (MRCC) and from the LSHTM Ethics Committee.

Before MkV1 FS activities start in Mwanza Region, senior programme staff will visit the Regional Administrative Secretary (RAS), the Regional Education Officer (REO) and the Regional Medical Officer (RMO) to introduce the project. They will request the RAS to write a letter introducing MkV1 FS to the District Executive Directors (DED) and the District Executive Officers (DEO) in each of the 4 MkV1 districts.

In each of the four MkV1 FS Districts, senior MkV1 FS staff will organise a forum to inform invited district representatives about the survey and to allow the districts to give their feedback on the proposed plans. Permission to carry out survey activities in their district and letters of introduction to the Ward Executive Officer will be requested.

District Forums will be followed by a series of meetings at the ward and village level during which community members will have an opportunity to hear about the survey and ask questions.

2.3 Procurement/ Rental of vehicles

IT equipment and clinical/medical supplies that are not available locally in Tanzania will be purchased in the UK and shipped to Mwanza. All other equipment (including vehicles) will be purchased locally in Tanzania. Lease agreements will be signed for Landcruiser and minibus hire.

2.4 Staff recruitment

All positions on the MkV1 FS team that cannot be filled by existing MITU/NIMR staff will be advertised in East African Newspapers (senior positions) and/or on local notice boards. Senior MkV1 FS and MITU/NIMR staff will shortlist candidates based on pre-defined person specifications and job descriptions. Tests and interviews will be held at NIMR, Mwanza in front of a panel of MkV1 FS and MITU/NIMR staff. The majority of staff will be employed for a period of 12 months on standard NIMR/AMREF/LSHTM Collaborative project contracts. Contracts include a 3-month probation period and standard terms and conditions i.e. allowances for annual, sickness, maternity (paternity) and compassionate leave etc.

Senior MkV1 FS staff (Fieldwork Manager and Fieldwork Supervisor and technical field staff (Clinicians, Counsellors, Laboratory Technicians, Drivers) will be recruited and trained in February 2007 and March 2007 respectively. A large number of fieldworkers are needed (interviewers, census workers, tracers) and assessment for suitability for the posts will be based initially on application letter and CV. A selection of applicants will be

invited to the first week of training in April 2007. Only those who do well on the written test at the end of this week and who perform well during group activities will be invited to participate in the second week of training. The final composition of the two field teams will be decided at the end of the second week of training. The third and final week of training will involve a pilot study. Throughout the survey, there will be ongoing supervision and on the job training.

2.5 Standard Operating Procedures

A detailed description of all the procedures that must be followed at each step of the survey (field, data and laboratory) will be written. These procedures will be revised following the pilot study and, if necessary, during the survey itself.

2.6 Training of Field Staff

Preparation and training for all field staff will take place from 23rd April 2007 to 25th May 2007. The first week of training will focus on the survey protocol and main questionnaire and the second week will focus on use of the Personal Data Assistants (PDA) and Geographical Positioning Systems (GPS) for the census (***Annex 4: Draft Field Team Training Schedule***). In addition, clinical, laboratory and counselling staff will have short-term placements at health facilities/laboratories in Mwanza and will spend some time observing staff on other research projects (HSV2 and Mwamko).

2.7 Pilot study

Each of the two field teams will have a third week of training which will include a pilot study in Nassa Ginery, Magu District. The aim of the pilot study will be to test the materials and questionnaires, and the procedures for mobilisation, registration, interviewing and collecting the data. The pilot study will include a census to find eligible young people, generation of a list of survey participants, setting up of a survey centre, collection and transport of laboratory specimens. Pilot data will be entered and analysed, and the results will be used for additional team training and finalisation of the survey instruments.

2.8 Additional materials to be prepared at NIMR prior to the survey

2.8.1 Lists for Registration Interviewer

- **Lists of those who were invited to the survey during the census (List A)**

The templates for these lists (List A1-A3) will be prepared at NIMR and lists will be generated in the field using the information downloaded from the census interviewers' PDA (see section 3.2.3).

- **List of MkV Trial primary schools with school codes (Annex 5: Codes for wards, villages and schools) (List B)**

- **Lists of young people who were exposed to MkV1 intervention between 1999 and 2002 (and control community equivalents) (List C and D)**

Lists of MkV1 trial cohort members will be prepared using MkV1 trial cohort data. An official request for copies of standard 7 exam results will be made to Mwanza Region Education Office.

C1: List of MkV1 Trial male cohort members
This will be a list of those who were enrolled during the baseline survey and will include those who participated in the MkV1 trial impact evaluation cohort ie born before 1st January 1985 (blue MkV1 ID card) and those born after 1st January 1985 (white MkV1 ID card).

C2: List of MkV1 Trial female cohort members (as C1 above)

D1: List of Std 7 exam results (2002)
This is an official list of those who completed std 7 of primary school in 2002 that can be obtained from the Regional and/or District Education Office. The list will be sorted by sex within each primary school.

D2: List of Std 7 exam results (2003) (as D1 above)

D3: List of Std 7 exam results (2004) (as D1 above)

2.8.2 Maps of study area and study communities

An official request will be made of the Regional Planning Authority for an up-to-date map of Mwanza Region. This map will be used to plan and monitor the survey.

2.8.3 Survey timetables

The MkV1 FS will use a slightly different order of communities to the MkV 2001/02 survey (**Table 2.1**). A provisional detailed timetable of activities in each of the communities will be provided as information to District and ward officials and will be used to book survey venues, accommodation and transport for fieldteams **(Annex 6: Draft timetable for community))**.

Table 2.1 Order of Communities for MkV1 FS

Order of communities	Community name & number		Community intervention status & stratum	
	Team 1	Team 2	Team 1	Team 2
1.	Katunguru (5)	Koromije (22)	I1	C1
2.	Kasamwa (11)	Mwagi (23)	I1	C1
3.	Nyang'whale (14)	Malya (24)	C2	I2
4.	Bukoli (13)	Misasi (18)	C1	I1
5.	Katoro (8)	Usagara (21)	I2	C2
6.	Kagu (9)	Nyakaliro (4)	I3	C3
7.	Lubanga (10)	Katwe (2)	C3	I3
8.	Busisi (6)	Nyehunge (3)	C2	I2
9.	Nkome (1)	Fukalo (17)	C3	I3
10.	Ihanamilo (12)	Hungumalwa (16)	I2	C2

2.8.4 Questionnaires and Forms

Questionnaires and forms will be based on materials used during MkV1, other surveys conducted in Mwanza and other surveys measuring sexual and reproductive health. Questionnaires and Forms will be designed in Microsoft Word.

Initially, all questionnaires and forms will be drafted in English and will then, where appropriate, be translated into Swahili and Sukuma and back translated into English. All questionnaires and forms will undergo a series of pre-tests and revisions. Final drafts will be used during the pilot study and revised before the survey if necessary.

The following questionnaires will be needed by the census and survey team (in English and Swahili/ Sukuma):

- Census questionnaire (programmed into PDA and back-up paper copy) **(Annex 7 a-d)**
- Main questionnaire **(Annex 8 a-e)**
- Main questionnaire- quality control **(Annex 9 a-b)**

A random 10% of participants will answer quality control questionnaires and a list of pre-selected sticker numbers will be prepared for the survey teamsby the data section**.**

Other field forms are described in the text below (Annex 10-54) and additional forms not mentioned are included in the Annexes (Annex 55-66).

2.8.5 Additional field materials

Equipment and materials needed by the field teams e.g. car stickers, staff t-shirts, numbered stickers and items are detailed in the team packing lists (Annex 10a-k) and will be obtained locally. Items not available locally will be obtained from the UK.

3. Study Procedures

3.1 Mobilisation

Two mobilisation officers will carry out mobilisation activities according to the mobilisation officer standard operating procedures **(Annex 11a)**

3.1.1 Regional Level

Senior members of the team will visit the Mwanza Regional Administrative Secretary (RAS) to explain the rationale, aims and procedures of the MkV1 Further Survey. They will request permission to carry out survey activities in Mwanza Region (including pilot work) and if permission is obtained will also request letters of introduction addressed to the DED in each of the survey Districts (Geita, Sengerema, Missungwi, Kwimba) and the districts involved in pilot work (Magu, Mwanza city). These introductory letters will indicate to the Districts that the Region has given its consent and support to the MkV1 Further Survey.

A request will be made to the Regional Education Officer for permission to access and photocopy standard 7 exam results for the years 2002-2004.

3.1.2 District Level

The initial mobilisation will be done by senior members of the field survey team (Mobilisation Officer (MO) accompanied by either Project Co-ordinator (PC), Fieldwork Manager (FM) or Fieldwork Supervisor (FS)) who will travel by car to all study Districts capitals. They will spend 2-3 days in each District and will be responsible for introductions and permissions and other pre-survey preparations at the district level.

The MO, accompanied by at least one senior MkV1 FS staff member, will visit all districts to deliver the letter of introduction from the RAS and to ask the DED for permission to carry-out survey activities in their District. The possibility of having an MkV1 FS information forum for district officials will also be discussed. Targeted district officials include the DC, DAS, DED, DEO, DMO, DPLO, DCDO/DAC, CDE-Inspector, Chair person- District Council, and the MkV TA (AMREF). The venue and date for the forum will be agreed during this visit and the DPLO will be requested to distribute invitations to the agreed list of participants.

The MkV1 FS information forum will be held during one half day at a suitable venue in the District capital and will involve about 15 people from the district and 2 –3 members of survey team ***(Annex 12:District Forum Mobilisation Schedule).*** Following a brief presentation on MKV1FS, the participants will be provided with the provisional survey

timetable **(Annex 13: Order of communities for District)**, survey information sheet for officials **(Annex 14a-b)** and will be given an opportunity to ask questions. During these forums district officials will be paid seating allowance of Tshs. 10,000/= per person (20,000/= for DC, DAS & DED) and refreshments (chai) will be provided.

Following the meeting, the DED will be requested to write introductory letters to the leaders in each of the MkV1 FS communities (wards) in their District. These letters will indicate the districts' consent and support for the research. The MO will follow-up and ensure that these letters are collected from the DED.

The Mobilisation Officer (MO) will visit the District Medical Officer (DMO) in each District to explain that MkV1 FS will be offering syndromic management for STIs for participants and will be providing contact slips for partners. The district hospitals and health facilities should therefore expect a small increase in the number of people attending for STI treatment. The MO should also discuss the referral system for participants who test positive for HIV.

When mobilisation in the district (or part of district) has been completed, one or both MOs should visit the DED and debrief him/her about the mobilisation activities in that District. He/she should remind the District Officials that the census and survey are about to start in that district (refer to survey timetable).

3.1.3 Ward level

The MO will travel to each of the MkV1 FS wards two to three weeks before the census team are due to arrive in that ward. The MO will introduce him/herself to the WEO, and deliver the introductory letter from the District, the information sheet for officials **(Annex 14a-b)** and the provisional survey timetable for that community (ward) **(Annex 6).** He/she will discuss with the WEO the possibility of holding an MkV1 FS information forum with a similar format to the district-level forum **(Annex 15 a-b: WDC Presentation)**. This forum should involve the WEO, WEC, Councillor, VEOs, VCs, HC/in-charge (WDC), and representatives from the religious community and government representatives. Those attending the forum will receive a sitting allowance of 5000 Tsh each. A Transport allowance of 1000 Tsh will be paid to those who travel from outside the ward capital (must be more than 1 hour walking distance from forum venue).

The MO will make a request to the WEO for a complete list of the Vitongoji (sub-village leaders) in each of the survey communities. The MO will also request the WEO to provide a letter of introduction to the village leaders to be distributed as follows: 1 copy to each Village Chairperson (VC), 1 copy to each Village Executive Officer (VEO), 1 copy to remain at Ward and 1 copy for MO (12-16 copies depending on the number of villages in that ward). The MO will assist the WEO in this task by providing him/her with the draft letter from Ward to Village leaders **(Annex 16 a-b: Letter of introduction from ward to villages)** that has been pre-prepared in Mwanza. Following the information forum the MO assigned to that ward will carry-out routine mobilisation activities at Ward and Village level. MOs will travel on motorcycle, and spend approximately ten days in each ward.

The MO should meet with any home based care groups working in the ward and discuss with them the process for referrals for HIV treatment and other support for those who test positive for HIV. He/she should also visit local health facilities to tell them about the

survey and to discuss the possibility that there will be an increase in people seeking treatment for STIs and/or family planning.

Before leaving the community, the MO should visit the WEO and debrief him/her about the exercise and remind him/her that the census and survey are about to start in that ward (refer to survey timetable).

3.1.4 Village level

When arriving in each village the MO will present him/herself to the Village executive officer (VEO), village chairman (VC) and sub-village /Vitongoji leaders. The MO will introduce the proposed survey (objectives and plans for the next few months) and leave copies of the information sheet **(Annex 14a-b)** and the draft village survey timetable **(Annex 6)**. The MO will ask these leaders to help him/her to identify potential venues for the survey e.g. rented houses, guesthouses, schools (during school holidays only),go downs. Guesthouses should only be used if there are no other suitable survey venues. The MO will discuss possible venues with the FS or FM and together they will decide on the most appropriate venue. The MO will then book the venue for the survey and also the accommodation for the fieldteams (both census and survey). The MO will use the survey timetable to identify the days that the venue/accommodation are needed. A contract must be signed between the MO and owner of survey venue/ accommodation to document the agreed price and conditions of hire **(Annex 17a-b: Letter for venue/accommodation agreement)**. The MO is responsible for ensuring that the survey venue is equipped for the survey team eg arrange chairs/benches (for 60 people), tables (at least 7), water supply, toilet facilities etc

The MO will also visit all head teachers of the schools, as well as some religious leaders, traditional healers and other influential individuals eg health NGOs working in the area, as seems appropriate.

The MO will request each of the Kitongoji (sub-village) leaders to list the households in their sub-village. The MO should explain in detail why the lists of households are needed and provide forms to be used **(Annex 18 a-d: List of Household Head Form)**. The MO should show the Kitongoji leader how the form should be completed. In addition to listing the household heads, the kitongoji leaders should indicate the households with young people aged 15-30 years (estimate most of the young people eligible for the survey will be aged 18-26 years). Each Kitongoji leader will be requested to mobilise the sub-village members so that they are prepared for the arrival of the census team on the dates specified in the survey timetable. During mobilisation the kitongoji leaders should distribute the MkV1 FS information sheet for household head **(Annex 19 a-b: Information Sheet for Household Head)** and the survey poster to all households **(Annex 20: Survey Information Poster)**. He/she should indicate on the list of households which households have received this information. This form should be completed using carbon paper so that the Kitongoji leader can keep one copy. When the MO returns to the kitongoji to collect the completed list of HH (~ 2 days later), he/she will check on the progress of the mobilisation. Mobilisation by the Kitongoji leader is estimated to take 2 days and if the Kitongoji leader has carried out the tasks assigned to him then he/she will be paid 5,000 Tsh (2,500 Tsh/day).

The MO will inform the vitongoji leaders that the census team leader will ask them to participate in house-to-house sensitisation and to help census interviewer during the census. During this task (house-to house census) they will be given an allowance of Tsh

3,000/= per day. If the Kitongoji has more than 30 households ask Kitongoji leader to arrange one census helper (will also receive Tsh 3,000/= per day). The Community helpers will be paid a daily rate and will not be paid per number of households visited.

3.1.5 Mobilisation report and supervision

The day after the MO returns to Mwanza, he/she should meet with the FS and/or FM to debrief them on ward mobilisation. The MO should also submit a brief written report to the MkV1 FS team within 4 days of returning from the ward. The report should contain information on villages/sub-villages visited and include names and contact details (Mobile phone numbers) of key individuals and groups eg Vitongoji leaders, school and health facility staff, home based care groups. The report should also provide information on the selected survey venues and field team accommodation **(Annex 21: Mobilisation Report Form)**. The MO will make a photocopy of each list of households prepared by the Vitongoji and leave the original in the MkV1 FS office and give the other copy to the Census Team Leader. The information on villages/sub-villages will then be used by the PC, FM and/or FS to produce the final survey timetable for that ward visited.

On the first few visits to the wards the MO will be accompanied by a senior member of project staff and will travel by car (nearest wards e.g. Katunguru and Koromije will be purposely selected to make this feasible). During the survey the FS will be in contact by telephone with the MO every week. If the MO encounters any difficulties he/she should contact the FS and/or FM as often as required.

3.2 Census

Following mobilisation, a census will be conducted in each of the survey communities in order to identify young people eligible to participate in the survey. A community (~ward) has a radius of 5-10 km, a population of approximately 18,000 and is made up of approx. 6 villages. There will be two census teams and each census team will work in 10 communities. Each team will comprise of a census team leader (survey deputy team leader) and 8-10 census interviewers and will travel in a project landcruiser. The census team leader (CTL) and census interviewers (CI) will work according to their standard operating procedures **(Annex 11 b-d)**

3.2.1 Pre-census preparations

With help and supervision from senior MkV1 FS staff, and in collaboration with other census team members, the Census Team Leader (CTL) should make sure that all logistic issues are taken care of and that the census team are ready to go to the field.

The day after the census team arrive in each Ward (Community) they will introduce themselves to the WEO, WEC and other ward officials and let them know that the survey is about to start in the ward. They will provide officials with an updated survey timetable indicating the days that census and survey teams will be in each village and sub-village.

The census team leader (CTL) will then travel to the nearest village (usually near the ward capital) and introduce him/herself to the village executive officer, the village chairperson and the Vitongoji leaders. (*The Mobilisation Officer will phone the leaders of this first village a few days before to prepare them for the visit of the census team).* He/she will remind them of the survey procedures, inform them that the census will start in their village the next day and provide them with the updated survey timetable. The Vitongoji leaders will be requested to mobilise the residents of their sub-village so that as many of them as possible are present in the sub-village on the day that the census team will visit. They will be encouraged to use the list of HH to help them with this mobilisation.

The CTL will also ask the Vitongoji leaders help to arrange for community helpers to assist the census workers during the census (Kitongoji leader should be one of the helpers). Whenever possible, the CTL should meet with local helpers and explain in detail the purpose and procedures of the census and ensure that the community helpers know their responsibilities during the exercise and amount of payment). These community helpers (CH) will receive 3000 Tsh/ day. Before leaving the kitongoji the CTL will make sure that each “census helper” received his or her incentives/money.

While census interviewers (CI) are carrying-out the census in one village/sub-village, the CTL will arrange a pre-census visit to the next village/sub-village as per census schedule (one day before). He/she will do all preparatory activities as explained above.

When the census starts in a village the census team leader (CTL) will go to the guesthouses/ houses that will be used in that village for the survey and ensure that they are ready for the survey team who will arrive 2-4 days later. The CTL will also visit the survey venue a day or two before the survey team arrive in a village in order to ensure that everything is ready for the survey team.

The CTL will also complete the Census village information sheet with the help of leaders in the community **(Annex 22: Census village information sheet)**.

3.2.2 Census

The CI will travel to the study site in a Land cruiser (one Land cruiser/team) and will then travel from household to household on foot or by bicycle (project bicycle or hired during the exercise). Each of the 10 CI will work 48 hours/week (8 hours/day for 6 days) and will need to interview approximately 150 households/week each (approx. 20 mins/HH; 25 HH/day/CI) (Table 3.1). A number of additional days will be needed in each village as some of the households will need to be visited more than once. It should take 3 weeks to complete the census in each community. In order to work more efficiently, the CTL may decide to assign each of the sub-villages in a village to a sub-group of CI.

Table 3.1 Estimated time taken to carry out census

	**Estimated number of Households (HH)**	**Days taken by team of 8-10 Census Interviewers (CI)**
		200-250 HH/day*
**Community**	**~ 3000**<br>(365 eligible M, 365 eligible F)	**12- 15 days**
**Village**	~ 500<br>(60 eligible M, 60 eligible F)	2-2.5 days

**25 HH/day/CI (1 HH interview in 20 mins, 8 hours work)*

Using the list of household heads prepared by the Kitongoji for the MO, a CI will copy the information on the households onto their CI form for HH **(Annex 23:CI form for household head)**. Using this form, they will go with their community helper (CH) to each of these households. If the CI happens to find some additional household(s) in the Kitongoji, which are not included in the list prepared before, CI will add the household to the list of additional household heads (Annex 18c-d).

In each household (HH) the CI and Community Helper (CH) will briefly introduce themselves to the head of HH or member of the HH and explain briefly the aim of census. The respondent can be the HH head or another member of the HH**.** Informed consent will be obtained from the respondent prior to questioning **(Annex 24a-b:Household head consent form).**

Each CI will use a hand-help computer (PDA) to directly enter information on household members aged 15-30 years. Eligibility to participate in the survey will be assessed immediately. Eligible young people will be given an appointment 2-4 days later to attend the survey that will be held in a nearby rented building (4-8 venues per study community). If the eligible young person is not present at the time of the census, the other household members will be asked to give the survey invitation to them. The survey invitation **(Annex 25a-b: Invitation to attend the main survey)** will contain the following information:

1. Location and time of the survey interview

2. Description of survey procedures, stressing that confidentiality will be maintained
3. Details of travel expenses and incentives that will be paid
4. Request to bring to the survey: invitation, any health survey ID cards and any official documents showing date of birth or age [MkV1 ID cards: ID number, name, sex, age, date of enrolment, ward, village]

If the eligible young person is <18 years of age then the parent or guardian will be given an additional information sheet explaining the survey procedures **(Annex 26a-b: Information sheet about main survey for Parent/guardian**) and the parent/guardian will be asked to sign a sheet to indicate that they consent for the young person to participate in the survey **(Annex 27a-b: Parent/Guardian consent form)**.

If the household members indicate that the eligible young person will not be able to attend the survey then the CI will record the details of their whereabouts on a moved away form **(Annex 28a-b: Moved away form)**.

Each CI will also have geographical positioning system (GPS) equipment that will allow them to record the exact location of the household and enter this location into the PDA. This information will be used to help locate individual households at a later date eg by tracers or for STI treatment and/or to map the location of the study participants for presentation and further analysis.

If there are any technical problems that prevent the CI using the PDA to record the census data then the CI must use the back-up paper census form (Annex 7a-d).

3.2.3 Generation of lists for survey team

Each evening, the CTL will be responsible for downloading the census data from the PDAs into the laptop. Each evening the CTL will make a copy of the data collected that day onto a blank CD. He/She will be responsible for the storage and safety of the census equipment (i.e. laptop, PDAs/GPS, chargers) and make sure that all equipment are ready for the next day (charged) **(Annex 29: PDA and GPS borrowing form)**. The CTL will, with the help of the CI, complete the census team daily progress report form **(Annex 30:CT daily progress report form)**.

If the paper back-up census questionnaire was used then, in the evening, the CTL with the assistance of the CI will enter the data into an Access Database.

When the census team have completed all the households in one village (~ every 3 days) the CTL will generate and print lists of eligible young people (Lists A1- A3). Lists will be delivered to the survey team (one day before survey).

A1: List of males identified during the MkV1 FS census

List A1 will be ordered by appointment date and then by first name and will contain the following variables: Name of young person (first, surname, other), appointment date, sex, Census ID number, village, sub-village. This list will also have space for the registration interviewer to enter date of survey, registration interviewer ID code and will have space for a sticker.

A2: **List of females identified during the MkV1 FS census** (As A1 above)

A3: List of Household heads
This list will be sorted by village and then by census ID number and will contain names of young person, sex, names of household heads, sub-village and GPS location.

At the end of every village the CTL will complete the census village summary form (Annex 31: Census Village Summary Form) and at the end of the community will complete the census community report form (Annex 32: Census community summary form).

3.3 Survey

There will be two survey teams with 22 team members each:

Team Leader (1)
Clinical Officer (1)
Counsellors (2)
Tracers (5)
Attendee Co-ordinator (1)
Registration interviewers (2)
Interviewers (3 male, 3 female)
Lab workers (2)
Data Checker (1)
Driver (1)

3.3.1 Pre-survey preparations

At least one week before the field trip the team should check that they have all required equipment, documents and other supplies. If there are any problems with the equipment then they must inform the STL. With help and supervision from senior MkV1 FS staff, and in collaboration with other survey team members, the Survey Team Leader (STL) should make sure that all logistic issues are taken care of and that the survey team are ready to go to the field

The day before travelling, the team must double-check that they have all equipment/supplies that are needed. They should also attend the ST briefing meeting with the Mobilisation Officer (MO), Field Supervisor (FS) and Fieldwork Manager AND the rest of the ST. During this meeting the MO will give you any important information about the community.

On the day of departure, the team will leave NIMR, Mwanza early in the morning so as to arrive in the survey community (ward capital) in the afternoon/early evening. **All travel to the field must start at NIMR, Mwanza.**

Each survey team and their equipment will travel from Mwanza to the survey communities in a hired bus (at least 22 seater bus). This bus will be hired only on the specific days that the survey team travel from one community to the next. Each survey team will also have a project land cruiser that will transport them and their equipment from village to village within a community.

23

When the Survey Team Leader (STL) arrives in the community he/she should meet the CTL and discuss progress with the census and any issues that are important for the survey. The CTL will provide the STL with printed copies of the list of those invited to the survey **(Lists A1-A3)**.

The CTL and STL will make brief visits to the WEO, WEC and other ward officials to provide an update on the progress of the census and remind the officials about the survey timetable and procedures. The STL will then visit leaders in the first village (including Vitongoji leaders) to introduce the survey team and finalise arrangements for the survey.

The CTL and STL will keep in regular contact (by text/ phone) about the survey venue and accommodation for fieldteams.

Each team will arrive in a village approx. 2-4 days after their respective census team and will set up a survey centre in the pre-booked guesthouse or house. The survey centre will have a registration and waiting area (may be outside in a shaded area), 6 rooms for face-to-face interviews, 1 room for the lab technicians, 2 rooms for the VCT counsellors and 1 room for the clinician (10 rooms + registration/waiting area).

The day after arriving in the community (Day 1) one member of each section of the survey team must go to the survey venue with the STL. The STL will allocate an area/ room for each staff member.

We estimate that there will be ~ 14,500 eligible young adults who will turn up at the interview sites. We anticipate that one team can interview 48 participants /day. It will take approximately 15 days to interview the 730 eligible males and females in a community. The team will spend a number of extra days in each community so that they can interview those who were unable to attend the survey at the designated time 'mop-up'. (Table 3.2).

Table 3.2 Estimated time taken to carry out survey

	**Estimated number of eligible young people**	**Days taken by survey team**
		48 people/day*
**Community**	**~ 730**<br>(365 eligible M, 365 eligible F)	**15 days**
**Village**	~ 122	2.5 days

** 4 males and 4 females / hour, 8 hours work*

Invited young people will make their own way to the survey centre on the day and time specified on the survey invitation. The census team will aim to invite 64 young people at 08:00 everyday. When the invitees arrive they will be greeted by the attendee co-ordinator **(Annex 11h)** who will take them to the registration interviewers (RI). The RI will greet attendees and record the date and time of interview, attendees name, village, sub-village and name of household head in the registration book. They will then be shown to the waiting area where they will be provided with an information sheet **(Annex**

33 a-b: Information Sheet for Participants) and a Walkman containing a recording of information on the project **(Annex 34: Walkman Lending Form)**. One of the team members will show attendees how they can play, pause and stop the recording. When an attendee has read the information sheet and/or listened to the Walkman, he/she will be interviewed by the Registration Interviewer.

3.3.2 Registration

There will be two registration interviewers; one for female attendees and one for male attendees and they will follow out the tasks detailed in the registration interviewer standard operating procedures (Annex 11e). Following reading of the information sheet and listening to the Walkman, the RI will interview the attendee and complete section A of the main questionnaire (Annex 8 a-b). The registration interviewer will verify the identification of the attendee by asking them for their survey invitation and/or by finding their name on the census list (A1-A2). They will enter their census ID number into the registration book. The RI will use lists B or C to help them to determine the eligibility of the attendee. If the young person is a member of the MkV1 cohort then the RI will indicate so in the column ‘MkV1’ in the registration book.

If an attendee is eligible then RI will ask the attendee a series of questions to determine if they are able to give informed consent **(Annex 35a-b: Informed Consent Questions)**. If the attendee is not able to give informed consent then they should return to the waiting area until one of the team members (STL, counsellor or RI) can discuss with them in more detail what the survey will entail. When the team member is confident that the attendee is able to give informed consent, the attendee will return to the RI and will be asked if they consent to participate in the survey. If consent is given then they must sign or thumbprint the informed consent sheet **(Annex 36 a-b).** The RI will place one sticker **(ST1)** with a unique survey ID number on the consent form, a second sticker **(ST2)** with the same number beside their name in the registration book and if their name is on the census list (List A1 or A2) then another sticker **(ST3)** will be placed beside their name on this list. The fourth, fifth and sixth stickers will be placed on sections A-C of the main questionnaire **(ST4-6).**
Note:

(1) Attendees will also be asked if they consent to receiving follow-up treatment for any STIs that is diagnosed after the day of the survey. If they consent to participate in the survey but would not like follow-up treatment then they are still eligible to participate in the survey.
(2) If an attendee is unable to give informed consent following discussion with a survey team member then they are deemed not eligible to participate in the survey but may access clinician and VCT services.

All eligible consenting attendees (‘participants’) will be given a plastic folder containing their main questionnaire and additional stickers **(ST7- 21)**. They will then wait in queue for their turn to be interviewed by a survey interviewer.

The registration book and list A1/A2 with stickers attached must be kept securely by the STL until the team returns to Mwanza.

3.3.3 Face-to-face interview

Eligible consenting attendees will then be interviewed using a face-to-face questionnaire (Annex 8 c-d: Main Questionnaire- Section B) by a survey interviewer (SI) of same sex and similar age. SI will follow the survey interviewer standard operating procedures **(Annex 11f).** The interviews must take place in a private place, and the completed questionnaires kept safe at all times. After the interview is complete, the main questionnaire should be returned to the participant and they should be directed to the Laboratory Technician.

3.3.4 Laboratory

The laboratory technicians will greet the participant and check that the stickers on the main questionnaire match the remaining stickers in the folder. The lab technicians will then proceed to collect the blood and urine samples for STI testing and screening for Schistosomiasis according to the Laboratory Technician Standard Operating Procedures **(Annex 11j)**. Serum tubes should be labelled with stickers **(ST7-9)** and urine tubes with stickers **(ST10-11)**. A sticker will be placed in the laboratory registration book and on the Laboratory Submission Form (Annex 45).

The laboratory technicians will test the urine for the presence of RBC using urine dipsticks. The respondent should be given the questionnaire and the ten remaining sticker and directed to the clinician. Female respondents should also be given the container containing the remaining urine sample. This container should be wrapped in tissue paper.

3.3.5 Clinician

The clinician's primary responsibility is to ensure that the survey participants get the correct treatment for any condition that they might have. Thus during the time of the survey, the clinician will only see the survey participants and invited young people who are ineligible for the survey. After the survey has finished, the clinician may see other attendees, but the clinician should avoid treating any other members of the community who did not attend the survey.

The clinician will receive the questionnaires and remaining stickers from the participant. The clinician will first ask about the symptoms that the respondent has at the present time. The treatment protocols are described in section C of the main questionnaire **(Annex 8e)** , and the clinician's instructions in **Annex 11k.** All drugs are to be pre-packed and clearly labelled before the survey starts. The clinician should not therefore waste time counting tablets or searching for drugs.

All males will receive an external genital examination. Only females who report genital ulcers will be examined. Females should be examined while lying on a mattress on the floor/ bed or chair and male participants should be examined in a standing position. The results should be recorded in section C of the main questionnaire. If a female participant requires treatment for a STI then the clinician must carry out a pregnancy test (on the urine remaining in the 60mL container) before deciding on the most appropriate treatment. All participants who are given treatment for STD related complaints must be asked about their sexual partners and given contact referral slips **(Annex 37: Contact Referral Slip)**. It will usually not be possible for the clinician to see the partners. Partners

will have to go to the nearest health centre or dispensary for their treatment. STD diagnosis and all treatment provided should be recorded on section C of the main questionnaire (Annex 8e) and in the clinician registration book.

The clinician will explain that Herpes Simplex Virus 2 is a sexually transmitted infection that cannot be cured but can be treated. He/she will explain that many young people are already infected with this disease and will describe the symptoms. He/she will encourage the participant to attend a health facility if they have any genital ulcers in the future.

Finally, the clinician should consult the QC list which indicates individuals (sticker numbers) that need to complete the Quality Control Questionnaire. The **Quality Control Questionnaire (Annex 9 a-b)** is printed on coloured paper and contains some of the questions from the main questionnaire. The Clinician should attach a sticker to the QC Questionnaire and direct the participant to the other same sex survey interviewer ie SI who did not interview them previously. The participant should complete the QC questionnaire before proceeding to the counsellor (if they would like to visit the counsellor).

3.3.6 Voluntary counselling and testing for HIV (VCT)

Voluntary counselling and testing for HIV will be offered by qualified VCT counsellors (VC) **(Annex 11 l-m)**. Pre-test counselling will be given by the VC and a request for HIV test results form must be signed by the attendee **(Annex 38a-b)**. Blood from those requesting to know their HIV status will be tested immediately at the survey site using two independent blood tests (Bioline and Determine). Post-test counselling will be provided before results are given

If the rapid tests result is indeterminate then the participant will be informed that they will need to wait a further test to be carried out on their blood at NIMR, Mwanza (double ELISA) and that a member of the field team will return as soon as possible to give them their result. The VC will complete a VCT discordants form in order to request HIV test results from NIMR laboratory **(Annex 39: VCT Discordants Form).**

All those who opt for VCT will be informed that a confirmatory test will be carried out on their serum at NIMR, Mwanza and that they may be contacted again if they find that there has been a problem with their test. They will be informed that it is unlikely that there will be a problem with their test.

The VC will complete a VCT results form **(Annex 40)** and place a sticker on the form. The VCT results form and the VCT registration book will contain a survey sticker but will not contain the name of the participants or any other identifying information.

All those who test positive for HIV will be referred **(Annex 41: HIV Treatment Referral Slip)** to the nearest health facility offering ART so that their eligibility for ARVs can be assessed. If there is a home based care organisation working in the survey area then they will be put in contact with the organisation so that they can receive supportive counselling and nutritional care.

Unused stickers must be kept in an envelope and returned to the NIMR data section at the same time as the samples and questionnaires are returned.

3.3.7 Checking of questionnaire

Before the participant returns to registration they will meet with the Data Checker who will, according to their SOP **(Annex 11g),** go through the questionnaire to check that all sections have been completed correctly. If there are any inconsistencies or omissions they will ask the staff member responsible to clarify with the respondent what the answer should be.

3.3.8 Survey Venue Flowchart

Figure 3.1 shows the steps that someone turning up at the survey venue will go through and the estimated time each step will take. Taking into account some waiting time between survey steps, the survey will take between 1.5- 2.5 hours for those participating in all steps of the survey.

MkV1FS_Protocol_Final.doc

Figure 3.1: Survey flowchart

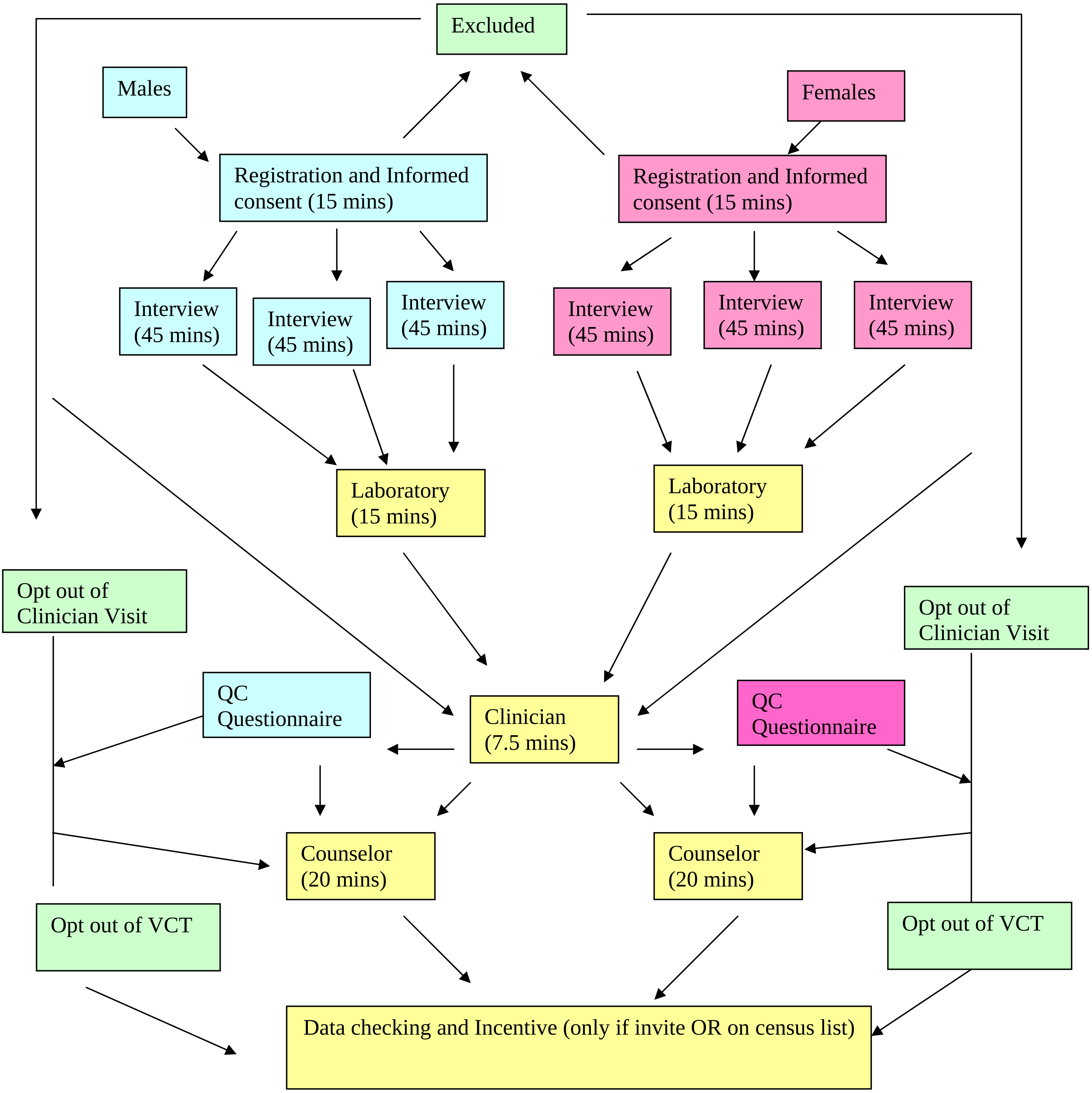

3.3.9 Participant Incentives

When the participants have completed the survey they will be given their incentive by the Data Checker or the Registration Interviewer. **Table 3.3** shows the transport costs and incentives that will be offered to the different categories of attendees.

Table 3.3: Incentives for Survey Attendees

	**Invitation *OR* on census list**	**Eligible for survey**	**STI treatment**	**VCT**	**Incentive and Transport allowance**
1	Yes	Yes	Yes	Yes	4000 Tsh +<br>Large bar of soap (approx. value 1000 Tsh)
2	Yes	No	Yes	Yes	2000 Tsh +<br>Half bar of soap (approx. value 500 Tsh)
3	No	Yes	Yes	Yes	4000 Tsh +<br>Large bar of soap
4	No	No	Only if time after other categories of attendees have availed of services		None

3.3.10 Tracing

There will be 5 tracers on each survey team and they will be responsible for ensuring that the young people identified during the census attend the survey venue **(Annex 11i).** On the first day of the survey in a particular village the tracing team will travel in the project landcruiser, by bicycle or by foot to the areas where those invited to the survey on that day are living. They will encourage the invited young people living in that area to attend the survey venue.

On the second day of the survey they will try to mobilise those invited on the second day and will also try to follow-up on those who did not attend on the first day. On the third day they will try to mobilise those invited to attend the survey on the third day and follow-up on those who were due to attend the previous days. When following-up on young people who did not attend they will record information on the tracing form **(Annex 42: Tracing Form)**.

3.3.11 Team Debrief

At the end of each day the survey team members should attend the team debrief and share their experiences with the rest of the team.

If requested, team members should assist the STL with other tasks including standing in for other members of staff who are ill or on leave.

At the end of the survey in each village the STL will complete the survey village summary form **(Annex 43: Survey village summary form)** and submit this form during the next sample/data collection.

In Mwanza (within 2 days of return from the field), all survey team members must complete the following tasks:

a. Attend the ST debriefing meeting
b. Return all equipment to the project storeroom
c. Complete all report **(Annex 44: Survey community report form)** and retirements required at NIMR office.

3.4 Census and Survey reporting and Field staff supervision

During the field visit the Census and Survey Team Leaders will send daily updates (numbers of households visited, YP interviewed, eligible YP interviewed etc) by text message to the mobile phone of the Field Supervisor or Project co-ordinator. They will also submit reports on each village visited.

The day after his/her team returns to Mwanza, the team leader (Census and Survey) should meet with the FS and/or FM to debrief them on ward mobilisation. The CTL and STL should also submit community reports (Annex 32 and Annex 44) to the MkV1 FS team within one week of returning from the ward. The report should contain information on villages/sub-villages visited, days spent at each survey venue, number of HH visited, number of young people interviewed, problems encountered etc.

The NIMR Mwanza-based Project Co-ordinator (PC), Fieldwork Manager (FM) and Field supervisor (FS) will all be involved in the initial supervision of the field teams ie first few communities. They will travel with the field teams and supervise the setting-up of the survey centre, all steps of the survey including specimen and data collection and each step of the survey process. They will also supervise the census fieldworkers, in particular ensuring that the team have no problems using the PDAs and creating the list of survey participants.

When the census and survey teams have completed the first few communities, field supervision will be carried out primarily by the Field supervisor (will spend approx. 75% of his/her time in the field) and will make supervision visits on alternate weeks to both census and survey teams. At each supervision visit, the supervisor must take out sufficient supplies for the 2 teams in the field; this includes the materials for the survey as well as food and personal requirements. All supervisors from Mwanza (FS, FM, PC, technical support) will complete a supervision report form within 2 days of returning to the office (Annex 45).

The FS will aim to visit one of the field teams each week and will spend at least one day with the census team and one day with the survey team during his/her visit. The FS and CTL will observe the CI at work and complete the CI supervision form **(Annex 46).** They will also conduct blind and non-blind repeat visits to households previously visited by CI and complete the census supervision form **(Annex 47)**. He/she will sit in on some survey interviews and will prepare a summary of the key findings. The FS will also conduct some quality control interviews with the young people and will feedback to the team

leader if any areas for improvement are identified. The FS will ensure that the team leaders are completing the summary forms as required. During the FS visit to the field, he/she will discuss any problems or concerns relating to the census or survey with local leaders. The PC and FM will occasionally accompany the Field Supervisor on visits to the field sites.

3.5 Other field activities

3.5.1 Specimen collection

Blood and urine samples will be processed and packaged for transport by the field laboratory technicians). An additional project Landcruiser will be used for collecting specimens and will travel to meet the teams and collect specimens approximately every week (collect - approx. 183 blood and 183 urine samples). Transportation to NIMR, Mwanza will be in portable freezer and/or heavy-duty coolbox with fresh ice-packs, so that samples are kept at maximum 4°C. Sample submission forms **(Annex 48: Lab Submission Form)** should be filled in for all samples sent to Mwanza and should be signed by the staff member who delivers the samples and the Lab Assistant who receives the samples in NIMR.

3.5.2 Data collection

Completed questionnaires and other forms (including village census and survey summary forms) will be collected at the same time as the laboratory specimen collection. These forms will be transported to the Data section of MITU where they will be double-entered. All paper forms and questionnaires sent to NIMR must be accompanied by a Data Submission Form **(Annex 49: Data Submission Form)** which should be signed by the staff member who delivers the forms and the data manager who receives the forms at NIMR.

3.5.3 Post-survey mopping up

The extent and intensity of mopping-up activities (eg revisiting survey communities, visit to major migration points etc) will depend on the number of young people that participate in the survey.

3.5.4 Post-survey STI treatment

The NIMR, Mwanza lab and data section will, within 5 weeks of receipt of laboratory samples, produce a list of participants who test positive for a treatable STI (active syphilis or NG or CT) and who were not treated using syndromic management. A dedicated team member will return to the communities approximately 2 months after the survey to offer treatment to these individuals **(Annex 11q)**. They will explain that they are making a follow-up to the survey and will only discuss the STI diagnosis and treatment with the participant. They will complete the Treatment after lab results form **(Annex 50: Treatment after lab results form)** and provide referral slips for contacts if necessary **(Annex 51: Contact Referral Slip- follow up STI Rx)**.

Those who test positive for HSV2 will not be visited, as this is not a curable although it is a treatable infection. During the survey the clinician will counsel all participants on the importance of attending a health facility if they have genital ulcers.

In the unlikely event that there is a discrepancy between the VCT test results and the HIV ELISA result then a participant will be revisited. They will be provided with the ELISA test result and will be offered further counselling. Only those who chose to avail of VCT during the survey will be informed if their ELISA test result is positive.

3.6 Logistics

3.6.1 Accommodation

Census and survey teams will sleep in guesthouses or camp (depending on the location) and will be responsible for the safe keeping of the field equipment. Whenever possible, field teams will stay in the same place.

3.6.2 Personnel Identification

Survey team members will be provided with ID cards and must carry them at all times. All field staff will be provided with project t-shirts so that they can be easily identified. Census interviewers and tracers will also be provided with project baseball caps.

3.6.3 Project Vehicles

The Fieldwork Manager and Team Leaders will be responsible for co-ordinating the use of project vehicles and ensure that they are kept in a roadworthy condition. Drivers will be responsible for refuelling, the basic maintenance and secure parking of the vehicles. Drivers must obey the rules of the road, wear a seatbelt and ensure that all passengers wear a seatbelt (if fitted). Drivers must complete the vehicle logbook for each journey completed **(Annex 11o).**

Mobilisation Officers are responsible for ensuring that the project motorcycles are kept in a roadworthy condition. They should keep a vehicle log for all journeys and obey the rules of the road. They are required to wear a safety helmet when riding a motorcycle **(Annex 11a: Mobilisation Officer SOPs)**.

3.6.4 Money for fieldwork

All MkV1 FS staff will receive an overnight allowance when they spend a night outside Mwanza carrying out survey activities. The team leader will be responsible for managing the overnight allowances in the field and will keep a list of people who return to Mwanza. The team leader will also manage the money to cover other field expenses. The project payment form will be used where payment is made without invoice or receipt eg payment of community helpers **(Annex 52: Payment Voucher)**.

3.6.5 Communications

Senior MkV1 FS staff will communicate by mobile phone and will be reimbursed for calls/text messages according to the MkV1 FS mobile phone policy. Text messaging will be used wherever possible.

3.6.6 Timeline

The proposed start date is in December 2006 and the project will last 30 months. Four months have been allowed for recruitment and training of the field teams, and for pre-testing and pilot testing of the survey procedures. It will take one team approximately 4

weeks to carry out the survey and census in each community. Allowance has been made for restocking and a short rest period back in Mwanza between each community and for a repeat visit to each community at the end of the study period to attempt to locate eligible young people who were absent during the first visit. **11.5 months** have therefore been allowed for the census and main survey.

**Date**	**Activity**
Dec'06-Mar'07	Finalising survey protocol, recruitment and training field teams, pilot study
May'07–Apr'08	Survey data & specimen collection
May'08–Sept'08	Completing data processing & lab analysis
Oct'08–May'09	Statistical analysis and writing-up, dissemination of results

4. Data management

A database will be created by the data section NIMR, Mwanza. Data checks and consistency checks will be built into each file entered by the data entry personnel. No names or other identifiers will be kept on the computers. All data will be double-entered and checked for inconsistencies.

The pilot study data will be examined to assess the success of the questionnaires and forms. The main survey data will be entered within 2 weeks of receipt of data. The first priority will be to enter the main questionnaire. Other questionnaires and forms will be entered in due course with regular feedback being given to both data entry personnel and the field staff on the quality of the data received.

5. Laboratory analysis

All the main research laboratory tests will be done in the NIMR STI lab **(Annex 53: Laboratory Analytical Plan).**

HIV will be tested for using two independent antibody/antigen ELISA tests (Murex, Vironostika), with additional confirmation using p24 antigen ELISA and Western Blot (Inno-Lia), as appropriate **(Annex 54: HIV testing algorithm)**.

HSV2 antibodies will be tested for using the kalon HSV2-specific ELISA test. The TPPA test will be used to detect syphilis, with positive also being tested for active syphilis using the RPR test. NG and CT antigen will be tested by PCR (Amplicor), initially in pools of 5 specimens with retesting of each specimen within positive pools.

The laboratory work will be processed within 3 weeks of the specimens being taken. The laboratory results will be taken and entered into the computer by the data entry personnel. Results will be merged and matched based on sticker numbers. Laboratory data from other sites will be collated and merged with the main questionnaire results.

7. Communication and Dissemination

The MkV1 Further Survey in collaboration with MkV2 have developed a MkV Communication Strategy which outlines the steps that will be taken to disseminate the results of the trial. Stakeholders at all levels from International to local village level will be

involved and information will be tailored to their specific needs. The MkV Advisory Committee will be revived and they will provide technical advice to the trial co-ordinators.

8. References

1. UNAIDS, WHO. AIDS epidemic update:2004: UNAIDS, 2004: 94.
2. Gallant M, Maticka-Tyndale E. School-based HIV prevention programmes for African youth. *Soc Sci Med* 2004;**58**(7)**:**1337-51
3. Clark LR, Brasseux C, Richmond D, Getson P, D'Angelo LJ. Are adolescents accurate in self-report of frequencies of sexually transmitted diseases and pregnancies? *J Adolesc Health* 1997;**21**(2)**:**91-6.
4. Plummer ML, Wight D, Ross DA, et al. Asking semi-literate adolescents about sexual behaviour: the validity of assisted self-completion questionnaire (ASCQ) data in rural Tanzania. *Trop Med Int Health* 2004;**9**(6)**:**737-54.
5. Plummer ML, Ross DA, Wight D, et al. "A bit more truthful": the validity of adolescent sexual behaviour data collected in rural northern Tanzania using five methods. *Sex Transm Infect* 2004;**80 Suppl 2:**ii49-ii56.
6. WHO. Steady....ready.... go! The Talloires consultation to review the evidence for policies and programmes to achieve the global goals on young people and HIV/AIDS. Geneva, 2004: 8.
7. Kirby D. Emerging Answers: Research Findings on Programs to Reduce Teen Pregnancy. Washington, DC: National Campaign to Prevent Teen Pregnancy, 2001: 187.

MkV1FS_Protocol_Final.doc

S/No.	Annex	Form	Languages
1		Protocol (English)	Eng
2	**1**	Protocol (Swahili)	Swah
3	**2**	MkV1 Results of 3-year evaluation	Eng
4	**3**	Allocation to intervention and comparison arms	Eng
5	**4**	Draft Fieldteam training schedule	Eng
6	**5**	Codes for wards, villages and schools	Eng
7	**6**	Draft timetable for community	Eng
8	**7a**	Back-up Census Questionnaire	Eng
9	**7b**	Back-up Census Questionnaire	Swah
10	**7c**	Back-up Census Questionnaire- additional sheet	Eng
11	**7d**	Back-up Census Questionnaire- additional sheet	Swah
12	**8a**	Main Questionnaire- Section A	Eng
13	**8b**	Main Questionnaire- Section A	Swah/Suk
14	**8c**	Main Questionnaire- Section B	Eng
15	**8d**	Main Questionnaire- Section B	Swah/Suk
16	**8e**	Main Questionnaire- Section C	Eng
17	**9a**	QC questionnaire	Eng
18	**9b**	QC questionnaire	Swah/Suk
19	**10a**	Packing list MO	Eng
20	**10b**	Packing list CT	Eng
21	**10c**	Packing list RI/SI/TRACER	Eng
22	**10d**	Packing list STL	Eng
23	**10e**	Packing list CLIN	Eng
24	**10f**	Packing list COUNS	Eng
25	**10g**	Packing list DRIVER	Eng
26	**10h**	Packing list LAB	Eng
27	**10i**	Packing list ST supplies	Eng
28	**10j**	Packing list for follow-up STI Rx	Eng
29	**10k**	Packing list supervisor	Eng
30	**10l**	Packing list Clinician- mopup round	
31	**10m**	Packing list Counsellors- mopup round	
32	**10n**	Packing list Drivers- mopup round	
33	**10o**	Packing list Lab tech- mopup round	
34	**10p**	Packing list MO- mopup round	
35	**10q**	Packing list Team Leader- mopup round	
36	**10r**	Packing list tracer- mopup round	
37	**11a**	SOP for Mobilisation	Eng
38	**11b**	SOP for Census Interviewer	Eng
39	**11c**	SOP for Census TL	Eng
40	**11d**	SOP for Census IT equipment	Eng
41	**11e**	SOP for Registration Interviewers	Eng
42	**11f**	SOP for Survey Interviewers	Eng
43	**11g**	SOP for Data checker	Eng
44	**11h**	SOP for Attendee Co-ordinator	Eng
45	**11i**	SOP for Tracer	Eng
46	**11j**	SOP for Lab. Technician	Eng
47	**11k**	SOP for Clinician	Eng
48	**11l**	SOP for VCT	Eng
49	**11m**	SOP for Bioline_Determine	Eng
50	**11n**	SOP for Survey TL	Eng
51	**11o**	SOP for Drivers	Eng
52	**11p**	SOP for Field Supervisor	Eng
53	**11q**	SOP for follow-up STI treatment	Eng
54	**11r**	SOP STI treatment post survey	Eng
55	**11s**	SOP Follow-up of HIV results	Eng
56	**11t**	SOP Tracing post-survey	Eng

S/No.	Annex	Form	Languages
57	**11u**	SOP Census checker	
58	**11v**	SOP Tracer- Mop up round	
59	**11w**	SOP Mobilisation officer- Mop up round	
60	**11x**	SOP Preparation of lists- Mop up round	
61	**12**	District Mobilisation forum schedule	Eng
62	**13**	Order of communities for District	Eng
63	**14a**	Information sheet for officials/community member	Eng
64	**14b**	Information sheet for officials/community member	Swah
65	**15a**	WDC presentation	Eng
66	**15b**	WDC presentation	Swah
67	**16a**	Letter of introduction from ward to villages	Eng
68	**16b**	Letter of introduction from ward to villages	Swah
69	**17a**	Letter for venue/acommodation agreement	Eng
70	**17b**	Letter for venue/acommodation agreement	Eng
71	**18a**	List of household heads form	Eng
72	**18b**	List of household heads form	Swah
73	**18c**	List of household heads form- add sheet	Eng
74	**18d**	List of household heads form- add sheet	Swah
75	**19a**	Information sheet for Household head	Eng
76	**19b**	Information sheet for Household head	Swah
77	**20**	Survey Information poster	Swah
78	**21**	Mobilisation report form	Eng
79	**22**	Census village information sheet	Eng
80	**23**	CI form for HH	Eng
81	**24a**	Household head consent form	Eng
82	**24b**	Household head consent form	Swah
83	**25a**	Invitation to Attend the Main survey	Eng
84	**25b**	Invitation to Attend the Main survey	Swah
85	**26a**	Information sheet about Main survey for parent/guardian	Eng
86	**26b**	Information sheet about Main survey for parent/guardian	Swah
87	**27a**	Parent/ guardian consent form	Eng
88	**27b**	Parent/ guardian consent form	Swah
89	**28a**	Moved away form	Eng
90	**28b**	Moved away form	Swah
91	**29**	PDA and GPS borrowing form	Eng
92	**30**	CT daily progress report form	Eng
93	**31**	Census village summary form	Eng
94	**32**	Census Community Report form	Eng
95	**33a**	Information sheet for participants	Eng
96	**33b**	Information sheet for participants	Swah
97	**34**	Walkman lending form	Eng
98	**35a**	Informed consent check questions	Eng
99	**35b**	Informed consent check questions	Swah
100	**36a**	Participants consent form	Eng
101	**36b**	Participants consent form	Swah
102	**37**	Contact referral slip	Eng/Swah
103	**38a**	Request for HIV test results form	Eng
104	**38b**	Request for HIV test results form	Swah
105	**39**	VCT Discordants form	Eng
106	**40**	HIV VCT results form	Eng
107	**41**	HIV treatment referral slip	Eng
108	**42**	Tracing form	Eng
109	**43**	Survey village summary form	Eng

S/No.	Annex	Form	Languages
110	**44**	Survey Community Report form	Eng
111	**45**	Supervision Report form	Eng
112	**46**	CI Supervision Form	Eng
113	**47**	Census Supervision Form	Eng
114	**48**	Lab Submission Form	Eng
115	**49**	Data submission form	Eng
116	**50**	Treatment after lab results form	Eng
117	**51**	Contact referral slip- follow-up STI Rx	Swah
118	**52**	Payment voucher	Eng?
119	**53**	Laboratory Analytical Plan	Eng
120	**54**	HIV Testing Algorithm	Eng
121	**55**	PEP report form	Eng
122	**56**	Policy on PEP for needle stick injury and other exposure to potentially infection materials for Lab, Counsellor & Clinician	Eng
123	**57**	PEP patient information sheet	Eng
124	**58**	PEP HIV test form	Eng
125	**59**	Agreement letter for use of project vehicles	Eng
126	**60**	Data analysis plan	Eng
127	**61**	Field Team extra supplies form	Eng
128	**62**	MkV1 FS Staff code of conduct	Eng
129	**63**	Progress Report Form	Eng
130	**64**	Calendar of events	Swah
131	**65**	Template for Survey Register books	Eng
132	**66**	Description of PDA database	Eng
133	**67**	HIV follow-up form	Eng
134	**68**	HSV2 lab testing algorithm	Eng
135	**69**	Syphilis lab testing algorithm	Eng
136	**70**	CT/NG lab testing algorithm	Eng
137	**71**	Office data submission form	Eng
138	**72**	Data section submission form	Eng
139	**73**	Extra census household numbers	Eng
140	**74**	Extra census invitation numbers	Eng
141	**75**	Contact referral slip- STI Rx round	Swah
142	**76**	Client referral slip- STI Rx round	Swah
143	**77**	Consent form- STI Rx round	Swah
144	**78**	General invitation- STI Rx round	Swah
145	**79**	Protocol- Mop up round	Eng
146	**80**	Household head list - Mop up round	Eng
147	**81**	Order of communities for Mop up round	Eng
148	**82**	Reminder invitation form- Mop up round	Swah
149	**83**	Moved away form- Mop up round	Swah
150	**84**	List of documents- Mop up round	Eng
151	**85**	CI form - Mop up round	Eng

MkV1FS Prot A1_Swahili protocol for training .doc

Taarifa ya MEMA kwa Vijana Trial Further Survey

08 May 07

Orodha ya vifupisho

ASRH	Adolescent Sexual and Reproductive Health (afya ya jinsia na uzazi ya vijana)
CDE- Inspector	
CT	Chlamydia trachomatis
DC	District Commissioner
DAC	District AIDS Coordinator (mratibu wa ukimwi wa wilaya)
DAS	District Administrative Secretary
DCDO	District Community Development Officer
DCI	Development Co-operation Ireland (now called Irish Aid)
DED	District Executive Director
DEO	District Education Officer
DFID	UK Department for International Development
DPLO	District Planning Officer
EC	European Commission
GPS	Geographical Positioning System
HIV	Human Immunodeficiency Virus
HSV2	Herpes Simplex Virus 2
IA	Irish Aid
MDG	Millennium Development Goals
MEMA	Mpango wa Elimu na Maadili ya Afya kwa Vijana
MkV1	MEMA kwa Vijana Trial
MkV1 FS	MEMA kwa Vijana Trial Further Survey
MkV2	MEMA kwa Vijana larger-scale implementation and operations research
MITU	Mwanza Intervention Trials Unit
MRC	Medical Research Council
NG	Neisseria gonorrhoeae (kisonono cha Neisseria)
NIMR	National Institute for Medical Research
PDA	Personal Data Assisistant
RAS	Regional Administrative Secretary
RCT	Randomised Controlled Trial
SRH	Sexual and Reproductive Health (afya ya jinsia na uzazi)
STI	Sexually Transmitted Infection (magonjwa ya zinaa)
TA	Technical Advisor
UNAIDS	Joint United Nations Programme on HIV/AIDS
UNGASS	
VCT	Voluntary Counselling and Testing for HIV (upimaji na ushauri wa kujitolea wa virusi vya ukimwi)
WEC	Ward Education Coordinator
WEO	Ward Executive Officer

Orodha ya maelezo

mhudhuria	Kijana anayeshiriki kwenye utafiti (anayefaa na asiyefaa)
mshiriki	Kijana anayefaa anayeshiriki kwenye utafiti
Kijana mwaalikwa	Kijana afaaye mtarajiwa aliyealikwa kushiriki kwenye utafiti

MkV1FS Prot A1_Swahili protocol for training .doc

Muktasari

Lengo kuu la MEMA kwa Vijana 1 Trial Further Survey (MkV1 FS) ni kutathmini matokeo ya mikakati ya MkV 1 kwa afya ya uzazi ya vijana.

MkV1 FS itagawanyika katika awamu 3:

- ***awamu ya uhamasishaji***: MkV1 Further Survey (sababu za utafitii, malengo na utaratibu) itawasilishwa kwa wahusika kitaifa, kimkoa, kiwilaya na kikata. Uhamasishaji utaenda sambamba na kuvitembelea vijiji vilivyolengwa katika utafiti.Wakati wa ziara hizi wanajamii watapata nafasi ya kupokea taarifa ya shughuli za utafiti na wataweza kuuliza maswali.
- ***awamu ya sensa***: Kupitia sensa ya kaya. Vijana wafaao kushiriki kwenye uchunguzi watajulikana na wataalikwa kushiriki kwenye utafiti.

- ***awamu ya utafiti***: Vijana wafaao watahojiwa kuhusiana na afya ya uzazi kwa kutumia njia ya ana kwa ana, wataombwa kutoa sampuli ya damu na haja ndogo kwa ajili ya kupima virusi vya ukimwi na magonjwa mengine ya zinaa(STIs).Washiriki watapewa pia tiba ya magonjwa ya zinaa (STIs) na ushauri wa hiari (VCT)pamoja na upimaji wa Virusi Vya Ukimwi(VVU).

1. Usuli

1.1 Utangulizi

Shirika la Umoja wa mataifa linalojihusisha na Ugonjwa wa Ukimwi (UNAIDS) wanakisia kwamba mwaka 2004 , asilimia 60 ya maambukizi ya Virusi Vya Ukimwi (VVU) yametokea Afrika kusini mwa jangwa la Sahara. Nusu ya maambukizi mapya yametokea kwa vijana wenye umri wa kati ya miaka 15-24, vijana wenye umri huu huwa na magonjwa mengi ya zinaa na mimba zisizotarajiwa. Kwa kuwa chanjo na tiba havijapatikana, mabadiliko ya tabia ni mkakati mkuu wa kuzuia VVU, hasa hasa kwa vijana. Hata kwenye nchi zilizoathirika zaidi maambukizi ya VVU na magonjwa ya zinaa(STIs) ni madogo kwa vijana wenye umri chini ya miaka 15, lakini hupanda haraka sana baada ya hapo.
Mikakati yenye manufaa inayowalenga vijana ikipatikana italeta mabadiliko makubwa upande wa janga la Ukimwi. Hata hivyo vithibitisho vichache vihusuvyo ufanisi wa mikakati inayolenga tabia inatokana na utafiti uliofanyika kwenye nchi zilizoendelea.Tena matokeo yake yanagongana.

Mikakati michache imekaguliwa kwa makini kwenye nchi zinazoendelea, ambapo mahitaji ni makubwa zaidi. Kwa mfano utafiti wa hivi karibuni uliweza kuonyesha programu 11 tu za kuzuia VVU shuleni Afrika kusini mwa jangwa la Sahara iliyokaguliwa kwa kutumia mbinu za majaribio (experimental) au mbinu za kufanana na hizo (quasi-experimental). Karibu programu zote zilionyesha kuboreka kwa uelewa kuhusiana na mambo ya afya ya uzazi, nyingi zaidi zikaonyesha kuboreka kwa mitazamo inayotajwa, lakini kazi nyingi zaidi hazikuonyesha mabadiliko ya tabia ihusuyo maswala ya ngono ama mabadiliko yaliyokuwepo kwa baadhi ya vikundi tu. Hakuna kazi iliyofanyika kukagua matokeo ya mikakati upande wa *biomedical* na hakuna iliyokagua matokeo baada ya ufuatiliaji wa miezi 24.Nyingi zaidi (8/11) zilikaguliwa kwa mara ya mwisho ndani ya miezi 6. Hata hivyo ukaguzi wa matokeo ya kibiolojia ni muhimu mno kwa sababu ya ukweli usio na uhakika uhusuo tabia zilizotajwa na vijana na kwa sababu kuna uwezekano mkubwa wa mikakati kubadilisha tabia zilizotajwa, ziwe zile tu

zinazojulikana kutakiwa kwenye eneo la mkakati. Vilevile ni muhimu sana kujua kama mikakati ina matokeo ya muda tu au kama itabadilisha tabia na kwa njia hii hubadilisha kuenea kwa VVU kwa miaka mingi. Ukaguzi wa hivi karibuni wa uthibitisho wa ufanisi wa mikakati ya kupunguza kuenea kwa VVU na kufanikisha UNGASS ihusuyo VVU na Malengo ya millenia yahusuyo vijana, *"Steady,ready,go"* ilionyesha wazi ukosefu wa kazi makini yenye matokeo ya kibiolojia. Serikali na ajenti za wafadhili, kwa hiyo hulazimika kutumia mikakati ya kubahatisha bila faida ya uthibitisho wa matokeo yao ya kupunguza kuenea kwa VVU.

1.2 Mikakati ya MEMA kwa Vijana

Mikakati ya MEMA kwa vijana ni programu ya afya ya uzazi (SRH) kwa ajili ya vijana vijijini Mwanza, Tanzania.

Mikakati inatekelezwa na AMREF pamoja na Wizara ya Afya na Wizara ya Elimu na Utamaduni kupitia miundo ya kiserikali iliyopo. Ilitengenezwa hasa kuwa endelevu na rahisi kurudia kwenye maeneo yasiyo na fedha nyingi. Kwa kipindi cha 1999-2002 (MkV1) ilikuwa na sehemu kubwa nne :

(i) elimu ya afya ya uzazi shuleni kwa madarasa ya 5, 6 na 7 ya shule za msingi kupitia programu shirikisho zinazoongozwa na mwalimu kwa msaada wa waelimishaji rika kwa njia za maigizo, hadithi na michezo. Muktasari unaunganisha tabia kuu zilizoonekana kuwa muhimu kwenye programu yenye ufanisi shuleni kuhusiana na mabadiliko ya tabia kwenye tafiti zilizotangulia.

(ii) Huduma za afya ya kijinsia na uzazi zinazowafaa vijana, kupitia mafunzo kwa wafanyakazi wa sekta ya afya kuhusu namna ya kutoa huduma za afya ya kijinsia na uzazi wenye kuvutia na ufanisi.

(iii) uenezi na ugawaji wa kondomu kwenye jamii kwa ajili ya vijana (2000-2001 ya MkV1 peke yake).

(iv) vitendo vinavyohusu jamii kuunda mazingira ya kuisaidia mikakati ya afya ya uzazi kwa vijana, ikiwemo wiki moja ya kuanzia ya kuhamasisha jamii, uundaji wa kamati ya kata kusaidia na kusimamia vitendo vya mradi, wiki ya afya ya vijana ya kila mwaka na siku za afya ya vijana za mara mbili kwa mwaka kwenye taasisi za afya za serikali.

1.3 MEMA kwa Vijana jaribio la kubahatisha la jamii

Jaribio la MEMA kwa Vijana (MkV1) ni jaribio la kwanza la kubahatisha kwa kusimamiwa (RCT) kote duniani lenye matokeo yanayopimika ya kibiolojia kuhusiana na kupima ufanisi wa mikakati ya afya ya kijinsia na uzazi kwa vijana (ASRH) ya kuzuia VVU na magonjwa mengine ya zinaa (STIs).Jaribio la MkV1 limefadhiliwa na Kamisheni ya Ulaya (European Commission (EC)), Shirika la maendeleo la Ireland (Development Cooperation of Ireland (DCI)) na Shirika la Utafiti wa Madawa la Uingereza (UK Medical Research Council (MRC)) na msaada wa ziada kutoka mashirika ya UNAIDS na UK DFID. Kuanzia Januari 1999, jamii 20 (kila moja ikilingana na kata) zipatikanazo popote maeneo ya wilaya za Sengerema, Geita, Kwimba na Missungwi za mkoa wa Mwanza zilichaguliwa kwa njia ya kubahatisha kupata (jamii 10 za mikakati zenye jumla ya shule

za msingi 58 na taasisi za afya 18) au kutopata (jamii 10 za kulinganisha zenye jumla ya shule za awali 63 na taasisi za afya 21) mikakati ya MkV.

Kwa kipindi cha mwezi wa kwanza 1999 hadi mwezi wa tatu 2002 matokeo ya mikakati kuhusu HIV, magonjwa mengine ya zinaa STIs, mimba zisizotarajiwa, maoni yanayotajwa na tabia za ngono pamoja na uelewa wa SRH ulitathminiwa kati ya kundi la vijana 9,645. Wakati wa kujiandikisha mwishoni mwa 1998, kundi zima lilikuwa na umri wa miaka 14 (>95% wenye miaka 14-18) na walikuwa wakihudhuria darasa la 4-6 la shule ya msingi **Uchunguzi wa awali** ulifanyika wakati wa kukusanya kundi mwaka wa 1998, na **uchunguzi wa kati** 2000, na **uchunguzi wa kufuatilia baada ya miaka mitatu** mwezi wa 10, 2001 hadi mwezi wa nne 2002.

Matokeo ya tathmini ya mwaka wa tatu yalionyesha kwamba mikakati ya MkV1 iliboresha kwa kiwango kikubwa uelewa, maoni yanayotajwa na baadhi (si wote) ya walitoa taarifa ya mabadiliko ya tabia, hasa hasa kati ya vijana wa kiume. Matokeo ya tathmini ya mafanikio ya kibiolojia hayakueleweka, sababu moja kuwa wakati ule, jaribio lilikosa nguvu ya takwimu ya kugundua matokeo muhimu ya afya ya jamii kuhusu VVU na magonjwa ya zinaa (STIs) mengine yaliyopimwa.Zilikuwepo ***zero conversion*** 5 tu za VVU kati ya wanaume na 40 kati ya wanawake, kati yao uwiano uliorekebishwa ulikuwa 0.75 (95% CI: 0.34, 1.66). Jumla ya maambukizi ya HSV2 wakati wa ufuatiliaji wa mwaka wa tatu ulikuwa 11.9% kwa wanaume na 21.1% kwa wanawake na uwiano uliorekebishwa wa maambukizo wa 0.92 (95% CI: 0.69, 1.22) na 1.05 (95% CI: 0.83, 1.32), Hakuna matokeo mengine yaliyoonyesha wazi matokeo ya kibiolojia, hata hivyo kulikukuwa na maambukizo makubwa zaidi ya kisonono cha Neisseria (NG) kati ya wanawake wa jamii yenye mikakati. (Annex 1: Matokeo ya tathmini ya MkV1 ya mwaka wa tatu).

1.4 Maendeleo tangu mwisho wa jaribio

Kati ya 2002 na 2005 elimu ya jinsia na afya ya uzazi shuleni, msaada na usimamizi wa huduma za afya ya jinsia na uzazi zinavyowafaa vijana, na vitendo vinavyohusisha jamii yote viliendelea kwenye jamii 10 zenye mikakati tu, kwa ufadhili wa Irish Aid (kabla ya hapo DCI).Mwaka 2006 MEMA kwa Vijana awamu ya 2 ilianza kwenye jamii za kulinganisha ikiwa sehemu ya upanuaji wa programu kwenda wilaya zote nne kwa ufadhili wa shirika la misaada la Ireland (Irish Aid (MkV2)).Upanuzi huu ulifadhiliwa na Irish Aid. Mikakati ya MkV2 hufanana sana na MkV1 isipokuwa sehemu ya utoaji wa kondomu haipo.Upanuzi huu ulienda sambamba na uchunguzi wa njia bora za utekelezaji wa mikakati, uchunguzi wa sera na uchunguzi wa uundaji kwa lengo la kubuni sehemu zingine za jamii zitakazoingizwa kwenye mikakati. Mikakati ya MkV2 huwalenga zaidi vijana (hasa wenye umri kati ya 13 na 16) wanaohudhuria miaka mitatu ya mwisho ya elimu ya msingi.

1.5 Sababu za uchunguzi zaidi

Maelezo mojawapo yatarajiwa ya kueleza uhaba wa matokeo ya moja kwa moja ya kibiolojia wakati wa miaka mitatu ya jaribio la MkV1, pamoja na mabadiliko makubwa upande wa uelewa, maoni yanayotajwa, na tabia za kujamiana zinazotajwa, ni kwamba mikakati inahitaji muda zaidi kufanya kazi. Muda wa ufuatiliaji wa miaka mitatu uliopangwa kwa ajili ya awamu ya kwanza ya jaribio ulisababisha vikwazo visivyozuilika vifuatavyo :

1. kikundi kilichomo hatarini zaidi (darasa la 6 wakati wa uteuzi) kilitoa asilimia 40 ya kundi lote la jaribio, lakini walipata mwaka mmoja tu wa sehemu ya mikakati iliyofanyika shuleni. .

2. Kuna tofauti kubwa ya umri kati ya wavulana na wasichana wenye mahusiano ya kujamiana kwenye jamii hii. Wenza wa kiume kwenye mahusiano ya kimapenzi wa wasichana hao wa kundi hawakuhusishwa kwenye sehemu iliyofanyika shuleni na vilevile wenza wa wavulana wanaokuwemo kwenye kundi yawezekana walikuwa wadogo zaidi na kwa hiyo hawakuhusishwa kwenye sehemu ya shuleni.Kulingana na tofauti ya kimamlaka kati ya wanaume na wanawake yaweza kuwa lazima washiriki wawili au hata yule wa kiume wawe wame husishwa na mikakati kabla ya mabadiliko makubwa ya tabia kutokea.

3. Kiasi kidogo ya HIV kwenye kundi la jaribio la waendao shule ilipunguza uwezo wa kugundua matokeo ya mikakati upande wa HIV.

Uchunguzi mwingine wa kufuatilia utafanyika 2007/8 kutathmini matokeo ya muda mrefu ya mikakati ya MkV1. **MkV1 Further Survey (MkV1 FS)** una faida kadhaa ukilinganishwa na awamu ya awali ya jaribio. Kwanza washiriki wa vikundi vitano kati ya vikundi sita waendao shule watakuwa wamepata nafasi kupokea si chini ya miaka miwili ya mikakati shuleni. Pili washiriki wengi wa kiume wa vijana wa kike waliomo kwenye uchunguzi watakuwa wamepewa sehemu ya MkV shuleni kabla ya hapo, hayo yaweza kusababisha matokeo makubwa zaidi upande wa wasichana hao. Uenezi wa HIV kwenye jamii hiyo yenye umri mkubwa zaidi utakuwa mkubwa zaidi na kwa hiyo uchunguzi utaongezewa uwezo wa kugundua tofauti kwenye uenezi wa HIV.Wanakundi wa kundi la awali watakuwemo pia na kwa hiyo matokeo ya muda mrefu kuhusu ujuzi, maoni na matokeo ya mara kwa mara mengine yataweza kupimika kwenye kikundi kidogo hicho.

<u>Wote watakaofaa kushiriki katika MkV1 FS watakuwa wamemaliza shule ya msingi kabla ya 2006 (Takwimu 1.1) Vijana kwenye jamii ya kulinganisha waliohusishwa kwenye sehemu ya mikakati ya MkV2 shuleni hawatashirikishwa</u>

Uchunguzi huu utafanyika miaka minane baada ya uanzilishi wa mikakati ya MkV1 na utaweza kuchunguza matokeo ya mikakati baada ya muda mrefu, baada ya kuhusisha makundi kadhaa ya vijana yaliyofuatana. Hata hivyo uchunguzi utabaki na manufaa ya muundo wa *community randomized controlled trial* ya awali.Mapendekezo hayo yatumia dirisha la pekee la nafasi kuchunguza moja kwa moja mafanikio ya muda mrefu wa mikakati ya kitabia ihusuyo HIV na STIs na kuendeleza ujuzi mkubwa na uzoefu uliopatikana kwenye awamu ya awali ya jaribio.

1.6 Lengo

Kupeleleza ikiwa kuna matokeo ya kudumu ya mikakati ya MEMA kwa Vijana (MkV1) kuhusu afya ya kijinsia ya vijana.

1.7 nadharia tete itakayochunguzwa

Baada ya muda mrefu zaidi MEMA kwa Vijana (MkV1)husababisha uboreshaji wa afya ya jinsia na uzazi

MkV1FS Prot A1_Swahili protocol for training .doc

1.8 matokeo ya jaribio

Matokeo ya awali yatakuwa :

- Uenezi wa HIV na uenezi wa kingamwilini dhidi ya HSV2

Matokeo ya pili yatakuwa:

- Uenezi wa kaswende, klamydia trachomatis (CT) na kisonono ya Neisseria (NG)
- Alama tatu za ujuzi wa afya ya kijinsia na alama moja ya maoni ya afya ya kijinsia (kila alama inatokana na maswali matatu, kama yalivyotumika kwenye chunguzi za MkV zilizotangulia)
- Tabia za kijinsia zinazotajwa, zikiwemo : tendo la kwanza la ngono,idadi ya washiriki wa ngono maishani, idadi ya washiriki wa ngono kwa muda wa miezi 12 iliyopita, matumizi ya kondomu na mshiriki wa mwisho ambaye si wa kudumu, matumizi ya mbinu ya kuzuia uzazi kwenye tendo la mwisho la ngono.
- Idadi ya mimba maishani iliyotajwa

1.9 Mpangilio wa uchunguzi

Uchunguzi wakilishi wa vijana wanavyoishi kwenye jamii ya majaribio 20 ya MkV1 (10ya mikakati, 10ya kulinganisha).

Vikundi maalum vitakavyoalikwa kushiriki kwenye uchunguzi huonyeshwa kwenye **Takwimu 1.1**. Kwa kifupi sifa za kushiriki zitakuwa::

- Kuonekana kuwa mwanakaya kwenye kaya ndani ya jamii 20 ya jaribio(Kiambatisho cha 2 huonyesha idadi ya vijana kwenye mkondo wenye mikakati na idadi ya vijana kwenye mkondo wa kulinganisha).Mwana kaya *de jure* ni yeyote anayelala au aliyelala hadi hivi karibuni mara kwa mara kwenye kaya husika.

- Amehudhuria darasa la 5,6 au 7 kwenye shule ya msingi ndani ya jamii ya jaribio kwa muda usiopungua mwaka mmoja kati ya 1999 na 2002 (kipindi ambapo mikakati ilitekelezwa kwa nguvu zaidi na chini ya usimamizi mkali)

- Tayari kukubali taratibu zote za uchunguzi baada ya kueleweshwa

Takwimu 1.1. Mchoro wa kundi unaoonyesha wafaao kwa ajili ya uchunguzi (2007/2008)-mgawanyiko wa umri

- Nambari ndani ya kila eneo huonyesha **idadi ya miaka ya kuhusishwa** na mikakati ya MkV shuleni, ikihesabiwa kufuatana na mwisho wa mwaka wa kalenda kwa waliokuwemo kwenye jamii 10 yenye mikakati. Idadi ya miaka ya kuhusishwa itakuwa 0 kwa vijana wale watakaoalikwa kushiriki kwenye jamii 10 za kulinganisha (hawaonyeshwi).
- **Vikundi vya miaka ya shule** vilivyo na wale wafaao kuwemo kwenye makundi ya jaribio ya awali vilisisitiswa kwa kutumia rangi ya manjano. Vikundi vya mwaka wa shule vingine walivyobahatisha kupata si chini ya mwaka mmoja ya mikakati shuleni wakati wa awamo ya utekelezaji wa nguvu (yaani 1999-2002) vikaonyeshwa kwa kutumia bluu mpauko. Uchunguzi wa kufuata utatekelezwa upande wa pili wa mwaka 2007 na utakuwa na vikundi vya miaka ya shule vyote viwili.(imechorwa sambamba/cross-hatched kwenye mstari kwa mwisho wa mwaka 2007)
- Wastani na pana wa umri vyatokana na matokeo ya uchunguzi wa awali (1998) kwa darasa la 4, 5 na 6 wakati ule bila kuingiza umri, ambapo < 1% wa kikundi cha mwaka wa shule walikuwa na umri huu.
- Vikundi vya mwaka wa shule walivyohusishwa na mikakati ya MkV2 upande wa wenye mikakati NA upande wa kulinganisha vilionyeshwa kwa pink. Mikakati ya MkV2 ilianza kwenye 25% za shule kwenye wilaya 4za jaribio katikati ya 2005. 50% nyingine za shule zilianzaOct,2006na25%zamwishozitaanzamikakatiMay07

Kitendo		Shule ya msingi				Miaka tangu kumaliza shule								
	Mwaka	dar 4	dar 5	dar 6	dar 7	+1	+2	+3	+4	+5	+6	+7	+8	+9
Uchunguzi wa kuandikisha (Aug-Dec '98)	Dec 1998	0	0	0										
Kuanza mikakati Jan '99	Dec 1999		1	1	1									
	Dec 2000		1	2	2	1								
Uchunguzi wa tathmini-mwaka wa tatu	Dec 2001		1	2	3	2	1							
	Dec 2002		1	2	3	3	2	1						
	Dec 2003		1	2	3	3	3	2	1					
	Dec 2004		1	2	3	3	3	3	2	1				
	Dec 2005		1	2	3	3	3	3	3	2	1			
	Dec 2006		1	2	3	3	3	3	3	3	2	1		
Uchunguzi wa tathmini mwaka wa nane	Dec 2007		1	2	3	3	3	3	3	3	3	2	1	
Wastani wa umri mwishoni mwa 2007(upana)kwa kila kundi la miaka ya shule – was kike		13 (10,16)	14 (11,17)	15 (12,18)	16 (13,19)	17 (14,20)	18 (15,21)	19 (16,22)	20 (17,23)	21 (18,24)	22 (19, 25)	23 (20, 26)	24 (21, 27)	25 (22, 28)
Wastani wa umri mwishoni mwa 2007 (upana)kwa kila kundi la miaka ya shule- wa kiume		14 (11,18)	15 (12,19)	16 (13,20)	17 (14,21)	18 (15,22)	19 (16,23)	20 (17,24)	21 (18,25)	22 (19,26)	23 (20, 27)	24 (21, 28)	25 (22, 29)	26 (23, 30)

MkV1FS Prot A1_Swahili protocol for training .doc

1.10 ukubwa wa sampuli na uwezo wake

Idadi ya wanajamii wanaofaa kwa uchunguzi huu ina mpaka (yaani lazima wawe wamehudhuruia shule za msingi za jaribio wakati wa muda wa jaribio lililotangulia) na kwa hiyo ukubwa wa sampuli wa 14, 520 ulipatikana (Sanduku 1.1) kutokana na idadi ya vijana wafaao :

(i) walio na umri wa miaka17-25
(ii) wanaoweza kupatikana wakati wa sensa na kuhudhuria uchunguzi
(iii) walio tayari kushiriki

Sanduku 1.1.

Tunakisia kutakuwa na wastani wa wanaume 720 na wanawake 720 kutoka kila jamii waliokuwa wakimaliza si chini ya mwaka mmoja kati ya miaka mitatu ya mwisho ya shule ya msingi kwenye jamii hiyo kati ya 1999 na 2002. Kufuatana na matokeo ya uchunguzi wa uandikishaji wa awali tunategemea kwamba 90% ya vijana hao watakuwa wametimiza miaka 17-25 ifikapo mwisho wa 2006. Tunategemea pia kwamba 70% ya vijana hao bado wanaishi kwenye jamii hizo na kwa hiyo wataandikishwa wakati wa sensa, na 80% watakubali kushiriki kwenye uchunguzi wa kufuatilia. Kwa hiyo jumla ya sampuli itakuwa **14,520** (wahojiwa 726 kwa kila jamii x jamii 20).
Uwezo wa uchunguzi kugundua upungufu wa kweli wa uwepo wa matokeo ya kibiolojia ulihesabiwa kwa wanaume na wanawake tukitegemea wanaume 365 na wanawake 365kwa kila jamii, jamii 10 kila mkondo na k=0.2, ambapo k ni kizigeu wiano cha badiliko kati ya jamii kuhusiana na matokeo haya (***Jedwali 1.1***). Uwepo wa alama za mwisho (endpoints) zote zingine hutegemewa kuwa zaidi ya 25% , kwa hiyo uchunguzi utakuwa na uwezo zaidi kwa alama hizo.

Sifa za kushiriki au kutoshiriki zikabadilishwa na sasa hakuna mpaka wa umri yaani vijana wafaao wenye umri wote waweza kushiriki kwenye uchunguzi. Hapo awali tulikisia kwamba 10% ya vijana waliohudhuria shule za jaribio wakati unaotakiwa wasingefaa kushiriki kutokana na umri na kwa kuondoa kizuizi cha umri tungetegemea ongezeko la ukubwa wa idadi ya jamii inayofaa. Hata hivyo kutokana na ukosefu wa uhakika kuhusu idadi ya vijana watakaopatikana wakati wa sensa na/au idadi ya vijana watakaokubali kushiriki hatukubadilisha ukubwa wa sampuli.

Pamoja na kazi ya awali ukosefu wa uhakika uko zaidi upande wa kuenea kwa HIV. Kwa kutumia kisio zuri zaidi uchunguzi utakuwa na uwezo wa kutosha (79%) kugundua upungufu wa 35% kwenye uenezi wa HIV upande wa wanawake, lakini utakuwa na uwezo mdogo tu (64%) kugundua upungufu wa40% upande wa wanaume. Vilevile uchunguzi utakuw ana uwezo mzuri kugundua tofauti ya 35% kwa kaswende na 25% kwa HSV2, kwa kila jinsia. Matokeo ya jinsia zote mbili yakifanana yataweza kujumlishwa kwa lengo la kuongeza uwezo. Uchunguzi wa vikundi kwa matokeo ya kawaida zaidi kama HSV2, ujuzi, maoni na tabia zitakuwa na matokeo kufuatana na umri, idadi ya miaka ya kupewa mikakati shuleni na kati ya wale waliokuwemo kwenye kundi la awali la MkV.

Jedwali 1.1 Uwezo wa kugundua kiwango cha matokeo mbalimbali

Matokeo	Jinsia	Uenezi (%) kwenye jamii ya kulinganisha [1]	Kiwango cha matokeo[2]			
			40%	35%	30%	25%
**HIV**	**Wanaume**	1.0	0.40	0.31	0.24	0.17
		2.0	0.64	0.51	0.39	0.28
		2.3	0.69	0.56	0.43	0.31
	**Wanawake**	3.0	0.77	0.64	0.50	0.36
		4.0	0.85	0.73	0.58	0.43
		4.5	0.88	0.76	0.62	0.46
		5.0	0.90	0.79	0.65	0.48
		6.0	0.93	0.83	0.69	0.52
		7.0	0.95	0.86	0.73	0.56
**Kaswende**	**Wanaume**	6.5	0.94	0.85	0.71	0.54
	**Wanawake**	10.0	0.97	0.92	0.80	0.63
**HSV2**	**Wanaume**	25.0	1.00	0.98	0.91	0.77
	**Wanawake**	35.0	1.00	0.98	0.93	0.80

maelezo

[1] imekisiwa kwa kutumia uenezi na uwepo uliokisiwa kutokana na chunguzi zingine mkoani Mwanza

[2] Tofauti ya uenezi kati ya jamii zenye mikakati na jamii za kulinganisha

□ Kisia bora ya uenezi wa kila tokeo

MkV1FS Prot A1_Swahili protocol for training .doc

2. Maandalizi upande wa taasisi ya kitaifa ya utafiti wa tiba

2.1 Agano la maelewano

Agano la maelewano (MoU) kati ya London School of Hygiene & Tropical Medicine (LSHTM) na Mwanza Intervention Trials Unit (MITU) /National Institute for Medical Research (NIMR), Mwanza itawekewa sahihi. Afisi na sehemu ya ghala maeneo ya NIMR, Mwanza zitatolewa kwa ajili ya uchunguzi.

2.2 Ruhusa rasmi na vibali

Kibali cha utafiti na maadili kwa ajili ya MkV kimeshapatikana kutoka Umoja wa Jamhuri ya Tanzania kupitia Medical Research Coordination Committee (MRCC) na kutoka LSHTM Ethics Committee.

Kabla vitendo vya MkV1 FS havijaanza mkoani Mwanza, wafanyakazi wakuu wa programu watamtembelea katibu tawala wa mkoa (RAS), afisa elimu wa mkoa (REO) na afisa tiba wa mkoa(RMO) kutambulisha mradi. Watamwomba RAS kuandika barua kutambulisha MkV1 FS kwa wakurugenzi watendaji wa wilaya (DED) na kwa afisa watendaji wa wilaya (DEO) kwa kila moja ya wilaya nne za MkV1.

Kwenye kila moja ya wilaya nne za MkV1 FS, wafanyakazi wakuu wa MkV1 FS wataandaa mkutano kuwajulisha wawakilishi waalikwa kuhusu uchunguzi na kuwapa nafasi wilaya kutoa maoni yao kuhusiana na mipango.Ruhusa ya kutekeleza vitendo vya uchunguzi kwenye wilaya yao na barua za utambulisho kwa maafisa watendaji wa kata zitaombwa. Mikutano ya wilaya itafuatana na mikutano kwenye kata na kjiji ambapo wanajamii watapata nafasi ya kupata habari ya uchunguzi na kuuliza maswali.

2.3 Ununuzi/ kukodi usafiri

Vifaa vya IT na vifaa vya tiba visivyopatikana nchini Tanzania vitanunuliwa Uingereza na kuletwa Mwanza kwa meli. Vifaa vingine (vikiwemo magari) vitanunuliwa Tanzania. Makubaliano ya kukodi itawekewa sahihi kwa ajili ya landcruiser na daladala.

2.4 Upaiekanaji wa wafanyakazi

Nafasi zote kwenye timu ya MkV1 FS ambazo haziwezi kujazwa na wafanyakazi waliopo wa MITU/NIMR zitatangazwa kwenye magazeti ya Afrika mashariki (nafasi za juu) na/au kwenye mbao za matangazo. Wafanyakazi wakuu wa MkV1 FS na MITU/NIMR wataandaa orodha fupi ya waombaji kufuatana na sifa binafsi na matakwa ya kazi. Mitihani na mahojiano yatafanyika NIMR, Mwanza mbele ya paneli ya wafanyakazi wa MkV1 FS na MITU/NIMR. Wafanyakazi wengi zaidi wataajiriwa kwa muda wa miezi 12 kwa msingi wa mikataba ya miradi ya kawaida ya NIMR/AMREF/LSHTM(Collaborative project contracts).Mikataba ina muda wa jaribio kwa miezi mitatu na makubaliano ya kawaida na masharti kmf posho ya likizo ya mwaka, ugonjwa, uzazi na kufiwa nk.

Wafanyakazi wakuu wa MkV1 FS (Meneja wa kazi ya field na msimamizi wa field) na wafanyakazi wa ufundi (Afisa tiba, washauri, mafundi maabara, dereva)watapatikana na kufunzwa mwezi wa pili na mwezi wa tatu 2007.Idadi kubwa ya wafanyakazi wa field inahitajika (wahojaji,wafanyakazi wa sensa, wafuatiliaji) Uamuzi kuhusu uwezo wa kazi utafanyika kwanza kwa msingi wa CV na barua ya maombi. Baadhi ya waombaji wataalikwa kwa wiki ya kwanza ya mafunzo mwezi wa nne, 2007. Wale tu watakaofanya

vizuri kwenye mtihani wa maandishi mwisho wa wiki na wanaofanya vizuri kwenye vitendo vya vikundi wataalikwa kwenye wiki ya pili ya mafunzo. Uundaji wa kudumu wa timu mbili za field utaamuliwa mwisho wa wiki ya pili ya mafunzo. Wiki ya tatu na ya mwisho itakuwa na uchunguzi wa kujaribishia (pilot study) Wakati wote wa uchunguzi utakuwa na usimamizi na mafunzo kazini.

2.5 Taratibu za kawaida za utekelezaji

Maelezo ya undani ya taratibu zote zinazopaswa kufuatwa kila hatua ya uchunguzi (field, data na maabara) yataandikwa. Taratibu hizo zitabadilishwa wakati wa uchunguzi wa kujaribishia na ikibidi hata wakati wa uchunguzi wenyewe.

2.6 Mafunzo ya wafanyakazi wa field

Mafunzo na maandalizi kwa ajili ya wafanyakazi wa field yataendelea kutoka tarehe 23 mwezi wa 4 hadi tarehe 25 mwezi wa 5 2007. Wiki ya kwanza ya mafunzo yatalenga taarifa ya uchunguzi na kidadisi kikuu na wiki ya pili yatahusu matumizi ya Personal Data Assistants (PDA) na Geographical Positioning Systems (GPS) kwa ajili ya sensa (Kiambatisho cha 3: Ratiba ya kwanza ya mafunzo ya wafanyakazi wa field). Pamoja na hayo wafanyakazi wa tiba, maabara na ushauri watakaa muda mfupi kwenye taasisi ya afya/maabara Mwanza na watatumia muda kuchunguza wafanyakazi wa miradi mingine ya utafiti (HSV2 na Mwamko).

2.7 uchunguzi wa kujaribishia

Kila moja ya timu ya field itakuwa na wiki tatu za mafunzo yakiwemo uchunguzi wa kujaribishia Nassa Ginery, Magu District. Lengo la uchunguzi wa kujaribishia ni kujaribu vifaa, vidadisi, na taratibu za kuhamasisha, kuandikisha, kuhoji na kukusanya data. Uchunguzi wa kujaribishia utakuwa na sensa kupata vijana wafaao, uandaaji wa orodha ya washiriki wa uchunguzi, uandaaji wa kituo cha sensa, ukusanyaji na usafrishaji wa sampuli za maabara. Data za kuanzia zitaingizwa na kuchunguzwa na matokeo yatatumika kwa mafunzo zaidi ya timu na kukamilisha vifaa vya uchunguzi.

2.8 vifaa vya ziada vya kuandaliwa na NIMR kabla ya uchunguzi

2.8.1 orodha za mhojaji uandikishaji

- **Orodha ya walioalikwa kwenye uchunguzi wakati wa sensa (Orodha A)**

Fomu za orodha hizo zitaandaliwa NIMR na zitakamilishwa wakati wa sensa na uandikishaji wa uchunguzi. Orodha A1- A3 zitatengenezwa field kwa kutumia taarifa zinazotokana na PDA ya wahojaji wa sensa (tazama sehemu 3.2.3).

- **Orodha za shule za la MkV pamoja na kiashilio chake (Kiambatisho xx: orodha ya shule za jaribio ya MkV) (orodha B)**

- **Orodha ya vijana waliohusika na mikakati ya MkV1 kati ya 1999 na 2002 (na waliomo kwenye kundi la kulinganisha) (Orodha C na D)**

Orodha ya wanakundi wa jaribio la MkV1 itaandaliwa kwa kutumia data za kundi la jaribio la MkV. Ombi rasmi la kupata nyakala za matokeo ya mtihani wa darasa la 7 litapelekwa afisi elimu ya mkoa wa Mwanza.

C1: orodha ya wanakundi wa kiume wa jaribio la MkV1
Hii itakuwa orodha ya walioandikishwa wakati wa uchunguzi wa kujaribishia na itakuwa na walioshiriki kwenye kundi la kutasmini matokeo ya jaribio la MkV1, yaani waliozaliwa kabla ya mwezi wa kwanza tarehe moja 1985 (kitambulisho cha bluu cha MkV1) na waliozaliwa baada ya tarehe moja mwezi wa kwanza 1985 (kitambulisho cheupe cha MkV1). Data zinazobadilikabadilika kwenye orodha hii zitakuwa pamoja na: majina ya kuandikishwa na majina mengineo, jinsia, nambari ya kitambulisho cha MkV1,jina la shule na miaka ya kuhudhuria darasa la 5,6 na 7.

C2: orodha ya wanakundi wa kike wa jaribio la MkV1 (kama C1 hapa juu)

D1: orodha ya matokeo ya mtihani wa darasa la 7 (2002)
Hii ni orodha rasmi ya wale waliomaliza darasa la 7 shule ya msingi 2002 inayopatikana kwenye afisi elimu ya wilaya au ya mkoa. Orodha itapangwa kufuatana na jinsia kwa kila shule ya msingi.

D2: orodha ya matokeo ya mtihani wa darasa la 7 (2003) (kama D1 hapa juu)

D3: orodha ya matokeo ya mtihani wa darasa la 7 (2004) (kama D1 hapa juu)

2.8.2 Ramani za maeneo ya uchunguzi na jamii ya uchunguzi

Ombi rasmi litapelekwa kwenye mamlaka ya mipango ya mkoa kupata ramani ya kisasa ya mkoa wa Mwanza.Ramani hii itatumika kupanga na kusimamia uchunguzi.

2.8.3 Ratiba za uchunguzi

MkV1 FS itatumia mpangilio wa jamii uleule sawa na uchunguzi wa MkV 2001/02 (**Jedwali 2.1**).Ratiba ya vitendo vilivyopangwa kwenye kila jamii itatolewa kama taarifa kwa wahusika wa wilaya na kata. Itatumika kukodi mahali pa kuendesha uchunguzi, malazi na usafiri wa timu ya field. **(Kiambatisho 9)**.Afisa uhamasishaji atabadilisha ratiba ya kwanza kufuatana na taarifa za kisasa anazozipata kuhusu ukubwa wa vijiji wakati wa matembezi yake ya uhamasishaji kwenye kata.

Jedwali 2.1 Mpangilio wa jamii kwa ajili ya MkV1 FS

Mpangilio wa jamii	Jina la jamii & namba		Hali ya mikakati ya jamii & tabaka	
	Timu 1	Timu 2	Timu 1	Timu 2
1.	Katunguru (5)	Koromije (22)	I1	C1
2.	Kasamwa (11)	Mwagi (23)	I1	C1
3.	Katoro (8)	Usagara (21)	I2	C2
4.	Busisi (6)	Nyehunge (3)	C2	I2
5.	Kagu (9)	Nyakaduha (4)	I3	C3
6.	Nkome (1)	Katwe (2)	C3	I3
7.	Lubanga (10)	Fukalo (17)	C3	I3
8.	Ihanamilo (12)	Hungumalwa (16)	I2	C2
9.	Bukoli (13)	Misasi (18)	C1	I1
10.	Nyang'whale (14)	Malya (24)	C2	I2

2.8.4 vidadisi na fomu

Vidadisi na fomu zitatokana na zile zilizotumika wakati wa MkV1,kwenye chunguzi zingine zilizofanyika Mwanza na chunguzi zingine zipimazo afya ya jinsia na uzazi. Vidadisi na fomu zitatengenezwa kwenye Microsoft Word.

Mwanzoni vidadisi na fomu zote zitaundwa kwa kiingereza halafu zitatafsiriwa kwa Kiswahili na kisukuma, baadaye zitatafsiriwa upya kwa kiingereza. Vidadisi vyote vitajaribishiwa mara kwa mara na vitabadilishwa. Nakala ya mwisho itatumika kwenye uchunguzi wa kujaribishia na ikibidi utabadilishwa kabla ya uchunguzi.

Vidadisi vifuatavyo vitahitajika na timu ya sensa na timu ya uchunguzi (kwa kingereza na Kiswahili/kisukuma):

- Kidadisi cha sensa (kilichomo kwenye PDA na nakala ya dharura kwenye karatasi) **(Kiambatisho cha 4)**
- Kidadisi kikuu **(Kiambatisho cha 5)**
- Kidadisi kikuu cha kuthibitisha ubora (aina10) **(Kiambatisho cha 6)**

10% ya washiriki waliochaguliwa bila utaratibu watajibu vidadisi vya kuthibitisha ubora na orodha ya nambari ya vibandiko vilivyochaguliwa kabla itaandaliwa kwa ajili ya timu ya uchunguzi **(Kiambatisho cha 7: orodha ya QC).** Fomu zingine za field **(Kiambatishocha cha x- x)** zitahitajika kwa kingereza na Kiswahili.

2.9.5 vifaa vingine vya field

kmf vibandiko vya gari, t-shirts za wafanyakazi, vibandiko vyenye nambari na vitu vilivyotajwa kwenye orodha ya kufungasha. **(Kiambatisho cha 8: orodha ya kufungasha ya afisa uhamasaji; Kiambatisho cha 9: orodha ya kufungasha ya timu ya sensa; Kiambatisho cha 10: Orodha ya kufungasha timu ya uchunguzi ; Kiambatisho cha 11:orodha ya kufungasha Sampuli/ ukusanyaji wa data; Kiambatisho cha 12:orodha ya kufungasha usimamizi wa field)**

3. Taratibu za uchunguzi

3.1 Uhamasaji

Kutakuwa na afisa wawili wa uhamasishaji watakaotekeleza vitendo vya uhamasishaji kufuatana na taratibu za kawaida za utekelezaji wa uhamasishaji kufuatana na taratibu za kawaida za utekelezaji **(Kiambatisho cha 9: SOPs ya afisa uhamasishaji).**

3.1.1 Upande wa mkoa

Wanatimu wakuu watamtembelea katibu tawala wa mkoa (RAS) kueleza sababu,malengo na taratibu za uchunguzi wa ufuatiliaji wa MkV1. Wataomba ruhusa kutekeleza vitendo vya uchunguzi mkoani Mwanza (ikiwemo kazi ya kujaribishia)na ruhusa ikipatikana wataomba barua ya utambulisho kwenda DED wa kila wilaya wa uchunguzi (Geita, Sengerema, Missungwi, Kwimba) na wilaya zinazohusika kwenye uchunguzi wa kujaribishia (Magu, jiji la Mwanza).Barua hizo za utambulisho zitatoa taarifa kwa wilaya kwamba mkoa umekubali na unasaidia uchunguzi wa ufuatiliaji wa MkV1.
Ombi litapelekwa kwa afisa elimu mkoani kupokea na kutoa nakala matokeo ya mtihani wa darasa la 7 ya miaka 2002-2004.

3.1.2 Upande wa wilaya

Uhamasishaji wa kuanzia utafanyika na wanatimu wa uchunguzi wa field. Afisa uhamasishaji (MO) akisindikizwa na mratibu wa mradi (PC),meneja ya kazi ya field (FM)au msimamizi wa kazi ya field (FS) watakaosafiri kwa gari katika kila wilaya na watawajibika kuhusiana na utambulisho na ruhusa na maandalizi mengine yanayoutangulia uchunguzi upande wa wilaya.
MO akisindikizwa na si chini ya mfanyakazi mkuu mmoja wa MkV1 FS, atatembelea wilaya zote kupeleka barua za utambulisho zitokazo RAS na ataomba ruhusa kwa DED kutekeleza vitendo vya uchunguzi kwenye wilaya yao. Uwezekano wa kuwa na mkutano wa kutoa taarifa kwa ajili ya wahusika wa wilaya utajadiliwa pia. Wahusika walengwa ni DC, DAS, DED, DEO, DMO, DPLO, DCDO/DAC, Mkaguzi wa CDE, Mwenyekiti wa halmashauri ya wilaya,na MkV TA (AMREF).Eneo na tarehe ya mkutano utakubaliwa wakati wa ugeni huu na DPLO ataombwa kutoa mialiko kwa washiriki waliokubalika.

Mkutano wa taarifa ya MkV1 FS utaendelea kwa muda wa nusu siku kwenye eneo lifaalo makao makuu wilayani na utahusisha wastani wa watu 15 toka wilayani na wanatimu wa uchunguzi 2-3. Baada ya maelezo mafupi ya MKV1FS, washiriki watapewa ratiba ya uchunguzi ulivyopangwa **(Kiambatisho cha 9)**,karatasi ya taarifa ya uchunguzi wa wahusika rasmi **(Kiambatisho cha 10)** na watapewa nafasi ya kuuliza maswali. Wakati wa mikutano hii wahusika wilayani watapewa posho ya Tshs. 10,000/= kila mmoja (20,000/= kwa DC, DAS & DED) na chai itatolewa.

Baada ya mkutano DED ataombwa kuandika barua za utambulisho kwa viongozi wa kila jamii ya MkV1 FS wilayani mwao. Barua hizo zitaonyesha kibali na msaada kwa uchunguzi wilayani. MO atafuatilia na kuhakikisha kwamba barua hizo zitafuatwa kwa DED.

Afisa uhamasishaji (MO) atamtembelea Afisa tiba wa wilaya (DMO) kwenye kila wilaya kueleza kwamba MkV1 FS itatoa tiba ya STI kwa washiriki na itatoa vipeperushi vyenye

anwani (contact slips) kwa washiriki wa ngono.Kwa hiyo hospitali za wilaya na taasisi za afya wategemee ongezeko dogo la watu wanaokuja kutibiwa STIs. MO ajadili pia taratibu za kuwapeleka kwingine washiriki watakaokutwa na HIV .

Baada ya kukamilisha uhamasishaji wilayani (au kwenye sehemu ya wilaya) MO mmoja au wote wawili wamtembelee DED na kumpa taarifa ya vitendo vya uhamasishaji wilayani kule. Awakumbushe DED kwamba sensa na uchunguzi hukaribia kuanza wilayani mle (rejea ratiba ya uchunguzi).

3.1.3 Upande wa kata

MO atatembelea kila kata ya MkV1 FS wiki 2 au 3 kabla ya timu ya sensa kuwasili kwenye kata hiyo. Mo atajitambulisha kwa WEO na atapeleka barua ya utambulisho kutoka wilayani, karatasi ya taarifa kwa wahusika rasmi **(Kiambatisho cha 10)**na ratiba ya uchunguzi uliopangwa **(Kiambatisho cha 9).** Atajadiliuwezekano wa WEO kuendesha mkutano wa taarifa MkV1 FS unaofanana na ule upande wa wilaya. Mkutano huu hupaswa kuwahusisha WEO, WEC,Mshauri, VEOs, VCs, HC mhusika (WDC), na wawakilishi wa dini na serikali. Watakaohudhuria mkutano watapata posho ya 5000 Tsh kila mmoja. Posho ya usafiri wa 1000 Tsh utatolewa kwa wanaotoka nje ya makao makuu ya kata (iwe zaidi ya saa moja kwa miguu kutoka sehemu ya mkutano).

Baada ya mkutano wa taarifa MO wa kata hiyo atatekeleza vitendo vya kawaida vya uhamasishaji upande wa kata na kijiji. Wataomba orodha ya vijiji na vitongoji kutoka kwa WEO wakitumia orodha hiyo kurekebisha ratiba ya uchunguzi uliopangwa upande wa kata. Kila MO atashughulikia kata mbili akiwajibika kuhusiana na utambulisho, ruhusa na maandalizi mengine upande wa kata na kijiji. MO watasafiri kwa pikipiki wakitumia wastani wa wiki moja katika kila kata.

MO akutane na vikundi vya matunzo majumbani (homebased care groups) vyovyote kwenye kata na wajadili utaratibu wa kuwaletea washiriki kwa ajili ya tiba ya HIV na misaada mengine kwa watakaokutwa wana HIV. Atatembelea pia taasisi za afya zilizopo kuwajulisha kuhusu uchunguzi na kujadili uwezekano wa ongezeko la watakaohitaji tiba ya STI na/au utaratibu wa uzazi wa mpango.

MO ataomba orodha kamili ya vitongoji vilivyomo kwenye kila jamii ya uchunguzi kutoka kwa WEO .

Kabla ya kuondoka MO amtembelee WEO na kutoa taarifa ya zoezi pamoja na kumkumbusha kwamba sensa na uchunguzi vimekaribia kuanza kwenye kata hiyo(angalia ratiba ya uchunguzi iliyopangwa).

3.1.4 upande wa kijiji

Anapofika kijijini MO atajitambulisha kwa afisa tawala kijijini (VEO), mwenye kiti wa kijiji (VC) na kwa viongozi wa vitongoji.MO atatambulisha uchunguzi unaotarajiwa (malengo na mipango kwa miezi ijayo) na ataacha nakala za karatasi ya taarifa **(Kiambatisho cha 10)** na ratiba ya kudumu ya uchunguzi wa kijiji (kwa lugha ya kiswahili) MO atawaomba viongozi hao wamsaidie kupata sehemu inayofaa kwa ajili ya uchunguzi, kmf nyumba ya kulala, nyumba nyingine, nyumba zisizotumika. MO atajadili sehemu hizo na FS au FM na kwa pamoja wataichagua sehemu inayofaa zaidi. MO atakodi sehemu kwa ajili ya uchunguzi na malazi kwa ajili ya timu ya field (kwa ajili ya sensa na uchunguzi) MO atatumia ratiba ya uchunguzi kuona siku ambapo sehemu na malazi yatahitajika . MO

huwajibika kuhakikisha kwamba sehemu ya kufanyia uchunguzi imeandaliwa kwa ajili ya timu ya uchunguzi kmf kupanga viti, kuandaa maji nk
MO pia atatembelea walimu wakuu wa shule zote, pamoja na viongozi wa kidini, waganga wa kienyeji na watu muhimu wegine kmf mashirika yasiyo ya kiserikali yanayofanya kazi kwenye eneo, ikiwa itakuwa muhimu kuwaalika.Labda kikao cha kijiji kitahitajika ili MO awaeleze watu muhimu kuhusu mpango wa MkV1 FS. Mkutano ukifanyika wanaohudhurio watapewa soda na biskuti. (Kisio:watu 10-20 watu/mkutano)

MO atamwomba kila kongozi wa kitongoji kuorodhesha kaya za kitongoji na kuonyesha kwenye orodha kaya zenye vijana wenye umri kati ya miaka 15-30 (tunakisia kwamba vijana wanaofaa kwa uchunguzi watakuwa na miaka 18-26) MO aeleze kwa undani sababu za orodha hizo kuhitajika na atoe fomu zitakazohitajika **(Kiambatisho cha 11: orodha za kaya kwenye kitongoji)**. MO amwonyeshe kiongozi wa kitongoji jinsi ya kujaza fomu. Fomu ijazwe mara mbili, kiongozi wa kitongoji abaki na wakala moja kumsaidia kuhamasisha. Kila kiongozi wa kitongoji ataombwa awahamasishe wanakitongoji ili wawe tayari kwa timu ya sensa kufika kwenye tarehe zilizopangwa kwenye ratiba ya uchunguzi. MO atakaporudi kwenye kitongoji kukusanya orodha za kaya zilizokamilika (~ wiki moja baadaye),atakagua maendeleo ya uhamasishaji. Kiongozi wa kitongoji akimaliza kazi aliyopewa atalipwa 2,000 Tsh.Ikiwa kitongoji kina zaidi ya kaya 25 kiongozi wa kitongoji atafute mtu mmoja kuwa “msaidizi wa jamii wa sensa” kusaidia wakati wa zoezi la sensa.

3.1.5 Taarifa ya uhamasishaji na usimamizi

Siku moja baada ya Mo kurudi Mwanza akutane na FS na/au FM kuwajulisha kuhusu uhamasishaji kwenye kata. MO hupaswa pia kutoa taarifa ya maandishi kwa timu ya MkV1 FS ndani ya wiki moja baada ya kurudi kutoka kata. Taarifa iwe na habari za vijiji/vitongoji vilivyotembelewa tena iwe na majina na anwani (nambari za simu ya mkononi) za watu muhimu na vikundi kmf viongozi wa vitongoji, shule na wafanyakazi wa taasisi za afya, vikundi vya kutoa matunzo majumbani nk. Taarifa pia iwe na habari kuhusu sehemu zilizochaguliwa na malazi ya timu za field. **(Kiambatisho cha 12: Fomu ya taarifa ya MO**).MO atatoa nakala 10 za kila orodha ya kaya iliyoandaliwa na kitongoji, nakala moja ibaki kwenye afisi yaMkV1 FS na nakala zingine ziende kwa kiongozi wa timu ya sensa. Ratiba ya kudumu ya uchunguzi wa kata ilyiotembelewa iende kwa FS na/au FM.

Wakati wa kutembelea kata kwa mara kadhaa za kwanza MO atasindikizwa na mfanyakazi mkuu wa mradi na watasafiri kwa gari (kata za karibu kmf Katunguru na Koromije zitachaguliwa kwa makusudi ili iwezekane kufanya hivyo). Wakati wa uchunguzi FS atawasiliana na MO kwa njia ya simu kila wiki. MO akipata shida zozote awasiliane na FS na/au FM kila inapohitajika.

3.2 Sensa

Baada ya uhamasishaji, sensa itafanyika katika kila jamii ya uchunguzi ili kuwagundua vijana wanaofaa kushiriki kwenye uchunguzi. Jamii ina eneo la km 5-10 km,idadi ya watu kwa wastani ni 18,000 na kwa wastani ina vijiji 6. Kutakuwa na timu mbili za sensa na kila timu ya sensa itafanya kazi kwenye jamii 10. Kila timu itakuwa na kiongozi wa timu ya sensa(kiongozi makamu wa timu ya uchunguzi) na wahoji wanane, watasafiri kwa landcruiser ya mradi. Kiongozi wa timu ya sensa (CTL) na wahoji wa sensa (CI) watafanya kazi kufuatana na utaratibu wa utekelezaji wa kawaida **(Kiambatisho cha 13: SOPs za kiongozi wa timu ya sensa ; Kiambatisho cha 14: SOPs ya mhojaji wa sensa)**

3.2.1 maandalizi kabla ya sensa

Kwa msaada na usimamizi kutoka kwa wafanyakazi wakuu wa MkV1 FS na kwa ushirikiano na wanatimu wengine wa sensa, kiongozi wa timu ya sensa (CTL) ahakikishe kwamba mambo yote yako tayari kwenda field.**(Kiambatisho cha 13: orodha ya kufungasha ya sensa)**.

Timu ya sensa itafikia kila kata (jamii) siku ya jumapili. Jumatatu asubuhi timu ya sensa itajitambulisha kwa WEO, WEC na wahusika rasmi wengine wa kata kuwajulisha kama sensa inakaribia kuanza kwenye kata. Watawapa wahusika rasmi ratiba ya uchunguzi uliorekebishwa unaoonyesha siku ambapo timu za sensa na uchunguzi watakuwepo kwenye kila kijiji na kitongoji.

Halafu kiongozi wa timu ya sensa(CTL) atasafiri kwenye kijiji kilicho karibu (kwa kawaida karibu na makao makuu ya kata) na atajitambulisha kwa afisa tawala wa kijiji, mwenye kiti wa kijiji na viongozi wa vitongoji.(Afisa *uhamasishaji atawapigia simu viongozi wa kijiji kabla, kuwaandaa kwa ugeni wa timu ya sensa).*Atawakumbusha taratibu za sensa, tena kuwajulisha kwamba sensa itaanza kijijini kwao kesho yake (jumanne) na kuwapa ratiba ya uchunguzi uliorekebishwa. Viongozi wa vitongoji wataombwa kuwahamasisha wakazi wa kitongoji chao ili wengi iwezekanavyo wawepo kwenye kitongoji wakati timu ya sensa itakapofika. Watatiwa moyo watumie orodha ya kaya kuwasaidia na uhamasishaji.

CTL atawaomba viongozi wa vitongoji kusaidia kupata wasaidizi kutoka jamii kuwasaidia wafanyakazi wa sensa wakati wa sensa. (kiongozi wa kitongoji awe mmoja wa wasaidizi).Kila panapowezekana CTL akutane na wasaidizi wa pale walipo na awaeleze kwa undani lengo na taratibu za sensa, wahakikishe wasaidizi wa sensa wajue wajibu wao wakati wa zoezi na kiwango cha malipo. Wasaidizi wa jamii hao(CH) watapata 3000 Tsh/ siku.Kabla ya kutoka kwenye kitongoji CTL ahakikishe kwamba msaidizi wa sensa atakuwa amepata motisha/pesa zake.

Wakati wahoji wa sensa (CI) wanatekeleza sensa kwenye kijiji / kitongoji kimoja CTL ataandaa ugeni wa kabla ya sensa kwa ajili ya kitongoji/kijiji kingine kufuatana na mipango ya ratiba ya sensa (siku moja kabla). Atafanya maandalizi yote yalivyoelezwa hapo juu.

MkV1FS Prot A1_Swahili protocol for training .doc

3.2.2 Sensa

CI atasafiri kwenye eneo la utafiti kutumia landcruiser (landcruiser moja kwa timu) halafu atakwenda kutoka kaya moja hadi nyingine kwa miguu au kwa baisikeli. (baisikeli ya mradi au iliyokodiwa wakati wa zoezi) Kila mmoja wa CL wanane atafanya kazi masaa 48 kwa wiki (masaa 8/siku kwa siku sita) na lazima ahoji kaya 25 kila wiki (wastani ya dakika 20 – 25 kwa kaya, kaya 21 kwa siku kwa CL)Inapaswa kuchukua wiki 3 kukamilisha sensa kwa kila jamii (Jedwali 3.1).Ili kufanikisha vizuri zaidi CTL aweza kuamua kuunganisha kila kitongoji kwenye kijiji kwa kikundi kidogo cha CL.

Jedwali 3.1 muda uliokisiwa wa kumalizia sensa

	**Idadi ya kaya (HH) iliyokisiwa**	**Siku zinazotumiwa na timu ya wahoji wanane wa sensa (CI)**
		168 HH/siku*
**Jamii**	**~ 3000**<br>(365 m wafaao, 365 k wafaao)	**18 siku**
**Kijiji**	~ 500<br>(60 m wafaao, 60 k wafaao)	3 siku

**21 HH/siku/CI (1 HH mahojiano ya kaya 1 kwa dakika 20-25,masaa 8 ya kazi)*

Kwa kutumia orodha ya wakuu wa kaya iliyoandaliwa na kitongoji kwa ajili ya MO, CL ataongozana naye (au na mwaakilishi wake) kwa kila kaya. Akikuta kaya zingine kwenye kitongoji ambazo hazikuandikishwa kwenye orodha ya awali, CL ataziongeza kwenye orodha.

Kwenye kila kaya (HH) CI na msaidizi wa jamii (CH) watajitambulisha kwa kifupi kwa mwenye kaya au mwanakaya na kueleza kwa kifupi lengo la sensa. Mhojiwa aweza kuwa mwenye kaya au mwanakaya mwingine. Kabla ya kumhoji mhojiwa atatoa kibali chake baada ya kueleweshwa. **(Kiambatisho cha 14:Karatasi ya kibali baada ya kueleweshwa kwa ajili ya kaya).**

Kila CI atatumia kompyuta ya mkono (PDA) kuingiza taarifa za wanakaya wenye umri kati ya 15-30 moja kwa moja.Papohapo itachunguzwa kama wafaa kuingizwa kwenye uchunguzi.
Vijana wafaao watapewa ahadi ya kuonana baada ya siku 2-4 kuhudhuria uchunguzi utakaofanyika kwenye nyumba ya malazi iliyoko karibu au kwenye jengo lingine lililokodiwa (sehemu 4-8 kwa kila jamii ya uchunguzi) Ikiwa kijana anayefaa hayupo wakati wa sensa wanakaya wengine wataombwa kumpa mwaliko. Mwaliko wa uchunguzi **(Kiambatisho cha 15: Mwaliko wa uchunguzi)** utakuwa na taarifa zifuatazo:

1. Sehemu na muda wa uchunguzi
2. Maelezo ya taratibu za uchunguzi yakisisitiza kwamba siri zitatunzwa
3. Taarifa za posho ya usafiri na motisha zitakazotolewa
4. Ombi la kuja kwenye uchunguzi na mwaliko, vitambulisho vya uchunguzi wa afya na vyeti vyovyote vilivyo rasmi vinavyoonyesha tarehe ya kuzaliwa na umri [kitambulisho ya MkV1:nambari ya kitambulisho, jina, jinsia, umri, tarehe ya kujiandikisha, kata, kijiji)

Ikiwa wanakaya wasema kijana afaaye hataweza kuhudhuria uchunguzi CI atatoa taarifa kuhusu mahali alipo kwenye fomu ya ufuatiliaji wa sensa. **(Kiambatisho cha 16:Fomu ya kuhama)**.

Kila CI atakuwa na vifaa vya kugundua eneo la kijiografia (GPS) vitakavyomwezesha kuandikisha sehemu maalum ya kaya na kuingiza sehemu hiyo kwenye PDA. Taarifa hizo zitasaidia kupata kaya baadaye kwa kutumia wafuatiliaji au kwa ajili ya tiba ya STI na/au kuchora ramani ya mahali walipo washiriki wa uchunguzi kwa ajili ya utoaji taarifa za uchunguzi kwa undani.

Ikiwa kuna matatizo yoyote ya kumzuia CI asitumie PDA kuingiza data ya sensa CI atumie fomu ya dharura ya karatasi ya sensa.**(Kiambatisho cha 17: Fomu ya dharura ya karatasi ya sensa.)**.

3.2.3 Uundaji wa orodha kwa ajili ya timu ya uchunguzi

Kila jioni CTL atawajibika kuhamisha data kutoka kwa PDAs kwenda kwenye laptop. Kila jioni CTL atatengeneza nakala za data zilizokusanywa kwenye CD tupu. Atawajibika kutunza vifaa vya(yaani laptop, PDAs/GPS, chargers) na ahakikishe viko tayari kwa ajili ya kesho yake(vimechaji).

Ikiwa fomu ya karatasi za kaya ya sensa ilitumika jioni CTL akisaidiwa na CI ataingiza data zilizo muhimu zaidi (majina, nambari ya kitambulisho cha sensa, tarehe/muda wa ahadi ya kufika kwenye uchunguzi) kwenye Access Database.

Timu ya sensa wakimaliza kaya zote kwenye kijiji kimoja (~ kila baada ya siku 3) CTL atatengeneza na kuchapisha orodha ya vijana wafaao (Orodha A1- A3). Orodha zitapelekwa kwa timu ya uchunguzi (siku moja kabla ya uchunguzi).

A1: orodha ya wanaume waliopatikana wakati wa sensa ya MkV1FS

Orodha A1 itapangwa kufuatana na tarehe ya ahadi ya kuonana na muda ikifuatwa na jina la ukoo. Itakuwa na data zinazojibadilisha zifuatazo: jina la kijana (la kwanza, la ukoo, mengine),tarehe ya ahadi ya kuonana na muda, nambari ya kitambulisho ya sensa, kijiji, kitongoji

A2: **orodha ya wanawake aliopatikana wakati wa sensa ya MkV1FS** (kama A1 hapo juu)

A3: orodha ya wenye kaya
Orodha hii itapangwa kufuatana na nambari ya kitambulisho ya sensa na itakuwa na majina ya kijana, majina ya wenye kaya, kitongoji,kijiji na sehemu ya GPS. Orodha hii itakuwa pia na nafasi ya mhojaji wa uandikishaji kuingiza tarehe ya uchunguzi, ishara ya kitambulisho ya mhojaji na itakuwa na nafasi ya kibandiko.

3.3 Uchunguzi

Kutakuwa na timu mbili za uchunguzi, kila moja ikiwa na wanatimu 22:

Kiongozi wa timu (1)
Afisa tiba (1)
Washauri (2)
Wafuatiliaji(5)
Wahojaji uandikishaji (2)
Wahojaji (3wa kiume, 3 wa kike)
Wafanyakazi wa maabara (2)
Msaidizi tawala wa maabara (1)
Msaidizi tawala wa data (1)
Dereva(1)

3.3.1 maandalizi kabla ya uchunguzi

Kwa msaada na chini ya usimamizi wa wafanyakazi wakuu wa MkV1 FS,na kwa ushirikiano na wanatimu wa uchunguzi wengine, kiongozi wa timu ya uchunguzi athibitishe kwamba mambo yote yanayohitajika yameshughulikiwa na kwamba timu ya uchunguzi iko tayari kwenda field **(Kiambatanisho cha 18:orodha ya kufungasha ya timu)**.

Kiongozi wa timu ya uchunguzi (STL) anapofika kwenye jamii akutane na CTL na kujadili maendeleo ya sensa na jambo lolote lililo na umuhimu kwa uchunguzi. CTL atampa STL nakala zilizochapishwa za orodha za waalikwa kwenye uchunguzi **(Orodha A1-A3)**.

CTL na STL watawatembelea WEO, WEC na wahusika rasmi wengine wa kata kuwajulisha hali halisi ya maendeleo ya sensa na kuwakumbusha wahusika rasmi ratiba na taratibu za uchunguzi. Halafu STI atawatembelea viongozi kwenye kijiji cha kwanza wakiwemo viongozi wa kitongoji kutambulisha timu ya uchunguzi na kukamilisha mipango ya uchunguzi.

Sensa inapoanza kwenye kijiji kiongozi wa timu ya sensa (CTL) atakwenda kwenye nyumba ya malazi/nyumba zitakazotumiwa kwa ajili ya sensa kwenye kijiji hiki, atahakikisha kwamba ziko tayari kwa ajili ya timu ya uchunguzi watakaofika siku 2-4baadaye. Pia STL atatembelea eneo la uchunguzi siku 1 au 2 kabla ya timu ya uchunguzi kufika kijijini ahakikishe kwamba kila kitu ni tayari kwa ajili ya timu ya uchunguzi. CTL na STL watawasiliana mara kwa mara kupitia SMS/simu kuhusu sehemu ya uchunguzi na malazi ya timu ya field.

Kila timu ya uchunguzi pamoja na vifaa vyao watasafiri kutoka Mwanza kwenda kwenye jamii ya uchunguzi kutumia basi la kukodi (si chini ya nafasi 22 za kukaa) Basi hili litakodiwa tu kwenye hizo siku za timu kusafiri kutoka jamii moja hadi nyingine. Kila timu ya uchunguzi itakuwa pia na gari la landcruiser la kuwasafrisha kutoka kijiji kimoja hadi kingine ndani ya jamii.

Kila timu itafika kijijini siku 2-4 baada ya timu yao ya sensa na wataandaa kituo cha uchunguzi kwenye nyumba au nyumba ya kulala iliyopangiwa kabla. Kituo cha uchunguzi kitakuwa na eneo la kujiandikisha na eneo la kusubiri (labda nje kwenye kivuli). Vyumba 6 kwa ajili ya mahojiano ya ana kwa ana, chumba kimoja kwa ajili ya fundi maabara, vyumba viwili kwa ajili ya washauri wa VCT na chumba kimoja kwa ajili ya afisa tiba (vyumba 10 + na eneo la kujiandikisha/kusubiri).

Tunakisia watakuwa vijana ~ 14,500 wafaao watakapofika kwenye maeneo ya mahojiano. Tunategemea timu moja wataweza kuhoji washiriki 48/siku. Itachukua siku 15 kuwahoji wanaume na wanawake 720 wafaao kwenye jamii moja. Timu itabaki siku za ziada kwenye kila kituo ili wawahoji wale walioshindwa kuhudhuria uchunguzi kwa muda uliopangwa. (Jedwali 3.2).

Jedwali 3.2 muda unaokisiwa kumalizia uchunguzi

	**Namba iliyokisiwa ya vijana wanaofaa**	**Siku zinazotumiwa na timu ya uchunguzi**
		48 watu/siku*
**Jamii**	**~ 720**<br>(365 m wafaao, 365 k wafaao)	**15 siku**
**Kijiji**	~ 120	2.5 siku

* *wanaume 4 na wanawake 4 / saar,masaa manane ya kazi*

Vijana waalikwa watakuja kituoni kwa siku na muda uliotajwa kwenye mwaliko wa uchunguzi. Timu ya sensa watawaalika vijana 30 saa 2 asubuhi kila siku na vijana 30 saa sita kila siku. Waalikwa wanapofika watasalimiwa na mwanatimu wa uchunguzi na wataonyeshwa maeneo ya kusubiri. Watapewa karatasi ya taarifa **(Kiambatisho cha 18: Karatasi ya taarifa kwa washiriki)** na walkman yenye kanda inayokuwa na taarifa ya mradi. Mmoja wa wanatimu atawaonyesha wahudhurio jinsi ya kuwasha, kusitisha na kuzima kanda. Mhudhurio atakapokuwa amesoma taarifa na/au amesikiliza habari, atahojiwa na mhojaji wa uandikishaji.

3.3.2 Uandikishaji

Wahojaji wa uandikishaji wawili watakuwepo Mmoja k.wa ajili ya wahudhurio wa kike, mwingine kwa ajili ya wahudhurio wa kiume. Watafuata hatua zilizoelezwa kwenye taratibu za utekelezaji za kawaida **(Kiambatisho cha 19: SOPs ya wahojaji wa uandikishaji)**. Wahojaji wa uandikishaji (RI) watawasalimia wahudhurio na wataandika tarehe na muda wa mahojiano, jina la mhudhurio, kijiji, kitongoji na jina la mwenye kaya kwenye kitabu cha uandikishaji. Baada ya hapo RI atamhoji mhudhurio akamilishe sehemu A ya kidadisi kikuu.Mhojaji wa uandikishaji atathibitisha utambulisho wa mhudhurio kwa kumwomba mwaaliko wake wa uchunguzi na/au kwa kutafuta jina lake kwenye orodha ya sensa (A1-A2). Ataingiza nambari ya kitamulisho chao cha sensa kwenye kitabu cha uandikishaji. RI atatumia orodha B au C kuona kama mhudhurio anafaa. Ikiwa kijana ni mwanakundi wa MkV1 RI ataweka alama kwenye safu ya MkV1 kwenye kitabu cha uandikishaji.

Ikiwa mhudhurio anafaa RI atamwuliza baadhi ya maswali kuona kama anaweza kutoa kibali baada ya kueleweshwa **(Kiambatisho cha 19:Maswali ya kukubali baada ya kueleweshwa)**. Ikiwa mhudhurio hawezi kutoa kibali baada ya kueleweshwa arudi kwenye eneo la kusubiri hadi mmoja wa wanatimu (STL, mshauri au RI) anaweza kujadili naye kwa undani zaidi maana ya uchunguzi. Pale ambapo mwanatimu ana uhakika kwamba mhudhurio ana uwezo wa kutoa kibali baada ya kueleweshwa, mhudhurio atarudi kwa RI na ataulizwa kama atakubali kushiriki kwenye uchunguzi. Kibali kikitolewa lazima aweke sahihi au alama ya kidole kwenye karatasi ya kutoa kibali baada ya kueleweshwa **(Kiambatisho cha 20: Fomu ya mshiriki ya kutoa kibali baada ya kueleweshwa).** RI atabandika kibandiko kimoja **(ST1)** kinachokuwa na nambari moja ya pekee ya utambulisho wa uchunguzi kwenye karatasi ya kibali. Kibandiko cha pili **(ST2)** chenye nambari hiyo hiyo kitawekwa karibu na jina lao kwenye kitabu cha uandikishaji. Ikiwa jina lipo kwenye orodha ya sensa (Orodha A3) kibandiko kingine **(ST3)** na jina hili kitawekwa karibu kwenye orodha**.** Kibandiko cha nne **(ST4)** kitabandikwa kwenye kidadisi kikuu..

Tahadhari:

(1) Wahudhurio wataulizwa pia kama wako tayari kupokea matibabu kwa ajili ya STI zozote zitakazogundulika siku moja baada ya uchunguzi. Ikiwa wanakubali kushiriki lakini wanakataa kutibiwa bado wanafaa kushiriki kwenye uchunguzi.
(2) Kama mhudhurio yeyote hawezi kutoa kibali baada ya kueleweshwa hata baada ya majadiliano na mwanatimu wa uchunguzi basi hafai kushiriki kwenye uchunguzi lakini anaweza kumwona afisa tiba na kupata huduma ya VCT

Wahudhurio wote wafaao waliokubali (washiriki) watapata faili ya plastiki ikiwa na kidadisi kikuu na vibandiko vya ziada (ST5- 11). Halafu watajipanga foleni kuhojiwa na mhojaji wa uchunguzi.
Kitabu cha uandikishaji na orodha A3 yenye vibandiko zitunzwe vizuri na STL hadi timu itakaporudi Mwanza.

3.3.3 Mahojiano ya ana kwa ana

Watu wafaao waliokubali watahojiwa na mhojaji wa uchunguzi (SI)Mhojaji atakuwa na jinsia ileile ya mhojiwa na atafanana naye kiumri kwa kutumia kidadisi cha ana kwa ana (kidadisi kikuu).Mhojaji atakuwa na jinsia ileile ya mhudhurio na atafanana naye kiumri SI atafuata taratibu za kawaida za utekelezaji kwa ajili ya mhojaji wa uchunguzi. **(Kiambatisho cha 21: SOP wa mhojaji wa uchunguzi).**Mahojiano yafanyike kwenye eneo lisilo na watu na vidadisi vitunzwe vizuri wakati wote. Baada ya kukamilisha mahojiano mshiriki arudishiwe kidadisi kikuu halafu aelekezwe kwa mfanyakazi wa maabara.

3.3.4 Maabara

Mfanyakazi wa maabara atamsalimia mshiriki na atathibitisha kwamba Kibandiko kwenye kidadisi kikuu kinakwenda sambamba na vile vilivyomo kwenye faili. Halafu wafanyakazi wa maabara wataendelea kuchukua damu na haja ndogo kwa ajili ya kupima STI na kipimo cha uchunguzi wa CD4 kufuatana na taratibu za utekelezaji wa kawaida za wafanyakazi wa maabara **(Kiambatisho cha 22: SOP ya mfanyakazi wa maabara)**. Neli za sampuli ya damu ziwe na vibandiko (ST5-7)na vilevile neli za haja ndogo (ST 8-9).

Wafanyakazi wa maabara watapima haja ndogo kuona kama mna RBC kwa kutumia vijiti vya kuingiza. Mhojiwa apewe kidadisi na vibandiko vilivyobaki aende kwa afisa tiba

3.3.5 Afisa tiba

Wajibu wa kwanza wa afisa tiba ni kuhakikisha kwamba washiriki wa uchunguzi wanapata tiba inayofaa kwa tatizo lolote walilo nalo. Kwa hiyo wakati wa uchunguzi afisa tiba atawaona washiriki wa uchunguzi tu pamoja na vijana waalikwa wanaofaa kushiriki kwenye uchunguzi. Baada ya uchunguzi kukamilika afisa tiba aweza kuwaona wahudhurio wengine, lakini asitibu wanajamii wengine wasioshiriki kwenye uchunguzi.

Afisa tiba atapokea vidadisi na vibandiko viwili (ST10, ST11) kutoka kwa mshiriki. Kwanza afisa tiba atamwuliza mshiriki dalili za ugonjwa alizo nazo wakati huu. Taarifa ya tiba huelezwa kwenye **Kiambatisho cha 23 Taarifa za tiba**, na maelekezo ya afisa tiba kwenye **Kiambatisho cha 24 SOPsya afisa tiba .** Madawa yote yafungiwe na kuwekewa maelezo kabla ya timu kutoka Mwanza. Afisa tiba asipoteze muda kuhesabu vidonge au kutafuta madawa.

Wanaume wote wanaoeleza dalili za STIs watafanyiwa uchunguzi wa sehemu za siri kwa nje.(Kiambatisho 23) Upande wa wanawake, wale tu wanaosema wana uvimbe watachunguzwa. Wanawake wachunguzwe wakilalia godoro chini/ kitandani au kiti na wanaume wachunguzwe wakisimama. Matokeo yajazwe kwenye sanduku la matokeo. Washiriki wote wanaotibiwa kufuatana na STI waulizwe kuhusu washiriki wao na wapewe vipeperushi vya kuwapeleka sehemu (referral slips) kwa ajili yao. Kwa kawaida afisa tiba hawezi kuwaona washiriki wa ngono wa wahudhurio. Wao waende kwenye kituo cha afya au hospitali iliyo karibu kupata tiba. Matokeo ya STD na matibabu yote yanayotolewa yaandikwe kwenye fomu ya STD.**(Kiambatisho cha 40:Fomu ya uchunguzi na tiba ya STD)**

Afisa tiba ataeleza kwamba Herpes Simplex Virus 2 ni ugonjwa wa zinaa usioponeka ila hutibika. Ataeleza kwamba vijana wengi tayari wanao ugonjwa huo na ataeleza dalili zake. Atawatia moyo washiriki kwenda kwenye taasisi ya afya wakiwa na uvimbe wa sehemu za siri kwa baadaye.

Hatimaye afisa tiba aangalie orodha ya QC **(Kiambatisho: xx)** inayowataja wanaotakiwa (nambari ya kibandiko) wanaotakiwa kujaza kidadisi cha kuhakikisha ubora **Kidadisi cha kuhakikisha ubora (Kiambatisho xxx:Kidadisi cha kuhakikishia ubora)** kimechapishwa kwenye karatasi yenye rangi na kina baadhi ya maswali ya kidadisi kikuu. Afisa tiba aweke kibandiko **(ST10)** kwenye kidadisi cha QC na ampeleke mshiriki kwa mhojaji wa uchunguzi wa jinsia yake yaani kwa SI ambaye hajamhoji mshiriki kabla ya hapo.

3.3.6 Ushauri na upimaji wa hiari wa HIV (VCT)

Ushauri na upimaji wa hiari wa HIV utafanyiwa na washauri wa VCT wanaostahili(VC) **(Kiambatisho x: SOPs ya VCT)**. Ushauri kabla ya kupima utatolewa na VC. Damu ya wanaoomba kujua hali yao ya HIV itachukuliwa papohapo kwenye eneo la uchunguzi kwa kutumia vipimo viwili tofauti. (Bioline na Determine). Ushauri baada ya upimaji utatolewa kabla ya kuelezwa matokeo. Ikiwa vipimo vya haraka vinagongana mshiriki ataambiwa kwamba inabidi asubiri matokeo ya uchunguzi mwingine utakaofanyika

NIMR, Mwanza (double ELISA) na kwamba mwanatimu atarudi haraka iwezekanavyo na matokeo yao.

Wote wanaoamua kupata VCT wataambiwa kwamba uchunguzi wa kuthibitisha utafanyika na damu yao NIMR, Mwanza na kama kuna tatizo watatafutwa. Wataambiwa kwamba uwezekano wa uchunguzi wao kuwa na utata ni mdogo.

VC atajaza fomu ya matokeo ya VCT **(Kiambatisho x:matokeo ya VCT)** na ataweka kibandiko **(ST11)** kwenye fomu. Matokeo ya VCT yatakuwa na kibandiko cha uchunguzi bila jina wala taarifa zozote zingine za kuonyesha taarifa hizo ni za nani.

Wote watakaokutwa na HIV watapelekwa kwenye taasisi ya afya iliyo karibu yenye huduma ya ART ili kuona kama wanapaswa kupewa ARVs.Ikiwa kuna kikundi cha kutoa matunzo majumbani wataunganishwa na kikundi hiki ili wapate ushauri na huduma ya chakula bora.

Vibandiko visivyotumika (ST10 na/au ST11) vitunzwe kwenye bahasha na kurudishwa kwenye sehemu ya data NIMR wakati mmoja na sampuli na vidadisi.

3.3.7 mchoro wa hatua kwa hatua wa uchunguzi

Umbo 3.1 huonyesha hatua zitakazopitiwa na yeyote antakayefikia eneo la uchunguzi na muda uliokisiwa kwa kila hatua.Kwa kuzngatia muda wa kusubiri uchunguzi utachukua masaa 1.5- 2.5 kwa wanaoshiriki kwenye uchunguzi.

Umbo 3.1: mchoro wa hatua kwa hatua wa uchunguzi

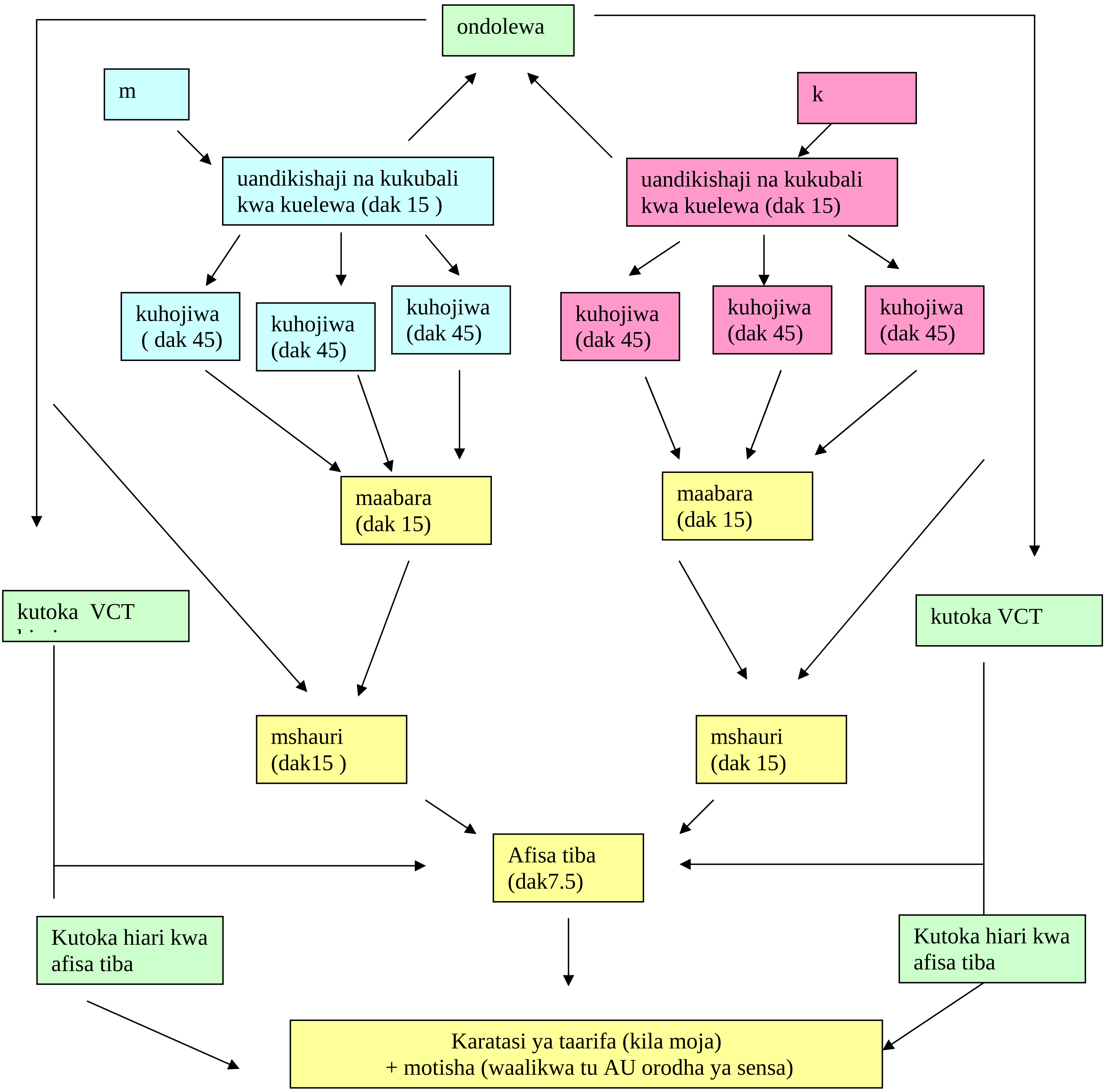

3.3.9 Motisha kwa mshiriki

Washiriki wakishamaliza uchunguzi warudi kwenye uandikaji kupokea motisha yao.**Jedwali 3.3** huonyesha gharama ya usafiri na motisha zitakazotolewa kwa vikundi mbalimbali vya walio hudhuria.

Jedwali 3.3: Motisha kwa wahudhurio

	**Mwaaliko *AU* kwenye orodha ya sensa**	**afaao kwa uchunguzi**	**Tiba ya STI**	**VCT**	**motisha na posho ya usafiri**
1	Ndiyo	Ndiyo	ndiyo	ndiyo	4000 Tsh + Kipande kikubwa cha sabuni (thamani ya 1000 Tsh)
2	Ndiyo	Hapana	ndiyo	ndiyo	2000 Tsh +nusu kipande cha sabuni (thamani ya 500 Tsh)
3	hapana	Ndiyo	ndiyo	ndiyo	4000 Tsh + Kipande kikubwa cha sabuni
4	hapana	hapana	Kama kuna muda baada ya kuwahudumia vikundi vingine vya wahudhurio		hamna

3.3.8 Ufuatiliaji

Kila timu ya uchunguzi ina wafuatiliaji watano watakaowajibika kuhakikisha kwamba vijana watakaopatikana kwenye sensa washiriki kwenye uchunguzi. Siku ya kwanza ya uchunguzi kijijini timu ya ufuatiliaji watasafir kwa landcruiser ya mradi, baisikeli au kwa miguu kwenye eneo wanapokaa waalikwa wa uchunguzi wa siku ile.(**Kiambatisho xx: SOPs wa wafuatiliaji)** Watawahamasisha vijana waalikwa wa eneo husika kufika kwenye sehemu ya uchunguzi.

Siku ya pili ya uchunguzi watawahamasisha walioalikwa siku ya pili na watawafuatilia wale ambao hawakufika siku ya kwanza. Siku ya tatu watawahamasisha walioalikwa kuhudhuria uchunguzi siku ya tatu na kuwafuatilia ambao walipaswa kufika siku zilizopita.

3.3.9 Taarifa ya uchunguzi na usimamizi wa wafanyakazi wa field

Viongozi wa timu ya sensa na viongozi wa timu ya uchunguzi watakamilisha fomu za muktasari za kijiji **(Kiambatisho xxx:Fomu ya muktasari wa sensa ya kijiji Kiambatisho xxx:Fomu ya muktasari wa uchunguzi wa kijiji).** Fomu hizo zitarudishwa Mwanza wakati ujao wa kukusanya vipimo na data na zitatumika na NIMR kusimamia maendeleo.

Siku moja baada ya kurudi Mwanza kiongozi wa timu (sensa na uchunguzi) akutane na FS na FM kuwapa taarifa ya uhamasishaji ya kata. CTL na STL watatoa taarifa fupi ya maandishi kwa timu ya MkV1 FS ndani ya wiki moja baada ya kurudi kutoka kata. Ripoti iwe na taarifa kuhusu kijiji/vitongoji vilivyotembelewa, siku zilizotumika kila sehemu, idadi za kaya zilizotembelewa, idadi ya vijana walivyohojiwa na matatizo yaliyotokea nk **(Kiambatisho xx: Fomu ya muktasari wa sensa ya kata; Kiambatisho xx:Fomu ya muktasari wa uchunguzi kata).**

Mratibu wa mradi (PC) aliyoko NIMR, Mwanza, meneja ya kazi ya field (FM) na msimamizi wa field watashirikiana kwenye usimamizi wa mwanzoni wa timu ya field, yaani jamii chache za mwanzoni. Watasafiri na timu ya field na kusimamaia uandaaji wa kituo cha uchunguzi, hatua zote za uchunguzi pamoja na ukusanyaji wa vipimo na data na kila hatua ya uchunguzi. Watawasimamia pia wafanyakazi wa field wa sensa, hasa hasa watahakikisha timu isiwe na shida ya kutumia PDAs na kutengeneza orodha ya washiriki wa uchunguzi.

Baada ya timu ya sensa na uchunguzi kumaliza jamii chache za kwanza usimamizi wa field utafanywa na msimamizi wa field (atatumia 75% ya muda wake akiwa field) atawatembelea timu ya sensa na uchunguzi kila wiki kuwasimamia. Kila anapowatembelea apeleke mahitaji kwa ajili ya timu zote mbili yakiwemo vifaa vya uchunguzi pamoja na chakula na mahitaji binafsi.

FS atalenga kutembelea timu moja ya field kila wiki na atakaa siku moja na timu ya sensa na siku moja na timu ya uchunguzi. Atahudhuria baadhi ya mahojiano na kuandaa muktasari wa matokeo muhimu. FS ataendesha baadhi ya mahojiano ya kuthibitisha ubora na vijana. Atajulisha kiongozi wa timu ikiwa kuna sehemu inayohitaji kuboreshwa. FS atahakikisha viongozi wa timu wajaze fomu za muktasari unavyotakiwa. Wakati FS anapotembelea field atajadili tatizo lolote linalohusu sensa na uchunguzi na viongozi wa pale. OC na FM watamsindikiza FS mara kwa mara anapotembelea eneo la field.

3.4 vitendo vingine vya field

3.4.1 ukusanyaji wa sampuli

Damu na haja ndogo zitatengenezwa na kufungiwa kwa ajili ya kusafrishwa na fundi maabara wa field. **(Kiambatisho xx: SOPs wa ukusanyaji wa sampuli na data).**Landrover ya ziada litatumika kukusanya sampuli na litaenda kukutana na timu na kukusanya sampuli mara moja kwa wiki (kusanya kama183 sampuli ya damu na183 sampuli ya haja ndogo). Usafrishaji hadi NIMR, Mwanza itakuwa kwenye jokovu la kubeba au sanduku la kutunza baridi lenye barafu ili sampuli zitunzwe kwa 4°CFomu ya jumla ya sampuli **(Kiambatisho xxx:Fomu ya jumla ya sampuli)** zijazwe kwa sampuli zote zinazotumwa Mwanza na ziwekewe sahihi na dereva anayepeleka sampuli na msaidizi wa maabara anayepokea sampuli NIMR.

3.4.2 Ukusanyaji wa data

Vidadisi vilivyojazwa na fomu zingine (zikiwemo fomu za muktasari za sensa na uchunguzi) zitakusanywa wakati mmoja na vipimo vya maabara **(Kiambatisho xx SOPs ukusanyaji wa sampuli na data)**. Fomu hizo zitapelekwa kwenye sehemu ya data ya MITU zitaingizwa mara mbili. Fomu zote na vidadisi vinavyotumwa NIMR lazima ziende

na fomu ya jumla ya data. **(Kiambatisho xx:Fomu ya jumla ya Data)** zilizosainiwa na dereva anazozipeleka na meneja wa data anazozipokea NIMR.

3.4.3 kazi ya kumalizia

Kiwango na nguvu za kazi za kumalizia (kmf kutembelea jamii ya uchunguzi kwa mara nyingine , kutembelea sehemu kuu ya uhamiaji) itategemea na idadi ya vijana watakaoshiriki kwenye uchunguzi.

3.4.4 matibabu ya STI baada ya uchunguzi

Maabara ya NIMR, Mwanza na kitengo ch data watatoa orodha ya washiriki watakaokutwa na STI inayopimika (kaswende hai au NG au CT) ndani ya wiki 5 baada ya kupokea sampuli ya maabara. Mwanatimu aliyechaguliwa atarudi kwenye jamii miezi miwili baada ya uchunguzi kutoa matibabu kwa watu wale. Wataeleza kwamba wanafuatilia uchunguzi na watajadili matokeo ya STI na matibabu peke yake na washiriki.

Watakaokutwa na HSV2 hawatatembelewa kwa sababu ni tatizo lisiloponeka ila linatibika. Wakati wa uchunguzi afisa tiba atawaeleza washiriki wote umuhimu wa kwenda kwenye taasisi ya afya wakipatauvimbe sehemu za siri.

Ikiwa kuna tofauti isiyotegemewa kati ya VCT na matokeo ya HIV ELISA mshiriki atatembelewa upya. Watapewa matokeo ya ELISA na ushauri zaidi. Wale tu waliokubali VCT wakati wa uchunguzi wataambiwa kama matokeo ya ELISA huonyesha HIV.

3.5 MAANDALIZI

3.5.1 malazi

Timu ya sensa na uchunguzi watalala kwenye nyumba ya malazi au kempu (inategemea na eneo) na watawajibika kutunza vifaa vya field. Kila panapowezekana timu ya field watakaa pamoja.

3.5.2 vitambulisho binafsi

Wanatimu wa uchunguzi watapewa vitambulisho na lazima wawe navyo muda wote. Wafanyakazi wa field wote wanapewa TShirt ili iwe rahisi kuwaona. Wahojaji wa sensa watapewa pia kofia za mradi

3.5.3 Magari ya mradi

Meneja wa kazi ya field na viongozi wa timu watawajibika kuratibu matumizi ya magari ya mradi na kuhakikisha linafaa kutembea barabarani. Dereva atawajibika kuweka mafuta, kufanya matengenezo ya msingi na kuegesha magari panapokuwa na usalama. Dereva afuate sheria za barabara avae mkanda na ahakikishe abiria wavae mkanda (kama upo). Dereva lazima ajaze kitabu cha usafiri kila safari inapokamilika. **(Kiambatisho xxx: SOPs ya dereva).**

MOs wanawajibika kuhakikisha pikpiki zina hali nzuri. Lazima wawe na kitabu kwa ajili ya safari za mbali na wafuate sheria za barabara. Lazima wavae kofia za chuma wanapoendesha pikipiki. (Kiambatisho xx: SOPs ya afisa uhamasaji).

3.5.4 Pesa ya kazi ya field

Wafanyakazi wote wa MkV1 FS wanapewa posho ya malazi wanapolala nje ya Mwanza kwa kazi ya uchunguzi. Kiongozi wa timu atawajibika kusimamia posho ya malazi field na atakuwa na orodha ya watu wanaorudi Mwanza. Kiongozi wa timu atasimamia pia fedha za kumudu ghara ingine za field.

3.5.5 Mawasiliano

Wafanyakazi wakuu wa MkV1 FS watawasiliana kupitia simu za mkononi na watarudishiwa gharama ya kupiga simu/SMS kufuatana na utaratibu wa simu za mkononi za MkV1 FS. SMS zitumike kila unapowezekana.

3.5.6 muda

Tarehe ya kuanza ni mwezi wa 12 2006 na mradi utaendelea kwa muda wa miezi 30. Miezi minne ni kwa ajili ya kuajiri na kufunza timu ya field, pamoja na kujaribishia na kuzifanyia uchunguzi taratibu za uchunguzi. Timu moja itachukua kama miezi minne kufanya sensa na uchunguzi kwenye kila jamii. Muda umewekwa kwa ajili ya kuongeza vifaa na mapumziko mafupi Mwanza baada ya kila jamii na kwa ajili ya kutembelea upya jamii mwisho wa muda wa utafiti kujaribu kuwapata vijana wanaofaa ambao hawakuwepo wakati wa uchunguzi. Kwa hiyo miezi 11.5 ilipangwa kwa ajili ya sensa na uchunguzi mkuu.

Muda	Kitendo
Dec’06-Mar’07	Kumalizia taarifa ya uchunguzi,kuandikisha na kuwafunza timu za field, uchunguzi wa jaribio
May’07–Apr’08	Kukusanya data ya uchunguzi & vipimo
May’08–Sept’08	Kumalizia uchunguzi wa data & uchunguzo wa maabara
Oct’08–May’09	Uchunguzi wa kitakwimu na kuandika, kusambaza matokeo

MkV1FS Prot A1_Swahili protocol for training .doc

4. Usimamizi wa data

Database itatengenezwa kwa mfumo mpya wa *David Beckels na* wanachama wa sehemu ya data NIMR, Mwanza.Ukaguzi wa data na ukaguzi wa udhabiti utakuwa sehemu ya kila faili itakayoingizwa na wafanyakazi wa kuingiza data. Hakuna majina wala vitambulisho vingine vitatunzwa kwenye kompyuta. Data zote zitaingizwa mara mbili na uthabiti utahakikishwa.

Data za uchunguzi wa kujaribishia zitaingizwa na kuchunguzwa kupima ufanisi wa vidadisi na fomu. Data za uchunguzi mkuu zitaingizwa ndani ya wiki mbili baada ya kuzipokea. Kwanza vidadisi vikuu vitaingizwa. Vidadisi vingine na fomu zitaingizwa baadaye na taarifa itarudishwa kwa wanaoingiza data pamoja na wafanyakazi wa field kuhusu ubora wa data zilizopokelewa.

5. Uchunguzi wa maabara

Vipimo vyote muhimu vitafanyika kwenye maabara ya NIMR STI

HIV itapimwa kwa kutumia vipimo viwili vya kujitegemea vya ELISA (Murex, Vironostika), pamoja na kuthibitishwa kupitia p24 antigen ELISA na Western Blot (Inno-Lia), panapohitajika **(Kiambatisho xxx: HIV - algorithm)**.

Kingamwili ya HSV2 itapimwa kutumia kipimo cha kalon cha HSV2-specific ELISA test. Kipimo cha TPPA kitatumika kugundua kaswende – inapoonekana kaswende hai itapimwa kupitia kipimo cha RPR. Kingamwili cha NG na CT kitapimwa kupitia PCR (Amplicor), kwanza sampuli 5 kwa pamoja ikiwa kingamwili kitaonekana kila sampuli kitapimwa peke yake.

Kazi ya maabara itafanyika ndani ya wiki 3 baada ya kuchukua sampuli. Matokeo ya maabara yataingizwa kwenye kompyuta na wafanyakazi wa kuingiza data. Matokeo yataunganishwa kwa kutumia nambari ya vibandiko. Data ya maabara kutoka sehemu zingine zitalinganishwa na kuunganishwa na matokeo ya vidadisi vikuu..

7. Mawasiliano na Usambasaji

MkV1 Further Survey kwa kushirikiana na MkV2 waliunda mbinu ya mawasiliano ya MkV inayoeleza hatua zitakazochukuliwa kusambaza matokeo ya jaribio. Wadau wa kimataifa hadi wa kijijini watahusika na taarifa zitatengenezwa kulingana na mahitaji yao. Kamati ya ushauri wa MkV itafufuliwa na itatoa ushauri kwa waratibu wa jaribio.

8. Marejeo
9. Kiambatisho

Kiambatisho cha 1: Taarifa (Swahili)

Matokeo ya tasmini ya MkV1 ya mwaka wa tatu

Kiambatisho cha 2: mgawanyo wa mkondo wa mikakati na kulinganisha

Kiambatisho cha 3: Ratiba ya kwanza ya mafunzo ya wafanyakazi ya *field*

Kiambatisho cha 4: Kidadisi ya sensa

Kiambatisho cha 5: Kidadisi kikuu

Kiambatisho cha 6 : Kidadisi kikuu-usimamizi wa ubora

Kiambatisho cha 7.1: orodha ya QC

Kiambatisho cha 7.2 : Fomu ya field

Kiambatisho cha 8: orodha ya kufungasha

Kiambatisho cha 9.1:Afisa Uhamasishaji SOPs

Kiambatisho cha 9.2: Ratiba ya uchunguzi ulivyopangwa

Kiambatisho cha 10: Karatasi ya taarifa ya uchunguzi kwa ajili ya wahusika rasmi

Kiambatisho cha 11: Orodha ya kaya Kitongoji

Kiambatisho cha 12: Fomu ya taarifa ya MO

Kiambatisho cha 13: SOPs ya kiongozi wa timu ya sensa

Kiambatisho cha 14: SOPs ya Mhojaji wa sensa

Kiambatisho cha 13.2:orodha ya kufungasha ya timu ya sensa

Kiambatisho cha 14.2: Karatasi ya kukubali baada ya kueleweshwa kwa ajili ya kaya

MkV1FS Prot A1_Swahili protocol for training .doc

Kiambatisho cha 15: Mwaaliko wa uchunguzi

Kiambatisho cha 16: Fomu ya kuhama

Kiambatisho cha 17:Fomu ya karatasi ya sensa ya kaya

Kiambataisho cha 29: Fomu ya jumla ya sensa ya kijiji

Kiambatisho cha 18: orodha ya kufungasha kwa timu ya uchunguzi

Kiambatisho cha 19.1: karatasi ya taarifa kwa washiriki

Kiambatisho cha 19.2: SOPs ya mhojaji wa uandikishaji

Kiambatisho cha 20.1: Maswali ya kuthibitishia kukubali baada ya kueleweshwa

Kiambatisho cha 20.2: Fomu ya washiriki ya kukubali baada ya kueleweshwa

Kiambatisho cha 21: SOPs ya mhojaji wa kuandikisha

Kiambatisho cha 22: SOPs ya mhojaji wa uchunguzi

Kiambatisho cha 23: SOPs ya wafanyakazi wa maabara

Kiambatisho cha 24: Taarifa za matibabu

Kiambatisho cha 25: SOPs za afisa tiba

Kiambatisho cha 26: VCT SOPs

Kiambatisho cha 27: SOPs ukusanyaji ya data na vipimo

Kiambatisho cha 28: Fomu ya jumla ya sampuli

Kiambatisho cha 29: Algorithem ya kupima HIV

Kiambatisho cha 30: Fomu wa muktasari ya uchunguzi

Kiambatisho cha 31: Fomu ya jumla ya data

MkV1FS Prot A1_Swahili protocol for training .doc

Karatasi ya muktasari wa kila kijiji kinachotembelewa wakati wa sensa
Mpangilio wa uchunguzi, zikiwemo jedwali za kujazia

Annex 2: Results of 3-year MkV1 Evaluation Survey: Impact on knowledge, reported attitudes, reported behaviours, and biological outcomes by sex

Outcome	Male			Female		
	Frequency[1]		Adjusted RR[2] (CI)	Frequency[1]		Adjusted RR[2] (CI)
	Intervention (N=2076) n (%)	Comparison (N=2024) n (%)		Intervention (N=1448) n (%)	Comparison (N=1492) n (%)	
**Knowledge** (% with all 3 responses "correct")						
HIV acquisition	1356 (65%)	908 (45%)	1.44 (1.25,1.67)	832 (58%)	601 (40%)	1.41 (1.14,1.75)
STD acquisition	1074 (52%)	807 (40%)	1.28 (1.07, 0.54)	522(36%)	376 (25%)	1.41 (1.06,1.88)
Pregnancy prevention	1746 (84%)	1018 (50%)	1.66 (1.55,1.78)	1047 (72%)	688 (46%)	1.58 (1.26,1.99)
**Reported Attitudes** (% with all 3 responses "correct")						
Attitudes to sex	454 (22%)	247 (12%)	1.77 (1.42,2.22)	383 (27%)	283 (19%)	1.42 (1.11,1.81)
**Reported Sexual Behaviour** (% with outcome)						
Sexual debut during follow-up[3]	638 (60%)	677 (72%)	0.84 (0.71,1.01)	801 (68%)	763 (67%)	1.03 (0.91,1.16)
More than 1 partner in last 12 months	394 (19%)	556 (28%)	0.69 (0.49,0.95)	123 (9%)	116 (8%)	1.04 (0.58,1.89)
First used condom during follow-up[4]	548 (39%)	427 (28%)	1.41 (1.15,1.73)	387 (38%)	297 (28%)	1.30 (1.03,1.63)
Used condom at last sex[5]	431 (29%)	326 (20%)	1.47 (1.12,1.93)	284 (27%)	238 (22%)	1.12 (0.85,1.48)
Went to health facility for most recent STI symptoms within the last 12 months[6]	26/91 (29%)	52/150 (35%)	0.84 (0.50,1.41)	33/93 (36%)	54/160 (34%)	1.02 (0.62,1.70)
**Primary biological outcomes**						
HIV incidence (/1,000py)	3 (0.43)	2 (0.30)	NA	16 (3.18)	24 (4.73)	0.75 (0.34,1.66)
HSV-2 prevalence	234 (11.3%)	251 (12.5%)	0.92 (0.69,1.22)	305 (21.3%)	309 (20.8%)	1.05 (0.83,1.32)
**Secondary biological outcomes**						
Syphilis prevalence	28 (1.4%)	37 (1.8%)	0.78 (0.46,1.30)	47 (3.3%)	54 (3.6%)	0.99 (0.67,1.46)
Chlamydia prevalence	11 (0.5%)	11 (0.5%)	1.14 (0.53,2.43)	71 (4.9%)	54 (3.6%)	1.37 (0.98,1.91)
Gonorrhoea prevalence	8 (0.4%)	2 (0.1%)	NA	35 (2.4%)	18 (1.2%)	1.93 (1.01,3.71)
Trichomonas prevalence[7]				413 (28.6%)	383 (25.8%)	1.13 (0.92,1.37)
Pregnancy (test) prevalence[7]				277 (19.2%)	268 (18.0%)	1.09 (0.85,1.40)
Reported pregnancy during follow-up[7,8]				489 (46.9%)	489 (45.5%)	1.03 (0.89,1.20)

Key:

1. Prevalence, risk or rate depending on outcome
2. Adjusted for: Age group (≤17, 18, ≥19 years at final survey), stratum, tribe (Sukuma vs non-Sukuma), number of lifetime partners at baseline (0, 1, 2, ≥3)
3. Among those who reported never having had sex at recruitment
4. Among those who reported having had sex at the final round, who had not reported ever using a condom at recruitment
5. Among those who reported having had sex at the final round
6. Among those reporting STI symptoms within the last 12 months
7. Females only
8. Among those who reported never having been pregnant at recruitment

NA Number of cases too small to justify comparison (<10 in each group)

Annex 3: Allocation to Intervention (I) and Comparison (C) arms for analysis

	**'*de jure*' residence**	**Primary Schools attended**	**Probability of…**		**Time spent in primary school std 5, 6 or 7 (1999-2002)**		**Impact**
			**Scenario**	**Locating population**	**>=6 months of 1 yr**	**< 6 months of 1 yr**	
1	Intervention (I) community	I only	High	High	I arm	Exclude	
2		I/ non-trial	Medium	Depends on current location	I arm	Exclude	Underestimate impact depending on length of time outside intervention community
3		I/ C	Low	High	Exclude	Exclude	Decrease size of eligible population
4		I/ C/ Non-trial	Low	Depends on current location	Exclude	Exclude	Decrease size of eligible population
5	Comparison (C) community	C only	High	High	C arm	Exclude	
6		C/ Non-trial	Medium	Depends on current location	C arm	Exclude	Underestimate impact exposed to other ASRH intervention in non-trail community
7		C /I	Low	High	Exclude	Exclude	Decrease size of eligible population
8		C/I/ Non-trial	Low	Depends on current location	Exclude	Exclude	Decrease size of eligible population

	**MkV1 FS - Field Staff Training Schedule (WEEK 1)**				
	**Monday 23rd April 07**	**Tuesday 24th April 07**	**Wednesday 25th April 07**	**Thursday 26th April 07**	**Friday 27th April 07**
**8:30-9:00**	Introduction (AD / MJ)	Protocols, good research practice, data flow (MJ)(GM, GS, RM)	Questionnaires (MJ)	Introduction to ethics (BA)	Community entry techniques and how to behave in the field/community (BA, MH)
**9:00-9:30**	Introduction to health research (MJ)	MkV1 FS Study Protocol (MH)		Interviewing techniques/skills (EK, MH)	**0915- 10:00** Test for FWs
**9:30-10:30**	Overview of MkV (MH, GM, RM) (BA, NS, GS)	Individual reading assignment on protocols (MJ, MH, AD)	Standard letters/numbers & questionnaire instructions (RB, EK)		**10:00** Lab submission (DM)
**10:30-11:00**			**Breakfast**		
**11:00-11:30**	What are HIV, AIDS and STIs? (MJ)	Group Activity- Components of MkV FS (MJ, AD,MH, LM, GM, GS, RM, NS)	MkV1 FS questionnaire- Demographics (MH, EK)	MkV1 FS questionnaire- sexual behaviour (MH, EK)	MkV1 FS questionnaire- other sections (MH, EK)
**11:30-12:30**	Introduction to MkV1 Further Survey (MJ)				
**12:30- 1:00**	Questions and Answers (MH, MJ, AD, NS, GS, GM, RM)				
**1:00-2:00**			**Lunch**		
**2:00:-2:30**	The challenges of discussing sexual behaviour (MH)	Presentations of group work (MJ, AD,MH, LM, GM, GS, RM, NS)	Practical- Questionnaire (Demog) (MH, RB, EK)	Practical- Questionnaire (sex behav) (MH, RB, EK)	Question and Answers (MH, MJ, EK, RB)
**2:30-3:00**	Group activity- Sexual Terms (ND, MR, MH, MJ, LM, TM, DG, EK)				
**3:00-4:00**	Group presentations (ND, MR, MH, MJ)	Group Discussion (MJ, AD,MH, LM, GM, GS, RM, NS)	Questions and Answers (MH, MJ, EK, RB)	Questions and Answers (MH, MJ, EK, RB)	Feedback on test and performance with questionnaire

**4:00- 4:30**	Summary of day (AD/MJ)	Summary of day (AD/MJ)	Summary of day (AD/MJ)	Summary of day (AD/MJ)	Summary of week and selection of trainees for week 2 (AD/MJ)
	**MkV1 FS - Field Staff Training Schedule (WEEK 2)**				
	**Mon 30th April 07**	**Tues 1st May 07**	**Wed 2nd May 07**	**Thurs 3rd May 07**	**Fri 4th May 07**
**8:30-9:00**	Introduction (AD / EM)	BANK HOLIDAY- NO TRAINING	MkV1 FS Census procedures, SOPs (MH)	Introduction to GPS (EM/KS)	Charging PDA (EM/KS)
**9:00-9:30**	Introduction to PDA (EM/KS)		Census questionnaire - intro to paper version (MH)	GPS Practical: Introduction, recording co-ordinates (EM/KS)	**Group 1**: PDA test<br>**Group 2:** Census procedures
**10:00-10:30**	Questions and Answers		Census questionnaire- individual reading (MH)		**Group 1:** Census procedures<br>**Group 2:** PDA test
**10:30-11:00**	**Breakfast**				
**11:00-12:00**	PDA Practical: Introduction, Alphabet practice		Practical: Scenario-1<br>**Group 1:** PDA Census questionnaire<br>**Group 2:** Paper census questionnaire	Practical: scenario-1<br>**Group 1:** PDA Census questionnaire/PDA<br>**Group 2:** Main questionnaire/paper	Demonstration of synchonisation, CD burning and generation of lists for survey (EM/KS)
**12:00-1:00**					
**1:00-2:00**	**Lunch**				
**2:00-3:30**	PDA Practical: CV		Practical: scenario-2<br>**Group 1:** Paper Census questionnaire<br>**Group 2:** PDA Census questionnaire	Practical: scenario-2<br>**Group 1:** Main questionnaire<br>**Group 2:** PDA Census questionnaire	Feedback on PDA test and practicals (error rates) (EM/KS)
**3:30- 4:00**	Questions and Answers				Questions and Answers
**4:00- 4:30**	Summary of day (AD/MJ)		Summary of day (AD/MJ)	Summary of day (AD/MJ)	Summary of week and selection of trainees to join MkV1 FS teams (AD/MJ)

MEMA kwa Vijana Trial Further Survey (2007-2008)

District	Ward	Code	Village	Code	Schools	Code
SENGEREMA	Katwe	02	Katwe	001	Katwe	201
			Mwangika	002	Mwangika	202
			Luhama	003	Luhama	203
			Kahunda	004	Kahunda	204
			Kasheka	005	Kasheka	205
	Nyehunge	03	Nyehunge	006	Nyehunge	206
			Isaka	007	Isaka	207
			Kayenze	008	Kayenze	208
			Nyamadoke	009	Nyamadoke	209
	Nyakalilo	04	Nyakaliro	010	Nyakaliro	210
			Lumeya	011	Lumeya	211
			Bukokwa	012	Bukokwa	212
			Sukuma	013	Sukuma	213
			Bukokwa	012	(Nyabutanga)	214
	Katunguru	05	Katunguru	014	Katunguru	215
			Katunguru	014	(Bugalama)	216
			Nyamtelela	015	Nyamtelela	217
			Kasomeko	016	Kasomeko	218
			Chambanda	017	Chamabanda	219
			Chambanda	017	(Igalagalilo)	220
			Nyamililo	018	Nyamililo (Kasungamile ward)	221
	Busisi	06	Busisi	019	Busisi	222
			Busisi	019	(Mkomba)	223
			Nyitundu	020	Nyitundu	224
			Nyamasale	021	Nyamasale	225
			Kahumulo	022	(Lubanda)	226
			Lubanda	118	NB Lubanda 118 can be recoded to Kahumulo 022	
			Nyampande	023	Nyampande (Tabaruka ward)	227
			Kahumulo	022	Kahumulo	228
MISSUNGWI	Misasi	18	Misasi	025	Misasi	229
			Manawa	026	(Manawa)	230
			Mwasegela	027	Mwasegela	231
			Inonelwa	028	Inonelwa	232
	Usagara	21	Usagara	029	Busagara	233
			Fela	030	Fela	234
			Nyang'homango	031	Nyang'homango	235
			Bujingwa	032	Fulo	236
			Ng'wasonge	033	Mayolwe (Idetemia ward)	237
	Koromije	22	Koromije	034	Koromije	238
			Ibongoya "A"	035	Ibongoya	239
			Bugomba	036	Bugomba	240
			Mamaye	037	Mamaye	241
			Mwalwigi	038	Mwalwigi	242
			Ibongoya "B"	039	Nyabugeni	243

MEMA kwa Vijana Trial Further Survey (2007-2008)

District	Ward	Code	Village	Code	Schools	Code
**GEITA**	Katoro	**08**	Katoro	**040**	Katoro	**244**
			Chibingo	**041**	Chibingo	**245**
			Nyamigota	**042**	Nyamigota	**246**
			Iyara	**043**	Iyara	**247**
			Ibondo	**044**	Ibondo	**248**
			Kasesa	**045**	Kasesa (Kasema ward?)	**249**
	Kagu	**09**	Kasota	**046**	Kasota	**250**
			Bugulula	**047**	Bugulula	**251**
			Nyamwilimilwa	**048**	Nyamwilimilwa	**252**
			Kagu	**049**	Kagulumulwina	**253**
			Nyamilyango	**050**	Nyamilyango	**254**
	Lubanga	**10**	Lubanga	**051**	Lubanga	**255**
			Mtakuja	**052**	Mtakuja	**256**
			Ng'wabagalu	**053**	Ng'wabagalu	**257**
			Isulwabutundwe	**054**	Isulwabutundwe	**258**
			Nyakaduha	**055**	Nyakaduha	**259**
			Igaka	**056**	Igaka	**260**
			Ibisabageni	**057**	Ibisabageni	**261**
			Mwamitilwa	**117**		
	Kasamwa	**11**	Kasamwa	**058**	Kasamwa	**262**
			Kasamwa	**058**	(Nyampa)	**263**
			Ibanda	**059**	Ibanda	**264**
			Ibanda	**059**	(Mwilima)	**265**
			Chabulongo	**060**	Chabulongo	**266**
			Nyamahuna	**061**	Nyamahuna (not cohort)	**267**
			Mshinde	**062**	Mshinde (not cohort)	**268**
			Nyalubele	**063**	Nyalubele (not cohort)	**269**
			Bung'wangoko	**064**	Bung'wangoko	**270**
	Ihanamilo	**12**	Bunegezi	**065**	Bunegezi	**271**
			Nyakato	**066**	Nyakato	**272**
			Nyanguku	**067**	Nyanguku	**273**
			Shinyamwenda	**068**	Shinyamwenda	**274**
			Igenge	**069**	Igenge	**275**
			Ikulwa	**070**	Ikulwa	**276**
			Mwagimwagi	**071**	(Mwagimwagi)	**277**
	Bukoli	**13**	Bugogo	**072**	Bugogo	**278**
			Wigo	**073**	Wigo	**279**
			Nyaruyeye	**074**	Nyaruyeye	**280**
			Nyaruyeye	**074**	(Ikandilo)	**281**
			Ikina	**075**	Ikina	**282**
			Muhama	**076**	Mhama-Lwina	**283**
	Nyang'whale	**14**	Nyang'whale	**077**	Nyang'whale	**284**
			Nyarubele	**078**	Nyarubele	**285**
			Nyijundu	**079**	Nyijundu	**286**
			Nyaruguguna	**080**	Nyaruguguna	**287**
			Iyogelo	**081**	Iyogelo (not cohort)	**288**
			Kaseme	**082**	Kaseme	**289**
			Nyakaswi	**083**	Nyakaswi	**290**
			Ibambila	**084**	Ibambila	**291**
	Nkome	**01**	Nkome	**085**	Nkome	**292**
			Nkome	**085**	(Ihumilo)	**293**
			Katoma	**086**	Katoma	**294**
			Katoma	**086**	(Nyakazeze)	**295**
			Katoma	**086**	(Itale)	**296**
			Nyamboge	**087**	Nyamboge	**297**

District	Ward	Code	Village	Code	Schools	Code
**Kwimba**	Mwagi	**23**	Mwabilanda	**088**	Mwabilanda	**298**
			Ligembe	**089**	Ligembe	**299**
			Ligembe	**089**	(Mwagi)	**300**
			Kishili	**090**	Kishili	**301**
			Mwamajiro	**091**	Mwamajiro	**302**
			Mwaginghi	**092**	Mwaginghi	**303**
			Nkalalo	**093**	Nkalalo	**304**
			Mwanawe	**094**	Mwanawe	**305**
			Nyambiti	**095**	Nyambiti (Nyambiti ward)	**306**
	Malya	**24**	Malya	**096**	Malya	**307**
			Kitunga	**097**	Kitunga	**308**
			Mwitambu	**098**	Mwitambu	**309**
			Talaga	**099**	Talaga	**310**
			Lyoma	**100**	Lyoma (Lyoma ward)	**311**
			Kimiza	**101**	Kimiza (Lyoma ward)	**312**
			Busule	**102**	Busule (Lyoma ward; non-coh	**313**
			Nkungulu	**103**	Nkungulu (Lyoma ward)	**314**
	Hungumalwa	**16**	Humgumalwa	**104**	Humgumalwa	**315**
			Buyogo	**105**	Buyogo	**316**
			Kibitilwa	**106**	Kibitilwa	**317**
			Ilula	**107**	Ilula	**318**
			Kabalerunere	**108**	Runere/Ibaya	**319**
			Manayi	**109**	Igaga	**320**
	Fukalo	**17**	Sanga	**110**	Mwamashimba	**321**
			Kawekamo	**111**	Kawekamo	**322**
			Nyang'honge	**112**	Nyang'honge	**323**
			Chibuji	**113**	Chibuji	**324**
			Ndamhi	**114**	Ndamhi	**325**
			Bupamwa	**115**	Bupang'wa (Bupamwa ward)	**326**
			Chasalawi	**116**	Chasalawi (Bupamwa ward)	**327**
**District**	**Ward**	**code**	**Village**	**code**		
**Sengerema**	**Sengerema**	**30**	**Bomani**	**173**		
	**Lugata**	**35**	**Kome Mchangar**	**172**		
	**Bupandwa**	**36**	**Bupandwa**	**162**		
**Geita**	**Kalangalala**	**32**	**Kalangalala**	**174**		
	**Nzera**	**33**	**Lwenzera**	**164**		
	**Rwamgasa**	**31**	**Rwamgasa**	**161**		
**Magu**	**Magu**	**45**	**Magu**	**195**		
**Ngudu**	**Ngudu**	**46**	**Ngugu**	**196**		
**Misungwi**	**Misungwi**	**47**	**Misungwi**	**197**		
**Kahama**	**Kahama**	**48**	**Kahama**	**198**		
**Shinyanga**	**Shinyanga**	**49**	**Shinyanga**	**199**		
**Mwanza**	**Mkolani**	**66**	**Mkolani**	**228**		
	**Nyakato**	**71**	**Buzuruga**	**233**		
	**Pasiansi**	**73**	**Pasiansi**	**238**		

MkV1 FS Protocol Annex 6: Draft Community Survey Timetable (Bukoli)

**MkV1 FS Team 1: Bukoli, Geita (2nd Sept- 28th Sept 2007)**					
**Kijiji**	**Kitongoji**		**# of HH**	**Census**	**Survey**
**A. Bugogo (072)**	**A1**	Buzibila	179	4th-11th Sept	11-14 and 17-18 Sept
	**A2**	Shilungule	134		
	**A3**	Kayenze A	146		
	**A4**	Kayenze B	308		
	**A5**	Bugogo A	219		
	**A6**	Bugogo B	261		
		**TOTAL**	**1247**		
**B. Muhama B (076)**	**B1**	Lwina	63	11th- 13th Sept	15th Sept
	**B2**	Muhama	87		
	**B3**	Ivumvumila	50		
	**B4**	Senga	57		
		**TOTAL**	**257**		
**C. Ikina (075)**	**C1**	Mzalendo	106	12th-14th Sept	19th- 20th Sept
	**C2**	Mwamanyili	109		
	**C3**	Mwendapole	58		
		**TOTAL**	**273**		
**D. Wigo (073)**	**D1**	Busolwa	73	14th-18th Sept	21st-24th Sept
	**D2**	Imalabaniti	79		
	**D3**	Ilelema	56		
	**D4**	Busekwanoni	134		
	**D5**	Wigo A	140		
	**D6**	Wigo B	84		
		**TOTAL**	**566**		
**E. Nyanyeye (074)**	**E1**	Azimio	129	18th-21st Sept	25th-27th Sept
	**E2**	Songambele	152		
	**E3**	Mtakuja	82		
	**E4**	Nyamyeye A	95		
	**E5**	Nyamyeye B	164		
		**TOTAL**	**622**		
		**GRAND TOTAL**	**2965**		
**Mop-up**				**NA**	**28th Sept**
**Travel to Katoro**				**22nd Sept**	**29th Sept**

MkV1 Further Survey: Back-up Census Questionnaire

Section A - Household		
1.01	Date of interview	/ /200
1.02	Ward *(record ward code number)*	ward name: _
1.03	Village *(record village code number)*	village name: _
1.04	Kitongoji *(record village code number)*	kitongoji name: _
1.05	Census Interviewer's staff code	
1.06	Household number	
1.07	GPS- LATITUDE	.
1.08	GPS- LONGITUDE	.
1.09	Name(s) of Household Head	_ _
1.10	Sex of head of household	*(circle one)* Male 1<br>Female 2
1.11	Has the respondent signed the consent form?<br><br>If NO or not able to give informed consent then not eligible to continue. Thank and end interview	*(circle one)* Yes 1<br>No 2<br>No, refused/ not able to give informed consent 3
1.12	Are there any young people aged 15-30 years living in this household?<br>If yes how many?<br>If no then enter '00' and proceed to next household	
IF 1 OR MORE THEN COMPLETE SECTION B FOR EACH YOUNG PERSON AGED 15-30 YRS		

Section B – Young Person

1.13	Name(s) of Young Person	- -	
1.14	Sex of young person *(do not ask young person)*	*(circle one)* Male<br>Female	1<br>2
1.17	Have you left primary school ?<br><br>If NO or 'Did not attend primary school' or NK then this young person is not eligible and proceed to next young person in the household.	*(circle one)* Yes<br>No<br>Did not attend primary school<br>NK	1<br>2<br>3<br>9
1.18	What was the highest standard that you reached in primary school? *(circle one)*<br><br>If the young person did not reach std 5 in primary school then not eligible to continue- thank and proceed to next young person in household.<br><br>If don't know or can't remember then continue.	Std 1-4<br>Std 5<br>Std 6<br>Std 7<br>Don't know / don't remember	4 →*end interview*<br>5<br>6<br>7<br>9
1.19	What primary school(s) did you attend?<br>*Check name(s) of primary school(s) with list of primary schools in the community and enter school name and school code. 99 = school not on list .*<br><br>Name of school: ________________________________<br>Name of school: ________________________________<br><br>If did not attend eligible primary school then thank and end interview	School Code:<br>School Code:	__ __<br>__ __
1.20	What year did you leave primary school?<br>9999=NK<br><br>*If left in 1998 or earlier OR if left in 2005 or later then NOT ELIGIBLE. Please thank and proceed to next young person in HH*		

If left between 1999 and 2004 or 'not known' then ELIGIBLE
(1) WRITE CENSUS ID NUMBER ON QUESTIONNAIRE AND INVITATION

1.21	Census ID No.	⊗	⊗	⊗	⊗
		Ward (Q1.02) Village (Q1.03)	Kitongoji (Q1.04)	HH no. (Q1.06)	PDA no. (use no. of your PDA)

(2) WRITE DATE OF APPOINTMENT ON QUESTIONNAIRE AND INVITATION

1.22	Appointment date on Invitation:	/ /200

(3) PLEASE GIVE INVITATION TO ELIGIBLE YOUNG PERSON

MEMA kwa Vijana Trial Further Survey (2007-2008)

MkV1 Further Survey: Back-up Census Questionnaire

Sehemu A - Kaya		
1.01	Tarehe ya leo	/ /200
1.02	Kata *(andika jina na namba ya siri)*	Jina la kata: _
1.03	Kijiji *(andika jina na namba ya siri)*	Jina la kiji. _
1.04	Kitongoji *(andika jina)*	Jina la kitongoji : _
1.05	Namba ya muhojaji wa sensa	
1.06	Namba ya kaya (toka kwenye orodha ya mkuu wa kaya)	
1.07	GPS- LATITUDE (Kama haijulikani ingiza 99.99999)	.
1.08	GPS- LONGTUDE (kama haijulikani ingiza 999.99999)	.
1.09	Majina ya mkuu wa kaya<br>(Majina kamili 3)	_ _
1.10	Jinsia ya mkuu wa kaya	*(Zungushia moja)* Mme 1<br>Mke 2
1.11	Je, muhojiwa amesaini fomu ya kukubali kushiriki?*(Zungushia moja)*<br>Ndiyo 1<br>Hapana 2<br>Amekataa/ Hawezi kutoa ridhaa ya kukubali kushiriki 3<br>Kama HAPANA au hawezi kutoa ridhaa ya kukubali kushiriki hastahili kuendelea. Mshukuru na maliza mahojiano.	
1.12	Kuna jumla ya watu wangapi wanaoishi kwenye kaya hii?<br>Ingiza idadi	
1.13	Je kuna kijana yeyote mwenye umri wa miaka 15-30 katika kaya hii?<br>Kama jibu ni NDIYO, Ingiza idadi<br>Kama hapana basi ingiza '00' na uendelee katika kaya nyingine	
Kama ni mtu mmoja "1" AU ZAIDI BASI JAZA SEHEMU B KWA KILA KIJANA MWENYE UMRI WA MIKA 15-30 YRS		

Sehemu B – Kijana

	Jina la kitongoji:	- -		
	Majina ya mkuu wa kayaa (majina kamili 3)	- -		
1.14	Majina ya kijana (majina kamili 3)	- -		
1.15	Jinsia ya kijana *(usimwuulize kijna)*	*(zungushia moja)*	Mme<br>Mke	1<br>2
1.16	Umeshamaliza elimu ya shule ya msingi ?<br>Kama HAPANA au hakwenda shule ya msingi' au haijulikani basi kijana huyu hastahili na endelea na kijana mwingine katika kaya.	*(zungushia moja)*	Ndiyo<br>Hapana<br>Sikwenda shule ya msingi<br>Haijulikani	1<br>2<br>3<br>9
1.17	Ni kiwango gani cha juu ulichofikia katika elimu ya msingi?<br>*(zungushia moja)*<br>Kama muhojiwa hakufikia darasa la 5 katika shule ya msingi basi hahusiki kuendelea-mshukuru na endelea na kijana mwingine katika kaya.<br>Kama hajui au hawezi kukumbuka basi endelea.		Darasa 1-4<br>Darasa 5<br>Darasa 6<br>Darasa 7<br>Sijui / Sikumbuki	4 →maliza mahojiano kwa kijana huyu<br>5<br>6<br>7<br>9
1.18	Ni shule gani ya msingi (s) uliyosoma?<br>*Angalia jina(s)la shule ya msingi(s) katika orodha ya shule za msingi katika jamii na ingiza jina la shule na namba ya siri ya shule. 99 = Shule haipo katika orodha .*<br>Jina la shule: ____________________<br>Jina la shule l: ____________________<br>Kama hakusoa katika shuleya msingi husika basi mshukuru na maliza mahojiano		Code ya shule:<br>Code ya shule:	__ __<br>__ __
1.19	Ni mwaka gani ulihitimu elimu ya msingi?<br>*Kama alimaliza katika mwaka 1998 au mapema AU kama alimaliza katika mwaka 2005 au baada basi HAHUSIKI Tafadhali mshukuru na uendelee na kijana mwingine katika kaya*<br>Jaza 9999=NK (kama mwaka haufahamiki)			

Kama alihitimu kati ya mwaka 1999 na 2004 au 'haijulikani' basi ANAHUSIKA
(1) ANDIKA NAMBA YA UTAMBULISHO YA SENSA JUU YA DODOSO NA BARUA YA MWALIKO

1.20	Je,mtu huyu anahusika?	1.Ndiyo<br>2.Hapana → maliza mahojiano kwa kijana huyu	
	Namba ya kitambulisho ya sensa.<br>*Tumia namba zinazofuata katika dodoso hii kutoka kwenye orodha ya namba zasiri za sensa*		⊗ ⊗

(2) ANDIKA TAREHE YA AHADI (APPOINTMENT) JUU YA DODOSO NA KWENYE BARUA YA MWALIKO

1.21	Tarehe ya ahadi juu ya barua ya mwaliko:	/ /200

(3) TAFADHALI TOA BARUA YA MWALIKO KWA KIJANA ANAYESITAHILI

MEMA kwa Vijana Trial Further Survey (2007-2008)

Sehemu B – Additional Kijana (unganisha karatasi hii na dodoso kuu ya sensa)

	Jina la kitongoji:	- -	
	Majina ya mkuu wa kayaa (majina kamili 3)	- -	
1.14	Majina ya kijana (majina kamili 3)	- -	
1.15	Jinsia ya kijana *(usimwuulize kijna)*	*(zungushia moja)*	Mme 1<br>Mke 2
1.16	Umeshamaliza elimu ya shule ya msingi ?<br>Kama HAPANA au hakwenda shule ya msingi' au haijulikani basi kijana huyu hastahili na endelea na kijana mwingine katika kaya.	*(zungushia moja)*	Ndiyo 1<br>Hapana 2<br>Sikwenda shule ya msingi 3<br>Haijulikani 9
1.17	Ni kiwango gani cha juu ulichofikia katika elimu ya msingi?<br>*(zungushia moja)*<br>Kama muhojiwa hakufikia darasa la 5 katika shule ya msingi basi hahusiki kuendelea-mshukuru na endelea na kijana mwingine katika kaya.<br>Kama hajui au hawezi kukumbuka basi endelea.		Darasa 1-4 4 →maliza mahojiano kwa kijana huyu<br>Darasa 5 5<br>Darasa 6 6<br>Darasa 7 7<br>Sijui / Sikumbuki 9
1.18	Ni shule gani ya msingi (s) uliyosoma?<br>*Angalia jina(s)la shule ya msingi(s) katika orodha ya shule za msingi katika jamii na ingiza jina la shule na namba ya siri ya shule. 99 = Shule haipo katika orodha .*<br>Jina la shule: ___________________________<br>Jina la shule I: ___________________________<br>Kama hakusoa katika shuleya msingi husika basi mshukuru na maliza mahojiano		Code ya shule: ___ ___<br>Code ya shule: ___ ___
1.19	Ni mwaka gani ulihitimu elimu ya msingi?<br>*Kama alimaliza katika mwaka 1998 au mapema AU kama alimaliza katika mwaka 2005 au baada basi HAHUSIKI Tafadhali mshukuru na uendelee na kijana mwingine katika kaya*<br>Jaza 9999=NK (kama mwaka haufahamiki)		
	**Kama alihitimu kati ya mwaka 1999 na 2004 au 'haijulikani' basi ANAHUSIKA (1) ANDIKA NAMBA YA UTAMBULISHO YA SENSA JUU YA DODOSO NA BARUA YA MWALIKO**		
1.20	Je,mtu huyu anahusika?<br>Namba ya kitambulisho ya sensa.<br>*Tumia namba zinazofuata katika dodoso hii kutoka kwenye orodha ya namba zasiri za sensa*	1.Ndiyo<br>2.Hapana → maliza mahojiano kwa kijana huyu	⊗ ⊗
	**(2) ANDIKA TAREHE YA AHADI (APPOINTMENT) JUU YA DODOSO NA KWENYE BARUA YA MWALIKO**		
1.21	Tarehe ya ahadi juu ya barua ya mwaliko:		/ /200
	**(3) TAFADHALI TOA BARUA YA MWALIKO KWA KIJANA ANAYESITAHILI**		

Sticker_numbera

Place Stiker Here	
If Eligible:	*FS_ _ _ _ _ _ _*
Non Eligible:	*XFS_ _ _ _ _ _*

SECTION A: 1. Respondent's ID (completed by REGISTRATION INTERVIEWER)

01.01	Date of interview		/ /200<br>**q0101_day q0101_month q0101_year**
01.02	Ward *(record ward code number)*	ward name: - - - - - - - - **q0102** - - - - - - - - - - -- - - - - - - -	
01.03	Village *(record village code number)*	village name: - - - - - - - --**q0103** - - - - - - - - - - - - -- - - - -	
01.04	Registration Interviewer's staff code	**q0104**	
01.05	Sex of respondent *(do not ask respondent)*	*(circle one)*<br>**q0105**	Male 1<br>Female 2
01.06	Do you have MkV1 FS Invitation letter with you?	*(circle one)*<br>**q0106**	Yes 1<br>No, but received 2<br>Never received 3
01.07	Identification: name of attendee on list A?<br>*Find name of attendee on census list (list A).*	*(circle one)*<br>**q0107**	Yes 1<br>No 2
01.08	Census ID No.<br>*If invitation is produced or name on census list then enter census id number*<br>*If no invitation and not on census list then enter 88 88 88 88*		⊗ ⊗<br>**q0108a q0108b q0108c**
01.09	What is your date of birth?<br>99/99/1999=NK<br><br>If <u>full</u> date of birth NOT KNOWN then ask<br><br>How old are you?<br><br>Use calendar of events to assist young person to remember his/her age in completed years		/ /19<br>If full date of birth known → 01.10<br>**q0109a1 q0109a2 q0109a3**<br><br>age in years<br>99= Not known<br>**q0109b**

01.10	Proof of date of birth (Circle only one option)	q0110	Birth certificate 1<br>Clinic/ MCH card 2<br>Baptismal cert 3<br>Health survey ID card 4<br>School leaving card 5<br>Voters ID 6<br>Other documentation 7<br>No documentation 8
01.11	What was the highest standard that you completed in primary school? *(circle one)*<br><br>If attendee did not reach std 5 in primary school then not eligible to continue- thank and end interview.<br><br>If don't know or can't remember then continue.	q0111	Std 1-4 4 →*end interview*<br>Std 5 5<br>Std 6 6<br>Std 7 7<br>Did not attend primary school 8 →*end interview*<br>Don't know / don't remember 9
01.12	What primary school(s) did you attend?<br>*Check name(s) of primary school(s) with list of primary schools in the community and enter school name and school code. 999 = school not on list .*<br><br>Name of school: ____________________<br>Name of school: ____________________<br>Name of school: ____________________<br><br>If did not attend eligible primary school then thank and end interview	q0112a<br>q0112b<br>q0112c	School Code:<br>School Code:<br>School Code:

01.13 Which primary school and standard were you attending in (…year…)?

Ask first about 2004 then 2003 etc

If not in primary school then enter school code=888 and standard=8.

School codes
enter school code from 01.12 above
(888=not in primary school)
(999=school not on list)

Standard codes
enter standard code
e.g. std 1 = '1'
not in school = '8'

Year	School code	Standard
2004	q0113a1	q0113a2
2003	q0113b1	q0113b2
2002	q0113c1	q0113c2
2001	q0113d1	q0113d2
2000	q0113e1	q0113e2
1999	q0113f1	q0113f2
1998	q0113g1	q0113g2

01.14	Have you previously taken part in a health survey where specimens (urine and/or blood) were collected?<br><br>If no then proceed to q01.18	q0114	(circle one)	Yes 1<br>No 2 →01.18
01.15	Which survey?<br><br>If did not take part in MkV1 then proceed to q01.18	q0115	(circle one)	MkV1 1<br>Other 2 →01.18
01.16	Ask if man/woman has MkV1 ID card.<br>• If card seen then indicate colour of card.<br>• If do not have MkV1 ID card then look on list of MkV1 participants to see if on list.<br><br>If 4=not on list then proceed to q01.18	q0116		MkV1 White card 1<br>MkV1 Blue card 2<br>Name on MkV1 list 3<br>Not on list 4 →01.18
01.17	MkV1 ID number<br>(*write number from ID card or MkV1 list*)	q0117a q0117b q0117c q0117d q0117e q0117f<br>⊗ / ⊗ / ⊗ / ⊗ ⊗ /		*This person is eligible. Please proceed to q01.19*
01.18	Look at Q 01.13 to see what year were they in Std 7 OR what year would they have been in Std 7 if had reached Std 7.<br><br>Take list D.<br>Find name of attendee<br>OR if left before std 7 must name 2 classmates who reached Std 7	q0118a<br><br>q0118b	Std 7 year	Std 7 list (list D) 1 → *eligible*<br>Std 7 list (list D) – classmates 2 → *eligible*<br>Neither of the above 3 → *NOT eligible*
01.19	ELIGIBLE?	q0119	*(circle one)*	Yes 1<br>No 2
	PLEASE ASSESS IF ELIGIBLE PERSON IS ABLE TO GIVE INFORMED CONSENT AND IF SO ASK THEM TO SIGN CONSENT FORM			
01.20	Has the participant signed the consent form?<br><br>If refuse or not able to give informed consent then not eligible to continue. Thank and end interview.	q0120	*(circle one)*	Yes 1<br>No- refused 2<br>Not able to give informed consent 3
01.21	If the laboratory tests in Mwanza show that you have a sexually transmitted infection (Syphlis, CT, NG) would you like us to return to treat you?	q0121	*(circle one)*	Yes 1<br>No 2

Sticker_numberb

Place Sticker here
FS_ _ _ _ _ _ _ _

SECTION B: 2. Demographic Information (completed by MAIN INTERVIEWER)

02.01	Main Interviewer's staff code q0201		
02.02	I am going to ask you some questions. This will take about half an hour. I can ask the questions in either Swahili or Sukuma, depending on which language you would prefer. Would you like me to ask the questions in Swahili or Sukuma?<br><br>*Say "Remember that I can also use the other language at any point if you think you will understand better. Just tell me."*	q0202<br>*(circle one)*	
		Swahili	1
		Sukuma	2
02.03	What is your tribe? *(circle only one)* q0203	Sukuma	1
		Jita	2
		Zinza	3
		Kara	4
		Kerewe	5
		Sumbwa	6
		Rongo	7
		Other	8
02.04	What is your religion? *(circle only one)* q0204	Catholic	1
		Other Christian	2
		Moslem	3
		Other religion (including traditional)	4
		No religion	5
02.05	What is the highest education level you have attained? *(circle only one)* q0205	Did not complete primary school	1
		Completed primary school	2
		Incomplete secondary	3
		Secondary school	4
		Advanced Secondary school	5
		Vocational training	6
		College (e.g. Teachers' Training College, Police college, etc)	7
		University	8
		Other studies (Islamic College: theological college)	9

02.06 What are the main kinds of work/activities (shughuli or kazi) you do or have done over the last 12 months?
Do Not prompt the respondent, but after each answer ask him/her to mention as many as possible

(TICK ALL THAT APPLY)

Mine employee professional (*engineer, accountant, geologist, surveyor etc*)	**q0206a**
Mine employee non professional (labourer, workman, watchman, driver etc)	**q0206b**
Professional (*eg teacher/nurse/accountant/police*)	**q0206c**
Other manual (*eg carpenter / tailor*)	**q0206d**
Business (e.g. duka owner, garage owner, Bar & Guest owner, commercial farming)	**q0206e**
Petty Trade (e.g. produce at market, or sell peanuts in evening, food vendor, alcohol brewer)	**q0206f**
Bar worker / guest house worker / hotel worker	**q0206g**
Truck Driver	**q0206h**
Turn boy	**q0206i**
Fisherman	**q0206j**
Farmer	**q0206k**
Housewife	**q0206l**
House girl / domestic worker	**q0206m**
At School/university	**q0206n**
None	**q0206o**
Other	**q0206p**

Other (specify: ____________ **q0206q [string]** ______________________________)

02.07 In the past 4 weeks, have you slept away from your ward? **q0207** If yes, → for how many night(s)
If no, put "00"

If yes, circle 1 to Q02.08 and ask how many times did you sleep at least 1 night away in the past 12 months?

02.08 In the past 12 months, have you sept away from your ward? **q0208a**
(circle one)

Yes	1
No	2
don't know	9

If NO or don't know*→skip to section 3*

If YES: How many times did you sleep at least 1 night away? **q0208b**

If YES: What was the total length of time you slept away in the past 12 months? **q0208c** N N N
Enter as respondent chooses to answer: Indicate unit (D,W,M) in the last box
e.g. '003M' = 3 months; '060D' = 60 days; '012W' = 12 weeks; '999X' = NK **q0208d**unit U

SECTION C: 3. SEXUAL and REPRODUCTIVE HEALTH KNOWLEDGE AND ATTITUDES				
** * * * Interviewer: Read the explanatory sheet now * * * **				(completed by MAIN INTERVIEWER)
I'm now going to ask you some questions about reproductive health knowledge, diseases and making love. Always when I mention the word making love I am talking about having penetrative sex with somebody. This will include sexual intercourse where one of the two has not agreed (one part forced). We know that some young people like you are already having sex and some are not. We are only interested in hearing the truth about young peoples' sexual experience even if you have not ever had sex yet. This discussion is very confidential between you and me, so I hope that you will be free to tell me about your life sexual experience.				
03.01	Can pus or abnormal fluids coming out of the private parts be caught by making love with someone?	**q0301**	*(Circle one)*	Yes 1<br>No 2<br>NK 9
03.02	Can schistosomiasis be caught by making love with someone?	**q0302**	*(Circle one)*	Yes 1<br>No 2<br>NK 9
03.03	Can an ulcer on the private parts be caught by making love with someone?	**q0303**	*(Circle one)*	Yes 1<br>No 2<br>NK 9
03.04	Can HIV be caught by making love with someone?	**q0304**	*(Circle one)*	Yes 1<br>No 2<br>NK 9
03.05	Can you catch HIV by sharing a plate of food with an HIV positive person?	**q0305**	*(Circle one)*	Yes 1<br>No 2<br>NK 9
3.06	Can a person who looks strong and healthy have HIV?	**q0306**	*(Circle one)*	Yes 1<br>No 2<br>NK 9
03.07	If a man wants to make love with a woman, can she refuse to make love with him if he is older than her?	**q0307**	*(Circle one)*	Yes 1<br>No 2<br>NK 9
03.08	If a man wants to make love with a woman, can she refuse to make love with him if he is her lover?	**q0308**	*(Circle one)*	Yes 1<br>No 2<br>NK 9
03.09	If a young woman accepts a gift from a man, must she agree to make love with him?	**q0309**	*(Circle one)*	Yes 1<br>No 2<br>NK 9
I'm now going to ask you some questions about pregnancy				
03.10	Is it possible for a girl to become pregnant the first time she makes love?		**q0310**<br>*(Circle one)*	Yes 1<br>No 2<br>NK 9

03.11	Is it possible for a person to prevent pregnancy by not making love at all?	q0311 (Circle one)	Yes 1<br>No 2<br>NK 9
03.12	Is it possible for a person to prevent pregnancy by using a condom while making love?	q0312 (Circle one)	Yes 1<br>No 2<br>NK 9
I'm now *going* to ask you questions about condoms			
03.13	Do you know what a condom is?<br>If answer is No → skip to q04.01	q0313 (Circle one)	Yes 1<br>No 2 →section 4
03.14	Has anyone ever shown you how to use a condom?	q0314 (Circle one)	Yes 1<br>No 2<br>NK 9
03.15	Do some male condoms have HIV in them before they are used?	q0315 (Circle one)	Yes 1<br>No 2<br>NK 9
03.16	Does using a male condom when making love prevent the man/woman being infected with HIV?	q0316 (Circle one)	Yes 1<br>No 2<br>NK 9
03.17	Where could you get condoms in your village if you needed them?<br>Do NOT prompt the respondent, but after each answer ask, “Thank you. Anywhere else?”<br>(TICK ALL THAT ARE MENTIONED)	Store / kiosk / pharmacy<br>Health facility<br>Non-governmental organisation representative<br>Other private person<br>Other (specify): ____________<br>Don’t know	q0317a<br>q0317b<br>q0317c<br>q0317d<br>q0317e<br>q0317f
03.18	Where could you get condoms <u>for free</u> in your village if you needed them?<br>Do NOT prompt the respondent, but after each answer ask, “Thank you. Anywhere else?”<br>(TICK ALL THAT ARE MENTIONED)	Store / kiosk / pharmacy<br>Health facility<br>Non-governmental organisation representative<br>Other private person<br>Other (specify): ______ q0318f (string)______<br>Don’t know	q0318a<br>q0318b<br>q0318c<br>q0318d<br>q0318e<br>q0318g

4. SEXUAL BEHAVIOUR

(completed by MAIN INTERVIEWER)

I'm now going to ask you some questions about making love. Always when I mention the word making love I am talking about having penetrative sex with somebody. This will include sexual intercourse where one of the two has not agreed (one part forced). We know that some young people like you are already having sex and some are not. It is not important whether you have or have not had sex, as we are only interested in hearing the truth about young peoples' sexual experience. This discussion is very confidential between you and me, so I hope that you will be free to tell me about your life sexual experience. Some of these questions are about long periods of months and years. It may be difficult to quickly recall experiences over such a long period of time. What is most important is that you answer such questions as fully and accurately as possible. So please, take as much time as you need to think about them when answering.

04.01	Have you ever made love?<br>If "No", Check by asking: "Have I understood you correctly, that you have never made love throughout your whole life?"<br>If still says that (s)he has never made love: Circle 2 = No	*(Circle one)*<br>**q0401**	Yes 1<br>No 2
	**If still says that (s)he has <u>never</u> made love, skip to section 8**		
04.02	Have you <u>ever</u> used a male condom?	**q0402**	Yes 1<br>No 2<br>NK 9
04.03	I now want you to think of the <u>first time</u> you made love with someone. How old were you when you first made love with someone?<br>(Please try to help respondent to remember the actual age at first sex, e.g which std were you in primary school etc.)<br>*Enter age at first sex* years **q0403a** *If exact age <u>not known</u> estimate (tick)*<br>12yrs or less 44 13-14yrs 55 15-17yrs 66 18yrs or more 77 NK 99 **q0403b**		
04.04	I now want you to think of the first person that you made love with.<br>Was that person older, younger, or the same age as you?<br>*If 'same age' or 'not known' then skip to q04.06*	**q0404**	Older 1<br>Younger 2<br>Same age 3 →q 04.06<br>Not known 9 →q 04.06
04.05	How many years older/younger?<br>*If not known estimate (tick)*<br>1yr or less 44 2-4yrs 55 5-9yrs 66 10-14yrs 77 15yrs or more 88 NK 99 **q0405a**		*Enter Years*<br>**q0405**
04.06	I now want you to think of all the people you have made love with in the past <u>four weeks</u>.<br>How many people have you made love with in the past four weeks?	**q0406**	Enter number (two digits)
04.07	I would now like you to think about where you were a <u>year ago</u> at this time.<br>That would be xx month (mention the current month) last year till today, and / or xx season eg.raining season, harvesting season etc last year till today. Do you remember?<br>Can you now recall how many people you made love with since that time?<br>Again please take your time as you think about this.<br>NB Check that this number include partners in the last 4 weeks.		Enter number (two digits)<br>*unknown = 99*<br>**q0407**

04.08	How many of these partners were new to you?<br>(ie made love for the first time in the last 12 months)	q0408	Enter number (two digits)<br>*not applicable = 88*<br>*unknown = 99*

If says that (s)he has not made love in past 12 months, skip to section 6

5. SEXUAL PARTNERS IN LAST 12 MONTHS

(completed by MAIN INTERVIEWER)

** * * * Fill in one column for each partner, starting with the most recent * * * **

			Most recent	2nd most recent	3rd most recent
05.01	Think about the last / second last / third last person that you made love with in the past twelve months. These do not need to be new partners.<br><br>Do you have them in mind?<br>*NO:*<br>*Try to make him / her recall by specifying the period e.g. since last Christmas*<br><br>Do you have them in mind?<br>*YES - continue*<br><br>Was this your wife/husband, another regular partner, a casual partner, or a CSW?		q0501a	q0501b	q0501c
		Spouse	1	1	1
	(circle one for each partner)	Other regular partner	2	2	2
		Casual partner	3	3	3
		Commercial Sex Worker	4	4	4
05.02	Do you live with this person?	Yes	q0502a 1	q0502b 1	q0502c 1
	(circle one for each partner)	No	2	2	2
05.03	Is this person older, younger or the same age as you?	Older	q0503a 1	q0503b 1	q0503c 1
	(circle one for each partner)	Younger	2	2	2
		Same age→Q05.05	3	3	3
		Don't know→Q05.05	9	9	9
05.04	How many years older/younger?<br>enter exact years, if not known enter 99		q0504a N N	q0504b N N	q0504c N N
05.05	Did this person go to primary school in this ward?	Yes	1	1	1
		No	q0505a 2	q0505b 2	q0505c 2
		Don't know	9	9	9
05.06	What was the highest standard that this person reached in primary school?<br>*(circle one for each partner)*		q0506a	q0506b	q0506c
		Std 1-4	4	4	4
		Std 5	5	5	5
		Std 6	6	6	6
		Std 7	7	7	7
		Did not attend primary school	8	8	8
		Don't know / don't remember	9	9	9

No.	Question	Partner 1	Partner 2	Partner 3
05.07	What are the main kinds of work/activities (shughuli or kazi) this partner does / has done over the last 12 months?<br>*Do Not prompt the respondent, but after each answer ask him/her to mention as many as possible*<br>*(for each partner tick ALL that apply)*			
	Mine employee professional (*engineer, accountant, geologist, surveyor etc*)	q0507a01	q0507b01	q0507c01
	Mine employee non professional (labourer, workman, watchman, driver etc)	q0507a02	q0507b02	q0507c02
	Professional (eg teacher / nurse / accountant / police)	q0507a03	q0507b03	q0507c03
	Other manual (eg carpenter / tailor)	q0507a04	q0507b04	q0507c04
	Business (e.g. duka owner, garage owner, Bar & Guest owner, commercial farming)	q0507a05	q0507b05	q0507c05
	Petty Trade (e.g. produce at market, sell peanuts in evening, food vendor, alcohol brewer)	q0507a06	q0507b06	q0507c06
	Bar worker / guest house worker / hotel worker	q0507a07	q0507b07	q0507c07
	Truck driver	q0507a08	q0507b08	q0507c08
	Turn boy	q0507a09	q0507b09	q0507c09
	Fisherman	q0507a10	q0507b10	q0507c10
	Farmer	q0507a11	q0507b11	q0507c11
	Housewife	q0507a12	q0507b12	q0507c12
	House girl/domestic worker	q0507a13	q0507b13	q0507c13
	At School/ University	q0507a14	q0507b14	q0507c14
	None	q0507a15	q0507b15	q0507c15
	Don't know	q0507a16	q0507b16	q0507c16
	Other (specify): partner 1: ______<br>partner 2: ______<br>partner 3: ______	q0507a17	q0507b17	q0507c17
05.08	How long ago was the <u>first time</u> you made love with this person?<br>Enter as respondent chooses to answer: Indicate unit (D,W,M, Y) in the last box<br>e.g. '002Y' = 2 years ago; '003M' = 3 months ago; '999X' = DK	q0508a1 N N N<br>unit U q0508a2	q0508b1 N N N<br>unit U q0508b2	q0508c1 N N N<br>unit U q0508c2
05.09	How long ago was the <u>last time</u> you made love with this person?<br>Enter as respondent chooses to answer: Indicate unit (D,W,M) in the last box<br>e.g.'003M' = 3 months ago; '999X' = NK	q0509a1 N N N<br>unit U q0509a2	q0509b1 N N N<br>unit U q0509b2	q0509c1 N N N<br>unit U q0509c2
05.10	How many times did you make love with this person in the last four weeks?<br>(Enter number- two digit) OR 99=NK	N N<br>q0510a	N N<br>q0510b	N N<br>q0510c
05.11	What is your current sexual relationship status with this person?	q0511a	q0511b	q0511c
	We are still in a relationship and will make love again	1	1	1
	Our relationship is not continuing but we might make love again	2	2	2
	Our relationship has completely ended	3	3	3
	Don't know	9	9	9

05.12	Did you use any family planning method to prevent pregnancy the last time you made love with this partner? *(Circle one)*	q0511a0		q0511b0		q0511c0
	Yes	1		1		1
	No	2		2		2
	If YES: What did you use to prevent pregnancy? *(tick ALL that apply)*					
	Condom	q0512a1		q0512b1		q0512c1
	Oral contraceptive pills	q0512a2		q0512b2		q0512c2
	Injectable contraceptives	q0512a3		q0512b3		q0512c3
	Rhythm method	q0512a4		q0512b4		q0512c4
	Traditional medicines	q0512a5		q0512b5		q0512c5
	Withdrawal method	q0512a6		q0512b6		q0512c6
	Douching after sex	q0512a7		q0512b7		q0512c7
	Don't know	q0512a8		q0512b8		q0512c8
	Other (specify): partner 1: ____________	q0512a9		q0512b9		q0512c9
	partner 2: ____________					
	partner 3: ____________					
05.13	Did you use a condom the last time you made love with this person? *If yes* Did you use the condom throughout making love this last time? *Using a condom throughout making love means start AND end making love using condom.*	q0513a		q0513b		q0513c
	(circle one for each partner) Yes, throughout making love→q05.15	1		1		1
	Yes, but not throughout→q05.14	2		2		2
	No→q05.14	3		3		3
	If answer in 05.13 is NO, DID NOT USE A CONDOM or YES, BUT NOT THROUGHTOUT ask q05.14 below. Otherwise skip to q05.15.					
05.14	Why didn't you and your partner use a condom that time? or Why didn't you and your partner use a condom throughout making love that time?					
	(tick ALL that apply)					
	Not available/ Don't know where to get them	q0514a01		q0514b01		q0514c01
	Too expensive	q0514a02		q0514b02		q0514c02
	Do NOT prompt the respondent, — Partner objected	q0514a03		q0514b03		q0514c03
	but after each answer ask, — Don't like them	q0514a04		q0514b04		q0514c04
	"Thank you. Anything else?" — Don't know how to use them	q0514a05		q0514b05		q0514c05
	Morally wrong / against religion	q0514a06		q0514b06		q0514c06
	Used other contraceptive	q0514a07		q0514b07		q0514c07
	Don't have many partners	q0514a08		q0514b08		q0514c08
	Trust partner	q0514a09		q0514b09		q0514c09
	Didn't think of it / forgot	q0514a10		q0514b10		q0514c10
	Did not want to prevent pregnancy	q0514a11		q0514b11		q0514c11
	Condom broke	q0514a12		q0514b12		q0514c12
	Don't know	q0514a13		q0514b13		q0514c13
	Other (specify): partner 1: ____________	q0514a14		q0514b14		q0514c14
	partner 2: ____________					

	partner 3: ______________________					
05.15	Has there been an occasion when you did not want to make love with this partner?	q0515a0		q0515b0		q0515c0
	Yes	1		1		1
	No	2		2		2
	Don't remember	9		9		9
	If YES: What did you do the <u>last time</u> that this happened?	q0515a1		q0515b1		q0515c1
	Refused and did not have sex	1		1		1
	Refused but still had sex	2		2		2
	Did not refuse	3		3		3
	Don't remember	9		9		9

6. CONTRACEPTION

(completed by MAIN INTERVIEWER)

06.01 Throughout your whole life up to now, how many different men / women have you made love with? (Including current partners, past partners, spouse and all other kinds of partner) Again please take as much time as you need to remember this accurately.

q0601

Enter number (two digits)

If participant cannot remember the exact number please ask them to give an approximate number

06.02 – 06.04 FEMALES ONLY (skip to q06.05 for MALES)

06.02 < Females > How many times have you been pregnant?

q0602

Enter number (two digits)
not known = 99

06.03 <Females> In what year in school did you first get pregnant?

q0603

Not in school / left school	2
Std 3	3
Std 4	4
Std 5	5
Std 6	6
Std 7	7
Not applicable	8
Don't know / don't remember	9

06.04 < Females > Sometimes a girl or young woman becomes pregnant when she does not plan to (not a good time to become pregnant).

Have you ever become pregnant when you did not plan to (when it was not a good time)?

Yes	1
No	2
NK	9

q0604

06.05 – 06.07 MALES ONLY (skip to q06.08 for FEMALES)

06.05 < Males > How many times have you made girls pregnant?

If the answer is NO, enter 00 then→go to 06.08

q0605

Enter number (two digits)
not known = 99

06.06 <Males> In what year in school did you first make a girl pregnant?

q0606

Not in school / left school	2
Std 3	3
Std 4	4
Std 5	5
Std 6	6
Std 7	7
Not applicable	8
Don't know / don't remember	9

06.07 < Males > Sometimes a girl or young woman becomes pregnant when she does not plan to (not a good time to become pregnant).

Have you ever got a girl pregnant when you did not plan to (when it was not a good time)?

Yes	1
No	2
NK	9

q0607

06.08	Did you ever use any contraceptive methods to prevent pregnancy while you were making love?	q0608	Yes<br>No<br>NK	1<br>2→q 07.01<br>9→q 07.01
	If YES: Which contraceptive methods did you ever use to prevent pregnancy<br><br>Do not prompt the respondent, but after each answer ask "Thank you. Anything else?"<br><br>(TICK ALL THAT ARE MENTIONED)	q0608a1	Condom	
		q0608b1	Oral contraceptive pills	
		q0608c1	Injectable contraceptives	
		q0608d1	Rhythm method	
		q0608e1	Traditional medicines	
		q0608f1	Withdrawal method	
		q0608g1	Douching after sex	
		q0608h1	Other (specify): ____________	

7. MARRIAGE

(completed by MAIN INTERVIEWER)

I'm now going to ask you some questions about marriage experience "marital status". Always when I mention the word marriage I am talking about living with somebody as wife/husband. This will include unofficial marriage (in Swahili "Kimada/ Nyumba ndogo") or living with somebody as wife/husband without any initiation ceremony. We know that some young people like you have been married once, others have been married more than once and some have never been married. We are only interested in hearing the truth about young peoples' marriage experiences. This discussion is very confidential between you and me, so I hope that you will be free to tell me about your life marriage experience.

07.01 In total, how many times have you been married OR lived with man / woman as married?

Enter '00' if never married or lived as married and skip to question 07.08

Enter number (two digits)
not known = 99
q0701

07.02 How old were you when you first married OR lived with a man / woman as married?

Enter age at first marriage years **q0702** *If exact age __not known__ estimate (tick)*

12yrs or less 44 13-14 55 15-17yrs 66 18yrs or more 77 NK 99 **q0702a**

07.03 <Males> Are you currently married or living with woman as married? *(Circle one)* **q0703**
Yes 1
No 2 → q07.08

07.04 <Females> Are you currently married or living with man as married? *(Circle one)* **q0704**
Yes 1
No 2 → q07.08

07.05 <Males> How many wives currently do you stay/live with? **q0705**

Enter number (two digits)
not known = 99

07.06 <Females> How many wives, including you, does your husband have now? **q0706**

Enter number (two digits)
not known = 99

07.07 Do you live with (any of) your spouse(s)? *(Circle one)* **q0707**
Yes 1 → q08.01
No 2

07.08 Do you live with a lover(s)? *(Circle one)* **q0708**
Yes 1
No 2

8. EXPERIENCE OF SEXUALLY TRANSMITTED DISEASES (completed by MAIN INTERVIEWER)

08.01 Have you ever had pus or abnormal fluids coming out of your private parts (vagina/penis) in the past 12 months? **q0801**

(Circle one)

Yes	1
No	2
Don't know	9

08.02 Have you had ulcers or blisters on your private parts in the past 12 months? **q0802**

(Circle one)

Yes	1
No	2
Don't know	9

If both 08.01 and 08.02 are answered No or Don't know, skip to question 08.06

08.03 Did you do any of the following the last time (i.e. the most recent time) you had a genital ulcer/blister or pus/abnormal fluids coming out of your private parts?

READ ANSWER OPTIONS ALOUD, then tick all that apply .

**q0803a**	Self- treatment (traditional medicine at home)	
**q0803b**	Self- treatment (western / modern medicine at home)	
**q0803c**	Sought advice / medicine from a government hospital or health facility	→q08.05
**q0803d**	Sought advice / medicine from a private hospital or health facility	→q08.05
**q0803e**	Sought advice / medicine from a traditional healer	
**q0803f**	None of the above	

08.04 What are the reasons, why you did not go to a hospital/health facility?

Do not prompt the respondent, but after each answer ask "Thank you. Anything else?"

(TICK ALL THAT ARE MENTIONED)

**q0804a**	Unfamiliarity with services	→08.06
**q0804b**	Too far	→ 08.06
**q0804c**	High cost	→ 08.06
**q0804d**	Poor treatment	→ 08.06
**q0804e**	Lack of confidentiality	→ 08.06
**q0804f**	Unfriendly staff	→ 08.06
**q0804g**	Embarrassment/Shyness	→ 08.06
**q0804h**	Other (specify): ___**q0804i (string)** _	→ 08.06

8.05 How long after first experiencing symptoms did you go to a hospital [health facility]? **q0805**

(Circle one)

Immediately (1 to 2 days)	1
3 to 6 days	2
One week and above	3
Not applicable	8
Don't know	9

8.06	Have you had a blood transfusion in the last 5 years?	(Circle one) q0806	Yes<br>No<br>Don't know	1<br>2<br>9
8.07	How many injections have you had in the last 12 months?	q0807	Enter number (two digits)<br>*not known = 99*	
8.08	We are aware that some men / women in this area are circumcised. Are you circumcised?<br>*If answer is 2 or 9 skip to section 9*	(Circle one) q0808	Yes<br>No<br>Don't know	1<br>2 → q09.01<br>9 → q09.01
8.09	If yes, how old were you when this was done?	q0809	years<br>Enter age (two digits)<br>*not known = 99*	

9. SCHOOL LESSONS & MEMA KWA VIJANA INTERVENTION (completed by MAIN INTERVIEWER)

I'm now going to ask you questions about health and some diseases

09.01	Did you ever attend a MkV session when you were in primary school?	(Circle one) q0901	Yes<br>No<br>Don't know	1<br>2<br>9
09.02	Did you go with your primary school class to visit a hospital/health facility/ dispensary?	(Circle one) q0902	Yes<br>No<br>Don't know	1<br>2<br>9
09.03	Did a health care worker ever visit your class in primary school?	(Circle one) q0903	Yes<br>No<br>Don't know	1<br>2<br>9
09.04	Did any of your primary teachers ever talk to your class about resisting pressure to make love?	(Circle one) q0904	Yes<br>No<br>Don't know	1<br>2<br>9
09.05	Did any of your primary teachers ever talk to your class about using condoms to avoid HIV?	(Circle one) q0905	Yes<br>No<br>Don't know	1<br>2<br>9
09.06	Were you a MEMA kwa Vijana CPE (Class Peer Educator)?	(Circle one) q0906	Yes<br>No<br>Don't know	1<br>2<br>9

All respondents should be directed to the Laboratory

10. LAB SPECIMENS (completed by LAB WORKERS)

10.01	Lab workers' staff codes	q1001a, q1001b	
10.02	When did you last urinate?	(Swahili time) q1002a : q1002b	Morning Afternoon Evening (circle one) q1002c
10.03	Time urine container given to respondent<br>Leave as is for now, to review after pilot study	(Swahili time) q1003a : q1003b	Morning Afternoon Evening (circle one) q1003c
10.04	Number of serum aliquots taken<br>If <3, specify reason:	q1004<br>______________________________________	
10.05	Number of urine aliquots taken *[take 2 samples]*	q1005	If 2 samples taken → q. 10.07
10.06	Why were < 2 urine aliquots taken?	q1006<br>*(Circle one)*	No urine 1<br>Insufficient urine 2<br>Refused 3<br>Other (specify): __________________ 4
10.07	RBC result	*(Circle one)*<br>q1007	negative 0<br>+ 1<br>+ + 2<br>+ + + 3<br>+ + + + 4

11. CLINICAL (completed by CLINICIANS)

Sticker_numberc

Place Stiker Here	
If Eligible:	*FS_ _ _ _ _ _ _*
If not Eligible:	*XFS_ _ _ _ _ _*

11.01 Clinician's staff code q1101		
< Males > skip to q 11.04		
11.02 < Females> Are you pregnant? *(Circle one)* q1102	Yes 1<br>No 2<br>don't know 9	
< Females > Only examine genitals if report symptoms of ulcers (GUS)<br>< Males > Examine genitals of all males.		
	SYMPTOMS	EXAM
11.03 Do you have either pus or an abnormal discharge coming from your private parts (vagina or penis) at the moment (GDS)?	q1103a<br>Yes 1<br>No 2<br>NK 9	q1103b<br>Yes, seen 1<br>No, not seen 2<br>Refused exam 7<br>Not examined 8
11.04 *Type of discharge (Women only)*	Curdlike 1<br>Non-curdlike 2<br>Not applicable 8	q1104
11.05 Do you have an ulcer or sore or blister on your private parts at the moment (GUS)?	Yes 1<br>No 2<br>NK 9<br>q1105a	Yes, seen 1<br>No, not seen 2<br>Refused exam 7<br>Not examined 8<br>q1105b
11.06 Do you have any genital itching at the moment?	Yes 1<br>No 2<br>NK 9	q1106
11.07 Do you have any pain during micturation/urination?	Yes 1<br>No 2<br>NK 9	q1107

11.08	< Males > skip to question 11.09<br><br>< Females > Do you have any lower abdominal / pelvic pain at the moment or pain in your abdomen when you have sex?	Yes 1<br>No 2<br>NK 9	q1108
11.09	Do you have any genital warts at the moment?	Yes 1<br>No 2<br>NK 9<br>q1109a	Yes, seen 1<br>No, not seen 2<br>Refused exam 7<br>Not examined 8<br>q1109b
11.10	Do you have any lumps in the groin at the moment?	Yes 1<br>No 2<br>NK 9<br>q1110a	Yes, seen 1<br>No, not seen 2<br>Refused exam 7<br>Not examined 8<br>q1110b
11.11	< Females > skip to question 11.12<br><br>< Males > Examine for circumcision	q1111	Circumcised 1<br>Not circumcised 2<br>Refused exam 7<br>Not examined 8

11.12	DIAGNOSES			
	diagnosed	treated	referred	refused
None	q1112a			
GUS (men or women)	q1112b1	q1112b2	q1112b3	q1112b4
Genital blisters or vescicles	q1112c1	q1112c2	q1112c3	q1112c4
VDS without curd-like discharge (women only)	q1112d1	q1112d2	q1112d3	q1112d4
VDS with curd-like discharge (women only)	q1112e1	q1112e2	q1112e3	q1112e4

	Tick all that apply	PID (women only)	q1112f1	q1112f2	q1112f3	q1112f4
		Urethral discharge (men only)	q1112g1	q1112g2	q1112g3	q1112g4
		Epididymo-orchitis (men only)	q1112h1	q1112h2	q1112h3	q1112h4
		Bubo	q1112i1	q1112i2	q1112i3	q1112i4
		Pubic lice	q1112j1	q1112j2	q1112j3	q1112j4
		Scabies	q1112k1	q1112k2	q1112k3	q1112k4
		Warts	q1112l1	q1112l2	q1112l3	q1112l4
		Signs of secondary syphilis	q1112m1	q1112m2	q1112m3	q1112m4
		Symptoms of malaria	q1112n1	q1112n2	q1112n3	q1112n4
		Schistosomiasis	q1112o1	q1112o2	q1112o3	q1112o4
		Cough	q1112p1	q1112p2	q1112p3	q1112p4
		Headache	q1112q1	q1112q2	q1112q3	q1112q4
		Other (specify): ____________________	q1112r1	q1112r2	q1112r3	q1112r4
		__________ q1112s (string)____________________				

	<MALE> If participant needs treatment then complete Q11.16, if no treatment needed then end interview<br><FEMALE> If needs treatment for GUS, VDS, PID, Bubo or Schistosomiasis then please ask Q.11.13<br>If treatment not needed for these conditions then you may proceed to Q11.16 and provide other treatment.
11.13	Is this participant visibly pregnant? q1113 — Yes 1 → q11.16; No 2 → q11.14
11.14	When was your last menstrual period?<br>(99 / 99 / 2009 if unknown)<br>IF MORE THAN 1 MONTH SINCE LMP THEN TEST FOR PREGNANCY — / /200 q1114a q1114b q1114c
11.15	Result of Pregnancy Test (Circle one only)
	q1115 Positive 1 Negative 2 Refused 3 Not Done / Not applicable 8

11.16	TREATMENT GIVEN		*tick if given*
**GUS**			
GUS 1st line treatment	Benz. Penicillin 2.4 MU, im stat ½ in each buttock		q1116a1a
	Cotrimoxazole 400/80 mg, 5 tabs bid for 3 days		q1116a1b
GUS 1st line treatment if allergic to penicillin	Erythromycin 500mg qds for 7 days		q1116a2a
	Cotrimoxazole 400/80 mg, 5 tabs bid for 3 days		q1116a2b
GUS 1st line treatment if pregnant	Benz. Penicillin 2.4 MU, im stat ½ in each buttock		q1116a3a
	Erythromycin 500mg qds for 15 days		q1116a3b
GUS 2nd line treatment	Ceftriaxone 250 mg im, stat		q1116a4
**VDS**			
VDS (No curdlike discharge) 1st line treatment	Ciprofloxacin 500 mg, stat		q1116b1a
	Doxycycline 100 mg, bid for 7 days		q1116b1b
	Metronidazole 2 g, stat *warn about alcohol*		q1116b1c
VDS (no curdlike discharge) 1st line if in 1st trimester of pregnancy	Erythromycin 500mg qds for 7 days		q1116b2a
	OR Ceftriaxone 250 mg im, stat		q1116b2b
VDS (no curdlike discharge) 1st line if in 2nd or 3rd trimester of pregnancy	Erythromycin 500mg qds for 7 days		q1116b3a
	OR Ceftriaxone 250 mg im, stat		q1116b3b
	AND Metronidazole 2 g, stat (i.e. give with either erythomycin or cetriaxone) *warn about alcohol*		q1116b3c
VDS 2nd line treatment	Clotrimazole pessaries 100 mg, OD, PV		q1116b4a
	Ceftriaxone 250 mg im, stat		q1116b4b
	Doxycycline 100 mg, bid for 7 days		q1116b4c
	Metronidazole 2 g, stat *warn about alcohol*		q1116b4d
VDS 1st line if complains of curdlike discharge (add to standard 1st line treatment for VDS)	With vulval itching	Clotrimazole pessaries 100 mg, OD, PV	q1116b5a1
		Clotrimazole cream, 1 tube	q1116b5a2
	No vulval itching	Clotrimazole pessaries 100 mg, OD, PV	q1116b5b

**PID**		
PID 1st line treatment	Ciprofloxacin 500 mg, stat	q1116c1a
	Doxycycline 100 mg, bid for 14 days	q1116c1b
	Metronidazole 400mg bid, for 14 days *warn about alcohol*	q1116c1c
PID 1st line treatment if lactating mother or pregnant	Erythromycin 500mg qds for 7 days	q1116c2a
	OR Ceftriaxone 250 mg im, stat	q1116c2b
	AND Metronidazole 400mg bid, for 14 days *if not in 1st trimester of pregnancy warn about alcohol*	q1116c2c
PID 2nd line treatment	Ceftriaxone 250 mg im, stat	q1116c3
**URETHRAL DISCHARGE**		
Urethral discharge 1st line treatment	Ciprofloxacin 500 mg, stat	q1116d1a
	Doxycycline 100 mg, bid for 7 days	q1116d1b
Urethral discharge 2nd line treatment	Doxycycline 100 mg, bid for 7 days	q1116d2a
	Ceftriaxone 250 mg im, stat	q1116d2b
	Metronidazole 2 g, stat *warn about alcohol*	q1116d2c
**EPIDIDYMO-ORCHITIS**		
Painful scrotal swelling (epididymo-orchitis)	Ciprofloxacin 500 mg, stat	q1116e1
	Doxycycline 100 mg, bid for 7 days.	q1116e2
**BUBO**		
Bubo 1st line treatment	Doxycycline 100 mg, bid for 14 days	q1116f1
Bubo 1st line treatment if pregnant	Erythromycin 500mg qds for **14** days	q1116f2
**PUBIC LICE**		
Pubic lice treatment	Lindane 1% lotion or cream. Apply and wash off after 8hrs	q1116g1
	**OR** Lindane shampoo. Apply and wash off after 4 minutes	q1116g2
**BLISTERS/VESICLES**		
Blisters/vesicles	Gentian Violet paint	q1116h
**SCABIES**		
Scabies treatment	Benzyl Benzoate 25% lotion nightly for 2 nights	q1116i
**SKIN CONDITIONS**		
Skin conditions	Whitfield's ointment	q1116j

MEMA kwa Vijana Trial Further Survey (2007-2008)

**PAIN RELIEF**		
Analgesia	Paracetamol 500mg (no more than 6 tablets per day for 2 days) *write number of tablets given*	q1116k
**SCHISTOSOMIASIS**		
Schistosomiasis	Praziquantel *tick here if given*<br>Weight in kilograms<br>State number of tablets given	45 ✓ q1116l1<br>45a NNN kg q1116l2<br>45b N q1116l3
**COUGH**		
Cough	Erythromycin 500mg, tid, 5 days	q1116m1
	Cotrimoxazole 960mg, bid, 5 days	q1116m2
**MALARIA**		
Malaria	ALU 4 st, 4 after 8 hrs, 4 bid for 2 days	q1116n
**OTHER MEDICATIONS**		
Indication ______________________	Other (specify name of drug, number of tablets and dose) ______________________	q1116o1a q1116o1b (string)
Indication ______________________	Other (specify name of drug, number of tablets and dose) ______________________	q1116o2a q1116o2b (string)

12. VOLUNTARY HIV TESTING (completed by COUNSELLOR)

All participants should be given the following information about the voluntary HIV testing service.
As the interviewer told you earlier, all the information and the results of the tests on the urine, and blood you have given us will be kept secret. However, if you would like to know whether you are infected with HIV, there is a separate procedure to be followed. If you are interested, I can tell you about this procedure. If, after that, you want to continue, we can do a test now and I can tell you the results of your HIV test. I will also give you further advice. Nobody except you, me and the project's senior staff in Mwanza will know the results

Washiriki wote wapewe taarifa hii ya huduma ya kupima virusi vya UKIMWI kwa HIARI
Kama msaili alivyokueleza hapo awali, taarifa zote na majibu ya vipimo vya mkojo na damu tulivyochukua vitakuwa vya siri kabisa. Hata hivyo, kama unataka kujua kama umeathirika na virusi vya UKIMWI, kuna utaratibu tofauti wa kufuata, kama utapenda ninaweza kukuelezea utaratibu wa kufuata, na kama baada ya hapo utapenda kuendelea nitafanya kipimo na kukupatia majibu yako sasa hivi, na nitakupatia ushauri zaidi. Hakuna mtu zaidi yako wewe, mimi na mkuu wa mradi huko Mwanza atakayejua majibu ya kipimo chako.

12.01	Counsellor's staff code	q1201				
12.02	Do you want to be told your HIV test result?<br>*If Yes, give the pre-test counselling.*	q1202<br>*(Circle one)*	Yes<br>No	1<br>2	 → end
12.03	Are you satisfied with this pre-test counselling?<br>*If NO, then do not proceed with test but carry out full pre-test counselling*	q1203<br>*(Circle one)*	Yes<br>No	1<br>2	→ 12.05<br>→ 12.04
12.04	Are you <u>now</u> satisfied with this pre-test counselling?	q1204<br>*(Circle one)*	Yes<br>No	1<br>2	 → end
12.05	Do you want to be told your HIV result?	q1205<br>*(Circle one)*	Yes<br>No	1<br>2	 → end
	If participant wants to be told HIV result complete the Request for HIV Test Results Form, carry out the rapid HIV tests and carry out post-test counselling				
12.06	Have you completed a Request for HIV Test Results form?	q1206<br>*(Circle one)*	Yes<br>No	1<br>2	 → end
12.07	Have you given full post-test counselling?<br><br>If no, why was full post-test counselling not given?	q1207<br>*(Circle one)*<br><br>____________________	Yes<br>No	1<br>2	
	If the Request form is completed and you have given full post-test counselling then give the participant their result. Provide further counselling and advice.				

MkV1 Trial Further Survey Main Questionnaire

Weka stika hapa
kama anastahili: FS_ _ _ _ _ _ _
kama hastahili: XFS_ _ _ _ _ _

Sehemu A: 1. Namba ya mshiriki (Ijazwe na msaili wa usajili)		
1.01	Tarehe ya usaili (tarehe/mwezi/mwaka)	/ /200
1.02	Kata *(andika jina la kata na namba ya siri)*<br><br>Jina la Kata: _.. _ _ _ _ _ _ _	
1.03	Kijiji *(andika jina la kijiji na namba ya siri)*<br><br>Jina la Kijiji: _.. _ _ _ _ _ _ _	
1.04	Alama ya siri ya msaili	
1.05	Jinsia ya mshiriki (Usimuulize mshiriki)	*(Zungushia moja)* Mwanaume 1<br>Mwanamke 2
1.06	Je, unayo barua ya mwaliko wa MkV1 FS?<br><br>*Uli na nyaluba ya kwitanwa na MKV1 FS?*	*(Zungushia moja)* Ndiyo 1<br>Hapana, ila niliupokea 2<br>Sikuupokea kamwe 3
1.07	**Utambulisho**: Je, jina la mhudhuriaji lipo kwenye orodha A?<br>TAFUTA JINA LA MHUDHURIAJI KWENYE ORODHA YA SENSA (ORODHA A).	*(Zungushia moja)* Ndiyo 1<br>Hapana 2
1.08	Namba ya utambulisho ya sensa<br>Kama mwaliko umetolewa au jina kwenye orodha ya sensa basi ingiza namba ya Utambulisho ya sensa. Kama hakuna mwaliko au hayumo kwenye orodha basi ingiza 88888 88 88	
1.09	Ulizaliwa tarehe gani?<br>*Ukabyalwa tarehe jinga?*<br>*Kama haufahamika (NK) ingiza 99/99/9999*<br><br>*Kama <u>tarehe kamili</u> ya kuzaliwa HAIFAHAMIKI basi uliza*<br>Una umri gani?<br>Uli na myaka yinga?<br>*Tumia kalenda ya matukio kumsaidia kijana kukumbuka umri wake katika miaka kamili.*	/ /19<br>Kama tarehe kamili inafahamika → 1.10<br><br>Umri kwa miaka<br>99= Haifahamiki
1.10	Uthibitisho wa umri *(Zungushia moja)*	Cheti cha kuzaliwa 1<br>Cheti ya Kliniki/MCH 2<br>Cheti cha ubatizo 3<br>Kadi ya utambulisho wa upimaji wa afya 4<br>Cheti cha kumaliza shule 5<br>Kitambulisho cha mpiga kura 6<br>Maandishi mengine 7<br>Hakuna maandishi 8

1.11 Je, katika shule ya msing ni darasi gani la juu ulisoma na kulimaliza? (Liwe ni darasa alilosoma kwa mwaka mzima)
Ukasoma shule ya msingi mpaga mu kanga?
(zungushia moja)

KAMA MHUDHURIAJI HAKUFIKA DARASA LA 5 KATIKA SHULE YA MSINGI BASI HASITAHIRI KUENDELEA – MSHUKURU NA UMALIZE MAHOJIANO.

KAMA HAFAHAMU AU HAWEZI KUKUMBUKA BASI ENDELEA

Darasa 1-4	4	→maliza mahojiano
Darasa 5	5	
Darasa 6	6	
Darasa 7	7	
Sikuhudhuria shule ya msingi	8	→ maliza mahojiano
Sifahamu/sikumbuki	9	

1.12 Je, ni shule gani za msingi ulizosoma?
Ukasomela shule ki ya/ja msingi?

Kagua jina/majina ya shule ya/za msingi kwenye orodha ya shule za msingi kwenye kata na ingiza jina la shule na alama ya siri ya shule. Kwa shule isiyokuwepo kwenye orodhai ingiza namba ya siri 999

Jina la shule: ______________________ Namba ya siri:

Jina la shule: ______________________ Namba ya siri:

Jina la shule: ______________________ Namba ya siri:

KAMA HAKUHUDHURIA SHULE YA MSINGI INAYOSTAHILI BASI MSHUKURU NA UMALIZE MAHOJIANO

1.13 Je, ni darasa gani ulikuwa kwenye shule ya msingi husika katika (mwaka)?

idalasa ki wali mu shule ya msingi mung'waka ?

Uliza kwanza 2004 kisha 2003 nakuendelea

Kama hakuwa shule ya msingi basi ingiza namba ya siri ya shule =888 na namba ya siri ya darasa=8.

<u>Namba ya siri ya shule</u>
Ingiza namba ya siri kutoka swali 1.12 juu

(888= Hakuwa shuleni)
(999=shule haistahili)

<u>Namba ya siri ya darasa</u>
Ingiza namba ya siri
Mf: darasa 1 = '1'
Hakuwa shuleni = '8'

Mwaka *Ng'waka*	Namba ya siri *Namba ya mbisila*	Darasa *Idalasa*
2004		
2003		
2002		
2001		
2000		
1999		
1998		

1.14 Kipindi cha nyuma uliwahi kushiriki kwenye utafiti wa afya ambapo vipimo (mkojo na/au damu) vilichukuliwa?

Umu shiku ja hanuma wagema kubiza mu butafiti wa afya ng'witilwa na buping'wa wa mine na/nulu mininga?
Kama HAPANA basi nenda swali la 1.18

(Zungushia moja)

Ndiyo	1	
Hapana	2	→*1.18*

1.15 Utafiti upi

Butafiti ki?
Kama hakushiriki kwenye MkV1 basi nenda swali la 1.18

(Zungushia moja)

MkV1	1	
Mwingine	2	→*1.18*

1.16 Unayo kadi ya MkV1?
Ulina Kadi ya MkV1?
- *Kama anayo kadi basi onyesha rangi ya kadi kwa kuzungushia jibu.*
- *Kama hana kadi ya MkV1 basi angalia kwenye orodha ya washiriki wa MkV1 kuona kama yuko.*

KAMA 4 = HAYUMO KWENYEORODHA BASI NENDA Q 1.18

Kadi nyeupe ya MkV1	1	
Kadi ya bluu ya MkV1	2	
Jina kwenye orodha ya MkV1	3	
Hayumo kwenye orodha	4	→*1.18*

1.17 Namba ya utambulisho ya *MkV1*
(andika namba toka kwenye kadi ya utambulisho au orodha ya MkV1)

⊗ ⊗ ⊗ ⊗ ⊗

MTU HUYU ANASTAHILI, Tafadhari nenda Q1.19

1.18	Angalia Q 1.13 kuona ilikuwa ni mwaka gani alikuwa darasa la 7 AU ni mwaka gani angekuwa darasa la 7 kama angefika darasa la 7.<br><br><u>Chukua orodha D</u><br>Tafuta jina lake (muhudhuriaji)<br>AU kama aliacha kabla ya darasa la 7 lazima ataje wanadarasa wenzie 2 waliofika darasa la 7	Mwaka darasa la 7<br><br>*(Zungushia moja)*<br>Orodha ya darasa la 7 (Orodha D) 1 →Ana*stahili*<br>Orodha ya darasa la 7 (Orodha D) – wanadarasa wenzie 2 →Ana*stahili*<br>Haipo kati ya hizi juu 3 → *Hastahili*<br>*Kama jibu ni “3”=”Hastahili” Basi Mshukuru na umalize mahojiano*
1.19	ANASTAHILI?	*(Zungushia moja)*<br>Ndiyo 1<br>Hapana 2
TAFADHALI PIMA KAMA MTU ANAYESITAHIRI ANAWEZA KUTOA TAARIFA YA RIDHAA NA KAMA NDIVYO MUOMBE ASAINI FOMU YA RIDHAA		
1.20	Je, mshiriki amesaini fomu ya ridhaa/ makubaliano?	*(Zungushia moja)*<br>Ndiyo 1<br>Amekataa 2<br>Hawezi kutoa ridhaa 3
	Kama amekataa kujaza AU hawezikutoa ridhaa basi HASTAHILI kuendelea. Mshukuru na maliza usahili.	
1.21	Uliza RIDHAA kwa ajili ya kufuatilia matibabu kama yatahitajika:<br>Je, kama vipimo vya maabara Mwanza vitaonyesha kwamba una ugonjwa wa zinaa (kama vile kaswende, Clamidia, NG) Utapenda turudi kukutibu?<br><br>*Ulu shipimo sha maabara ga Ng’wanza sholekeja giki uli na busatu wa bunyolo (Kaswende, CT, NG) ukutogwa tushoke kwiza kukulagula*	*(Zungushia moja)*<br>Ndiyo 1<br>Hapana 2

MEMA kwa Vijana Trial Further Survey (2007-2008)

Weka stika hapa
FS_ _ _ _ _ _ _ _

Sehemu B (ijazwe na msaili mkuu)

2. Taarifa ya takwimu za watu

2.01	Alama ya siri ya msaili mkuu		
2.02	*SOMA MAELEZO HAYA*<br>Nitakuuliza maswali kadhaa kwa muda kama wa nusu saa hivi. Ninaweza kukuuliza maswali kwa Kiswahili au Kisukuma, kutegemea na jinsi unavyopenda. Je, utapenda nikuulize maswali kwa Kiswahili au Kisukuma?<br>*Nakukubuja mabujo ganga ganga/malebe ayo gakusola makanza giti ga nusu saa giki. Nadul'ukubuja mabujo aya mu Kiswahili nulu mu Kisukuma, bebe ng'wenekele umo ulatogelwe. Kinehe ulitogwa nagabuje a mabujo mu Kiswahili nulu mu Kisukuma?*<br>***Kama ni Kiswahili, mueleze:***<br>"Kumbuka kwamba ninaweza pia kutumia Kisukuma wakati wowote kama unafikiri utaelewa vizuri. Nieleze kama ungependa nitumie lugha ya Kisukuma."	*(Zungushia moja)*<br>Kiswahili<br>Kisukuma	 1<br>2
2.03	Je, wewe ni kabila gani?<br>*Ubebe uli kabila ki*	*(Zungushia moja)* Msukuma<br>Mjita<br>Mzinza<br>Mkara<br>Mkerewe<br>Msumbwa<br>Mrongo<br>Nyingine………………………………………………	1<br>2<br>3<br>4<br>5<br>6<br>7<br>8
2.04	Je, wewe ni dini gani?<br>*Bebe uli dini ki?*	*(Zungushia moja)* Katoliki<br>Nyingine ya kikristo<br>Mwislamu<br>Dini nyingine (pamoja na asili)<br>Hana dini	1<br>2<br>3<br>4<br>5
2.05	Umefikia kiwango gani cha juu cha elimu?<br>*Wasoma mpaga mu kanga?*	*(Zungushia moja)* Sikumaliza shule ya msing<br>Nilimaliza shule ya msing<br>Sikumaliza shule ya sekondari<br>Shule ya sekondari kidato cha 4<br>Shule ya sekondari kidato cha 6<br>Mafunzo ya Ufundi<br>Chuo (e.g. Chuo cha ualimu, Chuo cha polisi, n.k.)<br>Chuo kikuu<br>Masomo mengine (e.g.Chuo cha kiislamu: chuo cha thiolojia)	1<br>2<br>3<br>4<br>5<br>6<br>7<br>8<br>9

2.06	Ni kazi au shughuli gani unafanya/ umewahi kufanya ndani ya cha miezi 12 iliyopita?<br>*Milimo ki nulu shuguli ki iyo ukitaga nulu iyo walitaga umumakanza ga myeji 12 iyashilile?*<br>*Usimtajie majibu msailiwa, lakini kila baada ya jibu, sema, "Asante. Kazi nyingine?"*<br>*Weka vema kwa kila lililo tajwa*<br>Mfanyakazi Mgodini- Kazi ya ujuzi (Uhandisi, Uhasibu, surveya n.k.)<br>Mfanyakazi Mgodini- Kazi isiyo ya ujuzi (kibarua, Ulinzi, dereva)<br>Kazi ya ujuzi (eg Mwalimu, Muuguzi, Muhasibu, Polisi<br>Kazi nyingine isiyo ya ujuzi (eg seremala/ fundi cherehani)<br>Biashara kubwa (eg mmiliki wa duka, gereji, baa & gesti, shamba kubwa la kilimo cha biashara)<br>Biashara ndogo ndogo (mf: vibanda na biashara za magengeni jioni, Mama lishe, mpika pombe)<br>Mhudumu wa Baa, Mtumishi wa gesti, Mtumishi wa hoteli<br>Dereva<br>Tingo wa loli<br>Mvuvi<br>Mkulima<br>Mama wa nyumbani<br>Mtumishi wa kazi za ndani (nyumbani)<br>Yupo chuo au shule<br>Hapana shughuli<br>Nyingine (Taja)………………………… ……………………………………………………...)
2.07	Je, katika wiki nne zilizopita, umeishalala nje ya Kata yako?<br>*Umunyuma inne ijabitile walalalile hanze ya Kata yako?*<br><br>*Kama "NDIYO"*→ Siku ngapi? (Ingiza idadi ya siku)<br>*Kama "HAPANA*" INGIZA "00"<br>Kama NDIYO, pia zungushia 1 kwa swali la 2.08 na uliza→ Je, nimala ngapi umelala nje ya kata yako angalau usiku mmoja katika miezi 12 iliyopita?
2.08	Je katika miezi 12 iliyopita, umeishawahi kulala nje ya kata yako?<br>*Umu myeji ikumi n'ibili iyo yabitile, wagema nulu kamo kulala hanze ya Kata yako?*<br><br>Ndiyo 1<br>*(Zungushia moja)* Hapana 2 *→3.01*<br>Sifahamu 9 *→3.01*<br>*Kama 'Hapana' au 'Sifahamu' nenda → 3.01*<br><br>Kama ndiyo: Je, ni mara ngapi umelala angalau usiku 1 nje ya kata yako?<br>Kama ndiyo: Kwa ujumla ni kipindi cha muda gani ulilala nje ya Kata yako katika kipindi cha miezi 12 iliyopita? NNN<br>unit U<br>*Ingiza kama mshiriki anavyochagua kujibu: Onyesha kipimo (Siku, Wiki, Mwezi, Mwaka) kwenye kisanduku cha mwisho k.m.'003M'=miezi mitatu;* '060D'=siku sitini; '012W'=wiki kumi na mbili; *'999x'=NK*

3. Elimu ya afya ya uzazi na jinsia na tabia ya kufanya mapenzi

Sasa napenda kukuuliza maswali machache kuhusu Afya, Magojwa na kufanya mapenzi. Kila wakati ninapotaja neno kufanya mapenzi ninazungumzia kuhusu kujamiiana na mtu. Hii ni pamoja na kujamiiana ambapo mmoja kati ya wawili, hakukubali (mmoja amelazimishwa).

Ihaha nditogwa na kubuje mabujo magehu juu ya Afya, Basatu na kwilala. Buli ulu nayomba mhayo "kwilala" ndiyombela kuhusu kwilala na mun'hu. Yeniyi ili kihamo na kulala na mun'hu umo wa babili benabo atazunije (umo wabo wahadikijiwagwa).

3.01	Je, kutokwa usaha au maji maji yasiyo ya kawaida sehemu za siri kunaweza kuambukizwa kwa kufanya mapenzi?<br><br>*Kuzwa buhila nulu minzi minzi kuwima mbele kudugije kwenhwa kulwa nguno ya kwilala?*	*(Zungushia moja)*	Ndiyo<br>Hapana<br>Sifahamu	1<br>2<br>9
3.02	Je, kichocho kinaweza kuambukizwa kwa kufanya mapenzi na mtu?<br><br>*Udugije kutulilwa kisambale ku lwakwilala na mun'hu?*	*(Zungushia moja)*	Ndiyo<br>Hapana<br>Sifahamu	1<br>2<br>9
3.03	Je, vidonda kwenye sehemu za siri vinaweza kuambukizwa kwa kufanya mapenzi?<br><br>*N'hondo ku wimambele jidugije kwenhelejiwa ku lwa kwilala?*	*(Zungushia moja)*	Ndiyo<br>Hapana<br>Sifahamu	1<br>2<br>9
3.04	Je, virusi vya UKIMWI vinaweza kuambukizwa kwa kufanya mapenzi?<br><br>*BUKIMWI budugije kutulilwa ku lwa kwilala?*	*(Zungushia moja)*	Ndiyo<br>Hapana<br>Sifahamu	1<br>2<br>9
3.05	Je, unaweza kuambukizwa virusi vya UKIMWI kwa kula katika sahani moja na mtu mwenye virusi vya UKIMWI?<br><br>*Udugije kutulilwa BUKIMWI ku lwa kulila musahani imo na mun'hu uyo alina BUKIMWI?*	*(Zungushia moja)*	Ndiyo<br>Hapana<br>Sifahamu	1<br>2<br>9
3.06	Je, mtu anayeonekana kuwa na nguvu na afya nzuri anaweza kuwa na virusi vya UKIMWI?<br><br>*Ulu mun'hu alibonekana alina afya ya wiza adugije kubiza na BUKIMWI?*	*(Zungushia moja)*	Ndiyo<br>Hapana<br>Sifahamu	1<br>2<br>9
3.07	Je, kama mwanaume au mvulana anataka kufanya mapenzi na msichana, msichana anaweza kukataa kufanya naye mapenzi kama mwanaume huyo ana umri mkubwa kuliko yeye?<br><br>*Ulu ngosha nulu nyanda ulu alihaya/alitogwa kwilala na ng'waniki, ng'waniki adugije kulema kwilala ulu ngosha ali na umri butale kukila uwakwe?*	*(Zungushia moja)*	Ndiyo<br>Hapana<br>Sifahamu	1<br>2<br>9
3.08	Je, kama mwanaume au mvulana anataka kufanya mapenzi na msichana, msichana anaweza kukataa kufanya naye mapenzi kama mwanaume au mvulana huyo ni mpenzi wake?<br><br>*Ulu ngosha nulu nyanda ulu uhaya kwilala na ng'waniki adugije kulema kwilala ulu ngosha nulu nyanda ng'wenuyo ali nsumba wakwe?*	*(Zungushia moja)*	Ndiyo<br>Hapana<br>Sifahamu	1<br>2<br>9

3.09	Je, kama msichana akikubali zawadi kutoka kwa mwanaume au mvulana nje ya familia, ni lazima akubali kufanya naye mapenzi?<br><br>*Ng'waniki ulu uzunya kwanukula manong'ho kwinga kuli ngosha nulu kuli nyanda uyo ati wa mukaya yabo, ili lazima azunye kwilala nang'hwe?*	*(Zungushia moja)*	Ndiyo<br>Hapana<br>Sifahamu	1<br>2<br>9
Sasa napenda kukuuliza maswali machache kuhusu mimba.<br>*Ihaha nditogwa nakubuje mabujo magehu kuhusu nda.*				
3.10	Je, inawezekana msichana kupata mimba mara ya kwanza anapofanya mapenzi?<br><br>*Ng'waniki adugijje kubiza budito ku lwa kwilala na mun'hu lukangala lwa kwandya?*	*(Zungushia moja)*	Ndiyo<br>Hapana<br>Sifahamu	1<br>2<br>9
3.11	Je, inawezekana mtu kuzuia mimba kwa kutokufanya mapenzi kabisa?<br><br>*Idulikanile mun'hu kukija kubiza nda getu ku lwakulela kwilala?*	*(Zungushia moja)*	Ndiyo<br>Hapana<br>Sifahamu	1<br>2<br>9
3.12	Je, inawezekana mtu kuzuia mimba kwa kutumia kondom wakati wa kufanya mapenzi?<br><br>*Idulikanile mun'hu kukija kubiza nda ulu utumila kondomu ikanza lya kwilala?*	*(Zungushia moja)*	Ndiyo<br>Hapana<br>Sifahamu	1<br>2<br>9
Sasa napenda kukuuliza maswali machache kuhusu kondomu; Ninaposema kondomu kila mara ninamaanisha mpira wa uume unaotumika wakati wa kujamiiiana<br>*Ihaha nditogwa nakubuje mabujo magehu kuhusu kondomu.*				
3.13	Je unaifahamu kondom?<br><br>*Wimanile ikondomu?*<br>*Kama HAPANA → Nenda swali la 4.01*	*(Zungushia moja)*	Ndiyo<br>Hapana	1<br>2 →*4.01*
3.14	Je, kuna mtu yeyote aliyewahi kukuonyesha jinsi ya kutumia kondomu?<br><br>*Aliho mun'hu wose wose uwagema na kamo kukolekeja umo kondomu ikatumamilagwa?*	*(Zungushia moja)*	Ndiyo<br>Hapana<br>Sifahamu	1<br>2<br>9
3.15	Je, baadhi ya kondomu mpya huwa na virusi vya UKIMWI kabla hazijatumiwa?<br><br>*Kondomu jikabyagi na BUKIMWI kuko jitinandya kutumamilwa?*	*(Zungushia moja)*	Ndiyo<br>Hapana<br>Sifahamu	1<br>2<br>9
3.16	Je, kutumia kondom kunaweza kusaidia kuzuia wanaume na wanawake kuambukizana virusi vya UKIMWI?<br><br>*Kutumamila kondomu kudugijje kunzuia ngosha na nkima kutulilwa BUKIMWI?*	*(Zungushia moja)*	Ndiyo<br>Hapana<br>Sifahamu	1<br>2<br>9
3.17	Je, ni wapi ambapo unaweza kupata kondomu ukizihitaji kwenye kijiji chenu?<br><br>*Hali uko udugijje kupandika kondomu ulu ulijitogwa u munzengo gwing'we?*<br><br>***Usimtajie majibu msailiwa, lakini kila baada ya jibu, uliza, "Asante. Sehemu nyingine?***<br><br>(*WEKA VEMA KWA KILA LILILO TAJWA*)		Duka/Kibanda/duka la dawa<br>Kituo cha afya<br>Muwakilishi wa shirika lisilo la kiserikali<br>Mtu binafsi mwingine<br>Nyingine (taja): ____________<br>Sifahamu	

3.18	Je, ni wapi ambapo mtu anaweza kupata kondomu za bure kijijini kwenu?<br><br>*Hali u munzengo gwing'we uko mun'hu adugije kupandika kondomu ja dalali?*<br><br>*Usimtajie majibu msailiwa, lakini kila baada ya jibu, uliza, "Asante. Kitu kingine?"*<br><br>*(WEKA VEMA KWA KILA LILILO TAJWA)*	Duka/Kibanda/duka la dawa<br>Kituo cha afya<br>Muwakilishi wa shirika lisilo la kiserikali<br>Mtu binafsi mwingine<br>Nyingine (taja): ____________<br>Sifahamu

4. Tabia ya kufanya mapenzi

Sasa napenda kukuuliza tena maswali machache kuhusu kufanya mapenzi. Kama nilivyotaja awali, kila wakati ninapotaja neno kufanya mapenzi ninazungumzia kuhusu kujamiiana na mtu. Hii ni pamoja na kujamiiana ambapo mmoja kati ya wawili, hakukubali (mmoja amelazimishwa). Tunafahamu kuwa vijana kama wewe tayari wamekwishafanya mapenzi na wengine bado. Tunapenda tu kusikia ukweli kuhusu uzoefu wa vijana katika kufanya mapenzi. Majadiliano haya ni ya siri sana kati yako na mimi, hivyo natumaini kwamba utakuwa huru kuniambia kuhusu uzoefu wako katika kufanya mapenzi. Baadhi ya maswali haya yanahusu vipindi virefu vya miezi na miaka. Inaweza kuwa vigumu kukumbuka kwa haraka uzoefu kuhusu kipindi kirefu kama hicho. Cha muhimu zaidi ni kwamba ujibu maswali kama hayo kikamilifu na kwa usahihi kama iwezekanavyo. Hivyo tafadhali, chukua muda kadri unavyohitaji kufikiri kuhusu hayo wakati unapojibu.

Ihaha nditogwa na kubuje hangi mabujo magehu juu ya kwilala. Gitumo nali nayombela hawandijo, buli ulu nayomba mhayo "kwilala" ndiyombela kuhusu kwilala na mun'hu. Yeniyi ili kihamo na kulala na mun'hu umo wa babili benabo atazunije (umo wabo wahadikijiwagwa). Tumanile giki basumba/bang'hya giti bebe tayali bamala kwilala na ban'hu. Mahoya genaya gali ga mbisila sana, gali gise babili duhu, bebe na nene, giko lulu ndiganika giki ukubiza na wiyabi wa kuniwila kuhusu bumani wako wa mambo ga wilaji. Mabujo gangi umu genaya galihusu makanza malihu ga myeji na myaka. Idugije kubiza shidamu kwizukwa wangu bumani wako wa makanza malihu giti genayo. Kin'u ishitale zaidi ili giki kushosha mashosho ga mabujo giti genaya mung'hana gitumo udugigije. H'igiko lulu ndikulomba usole makanza ayo ulitogwa kwiganika yeniyo makanza ulu ulishosha.

4.01	Je, umeshawahi kufanya mapenzi na mtu yoyote?<br><br>*Wagema kwilala na mun'hu wose wose?*<br><br>*Kama jibu ni "HAPANA", Dadisi kwa kuuliza: "Je nimekuelewa vizuri, kwamba haujawahi kufanya mapenzi au kujamiana na mtu yoyote katika maisha yako yote?"*<br>*Kama bado ataendelea kusema hajawahi kufanya mapenzi: zungushia "HAPANA=2"*	*(Zungushia moja)*	Ndiyo<br>Hapana	1<br>2
Kama jibu ni bado hajawahi kufanya mapenzi nenda swali la 8.				
4.02	Je, umeshawahi kutumia kondomu wakati wa kufanya mapenzi<br><br>*Wagema kutumamila kondomu ikanza aho ng'wilalaga ?*	*(Zungushia moja)*	Ndiyo<br>Hapana<br>Sifahamu	1<br>2<br>9
4.03	Sasa napenda ufikirie <u>mara ya kwanza</u> ulipofanya mapenzi na mtu. Je, ulikuwa na umri gani wakati ulipofanya mapenzi kwa <u>mara ya kwanza</u>?<br><br>*Ihaha nditogwa wiganike <u>ulukangala lwa kwandya</u> aho ukilala na mun'hu. Wali na myaka yinga aho ulilala na mun'hu <u>lukangala lwa kwandya?</u>*<br><br>*(Tafadhali jaribu kumsaidia mshiriki kukumbuka umri halisi wakati alipofanya mapenzi kwa mara ya kwanza k.m. ulikuwa darasa la ngapi katika shule ya msingi n.k.)*<br><br>Ingiza miaka kamili ☐☐ miaka<br><br>*Kama miaka halisi haifahamiki msaidie kukadiria miaka na weka vema kwenye boksi chini*<br><br>miaka 12 au pungufu 44 miaka 13-14 55 miaka 15-17 66 18 au zaidi 77 NK 99			

4.04	Sasa, naomba ufikiri juu ya mtu uliyefanya naye mapenzi kwa mara ya kwanza. Je, mtu uliyefanya naye mapenzi kwa mara ya kwanza alikuwa na umri mkubwa kuliko wewe, sawa na umri wako au na umri mdogo kuliko wewe?<br><br>*Ihaha ndilomba ung'wizuke umun'hu uyo ukilala nang'hwe lukangala lwa kwandya. Mun'hu uyo ukilala nang'hwe lukangala lwa kwandya wali ntale kukukila, ng'wali mulenganilile nulu wali ndo kukukila ubebe?*<br><br>***Kama jibu ni "3=umri sawa" au "9=hafaham" Nenda swali la 4.06***	*(Zungushia jibu moja)*<br>Umri mkubwa 1<br>Umri mdogo 2<br>Umri sawa 3 → q 4.06<br>Hafahamu 9 → q 4.06
4.05	Je, mtu uliyefanya naye mapenzi kwa mara ya kwanza alikuwa anakuzidi au unamzidi miaka mingapi?<br><br>*Umun'hu uyo ukilala nang'hwe lukangala lwa kwandya wali akukile nulu wali unkilile myaka yinga"*<br><br>***Kama miaka halisi haifahamiki msaidie kukadiria miaka na weka vema kwenye boksi chini***<br><br>miaka 1au pungufu 44 miaka 2-4 55 miaka 5-9 66 miaka 10-14 77 miaka 15 au zaidi 88 NK 99	Miaka
4.06	Sasa, naomba ufikiri juu ya watu wote uliofanya nao mapenzi katika wiki nne zilizopita: Je, ni watu wangapi umefanya nao mapenzi katika muda wa wiki nne zilizopita?<br><br>*Ihaha ndikulomba wiganike/wizukwe aban'hu bos abo ukilala nabo nyuma ine ijabitile: Ban'hu banga abo wilalile nabo umu nyuma ine ijabitile?*	Ingiza idadi (tarakim mbili)
4.07	Sasa, naomba ufikiri juu ya sehemu ulizowahi kuishi katika kipindi cha mwaka mmoja ulio pita. Kumbuka itakuwa kuanzia mwezi wa (***Taja mwezi huu)*** mwaka jana hadi leo au Kipindi cha (***taja majira mf masika, kiangazi***) mwaka jana hadi leo. Tafadhari tumia muda unaositahili kukumbuka. (***Mpatie muda mfupi wa kukumbuka kama dak. 2***) Je, unakumbuka?<br>Je, sasa unaweza kukumbuka ni jumla ya watu wangapi uliofanya nao mapenzi katika kipindi cha miezi 12.<br><br>*Ihaha ndikulomba wiganike kuhusu mapandi ayo wagema kwigasha/kuzenga umu ng'waka gumo ugo gwabitile. Izukagwa ibize kwingila ng'weji (**Taja mwezi huu)** g'wakakezo mpaga lelo iyi nulu makanza xxxx mpaga lelo. Tumilaga makanza ayo gigelile kwizukwa. (**Mpatie muda mfupi wa kukumbuka kama dak. 2**) Ulizukwa?*<br>*Udugije ihaha kwizukwa ijumula ya ban'hu banga abo wilala nabo umu makanza genayo?*<br>***Angalia kama namba hii inajumuisha wapenzi wa wiki 4 zilizo pita,***<br>***Kama jibu ni 00 andika 88 kwenye swali 4.08***	Ingiza idadi (tarakim mbili)<br>*Haifahamiki = 99*
4.08	Je, wangapi kati ya hawa watu uliofanya nao mapenzi katika kipindi cha mwaka mmoja uliopita walikuwa wapenzi wapya kwako? (Hii ina maana ulifanya nao mapenzi kwa mara ya kwanza katika kipindi cha mwaka mmoja).<br><br>*Banga umu ban'hu benabo abo ukilala nabo ng'waka gumo ugwabitile bali basumba/bang'hya bako bapya? (Yeniyi ili kihamo na babo abo ukilala nabo lukangalwa lwa kwandya mu makanza ga ng'waka gumo).*	Ingiza idadi (tarakim mbili)<br>*Haihusiki = 88*<br>*Haifahamiki = 99*

KAMA HAJAFANYA MAPENZI KATIKA KIPINDI CHA MWAKA MMOJA ULIOPITA, NENDA q6.01

5. Wapenzi KATIKA KIPINDI CHA MIEZI 12 ILIYO PITA (ijazwe na msahili mkuu)

*** * * *Muhimu: Jaza safu moja kwa kila mpenzi ukianza na mpenzi wa hivi karibuni sana * * * ***

Fikiria kuhusu mtu wa mwisho/wa pili toka mwisho/wa tatu toka mwisho uliyefanya naye mapenzi katika miezi kumi na mbili iliyopita. Hawa siyo lazima wawe wapenzi wapya. Unawakumbuka?

Wiganike munhu u wa ng'wisho/wa kabili kwinga wa ng'wisho/wa kadatu kwinga kung'wisho uyo ukalala nang'hwe myeji ikumi n'ibili iyashilike. Iti lazima babi basumba/bang'hya bapya. Ulibizukwa?

HAPANA→ Jaribu kumfanya akumbuke kwa kuainisha kipindi mfano: tangu Krismasi iliyopita
NDIYO → endelea

		1. Mwisho (hivi karibuni sana)	2. Pili kutoka mwisho (karibuni kiasi)	3. Tatu toka mwisho (karibuni)
5.01	Je, alikuwa mkeo/mumeo, mpenzi mwingine wa kudumu, mpenzi wa muda mfupi, au Mfanyabiashara ya ngono?<br>*Wali nke/ngoshi wako, mung'hya/nsumba wa shiku ningi, mung'hya/nsumba wa nshiku ngehu, nu Malahya?*<br>*(zungushia moja kwa kila mpenzi)*<br>Mkeo/Mumeo<br>Mpenzi mwingine wa kudumu<br>Mpenzi wa muda mfupi<br>Mfanyabiashara ya ngono	1<br>2<br>3<br>4	1<br>2<br>3<br>4	1<br>2<br>3<br>4
5.02	Je, unaishi naye huyu mtu? (*zungushia moja kwa kila mpenzi*)<br>*Ukigashaga n'umunhu ng'wenuyo?*<br>Ndiyo<br>Hapana	1<br>2	1<br>2	1<br>2
5.03	Je, mtu huyu ni mkubwa, mdogo ua ana umri sawa na wako?<br>*Mun'hu ng'wenuyo ntale, ndo nulu mlenganilile?*<br>(*zungushia moja kwa kila mpenzi*)<br>Mkubwa<br>Mdogo<br>Umri sawa →q.5.05<br>Sifahamu →q.5.05	1<br>2<br>3<br>9	1<br>2<br>3<br>9	1<br>2<br>3<br>9
5.04	Je, mtu huyo anakuzidi au una mzidi miaka mingapi?<br>*Mun'hu ng'wenuyo akukilile nulu unkilile myaka yinga?*<br>***Kama miaka halisi haifahamiki jaza namba "99"***	NN	NN	NN
5.05	Mpenzi huyo alisoma shule ya msingi katika kata hii?<br>*Mung'hya/nsumba wako ng'wenuyo ali mu shule ya msingi mu Kata iyi?*<br>(*zungushia moja kwa kila mpenzi*)<br>Ndiyo<br>Hapana<br>Sifahamu	1<br>2<br>9	1<br>2<br>9	1<br>2<br>9

5.06	Je, ni darasa gani la juu shule ya msingi mpanzi wako alisoma na kumaliza?<br><br>*Idalasa ki ilya highulya mu shule ya msingi ilo wamala u mung'hya/nsumba wako?*<br><br>Darasa 1-4<br>Darasa 5<br>(*zungushia moja kwa kila mpenzi*) Darasa 6<br>Darasa 7<br>Hakuhudhuria shule ya msingi<br>Sifahamu/Sikumbuki	4<br>5<br>6<br>7<br>8<br>9	4<br>5<br>6<br>7<br>8<br>9	4<br>5<br>6<br>7<br>8<br>9
5.07	Ni shughuli gani kuu (shughuli au kazi) mpenzi huyo hufanya/amefanya katika miezi 12 iliyopita?<br>*Milimo ki iyo mung'hya wako/nsumba wako akitaga/witile u mu myeji 12 iyo yabitile?*<br>***Usimtajie majibu msailiwa, lakini kila baada ya jibu, uliza, "Asante. Kitu kingine?"(kwa kila mpenzi veka vema linalo husika)***<br>Mfanyakazi Mgodini- Kazi ya ujuzi (Uhandisi, Uhasibu, surveya n.k.)<br>Mfanyakazi Mgodini- Kazi isiyo ya ujuzi (kibarua, Ulinzi, dereva)<br>Kazi ya ujuzi (eg Mwalimu, Muuguzi, Muhasibu, Polisi)<br>Kazi nyingine isiyo ya ujuzi (k.m seremala/ fundi cherehani)<br>Biashara kubwa (eg mmiliki wa duka; gereji, baa & gesti, Shamba kubwa la kilimo cha biashara)<br>Biashara ndogo ndogo (mf: vibanda na biashara za magengeni jioni, Mama lishe, mpika pombe)<br>Mhudumu wa Baa, Mtumishi wa gesti, Mtumishi wa hoteli<br>Dereva<br>Tingo wa lori<br>Mvuvi<br>Mkulima<br>Mama wa nyumbani<br>Mtumishi wa kazi za ndani (nyumbani)<br>Yupo chuo au shule<br>Hapana shughuli<br>Nyingine (taja..): Sifahamu<br>Mpenzi 1: ______<br>Mpenzi 2: ______<br>Mpenzi 3: ______	☐ (×16)	☐ (×16)	☐ (×16)
5.08	Je, ni muda gani umepita tangu ulipofanya mapenzi na mtu huyu kwa <u>mara ya kwanza</u>?<br><br>*Makanza ki gabitile kwingila aho ukilala nu mun'hu ng'wenuyo <u>lukangala lwa ng'wisho?</u>*<br>*Ingiza kama mshiriki anavyochagua kujibu: Onyesha kipimo (Siku, Wiki, Mwezi, Mwaka) kwenye kisanduku cha mwisho k.m.'003M'=miezi mitatu;* '060D'=siku sitini; '012W'=wiki kumi na mbili; *'999x'=NK*	NNN<br>unit U	NNN<br>unit U	NNN<br>unit U

5.09	Je, ni muda gani umepita tangu ulipofanya mapenzi na mtu huyu kwa mara ya mwisho?<br><br>*Makanza ki gabitile kwingila aho ukilala na mun'hu ng'wenuyo lukangala lwa ng'wisho?*<br>*Ingiza kama mshiriki anavyochagua kujibu: Onyesha kipimo (Siku, Wiki, Mwezi, Mwaka) kwenye kisanduku cha mwisho k.m.'003M'=miezi mitatu;* '060D'=siku sitini; '012W'=wiki kumi na mbili; '999x'=NK	NNN unit U	NNN unit U	NNN unit U
5.10	Je, ni mara ngapi ulifanya mapenzi na mtu huyo katika wiki nne zilizopita?<br>*Ng'hangala jinga ukilala na mun'hu ngwenuyo umu boyo bune uwabitile?*<br>(Ingiza namba - tarakimu mbili) AU 99=NK	NN	NN	NN
5.11	Je, hali ya uhusiano wenu wa kimapenzi na mtu huyo kwa sasa ikoje?<br>*Akikalile ka witogwa wing'we kali kinehe ihaha?*			
	Bado tuna mahusiano na tutaendelea kufanya mapenzi tena	1	1	1
	Hatuendelei na uhusiano, lakini tunaweza kufanya mapenzi tena	2	2	2
	Uhusiano wetu umevunjika kabisa	3	3	3
	Sifahamu	9	9	9
5.12	Je, mlitumia njia yoyote ya uzazi wa mpango kuzuia mimba mara ya mwisho ulipofanya mapenzi na mtu huyo?<br><br>*Mkatumila nzila yose yose ya kuzuia nda aho wilalaga lukangala lwa ng'wisho n'umun'u ng'wenuyo?* (*zungushia moja kwa kila mpenzi*)			
	Ndiyo	1	1	1
	Hapana	2	2	2
	Kama jibu ni "NDIYO" → Je, ulitumia kitu gani kuzuia mimba?<br>→ *Ukatumila kyi kuzuia nda?*<br>*Usimtajie majibu msailiwa, lakini kila baada ya jibu, uliza, "Asante. Kitu kingine?" (Weka vema kwa yote yanayotumika)*			
	Kondom	✓	✓	✓
	Vidonge vya uzazi wa majira	✓	✓	✓
	Sindano za uzazi wa majira	✓	✓	✓
	Kupima joto	✓	✓	✓
	Madawa ya kienyeji	✓	✓	✓
	Kutoa uume kwenye uke kabla ya kutoa shahawa	✓	✓	✓
	Kuosha uke baada ya kujamiana	✓	✓	✓
	Sifahamu	✓	✓	✓
	Nyingine (taja..):<br>Mpenzi 1: ____________________<br>Mpenzi 2:____________________<br>Mpenzi 3:____________________			

5.13	Je, ulitumia kondomu ulipofanya mapenzi mara ya mwisho na mtu huyo?<br>Kama NDIYO→<br>Je, ulitumia kondomu kwa matendo yote wakati mnafanya mapenzi?<br>(Mulianza tendo la kwanza hadi la mwisho mkitumia kondomu)<br><br>*Ukatumila kondomu ulukangala lwa ng'wisho aho wilalaga n'umun'hu ng'wenuyo?*<br>*Ulu Ehe→Mkatumila kondomu ng'hangala jose aho ng'wilalaga? (Mukatumila kondomu kwingila hawandijo mpaga ng'wisho)?*<br><br>Kama HAPANA→ Zungushia namba 3 kwa mpenzi husika<br><br>*(zungushia moja kwa kila mpenzi)*<br>Ndiyo, tulitumia kondom kwa muda/matendo yote→q.5.15<br>Ndiyo lakini siyo kwa matendo yote→q.5.14<br>Hapana→q.5.14	1<br>2<br>3		1<br>2<br>3		1<br>2<br>3
Kama jibu la swali namba 5.13 ni HAPANA, SIKUTUMIA KONDOM au NDIYO LAKINI SIYO MUDA WOTE uliza swali la 5.14 chini. Kama jibu ni NDIYO nenda swali la 5.15.						
5.14	Kwa nini wewe na mpenzi wako hamkutumia kondomu kwa wakati huo? Au Kwa nini wewe na mpenzi wako hamkutumia kondomu kwa matendo yote?<br>*Ni buli ubebe n'umung'hya/nsumba wako mutatumamilie kondomu ilikanza lyenilo?*<br>*Usimtajie majibu msailiwa, lakini kila baada ya jibu, uliza, "Asante. Kitu kingine?"*<br>(Weka vema kwa yote yanayotumika)<br>Sifahamu zinapopatikana<br>Ni ghali mno<br>Mpenzi alikataa<br>Hatuzipendi-zinapunguza raha<br>Hatujui namna ya kuzitumia<br>Kimaadili ni mbaya/kinyume na dini<br>Tulitumia kinga nyingine ya mimba<br>Sina wapenzi wengi<br>Namuamini mpenzi wangu<br>Hatukuifikiria/tulisahau<br>Tulitaka kupata mimba<br>Kondom ilipasuka<br>Sifahamu<br><br>Nyingine (taja..):<br>Mpenzi 1: ________________<br>Mpenzi 2: ________________<br>Mpenzi 3: ________________	✓<br>✓<br>✓<br>✓<br>✓<br>✓<br>✓<br>✓<br>✓<br>✓<br>✓<br>✓<br>✓		✓<br>✓<br>✓<br>✓<br>✓<br>✓<br>✓<br>✓<br>✓<br>✓<br>✓<br>✓<br>✓		✓<br>✓<br>✓<br>✓<br>✓<br>✓<br>✓<br>✓<br>✓<br>✓<br>✓<br>✓<br>✓

5.15	Je, imewahi kukutokea usijisikie kufanya mapenzi na huyo mpenzi wako wakati yeye anajisikia/anataka kufanyamapenzi?<br><br>*Lwakwilongela nulu lushiku lumo aho ukikumva kwilala n'umung'hya/nsumba wako ng'wenuyo?*					
	Ndiyo	1		1		1
	Hapana	2		2		2
	Sikumbuki	9		9		9
	Kama NDIYO: Je, ulifanya nini mara ya mwisho ilipotokea hali hiyo?<br>*Lukangala lwa ng'wisho aho yakwilongela yeniyo ukita ki?*					
	(zungushia moja kwa kila mpenzi) Nilikataa na hatukufanya mapenzi	1		1		1
	Nilikataa lakini bado tukafanya mapenzi	2		2		2
	Sikukataa	3		3		3
	Sifahamu	9		9		9

6. Uzuiaji mimba	(ijazwe na msahili mkuu)	
6.01	Sasa napenda ukumbuke watu uliowahi kufanyanao mapenzi katika muda wote wa maisha yako. Hii ina maana toka ulipofanya mapenzi kwa mara ya kwanza hadi mara ya mwisho hivi karibuni na inajumuisha watu wote mf: mpenzi wa sasa, wapenzi wa zamani, mchumba au mme/mke na watu wengine). Ni vizuri utumie muda unaokutosha ili uweze kukumbuka suala hili kwa usahihi<br><br>Je, katika kipindi chote cha maisha yako ni jumla ya watu wangapi umefanyanao mapenzi?<br><br>*Ihaha nditogwa ubizukwe aban'hu abo wilala nabo u mumaisha gako gose, hu kuhaya kwingila lushiku lwa kwandya kwilala na mun'hu mpaga mun'hu uyo wailalile nang'hwe lukangala lwa ng'wisho, (abose abo wilala nabo giti, mun'ghya/nsumba wa haha, wa kale, ngoshi/nke wako na ban'hu bangi). Ikubelela utumile makanza gose gose kusudi wizuke chiza mhayo gwenuyu.*<br><br>*U mumakanza gose ga bulamu wako wilala na ban'hu bamnga?*<br><br>*Kama mshiriki hawezi kukumbuka namba kamili, tafadhari muombe ajaribu kukadiria idadi ya namba.*	Ingiza namba (tarakimu 2)
Swali la 6.02 – 6.04 NI KWA WANAWAKE TU (WANAUME NENDA SWALI LA 6.05)		
6.02	<wanawake> Ni mara ngapi umekuwa mjamzito?<br><br>*Wabiza budito ng'hangala jinga?*	Ingiza namba (tarakimu 2)<br>au 99 = kama idadi haifahamiki
6.03	<wanawake> Ni mwaka gani wa shule ulipata ujauzito kwa mara ya kwanza?<br><br>*Nanali ukabiza nda mu shule lukangala lwa kwandya?*<br><br>(Zungushia jibu moja)	Siyo shuleni/nilikuwa nimeacha shule 2<br>Darasa la 3 3<br>Darasa la 4 4<br>Darasa la 5 5<br>Darasa 6 6<br>Darasa la 7 7<br>Haistahili 8<br>Sifamu/Sikumbukii 9
6.04	<wanawake> Wakati mwingine msichana hupata ujauzito bila kupanga kuwa hivyo na wakati ambapo siyo muda mzuri.<br><br>*Makanza gangi ng'waniki akabyagi budito bila kupanga kubi giko niyo mu makanza gabi ulu ali mushule*<br><br>Je, uliwahi kuwa mjamzito bila ya kuwa umepanga kuwa hivyo?<br><br>Wagema kubiza budito bila kupanga kubi giko?	Ndiyo 1<br>Hapana 2<br>Sifamu /Sikumbuki 9

<table>
<tr><th colspan="3">Swali la 6.05 – 6.07 ni kwa WANAUME TU (WANAWAKE NENDA Swali la 6.08)</th></tr>
<tr><td>6.05</td><td><wanaume> Ni mara ngapi umewapa wasichana au wanawake ujauzito?

Ng'hangala jinga wabagita nda baniki nulu bakhima?

Kama jibu ni HAPANA jaza "00" kasha nenda → 6.08</td><td>Ingiza namba (tarakimu 2)
au 99 = kama idadi haifahamiki</td></tr>
<tr><td>6.06</td><td><wanaume> Ilikuwa mwaka gani shule ya msingi ulipompa msichana mimba kwa mara ya kwanza?

(Zungushia jibu moja)</td><td>Siyo shuleni/nilikuwa nimeacha shule 2
Darasa la 3 3
Darasa la 4 4
Darasa la 5 5
Darasa la 6 6
Darasa la 7 7
Haistahili 8
Sifahamu/Sikumbuki 9</td></tr>
<tr><td>6.07</td><td><wanaume> Wakati mwingine msichana hupata ujauzito bila kupanga kuwa hivyo na wakati ambapo siyo muda mzuri.

Makanza gangi ng'waniki akabyagi budito bila kupanga kubi giko niyo mu makanza gabi ulu ali mushule

Je, uliwahi kumpa msichana ujauzito bila kupanga kufanya hivyo?
Wangita nda ng'waniki bila kupanga giko? (Zungushia jibu moja)</td><td>Ndiyo 1
Hapana 2
Sifamu /Sikumbuki 9</td></tr>
<tr><td>6.08</td><td>< wanawake NA wanaume >

Je, umekwishawahi kutumia njia yoyote ya kuzuia mimba wakati ulipokuwa ukifanya mapenzi?

Wagema kutumila nzila yose yose utizubiza nda aho ng'wilalaga?

Kama jibu ni "NDIYO"→ Ni njia zipi za kuzuia mimba umekwisha wahi kutumia?

Ulu ishosho lili "EHE" → Nzila ki ijowamala kutumia utizupandika nda?

Usimtajie majibu msailiwa, lakini kila baada ya jibu, uliza, "Asante. Kitu kingine?"

(Weka vema kwa yote yanayotumika)</td><td>Ndiyo 1
Hapana 2→ q 7.01
Sifahamu 9→ q 7.01

Kondom
Vidonge vya uzazi wa majira
Sindano za uzazi wa majira
Kupima joto
Madawa ya kienyeji
Kutoa uume kwenye uke kabla ya kutoa shahawa
Kuosha uke baada ya kujamiana (kuingiza kidole ukeni)
Nyingine (Bainisha) ______________________</td></tr>
</table>

7. KUOLEWA / KUOA

Sasa nakwenda kuuliza maswali kidogo kuhusu uzoefu wako katika ndoa. Mara zote ninapo taja neno ndoa ninamaanisha kuishi pamoja na mtu kama mme na mke. Hii inajumuisha kuishi pamoja kama mme na mke hata bila ya kufunga ndoa rasmi; Kimada; nyumba ndogo au kuwa katika ukewenza. Tunafahamu kwamba baadhi ya vijana kama wewe wamekwisha wahi kuolewa/ kuoa mara moja, wengine zaidi ya mara moja na baadhi hawajawahi kuoa/kuolewa. Tunacho hitaji zaidi ni kusikia ukweli juu ya uzoefu wa vijana katika ndoa. Kumbuka kwamba mazungumzo haya ni siri kubwa kati yako na yangu, kwa hiyo natumaini utakuwa huru kunieleza juu ya uzoefu wako wa ndoa katika maisha.

Ihaha naja kukubuja mabujo magehu kuhusu bumani wako wa mambo ga witoji. Ng'hangala jose uli nuyomba 'witoji' ndina maana ya kwigasha/kwikala na mun'hu giti ngoshi na nke wakwe. Yeniyi ili kihamo na mun'hu na nke nulu batinafunga ndoa; Kimada; Kaya/numba ndo nulu kutolwa mhali. Tumanile giki baadhi ya basumba/bang'hya giti bebe tayari batolwa/batola lukangala lumo, bangi zaidi ya lukangala lumo na bangi batali kutola/kutolwa. Tulitogwa sana bukweli juu ya bumani wa basumba na bang'hya wa mambo ga witoji. Izukagwa giki mahoya aya gali ga mbisila sana kuli bebe na nene. Hi giko lulu ndiganika ukubiza na wiyabi wa kuniwila kuhusu bumani wako kuhusu mambo ga witoji u mumaisha.

7.01	Je, kwa ujumla umeolewa/ umeoa mara ngapi AU umeishi na mtu kama mke/mme mara ngapi?<br>*Watolwa/watola ngh'angala jinga nulu wigasha na mun'hu na nke/ngoshi wakwe ngh'angala jinga*<br>*Ingiza '00' kama hajawahi kuolewa/kuoa AU kuishi na mtu kama mke/mme nenda swali la 7.08*		*Ingiza namba (tarakimu 2) au 99 = hafahamu*
7.02	Ulikuwa na umri gani ulipoolewa/oa AU ulipoishi na mwanaume/mwanamke kama uliyeolewa / uliyeoa kwa <u>mara ya kwanza</u>?<br>*Wali na myaka yinga aho ulitolwa/ulitola NULU aho uligasha na ngosha/nkima giti utolilwe/utolile <u>lukangala lwa kwandya?</u>*<br>*Ingiza umri wa kuolewa/kuoa kwa mara ya kwanza* *miaka*<br>*Kama namba halisi haifahamiki, kadilia (tick)*<br>miaka 12 au pungufu 44 miaka 13-14 55 miaka 15-17 66 miaka 18 au zaidi 77 NK 99		
7.03	<Mwanaume>	Je, kwa sasa Umeoa?<br>*Ihaha utolile?*<br>*Kama "HAPANA" Nenda swali la 7.08*	*(Zungushia moja)* Ndiyo 1<br>Hapana 2 → q7.08
7.04	<Mwanamke>	Je, kwa sasa Umeolewa?<br>*Ihaha utolilwe?*<br>*Kama "HAPANA" Nenda swali la 7.08*	*(Zungushia moja)* Ndiyo 1<br>Hapana 2 → q7.08
7.05	<Mwanaume>	Unao wake/wapenzi wangapi unaoishi nao? Chukulia kwa sasa, siyo hapo kabla<br>*Uli na bakae/bang'hya banga abo ukigashaga nabo? Ihah'iyi, iti kale*	*Ingiza namba (tarakimu 2) au 99 = hafahamu*
7.06	<Mwanamke>	Ni wake wangapi, ukiwemo na wewe, mme wako/mpenzi wako unayeishi naye anao?<br>*Bakima banga kihamo n'ubebe abo ngoshi wako/nsumba wako alinabo?*	*Ingiza namba (tarakimu 2) au 99 = hafahamu*
7.07	Je, unaishi na mke/mme wako?<br>*Uligasha na ng'witogwabitogwa bako?*<br>***KAMA "NDIYO" nenda swali la 8***		*(Zungushia moja)* Ndiyo 1→ q8.01<br>Hapana 2
7.08	Je, unaishi na mpenzi/wapenzi?<br>*Uligasha na ng'witogwa/bitogwa bako?*		*(Zungushia moja)* Ndiyo 1<br>Hapana 2

8. Uzoefu wa Magonjwa ya Ngono

8.01	Je, umewahi kutokwa na usaha au maji maji yasiyokuwa ya kawaida kwenye sehemu zako za siri katika miezi 12 iliyopita?<br>*Wagema kwinga buhila nulu minzi minzi agati ga kawaida kwinga ku wimambele u myeji 12 iyo yabitile?*	*(Zungushia moja)*	Ndiyo<br>Hapana<br>Sifahamu	1<br>2<br>9
8.02	Umewahi kuwa na vidonda au malengelenge kwenye sehemu zako za siri katika miezi 12 iliyopita?<br>*Wagema kubiza na buhele nulu malengelenge ku wimambele u mumyeji 12 iyabitile?*	*(Zungushia moja)*	Ndiyo<br>Hapana<br>Sifahamu	1<br>2<br>9
Kama Hapana, au sifahamu kwa maswali yote Q8.1 na 8.2 basi nenda swali la 8.06				
8.03	Je, Ulifanya lolote kati ya haya yafuatayo mara ya mwisho (muda wa karibuni kabisa) baada ya kuwa na kidonda/lengelenge kwenye sehemu zako za siri AU usaha/majimaji yasiyo ya kawaida yakitoka kwenye sehemu zako za siri?<br>*Ukita shose shose ayo gali aha lukangala lwa ng'wisho (makanza aga hah'iyi gete) aho wabiza na nh'ondo/ilengelenge kuwima mbele wako NULU buhila/minzi minzi ayo gati ga kawaida kufuma kuwima mbele wako)*<br>*SOMA CHAGUO ZA MAJIBU KWA SAUTI, kisha weka vema kwa yote yaliyo kubalika*		Kujitibu mwenyewe (nyumbani kwa dawa za kienyeji)<br>Kujitibu mwenyewe (nyumbani kwa dawa za hospitali)<br>Kutafuta ushauri/dawa toka hospitali au kituo cha afya cha serikali<br>Kutafuta ushauri/dawa toka hospitali au kituo cha afya cha binafsi<br>Kutafuta ushauri/dawa toka kwa mganga wa kienyeji<br>Hapana kati ya haya	 <br>→ q 8.05<br>→ q 8.05
8.04	Ni sababu zipi zilizokufanya usiende hospitali/ kituo cha afya?<br>*Nguno ki ijo jikakwenheleja ukije kuja kushibitali/ituo sha afya?*<br>*Usimtajie majibu msailiwa, lakini kila baada ya jibu, uliza, "Asante. Kitu kingine?"*<br>*Utizung'wila mashosho umujiwa, kwike buli ishosho, mujage, "Wabeja. Kin'hu ki shingi?"*<br>*(weka vema kwa yote yaliyo kubalika) (Tulaga vema ku gose agalizunilijiwa)*		Sijazoea kwenda hospitali<br>Ni mbali mno<br>Gharama ni kubwa mno<br>Matibabu ni duni/siyo mazuri<br>Hakuna usiri<br>Watumishi ni wakali<br>Inakera/Aibu<br>Nyingine (Bainisha): ________________	→ q 8.06<br>→ q 8.06<br>→ q 8.06<br>→ q 8.06<br>→ q 8.06<br>→ q 8.06<br>→ q 8.06<br>→ q 8.06

8.05	Ni muda gani baada ya kuona dalili ulikwenda hospitali au kituo cha afya kupata huduma?<br>*Makanza ki baada ya kubona dalili jenijo ukaja kushibitali nulu ku kituo sha afya kujulagulwa?*<br>*(Zungushia moja)*	Mara moja (siku1 hadi 2) 1<br>Siku 3 hadi 4 2<br>Siku 5 hadi 6 3<br>Wiki moja na kuendelea 4<br>Haistahili 8<br>Sifahamu 9
8.06	Umewahi kuongezewa damu ndani ya miaka 5 iliyopita?<br>*Wagema kungejiwa mininga u mu myaka 5 iyabitile*<br>*(Zungushia moja)*	Ndiyo 1<br>Hapana 2<br>Sifahamu 9
8.07	Umekwisha chomwa sindano ngapi ndani ya miezi 12 iliyopita?<br>*Waching'wa shinge jinga u mu myeji 12 iyabitile?*	*Ingiza namba (tarakimu 2)*<br>*au 99 = hafahamu*
8.08	Tunafahamu kuwa baadhi ya wanaume/wanawake katika eneo hili wametahiriwa au kukeketwa<br><Wanaume>. Je, wewe umetahiriwa?<br><Wanawake> Je, wewe umekeketwa?<br>*Tumanille giki baadhi ya bagosha/bakima u mumaeneo ging'we bagekwa. Kinehe ubebe wagekwa?*<br>Kama jibu ni HAPANA au SIFAHAMU nenda swali la 9<br>*(Zungushia moja)*	Ndiyo 1<br>Hapana 2⟶ q 9.01<br>Sifahamu 9⟶ q 9.01
8.09	*Kama ndiyo*<br><Wanaume> Je, ulikuwa na umri gani wakati unatahiliwa?<br><Wanawake> Je, ulikuwa na umri gani ulipokeketwa?<br>*Ulu "EHE" wali na myaka yinga aho uligekwa?*	miaka<br>Ingiza namba (tarakimu 2)<br>*AU 99=Hafahamu*

**9. Vipindi au mafunzo ya MEMA kwa Vijana shuleni**		(ijazwe na msahili mkuu)		
Sasa nakwenda kukuuliza kuhusu miaka yako michache ya mwisho katika shule ya msingi<br>*Ihaha ndihaya kukubuja kuhusu myaka yako migehu ya ng'wisho mu shule ya msingi*				
9.01	Umewahi kuhudhuria kipindi cha MEMA ulipokuwa shule ya msingi?<br>*Wagema kuja mu bulangwa wa MEMA aho wali mushule ya msingi?*	*(Zungushia moja)*	Ndiyo<br>Hapana<br>Sifahamu	1<br>2<br>9
9.02	Darasa lenu shule ya msingi mliwahi kwenda kutembelea hospitali/kituo cha afya au zahanati?<br>*Idalasa ling'we lyagema kuja na ng'walimu wing'we kuyelela shibitali/kituo sha afya nulu zahanati?*	*(Zungushia moja)*	Ndiyo<br>Hapana<br>Sifahamu	1<br>2<br>9
9.03	Mfanyakazi wa afya amewahi kutembelea darasa lenu Shule ya msingi?<br>*Ntumami wa afya wagema kuyelela idalasa ling'we ha shule ya msingi?*	*(Zungushia moja)*	Ndiyo<br>Hapana<br>Sifahamu	1<br>2<br>9
9.04	Kuna Mwalimu wenu yeyote aliwahi kuzungumza na darasa lenu kuhusu kuzuia/kukataa shinikizo la kufanya mapenzi?<br>*Aliho ng'walimu wing'we wose wose uyo wagema kuhoya n'idalasa ling'we kuhusu kulemejiwa/kuhadikijiwa kwilala?*	*(Zungushia moja)*	Ndiyo<br>Hapana<br>Sifahamu	1<br>2<br>9
9.05	Mwalimu wenu aliwahi kuzungumza na darasa lenu kuhusu matumizi ya kondomu kujikinga na VVU?<br>*Ng'walimu wing'we wagema kuhoya n'idalasa ling'we juu ya butumiji wa kondomu kwilinda na BUKIMWI?*	*(Zungushia moja)*	Ndiyo<br>Hapana<br>Sifahamu	1<br>2<br>9
9.06	Je, wewe ulikuwa muelimisha rika wa MEMA kwa Vijana katika darasa lenu?<br>*Bebe wali ng'welemishaji rika wa MEMA kwa Vijana u ng'widalasa ling'we?*	*(Zungushia moja)*	Ndiyo<br>Hapana<br>Sifahamu	1<br>2<br>9

Wasailiwa wote waelekezwe kwenda Maabara

10. LAB SPECIMENS		(completed by LAB WORKERS)
10.01	Lab workers' staff codes	
10.02	Umekojoa kwa mara mwisho saa ngapi? (Swahili time)	: Morning Afternoon Evening (circle one)
10.03	Time urine container given to respondent (Swahili time)	: Morning Afternoon Evening (circle one)
10.04	Number of serum aliquots taken<br>If <3, specify reason: ______	
10.05	Number of urine aliquots taken *[take 2 samples]*	If 2 samples taken → q. 10.07
10.06	Why were < 2 urine aliquots taken? *(Circle one)*	No urine 1<br>Insufficient urine 2<br>Refused 3<br>Other (specify): ______ 4
10.07	RBC result *(Circle one)*	negative 0<br>+ 1<br>+ + 2<br>+ + + 3<br>+ + + + 4

Weka stika hapa	
kama anastahili:	*FS_ _ _ _ _ _*
kama hastahili:	*XFS_ _ _ _ _ _*

Sehemu C

11. CLINICAL

(completed by CLINICIANS)

11.01	Clinician's staff code		
	< Males > skip to q 11.03		
11.02	< Females> Are you pregnant? *(Circle one)*	Yes 1<br>No 2<br>Don't know 9	
	< Females > Only examine genitals if report symptoms of genital ulcers (GUS)<br>< Males > Examine genitals of all males		
		SYMPTOMS	EXAM
11.03	Do you have either pus or an abnormal discharge coming from your private parts (vagina or penis) at the moment (GDS)?	Yes 1<br>No 2<br>NK 9	Yes, seen 1<br>No, not seen 2<br>Refused exam 7<br>Not examined 8
11.04	Type of discharge (women only)	Curdlike 1<br>Non-curdlike 2<br>Not applicable 8	
11.05	Do you have an ulcer or sore or blister on your private parts at the moment (GUS)?	Yes 1<br>No 2<br>NK 9	Yes, seen 1<br>No, not seen 2<br>Refused exam 7<br>Not examined 8
11.06	Do you have any genital itching at the moment?	Yes 1<br>No 2<br>NK 9	
11.07	Do you have any pain during micturation/urination?	Yes 1<br>No 2<br>NK 9	
11.08	< Males > skip to question 11.09<br>< Females > Do you have any lower abdominal / pelvic pain at the moment or pain in your abdomen when you have sex?	Yes 1<br>No 2<br>NK 9	
11.09	Do you have any genital warts at the moment?	Yes 1<br>No 2<br>NK 9	Yes, seen 1<br>No, not seen 2<br>Refused exam 7<br>Not examined 8

11.10	Do you have any lumps in the groin at the moment?	Yes 1<br>No 2<br>NK 9	Yes, seen 1<br>No, not seen 2<br>Refused exam 7<br>Not examined 8
11.11	< Females > skip to question 11.12<br><br>< Males > Examine for circumcision		Circumcised 1<br>Not circumcised 2<br>Refused exam 7<br>Not examined 8

11.12	DIAGNOSES				
		diagnosed	treated	referred	refused
	Tick all that apply None				
	GUS (men or women)				
	Genital blisters or vescicles				
	VDS without curd-like discharge (women only)				
	VDS with curd-like discharge (women only)				
	PID (women only)				
	Urethral discharge (men only)				
	Epididymo-orchitis (men only)				
	Bubo				
	Pubic lice				
	Scabies				
	Warts				
	Signs of secondary syphilis				
	Symptoms of malaria				
	Schistosomiasis				
	Cough				
	Headache				
	Other (specify): ____________________<br>________________________________				

	<MALE> If participant needs treatment then complete Q11.16, if no treatment needed then end interview<br><FEMALE> If needs treatment for GUS, VDS, PID, Bubo or Schistosomiasis then please ask Q.11.13<br>If treatment not needed for these conditions then you may proceed to Q11.16 and provide other treatment.
11.13	Is this participant visibly pregnant? Yes 1 → q11.16; No 2 → q11.14
11.14	When was your last menstrual period?<br>(99 / 99 / 2009 if unknown)<br>IF MORE THAN 1 MONTH SINCE LMP THEN TEST FOR PREGNANCY / /200
11.15	Result of Pregnancy Test (Circle one only)
	Positive 1 Negative 2 Refused 3 Not Done / Not applicable 8

11.16	TREATMENT GIVEN		*tick if given*
**GUS**			
GUS 1st line treatment		Benz. Penicillin 2.4 MU, im stat ½ in each buttock	1 ✓
		Cotrimoxazole 400/80 mg, 5 tabs bid for 3 days	2 ✓
GUS 1st line treatment if allergic to penicillin		Erythromycin 500mg qid for 15 days	3 ✓
		Cotrimoxazole 400/80 mg, 5 tabs bid for 3 days	4 ✓
GUS 1st line treatment if pregnant		Benz. Penicillin 2.4 MU, im stat ½ in each buttock	5 ✓
		Erythromycin 500mg qid for 7 days	6 ✓
GUS 2nd line treatment		Ceftriaxone 250 mg im, stat	7 ✓
**VDS**			
VDS (No curdlike discharge) 1st line treatment		Ciprofloxacin 500 mg, stat	8 ✓
		Doxycycline 100 mg, bid for 7 days	9 ✓
		Metronidazole 2 g, stat *warn about alcohol*	10 ✓
VDS (no curdlike discharge) 1st line if in 1st trimester of pregnancy		Erythromycin 500mg qds for 7 days	11 ✓
		OR Ceftriaxone 250 mg im, stat	12 ✓
VDS (no curdlike discharge) 1st line if in 2nd or 3rd trimester of pregnancy		Erythromycin 500mg tid for 7 days	13 ✓
		OR Ceftriaxone 250 mg im, stat	14 ✓
		AND Metronidazole 2 g, stat (i.e. give with either erythomycin or cetriaxone) *warn about alcohol*	15 ✓
VDS 2nd line treatment		Clotrimazole pessaries 100 mg, OD, PV	16 ✓
		Ceftriaxone 250 mg im, stat	17 ✓
		Doxycycline 100 mg, bid for 7 days (DO NOT give if lactating OR pregnant)	18 ✓
		Metronidazole 2 g, stat *warn about alcohol* (DO NOT give if pregnant)	19 ✓
VDS 1st line if complains of curdlike discharge (add to standard 1st line treatment for VDS)	With vulval itching	Clotrimazole pessaries 100 mg, OD, PV	20 ✓
		Clotrimazole cream, 1 tube	21 ✓
	No vulval itching	Clotrimazole pessaries 100 mg, OD, PV	22 ✓

**PID**		
PID 1st line treatment	Ciprofloxacin 500 mg, stat	23 ☑
	Doxycycline 100 mg, bid for 14 days	24 ☑
	Metronidazole 400mg bid, for 14 days *warn about alcohol*	25 ☑
PID 1st line treatment if lactating mother or pregnant	Erythromycin 500mg tid for 7 days	26 ☑
	OR Ceftriaxone 250 mg im, stat	27 ☑
	AND Metronidazole 400mg bid, for 14 days *if not in 1st trimester of pregnancy warn about alcohol*	28 ☑
PID 2nd line treatment	Ceftriaxone 250 mg im, stat	29 ☑
**URETHRAL DISCHARGE**		
Urethral discharge 1st line treatment	Ciprofloxacin 500 mg, stat	30 ☑
	Doxycycline 100 mg, bid for 7 days	31 ☑
Urethral discharge 2nd line treatment	Doxycycline 100 mg, bid for 7 days	32 ☑
	Ceftriaxone 250 mg im, stat	33 ☑
	Metronidazole 2 g, stat *warn about alcohol*	34 ☑
**EPIDIDYMO-ORCHITIS**		
Painful scrotal swelling (epididymo-orchitis)	Ciprofloxacin 500 mg, stat	35 ☑
	Doxycycline 100 mg, bid for 7 days.	36 ☑
**BUBO**		
Bubo 1st line treatment	Doxycycline 100 mg, bid for 14 days	37 ☑
Bubo 1st line treatment if pregnant	Erythromycin 500mg qds for **14** days	38 ☑
**PUBIC LICE**		
Pubic lice treatment	Lindane 1% lotion or cream. Apply and wash off after 8hrs	39 ☑
	**OR** Lindane shampoo. Apply and wash off after 4 minutes	40 ☑
**BLISTERS/VESICLES**		
Blisters/vesicles	Gentian Violet paint	41 ☑
**SCABIES**		
Scabies treatment	Benzyl Benzoate 25% lotion nightly for 2 nights	42 ☑
**SKIN CONDITIONS**		
Skin conditions	Whitfield's ointment	43 ☑

PAIN RELIEF		
Analgesia	Paracetamol 500mg (no more than 6 tablets per day for 2 days) *write number of tablets given* ……………………	44 ☐
**SCHISTOSOMIASIS**		
Schistosomiasis	Praziquantel *tick here if given*	45 ☐
	Weight in kilograms	45a NNN kg
	State number of tablets given	45b N
**COUGH**		
Cough	Erythromycin 500mg, tid, 5 days	46 ☐
	Cotrimoxazole 960mg, bid, 5 days	47 ☐
**MALARIA**		
Malaria	ALU 4 st, 4 after 8 hrs, 4 bid for 2 days	48 ☐
**OTHER MEDICATIONS**		
Indication ____________	Other (specify name of drug, number of tablets and dose) ____________	49 ☐
Indication ____________	Other (specify name of drug, number of tablets and dose) ____________	50 ☐

12. VOLUNTARY HIV TESTING (completed by COUNSELLOR)

All participants should be given the following information about the voluntary HIV testing service.
As the interviewer told you earlier, all the information and the results of the tests on the urine, and blood you have given us will be kept secret. However, if you would like to know whether you are infected with HIV, there is a separate procedure to be followed. If you are interested, I can tell you about this procedure. If, after that, you want to continue, we can do a test now and I can tell you the results of your HIV test. I will also give you further advice. Nobody except you, me and the project's senior staff in Mwanza will know the results

Washiriki wote wapewe taarifa hii ya huduma ya kupima virusi vya UKIMWI kwa HIARI
Kama msaili alivyokueleza hapo awali, taarifa zote na majibu ya vipimo vya mkojo na damu tulivyochukua vitakuwa vya siri kabisa. Hata hivyo, kama unataka kujua kama umeathirika na virusi vya UKIMWI, kuna utaratibu tofauti wa kufuata, kama utapenda ninaweza kukuelezea utaratibu wa kufuata, na kama baada ya hapo utapenda kuendelea nitafanya kipimo na kukupatia majibu yako sasa hivi, na nitakupatia ushauri zaidi. Hakuna mtu zaidi yako wewe, mimi na mkuu wa mradi huko Mwanza atakayejua majibu ya kipimo chako.

12.01	Counsellor's staff code				
12.02	Do you want to be told your HIV test result?<br>*If Yes, give the pre-test counselling.*	*(Circle one)*	Yes<br>No	1<br>2	 → end
12.03	Are you satisfied with this pre-test counselling?<br>*If NO, then do not proceed with test but carry out full pre-test counselling*	*(Circle one)*	Yes<br>No	1<br>2	→ 12.05<br>→ 12.04
12.04	Are you <u>now</u> satisfied with this pre-test counselling?	*(Circle one)*	Yes<br>No	1<br>2	 → end
12.05	Do you want to be told your HIV result?	*(Circle one)*	Yes<br>No	1<br>2	 → end
	If participant wants to be told HIV result complete the Request for HIV Test Results Form, carry out the rapid HIV tests and carry out post-test counselling				
12.06	Have you completed a Request for HIV Test Results form?	*(Circle one)*	Yes<br>No	1<br>2	 → end
12.07	Have you given full post-test counselling?<br><br>If no, why was full post-test counselling not given? ________________	*(Circle one)*	Yes<br>No	1<br>2	
	If the Request form is completed and you have given full post-test counselling then give the participant their result. Provide further counselling and advice.				

MkV FS: QC Individual Questionnaire

Weka stika hapa
kama anastahili: FS_ _ _ _ _ _ _
kama hastahili: XFS_ _ _ _ _ _

Sehemu A: 1. Namba ya mshiriki (Ijazwe na msaili wa usajili)

1.01	Tarehe ya usaili (tarehe/mwezi/mwaka)	/ /200
1.02	Alama ya siri ya msaili	
1.03	Jinsia ya mshiriki (Usimuulize mshiriki)	(*Zungushia moja)*<br>Mwanaume 1<br>Mwanamke 2
1.04	Ulizaliwa tarehe gani?<br>*Ukabyalwa tarehe jinga?*<br>*Kama haufahamika (NK) ingiza 99/99/9999*<br><br>*Kama <u>tarehe kamili</u> ya kuzaliwa HAIFAHAMIKI basi uliza*<br>Una umri gani?<br>Uli na myaka yinga?<br>*Tumia kalenda ya matukio kumsaidia kijana kukumbuka umri wake katika miaka kamili.*	/ /19<br>Kama tarehe kamili inafahamika → 1.05<br><br>Umri kwa miaka<br>99= Haifahamiki
1.05	Je, katika shule ya msing ni darasi gani la juu ulisoma na kulimaliza? (Liwe ni darasa alilosoma kwa mwaka mzima)<br>*Ukasoma shule ya msingi mpaga mu kanga?*<br>*(zungushia moja)*	Darasa 1-4 4 →maliza mahojiano<br>Darasa 5 5<br>Darasa 6 6<br>Darasa 7 7<br>Sikuhudhuria shule ya msingi 8 → maliza mahojiano<br>Sifahamu/sikumbuki 9

Sehemu B (ijazwe na msaili mkuu)

2. Taarifa ya takwimu za watu

2.01	Umefikia kiwango gani cha juu cha elimu?<br>*Wasoma mpaga mu kanga?*	*(Zungushia moja)*<br>Sikumaliza shule ya msing 1<br>Nilimaliza shule ya msing 2<br>Sikumaliza shule ya sekondari 3<br>Shule ya sekondari kidato cha 4 4<br>Shule ya sekondari kidato cha 6 5<br>Mafunzo ya Ufundi 6<br>Chuo (e.g. Chuo cha ualimu, Chuo cha polisi, n.k.) 7<br>Chuo kikuu 8<br>Masomo mengine (e.g.Chuo cha kiislamu: chuo cha thiolojia) 9
2.02	Je, katika wiki nne zilizopita, umeishalala nje ya Kata yako?<br>*Umunyuma inne ijabitile walalalile hanze ya Kata yako?*	*Kama "NDIYO"*→ Siku ngapi? (Ingiza idadi ya siku)<br>*Kama "HAPANA"* INGIZA "00"<br><br>Kama NDIYO, pia zungushia 1 kwa swali la 2.03 na uliza→ Je, nimala ngapi umelala nje ya kata yako angalau usiku mmoja katika miezi 12 iliyopita?

2.03	Je katika miezi 12 iliyopita, umeishawahi kulala nje ya kata yako?<br><br>*Umu myeji ikumi n'ibili iyo yabitile, wagema nulu kamo kulala hanze ya Kata yako?*<br><br>Kama ndiyo: Je, ni mara ngapi umelala angalau usiku 1 nje ya kata yako?<br><br>Kama ndiyo: Kwa ujumla ni kipindi cha muda gani ulilala nje ya Kata yako katika kipindi cha miezi 12 iliyopita?<br><br>*Ingiza kama mshiriki anavyochagua kujibu: Onyesha kipimo (Siku, Wiki, Mwezi, Mwaka) kwenye kisanduku cha mwisho k.m.'003M'=miezi mitatu;* '060D'=siku sitini; '012W'=wiki kumi na mbili; *'999x'=NK*	*(Zungushia moja)*<br>Ndiyo 1<br>Hapana 2 *→3.01*<br>Sifahamu 9 *→3.01*<br>*Kama 'Hapana' au 'Sifahamu' nenda → 3.01*<br><br>N N N<br><br>unit U

3. Elimu ya afya ya uzazi na jinsia na tabia ya kufanya mapenzi

Sasa napenda kukuuliza maswali machache kuhusu Afya, Magojwa na kufanya mapenzi. Kila wakati ninapotaja neno kufanya mapenzi ninazungumzia kuhusu kujamiiana na mtu. Hii ni pamoja na kujamiiana ambapo mmoja kati ya wawili, hakukubali (mmoja amelazimishwa).

Ihaha nditogwa na kubuje mabujo magehu juu ya Afya, Basatu na kwilala. Buli ulu nayomba mhayo "kwilala" ndiyombela kuhusu kwilala na mun'hu. Yeniyi ili kihamo na kulala na mun'hu umo wa babili benabo atazunije (umo wabo wahadikijiwagwa).

3.01	Je, kichocho kinaweza kuambukizwa kwa kufanya mapenzi na mtu?<br><br>*Udugije kutulilwa kisambale ku lwakwilala na mun'hu?*	*(Zungushia moja)*	Ndiyo<br>Hapana<br>Sifahamu	1<br>2<br>9
3.02	Je, vidonda kwenye sehemu za siri vinaweza kuambukizwa kwa kufanya mapenzi?<br><br>*N'hondo ku wimambele jidugije kwenhelejiwa ku lwa kwilala?*	*(Zungushia moja)*	Ndiyo<br>Hapana<br>Sifahamu	1<br>2<br>9
3.03	Je, virusi vya UKIMWI vinaweza kuambukizwa kwa kufanya mapenzi?<br><br>*BUKIMWI budugije kutulilwa ku lwa kwilala?*	*(Zungushia moja)*	Ndiyo<br>Hapana<br>Sifahamu	1<br>2<br>9
3.04	Je, unaweza kuambukizwa virusi vya UKIMWI kwa kula katika sahani moja na mtu mwenye virusi vya UKIMWI?<br><br>*Udugije kutulilwa BUKIMWI ku lwa kulila musahani imo na mun'hu uyo alina BUKIMWI?*	*(Zungushia moja)*	Ndiyo<br>Hapana<br>Sifahamu	1<br>2<br>9
3.05	Je, mtu anayeonekana kuwa na nguvu na afya nzuri anaweza kuwa na virusi vya UKIMWI?<br><br>*Ulu mun'hu alibonekana alina afya ya wiza adugije kubiza na BUKIMWI?*	*(Zungushia moja)*	Ndiyo<br>Hapana<br>Sifahamu	1<br>2<br>9

Sasa napenda kukuuliza maswali machache kuhusu kondomu; Ninaposema kondomu kila mara ninamaanisha mpira wa uume unaotumika wakati wa kujamiiiana
Ihaha nditogwa nakubuje mabujo magehu kuhusu kondomu.

3.06	Je, inawezekana mtu kuzuia mimba kwa kutumia kondom wakati wa kufanya mapenzi?<br><br>*Idulikanile mun'hu kukija kubiza nda ulu utumila kondomu ikanza lya kwilala?*	*(Zungushia moja)*	Ndiyo<br>Hapana<br>Sifahamu	1<br>2<br>9

3.07	Je, baadhi ya kondomu mpya huwa na virusi vya UKIMWI kabla hazijatumiwa?<br>*Kondomu jikabyagi na BUKIMWI kuko jitinandya kutumamilwa?*	*(Zungushia moja)*	Ndiyo<br>Hapana<br>Sifahamu	1<br>2<br>9
3.08	Je, kutumia kondom kunaweza kusaidia kuzuia wanaume na wanawake kuambukizana virusi vya UKIMWI?<br>*Kutumamila kondomu kudugije kunzuia ngosha na nkima kutulilwa BUKIMWI?*	*(Zungushia moja)*	Ndiyo<br>Hapana<br>Sifahamu	1<br>2<br>9
3.09	Je, ni wapi ambapo unaweza kupata kondomu ukizihitaji kwenye kijiji chenu?<br>*Hali uko udugije kupandika kondomu ulu ulijitogwa u munzengo gwing'we?*<br>***Usimtajie majibu msailiwa, lakini kila baada ya jibu, uliza, "Asante. Sehemu nyingine?***<br>(*WEKA VEMA KWA KILA LILILO TAJWA*)		Duka/Kibanda/duka la dawa<br>Kituo cha afya<br>Muwakilishi wa shirika lisilo la kiserikali<br>Mtu binafsi mwingine<br>Nyingine (taja): ____________<br>Sifahamu	
3.10	Je, ni wapi ambapo mtu anaweza kupata kondomu za bure kijijini kwenu?<br>*Hali u munzengo gwing'we uko mun'hu adugije kupandika kondomu ja dalali?*<br>***Usimtajie majibu msailiwa, lakini kila baada ya jibu, uliza, "Asante. Kitu kingine?"***<br>*(WEKA VEMA KWA KILA LILILO TAJWA)*		Duka/Kibanda/duka la dawa<br>Kituo cha afya<br>Muwakilishi wa shirika lisilo la kiserikali<br>Mtu binafsi mwingine<br>Nyingine (taja): ____________<br>Sifahamu	

4. Tabia ya kufanya mapenzi

Sasa napenda kukuuliza tena maswali machache kuhusu kufanya mapenzi. Kama nilivyotaja awali, kila wakati ninapotaja neno kufanya mapenzi ninazungumzia kuhusu kujamiiana na mtu. Hii ni pamoja na kujamiiana ambapo mmoja kati ya wawili, hakukubali (mmoja amelazimishwa). Tunafahamu kuwa vijana kama wewe tayari wamekwishafanya mapenzi na wengine bado. Tunapenda tu kusikia ukweli kuhusu uzoefu wa vijana katika kufanya mapenzi. Majadiliano haya ni ya siri sana kati yako na mimi, hivyo natumaini kwamba utakuwa huru kuniambia kuhusu uzoefu wako katika kufanya mapenzi. Baadhi ya maswali haya yanahusu vipindi virefu vya miezi na miaka. Inaweza kuwa vigumu kukumbuka kwa haraka uzoefu kuhusu kipindi kirefu kama hicho. Cha muhimu zaidi ni kwamba ujibu maswali kama hayo kikamilifu na kwa usahihi kama iwezekanavyo. Hivyo tafadhali, chukua muda kadri unavyohitaji kufikiri kuhusu hayo wakati unapojibu.

Ihaha nditogwa na kubuje hangi mabujo magehu juu ya kwilala. Gitumo nali nayombela hawandijo, buli ulu nayomba mhayo "kwilala" ndiyombela kuhusu kwilala na mun'hu. Yeniyi ili kihamo na kulala na mun'hu umo wa babili benabo atazunije (umo wabo wahadikijiwagwa). Tumanile giki basumba/bang'hya giti bebe tayali bamala kwilala na ban'hu. Mahoya genaya gali ga mbisila sana, gali gise babili duhu, bebe na nene, giko lulu ndiganika giki ukubiza na wiyabi wa kuniwila kuhusu bumani wako wa mambo ga wilaji. Mabujo gangi umu genaya galihusu makanza malihu ga myeji na myaka. Idugije kubiza shidamu kwizukwa wangu bumani wako wa makanza malihu giti genayo. Kin'u ishitale zaidi ili giki kushosha mashosho ga mabujo giti genaya mung'hana gitumo udugigije. H'igiko lulu ndikulomba usole makanza ayo ulitogwa kwiganika yeniyo makanza ulu ulishosha.

4.01	Je, umeshawahi kufanya mapenzi na mtu yoyote?<br>*Wagema kwilala na mun'hu wose wose?*<br>*Kama jibu ni "HAPANA", Dadisi kwa kuuliza: "Je nimekuelewa vizuri, kwamba haujawahi kufanya mapenzi au kujamiana na mtu yoyote katika maisha yako yote?"*<br>*Kama bado ataendelea kusema hajawahi kufanya mapenzi: zungushia "HAPANA=2"*	*(Zungushia moja)*	Ndiyo<br>Hapana	1<br>2

Kama jibu ni bado hajawahi kufanya mapenzi nenda swali la 8.

4.02	Je, umeshawahi kutumia kondomu wakati wa kufanya mapenzi<br><br>*Wagema kutumamila kondomu ikanza aho ng'wilalaga ?*	*(Zungushia moja)* Ndiyo 1<br>Hapana 2<br>Sifahamu 9
4.03	Sasa napenda ufikirie mara ya kwanza ulipofanya mapenzi na mtu. Je, ulikuwa na umri gani wakati ulipofanya mapenzi kwa mara ya kwanza?<br><br>*Ihaha nditogwa wiganike ulukangala lwa kwandya aho ukilala na mun'hu. Wali na myaka yinga aho ulilala na mun'hu lukangala lwa kwandya?*<br><br>*(Tafadhali jaribu kumsaidia mshiriki kukumbuka umri halisi wakati alipofanya mapenzi kwa mara ya kwanza k.m. ulikuwa darasa la ngapi katika shule ya msingi n.k.)*<br><br>Ingiza miaka kamili ☐☐ miaka<br>***Kama miaka halisi haifahamiki msaidie kukadiria miaka na weka vema kwenye boksi chini***<br><br>miaka 12 au pungufu 44 miaka 13-14 55 miaka 15-17 66 18 au zaidi 77 NK 99	
4.04	Sasa, naomba ufikiri juu ya mtu uliyefanya naye mapenzi kwa mara ya kwanza. Je, mtu uliyefanya naye mapenzi kwa mara ya kwanza alikuwa na umri mkubwa kuliko wewe, sawa na umri wako au na umri mdogo kuliko wewe?<br><br>*Ihaha ndilomba ung'wizuke umun'hu uyo ukilala nang'hwe lukangala lwa kwandya. Mun'hu uyo ukilala nang'hwe lukangala lwa kwandya wali ntale kukukila, ng'wali mulenganilile nulu wali ndo kukukila ubebe?*<br><br>***Kama jibu ni "3=umri sawa" au "9=hafaham" Nenda swali la 4.06***	*(Zungushia jibu moja)*<br>Umri mkubwa 1<br>Umri mdogo 2<br>Umri sawa 3 → q 4.06<br>Hafahamu 9 → q 4.06
4.05	Je, mtu uliyefanya naye mapenzi kwa mara ya kwanza alikuwa anakuzidi au unamzidi miaka mingapi?<br><br>*Umun'hu uyo ukilala nang'hwe lukangala lwa kwandya wali akukile nulu wali unkilile myaka yinga"*<br><br>***Kama miaka halisi haifahamiki msaidie kukadiria miaka na weka vema kwenye boksi chini***<br><br>miaka 1au pungufu 44 miaka 2-4 55 miaka 5-9 66 miaka 10-14 77 miaka 15 au zaidi 88 NK 99	Miaka
4.06	Sasa, naomba ufikiri juu ya watu wote uliofanya nao mapenzi katika wiki nne zilizopita:<br>Je, ni watu wangapi umefanya nao mapenzi katika muda wa wiki nne zilizopita?<br><br>*Ihaha ndikulomba wiganike/wizukwe aban'hu bos abo ukilala nabo nyuma ine ijabitile:*<br>*Ban'hu banga abo wilalile nabo umu nyuma ine ijabitile?*	Ingiza idadi<br>(tarakim mbili)
4.07	Sasa, naomba ufikiri juu ya sehemu ulizowahi kuishi katika kipindi cha mwaka mmoja ulio pita. Kumbuka itakuwa kuanzia mwezi wa (*Taja mwezi huu)* mwaka jana hadi leo au Kipindi cha (*taja majira mf masika, kiangazi*) mwaka jana hadi leo. Tafadhari tumia muda unaositahili kukumbuka. (*Mpatie muda mfupi wa kukumbuka kama dak. 2*) Je, unakumbuka?<br>Je, sasa unaweza kukumbuka ni jumla ya watu wangapi uliofanya nao mapenzi katika kipindi cha miezi 12.<br><br>*Ihaha ndikulomba wiganike kuhusu mapandi ayo wagema kwigasha/kuzenga umu ng'waka gumo ugo gwabitile. Izukagwa ibize kwingila ng'weji (Taja mwezi huu) g'wakakezo mpaga lelo iyi nulu makanza xxxx mpaga lelo. Tumilaga makanza ayo gigelile kwizukwa. (Mpatie muda mfupi wa kukumbuka kama dak. 2) Ulizukwa?*<br>*Udugije ihaha kwizukwa ijumula ya ban'hu banga abo wilala nabo umu makanza genayo?*<br>***Angalia kama namba hii inajumuisha wapenzi wa wiki 4 zilizo pita,***<br>***Kama jibu ni 00 andika 88 kwenye swali 4.08***	Ingiza idadi<br>(tarakim mbili)<br>*Haifahamiki = 99*

4.08	Je, wangapi kati ya hawa watu uliofanya nao mapenzi katika kipindi cha mwaka mmoja uliopita walikuwa wapenzi wapya kwako? (Hii ina maana ulifanya nao mapenzi kwa mara ya kwanza katika kipindi cha mwaka mmoja).<br><br>*Banga umu ban'hu benabo abo ukilala nabo ng'waka gumo ugwabitile bali basumba/bang'hya bako bapya? (Yeniyi ili kihamo na babo abo ukilala nabo lukangalwa lwa kwandya mu makanza ga ng'waka gumo).*	Ingiza idadi (tarakim mbili)<br>*Haihusiki = 88*<br>*Haifahamiki = 99*

KAMA HAJAFANYA MAPENZI KATIKA KIPINDI CHA MWAKA MMOJA ULIOPITA, NENDA q6.01

5. Wapenzi KATIKA KIPINDI CHA MIEZI 12 ILIYO PITA (ijazwe na msahili mkuu)

*** * * *Muhimu: Jaza safu moja kwa kila mpenzi ukianza na mpenzi wa hivi karibuni sana * * * ***

Fikiria kuhusu mtu wa mwisho/wa pili toka mwisho/wa tatu toka mwisho uliyefanya naye mapenzi katika miezi kumi na mbili iliyopita. Hawa siyo lazima wawe wapenzi wapya. Unawakumbuka?

Wiganike munhu u wa ng'wisho/wa kabili kwinga wa ng'wisho/wa kadatu kwinga kung'wisho uyo ukalala nang'hwe myeji ikumi n'ibili iyashilike. Iti lazima babi basumba/bang'hya bapya. Ulibizukwa?

HAPANA→ Jaribu kumfanya akumbuke kwa kuainisha kipindi mfano: tangu Krismasi iliyopita
NDIYO → endelea

		1. Mwisho (hivi karibuni sana)				
5.01	Je, alikuwa mkeo/mumeo, mpenzi mwingine wa kudumu, mpenzi wa muda mfupi, au Mfanyabiashara ya ngono?<br>*Wali nke/ngoshi wako, mung'hya/nsumba wa shiku ningi, mung'hya/nsumba wa nshiku ngehu, nu Malahya?*<br>Mkeo/Mumeo<br>*(zungushia moja)* Mpenzi mwingine wa kudumu<br>Mpenzi wa muda mfupi<br>Mfanyabiashara ya ngono	1<br>2<br>3<br>4				
5.02	Je, unaishi naye huyu mtu? (*zungushia moja*) Ndiyo<br>*Ukigashaga n'umunhu ng'wenuyo?* Hapana	1<br>2				
5.03	Je, mtu huyu ni mkubwa, mdogo ua ana umri sawa na wako?<br>*Mun'hu ng'wenuyo ntale, ndo nulu mlenganilile?* Mkubwa<br>(*zungushia moja*) Mdogo<br>Umri sawa →q.5.05<br>Sifahamu →q.5.05	1<br>2<br>3<br>9				
5.04	Je, mtu huyo anakuzidi au una mzidi miaka mingapi?<br>*Mun'hu ng'wenuyo akukilile nulu unkilile myaka yinga?*<br>***Kama miaka halisi haifahamiki jaza namba "99"***	NN				

5.05	Mpenzi huyo alisoma shule ya msingi katika kata hii?<br>*Mung'hya/nsumba wako ng'wenuyo ali mu shule ya msingi mu Kata iyi?*<br>Ndiyo<br>(*zungushia moja*) Hapana<br>Sifahamu	 <br>1<br>2<br>9				
5.06	Je, ni darasa gani la juu shule ya msingi mpanzi wako alisoma na kumaliza?<br>*Idalasa ki ilya highulya mu shule ya msingi ilo wamala u mung'hya/nsumba wako?*<br>Darasa 1-4<br>Darasa 5<br>(*zungushia moja*) Darasa 6<br>Darasa 7<br>Hakuhudhuria shule ya msingi<br>Sifahamu/Sikumbuki	 <br>4<br>5<br>6<br>7<br>8<br>9				
5.07	Je, ni muda gani umepita tangu ulipofanya mapenzi na mtu huyu kwa <u>mara ya mwisho</u>?<br>*Makanza ki gabitile kwingila aho ukilala na mun'hu ng'wenuyo <u>lukangala lwa ng'wisho</u>?*<br>*Ingiza kama mshiriki anavyochagua kujibu: Onyesha kipimo (Siku, Wiki, Mwezi, Mwaka) kwenye kisanduku cha mwisho k.m.'003M'=miezi mitatu;* '060D'=siku sitini; '012W'=wiki kumi na mbili; *'999x'=NK*	NNN<br>unit U				
5.08	Je, ni mara ngapi ulifanya mapenzi na mtu huyo katika <u>wiki nne zilizopita</u>?<br>*Ng'hangala jinga ukilala na mun'hu ngwenuyo umu boyo bune uwabitile?*<br>(Ingiza namba - tarakimu mbili) AU 99=NK	NN				
5.09	Je, hali ya uhusiano wenu wa kimapenzi na mtu huyo kwa sasa ikoje?<br>*Akikalile ka witogwa wing'we kali kinehe ihaha?*<br>Bado tuna mahusiano na tutaendelea kufanya mapenzi tena<br>Hatuendelei na uhusiano, lakini tunaweza kufanya mapenzi tena<br>Uhusiano wetu umevunjika kabisa<br>Sifahamu	 <br>1<br>2<br>3<br>9				

5.10	Je, mlitumia njia yoyote ya uzazi wa mpango kuzuia mimba <u>mara ya mwisho</u> ulipofanya mapenzi na mtu huyo?<br><br>*Mkatumila nzila yose yose ya kuzuia nda aho wilalaga lukangala lwa ng'wisho n'umun'u ng'wenuyo?* (*zungushia moja*)<br>Ndiyo<br>Hapana<br>*Kama jibu ni "NDIYO"* → Je, ulitumia kitu gani kuzuia mimba?<br>→ *Ukatumila kyi kuzuia nda?*<br>*Usimtajie majibu msailiwa, lakini kila baada ya jibu, uliza, "Asante. Kitu kingine?" (Weka vema kwa yote yanayotumika)*<br>Kondom<br>Vidonge vya uzazi wa majira<br>Sindano za uzazi wa majira<br>Kupima joto<br>Madawa ya kienyeji<br>Kutoa uume kwenye uke kabla ya kutoa shahawa<br>Kuosha uke baada ya kujamiana<br>Sifahamu<br>Nyingine (taja..):<br>Mpenzi 1: ____________________	 <br><br><br>1<br>2<br><br><br><br><br>☑<br>☑<br>☑<br>☑<br>☑<br>☑<br>☑<br>☑					
5.11	Je, ulitumia kondomu ulipofanya mapenzi mara ya mwisho na mtu huyo?<br>Kama NDIYO→<br>Je, ulitumia kondomu kwa matendo yote wakati mnafanya mapenzi? (Mulianza tendo la kwanza hadi la mwisho mkitumia kondomu)<br><br>*Ukatumila kondomu ulukangala lwa ng'wisho aho wilalaga n'umun'hu ng'wenuyo?*<br>*Ulu Ehe→Mkatumila kondomu ng'hangala jose aho ng'wilalaga? (Mukatumila kondomu kwingila hawandijo mpaga ng'wisho)?*<br><br>Kama HAPANA→ Zungushia namba 3 kwa mpenzi husika<br><br>*(zungushia moja)*<br>Ndiyo, tulitumia kondom kwa muda/matendo yote<br>Ndiyo lakini siyo kwa matendo yote<br>Hapana	 <br><br><br><br><br><br><br><br>1<br>2<br>3					

6. Uzuiaji mimba	(ijazwe na msahili mkuu)	
6.01	Sasa napenda ukumbuke watu uliowahi kufanyanao mapenzi katika muda wote wa maisha yako. Hii ina maana toka ulipofanya mapenzi kwa mara ya kwanza hadi mara ya mwisho hivi karibuni na inajumuisha watu wote mf: mpenzi wa sasa, wapenzi wa zamani, mchumba au mme/mke na watu wengine). Ni vizuri utumie muda unaokutosha ili uweze kukumbuka suala hili kwa usahihi<br><br>Je, katika kipindi chote cha maisha yako ni jumla ya watu wangapi umefanyanao mapenzi?<br><br>*Ihaha nditogwa ubizukwe aban'hu abo wilala nabo u mumaisha gako gose, hu kuhaya kwingila lushiku lwa kwandya kwilala na mun'hu mpaga mun'hu uyo wailalile nang'hwe lukangala lwa ng'wisho, (abose abo wilala nabo giti, mun'ghya/nsumba wa haha, wa kale, ngoshi/nke wako na ban'hu bangi). Ikubelela utumile makanza gose gose kusudi wizuke chiza mhayo gwenuyu.*<br>*U mumakanza gose ga bulamu wako wilala na ban'hu bamnga?*<br>*Kama mshiriki hawezi kukumbuka namba kamili, tafadhari muombe ajaribu kukadiria idadi ya namba.*	Ingiza namba (tarakimu 2)
Swali la 6.02 – 6.03 NI KWA WANAWAKE TU (WANAUME NENDA SWALI LA 6.04)		
6.02	<wanawake> Ni mara ngapi umekuwa mjamzito?<br>*Wabiza budito ng'hangala jinga?*	Ingiza namba (tarakimu 2)<br>au 99 = kama idadi haifahamiki
6.03	<wanawake> Ni mwaka gani wa shule ulipata ujauzito kwa mara ya kwanza?<br>*Nanali ukabiza nda mu shule lukangala lwa kwandya?*<br>(Zungushia jibu moja)	Siyo shuleni/nilikuwa nimeacha shule 2<br>Darasa la 3 3<br>Darasa la 4 4<br>Darasa la 5 5<br>Darasa 6 6<br>Darasa la 7 7<br>Haistahili 8<br>Sifamu/Sikumbukii 9
Swali la 6.04 ni kwa WANAUME TU (WANAWAKE NENDA Swali la 6.05)		
6.04	<wanaume> Ilikuwa mwaka gani shule ya msingi ulipompa msichana mimba kwa mara ya kwanza?<br>*(Zungushia jibu moja)*	Siyo shuleni/nilikuwa nimeacha shule 2<br>Darasa la 3 3<br>Darasa la 4 4<br>Darasa la 5 5<br>Darasa la 6 6<br>Darasa la 7 7<br>Haistahili 8<br>Sifahamu/Sikumbuki 9

6.05	< wanawake NA wanaume ><br><br>Je, umekwishawahi **kutumia** njia yoyote ya kuzuia mimba wakati ulipokuwa ukifanya mapenzi?<br>*Wagema kutumila nzila yose yose utizubiza nda aho ng'wilalaga?*<br><br>*Kama jibu ni "NDIYO"*→ Ni njia zipi za kuzuia mimba umekwisha wahi kutumia?<br>Ulu ishosho lili "EHE" → Nzila ki ijowamala kutumia utizupandika nda?<br>*Usimtajie majibu msailiwa, lakini kila baada ya jibu, uliza, "Asante. Kitu kingine?"*<br>(Weka vema kwa yote yanayotumika)	Ndiyo 1<br>Hapana 2→ q 7.01<br>Sifahamu 9→ q 7.01<br><br>Kondom<br>Vidonge vya uzazi wa majira<br>Sindano za uzazi wa majira<br>Kupima joto<br>Madawa ya kienyeji<br>Kutoa uume kwenye uke kabla ya kutoa shahawa<br>Kuosha uke baada ya kujamiana (kuingiza kidole ukeni)<br>Nyingine (Bainisha) ________________

7. KUOLEWA / KUOA

Sasa nakwenda kuuliza maswali kidogo kuhusu uzoefu wako katika ndoa. Mara zote ninapo taja neno ndoa ninamaanisha kuishi pamoja na mtu kama mme na mke. Hii inajumuisha kuishi pamoja kama mme na mke hata bila ya kufunga ndoa rasmi; Kimada; nyumba ndogo au kuwa katika ukewenza. Tunafahamu kwamba baadhi ya vijana kama wewe wamekwisha wahi kuolewa/ kuoa mara moja, wengine zaidi ya mara moja na baadhi hawajawahi kuoa/kuolewa. Tunacho hitaji zaidi ni kusikia ukweli juu ya uzoefu wa vijana katika ndoa. Kumbuka kwamba mazungumzo haya ni siri kubwa kati yako na yangu, kwa hiyo natumaini utakuwa huru kunieleza juu ya uzoefu wako wa ndoa katika maisha.

Ihaha naja kukubuja mabujo magehu kuhusu bumani wako wa mambo ga witoji. Ng'hangala jose uli nuyomba 'witoji' ndina maana ya kwigasha/kwikala na mun'hu giti ngoshi na nke wakwe. Yeniyi ili kihamo na mun'hu na nke nulu batinafunga ndoa; Kimada; Kaya/numba ndo nulu kutolwa mhali. Tumanile giki baadhi ya basumba/bang'hya giti bebe tayari batolwa/batola lukangala lumo, bangi zaidi ya lukangala lumo na bangi batali kutola/kutolwa. Tulitogwa sana bukweli juu ya bumani wa basumba na bang'hya wa mambo ga witoji. Izukagwa giki mahoya aya gali ga mbisila sana kuli bebe na nene. Hi giko lulu ndiganika ukubiza na wiyabi wa kuniwila kuhusu bumani wako kuhusu mambo ga witoji u mumaisha.

7.01	Je, kwa ujumla umeolewa/ umeoa mara ngapi AU umeishi na mtu kama mke/mme mara ngapi?<br>*Watolwa/watola ngh'angala jinga nulu wigasha na mun'hu na nke/ngoshi wakwe ngh'angala jinga*<br>*Ingiza '00' kama hajawahi kuolewa/kuoa AU kuishi na mtu kama mke/mme nenda swali la 7.08*	*Ingiza namba (tarakimu 2) au 99 = hafahamu*
7.02	Ulikuwa na umri gani ulipoolewa/oa AU ulipoishi na mwanaume/mwanamke kama uliyeolewa / uliyeoa kwa <u>mara ya kwanza</u>?<br>*Wali na myaka yinga aho ulitolwa/ulitola NULU aho uligasha na ngosha/nkima giti utolilwe/utolile <u>lukangala lwa kwandya?</u>*<br>*Ingiza umri wa kuolewa/kuoa kwa mara ya kwanza* *miaka*<br>*Kama namba halisi haifahamiki, kadilia (tick)*<br>miaka 12 au pungufu 44 miaka 13-14 55 miaka 15-17 66 miaka 18 au zaidi 77 NK 99	

7.03	<Mwanaume>	Je, kwa sasa Umeoa?<br>*Ihaha utolile?*<br>*Kama "HAPANA" Nenda swali la 7.08*	*(Zungushia moja)* Ndiyo 1<br>Hapana 2 → q7.08
7.04	<Mwanamke>	Je, kwa sasa Umeolewa?<br>*Ihaha utolilwe?*<br>*Kama "HAPANA" Nenda swali la 7.08*	*(Zungushia moja)* Ndiyo 1<br>Hapana 2 → q7.08
7.05	<Mwanaume>	Unao wake/wapenzi wangapi unaoishi nao? Chukulia kwa sasa, siyo hapo kabla<br>*Uli na bakae/bang'hya banga abo ukigashaga nabo? Ihah'iyi, iti kale*	*Ingiza namba (tarakimu 2) au 99 = hafahamu*
7.06	<Mwanamke>	Ni wake wangapi, ukiwemo na wewe, mme wako/mpenzi wako unayeishi naye anao?<br>*Bakima banga kihamo n'ubebe abo ngoshi wako/nsumba wako alinabo?*	*Ingiza namba (tarakimu 2) au 99 = hafahamu*
7.07	Je, unaishi na mke/mme wako?<br>*Uligasha na ng'witogwabitogwa bako?*<br>*KAMA "NDIYO" nenda swali la 8*		*(Zungushia moja)* Ndiyo 1→ q8.01<br>Hapana 2
7.08	Je, unaishi na mpenzi/wapenzi?<br>*Uligasha na ng'witogwa/bitogwa bako?*		*(Zungushia moja)* Ndiyo 1<br>Hapana 2

8. Vipindi au mafunzo ya MEMA kwa Vijana shuleni (ijazwe na msahili mkuu)

Sasa nakwenda kukuuliza kuhusu miaka yako michache ya mwisho katika shule ya msingi

Ihaha ndihaya kukubuja kuhusu myaka yako migehu ya ng'wisho mu shule ya msingi

8.01	Umewahi kuhudhuria kipindi cha MEMA ulipokuwa shule ya msingi?<br>*Wagema kuja mu bulangwa wa MEMA aho wali mushule ya msingi?*	*(Zungushia moja)* Ndiyo 1<br>Hapana 2<br>Sifahamu 9
8.02	Mwalimu wenu aliwahi kuzungumza na darasa lenu kuhusu matumizi ya kondomu kujikinga na VVU?<br>*Ng'walimu wing'we wagema kuhoya n'idalasa ling'we juu ya butumiji wa kondomu kwilinda na BUKIMWI?*	*(Zungushia moja)* Ndiyo 1<br>Hapana 2<br>Sifahamu 9

MEMA kwa Vijana Trial Further Survey (2007-2008)

MKV1 FS Packing List: Mobilisation Officer

NO	ITEM	DETAILS	No. required 1 cmty	No. remaining from last cmty	No. to be requested	No. taken to the field	Comment	CHECKED BY…………..<br><br>Date…………
**1**	**Documents:**							
1.1	Copies of Permission letter from district authorities		3 / ward					
1.2	MKV1 FS information sheet for officials (Swahili version)		100					
1.3	MKV1 FS information sheet for HH (Swahili version)		4000					
1.3	MKV1 FS information sheet for HH (Swahili version)		4000					
1.4	Forms of lists of HH head		150					
1.5	List of HH head additional sheet		50					
1.6	Ward DC presentation		30					
1.7	Motorcycle related documents (ownership card)		Original + 1 copy					
1.8	Copy of survey permit		2					
1.9	Survey activities schedule		1					
1.10	MkV1 FS Protocol		1					

MEMA kwa Vijana Trial Further Survey (2007-2008)

NO	ITEM	DETAILS	No. required 1 cmty	No. remaining from last cmty	No. to be requested	No. taken to the field	Comment	CHECKED BY…………..<br><br>Date…………
1.11	MO Standard Operating Procedures (S.O.P)		1					
1.12	Draft letter from Ward to Village leaders		20					
1.13	Mobilisation report guide and form		3					
1.14	List of wards, villages and schools		2					
1.15	Payment vouchers		20					
1.16	Copy of Contract form for household rent		20					
1.17	ID card		1					
1.18	Driving license		Original + photocopy					
**2**	**Equipments:**							
2.1	Safety set for motorcycle driving (helmet, coat, boot, glove)		1					
2.2	MkV1 FS T-shirt		1					
2.3	Rain coat		1					
2.4	Gum boots		1					
2.5	Water-proof carrying bag		1					
2.6	Clear bag		4					
2.7	Bed sheets (pair)		1					

MEMA kwa Vijana Trial Further Survey (2007-2008)

NO	ITEM	DETAILS	No. required 1 cmty	No. remaining from last cmty	No. to be requested	No. taken to the field	Comment	CHECKED BY…………. Date…………
2.8	Torch		1					
2.9	Batteries		4					
2.10	Mosquito net (Treated net)		1					
2.11	First Aid Kit		1					
2.12	Note book		1					
2.13	Biro blue		50					
2.14	A4 envelopes		50					
2.15	Clip Board		1					
2.16	Plain A4 paper		250					
2.17	Stapler		1					
2.18	Staples (big box)		½					
2.19	Carbon paper		50					
2.20	Calculator		1					
2.21	Ruler		1					
2.22	Plastic bag		50					

MKV1 FS: Field packing list for Census Team

Team______________________ Communitie(s)_______________________

S/N	Item	Details	No. Required for 1 cmty	**No. Required for 2 cmty**	**No. remaining from last cmty**	**No. to be requested**	**No. taken to field**	**Comment**	CHECKED BY……………….. Date……………..
1	Documents:								
1.1	Census timetable	Specific for that community	11	22					
1.2	Copy of ethical approval		2	2					
1.3	Copy of official letter from regional, district & ward authority		3 letters	6 letters					
1.4	Codes for ward, villages, schools		11 copies	11 copies					
1.5	Master Lists of HH head (ORIGINAL TO BE LEFT IN MWANZA)	per kitongoji in specific community	2copies	2 copies					
1.6	CI form for HH head		300 copies	600 copies					
1.7	Information sheet for officials / community members		25 copies	50 copies					
1.8	Information sheet for HH		3500 copies	7000 copies					
1.9	HH consent form		200 copies	400 copies					
1.10	Invitation letter for eligible young people		1000 copies	2000 copies					
1.11	Back-up Census questionnaire		1000 copies	1000 copies					

S/N	Item	Details	No. Required for 1 cmty	**No. Required for 2 cmty**	**No. remaining from last cmty**	**No. to be requested**	**No. taken to field**	**Comment**	CHECKED BY……………….. Date………………
1.12	Back-up Census Q Add		1000 copies	1000 copies					
1.13	List of Back-up Census ID numbers		2 copies	2 copies					
1.14	Census standard operation procedures (SOP)		11 copies	11 copies					
1.15	Payment forms for CH/WEO-WEC and others		50 copies	100 copies					
1.16	Identity Card		11	11					
1.17	Diary		1	1					
1.18	List of staff codes		2	2					
1.19	Calendar of events		11	11					
1.20	Village Information Form		10	20					
1.21	Information sheet for parent/guardian		250	500					
1.22	Consent form for parent/guardian		250	500					
1.23	Moved away form		100	200					
1.24	PDA borrowing list		40	80					
1.25	Census Daily Progressive Report		40	80					
1.26	Census village summary form		10	20					
1.27	Census team community report		2	4					

S/N	Item	Details	No. Required for 1 cmty	**No. Required for 2 cmty**	**No. remaining from last cmty**	**No. to be requested**	**No. taken to field**	**Comment**	CHECKED BY……………….. Date………………
2	Equipment:								
2.1	Mobile phone + charger		1	1					
2.2	PDAs		11	11					
2.3	PDA chargers		11	11					
2.4	PDA hard cases		11	11					
2.5	PDA synchronization cable		2	2					
2.6	GPS		11	11					
2.7	GPS data transfer cable		2	2					
2.8	Batteries for GPS (AA)		80	160					
2.9	Laptop (including 6-cell battery and laptop power adaptor)		1	1					
2.10	Spare laptop battery (12-cell)		1	1					
2.11	Laptop Surge protector		1	1					
2.12	Laptop skin bag		1	1					
2.13	Laptop waterproof carrying bag		1	1					
2.14	Flash drive (512MB)		1	1					
2.15	CD ROM	To back-up data	30	60					
2.16	Printer	338HP	1	1					
2.17	Printer spare cartridges	338HP	2	4					
2.18	Extension cable		3	3					
2.19	Solar charger + connector	To charge PDA	4	4					

S/N	Item	Details	No. Required for 1 cmty	**No. Required for 2 cmty**	**No. remaining from last cmty**	**No. to be requested**	**No. taken to field**	**Comment**	CHECKED BY……………….. Date………………
2.20	Belkin AC anywhere	To charge laptop/PDA	1	1					
2.21	Palm car charger (PDAs)	To charge PDA	2	2					
2.22	Torch		11	11					
2.23	Energizer battery charger	To charge batteries for torch	1	1					
2.24	Rechargeable batteries	For torches only	22 (OR 40 non-rech)	22 (OR 80 non-rech)					
2.25	Bicycles + 2 spare tubes+ pump		4	4					
2.26	Clip boards	To carry documents	11	11					
2.27	Hardback Note books	To record problems	11	11					
2.28	Ball point pens		35 pens	70 pens					
2.29	Padlock	For locking tin trunks & equipment in guesthouse	4	4					
2.30	Table lamp + bulb		1	1					
2.31	Ream paper		1	1					
2.32	Permanent maker pen		1pkt (2-3)	1pkt (2-3)					
2.33	Stapler		11	11					
2.34	Stapler pin		30 small boxes	60 small boxes					
2.35	Paper folders		4	4					
2.36	Punch machine		1	1					

S/N	Item	Details	No. Required for 1 cmty	**No. Required for 2 cmty**	**No. remaining from last cmty**	**No. to be requested**	**No. taken to field**	**Comment**	CHECKED BY……………….. Date………………
2.37	Tin trunk	one must be v strong for packing PDA/GPS	1	1					
2.38	Sticker note		1pkt	2pkt					
2.39	Ink pad		11	11					
2.40	Rulers		11	11					
2.41	Ink		2 bottle	2 bottle					
2.42	Envelope (A4)		20	40					
2.43	Calculator		1	1					
2.44	Box file/ Ring binders		2	2					
3	Personal Supplies								
3.1	Bed sheets (2 per person)		22	22					
3.2	Stove (Kerosene)		2	2					
3.3	Stove (Charcoal) big size		2	2					
3.4	Cooking pots (Sufuria) big size		2	2					
3.5	Cooking pots (Sufuria) medium size		2	2					
3.6	Pot lids (Mifuniko ya Sufuria) No. 4 & 5		4	4					
3.7	Basin (for washing utensils)		2	2					
3.8	Small basin		3	3					
3.9	Tea mugs		15	15					
3.10	Plates (hard plastic)		15	15					
3.11	Tablespoons		15	15					

S/N	Item	Details	No. Required for 1 cmty	**No. Required for 2 cmty**	**No. remaining from last cmty**	**No. to be requested**	**No. taken to field**	**Comment**	CHECKED BY……………….. Date………………
3.12	Teaspoons		15	15					
3.13	Metal glasses		15	15					
3.14	Serving spoons		3	3					
3.15	Wooden spoons		3	3					
3.16	Knife		2	2					
3.17	Hotpots (big)		2	2					
3.19	Tea sieve (Chujio la chai)		2	2					
3.20	Sugar container		2	2					
3.21	Plastic jugs		4	4					
3.22	Thermos flask		2	2					
3.23	Buckets (small)		1 big, 4 small	1 big, 4 small					
3.24	Water jerry cans		5	5					
3.25	Kerosene jerry cans (20 lts)		1	1					
3.26	Frying pans		1	1					
3.27	Chapati board (Kibao cha chapati)		1	1					
3.28	Tray (sinia)		3	3					
3.29	Maize flour sieve		1	1					
3.30	Ungo		2	2					
3.31	Mosquito net		11	11					
3.32	T-shirts		11	11					
3.33	Caps		11	11					
3.34	String		2 rolls	2 rolls					
3.35	Matresses		0-6 depends on cmty	0-6 depends on cmty					
3.36	Dettol soap		11 soaps	22 soaps					

S/N	Item	Details	No. Required for 1 cmty	**No. Required for 2 cmty**	**No. remaining from last cmty**	**No. to be requested**	**No. taken to field**	**Comment**	CHECKED BY……………….. Date………………
3.37	Lanterns		2	2					
3.38	First Aid kit (plasters; diclofenac (60 tabs for 2 cmtys); paracetamol (50 for 2 cmtys); Iodine; cotton wool; ALU (2 doses))		1	1					
3.39	Tarpaulin		1	1					
3.40	Gum boots		11	11					
3.41	Rain coat		11	11					
3.42	Umbrella		11pcs	11pcs					
3.43	Bag for carrying PDA and docs		10	10					
3.44	Small bowls		11	11					

MEMA kwa Vijana Trial Further Survey (2007-2008)

MKV 1 FS: PACKING LIST FOR REGISTRAR/INTERVIEWERS/TRACERS

NO	ITEM	DETAILS	No. Required for 1 cmty	No. Required for 2 cmty	No. remaining from last cmty	No. requested	No. taken to field	Comment	CHECKED NAME: DATE:
1	SOPs		13	13					
2	Time table		13	13					
3	Staff ID card		13	13					
4	MkV1FS protocol		13	13					
5	Calendar of events		13	13					
6	Hard cover note book for error recording	For error recording	13	13					
7	Post-it-maker	For brief note	13	26					
8	Blue biros		100	150					
9	Maker pens	For making equipment	4	4					
10	Ruler		13	13					
11	MkV1FS T-shirts		13	13					
12	Clipboard		13	13					
13	Registration books		2	2					
14	Stickers for part (FS…)		1000	2000					
15	Stickers for non-part (XFS…)		250	500					
16	Information sheet for participants		1000	2000					
17	Consent forms for participant		1000	2000					
18	List of codes for ward, village and schools (list B)		7	7					
19	MkV 1 list of names (list C)		1	1					

MEMA kwa Vijana Trial Further Survey (2007-2008)

NO	ITEM	DETAILS	No. Required for 1 cmty	No. Required for 2 cmty	No. remaining from last cmty	No. requested	No. taken to field	Comment	CHECKED NAME: DATE:
20	Standard 7 exam results (list D)	For all 4 districts	1	1					
21	Informed consent checks questions		4	4					
22	MkV1FS Main Questionnaire-section A		1500	3000					
23	MkV1FS Main Questionnaire-section B		1000	2000					
24	MkV1FS Main Questionnaire-section C		1500	3000					
25	MkV1FS QC Questionnaire		100	200					
26	Magazines/ newspapers		25	25					
27	Playing cards		5 packs	5 packs					
28	Draughts		3 sets	3 sets					
29	Radio cassette player		1	1					
30	Batteries for radio		2 sets	4 sets					
31	Scissors	For cutting stickers	2	2					
32	Ring binder	For filing documents	2	2					
33	Big empty box	For quests	2	2					
34	Panasonic walkman		6	6					
35	Information tapes (recorded one)		6	6					
36	Rechargeable battery for walkman (size "AA")		24	24					

MEMA kwa Vijana Trial Further Survey (2007-2008)

NO	ITEM	DETAILS	No. Required for 1 cmty	No. Required for 2 cmty	No. remaining from last cmty	No. requested	No. taken to field	Comment	CHECKED NAME: DATE:
37	Battery charger	For walkman batteries	2	2					
38	Extension cable	For charging batteries	1	1					
39	Stamp pad		1	1					
40	Stapler -small		1	1					
41	Stapler- heavy duty		1	1					
42	Staples (big boxes)		2	4					
42	Sellotape (roll)	For stickers	1	2					
43	Sellotape Dispensor		1	1					
44	Table clock		1	1					
45	Tarpaulin	For Registration	1	1					
46	Hole punch		1	1					
47	File folders	For keeping forms each will be labeled type of form in	10	10					
48	Plastic bags	For client soaps	1000	2000					
49	Pad lock	For locking tin trunk	2	2					
50	Tin trunk medium size	For keeping documents	1	1					
51	Highlighter pens		4	8					
52	Dust bin		1	1					

MEMA kwa Vijana Trial Further Survey (2007-2008)

53	Small towel for dusting tables		2	2					
54	Waterproof carrying bags		5	5					
55	Gum Boots		5	5					
56	Rain Coats		5	5					
57	MkV1FS Caps		5	5					
58	Umbrella		5	5					
59	Moved away form		100	200					

MEMA kwa Vijana Trial Further Survey (2007-2008)

MKV1 FS Packing List: Team Leader

NO	ITEM	DETAILS	No. required 1 cmty	No. required 2 cmty	No. remaining from last cmty	No. to be requested	No. taken to the field	Comment	CHECKED BY…………… Date…………
**1**	**Documents:**								
1.1	SOPs		1	1					
1.2	ID Card		1	1					
1.3	Survey Time table								
1.4	Payment vouchers form		100	200					
1.5	PROTOC0L INCLUDING COPIES OF ALL FORMS	For reference	1	1					
**2**	**Equipments:**								
2.1	Padlock	For locking equipment in a room	3	3					
2.2	Tin trunk (small size)	For keeping documents	1	1					
2.3	Cardboard folders	For documents	2	2					
2.4	Nokia Mobile Phone	Communication	1	1					
2.5	Calculator	For calculation	1	1					
2.6	Highlighter	Highlighting important note	1 set	1 set					
2.7	Bag	For carrying documents	1	1					
2.8	Diaries	For planning	1	1					
2.9	Extension cable	For backup	1	1					

MEMA kwa Vijana Trial Further Survey (2007-2008)

**2**	**Equipments:**								
2.10	Holepunch		1	1					
2.11	Paper glue	For pasting receits etc	1 bottle	1 bottle					
2.12	Marker pens		2	2					
2.13	Paper ream	For all team members	1	1					
2.14	Ring binder (small)		1	1					
2.15	Stamp pad	For back up	1	1					
2.16	Stamp pad ink	For back up	1 bottle	1 bottle					
2.17	Staples (big box)		1 box	2 box					
2.18	Stapler machine		1	1					
2.19	Correction fluid		1	1					
2.20	Eraser		1	1					
2.21	Pencil		2	2					
2.22	Blue biros		10	20					
2.23	Red biros		2	2					
2.24	Rulers	For drawing lines and one back up	2	2					

MEMA kwa Vijana Trial Further Survey (2007-2008)

MkV 1 FS: PACKING LIST FOR CLINICIAN

NO	ITEM	DETAILS	NO. / PART	No. required for 1 cmty	No. required for 2 cmty	No. remaining from last cmty	No. requested	NO. TAKEN TO FIELD	Comment	CHECKED BY NAME: DATE:
1	MkV1FS Protocol			1	1					
2	ID card			1	1					
3	SOP			1	1					
4	PEP reports forms			2	2					
5	Patient information sheet (PEP)			2	2					
6	HIV Test results forms (PEP)			2	2					
7	Policy on PEP form			2	2					
8	Contact referral slip	For referring of contacts to dispensary	1	200	400					
9	Counter book (4 quire)	For drug and clients record		1	1					
10	Blue biros			6	12					
11	Red biros			2	2					
12	Ruler			1	1					
13	Marker pen (thick)			1	1					
14	Post-it-maker tabs			1	2					
15	Clipboard			1	1					
16	PEP starter kit	PEP		1	1					
17	Doxycillin	For STI treatment	14 tabs	6000	12000					
18	Ciprofloxacillin	-do-	1 tab	4000	8000					
19	Metronidazole	-do-	10 tabs	4000	8000					

MEMA kwa Vijana Trial Further Survey (2007-2008)

NO	ITEM	DETAILS	NO. / PART	No. required for 1 cmty	No. required for 2 cmty	No. remaining from last cmty	No. requested	NO. TAKEN TO FIELD	Comment	CHECKED BY NAME: DATE:
20	Erythromycin	-do-	56 tabs	4000	8000					
21	Benzathine Penicillin	-do-	1 vial	10 vials	20					
22	Ceftriaxone Injection	-do-	1 vial	10 vials	20					
23	Clotrimazole V/P	-do-	1 box of 6 tabs/woman	200 boxes	400 boxes					
24	Clotrimazole Cream	-do-	1 tube	50 tubes	100 tubes					
25	ALU	(STAFF ONLY)	24 (adult dose)	15 doses	30 doses					
26	Paracetamol	-do-	18 tabs	3000	6000					
27	Prazquantel Tabs	-do-	3-4 tabs	1000	2000					
28	Adrenalin Inj.	In case of drug reaction	1	10 ampules	10 ampules					
29	Hydrocortisone Inj.	-do-	1	10 ampules	10 ampules					
30	Piriton tabs	-do-	10	1000	2000					
31	Piriton inj.	-do-	1	20 inj.	40 inj					
32	Prednisolone tabs	-do-	10	1000	2000					
33	BBE	For scabies	20 mills	500 mls	500 mls					
34	Septrin (Cotrimoxazole)	Rx of GUS etc	30 tabs	3000	6000					
35	Water for inj.	Dilution of injectables	1	30	60					
36	Tincture Iodine (bottle)			3	6					
37	Lignocane (bottle)			2	4					
38	Buscopan			1000	2000					
39	Magnesium			500	1000					

MEMA kwa Vijana Trial Further Survey (2007-2008)

NO	ITEM	DETAILS	NO. / PART	No. required for 1 cmty	No. required for 2 cmty	No. remaining from last cmty	No. requested	NO. TAKEN TO FIELD	Comment	CHECKED BY NAME: DATE:
40	Eusol	Washing wounds		5	10					
41	Whitefield			20	35					
42	Pregnancy TEST	For pregnancy TEST		100	200					
43	Syringes and needles		1	20	40					
44	Sutures			24	48					
45	Mediswabs		1	200	400					
46	Sharp Bins		1	1	2					
47	Small bucket	Hand washing water			1					
48	Gloves (pairs)		1 pair	1000	2000					
49	Small jug	for washing hands		1	1					
50	Small basin			1	1					
51	Scissors			1	1					
52	Forceps			1	1					
53	Buckets	For waste disposal	1	1	1					
54	Plastic bags	For packing an individuals drugs	3	1000	2000					
55	Plastic sheet	For female exam	1	1	1					
56	Green sheet	For covering	1	1	1					
57	Weighing scale	During treatment of SCHST	1	1	1					

MEMA kwa Vijana Trial Further Survey (2007-2008)

NO	ITEM	DETAILS	NO. / PART	No. required for 1 cmty	No. required for 2 cmty	No. remaining from last cmty	No. requested	NO. TAKEN TO FIELD	Comment	CHECKED BY NAME: DATE:
58	Digital Thermometer	Measurement of body temperature	1	2	2					
59	Disposable Thermometer cover	Covers for digital thermometer		100	200					
60	Condoms	Part of STI management	3=1pkt; 1 box= 24 pkt; 1 carton= 8 boxes	3 cartons	6 cartons					
61	Urine containers	For taking urine to clients.		50	100					
62	Small tissue hand towel	Drying hands after procedure		85 towels	170 towels					
63	Medicated hand soap	For washing hands		2	4					
64	Swab container	For swabs		1	1					
65	Tin trunk	For carrying EQ	1 medium size<br>1 large size	2	2					
66	Tall examination lamp			1	1					
67	Bulb for examination lamp (screw)			2	4					
68	Padlock			3	3					
69	Extension cable (2m)			1	1					
70	Extension cable (10m)			1	1					
71	Screen			1	1					

MEMA kwa Vijana Trial Further Survey (2007-2008)

MKV 1 FS: PACKING LIST FOR COUNSELLORS

NO	ITEM	DETAILS	No. required for 1 cmty	No. required for 2 cmty	No. remaining from last cmty	No. requested	NO. TAKEN TO FIELD	Comment	CHECKED NAME: DATE:
1	MkV1FS Protocol		2	2					
2	Timetable		2	2					
3	ID cards		2	2					
4	SOP	For following procedure	2	2					
5	Referral form for HIV positive client		100	100					
6	HIV results form	2 clients/form	600	1200					
7	HIV record book		2	2					
8	HIV test request Consent forms		600	1200					
9	Blue biros		6	12					
10	Rulers		2	2					
11	Record book hard cover (small A5)	For error recording	2	2					
12	Stamp pad	For consent	2	2					
13	File Folders	For keeping forms in field	8	8					
14	Ring binder	For filling documents	2	2					
15	Post –it- maker tabs	For short note	2	4					
16	Clip board		2	2					
17	Bioline and chaser buffer	For testing HIV	600	1200					
18	Determine test		600	1200					
19	Determine buffer		6	12					
20	Yellow tips		100	200					
21	Pipette gilsen	For tips connection	2	2					

MEMA kwa Vijana Trial Further Survey (2007-2008)

NO	ITEM	DETAILS	No. required for 1 cmty	No. required for 2 cmty	No. remaining from last cmty	No. requested	NO. TAKEN TO FIELD	Comment	CHECKED NAME: DATE:
22	Capillary Tubes		1200	2400					
23	Lancet	For finger prick	600	1200					
24	Container for yellow tips		2	2					
25	Vacutainer tubes		100	200					
26	Vacutainer holders		100	200					
27	Vacutainer needles		100	200					
28	Syringe and needles 10mls (1 box)		10	20					
29	Digital timers	For timing while testing	2	2					
30	Spare batteries for digital timer		4	8					
31	Gloves	For hand protection	1200	2400					
32	Torniquets	For easy vein protection	2 M + 2 L	2 M + 2 L					
33	Tissue (kitchen roll)	Wiping hand after touching	4 rolls	8 rolls					
34	Condom (male)	For demonstration and for distribution	17 cartons	34 cartons					
35	Swab container	For dry swabs	2	2					
36	Cryomaker	For labeling	4	4					
37	Table cover	Or covering table	2	2					
38	Penile Model	For showing proper use of condom	2	2					
39	Sharps bin	For disposing	2	4					
40	Bucket	For disposing used cotton etc	2	2					

MEMA kwa Vijana Trial Further Survey (2007-2008)

NO	ITEM	DETAILS	No. required for 1 cmty	No. required for 2 cmty	No. remaining from last cmty	No. requested	NO. TAKEN TO FIELD	Comment	CHECKED NAME: DATE:
41	Safety glass	For eye protection	2 pairs	2					
42	Medicated soap (Deto)		4pcs	8					
43	Small basin		2	2					
44	Small jug		2	2					
45	Cool box		2	2					
46	Ice pack		8	8					
47	Tin trunk (medium size)	Putting in equipment	2	2					
48	Padlock	For locking tin trunk	2	2					

MEMA kwa Vijana Trial Further Survey (2007-2008)

MKV1 FS Packing List: Driver

NO	ITEM	DETAILS	No. required 1 cmty	No. required 2 cmty	No. remaining from last cmty	No. to be requested	No. taken to the field	Comment	CHECKED BY…………… Date…………
**1**	**Documents:**								
1.1	SOPs		1	1					
1.2	Logbook		1	1					
1.3	Registration Documents		1	1					
1.4	Drivers licence		1	1					
**2**	**Equipments:**								
2.1	Lift jack	If car stuck	1	1					
2.2	Overalls	For minor service	1	1					
2.3	Rain coat		1	1					
2.4	Gum boots	During rain	1	1					
2.5	Spade		1	1					
2.6	Tarpaulin		1	1					
2.7	First aid kit	To be kept in car	1	1					
2.8	Towing chain		1	1					
2.9	Nylon rope (manila)		1	1					
2.10	Brush		1	1					
2.11	Handtowel	For wiping vehicle	1	1					
2.12	Matchet (panga)	For cutting	1	1					
2.13	Triangle	As reflector for	1 set	1 set					

		breakdown along the road							
**2**	**Equipments:**								
2.14	Steering wheel cover	For covering steering wheel	1	1					
2.15	Blue biro		2	2					
2.16	Wheel cover (plain)	For covering wheel	1	2					

MEMA kwa Vijana Trial Further Survey (2007-2008)

MkV 1 FS: PACKING LIST FOR LAB TECHNICIANS

NO	ITEM	DETAILS	No. Required for 1 cmty	No. Required for 2 cmty	No. remaining from last cmty	No. requested	No. taken to field	Comment	CHECKED BY………………… DATE……………..
1	MkV1FS Protocol		2	2					
2	Sample submission form.	For specimens and forms	50	100					
3	Time table		2	2					
4	ID cards	To identify staff in a community	2	2					
5	SOP for Lab Testing	For following procedure	2	2					
6	Hard cover note book	For error recording	2	2					
7	Register book (4 quire)	For recording no of samples, date, staff code, sticker no.	1	2					
8	Blue biros		6	12					
9	Red biros		2	4					
10	Ruler	For notebook	2	2					
11	Sellotape (rolls)	For sticking stickers	5	10					
12	Table cover		2	2					
13	Scissors		2	2					
14	Post it maker (pad)	For brief note	3	6					
15	Clear bag	For keeping forms	4	4					
16	Clip board	For holding forms	2	2					
17	Sellotape dispenser	For cutting sellotape	2	2					
18	Desk lamp		1	1					

MEMA kwa Vijana Trial Further Survey (2007-2008)

NO	ITEM	DETAILS	No. Required for 1 cmty	No. Required for 2 cmty	No. remaining from last cmty	No. requested	No. taken to field	Comment	CHECKED BY………………… DATE……………..
19	Bulb 60W		2	4					
20	Lab coats		4	4					
21	Safety glasses	To protect against splash	2	2					
22	Mask	1 every day	25	50					
23	Gloves **(PAIRS)**		1000	2000					
24	Swab container		1	1					
25	Cotton wool (roll)		1	1					
26	Tissue- hand towels (pack)		1	2					
27	Tissue – kitchen roll		3	6					
28	Washing soap medicated	For washing hands and towels	5	10					
29	Cyromaker	For marking cyrobox	4	4					
30	Mediswabs		1000	2000					
31	Vacutainer tube 10ml		1000	2000					
32	Vacutainer holder (disposable)		1000	2000					
33	Vacutainer needle (green)		1000	2000					
34	Syringe 10ml	if can't draw blood with vacutainer	10	20					
35	Sterile pasture pipette 1ml	For aliquoting serum and urine	2000	4000					
36	Scarp vein needle (size 23,24,25)	In case difficult to find vein	30	30					
37	Torniquet (med)		1	1					

MEMA kwa Vijana Trial Further Survey (2007-2008)

NO	ITEM	DETAILS	No. Required for 1 cmty	No. Required for 2 cmty	No. remaining from last cmty	No. requested	No. taken to field	Comment	CHECKED BY………………… DATE………………
38	Tourinquet (large)		1	1					
39	Urine container		1000	2000					
40	Urine dipstick	For testing schistosomiasis	1000	2000					
41	Buckets		1	1					
42	Disinfectant (Jik)-gallon	Shared with Clin and Couns	1	1					
43	Sharp bins		3	6					
44	Centrifuge machine		1	1					
45	Serum tube	5 / participant	5000	10000					
46	Cryobox (yellow)	For urine	25	50					
47	Cryobox (white)	For serum	50	100					
48	Plastic ice packs		8	8					
49	Freezer		2	2					
50	Extension cables		2	2					
51	Voltage Stabilizer		1	1					
52	Fridge(Thermo electric cool box)		1	1					
53	Battery charger (Dolphin)	For charging battery	1	1					
54	Battery (Lifeline)		2	2					
55	Connectors: freezers to ext battery (sets)		4	4					
56	Generator		2	2					
57	Digital multimeter	Test batteries	1	1					
58	Adjustable wrench		1	1					
59	10m cable reel	For connecting from generator	1	1					
60	Digital timer		1	1					

MEMA kwa Vijana Trial Further Survey (2007-2008)

NO	ITEM	DETAILS	No. Required for 1 cmty	No. Required for 2 cmty	No. remaining from last cmty	No. requested	No. taken to field	Comment	CHECKED BY………………… DATE……………..
61	Spare battery for digital timer		2	4					
62	Freezer thermometer		2	2					
63	Tin trunk (big)		1	1					
64	Padlocks		2	2					
65	Screw driver set	For tighting refrigerator handle	1	1					
66	Small bucket		1	1					
67	Small jug		1	1					
68	Screen		1	1					
69	Small basin		1	1					
70	Match box	For burning	12	24					
71	Kudu		1 roll	2 rolls					
72	Plastic container 20L		2	2					
*73	Petrol	For generator	20Lts	20Lts					
*74	Oil	For generator	3Lts	3Lts					
*75	Kerosene	For burning disposable							

*To purchase locally in field

MKV1 FS Packing List: ST Supplies – Personal Supplies List

NO	ITEM	DETAILS	No. required 1 cmty	No. required 2 cmty	No. remaining from last cmty	No. to be requested	No. taken to the field	Comment	CHECKED BY…………. Date…………
**1**	**Equipment**								
1.1	Lanterns		5	5					
1.2	Stove (kerosene)		2	2					
1.3	Stove (charcoal big size)		2	2					
1.4	Cooking pot (surfuria) big size		3	3					
1.5	Cooking pot (sulfiria)		3	3					
1.6	Cooking pot lids (mifuniko ya sulfuria) no.4 & 5		6	6					
1.7	Small plastic buckets 10 lts (for bathing)		10	10					
1.8	Basin for washing utensils		6	6					
1.9	Small basin (for washing hands)		6	6					
1.10	Tea mugs		24	24					
1.11	Plates (hard plastic)		24	24					
1.12	Tablespoons		24	24					
1.13	Serving Spoons		4	4					

**2**	**Equipments:**								
1.14	Wooden spoons (mwiko na upawa)		3	3					
1.15	Knife		3	3					
1.16	Pot holder (handle)		2	2					
1.17	Tea sieve (chujio la chai)		3	3					
1.18	Sugar container		4	4					
1.19	Salt container		4	4					
1.20	Plastic jugs		4	4					
1.21	Thermos flask		5	5					
1.22	Water container (60 lts)		2	2					
1.23	Buckets (20 lts)		4	4					
1.24	Water jerry cans		5	5					
1.25	Kerosene jerry cans		1	1					
1.26	Frying pans		2	2					
1.27	Chapatti board		2	2					
1.28	Sinia		4	4					
1.29	Maize flour sieve		2	2					
1.30	Battery	Purchase rechargeable batteries if torch takes AA	66	132					
1.31	Mosquito nets	Come with Ngao tablets	22	22					
1.32	Bed sheets		44	44					
1.33	Torch (AA batteries)		22	22					
1.34	Clear bags		22	22					
1.35	T-shirts		22	22					

MEMA kwa Vijana Trial Further Survey (2007-2008)

MkV 1 FS: PACKING LIST FOR STI TREATMENT

NO	ITEM	DETAILS	NO. / PART	No. required for 1 cmty	No. remaining from last cmty	No. requested	NO. TAKEN TO FIELD	Comment	CHECKED BY NAME: DATE:
	**Documents**								
1	MkV1FS Protocol			1					
2	ID card			1					
3	Permission letter (District to Ward)			2					
4	Survey Permit			2					
5	Motorcycle documents			1					
6	Timetable for activities			1					
7	List of wards, village and subvillages			1					
8	SOP			1					
9	Treatment after lab results form			60					
10	List A- names of participants to be treated (CONFIDENTIAL)			1					
11	List B- Treatment list for NG, CT and Syphilis			1					
12	List C- Tracing List								
13	PEP reports forms			2					
14	Patient information sheet (PEP)			2					
15	Policy on PEP form			2					
16	Contact referral slip			2					
17	Clipboard			1					

MEMA kwa Vijana Trial Further Survey (2007-2008)

NO	ITEM	DETAILS	NO. / PART	No. required for 1 cmty	No. remaining from last cmty	No. requested	NO. TAKEN TO FIELD	Comment	CHECKED BY NAME: DATE:
	**Equipments**								
18	Safety set for motorcycle (Helmet, coat, boots, gloves)			1					
19	Rain coat			1					
20	Gum Boot			1 pair					
21	Bed sheets			1 pair					
22	Torch			1					
23	Batteries for torch			4					
24	Mosquito net			1					
25	First Aid Kit			1					
26	Waterproof carrying bag			1					
27	Clear bag			2					
28	Clipboard			1					
29	Notebook			1					
30	Blue biros			5					
31	A4 Envelopes			20					
	**Medical Supplies**								
32	PEP starter kit	PEP		1					
33	Doxycyclin	CT treatment	14 tabs	250					
34	Ciprofloxacillin (500mg)	NG treatment	1 tab	20					
35	Benzathine Penicillin	Syphilis treatment	1 vial	20 vials					
36	Ceftriaxone Injection	NG 2nd line	1 vial	10 vials					

MEMA kwa Vijana Trial Further Survey (2007-2008)

NO	ITEM	DETAILS	NO. / PART	No. required for 1 cmty	No. remaining from last cmty	No. requested	NO. TAKEN TO FIELD	Comment	CHECKED BY NAME: DATE:
37	Erythromycin (500mg)	CT 2nd line	56 tabs (250mg/ tab)	250					
38	Adrenalin Inj.	In case of drug reaction	1	5 ampules					
39	Hydrocortisone Inj.	In case of drug reaction	1	5 ampules					
40	Piriton inj.	In case of drug reaction	1	5 ampules					
41	Gloves (pairs)			50					
42	Syringes + needles			50					
43	Water for inj.	Dilution of injectables	1	50					
44	Pregnancy TEST	For pregnancy TEST		50					
45	Mediswabs		1	50					
46	Plastic sheet	For female exam	1	1					
47	Cotton wool			1 roll					
48	Condoms	Part of STI management	3=1pkt; 1 box= 24 pkt	4 boxes					
49	Urine containers	For taking urine to clients.		30					
50	Small tissue hand towel	Drying hands after procedure		40 towels					
51	Medicated hand soap	For washing hands		1					
52	Padlock			1					

MEMA kwa Vijana Trial Further Survey (2007-2008)

MkV 1 FS: PACKING LIST FOR CLINICIAN

NO	ITEM	DETAILS	NO. / PART	No. required per team	No. requested	NO. TAKEN TO FIELD	Comment	CHECKED BY NAME: DATE:
1	MkV1FS Protocol			1				
2	ID card			1				
3	SOP			1				
4	PEP reports forms			1				
5	Patient information sheet (PEP)			1				
6	HIV Test results forms (PEP)			1				
7	Policy on PEP form			1				
8	Contact referral slip	For referring of contacts to dispensary	1	134				
9	Counter book (4 quire)	For drug and clients record		1				
10	Blue biros			2				
11	Red biros			2				
12	Ruler			1				
13	Marker pen (thick)			1				
14	Post-it-maker tabs			1				
15	Clipboard			1				
16	PEP starter kit	PEP		1				
17	Doxycillin	For STI treatment	14 tabs	4000				
18	Ciprofloxacillin	-do-	1 tab	2700				
19	Metronidazole	-do-	10 tabs	2700				

MEMA kwa Vijana Trial Further Survey (2007-2008)

NO	ITEM	DETAILS	NO. / PART	No. required per team	No. requested	NO. TAKEN TO FIELD	Comment	CHECKED BY NAME: DATE:
20	Erythromycin	-do-	56 tabs	2700				
21	Benzathine Penicillin	-do-	1 vial	7				
22	Ceftriaxone Injection	-do-	1 vial	7				
23	Clotrimazole V/P	-do-	1 box of 6 tabs/woman	135 boxes				
24	Clotrimazole Cream	-do-	1 tube	35 tubes				
25	ALU	(STAFF ONLY)	24 (adult dose)	30 doses				
26	Paracetamol	-do-	18 tabs	2000				
27	Prazquantel Tabs	-do-	3-4 tabs					
28	Adrenalin Inj.	In case of drug reaction	1	3 ampules				
29	Hydrocortisone Inj.	-do-	1	4 ampules				
30	Piriton tabs	-do-	10	700				
31	Piriton inj.	-do-	1	13 inj				
32	Prednisolone tabs	-do-	10	700				
33	BBE	For scabies	20 mills	117 mls				
34	Septrin (Cotrimoxazole)	Rx of GUS etc	30 tabs	2000				
35	Water for inj.	Dilution of injectables	1	20				
36	Tincture Iodine (bottle)			2				
37	Lignocane (bottle)			1				
38	Buscopan			700				
39	Magnesium			500				

MEMA kwa Vijana Trial Further Survey (2007-2008)

NO	ITEM	DETAILS	NO. / PART	No. required per team	No. requested	NO. TAKEN TO FIELD	Comment	CHECKED BY NAME: DATE:
40	Eusol	Washing wounds		3				
41	Whitefield			35				
42	Pregnancy TEST	For pregnancy TEST		70				
43	Syringes and needles		1	13				
44	Sutures			16				
45	Mediswabs		1	133				
46	Sharp Bins		1	1				
47	Small bucket	Hand washing water		1				
48	Gloves (pairs)		1 pair	700				
49	Small jug	for washing hands		1				
50	Small basin			1				
51	Scissors			1				
52	Forceps			1				
53	Buckets	For waste disposal	1	1				
54	Plastic bags	For packing an individuals drugs	3	700				
55	Plastic sheet	For female exam	1	1				
56	Green sheet	For covering	1	1				
57	Weighing scale	During treatment of SCHST	1	1				

MEMA kwa Vijana Trial Further Survey (2007-2008)

NO	ITEM	DETAILS	NO. / PART	No. required per team	No. requested	NO. TAKEN TO FIELD	Comment	CHECKED BY NAME: DATE:
58	Digital Thermometer	Measurement of body temperature	1	1				
59	Disposable Thermometer cover	Covers for digital thermometer		70				
60	Condoms	Part of STI management	3=1pkt; 1 box= 24 pkt; 1 carton= 8 boxes	2 cartons				
61	Urine containers	For taking urine to clients.		35				
62	Small tissue hand towel	Drying hands after procedure		57 towels				
63	Medicated hand soap	For washing hands		2				
64	Swab container	For swabs		1				
65	Tin trunk	For carrying EQ	1 medium size<br>1 large size	1				
66	Tall examination lamp			1				
67	Bulb for examination lamp (screw)			2				
68	Padlock			1				
69	Extension cable (2m)			1				
70	Extension cable (10m)			1				
71	Screen			1				

MEMA kwa Vijana Trial Further Survey (2007-2008)

MKV 1 FS: PACKING LIST FOR COUNSELLORS- MOP-UP

NO	ITEM	DETAILS	No. required per team	No. requested	NO. TAKEN TO FIELD	Comment	CHECKED NAME: DATE:
1	MkV1FS Protocol		1				
2	Timetable		1				
3	ID cards		1				
4	SOP	For following procedure	1				
5	Referral form for HIV positive client		35				
6	HIV results form	2 clients/form	300				
7	HIV record book		1				
8	HIV test request Consent forms		600				
9	Blue biros		2				
10	Rulers		1				
11	Record book hard cover (small A5)	For error recording	1				
12	Stamp pad	For consent	1				
13	File Folders	For keeping forms in field	2				
14	Ring binder	For filling documents	1				
15	Post –it- maker tabs	For short note	2				
16	Clip board		1				
17	Bioline and chaser buffer	For testing HIV	600				
18	Determine test		600				
19	Determine buffer		3				
20	Yellow tips		70				
21	Pipette gilsen	For tips connection	1				

MEMA kwa Vijana Trial Further Survey (2007-2008)

NO	ITEM	DETAILS	No. required per team	No. requested	NO. TAKEN TO FIELD	Comment	CHECKED NAME: DATE:
22	Capillary Tubes		1000				
23	Lancet	For finger prick	600				
24	Container for yellow tips		1				
25	Vacutainer tubes		70				
26	Vacutainer holders		70				
27	Vacutainer needles		70				
28	Syringe and needles 10mls (1 box)		8				
29	Digital timers	For timing while testing	1				
30	Spare batteries for digital timer		4				
31	Gloves	For hand protection	800				
32	Torniquets	For easy vein protection	1 M + 2 L				
33	Tissue (kitchen roll)	Wiping hand after touching	4 rolls				
34	Condom (male)	For demonstration and for distribution	8 cartons				
35	Swab container	For dry swabs	1				
36	Cryomaker	For labeling	2				
37	Table cover	Or covering table	1				
38	Penile Model	For showing proper use of condom	1				
39	Sharps bin	For disposing	2				
40	Bucket	For disposing used cotton etc	1				

MEMA kwa Vijana Trial Further Survey (2007-2008)

**NO**	**ITEM**	**DETAILS**	**No. required per team**	**No. requested**	**NO. TAKEN TO FIELD**	**Comment**	**CHECKED NAME: DATE:**
41	Safety glass	For eye protection	1				
42	Medicated soap (Deto)		2				
43	Small basin		1				
44	Small jug		1				
45	Cool box		1				
46	Ice pack		2				
47	Tin trunk (medium size)	Putting in equipment	1				
48	Padlock	For locking tin trunk	1				

MEMA kwa Vijana Trial Further Survey (2007-2008)

MKV1 FS Packing List: Driver for Mop- Up

NO	ITEM	DETAILS	No. required 1 cmty	No. required 2 cmty	No. remaining from last cmty	No. to be requested	No. taken to the field	Comment	CHECKED BY…………… Date…………
**1**	**Documents:**								
1.1	SOPs		1	1					
1.2	Logbook		1	1					
1.3	Registration Documents		1	1					
1.4	Drivers licence		1	1					
**2**	**Equipments:**								
2.1	Lift jack	If car stuck	1	1					
2.2	Overalls	For minor service	1	1					
2.3	Rain coat		1	1					
2.4	Gum boots	During rain	1	1					
2.5	Spade		1	1					
2.6	Tarpaulin		1	1					
2.7	First aid kit	To be kept in car	1	1					
2.8	Towing chain		1	1					
2.9	Nylon rope (manila)		1	1					
2.10	Brush		1	1					
2.11	Handtowel	For wiping vehicle	1	1					
2.12	Matchet (panga)	For cutting	1	1					
2.13	Triangle	As reflector for	1 set	1 set					

		breakdown along the road							
**2**	**Equipments:**								
2.14	Steering wheel cover	For covering steering wheel	1	1					
2.15	Blue biro		2	2					
2.16	Wheel cover (plain)	For covering wheel	1	2					

MEMA kwa Vijana Trial Further Survey (2007-2008)

MKV1FS 1 FS: PACKING LIST FOR LAB TECHNICIANS for Mop-Up

NO	ITEM	DETAILS	No. Required per team	No. requested	No. taken to field	Comment	CHECKED BY………………… DATE………………
1	MkV1FS Protocol		1				
2	Sample submission form.	For specimens and forms	37				
3	Time table		1				
4	ID cards	To identify staff in a community	1				
5	SOP for Lab Testing	For following procedure	1				
6	Hard cover note book	For error recording	1				
7	Register book (4 quire)	For recording no of samples, date, staff code, sticker no.	1				
8	Blue biros		4				
9	Red biros		1				
10	Ruler	For notebook	1				
11	Sellotape (rolls)	For sticking stickers	3				
12	Table cover		1				
13	Scissors		1				
14	Post it maker (pad)	For brief note	2				
15	Clear bag	For keeping forms	2				
16	Clip board	For holding forms	1				
17	Sellotape dispenser	For cutting sellotape	1				
18	Desk lamp		1				

MEMA kwa Vijana Trial Further Survey (2007-2008)

NO	ITEM	DETAILS	No. Required per team	No. requested	No. taken to field	Comment	CHECKED BY………………… DATE……………..
19	Bulb 60W		1				
20	Lab coats		2				
21	Safety glasses	To protect against splash	1				
22	Mask	1 every day	17				
23	Gloves **(PAIRS)**		700				
24	Swab container		1				
25	Cotton wool (roll)		1				
26	Tissue- hand towels (pack)		1				
27	Tissue – kitchen roll		2				
28	Washing soap medicated	For washing hands and towels	3				
29	Cyromaker	For marking cyrobox	1				
30	Mediswabs		700				
31	Vacutainer tube 10ml		700				
32	Vacutainer holder (disposable)		700				
33	Vacutainer needle (green)		700				
34	Syringe 10ml	if can't draw blood with vacutainer	7				
35	Sterile pasture pipette 1ml	For aliquoting serum and urine	1400				
36	Scarp vein needle (size 23,24,25)	In case difficult to find vein	10				
37	Torniquet (med)		1				

MEMA kwa Vijana Trial Further Survey (2007-2008)

NO	ITEM	DETAILS	No. Required per team	No. requested	No. taken to field	Comment	CHECKED BY………………… DATE……………..
38	Tourinquet (large)		1				
39	Urine container		700				
40	Urine dipstick	For testing schistosomiasis	700				
41	Buckets		1				
42	Disinfectant (Jik)-gallon	Shared with Clin and Couns	1				
43	Sharp bins		2				
44	Centrifuge machine		1				
45	Serum tube	5 / participant	3500				
46	Cryobox (yellow)	For urine	17				
47	Cryobox (white)	For serum	34				
48	Plastic ice packs		3				
49	Freezer		1				
50	Extension cables		1				
51	Voltage Stabilizer		1				
52	Fridge(Thermo electric cool box)		1				
53	Battery charger (Dolphin)	For charging battery	1				
54	Battery (Lifeline)		1				
55	Connectors: freezers to ext battery (sets)		1				
56	Generator		1				
57	Digital multimeter	Test batteries	1				
58	Adjustable wrench		1				
59	10m cable reel	For connecting from generator	1				
60	Digital timer		1				

MEMA kwa Vijana Trial Further Survey (2007-2008)

NO	ITEM	DETAILS	No. Required per team	No. requested	No. taken to field	Comment	CHECKED BY………………… DATE……………..
61	Spare battery for digital timer		1				
62	Freezer thermometer		1				
63	Tin trunk (big)		1				
64	Padlocks		1				
65	Screw driver set	For tighting refrigerator handle	1				
66	Small bucket		1				
67	Small jug		1				
68	Screen		1				
69	Small basin		1				
70	Match box	For burning	12				
71	Kudu		1 roll				
72	Plastic container 20L		1				
*73	Petrol	For generator	7Lts				
*74	Oil	For generator	1Lts				
*75	Kerosene	For burning disposable					

*To purchase locally in field

MEMA kwa Vijana Trial Further Survey (2007-2008)

MKV1 FS Packing List for mop- up: Mobilisation Officer

NO	ITEM	DETAILS	No. required Mobilization	No. remaining from last cmty	No. to be requested	No. taken to the field	Comment	CHECKED BY…………. Date…………
**1**	**Documents:**							
1.1	Copies of Permission letter from district authorities		3 / ward					
1.2	MKV1 FS information sheet for officials (Swahili version)		100					
1.3	Motorcycle related documents (ownership card)		Original + 1 copy					
1.4	Copy of survey permit		2					
1.5	Survey activities schedule		1					
1.6	MkV1 FS Protocol		1					
1.7	MO Standard Operating Procedures (S.O.P)		1					
1.8	Mobilisation report guide and form		3					
1.9	List of wards, villages and schools		2					
1.10	Copy of Contract form for household rent		20					
1.11	ID card		1					
1.12	Driving license		Original + photocopy					
**2**	**Equipments:**							
2.1	Safety set for motorcycle		1					

MEMA kwa Vijana Trial Further Survey (2007-2008)

	driving (helmet, coat, boot, glove)							
2.2	MkV1 FS T-shirt		1					
2.3	Rain coat		1					
2.4	Gum boots		1					
2.5	Water-proof carrying bag		1					
2.6	Clear bag		4					
2.7	Bed sheets (pair)		1					
2.8	Torch		1					
2.9	Batteries		4					
2.10	Mosquito net (Treated net)		1					
2.11	First Aid Kit		1					
2.12	Note book		1					
2.13	Biro blue		5					
2.14	A4 envelopes		50					
2.17	Stapler		1					
2.22	Plastic bag		50					

MEMA kwa Vijana Trial Further Survey (2007-2008)

MKV1 FS Packing List: Team Leader For Mop-Up

NO	ITEM	DETAILS	No. Required per team	No. To be requested	No. Taken to the field	Comment	CHECKED BY…………… Date…………
**1**	**Documents:**						
1.1	SOPs		1				
1.2	ID Card		1				
1.3	Survey Time table						
1.4	Payment vouchers form		67				
1.5	PROTOC0L INCLUDING COPIES OF ALL FORMS	For reference	1				
**2**	**Equipments:**						
2.1	Padlock	For locking equipment in a room	1				
2.2	Tin trunk (small size)	For keeping documents	1				
2.3	Cardboard folders	For documents	1				
2.4	Nokia Mobile Phone	Communication	1				
2.5	Calculator	For calculation	1				
2.6	Highlighter	Highlighting important note	6 pcs				
2.7	Bag	For carrying documents	1				
2.8	Diaries	For planning	1				
2.9	Extension cable	For backup	1				

MEMA kwa Vijana Trial Further Survey (2007-2008)

**2**	**Equipments:**						
2.10	Holepunch		1				
2.11	Paper glue	For pasting receits etc	1 bottle				
2.12	Marker pens		1				
2.13	Paper ream	For all team members	1				
2.14	Ring binder (small)		1				
2.15	Stamp pad	For back up	1				
2.16	Stamp pad ink	For back up	1 bottle				
2.17	Staples (big box)		1 box				
2.18	Stapler machine		1				
2.19	Correction fluid		1				
2.20	Eraser		1				
2.21	Pencil		1				
2.22	Blue biros		7				
2.23	Red biros		2				
2.24	Rulers	For drawing lines and one back up	1				

MEMA kwa Vijana Trial Further Survey (2007-2008)

MKV 1 FS: PACKING LIST FOR REGISTRAR/INTERVIEWERS/TRACERS- mop up

NO	ITEM	DETAILS	No. Required per team	No. requested	No. taken to field	Comment	CHECKED NAME: DATE:
1	SOPs		15				
2	Time table		11				
3	Staff ID card		11				
4	MkV1FS protocol		11				
5	Calendar of events		11				
6	Hard cover note book for error recording	For error recording	11				
7	Post-it-maker	For brief note	11				
8	Blue biros		60				
9	Maker pens	For making equipment	2				
10	Ruler		12				
11	MkV1FS T-shirts		15				
12	Clipboard		12				
13	Registration books		1				
14	Stickers for part (FS…)		700				
15	Stickers for non-part (XFS…)		100				
16	Information sheet for participants		700				
17	Consent forms for participant		700				
18	List of codes for ward, village and schools (list B)		2				
19	MkV 1 list of names (list C)		1				

MEMA kwa Vijana Trial Further Survey (2007-2008)

NO	ITEM	DETAILS	No. Required per team	No. requested	No. taken to field	Comment	CHECKED NAME: DATE:
20	Standard 7 exam results (list D)	For all 4 districts	1				
21	Informed consent checks questions		4				
22	MkV1FS Main Questionnaire-section A		700				
23	MkV1FS Main Questionnaire-section B		700				
24	MkV1FS Main Questionnaire-section C		700				
25	MkV1FS QC Questionnaire		70				
26	Magazines/ newspapers		8				
27	Playing cards		2 packs				
28	Draughts		1 sets				
29	Radio cassette player		1				
30	Batteries for radio		2 sets				
31	Scissors	For cutting stickers	1				
32	Ring binder	For filing documents	1				
33	Big empty box	For quests	1				
34	Panasonic walkman		2				
35	Information tapes (recorded one)		2				
36	Rechargeable battery for walkman (size “AA”)		12				

MEMA kwa Vijana Trial Further Survey (2007-2008)

NO	ITEM	DETAILS	No. Required for 1 cmty	No. requested	No. taken to field	Comment	CHECKED NAME: DATE:
37	Battery charger	For walkman batteries	1				
38	Extension cable	For charging batteries	1				
39	Stamp pad		1				
40	Stapler -small		1				
41	Stapler- heavy duty		1				
42	Staples (big boxes)		1				
42	Sellotape (roll)	For stickers	1				
43	Sellotape Dispensor		1				
44	Table clock		1				
45	Tarpaulin	For Registration	1				
46	Hole punch		1				
47	File folders	For keeping forms each will be labeled type of form in	3				
48	Plastic bags	For client soaps	700				
49	Pad lock	For locking tin trunk	1				
50	Tin trunk medium size	For keeping documents	1				
51	Highlighter pens		2				
52	Dust bin		1				

MEMA kwa Vijana Trial Further Survey (2007-2008)

53	Small towel for dusting tables		1				
54	Waterproof carrying bags		12				
55	Gum Boots		12				
56	Rain Coats		12				
57	MkV1FS Caps		12				
58	Umbrella		12				
59	Moved away form		70				

MEMA kwa Vijana Trial Further Survey (2007-2008)

MkV1 FS: Standard Operating Procedures for Mobilisation Officers (MO)

Preparations

1. One week before leaving for field, make sure that you have the correct and current version of both SOP and the protocol, read the carefully and follow the instructions while working/in the field.
2. Two days before travelling, collect your imprest from the Project administrator.
3. Ensure that all required field documents/letter, equipment and other supplies are well-packed 2 days before mobilisation trip.
4. One week before departure make sure that you have information on the community that you will be visiting i.e. list of villages, schools, contact details of leaders
5. One week before departure make sure that you have a provisional fieldwork plan indicating the number of days that you expect to be in that particular community. Share your plans with the Field Supervisor/ Fieldwork Manager/ Project co-ordinator at least 2 days before travelling. Ten working days expected per community (Ward).

District capital

6. On your way to the ward pass by the District capital and greet the DMO, DED and MkV2 TA (If the district capital is not on the way to the ward then make sure that you visit the District capital during the first or second day of your stay in the ward). Inform them that you are about to start mobilisation activities in the ward and remind them of the timetable for the census and survey. Discuss with the DMO the possibility that some participants will be referred to clinics/hospitals in the district for follow-up treatment of STIs and for HIV care and treatment.

Ward capital

7. Arrive in the community one day before mobilisation is due to begin and report to WEO on the same day (WEO is the first person to meet).
8. Day two, meet and introduce yourself to the WEO and other Ward officials found at the office, explain briefly the mission of the visit and provide permission letter from district authorities and information sheet for officials. (Swahili version)
9. Ask the WEO to help you to identify suitable accommodation for the census and survey teams. Record this information on the Mobilisation Report Form.

Mobilisation Forum

10. Whenever possible, ask WEO to assist in organising mobilisation forum with WDC and make sure that other targeted people (not member of WDC) are also invited (approximately 20 people). Ask WEO about the possibility of meeting with other WDC members (assist to distribute invitation letter).
11. Prior to the mobilisation forum inform WEO about rates of seating allowance (Tshs. 5,000/=) and transport cost (Tsh. 1,000/=) for those who coming very far from the Ward capital (not less than 1 hour walking distance). The forum should take approximately 4 hours.
12. Day three: The forum should take place on day 3. During the forum, greet and introduce yourself to the members of the WDC and other community representatives (forum participants). Explain the purpose of your visit and the aim, importance and timing of the MKV1 Further Survey. Briefly explain the survey procedures (census, survey). Record all participants and questions asked by participants on the Mobilisation Report Form.
13. Inform the WDC & other members about the ethical approval (TZA government and UK government) for the survey and explain how confidentiality will be maintained.

14. Briefly explain the benefit of the survey to individual (survey participants); local; national and international communities. (Remember to mention free STIs treatment).

Visits to Villages

15. Before you leave the ward capital ask the WEO to give you a letter of introduction to the village leaders. You will need 12- 16 copies of this letter to be distributed as follows: 1 copy to each Village Chairperson (VC), 1 copy to each Village Executive Officer (VEO), 1 copy to remain at Ward and 1 copy for MO (12-16 copies depending on the number of villages in that ward). You should assist the WEO in this task by providing him/her with the Draft letter from Ward to Village leaders that has been pre-prepared in Mwanza.
16. After the mobilisation forum (pm hours), visit nearest village and vitongoji. In each village and vitongoji meet with the community leaders and other influential people. Remember to bring introduction letters from the WEO. Do your best to meet in person all of the vitongoji leaders. Ask the village leader to accompany you/ introduce you to vitongoji leaders. Record all visits and important information on the Mobilisation Report Form.
17. Ask the village leaders to help you to identify a suitable location for the survey venue. Please ensure that the venue has chairs/benches enough for 60 people and water and toilet facilities. Rented houses and other building are preferable to guesthouses. Make sure that the owner signs both copies of the rental contract form- leave one copy with the owner and take one copy back to Mwanza. If there is a difficulty in identifying a suitable location in a village then please consult the Field Supervisor as soon as possible.
18. In each village ask Vitongoji leaders to list the names of household heads on the List of Household Head Form. Provide them with Information sheets (+ survey poster) for Household head and ask them to distribute these information sheets to the households in their kitongoji. Make sure that they

understand that they must indicate on the form the households with young people aged 15-30 years and the households that have received the information sheets. Tell the vitongoji leaders that you will return in two days to collect the lists. Inform them that if the task is completed that they will receive 5000 Tsh for the work (2500/- per day for 2 days work).

19. Next visit the health facilities in that village and explain that the survey may be referring some young people and/or their partners for treatment for STIs and/or other conditions. Visit any health/ welfare NGOs working in that village to explain the plans for the survey. Provide information sheets for officials.
20. On the arranged day, return to the vitongoji and collect all completed List of HH Form. If a Kitongoji leader has carried out the tasks assigned to him/her then give him/her Tsh. 5,000/= and ask the Kitongoji leader to continue helping us by mobilising the community.
21. Inform the vitongoji leaders that the census team leader will ask them to participate in house-to-house sensitisation and to help census interviewer during the census if they agree with the amount to be given to them as allowance. During this task (house-to house census) they will be given an allowance of Tsh 3,000/= per day. If the Kitongoji has more than 25 households ask Kitongoji leader to arrange one census helper (will also receive Tsh 3,000/= per day).
22. Day four to day eight: Continue visiting other villages and vitongoji (include health facilities in each village). In each village MO will spend approximately 1 day (includes visit health facilities/ NGOs).
23. Day nine: Complete remaining sections of the Mobilisation Report Form.
24. Day ten: Before leaving the community, you should visit WEO and debrief him/her about the exercise and remind him/her of the upcoming survey.
25. Day eleven: Travel back Mwanza

Back in Mwanza

26. One day after you return to Mwanza, you should meet with the FS & FM and debrief them about your visit. It is your responsibility to arrange this meeting.
27. You must submit your retirement to the NIMR office on the day after you return to Mwanza.
28. You must produce and submit the mobilisation report to the FM within 4 days of your return to Mwanza.
29. You must make 1 copy of each list of households prepared by the Vitongoji and leave the original in the MkV1 FS office and give the copy to the CTL.
30. Make 1 copy of each of the contracts for accommodation/ survey venue. Leave the original in the office and give the copy to the CTL.

Motorbike

31. You will be issued with a project motorbike for your work. You must keep proper records on the motorbike, including filling in the logbook after every journey, keeping receipts for fuel and lubricants purchased for the bike, repairs, etc.
32. You must ensure regular maintenance of the motorcycle and provide a verbal report to the Fieldwork Manager after each trip and help him to arrange the repairs.
33. The motorbike should only be used for official duties. If you are found to have been using the motorbike for private use, you will be fined TSh 50,000 and given a written warning on the first occasion, and will be fined TSh 50,000 and dismissed on the second occasion. The fines will be deducted from your monthly salary payments from the project. You must sign a letter agreeing to these terms before being issued with the motorbike.
34. You must observe traffic regulations (e.g. speed, you must wear a safety helmet and other safety gear).
35. You must also report any accidents to the Field Office immediately by:

a. Phoning the office (AD, KM, LM or the Project Accountants).

b. Sending a written report on the accident to the Project co-ordinator by the fastest possible means.

MEMA kwa Vijana Trial Further Survey (2007-2008)

MKV1 FS: Standard Operation Procedures for Census Interviewer (CI)

1. Make sure that you have the **census timetable** at least one week before the field trip.
2. One week before leaving for the field make sure that you have the correct and current version of both the **SOP** and the **Protocol**, read them carefully and follow the instructions while working/in the field.

3. One week before the field trip check that you have all required equipment (e.g. PDAs, GPS), documents and other supplies ***(Census Team Packing List)***. If there are any problems with the equipment then inform the CTL.

4. Two days before travelling, collect your **imprest** from the Project administrator.

5. The day before travelling:

    a. Pack all equipment/supplies that you need. **All PDA and laptops (including spare batteries) must be fully charged.**
    b. Attend the CT briefing meeting with the Mobilisation Officer (MO), Field Supervisor (FS) and Fieldwork Manager AND the rest of the CT. During this meeting the MO will give you any important information about the community.

6. On the day of departure, you will leave NIMR, Mwanza early in the morning so as to arrive in the survey community (ward capital) in the afternoon/early evening. **All travel to the field must start at NIMR, Mwanza.**

7. The day after arriving in the community (Day 1) you must:

    a. help the CTL with preparations for the census.
    b. Pack your bag for Day 2, double-checking that all equipment is working and that you have the necessary forms for your work the following day. Where possible carry with you extra copies of necessary forms and sheets.

MEMA kwa Vijana Trial Further Survey (2007-2008)

c. Attend the briefing meeting that the CTL will hold at the end of Day 1 and carefully note the kitongoji and CH assigned to you.

8. On Day 2 attend the briefing meeting at the village/vitongoji office. You must sit down with the other CI and CH working in your assigned kitongoji and divide up the households. Then you should carefully record the name and sex of household heads and respective HH number from the **Master lists of HH head** onto your **CI form for HH head**. Under normal circumstances you should not use any **additional list or sheet** apart from official list/sheets.

9. On Day 2, travel to your assigned kitongoji by project vehicle, bicycle or on foot and introduce yourself to the Kitongoji leader and community helper(s). Discuss the list of households (HH) and agree the plan for the census (where to start). **All HH must be visited i.e. visit HH even if Kitongoji leader has indicated that there are no young people aged 15-30 years.**

10. Using your ***CI form for HH head***, travel with the CH from household to household on bicycle or on foot. At each household introduce yourself and the purpose of your visit to the household members. If the household head (or another adult household member) is present then read the information sheet to them and answer any questions that they have. **Read out loud the information on the HH informed consent sheet to the household member before asking them to sign the HH consent sheet.** If the household head does not consent to participate in the census then thank the household head and move to the next household. If the household head is absent then another adult member of the household may give consent to participate. Complete the first part of the census form on the PDA.

11. If you visit a HH that is not on the Master list of HH then record details on this household on the ***list of additional HH heads***, record details of HH on your CI form for HH head and continue with HH interview.

12. If there is nobody at the household when you visit then enter 2 = “absent” on the column of census status for this household. Make a maximum of two further

MEMA kwa Vijana Trial Further Survey (2007-2008)

attempts to visit that HH later in the day (visits on the same day must be at least 4 hours apart) or on the next day and complete the columns of census status for this household. Record the date and time of each visit (Swahili time) on your **CI form for HH head**. If possible, leave a message with the neighbours to say that you will be returning at a specified time. **The same CI form for HH head will be used for all days in the same kitongoji.**

**Codes for CI form**	
**1**	**Interviewed**
**2**	**Absent**
**3**	**HH Refused**
**4**	**Repeated HH**
**5**	**HH not Known**
6	**HH Moved away**

13. If there are any young people aged 15-30 years living in the household then they should respond to the questions in the PDA that relate to them.

    a. If the young person is not present then ask the household head (and other HH members) to provide the necessary information about the young person. Give all eligible young people a survey invitation.
    b. If the young person is not present then leave a survey invitation at the household.
    c. If the eligible young person will not be able to attend the survey because they are travelling for a long period of time/ have moved away to study etc. then fill out the ***moved away form*** but still leave an invitation. Indicate on the CI form for HH head that you have left a moved away form (Please write **M** to the left of the name of the Household head on the CI form). **You must submit used moved away forms to the CTL at the end of each day.**

14. If the eligible young person is aged 17 years or younger then you must leave a copy of the ***Information sheet for parent(s)/guardian(s)***. You must also leave an ***Informed consent sheet for parent(s)/guardian(s)***. If the parent/guardian is

MEMA kwa Vijana Trial Further Survey (2007-2008)

present then ask them to sign the informed consent sheet (read first to them the information on the IC sheet). Signing of the IC sheet must be witnessed by the kitongoji leader or the community helper (must also sign the form). If the parent/guardian is not present or will not immediately sign the IC sheet then inform the HH members that the invited YP who is <18 yrs of age must take the survey invitation **AND** the IC sheet to the survey. **You must submit completed IC sheet for parent/guardian to the CTL at the end of each day.**

15. At each household take a waypoint reading using the GPS and record the co-ordinates in the PDA form. If after 3 attempts, you are unable to get the co-ordinates then leave the GPS field in the PDA form blank. Report any problems with the GPS to the CTL as soon as possible.

16. You should use the ***calendar of events*** to help the respondents recall dates.

17. Report any problems with the PDA, GPS or other equipment to the CTL as soon as possible. All problems must be recorded in the ***CT equipment notebook***.

18. In the unlikely event that it is not possible to use the PDA on the day of the census then the **CTL** will instruct you to use the paper ***Back-up Census Questionnaire.*** You should use the next **back-up census household number** on the sheet of numbers and cross through this number so that it will not be used again. If the young person is eligible then you should use the next **back-up census invitation number (Mwaliko)** on the sheet of numbers and cross through this number so that it will not be used again. You must only use this paper questionnaire if you are instructed to by the CTL.

19. At the end of the day:
    a. Return the PDA, GPS and any completed forms to the CTL.
    b. Attend the daily debriefing meeting and share your experiences with the rest of the team.

20. When requested you must assist the CTL with other census tasks eg synchronisation, burning of CDs, generation and printing of lists.

MEMA kwa Vijana Trial Further Survey (2007-2008)

21. In Mwanza (within 2 days of return from the field), you must complete the following tasks:
    a. Attend the CT debriefing meeting
    b. Return all equipment to the project storeroom
    c. Complete and submit all report and retirements required at NIMR office.

22. PDA use
    a. All text must be entered in capital letters
    b. Avoid entering other characters by accident e.g. '.' or ','
    c. GPS coordinates must be entered with 5 numbers after the decimal point i.e. ##.#####

MEMA kwa Vijana Trial Further Survey (2007-2008

MKV1 FS: Standard Operation Procedures for Census Team Leader (CTL)

1. Make sure that you have the census timetable at least one week before the field trip.

2. One week before leaving for the field make sure that you have the correct and current version of both the **SOP** and the **Protocol**, read them carefully and follow the instructions while working/in the field.

3. One week before the field trip check that you have all required equipment and test them (e.g. PDAs, GPS & Laptop computer), documents and other supplies are ready ***(Census Team Packing List)***. If there are any problems with the equipment you must inform the **Fieldwork Manager and/or Project Co-ordinator**.

4. One week before departure, you should make sure that you have collected from the MO all important information on the community to be visited ie names of villages, names of vitongoji, list of household heads, names of contact persons and census helpers, details of accommodation.

5. One week before departure, you must check that all the equipment/ supplies on the ***Census Team Packing List*** are ready.

6. Three days before departure you should make the following phone calls (if telephone numbers are available):

    a. Telephone the guesthouse to confirm the accommodation for the CT
    b. Telephone the leaders of the first village to let them know that the census team will start working in their village on a specific day

7. Two days before travelling, collect your imprests from the Project administrator. You are responsible for all money and equipment supplied to you.

MEMA kwa Vijana Trial Further Survey (2007-2008

8. The day before travelling you must supervise the packing of all the census team equipment. **You are responsible for checking that each piece of electrical equipment is fully charged and/or has spare batteries.**

9. The day before travelling, you must arrange a briefing meeting with the Mobilisation Officer (MO), Field Supervisor (FS) and Fieldwork Manager AND the CI. During this meeting the MO will give you any important information about the community.

10. On the day of departure, you will make sure that the CT leaves NIMR Mwanza early in the morning so as to arrive in the survey community (ward capital) in the afternoon/early evening.

11. The day after arriving in the community (Day 1), you will visit the WEO, WEC and other ward officials at the ward office. You will introduce yourself to the ward officials and let them know that the survey is about to start in the ward. You will provide officials with the updated survey timetable indicating the days that the census and survey teams will be in each village and sub-village. During these visits you should ask the WEO if there are any community or political issues that will make the census or survey difficult to carry out. Whenever possible, you should ensure that all community leaders at ward level are aware of the exercise. If following these discussions you think that it is necessary to change the census timetable then you must immediately contact the Field Supervisor (or Fieldwork Manager/ Project Co-ordinator).

12. On the same day (Day 1), you will travel to the nearest village and introduce yourself to the VEO, VC and the Vitongoji leaders. You will remind them of the census and survey procedures and inform them that the census will start in that village the next day and provide them with an updated census and survey timetable. You must remind them of the need for community helpers, the fact that informed consent will be obtained from the household head (or proxy respondent), the survey eligibility criteria and that the CT will be using small computers (PDA) to collect data. You should request the Vitongoji leaders to mobilise the residents of their sub-village (house-to-house mobilisation) so that

MEMA kwa Vijana Trial Further Survey (2007-2008

as many as possible of the residents are present on the day (s) of the census. You should encourage him/her to use the list of households (HH) to help them with this mobilisation.

13. During this visit (Day 1), you should meet with the community helpers (identified during the mobilisation visit) and explain to them the purpose and procedures of the census. You must ensure that the community helpers (CH) know their responsibilities during the exercise and tell them that they will receive 3000 Tsh/day for their help. Arrange to meet the Kitongoji leader and the other CH at 8:00am the next day (Day 2).

14. On the evening of Day 1 you will hold a short meeting with the CI. Give them on update on your progress during the day and inform them of any changes to the timetable. Assign each CI to specific kitongoji and give them the name of the CH who will be working with them.

15. On the day of the census you must ensure that all CI are equipped to conduct the census (suitably dressed, equipment charged, sufficient copies of all documents etc) and ensure that they have transport to take them to their assigned Kitongoji.

16. Before the census begins you must arrange a meeting with the CI and the CH at the Village/Vitongoji office. During this meeting the CI and CH for each Kitongoji will sit together and discuss the division of households between the CI working in that Kitongoji (if more than 1 CI assigned to that kitongoji). Ensure that the CIs are completing the CI form for HH head correctly.

17. You must provide the CI with 64 invitations for the first day of the survey (e.g. if 8 CI then give 8 invitations each). You must make sure that CI only distribute invitations for the second day of the survey when they have used all invitations for the first day. It may be necessary for you to redistribute the invitations in the evening so that invitations for the first day are used as soon as possible. **Only in exceptional circumstances should less than 64 people be invited for the first day of the survey in a village.**

MEMA kwa Vijana Trial Further Survey (2007-2008

18. During the census, you should visit each CI & CH and get feedback from them about the progress of the census.

19. After the team has moved from one Kitongoji you have to visit some of the house to check if the CI did interview that HH, this includes the interviewed, shifted HH, refused and absent. This has to be done regularly and focus on checking at least once in HH in which each the CI interviewed and give feedback to the team what you have found.

20. **In the unlikely event that it is not possible to use one or all of the PDA on the day of the census then you must make an urgent call to the FS, FM or PC to discuss the situation**. Only the FS, FM or PC can decide that the CT should switch to the paper ***Back-up Census Questionnaire.*** If the Back-up Census Questionnaire is to be used then you must inform the CI of the decision. Before the paper questionnaire is used, you should provide the CI with a short refresher training on the use of the paper questionnaire. When the Back-up census questionnaire is used you must make sure that the CI have the list of Back-up census household numbers and the list of Back-up census ID numbers (Mwaliko).

21. At the end of each day you must arrange transport for CI from the Vitongoji to the guesthouse. Where necessary, arrange transport to support survey team. This however should be done in a convenient time to avoid affecting census daily activities plan.

22. At the end of each day you must pay an allowance to each of the CH. Each CH should sign the ***Official MkV FS Payment Voucher*** (village/kitongoji, name of helper, date, amount, signature of payee).

23. Any problems with the PDA, GPS or other equipment must be recorded in the ***CT equipment notebook***. You are responsible for recording problems reported by the CI. If there is a need for replacement equipment then you must inform the FS, FWM or PC as soon as possible.

MEMA kwa Vijana Trial Further Survey (2007-2008

24. At the end of each day collect the PDA, GPS, used CI form for HH head and used HH head consent form from the CI. If necessary recharge (PDA) or replace the batteries (GPS) before safely storing overnight.
    Important: PDAs should be recharged in every two working days.

25. At the end of each day hold a debriefing meeting (approx. 30 mins to 1 hour) with the CI. Encourage them to share their experiences and discuss how the CT can improve their performance.

26. Upload the information collected onto the PDAs onto the laptop 'Synchronisation' ***(Census data SOP)***. Back-up the data collected that day onto a blank CD and label the CD (ward, village, vitongoji, date, staff ID number). *[NOTE: If the Back-up Census Questionnaire was used then you must, each evening, with the help of CI, enter the data from the questionnaire into the database created on the laptop* ***(Back-up HH and Invitees information)***

27. At the end of census exercise in each kitongoji you must complete the ***Census Village Report Form (CVRF)*** in particular columns of respective kitongoji. Each day you must complete the Daily Progress with Census. You should send a text update to the FS every evening indicating the number of households visited and the number of young people invited etc.

28. Mop-up of households should be done during the census when the CIs are still in the village. Only in exceptional circumstances, in consultation with the FS and STL, will additional mop-up be done at the end of a community (ward). Survey day [date(s) and location] for eligible YPs identified during this exercise should be agreed between CTL and STL in consultation from FS/FWM/PC.

29. In each village, you must complete the ***Village Information Form*** by interviewing **at least two** reliable informants in each village e.g. VEO, VC and/or Vitongoji leaders.

30. When the census is underway, visit the survey venue in that village and ensure that the venue is ready for the arrival of the survey team. Confirm the

MEMA kwa Vijana Trial Further Survey (2007-2008

accommodation for the survey team. Contact (text message) the survey team leader (STL) 2-3 days before arrival of survey team in the village and provide him/her with an update on the progress of the census and the survey venue /accommodation arrangements.

31. While the CIs are busy conducting the census in village 1, you must go to the next village and carry out the introductions/tasks as in village 1 (see 11 and 12). The same procedures must be followed in each village in the ward.

32. Under normal circumstances the CT will not spend more than 3 days in each village.

33. When the census has been completed in a village you must generate and print **2 copies** of the lists of invitees ***(Census data SOP).*** Keep one copy of these lists. The next day, deliver 1 copy of the lists to the survey team leader. **You must also deliver completed moved away forms and parent/guardian IC sheets to the STL.**

34. One day before leaving the ward you must visit the WEO and debrief him/her about the census exercise (thank him/her for assistance).

35. Once a week (at the time of survey specimen and data collection) you must submit the back-up CDs for that week, your copy of lists A1-A3 and any completed forms that you are finished using (completed village information sheets, Household IC sheets, CI HH head forms, Back-up census questionnaires (if used) and census village report forms).

36. In Mwanza (within 2 days of return from the field), you must complete the following tasks:

    a. Arrange a debriefing meeting with the Field Supervisor (FS) or Fieldwork Manager or Project Co-ordinator AND census team members.
    b. Submit list of eligible YP to the project office, A1,A2 and A3.
    c. Return all equipment / supplies to the project storeroom

MEMA kwa Vijana Trial Further Survey (2007-2008

d. Submit all forms and questionnaires to the data section at NIMR.
e. Complete the Census Community Report Form and submit to the Project Office.
f. Complete and submit retirements required at NIMR office.
g. Ensure service of the generator.
h. Make sure that you update antivirus.
i. Make sure that the vehicle logbook is photocopied.

MKV1 FS: Standard Operation Procedures for Data Census Team Leader (STL)

A. Synchronization process (Transfer of Data from PDA to Laptop)

1. Turn on your laptop
2. Connect your synchronization cable to the laptop using the USB-port
3. Insert the synchronization cable into the PDA. You will get a beep sound when the cable is placed in the right way.
4. Press the synchronization button on the cable. You will hear a sound and see a message on your computer telling you that the process is taking place.
5. When the process has finished remove the PDA.
6. Repeat process 3 and 4 for all the PDA you have.
7. When you are finished with the process of synchronization copy the database onto the CD (see CD burning procedure).
8. This process must be done daily.

B. CD Burning

9. Label a blank CD with the following information: Ward name, village name, Team number, staff code and date.
10. Turn on your laptop and insert the labelled blank CD into the CD drive
11. Copy the MS. Access database icon on the desktop named Link.
12. Open the D drive through my computer icon on the desktop then open the CD and paste the MS Access database on CD.
13. Click button “Write these files on the disc (DC)’.
14. You will see a message saying that the file has been written onto the disc
15. The D drive will open. You can remove the CD and close the D drive.
16. Store the CDs in safe place and give to the specimen/data collection vehicle the next time that it visits your team. Do not put the CDs in direct sunlight, dust place or wet place.

C. How to generate a list for the survey

17. Turn on your laptop
18. Click the icon of MS access on the desktop written “Linbk”
19. Click on the Query object of the MS access
20. Click the query named “Kijana query” a list of vijana will open
21. From the main menu Click “File” choose “Print”
22. The list of Vijana eligible for survey will be printed.

D. Charging PDA using TANESCO power

23. Connect the PDA charging cable to the surge-protected extension cable before connecting to the TANESCO socket (insert the cable as the arrow of cable indicate)
24. Then switch on the TANESCO power to allow electricity to flow to the PDA.
25. You will hear a beep sound to show that PDA connection is correct.
26. Turn on your PDA to assure that the PDA is charging, you see the battery has been crossed by a yellow sign and that's mean it is charging.
27. Charge for at least two hours each day.

MEMA kwa Vijana Trial Further Survey (2007-2008)

MKV1 FS: Standard Operation Procedures for Registration Interviewer (RI)

1. Two days before travelling, collect your **imprest** from the Project administrator.

2. One week before leaving for the field make sure that you have the correct and current version of both the **SOP** and the **Protocol**, read them carefully and follow the instructions while working/in the field.

3. When the attendees arrive, greet them and ask them to be seated in the registration waiting area.

4. Invite each attendee, one at a time, to join you at the registration table. Take the **Registration Book** and on a new line in the book record the date and time of interview, the attendees name, village, sub-village and the name of household head. Then ask the attendee to return to the waiting area.

5. Wherever possible, one RI will interview male attendees and the other RI will interview female attendees. The RI will share the same registration book.

6. Interview the attendee and complete section A of the main questionnaire:

NB All those who attend the survey must be recorded in the registration book AND must be recorded on section A of the main questionnaire. You should not assess eligibility of an attendee before registering them or before completing section A of the main questionnaire.

a. If the name of the attendee is on the list of those invited to the survey **LIST A (A1 or A2)** then carefully copy the census ID number onto the questionnaire. Only copy the census ID number from the invitation if you cannot find the attendee on List A.
b. Carefully examine any documentation indicating the date of birth of the attendee. If more than one piece of documentation is produced then take the date of birth from the most reliable document eg Birth certificate.

MEMA kwa Vijana Trial Further Survey (2007-2008)

c. If full date of birth is not known then use the **calendar of events** to help the attendee to remember/ calculate his/her age.
d. Use the **list of primary schools (LIST B)** to find the code for the school (s) that the attendee attended.
e. Use the **list of MkV1 participants (LIST C)** to find the name of any attendee who reports that they took part in MkV1. If the attendee has brought an MkV1 ID card then carefully inspect to ensure that the name and photo match the person in front of you. Carefully record the MkV1 ID on the main questionnaire (NOTE: the MkV1ID number should end with a letter. This letter is present on the MkV1 ID cards but missing from LIST C. If using LIST C then cross through the last space on the questionnaire to indicate that the letter is missing). Tick the column 'MkV1' in the registration book. ***If you cannot read the full MkV1 ID then please enter only the digits that you can read and leave the others blank. Write 'not read', your staff code and the date beside the question.***
f. If attendee did not participate in MkV1 then look on the **Std 7 exam results sheets (LIST D)** to verify the school attended. If they did not reach Std7 then ask them to name 2 classmates who reached Std 7.

7. If the attendee is eligible then ask the Informed consent check questions to determine if they are able to give informed consent**.**
   a. If the attendee is not able to give informed consent then ask them to return to the registration waiting area until one of the team members (STL, attendee instructor, counsellor or RI) can discuss the survey procedures with them in more detail.
   b. When the team member is confident that the attendee is able to give informed consent, you should ask the attendee return to the registration table and ask them if they consent to participate in the survey.
   c. Ask the attendee if he/she also consent to receiving follow-up treatment for any STIs that is diagnosed after the day of the survey. Remind them that if they consent to participate in the survey but would not like follow-up treatment then they are still eligible to participate in the survey.
   d. If consent is given then ask the attendee to sign or thumbprint the informed consent sheet.

MEMA kwa Vijana Trial Further Survey (2007-2008)

8. If you are not sure whether an attendee is eligible then discuss with the STL.

ELIGIBLE ATTENDEES (will now be called PARTICIPANTS):

9. If informed consent is given then place one sticker **(ST1)** with a unique survey ID number on the consent form, a second sticker **(ST2)** with the same number beside their name in the registration book. If their name is on the census list (List A1/2) then place sticker **(ST3)** beside their name on this list. Place stickers on sections A-C of the main questionnaire **(ST4-ST6)**.
10. Give the eligible consenting attendee ('participant') a plastic folder (clear bag) containing their main questionnaire (sections A-C) and additional stickers **(ST7-21)**. Ask them to wait in line for their turn to be interviewed by a same sex survey interviewer.
11. If there is a delay until the next SI is available, you must keep the plastic folder so that the participant does not read the Main Questionnaire while waiting. Give the folder to the participant when the interview is to about to begin.

NON-ELIGIBLE ATTENDEES:

12. If an attendee is not eligible, refuses to give informed consent or cannot give informed consent then use stickers for non-participants (begin with XFS…). Place one sticker **(ST1)** on the registration book, a second sticker **(ST2)** on the census list (List A1/A2) and a third sticker **(ST3)** on section A of the main questionnaire. Thank the attendee and tell them that they are not eligible to participate in the survey. If they were invited to the survey (have invitation or on List A1/A2) then tell them that they may access the clinician and VCT services. If they wish to avail of these services then place a fourth sticker **(ST4)** on section C of the main questionnaire and direct them to the clinician and/or VCT counsellor. Give the non-participant a plastic folder (clear bag) containing their main questionnaire (sections A and C only) and additional stickers **(ST5- 10)**.

13. If they were not invited or do not wish to avail of these services then tell them that they are free to leave the survey venue.

14. You are responsible, with the help of the attendee co-ordinator, for ensuring that the attendees in the registration waiting area are comfortable. If there are a larger

MEMA kwa Vijana Trial Further Survey (2007-2008)

number than expected, a fewer number than expected or if attendees are leaving before being interviewed then you must immediately inform the STL.

15. You must make List A1/A2 available to the Tracers so that they can record on list A3 the names of the invited young people who have attended.

16. During the course of the survey, the field data assistant will check the consistency of the questionnaire and will look to see if questions are appropriately skipped. If he/she finds that there are some inconsistency or questions skipped he/she will ask you to re-interview the same client to correct the inconsistency and/or skipped questions. The will be done before the client leaves the survey site. **You should reassure the client that they have not done anything wrong but that you made a small mistake and would like to double-check some of the questions.**

17. At the end of the day, ensure that all the registration equipment including Walkman, earphones, batteries and cassettes have been collected and are still in good working order. Report any problems with the registration equipment (walkmans) in the ***ST equipment notebook***.

18. At the end of every day, return the registration book, list A1/A2 with stickers attached and all questionnaires and forms to the STL.

19. Pack up all the registration equipment and leave the registration area clean and tidy.

20. Attend the daily debriefing meeting and share your experiences with the rest of the team.

21. When requested you must assist the STL with other survey tasks

MEMA kwa Vijana Trial Further Survey (2007-2008)

MKV1 FS: Standard Operation Procedures for Survey Interviewer (SI)

1. Two days before travelling, collect your **imprest** from the Project administrator.

2. One week before leaving for the field make sure that you have the correct and current version of both the **SOP** and the **Protocol**, read them carefully and follow the instructions while working/in the field.

3. When the participant arrives, greet him/her and ask him/her to be seated on the chair opposite you. Do your best to make sure that the participant is relaxed.

4. Ask the participant to give you their plastic folder. Remove section B of the main questionnaire from the folder and check that there is a sticker attached and that the sticker matches the sticker on section A of the questionnaire and the remaining stickers. If section A of the main questionnaire has not been completed, the stickers do not match or stickers are not attached to the questionnaire then ask the participant to return to the registration interviewer. If a sticker is attached and the first part of the questionnaire has been completed then attach the questionnaire to your clipboard and start the interview.

5. Make sure that you ask the participant what language they would like you to use and ensure that you use the language that they request.

6. You must ask the questions slowly using the wording on the questionnaire and in the order that the questions appear.

7. You can repeat questions if you feel it will help the participant's understanding, but you should not prompt answers for the respondent unless explicitly instructed to do so on the questionnaire.

8. The questionnaire must be completed neatly with **BLUE PEN**. **All sections of the questionnaire must be completed.**

9. Make sure that you use the ***Calendar of events*** to help the participant to remember events in the last 12 months, last month etc.

MEMA kwa Vijana Trial Further Survey (2007-2008)

10. When asking the questions (especially the questions on sexual behaviour) you must always remain respectful and non-judgemental. You should not show surprise, shock or any negative or positive reactions they may have in response to questions or comments. You must not say anything or display any emotion that will make the participant feel uncomfortable.

11. You should also take care not to influence the participants' answers in any way. If a participant asks a question during the interview and it is one that can be answered without influencing them (e.g., "How long will this take?"), the interviewer can answer the question. However, if a participant asks a question and you feel an answer might influence them (e.g., "What is a condom?"), you should explain that you cannot answer the question at that time. Instead, you should instruct the participant to ask those questions of the person who gives them soap at the end of the survey.

12. If the participant is unwilling to answer a question then explain again the reason for asking the questions and explain again that all answers will be kept confidential. If the participant still does not want to answer a question then leave blank and continue to the next question.

13. If there are any inconsistencies in the responses provided by the participant (eg reported number of sexual partners) then you should politely remind the participant of the response (s) provided in the previous question/section and try to resolve the inconsistencies.

14. If you make a mistake then cross through the incorrect response and write your staff code and the date beside the correction.

15. During the course of the survey, the field data assistant will check the consistency of the questionnaire and will look to see if questions are appropriately skipped. If he/she finds that there are some inconsistency or questions skipped he/she will ask you to re-interview the same client to correct the inconsistency and/or skipped questions. The will be done before the client

MEMA kwa Vijana Trial Further Survey (2007-2008)

leaves the survey site. **You should reassure the client that they have not done anything wrong but that you made a small mistake and would like to double-check some of the questions.**

16. Ten percent of all participants will be also be interviewed using a quality control (QC) questionnaire to check the consistency of reported data. The QC interviews will be carried out by a different, same-sex SI after the participant's visit to the Clinician. The QC questionnaire includes about 3 pages of the main questionnaire. General questions have been added to the front of the QC questionnaire, so that it does not begin with very sensitive questions. After a QC interview, you should accompany the participant to the RI to hand over the QC questionnaire

17. When requested you must assist the STL with other survey tasks.

MEMA kwa Vijana Trial Further Survey (2007-2008)

MkV1FS: Standard Operating Procedures for Data Checker

1. Two days before travelling, collect your **imprest** from the project administrator

2. One week before leaving for the field make sure that you have the correct and current version of both the **SOP** and the **Protocol**, read them carefully and follow the instructions while working/in the field.

3. On the day of departure, you will leave NIMR, Mwanza early in the morning so as to arrive in the survey community (ward capital) in the afternoon/early evening. **All travel to the field must start at NIMR, Mwanza**

4. Great the participant when she/he arrives, and ask him to sit on a chair opposite to you.

5. Ask the participant to give you his/her plastic folder with questionnaire. Then apologise to the client that he/she might take a long time waiting while checking the questionnaire.

6. Remove section A, B and C of the questionnaire and check if is attached with stickers and if the stickers' numbers match to each section of the questionnaire.

7. Take section A and B of the questionnaire, then start to check the following:

   (a) All skips are correct
   (b) All questions that should be completed are completed
   (c) There is consistency between section A and B eg female in part A should be female in part B, age at first sex or marriage is less than current age
   (d) In section B also check the consistency between question No. 4 and No.5.

8. If you see any skipped questions or inconsistency, ask the client to wait, and inform him/her that he/she might be interviewed again on some of the questions. Check the staff code of the interviewer and then go and inform him/her that there is some inconsistency in the questionnaire or some questions have not been answered. Then ask the client to go back for the interview. If the particular interviewer is in the middle of an interview with another client then wait till he/she finishes that interview.

MEMA kwa Vijana Trial Further Survey (2007-2008)

9. After the repeat interview, check again the questionnaire to see if the inconsistency or skip has been corrected. If there is still a problem then you should contact the STL and ask for their assistance. When you are satisfied with the questionnaire, thank the client for being patient give him/her the incentives and tell the client that he/she is free to leave.

10. If the interviewer does not understand the mistakes that have been made, immediately report to the STL to avoid delays to the client.

11. If necessary eg when there are a large number of clients waiting to be interviewed you will work as a backup survey interviewer.

12. You should record in your error record book all the errors found for each questionnaire checked, and then at the end of the day discuss with the TL.

13. At the end of the survey day collect all the questionnaires and arrange them by sections and then submit them to the TL with consent forms from registration and payment vouchers.

14. When requested you must assist the STL with other survey tasks.

MEMA kwa Vijana Trial Further Survey (2007-2008)

MkV1FS: Standard Operating Procedures for Attendee Co-ordinator

1. Two days before travelling, collect your **imprest** from the Project administrator.

2. One week before leaving for the field make sure that you have the correct and current version of both the **SOP** and the **Protocol**, read them carefully and follow the instructions while working/in the field.

3. You should ensure that the reception and registration section is well arranged and organized.

4. When an attendee arrives at the survey site give each attendee a copy of the ***Information Sheet for Participants*** and a Walkman with the project information cassette. Show the group of attendees how to play, stop and rewind the cassette and ask them to listen carefully to all of the information on the cassette. Answer any questions or queries raised by the attendees.

5. After the attendee has listened to the walkman and read the information sheet you should sit down with the attendee (or group of attendees) and explain the procedures of the survey and answer any questions that they have. If you have difficulty in answering any question then you should seek advice from the Survey Team Leader (STL).

6. When the attendees have listened to the cassette/ have read the information sheet invite them, one at a time, to join you at the registration desk. Make sure that they return the walkman+ earphones+ cassette+ batteries and mark that they have done so on the **Walkman Lending List**. You are responsible for taking care of the Walkmans and walkman accessories.

7. While the survey is ongoing you should help the STL to ensure that everything is flowing smoothly. In particular, you must make sure that the attendees know where they are going and you should accompany them to the next part of the survey if necessary.

MEMA kwa Vijana Trial Further Survey (2007-2008)

8. When the attendee has completed all relevant stages of the survey, provide all non-eligible invitees (only those who were invited i.e. have invitation and/or name appears on List A) with TSH 2000 and half a bar of soap. **Ensure that they sign the payment form.** Provide all eligible invitees with TSH 4000 and a full bar of soap. **Ensure that they sign the payment form.** Non-eligible attendees who were not invited by the census team should not receive any money or soap.

9. Sometimes the attendees need to wait for a long time and you should do your best to make sure that their wait is enjoyable eg by chatting to them, providing them with newspapers/magazines etc. If the STL decides that refreshments should be provided you should ensure that these are distributed fairly. Refreshments will be provided for the participants and not for staff members.

10. When requested you must assist the Survey Team Leader (STL) with other survey tasks.

MEMA kwa Vijana Trial Further Survey (2007-2008)

MKV1 FS: Standard Operation Procedures for Tracers

1. When the survey is up and running, you will travel to the sub-village allocated to you by the STL. Before leaving you will transfer information from census list A3 (List of Household heads) and registration lists A1/A2 onto your **Tracing Form**. You will use this tracing form to help you when you are tracing and will record the outcome of your activities on this form. You will use either the project car or the bicycle for the villages/sub-villages, which are very far from the survey venue, or go on foot for the nearest sub-villages.

2. Two days before travelling, collect your **imprest** from the Project administrator.

3. The day before travelling:
    a. Attend the ST briefing meeting with the, Project Coordinator (PC), Field Supervisor (FS) and Fieldwork Manager AND the rest of the ST.

4. On the day of departure, you will leave NIMR, Mwanza early in the morning so as to arrive in the survey community (ward capital) in the afternoon/early evening. **All travel to the field must start at NIMR, Mwanza.**

5. If you discover that the young person will not be able to attend the survey because they are temporarily living elsewhere then you must complete a **Moved Away Form** for this person.

6. Every evening you will, under the guidance of the Survey Team Leader (STL), make a note of all those who did not attend the survey on that day (by checking the names on List A1/A2 that have no sticker and date of survey beside their name).

7. On subsequent days of the survey you will mobilise those who are due to attend the survey on that day but will also try to find those who did not attend on previous days.

8. When mobilising in the sub-village you should try to speak directly with the invited young person. Explain again the purpose of the survey and request that

MEMA kwa Vijana Trial Further Survey (2007-2008)

he/she helps the survey team in their work. Make sure that he/she understands that he/she will be compensated for their time and traveling expenses. If the invited young person refuses to participate then thank them politely and leave.

9. You may use a GPS and/or ask other community members to help you to locate the households of the young people.

10. When requested you must assist the Survey Team Leader (STL) with other survey tasks.

MEMA kwa Vijana Trial Further Survey (2007-2008)

MKV1 FS: Standard Operation Procedures for Laboratory Technician (LT)

Procedure for collection of urine specimens and blood by venepuncture

Applicable to: All field laboratory personnel

Responsible person: Field Laboratory technicians for MKV1 FS

Aim: To describe the procedure for collecting and aliquoting urine and blood specimens.

Safety:

- Treat all samples as potentially infectious.
- Appropriate biosafety practices should be adhered to when handling specimens and reagents.
- These precautions include but not limited to the following:
  - Wear gloves
  - Do not pipette by mouth
  - Do not eat, drink, smoke, apply cosmetics or handle lenses at the areas of work.
  - Clean and disinfect all spills of specimens, reagent, blood and other potentially contaminated or infectious materials in accordance with local regulations.
- NB: If at any stage the gloves get dirty or split, or you are worried that they might have been holed, you should replace them with a new pair. Any discarded pair of gloves must be put into a bucket that is partially filled with disinfectant.

Procedure:

1. Two days before travelling, collect your imprest from the Project administrator.

2. One week before leaving for the field make sure that you have the correct and current version of both the **SOP** and the **Protocol**, read them carefully and follow the instructions while working/in the field.

3. When the participant arrives, greet him/her and try to put him/her at ease.

4. Ask him/her for the plastic folder and check that it contains the main questionnaire and remaining stickers.

5. Check that the sticker on the questionnaire matches the remaining stickers. If they do not match then ask the participant to return to the RI.

6. Enter LT staff codes in the Main Questionnaire (Q10.01) and record the date and sticker number in the **laboratory technician register book**.

Urine specimen collection

7. Ask the participant when s(he) last urinated. If this was less than 2 hours ago then ask him/her to return to the registration area to please wait until 2 hours are up.

8. Put a sticker on the urine container with sellotape.

9. Explain to the participant how to collect urine specimen, and give the labelled container to the participant, asking him/her to go to the latrine to collect urine and bring the urine container back.
NB: Be careful not to send more than one or two participants to the latrine at the same time, to reduce the chance of exchange of urine specimens.

10. Keep the Main Questionnaire and remaining stickers while the participant goes to collect urine specimen.

11. When the participant returns with his or her urine container, one of you should receive the urine container and process the specimen while the other does blood collection (see below).

12. Label two polypropylene tubes with stickers, and check to ensure that the sticker numbers match the one on the urine container. Secure the stickers with sellotape.

13. Using a sterile plastic pasteur pipette, aliquot slightly less than 2mL (approx. 1.8mL) into each of the pre-labelled tubes.

14. Put each aliquot into the appropriate pre-labelled cryobox and place the cryoboxes with the urine specimens in a freezer. Cryoboxes should be labelled as follows:

**MkV1 FS**
**Urine Samples**
**Ward */ Village ****

**dd/mm/yy**

*SGM= Sengerema; GTA= Geita; MSN= Missungwi; KWM= Kwimba

** Full name of village

NB: That the last slot in each UPT box must be left empty for the positive control.

15. Dip the LED dipstick into the urine remaining in the 60mL container and start the stop clock (1 minute).

16. After one minute, compare the colour of the dipstick against the colour chart on the dipstick bottle.

17. Record the results (presence or absence of erythrocytes) of the LED dipstick in the main questionnaire and in the laboratory technician register book. Score in the following way:

0 = Negative
1 = Positive (+)
2 = Positive (++)
3 = Positive (+++)
4 = Positive (++++) (NB Some LED dipsticks may not have this result)

18. For female participants: Wrap the 60mL container with the remaining urine in an envelope and give to the participant. (Discard the remaining urine specimen of Male participants).

19. Place the pipette and discarded dipstick into a bucket containing disinfectant.

20. If less than 2 aliquots of urine are collected then please note this on the questionnaire and in the laboratory technician register book.

Blood collection and aliquoting

21. Label with stickers one plain 10mL vacutainer tube and three serum tubes per participant. Ensure the stickers are sealed with sellotape.

22. Explain to the client that you are going to collect blood by venepuncture.

23. Explain to her that there will be a small pain as the needle is inserted into the vein.

24. Apply a tourniquet above the elbow until the vein is raised.

25. Remove the sheath from the lower part of the needle and fit it on the holder and then place the vacutainer into the holder.
Do NOT push the vacutainer tube completely onto the needle as the vacuum might be lost.

26. Swab the skin using cotton wool soaked in methylated spirit and wipe excess spirit with a dry cotton wool (or mediswab if available).

27. Remove the upper vacutainer needle cover, push the needle into the vein until you are sure it is in and then push in the vacutainer tube completely into the needle.

28. Watch the blood flow in the vacutainer until it nearly reaches the top.

29. Release the tourniquet and then remove the needle. Put cotton wool soaked in spirit over the puncture mark and ask the client to press on the cotton wool.

30. Unscrew the needle from the holder and dispose of in a safe container/disposal bin.

31. Place the vacutainer tube flat on the work bench and leave blood to clot for at least **30 mins** (you should use timer to measure 30 mins).

32. If you have difficulty in drawing blood using the vacutainer then you (or your fellow lab tech) should try again using a new vacutainer on a different vein. If you are still unsuccessful then you must call the Clinician who should make a maximum of two further attempts to draw blood using a direct needle. In the rare event of all these attempts failing then the participant should be allowed to proceed to the Clinician.

33. Complete and return the questionnaire to the participant together with the remaining stickers and **(for females only)** the urine container. Thank the participant and direct him/her to the Clinician.
NOTE: You should not wait until you have processed the serum to send the participant on to the Clinician. Leave Q10.04 blank and complete in the evening at the guesthouse (see below).

Blood processing

34. Place the vacutainer tubes in the centrifuge and spin at 3500 rpm for 10 minutes.

35. At the end of centrifugation time remove vacutainer tubes from the centrifuge and place the tubes upright on the rack.

36. Using a sterile disposable pasteur pipette, transfer 3 x 1mL aliquots into the pre-labelled tubes. Each aliquot must be of 1mL. If less than 3 aliquots are collected then record this on the lab submission form and in the laboratory technician register notebook.

37. Put each aliquot into the appropriate pre-labelled cryobox. Place the cryoboxes with serum tubes in the freezer

**MkV1 FS**
**Serum Samples**
**Ward */ Village ****
**dd/mm/yy**

*SGM= Sengerema; GTA= Geita; MSN= Missungwi; KWM= Kwimba

** Full name of village

Note: The last slot in each serum box must be left empty for the positive control.

38. Place the Pasteur pipettes and vacutainer tube in the sharps container.

NB: At the end of the day, pack all equipment and swab-down all work surfaces with a disinfectant.

39. During the course of the survey, the field data assistant will check the consistency of the questionnaire and will look to see if questions are appropriately skipped. If he/she finds that there are some inconsistency or questions skipped he/she will ask you to re-interview the same client to correct the inconsistency and/or skipped questions. The will be done before the client leaves the survey site. **You should reassure the client that they have not done anything wrong but that you made a small mistake and would like to double-check some of the questions.**

40. Before leaving the survey site, you must ensure that all of the disposable materials (urine containers, gloves, dipsticks, used hand towels, and the used disinfectant) that you and the counsellor and clinician have used are disposed of properly, either in the pit latrine or burying them.

MEMA kwa Vijana Trial Further Survey (2007-2008)

41. Every evening, back in the guesthouse, check that all the specimens are stored in the freezers. You should also prepare all the other supplies (including cool packs) that you will need for the next day, and ensure that all specimens and necessary forms that they have been taken to-date are ready to be sent to Mwanza if someone was to come for them the next day (even if this is not the expected day for specimen collection).

42. You must collect all the Main Questionnaire from the Team Leader and record the number of serum aliquots taken on each of the Main Questionnaire forms (Section D, Q10.04). You should do this by transferring the data from the laboratory submission form and/or laboratory technician register notebook. Staple the lab submission form, and hand it to the Team Leader, along with the Main Questionnaires (in serial order of Sticker Numbers) for subsequent submission to the Data Section.

MEMA kwa Vijana Trial Further Survey (2007-2008)

MKV1 FS: Standard Operating Procedures for Clinician

1. Two days before travelling, collect your **imprest** from the Project administrator.

2. One week before leaving for the field make sure that you have the correct and current version of both the **SOP** and the **Protocol**, read them carefully and follow the instructions while working/in the field.

3. When the young person arrives, greet him/her and ask him/her to be seated on the chair opposite you. Do your best to make sure that they are relaxed.

4. If the young person is a participant ask him/her to give you their plastic folder. Remove section C of the main questionnaire from the folder and check that there is a sticker attached and that the sticker matches the stickers on Sections A and B and the remaining stickers. If any of the previous sections of the questionnaire have not been completed, the stickers do not match or a sticker is not attached to the questionnaire then ask the participant to return to the registration interviewer. If everything is in order then you may proceed. If the young person is not participating ('Non-participant') in the survey i.e. has a sticker number starting with XFS… then they will not have section B of the questionnaire.

5. In order to put the young person at ease, you should then enquire about their general health (i.e., "How are you today?"). After these introductory questions, you should explain what you are going to do next: Ask some questions about the person's reproductive health and treat any illnesses that you find free-of-charge. You should then check whether the participant is happy to continue.

6. If the young person consents, you should ask each female whether she is pregnant or is currently having her menstrual period, and record the results in Section C of the Main Questionnaire.

7. You should then ask the young person whether they have any reproductive tract symptoms (e.g. abnormal genital discharge, genital ulcer, genital itching, genital

MEMA kwa Vijana Trial Further Survey (2007-2008)

pain, lower abdominal/pelvic pain, genital warts, swollen inguinal glands), entering responses in Section C of the Main Questionnaire.

8. You should examine the genitals of ALL consenting males. Males should be examined in the standing position. Genital examination (external genitals only) should be carried out on ONLY those consenting females who report the symptoms of GUS. Females should be examined while lying on a bed or mattress on the floor.

9. You should then tell the young person (participants only) the result of the RBC dipstick test.

    a. If it is less than 3+, you should reassure them, saying that they did not have a significant amount of blood in the urine, so they do not require treatment for schistosomiasis.

    b. **For males:** If the RBC result is 3+ or 4+, you should explain that the urine test showed that the urine had blood in it, and you will treat him for schistosomiasis.

    c. **For females**: If the RBC result is 3+ or 4+, but the woman is menstruating, you should explain that the urine test showed that the urine had blood in it, but this might be due to the menstrual period. You should ask whether the woman has noticed bloody urine when she is not menstruating. If the RBC result is 3+ or 4+, and the woman is not menstruating, you should explain that the urine test showed that the urine had blood in it, and that you will treat her for schistosomiasis.

10. If the young person had symptoms (or signs) that are suggestive of an STI, you should also explain that you will give them the appropriate treatment. If not, you should reassure them.

11. You should then give the young person any treatment that is indicated following Tanzanian national guidelines for the syndromic management of STIs and/or

MEMA kwa Vijana Trial Further Survey (2007-2008)

schistosomiasis (see table). If a female participant requires treatment for a STI or for schistosomiasis and she is not visibly pregnant then you must carry out a pregnancy test (on the urine remaining in the 60mL container) before deciding on the most appropriate treatment *(If necessary female non-participants should be asked to provide a urine sample at this point).*

Number of Praziquantel tablets to be given to subjects under the 40mg/kg regimen

(In this table 13-15 means 13.1kg to 15.9kg. The same applies to other weight ranges)

**Bodyweight (kg)**	**Number of tablets**
13-15	1
16-18	1 ¼
19-22	1 ½
23-25	1 ¾
26-29	2
30-33	2 ¼
34-37	2 ½
38-40	2 ¾
41-44	3
45-48	3 ¼
49-52	3 ½
53-55	3 ¾
56-59	4
60-63	4 ¼
64-66	4 ½
67-70	4 ¾
71-75	5

12. **Ask the clients to repeat the frequency of how to take drugs to ensure that they have understood and will not overdose or under-dose the drugs when they reach home.**
13. **Weigh every client who needs schistosomiasis treatment.**

MEMA kwa Vijana Trial Further Survey (2007-2008)

14. **If pregnant clients have schistosomiasis, she should be referred to a near by Health center for treatment after delivery.**

15. All young people with a STI will be requested to notify all their recent sexual partners. You should be given a ***Contact Referral Slip*** for each of their partners. The participant will be asked to request their partners to attend a local health facility to receive advice and treatment.

16. If you discover that a young person has a genital ulcer then you should explain that Herpes Simplex Virus 2 is a sexually transmitted infection that cannot be cured but can be treated. You should explain that many young people are already infected with this disease and should describe the symptoms. You should encourage the participant to attend a health facility if they have any genital ulcers in the future.

17. Treatment for other acute and important health problems should also be provided as appropriate. Anyone with health problems that require further treatment should be advised to attend the nearest appropriate health facility. In the case of emergencies, the Team Leader may allocate staff and a car to accompany the participant/ non-participant to a health facility.

18. You should record all treatment given in the ***Clinician's Treatment Register Book***.

19. Consult the QC list of the sticker numbers of participants who should have a QC Questionnaire administered. If the sticker number on the questionnaire is on the QC list:
    a. Put a sticker on the appropriate QC Questionnaire, and check which interviewer did the original interview.
    b. Thank the participant and ensure that one of the team members takes him/her and their QC Questionnaire to a different same sex survey interviewer for a QC interview.
    c. Make sure that the participant's Main Questionnaire is left with the RI or VCT Counsellor (If he/she would like to avail of the VCT service).

MEMA kwa Vijana Trial Further Survey (2007-2008)

20. If the young person is not selected for a QC interview, you should thank him or her, and direct him/her to the RI or VCT Counsellor (if he/she would like to avail of the VCT services).

21. During the course of the survey, the field data assistant will check the consistency of the questionnaire and will look to see if questions are appropriately skipped. If he/she finds that there are some inconsistency or questions skipped he/she will ask you to re-interview the same client to correct the inconsistency and/or skipped questions. The will be done before the client leaves the survey site. **You should reassure the client that they have not done anything wrong but that you made a small mistake and would like to double-check some of the questions.**

22. You should make sure that you are familiar with the project's Post-exposure prophylaxis (PEP) guidelines and ensure that you have copies of all four PEP forms. In the event of a needle stick injury you should assess exposure and administer first aid. If necessary you should administer PEP drugs. Encourage pre-test counselling and HIV testing for the source and exposed according to the PEP guidelines. You must ensure that the exposed person travels with 2 vacutainers of blood well labelled ('source' and 'exposed') in a closed envelope with PEP report to PEP supervisors in Mwanza. The STL will assist you if necessary.

23. You should stand in for the Team Leader in the event that the Team Leader is ill or absent for any reason.

MEMA kwa Vijana Trial Further Survey (2007-2008)

MKV1 FS: Standard Operating Procedures for VCT Counsellor (VC)

1. Two days before travelling, collect your **imprests** from the Project administrator.

2. One week before leaving for field, make sure that you have the correct and current version of both **SOP** and the **Protocol**, read them carefully and follow the instructions while working/in the field.

3. On the day of departure, you will leave NIMR, Mwanza early in the morning so as to arrive in the survey community (ward capital) in the afternoon/early evening. **All travel to the field must start at NIMR, Mwanza.**
4. The day before travelling:
    a. Attend the ST briefing meeting with the, Project Coordinator (PC), Field Supervisor (FS) and Fieldwork Manager AND the rest of the ST.

5. When the young person arrives, greet him/her and ask him/her to be seated on the chair opposite you. Do your best to make sure that they are relaxed.

6. If the young person is a participant ask him/her to give you their plastic folder. Remove section C of the main questionnaire from the folder and check that there is a sticker attached and that the sticker matches the stickers on section A and B and the remaining stickers *(NOTE: Non-participants will not have section B)*. If any of the previous parts of the questionnaire have not been completed, the stickers do not match or a sticker is not attached to the questionnaire then ask the participant to return to the registration interviewer. If everything is in order then you may proceed.

Pre-test Counselling

7. Read the "information about the voluntary HIV testing service" that is on the Main Questionnaire to the young person.
8. Ask the question: "Do you want to be told your HIV test result?"
9. **If they answer no**, they do not want to know their HIV test result: Thank the young person and tell them that they should return to the RI to collect their incentive.
10. **If they answer yes**, they do want to know their HIV test result, continue with the pre-test counselling described below.
11. **For participants:** First, check that they really understood the question, by explaining again that the blood specimen that they just gave to the lab worker will be taken to NIMR Mwanza where it will be tested for HIV and other infections. The results of those tests will be kept completely secret. However, if they want to know their HIV

test result, then you can perform another test and give them their results today. If they want to continue.

12. **For non-participants:** Do not discuss laboratory specimens and simply proceed to (13).
13. Explain the following:
    a. What an HIV test is (a test for the body's reaction to infection by the HIV virus).
    b. The body does not react to the HIV virus immediately, and the test can still be negative for up to about 6 months after HIV infection. In other words, it is possible for someone who has been infected recently to test negative.
    c. The difference between HIV infection (the body being infected by the HIV virus), and AIDS (the disease that is caused by infection with the HIV virus), and the fact that someone can be infected by the HIV virus for many years before developing any illness (AIDS).
    d. Someone who has been infected with HIV virus (and is HIV-positive) cannot become HIV-negative. There are no drugs or other treatments that can get rid of this infection.
    e. Drugs are now available through government health facilities that can delay the onset of AIDS and people who take these drugs can live healthy lives for many years.
    f. Different people have different risks of being infected with HIV. The HIV virus is passed from one person to another mainly by sexual intercourse ("making love"). Ways to reduce the risk of becoming infected yourself and/or of infecting someone else include:
    - Not making love at all (sexual abstinence). This is the safest way.
    - Only making love with one partner whom you trust.
    - Particularly avoiding casual (one-off) sexual partners
    - Always using a condom when making love.
14. Ask the young person why they want to know their HIV test result. This should lead naturally into asking them what they think the potential advantages and disadvantages of knowing their HIV status are.

You should ensure that this discussion includes:

Potential Advantages	Potential Disadvantages

1. **Make better informed plans for the future** (such as about whether to get pregnant or whether to get married)<br>**2. If the result is negative:**<br>• Make better informed decisions to ensure you stay HIV negative<br>**3. If the result is positive:**<br>• Make better informed decisions to avoid infecting others<br>• Be aware that after some months or years, HIV-infected people start to get other infections, such as chronic cough or diarrhoea, that can be treated with drugs, so they should go immediately to a health facility to get treatment if they develop any illness<br>• Care and Treatment is available through government health facilities to help to delay the onset of AIDS<br>• You may be able to join a support group for people who are HIV infected if there is one in your village	**1. If the result is negative:**<br>• There is a small chance that this result will be misleading if you have been infected within the past 6 months and your body has not developed enough of a reaction to the HIV virus for this to be detected by the HIV test<br>**2. If the result is positive:**<br>• You may find this very distressing<br>• If you tell other people and it becomes widely known in the village that you have HIV infection, some people may condemn you and say bad things about you. Also, some people might not want to marry or make love with someone they know is HIV infected.

15. Ask whether the young person has any further questions, and answer them to the best of your ability, and as truthfully as possible.
16. When you have finished giving the pre-test counselling, ask the attendee/participant if they are satisfied with the pre-test counselling. If they are not then answer any additional questions that they have and if necessary repeat the information that you have already provided. If they are then satisfied enter 1=Yes to either Q12.03 or Q12.04 on the main questionnaire.
17. Ask again: “Do you want to be told your HIV test result?”, and enter the answer on the Main Questionnaire.
18. If they answer no, they do not want to know their HIV test result: Thank the young person and ask them to return to the RI to collect their incentive.
19. If they answer yes, they do want to know their HIV test result: Complete a ***Request for HIV Test Result Form*** and place an ID sticker on this form and a sticker in the ***Counsellor record book.***

HIV testing

20. See SOP for Bioline and Determine Rapid HIV tests.
21. On the **HIV VCT Results Form**, you should write result of test 1 (Bioline), the result of test 2 (Determine), and the overall HIV result, the date of the final result, your staff Code, and then sign the form. Fold the form in half and put to one side.

MEMA kwa Vijana Trial Further Survey (2007-2008)

Post-test Counselling

22. Before giving the young person their results ask them whether they have any questions they would like answered first.
23. After answering any questions, ask them whether they still want to know their HIV test result.
24. If the participant does not want to know the result:
    - Thank them and tell them that you will now burn their result in front of them, so nobody will ever be able to know their result.
    - Burn the HIV VCT Results Form front of the participant.
    - Thank them, ask them if they have any further questions and end the consultation.
    - Record in the Counsellor Record Book that the young person did not want to know their result.
25. If the participants say they want to know the result:
    - Tell them that, before giving them their result, you want to go over the same information that you gave them before the test. Remind them of the information in (9) and (10) above.
    - Then say: “I am now about to tell you your results. If the result is negative, what will you do?” Discuss this with them.
    - Then say: “I am now about to tell you your results. If the result is positive, what will you do?” Discuss this with them.
    - Ask whether the participant has any further questions, and answer them to the best of your ability, and as truthfully as possible.
    - Tell them their result.
    - **If the result is negative**:
        - Remind them that the body does not react to the HIV virus immediately, and the test can still be negative for up to about 6 months after HIV infection. In other words, it is possible for someone who has been infected recently to test negative.
        - Discuss a personal risk-reduction plan with them to ensure that they stay negative.
        - Ask them whether they have any questions, and answer them to the best of your ability, and as truthfully as possible.

Potential Elements of a Personal Risk-Reduction Plan
• Not making love at all (sexual abstinence). This is the safest way.<br>• Only making love with one partner whom you trust.<br>• Particularly avoiding casual (one-off) sexual partners.<br>• Always using a condom when making love.

- **If the result is positive**:
  - Give them time to absorb the information.
  - Offer emotional support and ask about individuals or groups within their village that they can turn to for help and support after the counselling is over.
  - Discuss possible reactions; denial, shock, anger & revenge, fear, depression & resignation.
  - Remind them of the difference between HIV infection (the body being infected by the HIV virus), and AIDS (the disease that is caused by infection with the HIV virus), and the fact that the HIV virus can infect someone for many years before developing any illness (AIDS).
  - Discuss sharing the result with sexual partner(s) (+/- whether partner(s) would like to be tested and where this is possible).
  - Discuss the importance of attending a HIV clinic for assessment for elibility for antiretroviral therapy. Explain what CD4 count and antiretroviral therapy are.
  - Encourage a positive approach to staying healthy.

**Positive approach to staying healthy**
• Seek treatment immediately for any infections (eg. Chronic cough or diarrhoea, fever, etc)<br>• Getting enough rest if you feel very tired or ill<br>• Eating fruits and some meat of fish at least once per week (if possible)

  - Discuss ways in which they can reduce the risk of infecting other people.

**Ways to Reduce the Risk of Infecting Other People**
• Not making love at all (sexual abstinence). This is the safest way.<br>• Always using a condom when making love.

  - Before closing the session, ensure that their immediate plans, intentions and actions have been reviewed.
- **If the result is indeterminate**
  - **If Eligible and blood sample has been collected by lab:** Explain to the client that the result is not clear and that you need to wait for a confirmatory test that will be carried out on the blood sample that has already been collected.
  - **If not eligible OR blood sample has not been collected by lab:** Take a blood sample using a vacutainer (according to the Blood collection and aliquoting SOP). Deliver the labelled vacutainer immediately to the

laboratory and explain to them that this sample should be processed and the serum sent to NIMR, Mwanza during the next sample collection. The laboratory should be informed that testing of this sample is priority and that the MkV1 FS co-ordinators should be informed when the results are ready.

- Make an appointment in **4 weeks time** with the client and explain that a counsellor (could be another counsellor) will come to meet him/her with the result. Fill out a VCT Discordants form and submit to NIMR at the time of next sample collection.

{Blood collection and aliquoting SOP

i. *Label with sticker one plain 10mL vacutainer tube. Ensure the stickers are sealed with sellotape.*

ii. *Explain to the client that you are going to collect blood by venepuncture.*

iii. *Explain to her that there will be a small pain as the needle is inserted into the vein.*

iv. *Apply a tourniquet above the elbow until the vein is raised.*

v. *Remove the sheath from the lower part of the needle and fit it on the holder and then place the vacutainer into the holder.*

vi. *Do NOT push the vacutainer tube completely onto the needle as the vacuum* ***might be lost.***

vii. *Swab the skin using cotton wool soaked in methylated spirit and wipe excess spirit with a dry cotton wool (or mediswab if available).*

viii. *Remove the upper vacutainer needle cover, push the needle into the vein until you are sure it is in and then push in the vacutainer tube completely into the needle.*

ix. *Watch the blood flow in the vacutainer until it nearly reaches the top.*

x. *Release the tourniquet and then remove the needle. Put cotton wool soaked in spirit over the puncture mark and ask the client to press on the cotton wool.*

xi. *Unscrew the needle from the holder and dispose of in a safe container/disposal bin.*

xii. *If you have difficulty in drawing blood using the vacutainer then you should try again using a new vacutainer on a different vein. If you are still unsuccessful then you must call the Clinician who should make a maximum of two further attempts to draw blood using a direct needle. }*

26. For all those who agree to have their test result, enter the HIV test results beside the sticker number in the VCT Counsellor record book.
27. For all those who agree to have their test result, explain to them that a confirmatory HIV test will be carried out on their serum in Mwanza and that they may be contacted again if there has been a problem with their test. Inform them that it is very unlikely that there will be a problem with their test.
28. For all those who test positive for HIV, fill out a ***HIV treatment referral slip.*** Give them information on the nearest referral centre. Provide them with enough transport money for transport for 3 trips to the referral centre. If there is a home based care organisation working in the survey area then put them in contact with the organisation so that they can receive supportive counselling and nutritional care.
29. When participant/ attendees have completed VCT direct them to the Field Data Assistant (FDA) so that they can collect their incentive for participating in the survey.
30. The HIV VCT Results Form should then be submitted to the data section for storage.

MEMA kwa Vijana Trial Further Survey (2007-2008)

MkV1FS: Standard Operating Procedures for the SD-Bioline HIV 1 / 2 and Determine Rapid Tests

Applicable to: All personnel of the field team

Responsible person: Counsellors

Aim: To describe the procedure for performing the SD-Bioline and Determine HIV rapid tests.

Safety:

Treat all samples as potentially infectious.

Appropriate biosafety practices should be adhered to when handling specimens and reagents. These precautions include but not limited to the following:

- Wear gloves
- Do not pipette by mouth
- Do not eat, drink, smoke, apply cosmetics or handle lenses at the areas of work.
- Clean and disinfect all spills of specimens, reagent, blood and other potentially contaminated or infectious materials in accordance with local regulations.

NB: If at any stage the gloves get dirty or split, or you are worried that they might have been holed, you should replace them with a new pair. Any discarded pair of gloves must be put into a bucket that is partially filled with disinfectant.

Specimen collection

Whole blood collected by finger prick should be done aseptically.

A. Preparations:

1. Check the expiry date of the package. Record the expiry date and lot number on the results sheet for that day.

DO NOT use expired Kit.

2. Make sure you have:

(i). Alcohol/Spirit 70%

(ii). Cotton wool

(iii). Lancet

(iv). Bioline Assay diluent

(v). Heparinised capillary tube (80ul)

(vi). Timer

(vii). SD Bioline Test Device

(viii) Determine Test

(ix) Determine diluent

B. Performing the Test:

3. After pre-test counselling
4. Remove the test devise from the foil pouch
5. Place it on a clean, flat and dry surface
5. Explain to the client that there will be a small pain as the finger is pricked with a lancet.
6. Swab the client's finger with cotton soaked in alcohol, then allow to air dry
7. While waiting, remove the blade safety cover from the lancet
8. Hold the client's finger so that it is vertical
9. Prick the middle section of the finger and discard the lancet in the disposal bin
10. Gently squeeze the finger to allow blood to flow.

11. Place the unmarked end of capillary tube at right angle to allow enough blood to flow in to reach the red line. Avoid air bubbles.

Bioline

12 Add 10ul (about 1/8 of the total volume) of blood from the capillary tube to the sample well 'S' of the test device.

13. Add 4 (120µl) free falling drops of assay diluent onto the sample well (S) of the test device.
14. Set the timer
15. Wait for sample to move to the results window
16. **READ the results in 15 Minutes**

Determine

17. Add 50ul of blood from the capillary tube to the tissue of the test device.
18. Add 1 (30µl) free falling drop of assay diluent onto the tissue of the test device.
19. Set the timer
20. Wait for sample to move to the results window
21. **READ the results in 15 Minutes**

Interpretation of results:

Bioline

1. A purple colour band at the section marked ‘C’ of the device indicate the test is working.
2. A purple colour band on position 1 or 2 plus on ‘C’ indicate a reactive sample.

NEGATIVE results: One purple colour Band on the ‘C’ section of the test device only.

POSITIVE results: Purple Bands appear, on ‘C’ and 2 or ‘C’ and 1 or on ‘C’ and 1 and 2 sections of the test device.
Note: Presence of bands on position 1 or 2 indicate infection with HIV1 and HIV2 respectively.
INVALID results: No colour Band on the ‘C’ section of the test device.

Determine

1. A purple colour band in the reagent control of the device indicates the test is working.
2. A purple colour band on the client control indicates a reactive sample.

NEGATIVE results: One purple colour Band on the reagent control section of the test device only.

POSITIVE results: Purple Bands appear, on client control and reagent control sections of the test device.

INVALID results: No colour Band on the test device.

MEMA kwa Vijana Trial Further Survey (2007-2008)

Standard Operation Procedures for Survey Team Leader (STL)

1. Make sure that you have the survey timetable at least one week before the field trip.

2. One week before leaving for the field make sure that you have the correct and current version of both the **SOP** and the **Protocol**, read them carefully and follow the instructions while working/in the field.

3. One week before the field trip check that you, and all your team members, have all required equipment, documents and other supplies ***(Survey Team Packing Lists)***. If there are any problems with the equipment you must inform the Fieldwork Manager and/or Project Co-ordinator.

4. One week before departure, you should make sure that you have collected from the MO all important information on the community to be visited i.e. names of villages, details of survey locations, names of contact persons, details of accommodation.

5. Three days before departure you should contact the Census TL to ensure that the survey location and accommodation for the survey team are organized.

6. Two days before traveling, collect your imprests from the Project administrator.

7. The day before travelling you must supervise the packing of all the survey team equipment. **You are responsible for double-checking that each piece of electrical equipment is fully charged and/or has spare batteries.**

8. The day before travelling, you must arrange a briefing meeting with the Mobilisation Officer (MO), Field Supervisor (FS) and Fieldwork Manager AND the ST. During this meeting the MO will give you any important information about the community.

MEMA kwa Vijana Trial Further Survey (2007-2008)

9. On the day of departure, you will make sure that the ST leaves NIMR Mwanza early in the morning so as to arrive in the survey community (ward capital) in the afternoon/early evening. **All travel to the field must start at NIMR, Mwanza.**

10. The day you arrive you will meet with the census TL. The CTL will give you a brief update on the progress of the census and give you lists A1, A2 and A3 for the first village. The CTL will also give you the parent/guardian informed consent sheets for the invitees <18 years of age and any completed moved away forms.

11. Along with the CTL, you are responsible for negotiation with the village and sub-village leaders to ensure their active co-operation, and paying them as necessary for assistance.

12. On the day after arriving in the community (Day 1), your team will proceed to the survey venue in the first village. You will introduce yourself and your team to the venue owners and look at all the rooms in the venue. Then you will allocate rooms/areas to the different team members and ask the team to begin setting up their equipment.

13. While the team are setting up their equipment you should introduce yourself to the VEO, VC and the Vitongoji leaders in that Village and remind them of the length of time that the survey will be taking place in their village. You should request the Vitongoji leaders to mobilise the residents of their sub-village (house-to-house mobilisation) so that as many as possible of the invited young people attend the survey location.

14. On the day of the survey you must ensure that all survey team members are equipped to conduct their duties (suitably dressed, equipment charged etc). The work areas should be set up as follows:

    Registration area:

    - Registration Table(s) and chairs: Each RI will need a table for the registration documentation (one table for male attendees and one table

MEMA kwa Vijana Trial Further Survey (2007-2008)

for female attendees). Four chairs are needed (2 for RI and 2 for attendees during registration).

- Registration waiting area: Must be big enough to sit 30 people. Make sure that it is in the shade and if possible provide drinking water and magazines/newspapers.

NB All equipment including magazines/newspapers/games must be marked MkV1FS with a black marker

Survey Interview area (one for each SI):

- Two chairs must be placed facing each other. Each SI must ensure that it is not possible for anyone to listen to conversations that will take place in the survey interview area.

Laboratory area:

- Laboratory Table: Cover the table with the plastic bench cover and swab the cover with disinfectant. Lay out the equipment needed for taking and processing the blood and urine samples.
- The lab room must have a screened area so that the participants who are waiting cannot see you at work.

The VCT counsellor area:

- Two chairs must be placed facing each other. Ensure that it is not possible for anyone to listen to conversations that will take place in your counseling area.
- Set up your laboratory testing equipment on a table that has been covered with a plastic bench cover.

The Clinician area:

- Two chairs must be placed facing each other. Ensure that it is not possible for anyone to listen to conversations that will take place in your clinician area.

MEMA kwa Vijana Trial Further Survey (2007-2008)

- Set up the mattress and plastic sheet that will be used for examination on the floor or bed.

15. During the survey activities you must visit each of the team members to ensure that they are carrying out their tasks according to their SOPs.

16. You must pay particular attention to the informed consent procedures and assist the RI in explaining the survey procedures to the attendees.

17. You are responsible for ensuring that the survey activities proceed at the expected rate. If there is one activity which is taking longer than planned and hence slowing down the flow of participants then you must try to help the team members to speed up their work. It may be necessary to reallocate staff to help with the activity.

18. You are responsible for solving any other problems that occur during the course of the survey. If you are unsure about how to deal with a situation or need advice then you should contact the Field Supervisor (FS), Fieldwork Manager (FWM) or Project Co-ordinator (PC).

19. You should do the work of the Registration Interviewer, Lab Technician, Tracer or other staff person if one is sick or absent for any reason.

20. Any problems with the survey equipment must be recorded in the ***ST equipment notebook***. You are responsible for recording problems reported by the survey team members. If there is a need for replacement equipment then you must inform the FS, FWM or PC as soon as possible.

21. At the end of each day hold a debriefing meeting (approx. duration of 1 hour) with the survey team. Encourage them to share their experiences and discuss how the ST can improve their performance.

22. Each evening you should send a text update to the FS indicating how many eligible and non-eligible young people were interviewed that day etc.

23. At the end of each day compare the QC questionnaires to the main survey questionnaires. Discuss your findings with the SI the following day.

24. You must check the questionnaires and submitting them to the data section along with the ***Data Submission Form.***

25. When the survey is underway in the first village, visit the next village and introduce yourself to the village leaders (as in 11. above). Visit the survey venue in the next village and ensure that the venue is ready for the arrival of the survey team. Confirm the accommodation for the survey team.

26. Under normal circumstances the ST will not spend more than 3 days in each village.

27. At the end of survey activities in one village you will complete the survey village report form and submit this form during the next sample/data collection visit.

28. At the end of the survey activities in one community you will co-ordinate mop-up day(s) based on where the team is most likely to locate and survey most missing participants.

29. In Mwanza (within 2 days of return from the field), you must complete the following tasks:
    a. Arrange a debriefing meeting with the Field Supervisor (FS) or Fieldwork Manager or Project Co-ordinator AND census team members.
    b. Return all equipment / supplies to the project storeroom
    c. Submit all forms and questionnaires to the data section at NIMR.
    d. Complete all retirements required at NIMR office.
    e. Complete and submit to the Project Office the Survey Community Report form.

MEMA kwa Vijana Trial Further Survey (2007-2008)

MkV1 FS: Standard Operating Procedures for Drivers

1. Two days before travelling, collect your **imprest** from the Project administrator.

2. You must arrive at working place according to the official regulations reporting time (8:00am). Upon your arrival you must go to the MkV1 FS co-ordination office to indicate that you have arrived.

3. One week before leaving for the field make sure that you have the correct and current version of both the **SOP** and the **Protocol**, read them carefully and follow the instructions while working/in the field.

4. You have to make sure that the vehicle is kept safe and clean
   - ❑ Safety:
     - The drivers and all passengers must wear seatbelts
     - You must observe traffic regulations (e.g. driving speed should not exceed 100km/hr)
     - You must ensure that the vehicle is well packed and guarded properly
     - The car should not carry more than number of passengers allowed.
     - Non-staff passengers are not allowed to use the vehicle except in special cases. Transporting of non-staff passengers should never interfere with the project activities. Only the Team Leader (or supervisors) can make the decision about allowing non-staff to travel in the vehicle. Under this circumstance, the TL has to write a note for the event.
     - You must stay with your vehicle during repairs /services
     - The first AID kit must be in place and you are responsible for replacing informing the fieldwork manager about any items that need to be replaced.
     - The vehicle should be cleaned/washed once or twice a week when in field. Cleaning of the engine will be done when you return to Mwanza.

5. In the field you must handover the keys to the team leader in the evening after the working hours

MEMA kwa Vijana Trial Further Survey (2007-2008)

6. You must keep proper records on the vehicle, including filling in the logbook after every journey, keeping receipts for fuel and lubricants purchased for the vehicle, repairs, etc. You should let the Fieldwork Manager know when the vehicle is going to need its next service.

7. You must ensure that the vehicle has enough fuel for the journey and **when you refuel the vehicle you must fill both tanks**.

8. You should report to the supervisor/team leader any problem concerning with vehicle

9. You should follow all the instruction as assigned by the team leader, field supervisor, fieldwork manager and/or project co-ordinator. You should cooperate with all staff working on the project

10. While at the field site you should make sure that the vehicle is kept at the safe place (Team Leader must make decision regarding the car parking place).

11. The vehicle should only be used for official duties. If you are found to have been using the vehicle for private use, you will be fined TSh 50,000 and given a written warning on the first occasion, and will be fined TSh 50,000 and dismissed on the second occasion. The fines will be deducted from your monthly salary payments from the project. You must sign a letter agreeing to these terms before being issued with the vehicle.

12. You must also report any accidents to the Field Office immediately by:
    a. Phoning the office (PC, FC, FS or the Project Accountants).
    b. Sending a written report on the accident to the Project co-coordinator by the fastest possible means.

MEMA kwa Vijana Trial Further Survey (2007-2008)

MKV1 FS: Standard Operation Procedures for Field Supervisor

Preparation for Supervision

1. Make sure that you have the Teams timetable for the whole Month

2. Ensure that you have up-to-date copies of the **SOP** and packing list for all team members.

3. Look through the previous supervision reports to remind yourself of outstanding issues and procedures/staff members that you need to supervise closely.

Equipment

4. One week before the teams leave for the field, assist the TL and the MOs to get all required equipment, documents and other supplies (Team Packing Lists). Ask if there is any problem with the survey, Census and MO equipment and if they have recorded in the ST, CT and MO equipment notebook. If there is a need for replacement equipment then you have to report to the PC, FWM or FS, and return the broken equipment so that you are replaced with another one.

5. Before the teams leave for the field you will hold a briefing meeting with them and ensure that they are clear on the work procedures, equipment and documents. If possible arrange a briefing meeting with the Mobilisation Officer (MO), CTL, STL and Fieldwork Manager. During this meeting the MO will give you any important information about the community.

Vehicles

6. You must follow-up with the MOs and drivers to see that they keep proper records of the Motorcycles and vehicles, including filling in the logbook after every journey, keeping receipts for fuel and lubricants purchased for the motorcycles and vehicles, repairs, etc. Generators and vehicles should be serviced according to their service schedules.

Mobilisation- field supervision

7. Meet the MO and get the briefing of what is happening, go with him in the next mobilisation activities to see how he does job.

MEMA kwa Vijana Trial Further Survey (2007-2008)

8. Whenever possible visit with the MO the Proposed ST work site and accommodation for both CT and ST for the next community.

9. Visit some of the Vitongoji leaders/ Balozi in villages already mobilized to see that they are aware of the project and that they have the correct information.

10. Visit a random selection of households in areas that have been mobilised to see whether the household head has received the project information sheet.

11. At the end of your visit give the MO feedback, praising the good work and giving advice, if necessary, on how they can improve their mobilisation activities.

Census- field supervision

12. The day you arrive you will meet with the census TL. The CTL will give you a brief update on the progress of the census together with good things and challenges encountered. Meet also with the whole census team to hear how they have been getting on and to encourage them to continue their good work.

13. When in the field, go at least with two CIs in the field to observe how they do their work. You should randomly select the CIs that you will observe and you should not involve any staff including the CTL in this activity.

14. You should also make repeat visits to at least two HH from each category i.e. unknown, shifted, refused, absent and interviewed HH. Blind visits can also be made ie where you take only the name of the household head and do not know whether the household was interviewed, unknown, shifted etc.

15. Confirm if the completed moved away forms and parent/guardian IC sheets are submitted to the STL and then check if they are filled properly and match what CT reports.

MEMA kwa Vijana Trial Further Survey (2007-2008)

Survey Team Supervision

16. Check that all staff members
    a. have the correct version of the **SOPs** with them when they are working (Ask to see the **SOP**) and
    b. that they are following their **SOP** correctly. In particular you should check the following:

    Laboratory Technicians: follow the safety precautions in handling the urine and blood samples, registration book is completed correctly, time for leaving blood to clot

    Counsellors: seeking consent, time for Bioline and Determine Rapid HIV tests, registration book completed

17. Look through a random sample of questionnaires to check the quality and consistency.

18. Check the number of serum and the number of the main questionnaires if are the same.

19. At the end of your visit hold a meeting with the survey team and give them feedback on what they need to improve and congratulate them for the things they are doing well.

Back in Mwanza

20. Within 2 days of your return to Mwanza you should submit a supervision report to the PC. This report should be accompanied by any census or CI supervision forms used. In this report you should record reports from field staff and observations in the field. Decisions made in the field should also be documented in the supervision report for reference.

MEMA kwa Vijana Trial Further Survey (2007-2008)

21. When back from the field debrief with the other senior staff in the office. You are responsible for highlighting any outstanding issues and for ensuring that the relevant people have agreed to take follow-up action eg yourself, PC, FC etc

MEMA kwa Vijana Trial Further Survey (2007-2008)

MkV1 FS: STI Treatment SOP

(1) One week before departure make sure that you have information on the community that you will be visiting ie list of villages, sub villages, participants to be treated and contact details of community leaders. You should make a provisional fieldwork plan indicating the number of days that you will need to work in the community. This plan should be discussed and finalised with the FC/PC/FS at least 2 days be fore travelling.

(2) Ensure that all required field documents letters; equipments, drugs and other supplies are well-packed 2 days before STI treatment trip.

(3) **District Capital:** Travel to the District capital on your way to the community / ward pass the district capital and greet the DMO, DED and MkV2 TA. Discuss with the DMO that you are in the District to treat MkV1 FS participants who tested positive for NG, CT, and syphilis and who were not treated syndromically during the survey. Explain to the DED that you are in the District to follow-up with some clients who participated in MkV1 FS but do not specify that you will be treating clients for STI.

(4) **Ward capital:** On the same day report to the WEO

a. Explain that you are following up some clients who participated in MkV1 FS.
b. Do not specify that you are treating for STI but say that you are collecting some information from some of the clients that we already interviewed.
c. Ask the WEO to help you to identify the villages with health facility either a dispensary or health centre

(5) In each village:

a. Meet with the VEO and explain that you are collecting some extra information from clients that participated in MkV1 FS.
b. Visit the health facility (if one exists) and ask the staff if you can use the facility to treat clients for syphilis and other STI. Ask him/her to help you to find a room where you can see the clients.
c. Proceed to the first kitongoji where clients to be treated are living. Meet with the Kitongoji leader and explain that you have some things to discuss with clients that participated in MkV1 Fs. Ask the Kitongoji leader to help you to identify the households of the clients that you need to treat.
d. Visit the clients who are to be treated in the sub-village and at least 20% of YP who don't have to be treated so as to avoid identification and stigma. E.g. if you are to treat 30 YP then you need to visit extra 6 YP which makes a total of 36 YP.
e. When you have finished in one kitongoji proceed to the next kitongoji.

(6) At the household of client to be treated:

a. Confirm the identity of the client.

MEMA kwa Vijana Trial Further Survey (2007-2008)

b. If client is there then have a private meeting with the client and if they agree treat them as necessary for chlamydia or gonorrhoea OR invite them to the health facility to be treated for syphilis (see details below). If you invite a client to the health facility tell them that you will give them transport costs for the journey when they arrive at the health facility (up to a maximum of 2,000/= for a return journey). Give an appointment date and time after discussion with the client.
c. If the husband or wife of the STI positive client is at the household have an informed consent signed and then treat them. If one or both of them don't consent, give the client referral slips for partner (s) and explain where they can go for treatment.
d. If client is not present make an appointment to return to the household OR if this is not possible within the time that you have in the community then let them know when you will be in specific health facilities and invite them to come and see you there.
e. If client is travelling for some time and you will not be able to see him/her during your visit to the community you should leave a note for the participant and a referral slip for that client and request that they go to the health centre in the ward capital. Also leave referral slip at the clinic explaining the survey, diagnosis and date the specimen was taken.

<u>Treatment for STI</u>

Syphilis (NB TREATMENT AT HEALTH FACILITY ONLY)
- Injection Benz, Penicillin 2.4mu (1.2mu each buttock)
- If allergy to Penicillin refer to health facility specifying in referral slip that the client has tested positive for syphilis and reports an allergy to Penicillin.

Gonorrhea (If possible treat at HH if not possible then refer)
- Ciproflacin 500 mg stat
- If pregnant, breastfeeding or refused pregnancy test: Ceftriaxone 250mg im, stat

Chlamydia (If possible treat at HH if not possible then refer)
- Doxycyline 100mg bd x 7 days
- If pregnant, breastfeeding, refuses pregnancy test or side-effects: Erythromycin 500mg qds 7 days

- Ensure Compliance
- Provide Health Education
- Counsel
- Record no of Contact (s)
- Initiate Contact (s) Refaral
- Promote and Provide Condoms
- Advise no sex until they have completed the treatment and their partner(s) has been treated.

MEMA kwa Vijana Trial Further Survey (2007-2008)

(7) At the health facility:

a. Welcome the client and confirm their identity.
b. Explain to them that they need to be treated for Syphilis (and/or other STI).
c. If treating for Syphilis with Benz Penicillin ensure that you or health facility staff are ready to treat with adrenalin if client has allergic reaction.

(8) Before leaving the community visit the WEO to let him know that you have finished your work for now.

Back in Mwanza

(9) One day after return to mwanza you should meet with FS/PC and debrief them about your visit to the community.

(10) You must submit your retirement to NIMR office on the day after you return to mwanza.

(11) You must produce and submit the STI treatment report to the PC within 3 days of your return to mwanza.

Motorbike

(12) You will be issued with a project motorbike for your work. You must keep proper records on the motorbike, including filling in the logbook after every journey, keeping receipts for fuel and lubricants purchased for the bike repairs.

(14) You must ensure regular maintenance of the motorcycle and provide a written report to the field coordinator after each trip and help him to arrange the repairs.

(15) The motorbike should only be used for official duties. If you are found to have been using the motorbike for private use, you will be fined Tsh50, 000 and given a written warning on the first occasion. You will be fined Tsh50, 000 and dismissed on the second occasion. The fines will be deducted from your monthly salary payments from the project. You must sign a letter agreeing to these terms before being issued with the motorbike.

(16) You must observe traffic regulations (e.g. speed restrictions, you must wear a safety helmet and other safety gear).

(17) You must not drive the motorbike if you have consumed any alcohol within the previous 8 hours.

MEMA kwa Vijana Trial Further Survey (2007-2008)

(18) You must also report any accidents to the field office immediately by:
   (a) Phoning the office (PC, FC, FS)
   AND
   (b) Sending a written report on the accident to the project co-ordinator by the fastest possible means.

MEMA kwa Vijana Trial Further Survey (2007-2008)

MkV1 FS: STI Treatment post-survey SOP

A. Preparation

(1) Make sure that you have information on the community that you will be visiting ie list of villages, sub villages, participants to be treated (list from data section) and contact details of community leaders (survey mobilisation information).

(2) If the community has been visited before for STI treatment (by Clinician or during mop-up) then cross-check lists of names/sticker numbers to be sure that clients have not already been treated or traced.

(3) Look at registration book to get names and contact details of clients who were not invited by census team ie list produced by data will only have their sticker number.

(4) You should make a provisional fieldwork plan indicating the number of days that you will need to work in the community. This plan should be discussed and finalised with the FC/PC/FS at least 2 days be fore travelling.

(5) Ensure that all required field documents letters; equipments, drugs and other supplies are well-packed 2 days before STI treatment trip.

B. Mobilisation

(6) **District Capital:**

a. Travel to the District capital on your way to the community / ward pass the district capital and greet the DMO, DED and MkV2 TA.

b. Discuss with the DMO that you are in the District to treat MkV1 FS participants who tested positive for NG, CT, and syphilis and who were not treated syndromically during the survey.

c. Explain to the DED that you are in the District to follow-up with some clients who participated in MkV1 FS but <u>do not specify that you will be treating clients for STI.</u>

(7) Ward capital:

a. Meet with the WEO and explain that you are following up some clients who participated in MkV1 FS.

b. <u>Do not specify that you are treating for STI</u> but say that you are collecting some information from some of the clients that we already interviewed.

c. Ask the WEO to help you to identify the villages with health facility either a dispensary or health centre

MEMA kwa Vijana Trial Further Survey (2007-2008)

d. Visit the main health facility at the ward capital and meet with the doctor/nurse who is responsible for treatment of STIs. Explain that you may refer some clients to their health facility for treatment and give example of ***MkV1 FS client referral slip*** and ***MkV1 FS contact referral slip***.

(8) In each village:

a. Meet with the VEO and explain that you are collecting some extra information from clients that participated in MkV1 FS. Do not specify that you are treating for STI.

b. Visit the health facility (if one exists) and ask the staff if you can use the facility to treat clients for syphilis and other STI. Ask him/her to help you to find a room where you can see the clients.

c. Give names of those to be traced to the tracers. Tell them that these clients are on **List A.**

Treatment for STI

(9) At the health facility:

a. Welcome the client and confirm their identity.

b. Explain to them that they need to be treated for Syphilis, Chlamydia and/or Gonorrhoea.

c. If treating for Syphilis with Benz Penicillin ensure that you or health facility staff are ready to treat with adrenalin if client has allergic reaction.

d. Treat the client according to the National Guidelines for STI treatment (see box below).

e. Fill out a ***Treatment after Lab results form*** for each client treated.

f. Treat any partner of client who attends for STI treatment. Fill out a ***Treatment after lab results form*** for the partner and indicate at the bottom of the form the sticker number of the client (ie the sticker number of the person traced and treated).

g. Fill out referral forms for any partners that the client mentions. **(USE MKV1FS CONTACT REFERRAL SLIP).**

Revisit to WEO

h. Before leaving the community visit the WEO to let him know that you have finished your work for now.

MEMA kwa Vijana Trial Further Survey (2007-2008)

Revisit to Ward Health Facility

i. Revisit main health facility in the ward and leave a list of names of those who tested positive for a STI and who you did not manage to treat during the visit to the community ie those who were given MkV1 FS client referral slips.

Back in Mwanza

(10) One day after return to mwanza you should meet with FS/PC and debrief them about your visit to the community.

(11) You must submit your retirement to NIMR office on the day after you return to mwanza.

(12) You must produce and submit the STI treatment report to the pc within 3 days of your return to mwanza.

<u>Treatment for STI</u>

Syphilis (NB TREATMENT AT HEALTH FACILITY ONLY)
- Injection Benz, Penicillin 2.4mu (1.2mu each buttock)
- If allergy to Penicillin refer to health facility specifying in referral slip that the client has tested positive for syphilis and reports an allergy to Penicillin.

Gonorrhea
- Ciprofloxacin 500 mg stat
- If pregnant, breastfeeding or refused pregnancy test: Ceftriaxone 250mg im, stat

Chlamydia
- Doxycyline 100mg bd x 7 days
- If pregnant, breastfeeding, refuses pregnancy test or side-effects: Erythromycin 500mg qds 7 days

- Ensure Compliance
- Provide H/ Education
- Counsel
- Record no of Contact (s)
- Initiate Contact (s) Referral
- Promote and Provide Condoms
- Advise no sex until they have completed the treatment and their partner(s) has been treated.

MEMA kwa Vijana Trial Further Survey (2007-2008)

MkV1 FS: Follow-up of HIV results SOP

You will revisit clients who had an initial discordant VCT result and give them the NIMR HIV result ie the result based on analysis of their serum sample at NIMR using ELISA (the samples of some clients were also analysed using p24 and Western Blot).

You will also revisit clients if the VCT result does not match the NIMR HIV result.

A. Preparation (Counsellor/ Clinician)

(1) Make sure that you have information on the community that you will be visiting ie list of villages, sub villages, contact details of community leaders (survey mobilisation information).

(2) You will receive a list of sticker numbers form the data section. For each client you will be given the initial VCT result and the NIMR result.

(3) For each client cross-check the VCT results from the data section with the VCT results in the counsellor notebook. **If these do not match then please contact the Project co-ordinator or Field supervisor.**

(4) You should make a provisional fieldwork plan indicating the number of days that you will need to work in the community. This plan should be discussed and finalised with the FC/PC/FS at least 2 days before travelling.

(5) Ensure that all required field documents letters; equipments, test kits, serum/EDTA tubes and other supplies are well-packed 2 days before field trip.

B. Mobilisation (Counsellor/ Clinician)

(6) Your visit is likely to take place at the same time as revisits to treat clients for STI (see SOP for STI treatment post survey) and you will not need to make any specific visits to the District, Ward Capital or village leaders.

(7) If STI treatment is not taking place in that ward/ village then you should meet with the WEO and then the VEO and explain that you are collecting some extra information from clients that participated in MkV1 FS. <u>Do not specify that you are following up clients who had VCT.</u>

C. Tracing (Tracers, Driver)

(8) Give the names of clients in **Category 1 (c), 2, 3 and 4** to the tracers working with you on your team. Tell them that the names of these clients are on **List B**. They will invite clients to see you at the health facility.

(9) Clients in **Category 1(a) and 1 (b)** will not need repeat testing and will not need to visit the health facility. You will be able to visit these clients yourself at their households and give them the final NIMR result and carryout post-test counselling.

MEMA kwa Vijana Trial Further Survey (2007-2008)

D. Retesting for HIV (Counsellor/ Clinician)

(10) At the health facility:

a. Welcome the client and confirm their identity.

b. Explain to them that we have further information for them about their VCT HIV test results.

c. Look carefully at the information provided by the data section and decide which category the client belongs to. Carry out the procedures described below for that category.

d. If you repeat VCT then you should repeat the pre and post test counselling and following the testing procedures as described in the **SOP for VCT counsellor.**

e. Take the printed stickers that correspond to the original sticker number of the participant. Place one sticker in your notebook and one sticker on the follow-up HIV results form. Place one sticker on the consent for VCT form.

f. If you need to take a blood sample then follow the following procedures.

Collection of Purple topped EDTA tube sample:

g. Place one sticker on the sample submission form.

h. Label with stickers two EDTA purple topped 5mL vacutainer tubes. Ensure the stickers are sealed with sellotape.

i. Explain to the client that you are going to collect blood by venepuncture.

j. Explain to him/her that there will be a small pain as the needle is inserted into the vein.

k. Apply a tourniquet above the elbow until the vein is raised.

l. Remove the sheath from the lower part of the needle and fit it on the holder and then place the needle into the holder.

DO NOT PUSH THE VACUTAINER TUBE COMPLETELY ONTO THE NEEDLE AS THE VACUUM MIGHT BE LOST.

m. Swab the skin using a mediswab.

n. Remove the upper vacutainer needle cover, push the needle into the vein until you are sure it is in and then push the vacutainer tube completely into the needle.

o. Watch the blood flow in the vacutainer until it nearly reaches the top.

MEMA kwa Vijana Trial Further Survey (2007-2008)

p. Remove the vacutainer and attach another vacutainer.

q. Watch the blood flow in the vacutainer until it nearly reaches the top.

r. Release the tourniquet and then remove the needle. Put cotton wool soaked in spirit over the puncture mark and ask the client to press on the cotton wool.

s. Unscrew the needle from the holder and dispose of in a safe container/disposal bin.

t. You need to mix the EDTA in the tube with the blood that is collected by carefully turning each tube up and down 6 times. **NB DO NOT SHAKE**.

u. When you have mixed the contents of the vacutainer tube then store the tube between +4° to +25° degrees Celsius. **DO NOT FREEZE.**

v. **All EDTA tubes must be returned to Mwanza within 4 days of collection.** At the end of every community (~ every 3 days) one team member will travel by public transport to Mwanza carrying the samples in a carrier bag with rack for tubes inside. This bag should contain some ice packs. Samples should be brought to NIMR during working hours.

Back in Mwanza

(11) One day after return to mwanza you should meet with FS/PC and debrief them about your visit to the community.

(12) You must submit your retirement to NIMR office on the day after you return to mwanza.

(13) You must produce and submit the STI treatment report to the PC within 3 days of your return to mwanza.

MEMA kwa Vijana Trial Further Survey (2007-2008)

<u>Category 1. Initial VCT was **discordant**</u>

(a)Initial VCT discordant- final NIMR **positive**

- Tell the client that we have the NIMR result and that they are HIV positive

(b) Initial VCT discordant- final NIMR **negative**

- Tell the client that we have the NIMR result and that they are HIV negative

(c)Initial VCT discordant – final NIMR **indeterminate (not positive, not negative)**

Repeat VCT:

- If repeat VCT is positive then tell client that they are positive.
- If repeat VCT is negative then tell client that they are negative.
- If repeat VCT is discordant then tell client that it is not possible to know if they are positive or negative and that they should retest themselves after 3 months.

For all clients request the client to provide another blood sample. This blood sample will help us with our research. If they agree then collect **two EDTA tubes** of blood.

<u>Category 2. Initial VCT **positive**- NIMR result **negative**</u>

Repeat VCT.

- If repeat VCT is negative then tell client that they are negative and that their first VCT was a false positive.
- If repeat VCT is positive then tell client that they are positive.
- If repeat VCT is discordant then tell client that is not possible to know if they are positive or negative and that they should retest themselves after 3 months.

If repeat VCT is positive or discordant then request the client to provide another blood sample. This blood sample will help us with our research. If they agree then collect **two EDTA tubes** of blood.

MEMA kwa Vijana Trial Further Survey (2007-2008)

Category 3. Initial VCT **negative**- NIMR result **positive**

Repeat VCT.

- If VCT is negative then tell client that they are negative.
- If VCT is positive then tell client that they are now positive and their first VCT was a false negative.
- If VCT is discordant then tell client that is not possible to know if they are positive or negative and that they should retest themselves after 3 months.

If repeat VCT is negative or discordant then request the client to provide another blood sample. This blood sample will help us with our research. If they agree then collect **two EDTA tubes** of blood.

Category 4. Initial VCT **positive**- NIMR result **indeterminate**

Repeat VCT.

- If VCT is negative then tell client that they are negative and that the first VCT result was false positive. Recommend that they get retested after 3 months.
- If VCT is positive then tell client that they are positive.
- If VCT is discordant then tell client that is not possible to know if they are positive or negative and that they should retest themselves after 3 months.

For all clients request the client to provide another blood sample. This blood sample will help us with our research. If they agree then collect **two EDTA tubes** of blood.

MEMA kwa Vijana Trial Further Survey (2007-2008)

MkV1 FS: Tracing Post-survey SOP

a. The clinician and/or counsellor will give you names of clients to be traced. Write these names onto your tracing form.

b. The clinician and/or counsellor will tell you if these clients are on list A or list B. **Write A or B beside the name of the client on your tracing form.**

c. It is very important that no one in the communities that you are visiting know that you are following up young people who have laboratory results that we need to discuss with them.

d. Meet with the Kitongoji leader and explain that you have some things to discuss with clients that participated in MkV1 FS. Ask the Kitongoji leader to help you to identify the households of the clients that you need to invite to the health facility.

e. At the household, confirm the identity of the client.

List A clients:

a. If client is there then have a private meeting with the client and explain that we would like them to attend at the health facility.

b. Remind them that we promised to return and treat clients who tested positive for syphilis, Chlamydia or gonorrhoea.

c. Give an appointment date and time after discussion with the client (**MkV1 FS CLIENT INVITATION SLIP**).

d. Tell them that you will give them transport costs for the journey when they arrive at the health facility (up to a maximum of 2,000/= for a return journey).

e. Tell them that their partner (s) can attend with them and if they do so then they will also benefit from free treatment for STIs (if necessary).

f. If client is **NOT** there then leave invitation for them to come and see you at the health facility **(MKV1 FS CLIENT INVITATION SLIP)**.

g. If client is travelling and you will not be able to see during your visit to the community you should fill out a **MOVED AWAY FORM** for the client. Leave a referral slip for that client and request that they go to the health centre in the ward capital **(USE MKV1FS CLIENT REFERRAL SLIP).**

h. Before leaving the household indicate one your tracing form if the client was found and given invitation. If you filled out a moved away form or client referral slip then please indicate this on the tracing form.

MEMA kwa Vijana Trial Further Survey (2007-2008)

List B clients:

a. If client is there have a private meeting with the client and explain that we would like them to attend at the health facility.

b. Remind them that we are returning to clients who had a VCT HIV result that was not clearly positive or negative OR where the result of VCT is different from the result found at NIMR.

c. NB Bring the client immediately with you to the health facility.

d. If they are not able to go with you immediately then give an appointment date and time after discussion with the client (**MkV1 FS CLIENT INVITATION SLIP).**

e. Tell them that you will give them transport costs for the journey when they arrive at the health facility (up to a maximum of 2,000/= for a return journey).

f. If the client is **NOT** there then make an appointment to return to the household and leave a message saying when you will be in specific health facilities and invite them to come and see you there **(MKV1 FS CLIENT INVITATION SLIP)**.

g. Before leaving the household indicate one your tracing form if the client was found and given invitation. If you filled out a moved away form then please indicate this on the tracing form.

MEMA kwa Vijana Trial Further Survey (2007-2008)

MKV1 FS: Standard Operation Procedures for Census Checker (CC)

1. Make sure that you have the **census timetable** at least one week before the field trip.
2. One week before leaving for the field make sure that you have the correct and current version of both the **SOP** and the **Protocol**, read them carefully and follow the instructions while working/in the field.

3. One week before the field trip check that you have all required equipment (e.g. PDAs, GPS), documents and other supplies ***(Census Team Packing List)***. If there are any problems with the equipment then inform the CTL.

4. Two days before travelling, collect your **imprest** from the Project administrator.

5. The day before travelling:

    a. Pack all equipment/supplies that you need. **All PDA and laptops (including spare batteries) must be fully charged.**
    b. Attend the CT briefing meeting with the Mobilisation Officer (MO), Field Supervisor (FS) and Fieldwork Manager AND the rest of the CT. During this meeting the MO will give you any important information about the community.

6. On the day of departure, you will leave NIMR, Mwanza early in the morning so as to arrive in the survey community (ward capital) in the afternoon/early evening. **All travel to the field must start at NIMR, Mwanza.**

7. The day after arriving in the community (Day 1) you must:

    a. help the CTL with preparations for the census.
    b. Pack your bag for Day 2, double-checking that all equipment is working and that you have the necessary forms for your work the following day. Where possible carry with you extra copies of necessary forms and sheets.

MEMA kwa Vijana Trial Further Survey (2007-2008)

c. Attend the briefing meeting that the CTL will hold at the end of Day 1 and carefully note the kitongoji and CH assigned to you.

8. **You have to revisit at least 10% of all HH visited/interviewed by each CI in each village.**
9. **From day 2, each evening after the CIs have come from the field, collect the CI forms and identify the HH you will work on the following day by writing them on the new CI form. This should be done without involving any of CIs.**

10. Using your ***CI form for HH head***, travel with the CH from household to household on bicycle or on foot. At each household introduce yourself and the purpose of your visit to the household members. If the household head (or another adult household member) is present then read the information sheet to them and answer any questions that they have. **Read out loud the information on the HH informed consent sheet to the household member before asking them to sign the HH consent sheet.** If the household head does not consent to participate in the census then thank the household head and move to the next household. If the household head is absent then another adult member of the household may give consent to participate. Complete the first part of the census form on the PDA.

11. If there is nobody at the household when you visit then enter 2 = “absent” on the column of census status for this household. Make a maximum of two further attempts to visit that HH later in the day (visits on the same day must be at least 4 hours apart) or on the next day and complete the columns of census status for this household. Record the date and time of each visit (Swahili time) on your **CI form for HH head**. If possible, leave a message with the neighbours to say that you will be returning at a specified time. **The same CI form for HH head will be used for all days in the same kitongoji.**

MEMA kwa Vijana Trial Further Survey (2007-2008)

**Codes for CI form**	
**1**	**Interviewed**
**2**	**Absent**
**3**	**HH Refused**
**4**	**Repeated HH**
**5**	**HH not Known**
6	**HH Moved away**

12. If there are any young people aged 15-30 years living in the household then they should respond to the questions in the PDA that relate to them.

    a. If the **HH were not interviewed by the CI** and you find the young person then ask the household head (and other HH members) to provide the necessary information about the young person. Give all eligible **young people you find in the HH which were not interviewed** a survey invitation by writing on a **sticker note (not a green survey invitation)**.
    b. If the young person is not present then leave a survey invitation at the household.
    c. If the eligible young person will not be able to attend the survey because they are travelling for a long period of time/ have moved away to study etc. then fill out the ***moved away form*** but still leave an invitation. Indicate on the CI form for HH head that you have left a moved away form (Please write **M** to the left of the name of the Household head on the CI form). **You must submit used moved away forms to the CTL at the end of each day.**

13. If the eligible young person is aged 17 years or younger then you must leave a copy of the ***Information sheet for parent(s)/guardian(s)***. You must also leave an ***Informed consent sheet for parent(s)/guardian(s)***. If the parent/guardian is present then ask them to sign the informed consent sheet (read first to them the information on the IC sheet). Signing of the IC sheet must be witnessed by the kitongoji leader or the community helper (must also sign the form). If the parent/guardian is not present or will not immediately sign the IC sheet then inform the HH members that the invited YP who is <18 yrs of age must take the

survey invitation **AND** the IC sheet to the survey. **You must submit completed IC sheet for parent/guardian to the CTL at the end of each day.**

14. You should use the ***calendar of events*** to help the respondents recall dates.

15. At the end of the day:
    a. Attend the daily debriefing meeting and share your experiences with the rest of the team.
    b. By using rotary method select the CI whose previous HH you will visit the next day, one CI will pick the piece of paper on which the staff code of the CI is written.

16. When requested you must assist the CTL with other census tasks e.g. synchronisation, burning of CDs, generation and printing of lists.

17. In Mwanza (within 2 days of return from the field), you must complete the following tasks:
    a. Attend the CT debriefing meeting
    b. Return all equipment to the project storeroom
    c. Complete and submit all report and retirements required at NIMR office.

18. PDA use
    a. All text must be entered in capital letters
    b. Avoid entering other characters by accident e.g. '.' or ','
    c. GPS coordinates must be entered with 5 numbers after the decimal point i.e. ##.#####

MEMA kwa Vijana Trial Further Survey (2007-2008)

SOP for Tracing during Mop-up
DRAFT 06 June 08

There are 3 pieces of information that we will use during Mop-up:

A. List of households not interviewed
B. List of invited young people who did not attend
C. Master list of all those who did not attend including those not invited

A. Households not interviewed	B. Young people **invited** but did not attend	C. **MASTER LIST** All young people who did not attend- **invited** and **not invited**

The Team Leader will assign you to one or more kitongoji.

1. **Households:**
   (i) Write head of household names from the **'mop-up list of households'** onto your **CI form**.
   (ii) Visit all households on your CI form and complete the PDA/back-up census questionnaire.
   (iii) If a potentially eligible young person is identified (and has not already attended) then you should give them an invitation to attend the survey site. Try to bring them immediately to the survey site and/or make an appointment for the vehicle to collect them.
   (iv) Fill out a moved-away form if the young person is absent.

2. **Young People:**
   (i) Transfer names of young people to be traced from the **Master List** and/or the **List of invited not attended (L1/L2)** onto your **tracing form**.
   (ii) Visit the households of the young people on your tracing form.
   (iii) If you find a young person then invite them to the survey site. Try to bring them immediately to the survey site and/or make an appointment for the vehicle to collect them.
   (iv) Fill out a moved-away form if the young person is absent.

NB Completion of moved-away forms

- Ensure that you write the sex of the client (on the top right of the page) or in the space provided
- Ensure that you write the name of the husband/wife if the client is married
- For females write the name of the first child if she has had any children
- Get as detailed information as possible on their current location including a mobile phone no.

NB Prioritisation of females

- First you should try to trace females. If you are close to the household of a male then you should also trace the male living in that household.

MEMA kwa Vijana Trial Further Survey (2007-2008)

MkV1 FS: Standard Operating Procedures for Mobilisation Officers (MO)

Preparations.

1. One week before leaving for field, make sure that you have the correct and current version of both SOP and the protocol, read the carefully and follow the instructions while working/in the field.
2. Two days before travelling, collect your imprest from the Project administrator.
3. Ensure that all required field documents/letter, equipment and other supplies are well-packed 2 days before mobilisation trip.
4. When you reach at the ward capital, visit the ward official and explain to them that we are in the ward for a few days to do mop-up for the YP we didn't reach during the main survey.
5. Visit the village officials and inform them of the team revisiting the village for mop-up.
6. Book accommodation for the team for at least three days.
7. Find the survey site in the ward main village and make an agreement and sign a contract.
8. Visit the Primary schools in the Ward and ask the Head Teacher to identify the names of the HH against the list of YP and seek information of the students who completed Primary schools in 2004 and joined Secondary School. Identify the Sec. Schools they joined.
9. Ask the village leaders to help you to identify the names of the HH of some of the YP who miss the names of the head of the HH.
10. One day after you return to Mwanza, you should meet with the FS & FM and debrief them about your visit. It is your responsibility to arrange this meeting.
11. You must submit your retirement to the NIMR office on the day after you return to Mwanza.

Motorbike

12. You will be issued with a project motorbike for your work. You must keep proper records on the motorbike, including filling in the logbook after every journey, keeping receipts for fuel and lubricants purchased for the bike, repairs, etc.

MEMA kwa Vijana Trial Further Survey (2007-2008)

13. You must ensure regular maintenance of the motorcycle and provide a verbal report to the Fieldwork Manager after each trip and help him to arrange the repairs.
14. The motorbike should only be used for official duties. If you are found to have been using the motorbike for private use, you will be fined TSh 50,000 and given a written warning on the first occasion, and will be fined TSh 50,000 and dismissed on the second occasion. The fines will be deducted from your monthly salary payments from the project. You must sign a letter agreeing to these terms before being issued with the motorbike.
15. You must observe traffic regulations (e.g. speed, you must wear a safety helmet and other safety gear).
16. You must also report any accidents to the Field Office immediately by:
    a. Phoning the office (AD, KM, LM or the Project Accountants).
    b. Sending a written report on the accident to the Project co-ordinator by the fastest possible means.

MEMA kwa Vijana Trial Further Survey (2007-2008)

SOP for Preparation of Lists for Mop-up
06 June 08

There are 3 pieces of information that we will use during Mop-up.

A. List of households not interviewed (will be prepared in the office if possible)
B. List of invited young people who did not attend (prepared in the field)
C. Master list of all those who did not attend including those not invited (prepared in the field)

A. Households not interviewed	B. Young people **invited** but did not attend	C. **MASTER LIST** All young people who did not attend- **invited** and **not invited-**

A. Lists of Households to be visited

Needed: CI form, Master list of HH heads, blank mop-up household head list

1. Choose a Kitongoji.
2. Compare the Master list of HH heads to the CI form to see that every household has been listed on a CI form. If there are households that were not on CI form then put on mop-up list of household head.
3. Look at CI form to see which HH were absent on last visit ie ‘2’. List these households on the mop-up list of household head.
4. Repeat procedure for next kitongoji.

B. List of Invited and didn’t attend

Needed: List of invited not attended (L1 males and L2 females), list A1 and A2, lists L11 and L12, Tracing forms, Highlighter pens

1. Choose a village.
2. Obtain list L1-L2 (list of young people who were invited but who didn’t attend) from data section
3. Cross-check list L1-L2 against list A1-A2 for that village to see if any of the young people have attended (use name and census ID no.). If young person has already attended then write **‘ATT’** in front of her name and write the sticker number beside her name.
4. Take tracing forms for that village and check if any young people on list L1-L2 have already refused to attend. If these refused then place ‘**R’** in front of their name.
5. **Now highlight the names of any young people who we need to trace ie have <u>not already attended</u> and <u>not already refused</u>**
6. *(**Team Leader/ Deputy Team Leader:** If you find a young person on list L1-L2 who has already attended then find his/her sticker number on list L11-L12 (no merge) and write the correct census ID number and his/her name on list L11-L12.)*
7. Repeat steps 1-6 for next village

C. Master list of all potentially eligible

Needed: Master list of potentially eligible (male, female), List of invited not attended (L1-L2), lists of attended (L3, L4, L5, L6, L9, L10), Registration books for communities to be visited, highlighter pens.

1. Choose a school.
2. Obtain Master list of potentially eligible from office.
3. Take list of uninvited attended (L9, L10) and use registration book for the village that you are interested in and fill in names that match the sticker number (use village attended on L9/L10).
4. Cross-check against Master List to see if young person has already attended survey or has already been invited using all of the following lists:
    a. L3-L4, invited in community but attended survey anywhere (use school on L3-L4)
    b. L5-L6, school in community but invited anywhere (use school on L5-L6)
    c. L9-L10, uninvited but attended (use school on L9-L10)
    d. L1-L2, invited but didn't attend (use school on L1-L2)
5. If a name is on the Master list and the young person has attended already then tick column 'attended' (old version of Master list) OR enter sticker number (new version of master list).
6. If young person has been invited then enter Census ID no on Master List.
7. If young person has already refused then tick the box 'Refused?'.
8. If a young person has attended but is not on the Master list for their school then write their name and sticker number and census ID no at the end of the Master list.
9. If the teachers have provided the names of young people still living in the community then tick the box 'Still in cmty?'.
10. **Now highlight the names of all young people who we need to trace ie have <u>not already attended</u> and not already <u>refused</u>.**
11. If the teachers have
12. Repeat steps 1-9 with another school.

District Mobilization Forum

Potential Participants from District

1. District Commission
2. District Administrative Secretary
3. District Executive Director
4. Chairperson- District council (mayor)
5. District Planning Officer (1 person)
6. District Medical Officer
7. District Education Officer
8. District Community Development Officer (1 person)
9. District AIDS Coordinator & CAC (2 persons: 1from Health Dept.& 1 from Community Dept.), DTS (1 person), 12. Chief District School Inspector
13. Technical Assistant (AMREF district based staff)

Tentative Schedule

Time	Activities	Activity modality/format	Required reference	Responsible Person(s)
1100HRS TO 1145HRS	Arrive to the forum venue	Individual transport		ALL
	Open remark			DC
	Introduction	Participants to introduce his/her names, title and or Dept.		DED/MH
	Brief explanation of MKV1FS:<br>• Purpose<br>• Objectives<br>• Importance<br>• Ethical approve	Normal presentation	History of MEMA kwa Vijana. Community Information Sheet.	AD/DR
1145HRS TO 1300HRS	Survey procedures:<br>• Preparation visit<br>• Census<br>• Main survey	Normal presentation	Community Information Sheet	AD/MH
	• Confidentiality<br>• Activity at NIMR Mwanza	Normal presentation	Community Information Sheet	AD/MH
1300HRS TO 1400HRS	• **Lunch Beak**			ALL
1400HRS TO 1600HRS	• Discussion	Questions and answers		ALL
1600HRS	• Close			DED/MH

MEMA kwa Vijana Trial Further Survey (2007-2008)

MkV1 Further Survey- Order of Communities

Community name & number		Timing of Survey activities (approx.)	
**Team 1**	**Team 2**	**2007**	**2008**
Katunguru (5)		June	
Kasamwa (11)	Koromije (22)	July	
Nyang'wale (14)	Mwagi (23)	August	
Bukoli (13))	Malya (24)	September	
Katoro (8)	Misasi (18)	October	
Kagu (9)	Usagara (21)	November	
Lubanga (10)	Nyakaliro (4)	December	
	Katwe (2)		February
Busisi (6)	Nyehunge (3)		March
Nkome (1)	Fukalo (17)		April
Ihanamilo (12)	Hungumalwa (16)		May

Note: Order of Communities Slightly changed comparing to the (MkV final Survey 2001/2002)

MEMA kwa Vijana Trial Further Survey (2007-2008)

Information Sheet for Officials & Community Members

The MEMA kwa Vijana Programme is a collaborative project of the government of Tanzania (the Ministry of Health & Social Welfare and the Ministry of Education & Vocational Training), the National Institute for Medical Research (NIMR), AMREF, the London School of Hygiene and Tropical Medicine, UK (LSHTM) , the Liverpool School of Tropical Medicine (LSTM) and MRC Social and Public Health Sciences Institute, Glasgow, UK . The programme is working in four districts of Mwanza Region (Misungwi, Sengerema, Geita, and Kwimba). The programme supports improved treatment of sexually transmitted diseases in health facilities, provides additional training for health workers, and provides sexual and reproductive health education to primary school pupils.

During 2007 and 2008, the programme will conduct a survey to assess the impact of the intervention '***MkV1 Trial Further Survey'***. From previous surveys that were conducted between 2000 and 2002, we already know that the MEMA kwa Vijana intervention improved the knowledge and some reported sexual behaviours in young people in your community. This further survey will tell us if these improvements have been sustained, and whether the intervention has been effective in reducing the spread of HIV and other sexually transmitted infections in the longer-term. The information that we collect will help us to find the best ways to reduce the spread of HIV and other sexually transmitted diseases in your community.

Survey Procedures:
The survey will have two parts: (1) a census of all households in the selected wards and (2) the main survey in which young people who are identified during the census will be interviewed. With help of a Project Information Officer and local leaders, information on the days of the census, as well as information on the purpose and procedures of the census and main survey, will be sent in advance to each of the sub-village leaders.

First activity (Pre-survey and preparation for census)
The MEMA kwa Vijana Project's Information Officer will visit each survey community before the census team. He will meet with district, ward, village, and sub-village leaders and explain to them the importance, purpose and timing of the census and main survey, ask for their co-operation and request permission to proceed. Each sub-village leader will be asked to help with the census by providing a list of all the households in their sub-village. This list can be compiled with the assistance of other leaders in the sub-village. They will also be asked to indicate whether there is any young person between the ages of 15 and 30 years who is currently resident in each of these households. The information officer will provide a special form for this list and will indicate the day that he will return to collect the completed list of households.

Second activity (Census)
The census team will arrive in each community. They will have obtained the list of households from the Project Information Officer. With the help of the sub-village leaders, team members will visit each household in the sub-village. They will provide the most senior adult in the household verbal and written information about the survey and obtain their permission for the household to participate in the census. This "household head" will then be asked some questions about the sex and age of all those currently living in the household. This information will be entered directly into a tiny computer. If there is any young adults aged 15-30 years living in the household, further questions will be asked to check their age and attendance at primary school in order to identify if they are eligible to participate in the main survey. Only those who attended certain years of primary school will be eligible.

The census worker will give all young adults who are eligible for the main survey an invitation to attend a specific survey point (eg. a house or guesthouse) in the village 2–3 days later. They will also be provided with a special information sheet that will explain the purpose and procedures of the survey in more detail. They will be told that they will be given a small gift to compensate them for travelling to the survey point, and for giving up some of their time. If the

young person is aged 17 years, an information sheet will be left for their parent(s) or guardian(s). As well as explaining the purpose and procedures of the main survey, this will ask them to instruct the young person not to attend the main survey if they do not want them to. If there are no adults present when a household is visited, the census worker will make up to two further visits to the household. If the eligible young person is temporarily absent, the household head will be requested to give the invitation and information sheet to them. If the young person has previously participated in an MkV1 survey and has an MkV1 ID card then they should bring this ID card with them to the main survey.

Third activity (Main Survey)
On the scheduled day, the survey team will set up a survey point in a guesthouse, school or rented house in the village. All the young people who have been invited to attend the survey should come with their invitation. They will be asked to go through the following procedures:

1. **Registration:** All young persons will be first asked about their age and attendance at primary school (years and standards attended). All those eligible for the survey (participants) will proceed to the interview stage. If a young person is found not to be eligible, they will not be interviewed but will have the opportunity to be seen by the clinician and to be offered voluntary HIV counselling and testing, and will be given half a bar of soap and Tsh 2,000/=.
2. **Interview:** All participants will be asked some questions about their knowledge, attitudes and behaviour related to sexual and reproductive health.
3. **Biological specimens:** All participants will be asked to provide a small amount of urine, and a small sample of blood. The blood and urine samples will be tested for HIV and other sexually transmitted infections, and may be tested for other infections or illnesses at a future date. A thorough explanation will be given before any specimen is taken. All procedures will be done carefully and all regulations will be observed. No one will be forced to provide a specimen.
4. **Screening & Treatment:** A project clinician will see each participant and free treatment will be provided to those who need it. This will include treatment for any symptoms of sexually transmitted diseases, for schistosomiasis and other acute illnesses.
5. **VCT:** All participants will be offered voluntary counselling and testing for HIV.
6. **Incentives:** Each participant will be given a bar of soap and Tsh 4,000/=.

Confidentiality: None of the questionnaires, other forms, or specimens will be labelled with the participant's name. Instead, numbers will be used. This is to make sure that only the senior researchers can know who has answered what, and who has provided each specimen. All data and results will be kept secret, and any specimens will eventually be burnt, though they may be stored for many years at the National Institute for Medical Research in Mwanza before that is done.

Participation: We will encourage all potentially eligible young people to come for the survey. However, if anyone does not want to, we will not force them or inform anyone of this. A participant can also withdraw from the survey any time they want.

Further information: If you would like any further information on this project, please ask the representative who has brought you this information sheet. If you have additional questions or comments after (s) he has left, please address them to:

Ms. Aoife Doyle, Project Co-ordinator, MEMA kwa Vijana Trial Further Survey
Postal address: PO Box 11936, Mwanza
Location: National Institute for Medical Research, Isamilo, Mwanza
Telephone number: 2502203

- Please feel free to discuss this information sheet with anyone else you may wish to consult.
- Thank you for your time and assistance.

MEMA kwa Vijana Trial Further Survey (2007-2008)

Karatasi ya Maelezo kwa Wafanyakazi na Wanajamii

MEMA kwa Vijana ni mradi wa pamoja wa serikali ya Tanzania (Wizara ya Afya na Ustawi wa jamii Wizara ya Elimu na Mafunzo ya Ufundi), Taasisi ya Taifa ya Utafiti wa Magonjwa ya Binadamu (NIMR), AMREF, Chuo Kikuu cha Londoni cha Afya na Madawa katika Nchi za Joto (LSHTM- Uingereza), Chuo Kikuu cha Liverpool cha Madawa katika Nchi za Joto na Taasisi ya Afya ya Umma na Sayansi za Jamii (MRC, Glassgow, UK). Mradi huu unafanya kazi katika wiliaya nne za Mkoa wa Mwanza (Misungwi, Sengerema, Geita na Kwimba). Mradi unasaidia katika kuboresha matibabu ya magonjwa ya zinaa katika vituo vya afya, unatoa mafunzo ya ziada kwa wafanya kazi wa afya, na unatoa elimu ya afya ya uzazi kwa wanafunzi wa shule za msingi.

Katika mwaka 2007 na 2008 mradi utaendesha utafiti ili kutathmini matokeo ya utekelezaji wa mradi. Kutokana na tafiti za nyuma ambazo zilifanyika kati ya mwaka 2000 na 2002, tayari tunafahamu kwamba elimu ya MEMA kwa Vijana iliboresha ufahamu/elimu na baadhi ya mwienendo na tabia za kimapenzi kwa baadhi ya vijana katika jamii yako. Utafiti huu mwingine utatuambia kama maboresho haya yamekuwa endelevu na kama elimu hii imeweza kupunguza kasi ya maambukizi ya Virusi vya Ukimwi na magonjwa mengine ya zinaa kwa kipindi kirefu zaidi. Taarifa ambazo tutakusanya zitatusaidia kupata njia bora zaidi za kupunguza kuenea kwa virusi vya ukimwi na magonjwa mengine ya zinaa katika jamii yako.

Taratibu za Utafiti:
Utafiti utakuwa na sehemu mbili: (1) sensa (hesabu) ya kaya zote katika kata zilizochaguliwa na (2) utafiti mkuu ambao vijana waliolengwa/chaguliwa wakati wa sensa watahojiwa. Kwa msaada wa Afisa Habaari wa Mradi na viongozi wa eneo, taarifa kuhusu siku za sensa pamoja na taarifa juu ya madhumuni/nia na taratibu za sensa na utafiti mkuu, zitatumwa mapema kwa kila kiongozi wa kitongoji.

Kazi/Shughuli ya Kwanza (Utafiti wa awali na maandalizi ya sensa)
Afisa Habari wa MEMA kwa Vijana atatembelea kila eneo/jamii kabla ya timu ya sensa. Atakutana na viongozi wa kata, vijiji na vitongoji na kuwaeleza umuhimu, madhumuni na muda wa sensa na utafiti mkuu, kuwaomba ushirikiano wao na kuwaomba ruhusa ya kuendelea. Kila kiongozi wa kitongoji ataombwa kusaidia kwenye sensa kwa kutoa orodha ya kaya zote katika kitongoji chake. Orodha hii itaweza kutengenezwa/kutayarishwa kwa msaada wa viongizi wengine kwenye kitongoji. Pia wataombwa kuonyesha kama kuna kijana yeyote mwenye umri kati ya miaka 15 na 30 ambaye kwa wakati huu ni mkazi katika kaya hiyo. Afisa Habari atatoa fomu maalumu kwa ajili ya orodha hii na atataja siku ambayo atarudi kuchukua fomu za orodha za kaya ambazo tayari zitakuwa zimejazwa.

Kazi/Shughuli ya Pili (Sensa)
Timu ya sensa itafika kwa kila jamii. Watakuwa tayari wamepata orodha ya kaya kutoka kwa Afisa Habari wa Mradi. Kwa msaada wa viongozi wa vitongoji, wanatimu watatembelea kila kaya katika kitongoji. Watatoa kwa mdomo na kwa maandishi maelezo kwa mtu ambaye ni mkubwa zaidi katika kaya kuhusu utafiti na kupata ruhusa yao kwa kaya hiyo kushiriki katika sensa. “Mkuu wa Kaya” huyu ataulizwa maswali kuhusu jinsia na umri wa watu wote wanaoshi kwa wakati huo katika kaya hiyo. Taarifa hii itawekwa moja kwa moja katika kikompyuta kidogo. Kama kuna vijana wakubwa wenye umri kati ya miaka 15-30 wanaoishi katika kaya hiyo, maswali zaidi yataulizwa ili kuthibitisha umri wao na wataulizwa pia maswali kuhusu mahudhrurio yao ya shule za msingi ili kuona kama wanafaa kushiriki katika utafiti mkuu. Ni wale tu wenye umri kati ya mika 15-30 na wale waliosoma shule za msingi kwa miaka kadhaa ndio watakaoshiriki.

Mfanyakazi wa sensa atawapa vijana wote wakubwa ambao wanafaa kushiriki kwenye utafiti mkuu mwaliko wa kuhudhuria sehemu/kituo maalum (k.m. nyumba au nyumba ya kulala wageni) kijijini hapo siku 2-3 baadaye. Pia watapewa karatasi maalum ya maelezo ambayo itaeleza madhumuni na taratibu za utafiti kwa undani zaidi. Wataambiwa kwamba watapewa

zawadi kidogo ili kuwafidia kwa usafiri wao kwenda kituo cha utafiti, na kwa kutoa baadhi ya muda wao kwa ajili ya jambo hili. Kama kijana ana umri wa miaka 17, ataachiwa karatasi ya maelezo kwa ajili ya mzazi/wazazi au mlezi/walezi. Pamoja na kuwaeleza madhumuni makubwa ya utafiti, pia itawaomba kutohudhuria utafiti mkuu kama hawatapenda kufanya hivyo. Kama hapatakuwa na watu wazima wakati kaya itakapotembelewa, mfanyakazi wa sensa atatembelea tena kaya hiyo kwa safari mbili zaidi. Kama kijana anayehitajika hatakuwapo kwa muda, mkuu wa kaya ataombwa kumpa barua ya mwaliko na karatasi ya maelezo. Kama kijana amewahi kushiriki katika ufafiti wa MKV hapo nyuma na ana kadi ya Utambulisho ya MkV, atatakiwa aje na kitambulisho hicho kwenye utafiti mkuu.

Kazi ya tatu (Utafiti Mkuu)
Katika siku iliyopangwa, timu ya utafiti itafungua kituo cha utafiti kwenye nyumba ya kulala wageni, kwenye shule au nyumba ya kupanga kijijini. Vijana wote ambao watakuwa wameombwa kuhudhuria utafiti watatakiwa waje na barua zao za mwaliko. Wataombwa kupitia taratibu zifuatazo:

1. **Usajili:** Vijana wote wataulizwa kuhusu umri wao na mahudhurio yao ya shule za msingi
(miaka na madarasa waliyofikia). Wote wale watakaochaguliwa kwenye utafiti (washiriki) wataendelea kwenye hatua ya usaili. Kama kijana ataonekana kuwa hasitahili, hatahojiwa lakini atakuwa na nafasi ya kuonana na mganga na atapata ushauri nasaha na upimaji wa hiari wa Virusi vya Ukimwi, na atapewa nusu mche wa sabuni pamoja na shilingi 2,000/=.
2. **Usaili:** Washiriki wote wataulizwa kuhusu ujuzi, msimamo/mwelekeo na mienendo na tabia za mapenzi pamoja na elimu ya afya ya uzazi.
3. **Vipimo/Sampuli za kibiolojia:** Washiriki wote wataombwa kutoa kiasi kidogo cha mkojo na kiasi kidogo cha damu. Sampuli za damu na mkojo zitapimwa kwa ajili ya Virusi vya Ukimwi na magonjwa mengine ya zinaa, na wanaweza wakapimwa kwa ajili ya maambukizi mengine au magonjwa hapo baadaye. Maelezo kamili yatatolewa kabla ya kuchukuliwa vipimo. Taratibu zote zitafanywa kwa uangalifu na sheria/kanuni zote zitafuatwa. Hakuna mtu atakayelazimishwa kutoa vipimo endapo hatataka.
4. **Upimaji na Matibabu:** Mganga wa mradi ataonana na kila mshiriki na matibabu ya bure yatatolewa kwa wale watakaohitaji. Hii ni pamoja na matibabu ya dalili zozote za magonjwa ya zinaa na magonjwa mengine hatari.
5. **Motisha:** Kila mshiriki atapewa mche wa sabuni na shilingi 4,000/=.

Usiri: Hakuna hojaji/dodoso yeyote, fomu zozote au sampuli zitakazoandikwa jina la mshiriki. Badala yake zitatumika namba. Hii ni kuhakikisha kwamba ni watafiti waandamizi tu ndio watajua ni nani alijibu nini na aliyetoa kila vipimo. Data zote na matokeo yatakuwa ni siri, na baadaye kila sampuli itachomwa, ingawa zinaweza kutunzwa kwa miaka mingi kwenye Taasisi ya Taifa ya Utafiti wa Magonjwa ya Binadamu (NIMR) Mwanza kabla ya kufanya hivyo.

Ushiriki: Tutawapa moyo vijana wote wenye uwezo na wanaofaa kuja kushiriki kwenye utafiti. Hata hivyo, kama mtu yeyote hapendi, hatutamlazimisha au kumfahamisha mtu yeyote juu ya jambo hilo. Mshiriki anaweza pia kujitoa kwenye utafiti wakati wote akitaka.

Taarifa zaidi: Kama utapenda kupata taarifa zaidi kuhusu mradi huu, tafadhali mwambie mwakilishi aliyekuletea taarifa hii ya maelezo. Kama utakuwa na maswali ya ziada au maoni baada ya yeye kuondoka, tafadhali waandikie kwa anwani ifuatayo:
Ms.Aoife Doyle, Project Co-ordinator, MEMA kwa Vijana Trial Further Survey
Anuani ya Posta: PO Box 11936, Mwanza
Mahali: Taasisi ya Taifa ya Utafiti wa Magonjwa ya Binadamu, Isamilo Mwanza
Simu: 2502203

- Tafadhali jisikie huru kujadili karatasi hii ya maelezo na mtu yeyote ambaye utapenda akupe ushauri.
- Ahsante kwa muda na msaada wako.

MEMA kwa Vijana Trial Further Survey (2007-2008)

“Tathimini ya matokeo ya muda mrefu ya Mpango wa Elimu na Maadili ya Afya (MEMA) kwa Vijana”

MAELEZO KATIKA VIKAO VYA UHAMASISHAJI WA JAMII KWA NGAZI YA TARAFA, KATA NA VIJIJI

UTANGULIZI:

“MEMA” kwa Vijana ni kifupi cha maneno Mpango wa Elimu na Maadili ya Afya kwa Vijana. Wakati mwingine MEMA kwa Vijana hufupishwa na kusomeka “MkV1”. Mpango huu ulianza miaka ya 1998 kama mradi wa majaribio wa kuwaelimisha vijana kuhusu afya ya uzazi, elimu juu ya UKIMWI na maadili mema. Shughuli za mradi zilihusisha elimu ya afya mashuleni, uboreshaji huduma rafiki za afya kwa vijana katika vituo vya afya na uhamasishaji jamii.

Mradi ulifadhiliwa na shirika la misaada la Irish (Irish aid) na kutekelezwa kwa ushilikiano kati ya Serikali ya Tanzania (kupitia wizara za elimu na afya), Halmashauri za wilaya (Geita, Sengerema, Missungwi na Kwimba), Shirika linalojishughulisha na afya Africa (AMREF), Taasisi ya taifa ya utafiti wa tiba na madawa ya binadamu (NIMR), Chuo kikuu cha magojwa ya ukanda wa joto cha Londoni (LSHTM), Chuo kikuu cha magojwa ya ukanda wa joto cha Liverpool (LSTM) na Taasisi zingine za kimataifa. Mwanzo mradi ulitekelezwa katika kata 20 (10 zilipata mafunzo na 10 zilitumika kulinganisha matokeo ya mradi), kwa sasa mradi umepanuliwa na unazihusisha kata zote za wilaya nne za mkoa wa Mwanza kama zilivyo tajwa hapo awali na umepewa jina “MkV2” kutofautisha na ule wa awali ulio husisha baadhi tu ya kata.

Mradi huu ulikuwa ni moja ya jitihada za taifa kupunguza maambukizi ya Virusi vya UKIMWI, Magonjwa ya zinaa, mimba katika umri mdogo na kuchochea mabadiliko ya tabia kwa vijana. Ikumbukwe kuwa vijana ndiyo waliyo katika hatari zaidi ya kuambukizwa (mf: mwaka 2005 Bara la Africa lilikadiliwa kuwa na watu zaidi ya millioni 3 walioambukizwa virusi vya UKIMWI na zaidi ya nusu ni vijana kati ya umri wa miaka 15 na 25)**.**

I. LENGO KUU LA UTAFITI HUU (MkV1 FS)

Kutathimini na kuona kama mradi wa “MKV1” umesaidia kuleta matokeo/mabadiliko ya muda mrefu kwa vijana, hii ikiwa ni pamoja na :-

1. Ufahamu na Elimu juu ya maambukizi ya virusi vya UKIMWI, Magojwa ya zinaa
2. Kupungua kwa vitendo na tabia hatarishi na mitazamo potofu kuhusu janga la UKIMWI na matatizo mengine ya afya ya uzazi.
3. Kuongezeka kwa matumizi ya huduma za afya
4. Kupungua kwa maambukizi ya virusi vya UKIMWI na magonjwa ya zinaa

MEMA kwa Vijana Trial Further Survey (2007-2008)

II. MADHUMUNI

Kuwa na uhakika kama huduma ya MkV1 ni njia muafaka ya kuwasaidia vijana ili:-

- Waweze kutambua njia za kujikinga na maambukizi ya magonjwa ya zinaa na ukimwi.

UMUHIMU WA UTAFITI HUU

A) KWA VIJANA

- Kuwawezesha kujua uzito wa tatizo la maambukizi ya ukimwi kwa vijana ili waweze kuchukua tahadhari/kujikinga na maambukizi ya ukimwi
- Kupata tiba bure kwa wale watakaopatikana na magonjwa ya zinaa.
- Kupata ushauri nasaha na upimaji wa hiari wa virusi vya ukimwi kwa wale watakaohitaji

B) KWA JAMII

Kuwawezesha kujua ukubwa wa tatizo la ukimwi kwenye jamii hasa kwa vijana ili waweze kushiriki kikamilifu katika kukabiriana na tatizo hilo:-

- Waweze kuongea na watoto/vijana wao kwa uwazi juu ya tatizo la ukimwi
- Ushauri juu ya maadili mema kwa watoto/vijana wao.
- Waweze kujadili nini cha kufanya ili kupunguza tatizo

C) KWA TAIFA

- Kuliwezesha taifa kutambua uzito wa tatizo la maambukizi ya ukimwi kwa vijana ili taifa liweze kubuni mbinu/njia za kupambana na tatizo la ukimwi kwa vijana
- Kutambua uwezo/ufanisi wa mradi wa MKV katika kukabiliana na tatizo la HIV/AIDS kwa vijana
- Kubaini shughuri au miradi mingine inayoweza kusaidia kupunguza tatizo

D) KIMATAIFA

- Kuwawezesha wafadhiri waweze kutoa kipaumbele kwenye miradi yenye matokeo chanya ili kuwasaidia vijana katika mapambano dhidi ya ukimwi.

MEMA kwa Vijana Trial Further Survey (2007-2008)

UTARATIBU WA UTAFITI (HATUA MUHIMU ZA UTAFITI HUU)

1. Uhamasishaji
2. Sensa
3. Utafiti wenyewe

UHAMASISHAJI

Haya ni maandalizi ya awali ambapo maafisa uhamasishaji na habari wa mradi watafika kwenye kila kata ili kuonana na uongozi wa kata, vijiji na vitongoji kwa ajili ya kuelezea lengo na madhumuni pamoja na umuhimu wa utafiti unaotarajiwa kufanyika. Hii itafanyika kwa kufanya mikutano na mabaraza ya uongozi wa kata, ujio wa timu ya sensa na timu ya utafiti utaelezwa katika vikao hivyo.Viongozi wa vitongoji watapatiwa fomu maalum kwa ajili ya kuandikisha majina ya wakuu wa kaya zenye vijana wa umri katai ya miaka15-30

SENSA

- Timu ya sensa itafika kwenye jamii/ kwenye kata husika baada ya maafisa uhamasishaji na habari wa mradi kurudi toka katani na kupeleka majina ya wakuu wa kaya. Zoezi la sensa litafanyika ili kuwatambua vijana wenye sifa za kushiriki kwenye zoezi la utafiti.Wafanyakazi wa sensa watafanya zoezi hili kwa kushirikiana na viongozi wa vitongoji kwa kupitia kaya hadi kaya kuorodhesha vijana wote wenye sifa zote za kushiriki utafiti.
- Wafanyakazi wa sensa watatumia kompyuta ndogo(PDA) kuingiza takwimu za utafiti
- Kibali cha kuwaruhusu vijana kushiriki(kwa wale walioko chini ya miaka 18) kitatolewa na mkuu wa kaya au mtu mzima mwingine kwenye kaya husika

Sifa za kushiriki kwenye utafiti

Ni wale tu waliokuwa kwenye shule za msingi kwa kipindi cha mwaka mmoja katika madarasa ya 5, 6 na 7kati ya miaka ya 1999-2004 huo ndiyo muda MKV ilitoa mafunzo kwa shule za msingi husika.

UTAFITI WENYEWE

Katika siku maalum iliyopangwa timu ya utafiti itafika kwenye kata husika na itakuwa imeandaliwa sehemu maalum ya kufanyia kazi hii yawezekana ikawa kwenye nyumba ya kulala wageni, shuleni, nyumba ya kukodisha hapo kijijini.Nyumba itakayohitajika ni lazima iwe na vyumba visivyopungua 8.Washiriki wote watapitia hatua zifuatavyo:-

1. Uandikishaji(kuhakiki washiriki kama wana sifa za kushiriki kwenye utafiti)
2. Usaili (maswali ya ufahamu juu ya elimu ya afya jinsia na uzazi)
3. Uchukuaji wa sampuli za kibiolojia(sampuli kidogo ya damu pamoja na mkojo vitachukuliwa kwa ajili ya utafiti Sampuli za damu na mkojo zitapimwa kwa ajili ya maambukizi ya ukimwi na magonjwa mengine ya zinaa)

MEMA kwa Vijana Trial Further Survey (2007-2008)

4. Upimaji na matibabu: Mganga wa mradi ataonana na kila mshiriki na matibabu ya bure yatatolewa kwa wale watakaohitaji.
5. Ushauri nasaha na upimaji virusi vya ukimwi kwa hiari, huduma hii itatolewa bure na washauri nasaha wa mradi kwa washiriki watakao hitaji.

MOTISHA KWA WASHIRIKI

Washiriki wote (wenyesifa) watapewa mche wa sabuni kwa kila mshiriki pamoja na 4000/= hii ni kwa ajili ya kufidia muda wao alioutoa kwa ajili ya kushiriki kwenye utafiti na gharama za usafiri.

USIRI

Mambo yote yatakayofanyika wakati wa utafiti yatatunzwa kwa siri na jambo lolote mtafiti atakaloongea na washiriki halitatangazwa au kuzungumzwa popote.Usaili utafanywa na mtu wa jinsia moja na msailiwa.Pia vipimo pamoja na dodoso hazitaandikwa jina la msailiwa badala yake zitatumika namba ili kutunza usiri.Vipimo pamoja na takwimu vitatunzwa NIMR Mwanza kwa siri kwa kipindi cha hadi miaka 10.

Maelezo haya ni sehemu ya taarifa ya mradi kwa jamii, waweza pia kumshirikisha mtu yoyote kusoma. Kumbuka kama utakuwa na maoni au maswali waweza kumuuliza muwakilishi yeyote wa mradi au wasiliana nasi kwa anuani hii : -

Aoife D,

Mratibu
MkV1FS
National Institute of Medical Research (NIMR)
Box 11936, Mwanza- Isamiro area.

Ofisi ya Mtendaji wa Kata

Kata ya…………………….

S.L.P……………………….

Wilaya________________

Tarehe ________________

Kumb:

Mtendaji/Mwenyekiti wa kijiji

Kijiji cha………………………………..

S.L.P…………………………………

Wilaya______________________

YAH: Tathmini ya MEMA kwa Vijana.

Rejea kichwa cha habari hapo juu, unaombwa kuwapokea na kuwapa ushirikiano wa hali ya juu timu ya tathmini toka NIMR Mwanza ambayo inahitaji kufanya tathmini ya utekelezaji wa shughuli za mafunzo ya MEMA kwa Vijana yaliyofanyika katika kijiji chako cha ……………………………………….. tangu mpango huu uanze.

Nimatumaini yangu kuwa utawasaidia kwa kadri inavyowezekana katika kukamilisha shughuli walizopanga zitekelezwe kwa wakati huo.

Ahsante.

NATIONAL INSTITUTE FOR MEDICAL RESEARCH
MWANZA MEDICAL RESEARCH CENTRE

Telephone:028-2500399/2503012/40723
Direct:028-2500189 Fax: 028-2500654
Telegrams: MEDSEARCH
E-mail::mwanza@nimr.co.tz

P.O. Box 1462
Mwanza
Tanzania.

Tarehe:

Mr/Mrs:

YAH: HUDUMA YA UKUMBI/VYUMBA/NYUMBA WAKATI WA UTAFITI

Somo hapo juu la husika.
Haya ni makubaliano ya kutumia sehemu iliyotajwa hapo juu (____) kwa siku ______ kuanzia tarehe ______________ hadi ___________ kwa gharama ya tsh.__________ kwa siku.

Makubaliano haya ni kati ya MkV1FS (mpangaji) na Mr/Mrs___________________ (mwenye nyumba)

Makubaliano haya ni kwa mujibu wa **masharti** yaliyo kubaliwa kati ya muwakirishi wa shirika na mwenye nyumba kwa maelewano yasiyo ya kimaandishi.

Malipo ya awali (kama yapo)

Tsh. ________________

Walio saini makubaliano:

1. ____ (MkV1FS), Tarehe__________

2. ____________________________________ (Mwenye nyumba), Tarehe ________

Aoife D.

Mratibu- MkV1 FS

MEMA kwa Vijana Trial Further Survey (2007-2008)

MKV1 FS: List of Household Heads

Sehemu "A" Identification
(To be completed by Mobilisation Officer)

Staff code: ___ ___ ___

Tarehe: ___ ___ / ___ ___ / ___ ___ ___ ___

Jina la Wilaya: ________________________________, (code): ___ ___

Jina la Kata: ________________________, (code): ___ ___ ___

Jina la Kijiji: ________________________, (code): ___ ___ ___

Jina la Kitongoji: ________________________________

Sehemu "B": Majina ya wakuu wa Kaya(Kaya Zote)
(Ijazwe na kiongozi wa kitongoji/kijiji)

S/N ya Kaya	Jina kamili la mkuu wa KAYA	**Jinsia ya Mkuu wa kaya**	Watu wenye umri wa miaka 15-30<br><br>Weka √ kama yupo<br>Weka **X** kama hayupo	Uhamasishaji na ugawaji karatasi za maelozo kwa wakuu wa kaya<br><br>Weka √ kama umefanyika<br>Weka **X** kama haujafanyika
1				
2				
3				
4				
5				
6				
7				
8				
9				
10				
11				
12				
13				
14				
15				

MEMA kwa Vijana Trial Further Survey (2007-2008)

16				
17				
18				
19				
20				
21				
22				
23				
24				
25				
26				
27				
28				
29				
30				
31				
32				
33				
34				
35				
36				
37				
38				
39				
40				
41				
42				
43				
44				
45				
46				
47				
48				
49				
50				
51				

MEMA kwa Vijana Trial Further Survey (2007-2008)

52				
53				
54				
55				
56				
57				
58				
59				
60				
61				
62				
63				
64				
65				
66				
67				
68				
69				
70				
71				
72				
73				
74				
75				
76				
77				
78				
79				
80				
81				
82				
83				
84				

Jina la aliyejaza ______________________________:

Cheo____________________Tarehe______________________________

Sahihi_____________________, Anuani ______________________________

MEMA kwa Vijana Trial Further Survey (2007-2008)

MkV1 FS: List of Household heads (Additional Sheet)

Jina la Kijiji: ___________________________

Jina la Kitongoji: ______________________________________

S/N ya Kaya	Jina kamili la mkuu wa KAYA	**Jinsia ya Mkuu wa kaya**	Watu wenye umri wa miaka 15-30<br><br>Weka √ kama yupo<br>Weka **X** kama hayupo	Uhamasishaji na ugawaji karatasi za maelozo kwa wakuu wa kaya<br><br>Weka √ kama umefanyika<br>Weka **X** kama haujafanyika

MkV1FS Prot A18d_HH list Add (Swah 23May07).doc

MEMA kwa Vijana Trial Further Survey (2007-2008)

Information Sheet for Household Head

The MEMA kwa Vijana Programme is a collaborative project of the government of Tanzania (the Ministry of Health & Social Welfare and the Ministry of Education & Vocational Training), the National Institute for Medical Research (NIMR), AMREF, the London School of Hygiene and Tropical Medicine, UK (LSHTM), the Liverpool School of Tropical Medicine (LSTM) and MRC Social and Public Health Sciences Institute, Glasgow, UK. The programme is working in four districts of Mwanza Region (Misungwi, Sengerema, Geita, and Kwimba). The programme supports improved treatment of sexually transmitted diseases in health facilities, provides additional training for health workers, and provides sexual and reproductive health education to primary school pupils.

During 2007 and 2008, the programme will conduct a survey to assess the impact of the intervention ***'MkV1 Trial Further Survey'***. From previous surveys that were conducted between 2000 and 2002, we already know that the MEMA kwa Vijana intervention improved the knowledge and some reported sexual behaviours in young people in your community. This further survey will tell us if these improvements have been sustained, and whether the intervention has been effective in reducing the spread of HIV and other sexually transmitted infections in the longer-term. The information that we collect will help us to find the best ways to reduce the spread of HIV and other sexually transmitted diseases in your community.

The survey has two parts:

(1) A census of all households in the selected wards
(2) A main survey in which young people identified during the census will be interviewed.

We are now conducting a census of all households in this ward. Your sub-village leader is helping us to visit all the households in this sub-village. We would like to ask you some questions about the people who live here in this household. We are particularly interested in young people who have been to primary school in this ward and will invite some of these young people to be interviewed in a few days time. The information that you provide for us will help us chose the right young people to invite for the second part of our survey.

If you agree to take part in this census, we will ask you some simple questions about the age and sex of those living here. We will enter this information onto small computers. This information will be kept private. The questions should take approximately **10 minutes.**

We would like you to participate in this census. But, if you do not want to, please understand that we will not force you to, and that you will still be allowed to participate in other MEMA kwa Vijana Programme activities.

Do you have any questions or want me to repeat anything I have just told you?
Pause to solicit questions.

Further information: If you would like any further information on this project, please ask the representative who has brought you this information sheet. If you have additional questions or comments after (s)he has left, please address them to:
Ms. Aoife Doyle, Project Co-ordinator, MEMA kwa Vijana Trial Further Survey
Postal address: PO Box 11936, Mwanza
Location: National Institute for Medical Research, Isamilo, Mwanza
Telephone number: 2502203

- Please feel free to discuss this information sheet with anyone else you may wish to consult.
- Thank you for your time and assistance.

MEMA kwa Vijana Trial Further Survey (2007-2008)

Maelezo kwa mkuu wa kaya

MEMA kwa Vijana ni mradi wa pamoja wa serikali ya Tanzania (Wizara ya Afya na Ustawi wa jamii Wizara ya Elimu na Mafunzo ya Ufundi), Taasisi ya Taifa ya Utafiti wa Magonjwa ya Binadamu (NIMR), AMREF, Chuo Kikuu cha Londoni cha Afya na Madawa katika Nchi za Joto (LSHTM- Uingereza), Chuo Kikuu cha Liverpool cha Madawa katika Nchi za Joto na Taasisi ya Afya ya Umma na Sayansi za Jamii (MRC, Glassgow, UK). Mradi huu unafanya kazi katika wiliaya nne za Mkoa wa Mwanza (Misungwi, Sengerema, Geita na Kwimba). Mradi unasaidia katika kuboresha matibabu ya magonjwa ya zinaa katika vituo vya afya, unatoa mafunzo ya ziada kwa wafanya kazi wa afya, na unatoa elimu ya afya ya uzazi kwa wanafunzi wa shule za msingi.

Katika mwaka 2007 na 2008 mradi utaendesha utafiti ili kutathmini matokeo ya utekelezaji wa mradi. Kutokana na tafiti za nyuma ambazo zilifanyika kati ya mwaka 2000 na 2002, tayari tunafahamu kwamba elimu ya MEMA kwa Vijana iliboresha ufahamu/elimu na baadhi ya mwienendo na tabia za kimapenzi kwa baadhi ya vijana katika jamii yako. Utafiti huu mwingine utatuambia kama maboresho haya yamekuwa endelevu na kama elimu hii imeweza kupunguza kasi ya maambukizi ya Virusi vya Ukimwi na magonjwa mengine ya zinaa kwa kipindi kirefu zaidi. Taarifa ambazo tutakusanya zitatusaidia kupata njia bora zaidi za kupunguza kuenea kwa virusi vya ukimwi na magonjwa mengine ya zinaa katika jamii yako.

Utafiti umegawanyika sehemu 2

1) Sensa ya kaya zote katika kata zilizochaguliwa
2) Utafiti mkuu ambapo vijana watakaotambuliwa wakati wa sensa watahojiwa..

Sasa tunafanya Sensa ya kaya zote katika kata hii. Kiongozi wako wa kitongaji anatusaidia kutembelea kaya zote katika kitongoji hiki. Tutapenda kukuuliza maswali kuhusu watu wanaoishi katika Kaya hii. Tunapendelea zaidi vijana waliosoma shule ya msingi katika kata hii na tutawaalika baadhi ya vijana kushiriki usaili katika kipindi cha muda wa siku chache zijazo. Taarifa utakazotoa kwetu zitatusaidia kucnagua vijana wanaostahili kualikwa kwenye usahili wa mara ya pili.

Kama utakubali kushiriki kwenye Sensa, tutakuuliza maswali rahisi kuhusiana na umri, na jinsia za watu wanaoishi hapa. Tutaweka taarifa hizi katika chombo kidogo cha kitaalamu. Taarifa hizi zitahifadhiwa kwa usiri. Maswali yatachukua takribani dakika 10.

Tunahitaji wewe ushiriki katika sensa hii, ila kama hauhitaji, tafadhali elewa hatutakulazimisha na pia utaendelea kushiriki katika shughuli nyingine za programu ya MEMA kwa Vijana. Je, una swali lolote au unataka nirudie chochote nilichokwambia?
(subiri afikiri swali)

Taarifa zaidi: Kama utapenda kupata taarifa zaidi kuhusu mradi huu, tafadhali mwambie mwakilishi aliyekuletea taarifa hii ya maelezo. Kama utakuwa na maswali ya ziada au maoni baada ya yeye kuondoka, tafadhali waandikie kwa anwani ifuatayo:

Ms.Aoife Doyle, Project Co-ordinator, MEMA kwa Vijana Trial Further Survey
Anuani ya Posta: PO Box 11936, Mwanza; Mahali: Taasisi ya Taifa ya Utafiti wa Magonjwa ya Binadamu, Isamilo Mwanza; Simu: 2502203

- Tafadhali jisikie huru kujadili karatasi hii ya maelezo na mtu yeyote ambaye utapenda akupe ushauri.
- Ahsante kwa muda na msaada wako

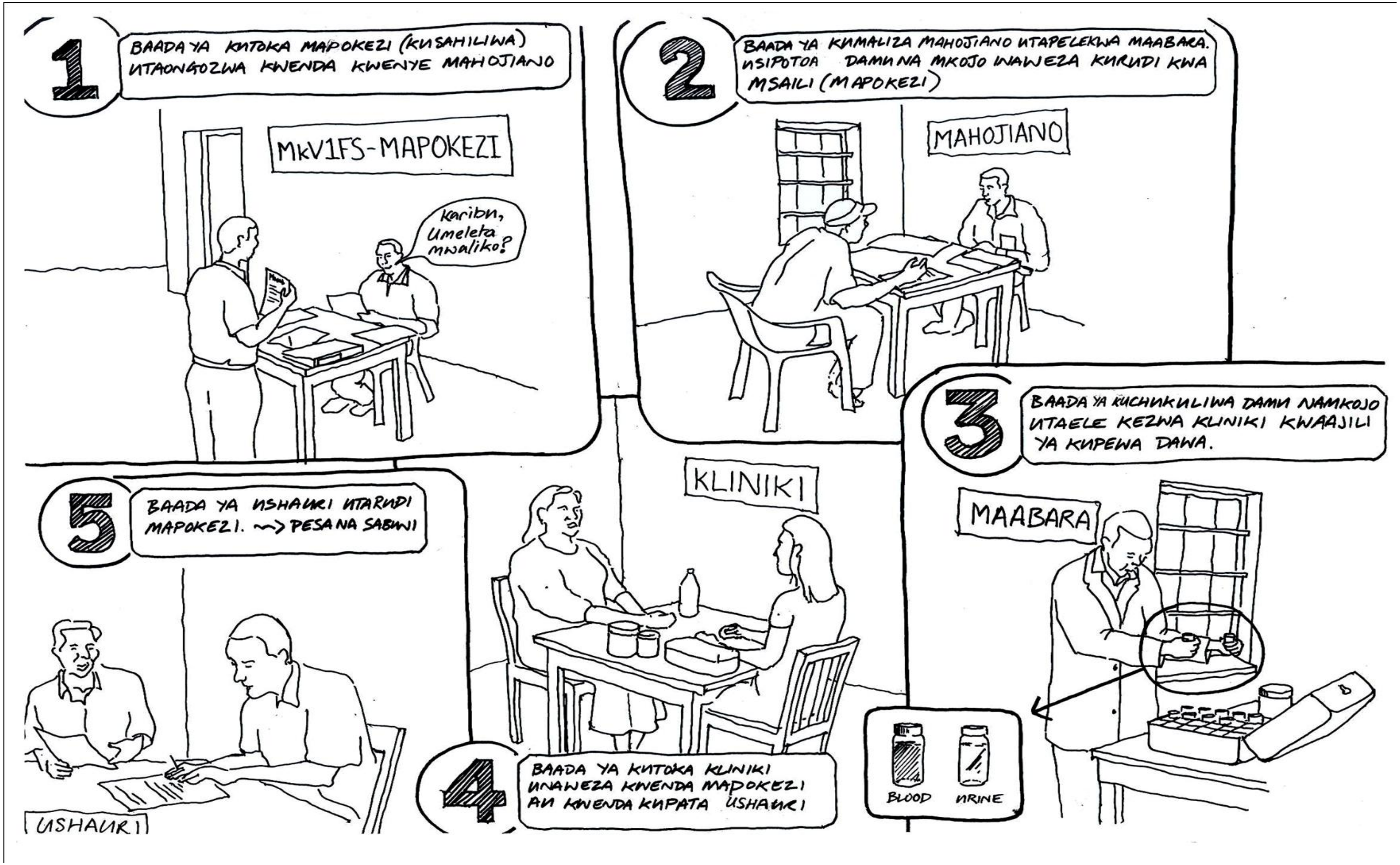
1
BAADA YA KUTOKA MAPOKEZI (KUSAHILIWA) UTAONGOZWA KWENDA KWENYE MAHOJIANO
MkV1FS-MAPOKEZI
Karibu, Umeleta mwaliko?
2
BAADA YA KUMALIZA MAHOJIANO UTAPELEKWA MAABARA. USIPOTOA DAMU NA MKOJO UNAWEZA KURUDI KWA MSAILI (MAPOKEZI)
MAHOJIANO
3
BAADA YA KUCHUKULIWA DAMU NAMKOJO UTAELE KEZWA KLINIKI KWAAJILI YA KUPEWA DAWA.
MAABARA
BLOOD
URINE
KLINIKI
4
BAADA YA KUTOKA KLINIKI UNAWEZA KWENDA MAPOKEZI AU KWENDA KUPATA USHAURI
5
BAADA YA USHAURI UTARUDI MAPOKEZI. ~> PESA NA SABUNI
USHAURI

MEMA kwa Vijana Trial Further Survey (2007-2008)

MKV1 FS
Mobilisation Report Form: Part "A"

1.1	Date of meeting (forum)		|__||__|/|__||__|/ 20|__||__|	IntDate
1.2	Mobilisation Officer's staff code		|__||__||__|	MOCode
1.3	District name & code		|__||__|	District
1.4	Community name & code		|__||__|	Cmty

Participants in Mobilisation Forum

S/N	Full name	Position/Title	Contact address		Signature
			Phone No.	Physical (name of village & Kitongoji)	
1					
2					
3					
4					
5					
6					
7					
8					
9					
10					
11					
12					

MEMA kwa Vijana Trial Further Survey (2007-2008)

13					
14					
15					
16					
17					
18					
19					
20					
21					
22					
23					
**OTHER PEOPLE VISITED AT WARD LEVEL (NOT IN FORUM)**					
1					
2					
3					
4					

MEMA kwa Vijana Trial Further Survey (2007-2008)

Mobilisation Report Form: Part "B1"

1.1	Date of visit village (first day)		|__||__|/|__||__|/ 20|__||__|	IntDate
1.2	Mobilisation Officer's staff code		|__||__||__|	MOCode
1.3	Ward name & code		|__||__|	wardCode
1.4	Village name & code		|__||__|	VilCode

People Informed about MkV1 FS (village level & Vitongoji)

S/N	Full name	Position/Title (if any)	Contact address		Signature	Date
			Phone No.	Physical (name of village & kitongoji)		
1						
2						
3						
4						
5						
6						
7						
8						
9						
10						
11						
12						
13						
14						

MEMA kwa Vijana Trial Further Survey (2007-2008)

15						
16						
17						
18						
19						
20						
21						
22						
23						
24						
25						
26						
27						
28						

Other Information

S/N	Name of Kitongoji	Total number of Household	No. of household with young people aged between 15-30
01			
02			
03			
04			
05			
06			
07			
08			
09			
10			

MEMA kwa Vijana Trial Further Survey (2007-2008)

Mobilisation Report Form: Part "B2"

1.1	Date of visit village (first day)		|__||__|/|__||__|/ 20|__||__|	IntDate
1.2	Mobilisation Officer's staff code		|__||__||__|	MOCode
1.3	Ward name & code		|__||__|	wardCode
1.4	Village name & code		|__||__|	VilCode

People Informed about MkV1 FS (village level & Vitongoji)

S/N	Full name	Position/Title (if any)	Contact address		Signature	Date
			Phone No.	Physical (name of village & kitongoji)		
1						
2						
3						
4						
5						
6						
7						
8						
9						
10						
11						
12						
13						
14						
15						

MEMA kwa Vijana Trial Further Survey (2007-2008)

16						
17						
18						
19						
20						
21						
22						
23						
24						
25						
26						
27						
28						

Other Information

S/N	Name of Kitongoji	Total number of Household	No. of household with young people aged between 15-30
01			
02			
03			
04			
05			
06			
07			
08			
09			
10			

MEMA kwa Vijana Trial Further Survey (2007-2008)

Mobilisation Report Form: Part "B3"

1.1	Date of visit village (first day)		|__||__|/|__||__|/ 20|__||__|	IntDate
1.2	Mobilisation Officer's staff code		|__||__||__|	MOCode
1.3	Ward name & code		|__||__|	wardCode
1.4	Village name & code		|__||__|	VilCode

People Informed about MkV1 FS (village level & Vitongoji)

S/N	Full name	Position/Title (if any)	Contact address		Signature	Date
			Phone No.	Physical (name of village & kitongoji)		
1						
2						
3						
4						
5						
6						
7						
8						
9						
10						
11						
12						
13						
14						
15						
16						

MEMA kwa Vijana Trial Further Survey (2007-2008)

17						
18						
19						
20						
21						
22						
23						
24						
25						
26						
27						
28						

Other Information

S/N	Name of Kitongoji	Total number of Household	No. of household with young people aged between 15-30
01			
02			
03			
04			
05			
06			
07			
08			
09			
10			

MEMA kwa Vijana Trial Further Survey (2007-2008)

Mobilisation Report Form: Part "B4"

1.1	Date of visit village (first day)		|__||__|/|__||__|/ 20|__||__|	IntDate
1.2	Mobilisation Officer's staff code		|__||__||__|	MOCode
1.3	Ward name & code		|__||__|	wardCode
1.4	Village name & code		|__||__|	VilCode

People Informed about MkV1 FS (village level & Vitongoji)

S/N	Full name	Position/Title (if any)	Contact address		Signature	Date
			Phone No.	Physical (name of village & kitongoji)		
1						
2						
3						
4						
5						
6						
7						
8						
9						
10						
11						
12						
13						

MEMA kwa Vijana Trial Further Survey (2007-2008)

14						
15						
16						
17						
18						
19						
20						
21						
22						
23						
24						
25						
26						
27						
28						

Other Information

S/N	Name of Kitongoji	Total number of Household	No. of household with young people aged between 15-30
01			
02			
03			
04			
05			
06			
07			
08			
09			
10			

MEMA kwa Vijana Trial Further Survey (2007-2008)

Mobilisation Report Form: Part "B5"

1.1	Date of visit village (first day)		|__||__|/|__||__|/ 20|__||__|	IntDate
1.2	Mobilisation Officer's staff code		|__||__||__|	MOCode
1.3	Ward name & code		|__||__|	wardCode
1.4	Village name & code		|__||__|	VilCode

People Informed about MkV1 FS (village level & Vitongoji)

S/N	Full name	Position/Title (if any)	Contact address		Signature	Date
			Phone No.	Physical (name of village & kitongoji)		
1						
2						
3						
4						
5						
6						
7						
8						
9						
10						
11						
12						
13						
14						
15						
16						

MEMA kwa Vijana Trial Further Survey (2007-2008)

17						
18						
19						
20						
21						
22						
23						
24						
25						
26						
27						
28						

Other Information

S/N	Name of Kitongoji	Total number of Household	No. of household with young people aged between 15-30
01			
02			
03			
04			
05			
06			
07			
08			
09			
10			

MEMA kwa Vijana Trial Further Survey (2007-2008)

Mobilisation Report Form: Part "B6"

1.1	Date of visit village (first day)		|__||__|/|__||__|/ 20|__||__|	IntDate
1.2	Mobilisation Officer's staff code		|__||__||__|	MOCode
1.3	Ward name & code		|__||__|	wardCode
1.4	Village name & code		|__||__|	VilCode

People Informed about MkV1 FS (village level & Vitongoji)

S/N	Full name	Position/Title (if any)	Contact address		Signature	Date
			Phone No.	Physical (name of village & kitongoji)		
1						
2						
3						
4						
5						
6						
7						
8						
9						
10						
11						
12						
13						
14						

MEMA kwa Vijana Trial Further Survey (2007-2008)

15						
16						
17						
18						
19						
20						
21						
22						
23						
24						
25						
26						
27						
28						

Other Information

S/N	Name of Kitongoji	Total number of Household	No. of household with young people aged between 15-30
01			
02			
03			
04			
05			
06			
07			
08			
09			
10			

MEMA kwa Vijana Trial Further Survey (2007-2008)

Mobilisation Report Form: Part "B7"

1.1	Date of visit village (first day)		|__||__|/|__||__|/ 20|__||__|	IntDate
1.2	Mobilisation Officer's staff code		|__||__||__|	MOCode
1.3	Ward name & code		|__||__|	wardCode
1.4	Village name & code		|__||__|	VilCode

People Informed about MkV1 FS (village level & Vitongoji)

S/N	Full name	Position/Title (if any)	Contact address		Signature	Date
			Phone No.	Physical (name of village & kitongoji)		
1						
2						
3						
4						
5						
6						
7						
8						
9						
10						
11						
12						
13						
14						
15						

MEMA kwa Vijana Trial Further Survey (2007-2008)

16						
17						
18						
19						
20						
21						
22						
23						
24						
25						
26						
27						
28						

Other Information

S/N	Name of Kitongoji	Total number of Household	No. of household with young people aged between 15-30
01			
02			
03			
04			
05			
06			
07			
08			
09			
10			

MEMA kwa Vijana Trial Further Survey (2007-2008)

Mobilisation Report Form: Part "C"

Ward (name):________________________ (code): |__||__|

School & Health centre

S/N	Name of Health centre	Village Code	Names of Contact person(s) (E.g. HT & HC/in-charge)	Address of contact person		Remark/Observation
				Phone No.	Physical (name of village & kitongoji)	
1						
2						
3						
4						
5						
6						
7						
8						
9						
10						
11						
12						

MEMA kwa Vijana Trial Further Survey (2007-2008)

Mobilisation Report Form: Part "D"

Narrative Report

Part I: **Survey site** (Mention all potential survey sites)

S/N	Village name	Proposed survey sites	Rooms available, status of chairs/tables /electricity/water/toilet etc	Cost /room/day	Contact person
1					
2					
3					
4					
5					
6					
7					
8					

MEMA kwa Vijana Trial Further Survey (2007-2008)

Part II: Accommodation (Mention the proposed guest house/ houses)

1.____ (Cost per room Tsh.____________)

2.____ (Cost per room Tsh.____________)

Part III: Important issues

____

____

____

____

Part IV: Questions and ideas of community leaders (during mobilisation meeting)

____

____

____

____

____

____

____

____

____

____

____

____

MEMA kwa Vijana Trial Further Survey (2007-2008)

MkV1 Further Survey: Village Information Form

COMPLETE THIS FORM BY INTERVIEWING AT LEAST 2 RELIABLE INFORMANTS			
1.01	Date of completion		/ /200
1.02	Name and position of informants who helped with completion of this form	1. ------------------------------<br>2. ------------------------------<br>3. ------------------------------	
1.03	Ward *(record ward code number)*	ward name: ------------------------------	
1.04	Village *(record village code number)*	village name: ------------------------------	
1.05	Census Interviewer's staff code		
1.06	Name the 3 most common places for people in this village seek care when they have sexually transmitted infections<br><br>*e.g. Hospital, Health centre, Mobile clinic, Dispensary, Private 'freelance' providers, Traditional, Self-treatment, Other*	1. ------------------------------<br>2. ------------------------------<br>3. ------------------------------	
1.07	How long does it take to travel to the nearest <u>permanent</u> government or NGO facility that provides treatment for sexually transmitted infections? *Enter 999 if NK*	*circle mode of transport*	mins<br>Vehicle 1<br>Bicycle 2<br>Foot 3
1.08	How long does it take to travel on foot to the nearest place that people living in this village can access family planning information/care? *Enter 999 if NK*	*circle mode of transport*	mins<br>Vehicle 1<br>Bicycle 2<br>Foot 3
1.09	How long does it take to travel to the nearest place that people living in this village can access voluntary counselling and testing for HIV? *Enter 999 if NK*	*circle mode of transport*	mins<br>Vehicle 1<br>Bicycle 2<br>Foot 3
1.10	Are there other Health NGOs working in this village or surrounding area? (if yes, please provide details)	*(Circle one)*	Yes 1<br>No 2<br>NK 9

MEMA kwa Vijana Trial Further Survey (2007-2008)

	DETAILS OF HEALTH NGOS:			
1.11	Are there any groups providing HIV/AIDS or RH education or services in this village or surrounding area? (if yes, please provide details)	(Circle one)	Yes<br>No<br>NK	1<br>2<br>9
	DETAILS OF GROUPS PROVIDING HIV/AIDS OR RH EDUCATION OR SERVICES IN THIS VILLAGE OR SURROUNDING AREA:			
1.12	How often are commercial video-shows held in this village or surrounding area? *(circle only one)*		Permanent<br>Weekly<br>Monthly<br>Seasonally<br>Less frequently<br>Never<br>Don't Know	1<br>2<br>3<br>4<br>5<br>6<br>9
1.13	How often are health promotion video-shows held in this village or surrounding area? *(circle only one)*		Permanent<br>Weekly<br>Monthly<br>Seasonally<br>Less frequently<br>Never<br>Don't Know	1<br>2<br>3<br>4<br>5<br>6<br>9
	DETAILS OF VIDEO SHOWS (COMMERCIAL AND/OR HEALTH PROMOTION):			
	OTHER OBSERVATIONS:			

MEMA kwa Vijana Trial Further Survey (2007-2008)

MKV1 FS: CI FORM FOR HOUSEHOLD HEAD

VILLAGE NAME: ___________________ *Code* :_____ Kitongoji: (NAME): _________________________________

S/NO	NAME OF HH HEAD	SEX	CENSUS STATUS			
		M	**Date & Time**			
		F	**Outcome** *1=Inter, 2= Absent, 3= Refused 4= Repeat HH, 5= HH unknown, 6= HH shifted*			
		M	**Date & Time**			
		F	**Outcome** *1=Inter, 2= Absent, 3= Refused 4= Repeat HH, 5= HH unknown, 6= HH shifted*			
		M	**Date & Time**			
		F	**Outcome** *1=Inter, 2= Absent, 3= Refused 4= Repeat HH, 5= HH unknown, 6= HH shifted*			
		M	**Date & Time**			
		F	**Outcome** *1=Inter, 2= Absent, 3= Refused 4= Repeat HH, 5= HH unknown, 6= HH shifted*			
		M	**Date & Time**			
		F	**Outcome** *1=Inter, 2= Absent, 3= Refused 4= Repeat HH, 5= HH unknown, 6= HH shifted*			
		M	**Date & Time**			
		F	**Outcome** *1=Inter, 2= Absent, 3= Refused 4= Repeat HH, 5= HH unknown, 6= HH shifted*			
		M	**Date & Time**			
		F	**Outcome** *1=Inter, 2= Absent, 3= Refused 4= Repeat HH, 5= HH unknown, 6= HH shifted*			
		M	**Date & Time**			
		F	**Outcome** *1=Inter, 2= Absent, 3= Refused 4= Repeat HH, 5= HH unknown, 6= HH shifted*			
		M	**Date & Time**			
		F	**Outcome** *1=Inter, 2= Absent, 3= Refused 4= Repeat HH, 5= HH unknown, 6= HH shifted*			
		M	**Date & Time**			
		F	**Outcome** *1=Inter, 2= Absent, 3= Refused 4= Repeat HH, 5= HH unknown, 6= HH shifted*			
		M	**Date & Time**			
		F	**Outcome** *1=Inter, 2= Absent, 3= Refused 4= Repeat HH, 5= HH unknown, 6= HH shifted*			
		M	**Date & Time**			
		F	**Outcome** *1=Inter, 2= Absent, 3= Refused 4= Repeat HH, 5= HH unknown, 6= HH shifted*			

MEMA kwa Vijana Trial Further Survey (2007-2008)

S/NO	NAME OF HH HEAD	SEX	CENSUS STATUS			
		M<br>F	**Date & Time**			
			**Outcome** *1=Inter, 2= Absent, 3= Refused 4= Repeat HH, 5= HH unknown, 6= HH shifted*			
		M<br>F	**Date & Time**			
			**Outcome** *1=Inter, 2= Absent, 3= Refused 4= Repeat HH, 5= HH unknown, 6= HH shifted*			
		M<br>F	**Date & Time**			
			**Outcome** *1=Inter, 2= Absent, 3= Refused 4= Repeat HH, 5= HH unknown, 6= HH shifted*			
		M<br>F	**Date & Time**			
			**Outcome** *1=Inter, 2= Absent, 3= Refused 4= Repeat HH, 5= HH unknown, 6= HH shifted*			
		M<br>F	**Date & Time**			
			**Outcome** *1=Inter, 2= Absent, 3= Refused 4= Repeat HH, 5= HH unknown, 6= HH shifted*			
		M<br>F	**Date & Time**			
			**Outcome** *1=Inter, 2= Absent, 3= Refused 4= Repeat HH, 5= HH unknown, 6= HH shifted*			
		M<br>F	**Date & Time**			
			**Outcome** *1=Inter, 2= Absent, 3= Refused 4= Repeat HH, 5= HH unknown, 6= HH shifted*			
		M<br>F	**Date & Time**			
			**Outcome** *1=Inter, 2= Absent, 3= Refused 4= Repeat HH, 5= HH unknown, 6= HH shifted*			
		M<br>F	**Date & Time**			
			**Outcome** *1=Inter, 2= Absent, 3= Refused 4= Repeat HH, 5= HH unknown, 6= HH shifted*			
		M<br>F	**Date & Time**			
			**Outcome** *1=Inter, 2= Absent, 3= Refused 4= Repeat HH, 5= HH unknown, 6= HH shifted*			
		M<br>F	**Date & Time**			
			**Outcome** *1=Inter, 2= Absent, 3= Refused 4= Repeat HH, 5= HH unknown, 6= HH shifted*			
		M<br>F	**Date & Time**			
			**Outcome** *1=Inter, 2= Absent, 3= Refused 4= Repeat HH, 5= HH unknown, 6= HH shifted*			
		M<br>F	**Date & Time**			
			**Outcome** *1=Inter, 2= Absent, 3= Refused 4= Repeat HH, 5= HH unknown, 6= HH shifted*			

MEMA kwa Vijana Trial Further Survey (2007-2008)

Household Head Consent Form

1. I have been read the information sheet that explains the reasons for the study, and all the procedures that I and other members of my household will be asked to participate in.
2. All the questions I had about this study have been answered.
3. I clearly understand what I and other members of my household will be required to do.
4. I also know that I and other members of my household have the right to leave the study at any time if we do not want to continue.
5. I agree for this household to take part in the census for this study.

No.	NAME 1<br>(Name of the household member who is providing consent)	NAME 2<br>(Name of the household head if the person providing consent is not HH head)	Signature/<br>Thumb print
1			
2			
3			
4			
5			
6			
7			
8			
9			
10			
11			
12			
13			
14			
15			
16			
17			
18			
19			
20			

MEMA kwa Vijana Trial Further Survey (2007-2008)

Form ya Ridhaa kwa Mkuu wa Kaya

Tarehe:____/ ___/ ________, Staff Code: ______

Village:___________________, Kitongoji:________________

1. Nimesoma karatasi ya maelezo ambayo imefafanua sababu za utafiti na taratibu zote ambazo mimi na wakazi wa kaya yangu wataulizwa kushiriki.
2. Maswali yote niliyokuwa nayo kuhusu utafiti huu yamejibiwa .
3. Nimeelewa vizuri mimi na watu katika kaya yangu tutakachotakiwa kufanya.
4. Na pia nimefahamu mimi na wakazi wa kaya yangu kwamba tuna haki ya kuachakushiriki kwenye utafiti wakati wowote kama hatutajisikia kuendelea.
5. Nimekubali kaya hii kushiriki katika zoezi la sensa kwenye utafiti huu.

N0.	JINA 1<br>(Jina la mshiriki wa kaya aliyetoa ruhusa)	JINA 2<br>(Jina la mkuu wa kaya kama aliyetoa ruhusa siyo mkuu wa kaya)	Sahihi au Dole gumba<br>(Aliyetoa ruhusa)
1			
2			
3			
4			
5			
6			
7			
8			
9			
10			
11			
12			
13			

MEMA kwa Vijana Trial Further Survey (2007-2008)

14			
15			
16			
17			
18			
19			
20			
21			
22			
23			
24			
25			
26			
27			
28			
29			
30			
31			
32			
33			
34			
35			

MkV1FS Prot A24b_ HH consent (Swah 28May07).doc

MEMA kwa Vijana Trial Further Survey (2007-2008)

Invitation to Attend the Main Survey

What is MEMA kwa Vijana?

The MEMA kwa Vijana (MkV) Programme is a collaborative project of the government of Tanzania (the Ministry of Health & Social Welfare and the Ministry of Education & Vocational Training), the National Institute for Medical Research (NIMR), AMREF, the London School of Hygiene and Tropical Medicine, UK (LSHTM), the Liverpool School of Tropical Medicine (LSTM) and MRC Social and Public Health Sciences Institute, Glasgow, UK. The MkV programme is working in four districts of Mwanza Region (Misungwi, Sengerema, Geita, and Kwimba). The programme supports improved treatment of sexually transmitted diseases in health facilities, provides additional training for health workers, and provides sexual and reproductive health education to primary school pupils.

Why is the MkV Trial Further Survey being conducted?

During 2007 and 2008, there will be a survey to assess the impact of the intervention '***MkV1 Trial Further Survey'***. From previous surveys that were conducted between 2000 and 2002, we already know that the MEMA kwa Vijana intervention improved the knowledge and some reported sexual behaviours in young people in your community. This further survey will tell us if these improvements have been sustained, and whether the intervention has been effective in reducing the spread of HIV and other sexually transmitted infections in the longer-term. The information that we collect will help us to find the best ways to reduce the spread of HIV and other sexually transmitted diseases in your community.

What does the survey involve?

(1) A census of all households in the selected wards

(2) A main survey in which young people identified during the census will be interviewed.

Why have I received this invitation?

We have visited your household during the census and have found out that you are eligible to participate in the second part of the survey.

What exactly will happen to me if I attend the survey?

- If you come to the survey you will be asked some **questions**, and, if it is confirmed that you are eligible, you will be asked to provide a **small sample of urine and blood**.
- You will not have to answer any questions or give any samples if you do not want to.
- You will be able to change your mind and stop participating in the study at any time.
- You will have the opportunity to visit a **clinician** and to receive **free treatment** if you need it.
- You will also be given the choice of having a free, confidential HIV test (**VCT**).

MEMA kwa Vijana Trial Further Survey (2007-2008)

- In the unlikely event that you are found not to be eligible for the survey, you will receive half a bar of washing soap and TSh 2,000/= to cover your travelling expenses.

- If you are eligible for the survey, you will receive a full bar of washing soap and TSh 4,000/= to compensate you for your time and to cover your travelling expenses.

We hope that you will help us with our research. This is your invitation:

This is an invitation for <*name*> ________________________________

Please come to <*site*> ____________________ **in <*village*>** _____________

on <*day*> _______________________ **<*Date*>** ________________**200_**

Census ID number: ⊗ ⊗

What should I take with me to the survey?

- It is very important that you take **this invitation** with you to your appointment and that you do not give this invitation to anyone else.

- If you have previously participated in a MEMA kwa Vijana survey and still have the **MkV ID card** that you were given, then please bring that ID card with you when you come to your appointment.

- If you have any other kind of ID card or a **document with your date of birth or age** then please also take these to your appointment.

If you already know that you will not be able to come at that time then please tell me, and I shall be happy to try to arrange another time for you.

Further information: If you would like any further information on this project, please ask the representative who has brought you this information sheet. If you have additional questions or comments after (s)he has left, please address them to:
Ms. Aoife Doyle, Project Co-ordinator, MEMA kwa Vijana Trial Further Survey
Postal address: PO Box 11936, Mwanza
Location: National Institute for Medical Research, Isamilo, Mwanza
Telephone number: 2502203

- **Please feel free to discuss this information sheet with anyone else you may wish to consult.**
- **Thank you for your time and assistance.**

MEMA kwa Vijana Trial Further Survey (2007 – 2008)

Mwaliko wa kuhudhuria utafiti Mkuu

MEMA kwa Vijana ni mradi wa pamoja wa serikali ya Tanzania (Wizara ya Afya na Ustawi wa jamii Wizara ya Elimu na Mafunzo ya Ufundi), Taasisi ya Taifa ya Utafiti wa Magonjwa ya Binadamu (NIMR), AMREF, Chuo Kikuu cha Londoni cha Afya na Madawa katika Nchi za Joto (LSHTM- Uingereza), Chuo Kikuu cha Liverpool cha Madawa katika Nchi za Joto na Taasisi ya Afya ya Umma na Sayansi za Jamii (MRC, Glassgow, UK). Mradi huu unafanya kazi katika wiliaya nne za Mkoa wa Mwanza (Misungwi, Sengerema, Geita na Kwimba). Mradi unasaidia katika kuboresha matibabu ya magonjwa ya zinaa katika vituo vya afya, unatoa mafunzo ya ziada kwa wafanya kazi wa afya, na unatoa elimu ya afya ya uzazi kwa wanafunzi wa shule za msingi.

Katika mwaka 2007 na 2008 mradi utaendesha utafiti ili kutathmini matokeo ya utekelezaji wa mradi. Kutokana na tafiti za nyuma ambazo zilifanyika kati ya mwaka 2000 na 2002, tayari tunafahamu kwamba elimu ya MEMA kwa Vijana iliboresha ufahamu/elimu na baadhi ya mwienendo na tabia za kimapenzi kwa baadhi ya vijana katika jamii yako. Utafiti huu mwingine utatuambia kama maboresho haya yamekuwa endelevu na kama elimu hii imeweza kupunguza kasi ya maambukizi ya Virusi vya Ukimwi na magonjwa mengine ya zinaa kwa kipindi kirefu zaidi. Taarifa ambazo tutakusanya zitatusaidia kupata njia bora zaidi za kupunguza kuenea kwa virusi vya ukimwi na magonjwa mengine ya zinaa katika jamii yako.

Utafiti unahusisha nini?

(1) Sensa ya kaya zote katika kata zilizochaguliwa
(2) Utafiti Mkuu ambapo vijana waliotambuliwa wakati wa sensa watahojiwa.

Kwa nini mimi nimepata mwaliko?

Tulitembelea kaya yako wakati wa sensa na tumebaini ya kwamba unasitahili kushiriki sehemu ya pili ya utafiti

Ni yapi hasa yatatokea kwangu kama nikishiriki utafiti?

- Kama ukija kwenye utafiti utaulizwa baadhi ya **maswali**, na, kama ikadhibitika kuwa unasitahili, utaulizwa kutoa kiasi kidogo cha vipimo vya **mkojo na damu**.
- Unaweza usijibu swali lolote au kutoa kipimo kama hautapenda.
- Unaweza kubadili mawazo na kusimama kushiriki katika utafiti wakati wowote
- Utapata fursa ya kumtembelea mganga na kupata **matibabu ya bure** kama utaitaji.
- Pia utapewa chaguo la kupata **ushauri nasaha na upimaji wa virus vya ukimwi** bure
- Katiba tukio lisilotarajiwa ya kuwa hausitahili katika utafiri, utapata nusu **mche wa sabuni ya kufulia na shilling 2,000/=** kulipia gharama zako za usafiri.
- Kama unasitahili kwa utafiti utapata **mche mzima wa sabuni ya kufulia na shillingi 4,000/=** kufidia muda wako na kulipia gharama zako za usafiri..

MEMA kwa Vijana Trial Further Survey (2007 – 2008)

Tunatumaini utatusaidia katika utafiti wetu. Huu ndiyo mwaliko wako:

Huu ni mualiko wa <Jina> ________________________________

Tafadhali njoo kwenye <*eneo*> ___________________ katika <*kijiji*> ______

<*siku*> ______________________ <*Tarehe*> _______________200___

<saa> __________

Census ID number: ⊗ ⊗

Ni kipi cha kuja nacho kwenye utafiti?

- Ni muhimu kuchukua barua hii ya mwaliko na kuja nayo na usimpe mwaliko huu mtu yoyote.
- Kama kipindi cha nyuma ulishiriki katika utafiti wa **MEMA kwa Vijana** nabado unacho kitambulisho cha MKV ambacho ulipewa, tafadhali chukua kitambulisho hicho na njoo nacho.
- Kama unakitambulisho chochote au karatasi yenye tarehe ya kuzaliwa au umri wako, tafadhali pia chukua uje nacho.

Kama unajua kuwa hautaweza kuja katika muda huu, tafadhali niambie , na nitafurahi tukipanga muda mwingine na wewe.

Taarifa zaidi: Kama utapenda kupata taarifa zaidi kuhusu mradi huu, tafadhali mwambie mwakilishi aliyekuletea taarifa hii ya maelezo. Kama utakuwa na maswali ya ziada au maoni baada ya yeye kuondoka, tafadhali yatume kwa anwani ifuatayo:
Ms.Aoife Doyle, Project Co-ordinator, MEMA kwa Vijana Trial Further Survey
Anuani ya Posta: PO Box 11936, Mwanza
Mahali: Taasisi ya Taifa ya Utafiti wa Magonjwa ya Binadamu, Isamilo Mwanza
Simu: 2502203

- Tafadhali jisikie huru kujadili karatasi hii ya maelezo na mtu yeyote ambaye utapenda akupe ushauri.
- Ahsante kwa muda na msaada wako.

MEMA kwa Vijana Trial Further Survey (2007-2008)

Information Sheet about Main Survey for Parent/Guardian

The MEMA kwa Vijana Programme is a collaborative project of the government of Tanzania (the Ministry of Health & Social Welfare and the Ministry of Education & Vocational Training), the National Institute for Medical Research (NIMR), AMREF, the London School of Hygiene and Tropical Medicine, UK (LSHTM), the Liverpool School of Tropical Medicine (LSTM) and MRC Social and Public Health Sciences Institute, Glasgow, UK. The programme is working in four districts of Mwanza Region (Misungwi, Sengerema, Geita, and Kwimba). The programme supports improved treatment of sexually transmitted diseases in health facilities, provides additional training for health workers, and provides sexual and reproductive health education to primary school pupils.

During 2007 and 2008, the programme will conduct a survey to assess the impact of the intervention '***MkV1 Trial Further Survey'***. From previous surveys that were conducted between 2000 and 2002, we already know that the MEMA kwa Vijana intervention improved the knowledge and some reported sexual behaviours in young people in your community. This further survey will tell us if these improvements have been sustained, and whether the intervention has been effective in reducing the spread of HIV and other sexually transmitted infections in the longer-term. The information that we collect will help us to find the best ways to reduce the spread of HIV and other sexually transmitted diseases in your community.

We have already made a census of your household, and

<*name*> ______________________

has been selected to participate in the second part of our survey(the main survey).

As (s) he is not yet 18 years of age, we would like to ensure that you understand what will happen if he/she attends the main survey, and to let you know what you should do if you do not want him/her to participate in the survey.

- When the participant arrives at the survey point they will be greeted and some of the survey staff will ask some simple questions about his/her age and where he/she went to school.
- If it is confirmed that (s)he is eligible, an interviewer of the same sex as the participant will ask him/her some questions about sexual and reproductive health issues, and (s)he will be asked to provide a small sample of urine and blood.
- (S)he will not have to answer any questions or give any samples if (s)he does not want to.
- (S)he will be able to change his/her mind and stop participating in the study at any time.
- (S)he will have the opportunity to visit a clinician and to receive free treatment if (s)he needs it.
- (S)he will also be given the choice of having a free, confidential HIV test.
- In the unlikely event that (s)he is found not to be eligible for the survey, (s)he will receive half a bar of washing soap and TSh 2,000/=.
- If (s)he is eligible for the survey, (s)he will receive a full bar of washing soap and TSh 4,000/=.

MEMA kwa Vijana Trial Further Survey (2007-2008)

The participants name will not be written on any of the questionnaires, other forms, or specimen containers. Instead, numbers will be used. This is to make sure that only the senior researchers can know what they have answered, and which are the specimens that they have provided. All data and results will be kept secret, and any specimens that they give us will eventually be burnt, though they may be stored for many years at the National Institute for Medical Research in Mwanza before that is done.

We hope that you will allow him/her to help us with our research. But, if you do not want them to, please understand that we will not force you to agree, and that they will still be allowed to participate in other MEMA kwa Vijana Project activities.

Also, even if you agree for them to participate now, if you change your mind, you can withdraw them from the survey at any time, or can refuse for them to give one or more of the biological specimens.

If you do not want them to participate in the survey, please just tell them not to come to the survey.

However, if you do want to allow them to participate, please help them to remember the time to come to the survey which is written on their invitation, and give them permission to come to the survey point on that day.

Do you have any questions or want me to repeat anything I have just told you?

Further information: If you would like any further information on this project, please ask the representative who has brought you this information sheet. If you have additional questions or comments after (s)he has left, please address them to:

Ms. Aoife Doyle, Project Co-ordinator, MEMA kwa Vijana Trial Further Survey
Postal address: PO Box 11936, Mwanza
Location: National Institute for Medical Research, Isamilo, Mwanza
Telephone number: 2502203

- **Please feel free to discuss this information sheet with anyone else you may wish to consult.**

- **Thank you for your time and assistance.**

MEMA kwa Vijana Trial Further Survey (2007-2008)

MEMA kwa vijana utafiti wa majaribio 2007 – 2008

Taarifa kuhusu utafiti mkuu kwa wazazi

MEMA kwa Vijana ni mradi wa pamoja wa serikali ya Tanzania (Wizara ya Afya na Ustawi wa jamii Wizara ya Elimu na Mafunzo ya Ufundi), Taasisi ya Taifa ya Utafiti wa Magonjwa ya Binadamu (NIMR), AMREF, Chuo Kikuu cha Londoni cha Afya na Madawa katika Nchi za Joto (LSHTM- Uingereza), Chuo Kikuu cha Liverpool cha Madawa katika Nchi za Joto na Taasisi ya Afya ya Umma na Sayansi za Jamii (MRC, Glassgow, UK). Mradi huu unafanya kazi katika wiliaya nne za Mkoa wa Mwanza (Misungwi, Sengerema, Geita na Kwimba). Mradi unasaidia katika kuboresha matibabu ya magonjwa ya zinaa katika vituo vya afya, unatoa mafunzo ya ziada kwa wafanya kazi wa afya, na unatoa elimu ya afya ya uzazi kwa wanafunzi wa shule za msingi.

Katika mwaka 2007 na 2008 mradi utaendesha utafiti ili kutathmini matokeo ya utekelezaji wa mradi. Kutokana na tafiti za nyuma ambazo zilifanyika kati ya mwaka 2000 na 2002, tayari tunafahamu kwamba elimu ya MEMA kwa Vijana iliboresha ufahamu/elimu na baadhi ya mwienendo na tabia za kimapenzi kwa baadhi ya vijana katika jamii yako. Utafiti huu mwingine utatuambia kama maboresho haya yamekuwa endelevu na kama elimu hii imeweza kupunguza kasi ya maambukizi ya Virusi vya Ukimwi na magonjwa mengine ya zinaa kwa kipindi kirefu zaidi. Taarifa ambazo tutakusanya zitatusaidia kupata njia bora zaidi za kupunguza kuenea kwa virusi vya ukimwi na magonjwa mengine ya zinaa katika jamii yako.

Tayari tumeshafanya sensa kwenye Kaya yako, na

<jina>______________________________________

Amechaguliwa kushiriki katika hatua ya pili ya utafiti wetu (Utafiti Mkuu)

Kwa kuwa hajafikia umri wa miaka 18 tunataka kukuakikishia kuwa umeelewa kitakachotokea kama me/ke atashiriki katika utafiti mkuu na kukufahamisha cha kufanya kama hautapenda me/ke ashiriki kwenye utafiti

- Pale mshriki atakapofika kwenye eneo la utafiti atakaribishwa na baadhi ya wafanyakazi wa utafiti watamuuliza maswali rahisi kuhusu umri na wapi aliposomea shule ya msingi.

- Kama itaakikiwa kuwa anastahili, mhojaji wa jinsia inayofanana na mshiriki atamuuliza baadhi ya maswali yanayohusiana na afya ya uzazi na ataulizwa kutoa kiasi kidogo cha vipimo vya mkojo na damu

- Anaweza kukataa kujibu swali lolote au kutoa kipimo chochote kama ataamua kufanyahivyo.

- Anaweza kubadili mawazo na kuacha kushiriki katika utafiti wakati wowote.

- Atapata fulsa ya kumtembelea mganga na kupata matibabu ya bure.

MEMA kwa Vijana Trial Further Survey (2007-2008)

- Atapata nafasi ya kupata huduma ya ushauri na upimaji wa virusi vya ukimwi kwa hiari bure.
- Katika tukio lisilotarajiwa kuwa hatausika kwenye utafiti atapewa **nusu mche wa sabuni ya kufulia na shilingi 2,000/=**

- Kama anastahili kushiriki utafiti atapewa **mche mzima wa sabuni ya kufulia na shilingi 4,000/=**

Jina la mshiriki **halitaandikwa** sehemu yoyote ya dodoso, fomu nyingine au kikopo cha vipimo. Badala yake namba ndiyo zitatumika. Hii ni kuhakikisha kuwa watafiti wakuu tu ndiyo watajua kilichojibiwa, na vipimo vipi vimetolewa. Taarifa zote na matokeo yatahifadhiwa kwa usiri, na kila vipimo watakavyotupa sisi vitachomwa. Ingawa vitahifadhiwa stoo kwa miaka mingi katika Taasisi ya Taifa ya Utafiti wa Magonjwa ya Binadamu – Mwanza kabla ya kuchomwa.

Tunategemea utakubali vi/kijana wako atusaidie katika utafiti wetu.
Ingawa, kama hautapenda wa/ashiriki, basi elewa hatukulazimishi wewe ukubali, na pia wataendelea kuruhusiwa kushiriki katika shughuli nyingine za mradi wa MEMA kwa Vijana.

Pia, hata kama ukikubali washiriki sasa, kama utabadili mawazo, unaweza kuwaondoa kutoka kwenye utafiti wakati wowote, au kukataa kwao kutoa kipimo kimoja au zaidi cha kibiologia (damu na mkojo).

Kama <u>hautataka</u> washiriki katika utafitri, Tafadhali waambie wasije kwenye utafiti.

Hatahivyo, kama <u>unataka</u> kuwaruhusu washiriki, Tafadhali wasaidie kukumbuka muda wa kuja kwenye utafiti ambao utakuwa umeandikwa kwenye barua zao za mwaliko, wape ruhusa ya kuja kwenye eneo la utafiti kwa siku hiyo.

Je, una swali lolote au unataka nirudie chochote nilichokwisha kukueleza?

Taarifa zaidi: Kama utapenda kupata taarifa zaidi kuhusu mradi huu, tafadhali mwambie mwakilishi aliyekuletea taarifa hii ya maelezo. Kama utakuwa na maswali ya ziada au maoni baada ya yeye kuondoka, tafadhali waandikie kwa anwani ifuatayo:
Ms.Aoife Doyle, Project Co-ordinator, MEMA kwa Vijana Trial Further Survey
Anuani ya Posta: PO Box 11936, Mwanza
Mahali: Taasisi ya Taifa ya Utafiti wa Magonjwa ya Binadamu, Isamilo Mwanza
Simu: 2502203

- **Tafadhali jisikie huru kujadili karatasi hii ya maelezo na mtu yeyote ambaye utapenda akupe ushauri.**
- **Ahsante kwa muda na msaada wako**

MEMA kwa Vijana Trial Further Survey (2007-2008)

Fomu ya Ridhaa kwa Mzazi/ Mlezi juu ya Ushiriki wa Vijana chini ya Umri wa Miaka 18 kwenye Utafiti Mkuu.

Tarehe: ____/____/______, Namba ya Mtumishi: ________

Kijiji:___________________, Kitongoji:___________________, No. ya Kaya:____

1. Nimesoma karatasi ya maelezo inayoeleza juu ya utafiti mkuu (Taarifa kuhusu utafiti mkuu kwa wazazi)
2. Maswali yote niliyokuwanayo kuhusu utafiti huu yamejibiwa
3. Nimefahamu hatua zote muhimu za kiutafiti ambazo kijana/vijana wangu watatakiwa kufanya au kushiriki kama nitawaruhusu kushiriki. Hatua hizi ni pamoja na: -
    a. **Atapatiwa maelezo sahihi juu ya sababu za utafiti huu na taratibu zote**
    b. **Ataweza kuuliza maswali na kupatiwa majibu na watumishi wa mradi**
    c. **Ataelezwa haki yake/yao ya kushiriki kwa ridhaa na haki ya kutoendelea na ushiriki wakati wowote a(wa)kijisikia kufanya hivyo.**
    d. **Ataulizwa maswali machache na kuombwa kutoa kiasi kidogo cha mkojo na kiasi kidogo cha damu kwa ajili ya vipimo vya utafiti.**
    e. **Atahakikishiwa usiri kwa taarifa na matokeo ya vipimo atakavyo toa.**
    f. **Kama itaonekana ana ugonjwa unaotibika atapatiwa matibabu.**
    g. **Atapatiwa motisha kama fidia ya gharama za usafili na muda waliyo tumia.**
    h. **Pia atatakiwa kusaini karatasi za ushiriki wa ridhaa.**
4. Na pia nimefahamu kuwa nina haki ya kukubali au kukataa kijana/ vijana wangu kushiriki katika utafiti huu. Na hatakama nimekubali vijana wangu washiriki, ninaweza kukataa wakati wowote nikijisikia kufanyahivyo.
5. Ninakubali kwa hiari yangu mwenyewe kuwa Kijana wangu…

1.(Jina)________________________________,Census IDno.__/__/__/__/__/◙/__/__/◙/__/__/

ashiriki katika utafiti huu.

JINA KAMILI LA MZAZI/ MLEZI	SAHIHI AU ALAMA YA DOLE GUMBA	Tarehe

Mashahidi: Jina _________________________, sahihi_____________, tarehe:

MkV FS_Parental IC_under 18YP_30June07

MEMA kwa Vijana Trial Further Survey (2007-2008)

MkV1FS: Moved Away Form

<table>
<tr><td>1.1</td><td>Staff code</td><td>|__||__||__|</td></tr>
<tr><td>1.2</td><td>Date</td><td>|__||__|/|__||__|/200|__|</td></tr>
<tr><td>1.3</td><td>Census ID number</td><td>|__||__||__||__||__||__||__||__|</td></tr>
<tr><td>1.4</td><td colspan="2">Name of Eligible Young Person:</td></tr>
<tr><td>1.5</td><td colspan="2">Other names (if any):</td></tr>
<tr><td>1.6</td><td>Who provided this information?
01=Parents; 02=Other relatives; 03=Neighbors; 05=Village or Sub-village Leaders; 05=Other

(Specify____)</td><td>|__||__|</td></tr>
<tr><td>1.7</td><td>Why did s(he) move? Write the main reason
01=Work; 02=To live with relatives; 03=Education; 04=Marriage/regular partnership; 05=Other

(Specify____)</td><td>|__||__|</td></tr>
<tr><td>1.8</td><td>Is the person moved a way permanently?

If NO→ when will the person come back?
01= within 1 week; 02= within 2 weeks; 03= within 3-4 weeks; 04= > than 4 weeks; 99= Not known</td><td>Yes 1
No 2

|__||__|</td></tr>
</table>

2 Place where s(he) moved NK=Not Known

<table>
<tr><td>2.1</td><td>District <write name> :</td><td></td></tr>
<tr><td>2.2</td><td>Ward <write name>:</td><td></td></tr>
<tr><td>2.3</td><td>Village/Town <write name></td><td></td></tr>
<tr><td>2.4</td><td>Kitongoji/neighbourhood/street <write name> :</td><td></td></tr>
<tr><td>2.5</td><td colspan="2">Head of Household <full name>:</td></tr>
<tr><td>2.6</td><td colspan="2">Name of husband or wife <<i>If marriage status known</i>>
____</td></tr>
<tr><td>2.7</td><td colspan="2">Other contact person at new place <name>:</td></tr>
</table>

MEMA kwa Vijana Trial Further Survey (2007-2008)

2.8	*Please write any other information that can help tracing (e.g. other contact person, details of house location, school, work and work place)*

MEMA kwa Vijana Trial Further Survey (2007-2008)

Moved Away Form (Swahili)

Village: ____________________, Kitongoji: ______________________

1.1	Namba ya siri ya mtumishi	|__||__||__|
1.2	Tarehe	|__||__|/|__||__|/200|__|
1.3	Namba ya siri ya sensa (Mwaliko)	⊗ ⊗
1.4	Jina la Kijana:	
1.5	Majina mengine (kama yapo)	
1.6	Nani aliyetoa taarifa hizi?<br>01=Mzazi/mlezi; 02= Ndugu wa karibu; 03= Jirani; 05= kiongozi wa jamii; 05= mwingine	|__||__|
1.7	Kijana ameondoka kwa sababu gani?<br>01= kikazi; 02= kuishi na ndugu; 03= masomo; 04= kuishi na mme/mke au mpenzi; 05= Nyingine	|__||__|
1.8	Je, Kijana ameondoka kimoja? (zungushia moja)<br><br>Kama HAPANA→ Anategemewa kurudi lini?<br>01= Ndani ya wiki 1; 02= ndani ya wiki 3; 03= zaidi ya wiki 3; 99= haifahamiki	Yes 1<br>No 2<br>|__||__|

2 Sehemu aliyohamia

2.1	Wilaya ____	
2.2	Kata ____	
2.3	Kijiji ____	
2.4	Kitongoji/mtaa/barabara ____	
2.5	Jina la mkuu wa kaya <anakoishi kwa sasa>____	
2.6	Jina la mke au mme *<kama ameoa/ameolewa>*<br>____	
2.7	Jina la mtu mwingine wa kumuuliza <sehemu ya sasa> ___________________________________	
2.8	*Tafadhari andika taarifa zingine zitakazo weza kusaidia kumpata huyu kijana (mf: annuani nyingine; maelezo ya sehemu nyumba anayoishi ilipo; shule anayo soma; kazi anazofanya na eneo zilizopo.*<br>____<br>____<br>____<br>____<br>____<br>____<br>____<br>____	

MEMA kwa Vijana Trial Further Survey (2007-2008)

MkV1FS: PDA and GPS Borrowing Form

S/no.	Name	Code no.	PDA no.	GPS no	Date issued	Signature	Date returned	Signature	Other information
1									
2									
3									
4									
5									
6									
7									
8									
9									
10									

MEMA kwa Vijana Trial Further Survey (2007-2008)

MKV1 FS: Daily Progress with census

Census Team Leader staff code		|__||__||__|
Community name & code		|__||__|
Village name (s) & code (s)		|__||__||__
__||__||__|
Date		

Kitongoji	CI (staff code)	Number of census Hours	Number of Households visited				Number of eligible YP		
			Interviewed	Refused	Absent	HH moved	Invited	Moved Away	Parent consent

MEMA kwa Vijana Trial Further Survey (2007-2008)

MkV1FS: Census Village Report

Date of report:
Team Leader:
Community:
Village:
Team:

(1) SUMMARY STATISTICS

	Households									Invited YP			Other	
**Vitongoji**	*Mobilisation list*	*Add HH*	*Interv*	*Refuse*	*Repeat*	*Not known*	*Shifted*	*Not interv - visited 3 times*	*Not interv- visited <3 times*	*M*	*F*	*Total*	*Moved away Forms*	*Parental Consent Forms*
**TOTAL**														

MEMA kwa Vijana Trial Further Survey (2007-2008)

	Name of Kitongoji	Total number of Household	Date(s) of census	YP Invited to the Survey	
				SURVEY DATE	NO. INVITED
01					
02					
03					
04					
05					
06					

MEMA kwa Vijana Trial Further Survey (2007-2008)

MkV1FS: Census Community Report

**Date of report:**	
**Team Leader:**	
**Community:**	
**Team:**	

(1) SUMMARY STATISTICS

	**Households**									**Invited YP**			**Other**	
**Village**	*Mobilisation list*	*Add HH*	*Interv*	*Refuse*	*Repeat*	*Not known*	*Shifted*	*Not interv - visited 3 times*	*Not interv- visited <3 times*	*M*	*F*	*Total*	*Moved away Forms*	*Parental Consent Forms*
**TOTAL**														

MEMA kwa Vijana Trial Further Survey (2007-2008)

(2) NARRATIVE SUMMARY
(Observations, Issues arising, immediate solutions, Proposed Solutions)

(a) Preparation, Logistics and IT
(b) Census Interviewers
(c) Census Team Leader
(d) Driver

MEMA kwa Vijana Trial Further Survey (2007-2008)

Conclusions and recommendations
(What lessons have you learnt and what do you recommend for the future?)

MEMA kwa Vijana Trial Further Survey (2007-2008)

Information Sheet for Participants

The MEMA kwa Vijana Programme is a collaborative project of the government of Tanzania (the Ministry of Health & Social Welfare and the Ministry of Education & Vocational Training), the National Institute for Medical Research (NIMR), AMREF, the London School of Hygiene and Tropical Medicine, UK (LSHTM), the Liverpool School of Tropical Medicine (LSTM) and MRC Social and Public Health Sciences Institute, Glasgow, UK. The programme is working in four districts of Mwanza Region (Misungwi, Sengerema, Geita, and Kwimba). The programme supports improved treatment of sexually transmitted diseases in health facilities, provides additional training for health workers, and provides sexual and reproductive health education to primary school pupils.

During 2007 and 2008, the programme will conduct a survey to assess the impact of the intervention '***MkV1 Trial Further Survey'***. From previous surveys that were conducted between 2000 and 2002, we already know that the MEMA kwa Vijana intervention improved the knowledge and some reported sexual behaviours in young people in your community. This further survey will tell us if these improvements have been sustained, and whether the intervention has been effective in reducing the spread of HIV and other sexually transmitted infections in the longer-term. The information that we collect will help us to find the best ways to reduce the spread of HIV and other sexually transmitted diseases in your community.

Checking eligibility:
You have been invited to attend this survey. Thank-you for coming here today. First we will need to check whether you are really eligible for the main survey. To do this, one of our staff will ask you a few simple questions about your age and where you went to school.

If you are not eligible:
In the unlikely event that you are not eligible, you will not need to do anything more, but will be offered the chance to see a clinician who will check whether you have any illness and will provide free treatment if you need it. (S)he will also offer you the choice of having a free, confidential HIV test.

You will also be given half a bar of washing soap and TSh 2,000/= to cover your travelling expenses.

Your name will not be written on any of the forms. Instead, numbers will be used. This is to make sure that only the senior researchers can know what you have answered. All the data you have provided will be kept secret.

MEMA kwa Vijana Trial Further Survey (2007-2008)

If you are eligible:

If you are found to be eligible, in a few minutes, if you agree to participate, a young interviewer of the same sex as yourself will ask you some questions about sexual and reproductive health issues. You will then be asked to provide a small amount of urine, and a small sample of blood. If you do not want to provide any of these specimens, you will not be forced to. The blood and urine will be tested for HIV and other sexually transmitted infections, and may be tested for other infections in the future. You will also be seen by a project clinician, who will provide free treatment if you need it. You can also visit the counsellor and have a free test for HIV if you want to do that.

You will be given a bar of washing soap and Tsh 4,000/= to thank you for sparing time to participate in this research.

Your name will not be written on any of the questionnaires, other forms, or specimen containers. Instead, numbers will be used. This is to make sure that only the senior researchers can know what you have answered, and which are the specimens you provided. All data and results will be kept secret, and any specimens you give us will eventually be burnt, though they may be stored for many years at the National Institute for Medical Research in Mwanza before that is done.

We would like you to participate in all of these survey activities. But, if you do not want to, please understand that we will not force you to. Also, even if you agree to participate now, if you change your mind, you can withdraw from the survey at any time, or can refuse to give one or more of the biological specimens.

Further information:

- Does anyone have any questions or want me to repeat anything I have just told you?

- If you have additional questions or comments after we have left, you will find the name and address of the project on the paper you were given when one of us visited your home previously. Does anyone want another copy of that paper?

- Does anyone have any further questions?

MEMA kwa Vijana Trial Further Survey (2007-2008)

MkV1FS: Maelezo ya mshiriki

MEMA kwa vijana ni mradi wa pamoja wa serikali ya Tanzania (Wizara ya afya na ustawi wa jamii, Wizara ya Elimu na mafunzo yaufundi) Taasisi ya Taifa ya Utafiti wa magonjwa ya Binadamu (NIMR), AMREF, Chuo kikuu cha Londoni cha Afya na madawa katika Nchi za joto na Taasisi ya Afya ya Umma na sayansi za jamii (MRC, Glassgow, UK).Mradi huu unafanya kazi katika wilaya nne za mkoa wa Mwanza (Misungwi, Sengerema, Geita na kwimba) Mradi unasaidia katika kuboresha matibabu ya magonjwa ya zinaa katika vituo vya afya, unatoa mafunzo ya ziada kwa wafanya kazi wa afya, na utoaji elimu ya afya ya uzazi kwa wanafunzi wa shule za msingi.

Katika mwaka 2007 na 2008 mradi utaendesha utafiti ili kutathmini matokeo ya utekelezaji wa mradi. Kutokana na tafiti za nyuma ambazo zilifanyika kati ya mwaka 2000 na 2002, tayari tunafahamu kwamba elimu ya MEMA kwa vijana iliboresha ufahamu/elimu na baadhi ya mienendo na tabia za kimapenzi kwa baadhi ya vijana katika jamii yako. Utafiti huu mwingine utatuambia katika maboresho haya yamekuwa endelevu na kama elimu hii imeweza kupunguza kasi ya maambukizi ya virusi vya ukimwi zitatusaidia kupata njia bora zaidi za kupunguza kuenea kwa virusi vya ukimwi na magonjwa mengine ya zinaa katika jamii yako.

<u>KUHAKIKI USHIRIKI</u>

Umealikwa kushiriki utafiti, asante kwa kufika hapa leo, kwanza tengependa kuhakiki kama unastahili kwa ajili ya utafiti, kwa kufanya hivi, mmoja kati yetu atakuuliza maswali machache kuhusu umri wako na kuhusu muda ulio kuwa shule ya msingi.

<u>KAMA HAUSTAHILI KUSHIRIKI</u>

Umeshapata mwaliko kuhudhuria utafiti
Asante sana kuhudhuria leo. Kwanza tutataka kuhakikisha kama unastahili kuwa ndani ya tafiti huu mkuu. Ili kufanya hivyo mmoja kati ya wafanyakazi atakuuliza maswali rahisi kama umri wako na wapi ulisoma.

Kama Haustahili kushiriki.

Kwa bahati mbaya kama haustahili kuwa katika mradi huu wa utafiti hutahitajika kufanya kitu chochote bali utapatiwa fursa ya kuonana na mganga upimwe kama utakutwa na magonjwa utapata matibabu bure. Vilevile utapata ushahuri nasaha na kupima virusi vya ukimwi bure.

Utapatiwa nusu mche wa sabuni na shilingi 2,000/= kwa ajili ya usafiri wa kukurudisha nyumbani.

Jina lako alitaandikwa kwenye karatasi yoyote bali namba zitatumika. Hii ni kukuakikishia kwamba mtafiti mkuu tu ndiye atajua ulichojibu. Takwimu zote ulizotoa zitatunzwa kwa usiri.

MEMA kwa Vijana Trial Further Survey (2007-2008)

Kama unastahili kushiriki
Kama unafaa kuwa kwenye mradi tutakuwa na wewe kwa muda mfupi na kama utakubali kushiriki utahojiwa na mtu mnayelingana umri na jinsia moja atakuuliza maswali ya kuhusu elimu ya uzazi, utaulizwa kutoa mkojo kidogo na damu. Damu na mkojo vitapimwa virusi vya ukimwi na magonjwa mengine yanayoambukizwa kwa kufanya ngono zembe na unaweza kupimwa magonjwa mengine kwa baadaye (maishani). Vilevile utaonwa na mganga wa mradi ambaye atakupima na kupewa dawa bure kama utakutwa na matatizo ya kiafya.
Unaweza kuonana na mshauri nasaha na kupima virusi vya ukimwi kama utapenda bure.
Utapatiwa mche wa sabuni wa kufulia na shilingi elfu nne (4000/=) kwa kukushukuru kutumia muda wako kwa kushiriki katika utafiti.

Jina lako halitaandikwa kwenye karatasi yoyote ya maswali na fomu nyingine zozote au kwenye chombo chochote cha vipimo badala yake zitatumika nambari tu. Hii ni kuhakikisha kwamba ni watafiti wakuu tu ndiyo watafahamu nikitu gani ulichokijibu na vipimo ulivyo toa.

Majibu na taarifa zote zitabakia kuwa siri na vipimo ulivyotoa vitateketezwa kwa moto ingawaje vinaweza kutunzwa (National Institute for Medical Research Mwanza kwa muda mrefu (miaka)
Tungependa ushiriki katika shughuli zote za utafiti. Lakini kama hauhitaji hatutakulazimisha kufanya hivyo. Hata kama utakubali kushiriki sasa kama ukibadili mawazo unaweza ukajitoa katika utafiti wakati wowote. Au unaweza ukakataa kutoa vipimo.

- Je kuna mtu anaswali au nirudie nilivyokwisha kusema?
- Kama una ziada au maoni baada ya sisi kutoka, utapata jina la mratibu na anwani ya mradi kwenye karatasi uliyopewa na mmoja wetu alipowatembelea nyumbani siku za nyuma.
  Kuna yeyote anayetaka nakala ya zile karatasi za mwanzoni?
- Kuna yeyote mwenye swali zaidi?

MEMA kwa Vijana Trial Further Survey (2007-2008)

MkV1FS: WALKMAN LENDING FORM

Date	Walkman S/N	Attendee Name	Attendee Signature/print	Time Given	Time Returned	Lending Staff Code/ No	Lending Staff Signature

MEMA kwa Vijana Trial Further Survey (2007-2008)

Date	Walkman S/N	Attendee Name	Attendee Signature/print	Time Given	Time Returned	Lending Staff Code/ No	Lending Staff Signature

MEMA kwa Vijana Trial Further Survey (2007-2008)

Informed consent check questions.
Maswali ya kupima uelewa kwa mshriki.

1. Mradi wa mema kwa vijana unafanya kazi katika wilaya zipi za mkoa wa Mwamza?
2. Mradi wa Mema kwa Vijana umelenga kutoa elimu inayohusiana na nini zaidi?
3. Tunapima kuona kama elimu ya Mema kwa Vijana imepunguza kasi ya maambukizi kwa magonjwa yapi?
4. Kama haustahili kushiriki utapewa fursa ya kuonana na watu wapi katika mradi kwa hudhurio lako la leo?
5. Kama haustahili kushiriki utapewa nini kama ahsante kwa kuhudhuria?
6. Vipimo vyote vitakavyochukuliwa na maswali yote utakayojibu yatakuwa ni siri kati yako na nani?
7. Kama unastahili kushiriki vipimo gani utachukuliwa kwa siku ya leo?
8. Vipimo vya damu na mkojo vitapimwa nini?
9. Kama unastahili kushiriki baada ya kumaliza utapewa nini?
10. Badala ya kukutumia jina katika vipimo tutatumia nini ili kutunza siri?
11. Vipimo vyote vitatunzwa wapi kama ikihitajika viweze kupimwa tena hapo baadae?
12. Je ni lazima kukubari kushiriki katika utafiti huu? Na kama utakubari kushiriki unaweza ukaacha wakati gani?
13. Kama mtu anahitaji kuwasiliana na uongozi wa mradi atatumia anwani ipi baada ya timu ya watafiti kuondoka kijijini hapa?

MEMA kwa Vijana Trial Further Survey (2007-2008)

Participant Consent Form

1. I have been read the information sheet that explains the reasons for the study, and all the procedures that I am being asked to participate in.
2. All the questions I had about this study have been answered.
3. I clearly understand what I will be required to do, and what will be done to me, if I agree to participate in this study.
4. I also know that I have the right to leave the study at any time if I do not want to continue.
5. I know that I will be offered free treatment for sexually transmitted infections today, if the clinician thinks this is needed.
6. I know that I will be offered voluntary HIV counselling and testing.
7. I am aware that all the information that I give, all the findings of the clinician, and all the results of the laboratory tests, will be kept secret.
8. I am aware that if I am found to have any curable, harmful infections when my specimens are tested later, a clinician will return to my village to offer me treatment, unless I say I do not want this on this sheet.
9. I agree to take part in this study.

**No.**	**Name (*in capital letters*)**	**Later treatment if needed (1=Yes; 2=No)**	**Signature/ Thumb print**	**Sticker**
1				
2				
3				
4				
5				
6				
7				
8				
9				
10				
11				
12				
13				
14				
15				

MEMA kwa Vijana Trial Further Survey (2007-2008)

Fomu ya Ushiriki kwa Ridhaa

Tarehe: ____ /____/________, Staff code: _______

Kijiji: ___________________, Kitongoji: _______________

1. Nimesoma karatasi ya maelezo inayoeleza sababu ya utafiti huu,na taratibu zote nilizoulizwa ili kushiriki.
2. Maswali yote kuhusiana na utafiti huu yamejibiwa.
3. Nimeelewa ninachotakiwa kufanya ,na nitakachofanyiwa mimi,kama nitakubali kushiriki katika utafiti.
4. Na pia ninafahamu kuwa nina haki ya kutoendela kushiriki kwenye utafiti kama sitajisikia kuendelea.
5. Ninajua kuwa nitapata matibabu ya bure ya magonjwa ya zinaa leo,kama mganga ataona inahitajika.
6. Ninajua kwamba nitapata ushauri nasaha kuhusu virusi vya ukimwi na vipimo.
7. Nina fahamu kwamba taarifa zote nilizotoa ,na uchunguzi wote wa mtabibu,na pia matokeo yote ya vipimo vya maabara,yatatunzwa kwa siri.
8. Ninafahamu kwamba kama itaonekana nina ugonjwa wowote unaotibika, na maambukizi wakati vipimo vyangu vitakapo pimwa hapo baadae, mganga atarudi kijijini kunipatia matibabu, isipokuwa kama nitasema sihitaji matibabu ya baadaye kwenye karatasi hii.
9. Nimekubali kushiriki katika utafiti huu.

**Jina (herufi kubwa).**	**Matibabu ya baadae kama yatahitajika** (1= Ndiyo, 2= Hapana)	**Sahihi au *Dole gumba.***	**Sticker.**

MEMA kwa Vijana Trial Further Survey, NIMR, PO Box 11936, Mwanza

CONTACT REFERRAL SLIP

Clinician's Name:…………………………………………………………………………………

Date of diagnosis: Date|__||__|/ Mth|__||__|/ Yr 20|__||__|

Index Sticker No.: |__||__||__||__||__||__||__||__||__|

Ndugu;
Inawezekana unalo tatizo la afya, lakini hujajisikia vibaya.
Tafadhali, nenda na karatasi hii kwenye kituo cha afya kilicho karibu na wewe.
Wafanyakazi wa kituo watakuona, pia watatoa matibabu.

Message to the Clinician at the referral health unit: Please treat this person. (S)he is a contact of someone I treated for the condition (s) ***(circle condition(s))***:

**GUS**	**VDS**	**PID**
**UDS**	**Epididymo-orchitis**	**Bubo**
**PSS**	**Scabies**	

Other condition (specify): ____

……

MEMA kwa Vijana Trial Further Survey, NIMR, PO Box 11936, Mwanza

CONTACT REFERRAL SLIP

Clinician's Name:…………………………………………………………………………………

Date of diagnosis: Date|__||__|/ Mth|__||__|/ Yr 20|__||__|

Index Sticker No.: |__||__||__||__||__||__||__||__||__|

Ndugu;
Inawezekana unalo tatizo la afya, lakini hujajisikia vibaya.
Tafadhali, nenda na karatasi hii kwenye kituo cha afya kilicho karibu na wewe.
Wafanyakazi wa kituo watakuona, pia watatoa matibabu.

Message to the Clinician at the referral health unit: Please treat this person. (S)he is a contact of someone I treated for the condition (s) ***(circle condition(s))***:

**GUS**	**VDS**	**PID**
**UDS**	**Epididymo-orchitis**	**Bubo**
**PSS**	**Scabies**	

Other condition (specify): ____

MEMA kwa Vijana Trial Further Survey (2007-2008)

MEMA kwa Vijana Trial Further Survey: Request for HIV Test Result

Section A: **[To be completed by the Counsellor]**

**1.**	Sticker No.	|__||__||__||__||__||__||__||__||__|
**2.**	Staff code	|__||__||__|
**3.**	Date	|__||__|/|__||__|/20|__||__|
**4.**	Name of person requesting their HIV result:	

Section B. **[To be read by/to the participant]**
Today, I have received counselling about knowing the result of an HIV test that will be performed on my blood.

This has included a full explanation of:

- ❑ What an HIV test is.
- ❑ That is possible for someone who has been infected recently to test negative.
- ❑ The difference between HIV infection and AIDS, and the fact that someone can be infected by the HIV virus for many years before developing any illness (AIDS) and a discussion of the potential advantages and disadvantages of knowing my HIV test result.

Section C. **[To be completed by the participant]**

I want to be told the result of the HIV test on my blood:

Signature:

……………………………………………………………………..

Date: |__||__| / |__||__| / 20|__||__|
Date **Month** **Year**

MEMA kwa Vijana Trial Further Survey (2007-2008)

Request for HIV Test Result Form

SEHEMU A: [IJAZWE NA MSHAURI]

1.	Namba ya Sticker	|__| |__| |__| |__| |__| |__| |__| |__| |__|
2.	Namba ya Mshauri	|__| |__| |__|
3.	Tarehe	|__| |__| /|__| |__|/20 |__| |__|
4.	Jina la anayetaka kupima	______________________________

SEHEMU B: [ISOMWE NA/AU KWA MSHIRIKI]

Leo, nimepokea ushauri nasaha juu ya kipimo cha maambukizi ya virusi vya ukimwi ambacho kitafanywa kwenye damu yangu kwa hiari:

Hii imejumuisha maelezo kamili juu ya:

1. Nini maana ya virusi vya Ukimwi
2. Kwamba inawezekana kwa mtu aliyeambukizwa hivi karibuni akakutwa hana maambukizi
3. Tofauti kati ya maambukizi ya virusi vya ukimwi na ukimwi, pia inawezekana mtu akapata maambukizi ya virusi vya ukimwi na akaishi navyo kwa muda mrefu kabla hajaugua ukimwi, na majadiliano yanayohusu faida na hasara ya kujua majibu ya maambukizi ya virusi vya ukimwi

SEHEMU C: [IJAZWE NA MSHIRIKI]

Nahitaji kuambiwa matokeo/majibu ya kipimo cha maambukizi ya virusi vya ukimwi kwenye damu yangu:

Sahihi ya mshiriki: ……………………………………………………………………..

Tarehe: **|__| |__| /|__| |__|/20 |__| |__|**

MEMA kwa Vijana Trial Further Survey (2007-2008)

VCT Discordant Results Form

Counsellor to submit form to MkV1 FS office when Section A completed.
Section B and C will be completed by MkV1 FS office.

SECTION A (By counsellor)		
Team	ST1 ST2	
Staff code of counsellor	|__||__||__|	
Date of rapid test	|__||__|/ |__||__|/ |__||__|	
STICKER NUMBER		
Rapid Result	Bioline<br>P N	Determine<br>P N
Details of Test kit	Bioline- Stock #:	Determine- Stock #:
Appointment Date + Time<br>*Should be 4 weeks after the date of rapid test*	Date: |__||__|/ |__||__|/ |__||__| Time (English): |__||__|:|__||__|	
Appointment Location<br>*Write community and village only- personal details of participant should not be written here*		
SECTION B (By MkV1 FS Office)		
Final ELISA Result	P N D	
Staff code of person receiving final ELISA result	|__||__||__|	
Date result received	|__||__|/ |__||__|/ |__||__|	
SECTION C (By MkV1 FS Office)		
Date result delivered to participant	|__||__|/ |__||__|/ |__||__|	
Staff code of counsellor	|__||__||__|	
Comments		

MEMA kwa Vijana Trial Further Survey (2007-2008)

HIV VCT Results Form

1. Sticker Number	|__||__||__| |__||__| |__||__||__||__|
2. Result Test 1 (Bioline)<br>(P=Positive; N=Negative; X=Not Done)	|__| <br>Test Expiry Date: |__||__| **Mth**|__||__| **Yr**|__||__|
3. Result Test 2 (Determine)<br>(P=Positive; N=Negative; X=Not Done)	|__| <br>Test Expiry Date: |__||__| **Mth**|__||__| **Yr**|__||__|
4. Overall HIV Result<br>(P=Positive; N=Negative; Indeterminate;<br>X=Not Done)	|__|
5. Date of Results	**Date**|__||__| **Mth**|__||__| **Yr**|__||__|
6. Counsellor code	|__||__||__|
7. Signature of Counsellor	………………………………………………….

HIV VCT Results Form

1. Sticker Number	|__||__||__| |__||__| |__||__||__||__|
2. Result Test 1 (Bioline)<br>(P=Positive; N=Negative; X=Not Done)	|__| <br>Test Expiry Date: |__||__| **Mth**|__||__| **Yr**|__||__|
3. Result Test 2 (Determine)<br>(P=Positive; N=Negative; X=Not Done)	|__| <br>Test Expiry Date: |__||__| **Mth**|__||__| **Yr**|__||__|
4. Overall HIV Result<br>(P=Positive; N=Negative; I=Indeterminate;<br>X=Not Done)	|__|
5. Date of Results	**Date**|__||__| **Mth**|__||__| **Yr**|__||__|
6. Counsellor code	|__||__||__|
7. Signature of Counsellor	………………………………………………….

MkV1FS Protocol

MEMA kwa Vijana Trial Further Survey

SLIP TO BE RETAINED BY REFERRER

PLEASE DO NOT PHOTOCOPY THIS FORM. USE ONLY FORMS FROM MkV1FS

Form number 10000101 version **1.01**

Today's date (ddmmyy) ☐☐ ☐☐ ☐☐

Family name ☐

EXAMPLE ONLY

First name ☐

Middle name ☐

Date of birth (ddmmyy) ☐☐ ☐☐ ☐☐ **Age (yrs)** ☐☐

Sex M ☐ F ☐

Ward ☐

Village ☐

Subvillage ☐

Pregnant Yes ☐ No ☐ don't know ☐

Referred to ☐

Referred for CTC ☐ VCT ☐ PMTCT ☐

Referred by ☐ (counsellor's name)

MkV1FS ID number ☐

CUT ALONG DOTTED LINE

MEMA kwa Vijana Trial Further Survey (2007-2008)

MKV1 FS: Tracing Form

VILLAGE NAME: ____________________ *Code* :_____ STAFF CODE: ___________ DATE: ________________

S/NO	Jina la Kitongoji	Namba ya Mwaliko	Tarehe ya mwaliko	Jina la Kwanza la Kijana	Jina la pili la kijana	Jinsia	Majina kamili ya Mkuu wa kaya	1. Sababu za kutofika<br>2. Matokeo ya Ufuatiliaji
001.						M<br>F		1.<br>2.
002.						M<br>F		1.<br>2.
003.						M<br>F		1.<br>2.
004.						M<br>F		1.<br>2.
005.						M<br>F		1.<br>2.
006.						M<br>F		1.<br>2.
007.						M<br>F		1.<br>2.
008.						M<br>F		1.<br>2.
009.						M<br>F		1.<br>2.

MEMA kwa Vijana Trial Further Survey (2007-2008)

MKV1 FS: Survey Village Report Form

First Date of survey in village		|__||__|/|__||__|/ 20|__||__|
Survey Team Leader staff code		|__||__||__|
Community name & code		|__||__|
Village name & code		|__||__|

		**Date of Survey:**					
**Received invitation from Census Team**		**Male**					
		**Female**					
		**TOTAL**					
**Attended and Invited to Survey**	**E**	**Male**					
		**Female**					
		**TOTAL**					
		MkV Male					
		MkV Female					
		MkV TOTAL					
	**NE**	**Male**					
		**Female**					
		**TOTAL**					
	**R**	**Male**					
		**Female**					
		**TOTAL**					
**Attended and Not Invited to Survey**	**E**	**Male**					
		**Female**					
		**TOTAL**					
		MkV Male					
		MkV Female					
		MkV TOTAL					
	**NE**	**Male**					
		**Female**					
		**TOTAL**					
	**R**	**Male**					
		**Female**					
		**TOTAL**					
**TOTAL**	**E**	**Male**					
		**Female**					
		**TOTAL**					
		MkV Male					
		MkV Female					
		MkV TOTAL					
	**NE**	**Male**					
		**Female**					
		**TOTAL**					
	**R**	**Male**					
		**Female**					
		**TOTAL**					
**TOTAL ATTENDEES**							

MEMA kwa Vijana Trial Further Survey (2007-2008)

Observations/ Challenges

Date	Team members *(eg RI, SI, Lab, Clin, Couns, Tr, TL, All)*	Issue	Immediate Solution	Proposed Solution	Recommendations

MEMA kwa Vijana Trial Further Survey (2007-2008)

**Date**	**Team members** *(eg RI, SI, Lab, Clin, Couns, Tr, TL, All)*	**Issue**	**Immediate Solution**	**Proposed Solution**	**Recommendations**

MEMA kwa Vijana Trial Further Survey (2007-2008)

MKV1 FS: Survey Community Report Form

Date of report	|__||__|/|__||__|/ 20|__||__|
Survey Team Leader staff code	|__||__||__|
Community name & code	|__||__|
Dates of survey in community	

(1) SUMMARY STATISTICS

PLEASE ATTACH PRINTED COPY OF COMPLETED EXCEL SPREADSHEET FOR THE COMMUNITY AND COMPLETE THE FOLLOWING TABLE.

	Males	Females	TOTAL
**TOTAL INVITED**			
**- eligible**			
**- MkV1**			
**- non-eligible**			
**- not attended**			
**TOTAL NON-INVITED**			
**- eligible**			
**- MkV1**			
**- non-eligible**			
**TOTAL ELIGIBLE**			
**Total MkV1 (0)**			
**Total MkV1 (1)**			
**Total MkV1 (2)**			
**Total MkV1 (3)**			
**Total MkV1 (99)**			
**Refused Informed Consent**			
**Refused Blood sample**			

(2) NARRATIVE SUMMARY
(Observations, Issues arising, immediate solutions, Proposed Solutions)

(a) Preparation and Logistics

(b) Registration Interviewers
(c) Survey Interviewers
(d) Laboratory Technicians

(e) Clinician
(f) Counsellors
(g) Tracers

MEMA kwa Vijana Trial Further Survey (2007-2008)

(h) Team Leader
(I) Driver

MEMA kwa Vijana Trial Further Survey (2007-2008)

Conclusions and recommendations
*(*What lessons have you learnt and what do you recommend for the future?)

MEMA kwa Vijana Trial Further Survey (2007-2008)

MkV1FS: Supervision Report Form

**Date(s) of supervision visit:**
**Supervisor(s):**
**Location:**
**Team(s):**
**Purpose of supervision:**

Mobilisation Team
(Observations, Issues arising, immediate solutions, Proposed Solutions)

(1) Preparation and Logistics
(2) Mobilisation Officer
(3) Proposed survey venues and field team accommodation

MEMA kwa Vijana Trial Further Survey (2007-2008)

Census Team

(Observations, Issues arising, immediate solutions, Proposed Solutions)

(1) Preparation, Logistics and IT
(2) Census Interviewers
(3) Census Team Leader
(4) Driver

MEMA kwa Vijana Trial Further Survey (2007-2008)

Survey Team
(Observations, Issues arising, immediate solutions, Proposed Solutions)

(1) Preparation and Logistics
(2) Registration
(3) Survey Interviewers
(4) Laboratory
(5) Clinician
(6) Counsellors
(7) Tracers
(8) Drivers
(9) Team Leader

MEMA kwa Vijana Trial Further Survey (2007-2008)

General Observations:

Procedures/ documents to be revised:

Outstanding Issues:

MEMA kwa Vijana Trial Further Survey (2007-2008)

MKV1FS CI SUPERVISION FORM

1. **DATE**…………………………..
2. **TIME: FROM**………….. **TO**……
3. **COMMUNITY**…………………………….
4. **VILLAGE**……………………………………
5. **KITONGOJI**…………………………………

NUMBER OF HH SUPERVISED	INTERVIEWED	ABSENT	SHIFTED	ADDITIONAL

A. CI appropriately dressed.	
B. Interaction with CH	
C. Introduction at HH	
D. Informed consent procedures.	
E. Interviewing skills	
F. Interviewed all HH Members.	
G. Ability to answer questions and explain MKV1 FS	
H. Areas to improve in	

SUPERVISOR STAFF CODE………….. **SIGNATURE**……………………………

CI STAFF CODE ……… **SIGNATURE**……………………………**TEAM**………..

MEMA kwa Vijana Trial Further Survey (2007-2008)

CENSUS SUPERVISION REPORT FORM

WARD / / /

VILLAGE / / /

KITONGOJI…………………………….

S/N	Name of Household Head	CI REPORT				SUPERVISION REPORT	COMMENTS
		Interviewed	Absent	Shifted	Refused		
1.							
2.							
3.							
4.							
5.							
6.							
7.							
8.							
9.							
10.							

SUPERVISOR STAFF CODE…………… **SIGNATURE**……………………….

MKV1 FS-CI-Superv-Reportform (03Jan08)

MEMA kwa Vijana Trial Further Survey (2007-2008)

MkV1FS: Laboratory Submission Form

Submitted by:			
Clinic staff code	|__||__||__|	Village (name)	|______________________________|
Received by:			
Laboratory staff initials	|__||__|	Date dd/mm/yyyy	|__||__| / |__||__| / |__||__||__||__|

S/N	Lab Sticker Number	3 x SERUM aliquots submitted	URINE aliquots submitted	Comments
				**2 or Less SERUM** aliquots submitted
		|__|	|__|	|__|
		|__|	|__|	|__|
		|__|	|__|	|__|
		|__|	|__|	|__|
		|__|	|__|	|__|
		|__|	|__|	|__|
		|__|	|__|	|__|
		|__|	|__|	|__|
		|__|	|__|	|__|
		|__|	|__|	|__|
		|__|	|__|	|__|
		|__|	|__|	|__|
		|__|	|__|	|__|
		|__|	|__|	|__|
		|__|	|__|	|__|

Additional Comments:

MEMA kwa Vijana Trial Further Survey (2007-2008)

Data Submission Form

	By person who submitted data	By data section
Date of submission	|__||__|/ |__||__|/ |__||__|	|__||__|/ |__||__|/ |__||__|
Staff code of person submitting/receiving the forms	|__||__||__|	|__||__||__|
Certified correct by (Staff code)	------	|__||__||__|
Village code (s)	|__||__||__| |__||__||__| |__||__||__|	|__||__||__| |__||__||__| |__||__||__|
Type of Form	No.	No.
Moved Away Form	|__||__||__|	|__||__||__|
Main Questionnaire	|__||__||__|	|__||__||__|
VCT results form	|__||__||__|	|__||__||__|
	|__||__||__|	|__||__||__|
	|__||__||__|	|__||__||__|
	|__||__||__|	|__||__||__|
	|__||__||__|	|__||__||__|
	|__||__||__|	|__||__||__|
	|__||__||__|	|__||__||__|
	|__||__||__|	|__||__||__|
	|__||__||__|	|__||__||__|

Comments by person submitting the forms
Signature of person submitting the forms
Comments by data section
Signature of person receiving the forms

MEMA kwa Vijana Trial Further Survey (2007-2008)

TREATMENT AFTER RESULTS
OF LABORATORY TESTS

Study ID: FS

Date seen / /200 Male: Female:

Clinician's code

Community code

Section 1	PREGNANCY – FEMALES ONLY	
Is this participant visibly pregnant?	Yes 1 → Go to section 2<br>No 2	
When was your last menstrual period?<br>(99 / 99 / 2009 if unknown)	/ /200	
IF MORE THAN 1 MONTH SINCE LMP THEN TEST FOR PREGNANCY		
Result of Pregnancy Test (Circle one only)		
Positive 1 Negative 2 Refused 3 Not Done / Not applicable 8		

Section 2	TREATMENT GIVEN – MALES AND FEMALES	*tick if given*
Syphilis	Benz. Penicillin 2.4 MU, im stat ½ in each buttock	*1* ✓
Syphilis if allergic to penicillin	DO NOT TREAT IN FIELD – REFERRED TO CLINIC	*2* ✓
Gonorrhoea (1st line treatment)	Ciprofloxacin 500 mg, stat	*3* ✓
Gonorrhoea (2nd line treatment or pregnant)	Ceftriaxone 250 mg im, stat	*4* ✓
Chlamydia (1st line treatment)	Doxycycline 100 mg, bid for 7 days	*5* ✓
Chlamydia (2nd line treatment or pregnant)	Erythromycin 500mg qds for 7 days	*6* ✓
Other	Specify name of drug, number of tablets and dose<br>____	*7* ✓
REFUSED TREATMENT		*8* ✓

Section 3	CONTACT SLIPS – MALES AND FEMALES
	Number of contact slips given

MEMA kwa Vijana Trial Further Survey, NIMR, PO Box 11936, Mwanza

CONTACT REFERRAL SLIP

Clinician's Name:…………………………………………………………………………

Date of diagnosis: Date|__||__|/ Mth|__||__|/ Yr 20|__||__|

Index Sticker No.: |__||__||__||__||__||__||__||__||__|

Ndugu;
Inawezekana unalo tatizo la afya, lakini hujajisikia vibaya.
Tafadhali, nenda na karatasi hii kwenye kituo cha afya kilicho karibu na wewe.
Wafanyakazi wa kituo watakuona, pia watatoa matibabu.

Message to the Clinician at the referral health unit: Please treat this person. (S)he is a contact of someone I treated for the condition (s) ***(circle condition(s))***:

Chlamydia **Gonorrhoea** **Syphilis**

Other condition (specify): ____

…………………………………………………………………………………………………

MEMA kwa Vijana Trial Further Survey, NIMR, PO Box 11936, Mwanza

CONTACT REFERRAL SLIP

Clinician's Name:…………………………………………………………………………

Date of diagnosis: Date|__||__|/ Mth|__||__|/ Yr 20|__||__|

Index Sticker No.: |__||__||__||__||__||__||__||__||__|

Ndugu;
Inawezekana unalo tatizo la afya, lakini hujajisikia vibaya.
Tafadhali, nenda na karatasi hii kwenye kituo cha afya kilicho karibu na wewe.
Wafanyakazi wa kituo watakuona, pia watatoa matibabu.

Message to the Clinician at the referral health unit: Please treat this person. (S)he is a contact of someone I treated for the condition (s) ***(circle condition(s))***:

Chlamydia **Gonorrhoea** **Syphilis**

Other condition (specify): ____

MEMA KWA VIJANA 1 FURTHER SURVEY
PAYMENT VOUCHER

Date:

Purpose of Payment:

No	Name of payee	Designation	Rate per day	No. of days	Fare	Total	Date	Signature
	**Total**							

MkV1 FS requirements for NIMR Laboratory Testing- 18 Apr 2007

Sample	How often	Agent	Test	Total no. tests in approx. 12 mths**	Turn around Time
**Serum** *(3/ participant: 1 for HIV, HSV2 and syphilis and 2 for storage)*	720-1440* serum samples /wk	HIV	Murex	~16,000	3 weeks lab, 2 week data
			Vironostika	~16,000	
			p24	~800 (5%) (discordants only)	
			Western Blot	~400 (p24 negatives or indeterminant only)	
		Syphilis	TPPA	~16,000	3 weeks lab, 2 week data
			RPR	~3,200 (+ only)	
		HSV2	Kalon	~16,000	3 weeks lab, 2 week data
**Urine** *(2/ participant: 1 for CT/NG, 1 for storage)*	480- 960* urine samples /wk	CT/NG	PCR	~16,000	3 weeks lab, 2 week data

* Assuming 240 x 3= 720 serum samples and 240 x 2= 480 urine samples collected/ team/week and samples collected from field once a week. When 2 teams are in field at the same time then 1440 serum samples and 960 urine samples collected each week but if only 1 team then 720 serum samples and 480 urine samples collected each week.

**We expect the first samples to arrive in NIMR during the week of the 28th May and for samples to arrive weekly for a period of 10-12 months.

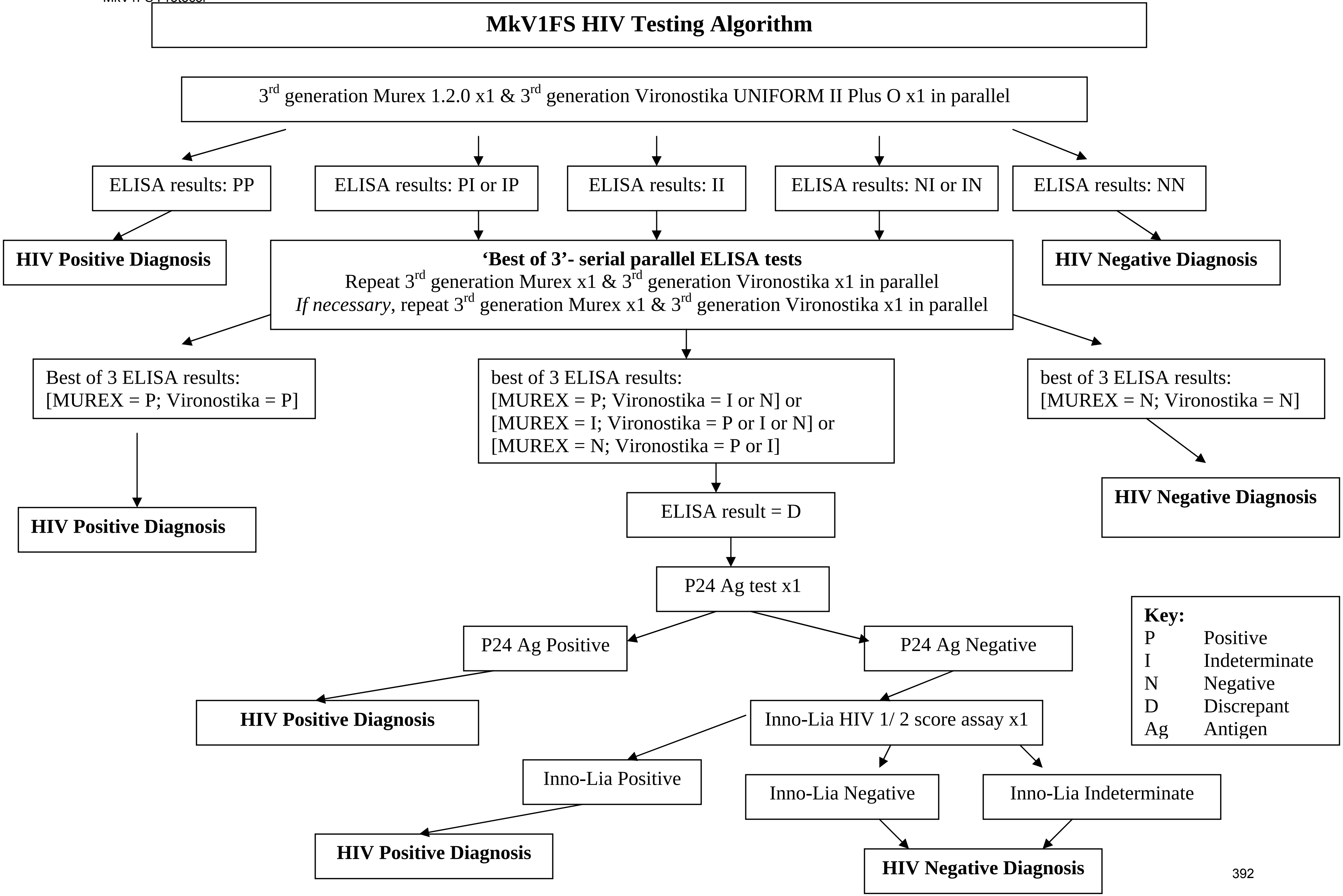
MkV1FS HIV Testing Algorithm
3rd generation Murex 1.2.0 x1 & 3rd generation Vironostika UNIFORM II Plus O x1 in parallel
ELISA results: PP
ELISA results: PI or IP
ELISA results: II
ELISA results: NI or IN
ELISA results: NN
HIV Positive Diagnosis
'Best of 3'- serial parallel ELISA tests
Repeat 3rd generation Murex x1 & 3rd generation Vironostika x1 in parallel
If necessary, repeat 3rd generation Murex x1 & 3rd generation Vironostika x1 in parallel
HIV Negative Diagnosis
Best of 3 ELISA results:
[MUREX = P; Vironostika = P]
best of 3 ELISA results:
[MUREX = P; Vironostika = I or N] or
[MUREX = I; Vironostika = P or I or N] or
[MUREX = N; Vironostika = P or I]
best of 3 ELISA results:
[MUREX = N; Vironostika = N]
HIV Positive Diagnosis
ELISA result = D
HIV Negative Diagnosis
P24 Ag test x1
P24 Ag Positive
P24 Ag Negative
HIV Positive Diagnosis
Inno-Lia HIV 1/ 2 score assay x1
Inno-Lia Positive
Inno-Lia Negative
Inno-Lia Indeterminate
HIV Positive Diagnosis
HIV Negative Diagnosis
Key:
P Positive
I Indeterminate
N Negative
D Discrepant
Ag Antigen

Annex 1: NIMR/AMREF/LSHTM Projects: PEP Report Form

This form is STRICTLY CONFIDENTIAL and should only be seen by the clinician(s) responsible for the persons involved in the incident (usually only the PEP Supervisor). The form should be anonymised by covering the names in Section A: Q2.1 and Q3.1 and photocopying the form, before the photocopied version can be shown to anyone else.

Section A. To be completed immediately

1. Basic information

1.1	Clinician's Staff code (Initials of their name)	|__||__|
1.2	Date of consultation	Date: |__||__| Mth:|__||__| Yr: 20 |__||__|
1.3	Time of consultation Swahili time	|__||__|.|__||__| Asubuhi Mchana Jioni
1.4	Place:	

2. Exposed Person

2.1	Name of Exposed Person	
2.2	Anonymous Code assigned to exposed person (eg. 01E)	|__||__| E
2.3	Sex 1=Male; 2=Female	|__|
2.4	Pregnant? 1=Definitely yes; 2=Possibly yes; 3=Probably no; 8=NA (Male)	|__|
2.5	Date of incident	Date: |__||__| Mth|__||__| Yr 20 |__||__|
2.6	Time of incident Swahili time	|__||__|.|__||__| Asubuhi Mchana Jioni
2.7	Interval between time of incident and time of consultation	Days|__||__| Hours|__||__| Mins|__||__|
2.8	Nature of exposure: 1=Needlestick ; 2=Splash; 3=Other (specify)	|__||__|
2.9	Description of exposure:	
2.10	Did you consult anyone else before making the assessment decision? 1=Yes; 2=No	|__|
2.11	Staff codes of others consulted: 88=NA	|__||__| : |__||__|
2.12	Assessment: 1=Prophylaxis needed; 2=Prophylaxis not needed	|__|
2.13	Initial prophylaxis accepted? 1=Yes; 2=No	|__|
2.14	First dose of Combivir (Zidovudine & Lamivudine) given? 1=Yes; 2=No	|__|
2.15	First dose of Nelfinavir given? 1=Yes; 2=No	|__|
2.16	If no, other drug given (name) …………………………………………………. 1=Yes; 2=No	|__|
2.17	Pre-test HIV counselling given? 1=Yes; 2=No	|__|
2.18	HIV test accepted? 1=Yes; 2=No<br>*If no, terminate the PEP procedures & enter 8s (NA) for the rest of this form*<br>*If yes, complete a Request for HIV Test Result Form*	|__|
2.19	Request for HIV Test Result Form completed? 1=Yes; 2=No	|__|
2.20	Full Blood Count Results:	
2.21	U&E Results:	
2.22	Liver Function Test Results:	
2.23	Number of additional doses of PEP given:	|__||__|
2.24	Referred to PEP Supervisor? 1=Yes; 8=NA, I am a PEP Supervisor	|__|

Ask them to come to see the PEP Supervisor again at 2 weeks post -exposure

Annex 1: NIMR/AMREF/LSHTM Projects: PEP Report Form

Section A (cont..d)

3. Source Person

3.1	Names & Sticker Number of Source Person	|__||__||__||__||__||__||__||__|
3.2	Anonymous Code assigned to source person (eg. 01S)<br>*Number should be the same as for the exposed person*	|__||__| S
3.3	Pre-test HIV counselling given? 1=Yes; 2=No	|__|
3.4	HIV test accepted? 1=Yes; 2=No<br>*If No, terminate the HIV test procedures <u>for the Source Person only</u>.*<br>*If Yes, complete a Request for HIV Test Result Form, an HIV VCT Linking Form, and a VT Slip for the Source Person*	|__|

Section B. To be completed by the PEP Supervisor

4. HIV Test Results

4.1	Clinician's Staff code	|__||__|
4.2	Date	Date: |__||__| Mth|__||__| Yr 20 |__||__|
4.3	Source Person's HIV Test Result: 1=Positive; 2=Negative; 8=NA (Test was not accepted)	|__|
4.4	Clinician's Staff code	|__||__|
4.5	Date	Date: |__||__| Mth|__||__| Yr 20 |__||__|
4.6	Exposed Person's HIV Test Result: 1=Positive; 2=Negative; 8=NA (Test was not accepted)	|__|
4.7	PEP continued? 1=Yes; 2=No	|__|
4.8	If no, Give Reason:	

5. Two Week Check-up

5.1	Clinician's Staff code	|__||__|
5.2	Date	Date: |__||__| Mth|__||__| Yr 20 |__||__|
5.3	Exposed Person Attended? 1=Yes; 2=No	|__|
5.4	Clinical details:	
5.5	Full Blood Count Results:	
5.6	U&E Results:	
5.7	Liver Function Test Results:	
5.8	PEP continued? 1=Yes; 2=No	|__|
5.9	If No, Give Reason:	

Ask them to come to see you again at 4 weeks post -exposure

Annex 1: NIMR/AMREF/LSHTM Projects: PEP Report Form

Section B (cont..d)

6. Four Week Check-up & HIV Test

7.1	Clinician's Staff code		|__||__|
7.2	Date	Date: |__||__| Mth|__||__| Yr 20 |__||__|	
7.3	Exposed Person Attended?	1=Yes; 2=No	|__|
7.4	Clinical details:		
7.5	Pre-test HIV counselling given?	1=Yes; 2=No	|__|
7.6	HIV test accepted?<br>*If no, ask them to come to see you again at 3 months*<br>*If yes, complete a Request for HIV Test Result Form & arrange a date for them to come for their HIV test result*	1=Yes; 2=No	|__|
7.7	Request for HIV Test Result Form completed?	1=Yes; 2=No	|__|

7. Four week HIV Test Post-Test Counselling

8.1	Clinician's Staff code		|__||__|
8.2	Date	Date: |__||__| Mth|__||__| Yr 20 |__||__|	
8.3	Exposed Person Attended?	1=Yes; 2=No	|__|
8.4	HIV Test Result:	1=Positive; 2=Negative; 8=NA (Test was not accepted)	|__|
8.5	Post-test counselling given:	1=Yes; 2=No; 8=NA (Test was not accepted)	|__|
8.6	Result given:	1=Yes; 2=No; 8=NA (Test was not accepted)	|__|
8.7	If No, Give Reason:		

If HIV-negative or decline testing or result, ask them to come to see you again at 3 months post –exposure
If HIV-positive, this is the end of the PEP procedures, but you should offer further post-test counselling

8. Three Months Check-up & HIV Test

8.1	Clinician's Staff code		|__||__|
8.2	Date	Date: |__||__| Mth|__||__| Yr 20 |__||__|	
8.3	Exposed Person Attended?	1=Yes; 2=No	|__|
8.4	Clinical details:		
8.5	Pre-test HIV counselling given?	1=Yes; 2=No	|__|
8.6	HIV test accepted?<br>*If no, ask them to come to see you again at 3 months*<br>*If yes, complete a Request for HIV Test Result Form & arrange a date for them to come for their HIV test result*	1=Yes; 2=No	|__|
8.7	Request for HIV Test Result Form completed?	1=Yes; 2=No	|__|

9. Post-Three Month HIV Test Post-Test Counselling

9.1	Clinician's Staff code		|__||__|
9.2	Date	Date: |__||__| Mth|__||__| Yr 20 |__||__|	
9.3	Exposed Person Attended?	1=Yes; 2=No	|__|
9.4	HIV Test Result:	1=Positive; 2=Negative; 8=NA (Test was not accepted)	|__|
9.5	Post-test counselling given:	1=Yes; 2=No; 8=NA (Test was not accepted)	|__|
9.6	Result given:	1=Yes; 2=No; 8=NA (Test was not accepted)	|__|
9.7	If No, Give Reason:		

Annex 1: NIMR/AMREF/LSHTM Projects: PEP Report Form

If HIV-negative or decline testing or result, ask them to come to see you again at 3 months post –exposure
If HIV-positive, this is the end of the PEP procedures, but you should offer further post-test counselling

10. Six Months Check-up & HIV Test

10.1	Clinician's Staff code		|__||__|
10.2	Date	Date: |__||__| Mth|__||__| Yr 20 |__||__|	
10.3	Exposed Person Attended?	1=Yes; 2=No	|__|
10.4	Clinical details:		
10.5	Pre-test HIV counselling given?	1=Yes; 2=No	|__|
10.6	HIV test accepted?<br>*If no, this is the end of the PEP Procedures*<br>*If yes, complete a Request for HIV Test Result Form & arrange a date for them to come for their HIV test result*	1=Yes; 2=No	|__|
10.7	Request for HIV Test Result Form completed?	1=Yes; 2=No	|__|

11. Post-Six Month HIV Test Post-Test Counselling

11.1	Clinician's Staff code		|__||__|
11.2	Date	Date: |__||__| Mth|__||__| Yr 20 |__||__|	
11.3	Exposed Person Attended?	1=Yes; 2=No	|__|
11.4	HIV Test Result:	1=Positive; 2=Negative; 8=NA (Test was not accepted)	|__|
11.5	Post-test counselling given:	1=Yes; 2=No; 8=NA (Test was not accepted)	|__|
11.6	Result given:	1=Yes; 2=No; 8=NA (Test was not accepted)	|__|
11.7	If No, Give Reason:		

If HIV-negative or decline testing or result, this is the end of the PEP procedures
If HIV-positive, this is the end of the PEP procedures, but you should offer further post-test counselling

NIMR/AMREF/LSHTM Collaborative Research Projects / Mwanza Interventions Trials Unit

Policy on Post-Exposure Prophylaxis for Needlestick Injuries, and Other Exposures to Potentially Infectious Materials

25 June 2007

1. **Background:**

The risk of someone being HIV-infected after a needlestick injury from an HIV-infected source person has been estimated at 3 per 1,000. The risk after exposure to splashes or contact with other tissues is lower than this. With post-exposure prophylaxis, this risk can be reduced by 50-95%.

In most of our current work (e.g. surveys of bar workers), the risk that a source person is HIV-infected is approximately 40-50%. The risk of HIV infection in other groups that we work with (adolescents, ANC attenders, community members) lies between <1-30%.

These guidelines are based on the references cited at the end of this policy.

2. **Eligibility:**

Post-exposure prophylaxis (PEP) will only be offered to staff who are exposed while carrying out duties that are directly related to the work they have been assigned by one of the NIMR/AMREF/LSHTM Collaborative Research Projects. PEP will NOT be offered to staff for sexual exposures, with the exception of rape while in the field, or exposures while carrying out private duties or duties that have been assigned by another institution.

3. **Prevention:**

All staff working for the NIMR/AMREF/LSHTM Collaborative Research Projects who handle potentially infectious materials (especially human blood) should receive a copy of this policy document. All such staff should receive guidance on how to avoid needlestick injuries and contacts with body fluids or other body tissues, and the importance of receiving immediate first aid if they do have such an injury/contact. This is being incorporated into the training seminars for the collaborative project staff on good clinical and laboratory practice.

4. **Reporting for assessment and advice:**

All staff should report incidents in which they have been exposed to needlestick injuries or a contact with body fluids or other body tissues **immediately** to one of the following PEP supervisors:

- **Dr Saidi Kapiga, MITU Director, NIMR Mwanza Centre**
- **Joseph Masanja, Acting Mwamko Project Manager.**

If neither of the PEP Supervisors is available, one of the other medically trained officers working for the NIMR/AMREF/LSHTM or MITU Projects should be contacted (e.g. currently **Mary Rusizoka, HSV**).

If the incident happens when working outside Mwanza City, this should be done through their Team Leader, or whoever is the most senior staff member with them (see below).

5. **Assessment:**
The PEP supervisor should start to complete a PEP Report Form (Annex 1:Section A). They should assess the risk of exposure to infection and the need for prophylaxis using the following guidelines:

- **Needlestick injury:** Recommend PEP in all genuine cases.
- **Mucosal contact** (e.g. mouth or eyes): Offer PEP if there was contact with blood or constituents of blood (e.g. serum/plasma), or was with untreated tissue (e.g. fresh vaginal swab material/semen). Do not offer PEP if the contact was with other body fluids (e.g. urine).
- **Skin contact:** Only offer PEP if there is an obvious portal of entry (e.g. a wound or ulcer) on the skin of the exposed person, or there was extreme contact with the blood or other untreated tissue (e.g. a major splash with blood). Do not offer PEP if there is no obvious portal of entry and the exposure was brief.
- **Rape:** If a staff member is raped while on field assignment for the collaborative projects, and there has been penetrative vaginal and/or anal intercourse, then they should be offered PEP. PEP should also be offered if there was any risk of oral mucosal contact with semen.
- **Time since incident:** If less than 4 days (i.e. ≤72 hours) have elapsed since the exposure incident, prophylaxis should be considered. If 4 days or more (>72 hours) have elapsed since the exposure incident, prophylaxis should not be considered. However, pre and post test counselling and baseline HIV testing and HIV testing at 6 weeks, 3 and 6 months should be performed with the consent of the exposed person.

The exposed person should immediately wash wounds with soap and water; flush mucous membranes with water.

6. **Prophylaxis:**
If the results of the assessment indicate that it is needed and the exposed person accepts it, prophylaxis should be started immediately with a single dose of both zidovudine (ZDV) and lamivudine (3TC) and nelfinavir (NFV):

- **Zidovudine 250mg and Lamivudine 150mg (Combivir) P.O.**
- **Kaletra 2 tablets P.O.** (i.e.400mg lopinavir + 100mg ritonavir)

Note: Kaletra is contraindicated if someone is taking the following drugs:
Antiarrythmics – Flecanide, Propafenone, Amiodarone
Antibiotics – Rifampacin
Antihistamines – Astemizole, Terfenadine
Benzodiazapines – Midazolam, Triazolam
Ergot derivatives – Ergotamine, Ergodryal and related drugs
GI motility agents – Cisapride
Herbal Products – St John's Wort
Neuroleptics – Pimozide
Statins – Lovastatin, Simvastatin

In this case the clinician or PEP coordinator should only give the emergency dose of Combivir and should seek advice as soon as possible from a senior doctor on the team.

Immediately after giving the first emergency dose of PEP, the need for an HIV test should be discussed, and full pre-test counselling given. The subject should be reassured that the results of the test will be strictly confidential between themselves and the PEP Supervisor who will give them post-test counselling. The test results will not affect their employment. However, prophylaxis will not be continued unless the HIV test is accepted.

If the HIV test is accepted, prophylaxis should be continued with:

- **Zidovudine 250mg twice per day and Lamivudine 150mg twice per day P.O.**
- **Kaletra 2 tablets P.O. twice per day (i.e.400mg lopinavir + 100mg ritonavir b.d.) swallowed whole**

The full course of PEP is 4 weeks (but see Section 7 for the rules about continuing or stopping prophylaxis depending on the HIV test result). They should read and given the PEP Patient Information Sheet (Annex 2). The drugs should be taken with a light meal or snack to ensure adequate absorption of nelfinavir.

Side effects: diarrhoea. This can usually be managed conservatively with anti-diarrhoeals. ZDV-associated anaemia is unlikely within 4 weeks of treatment unless the exposed person already has anaemia. If this is the case, an alternative regimen will be chosen once baseline blood tests have been checked. Increased total cholesterol and GGT are noted on laboratory testing. Other possible minor side effects with Kaletra include insomnia and headache.

Rarely Kaletra can cause pancreatitis. This is more common in people with pre-existing liver disease.

Kaletra also interferes with the oral contraceptive pill and reduces its effectiveness. In any event, whether the exposed person accepts HIV-testing or not, they should be encouraged to abstain from sex or to use a condom consistently during the first six months after exposure.

PEP supplies are held by Dr Saidi Kapiga and Joseph Masanja. A 3 day PEP starter pack is also held by the MkV FS field teams, under the supervision of the clinician working in each team.

7. HIV Testing:

Blood from both the source person and the exposed person should be tested for HIV as soon as possible after the incident. The source person should receive full pre-test counselling, and has the right to refuse to be tested. The importance of maintaining confidentiality for the exposed person is paramount. The PEP Supervisor is responsible for ensuring that the serological testing is done and reported promptly, and that the confidentiality of both the exposed person and the source person is maintained. This should be done by assigning a code to each person.

The decision to continue or stop prophylaxis should be based on the following:

- **Exposed person HIV-positive:** Stop prophylaxis
- **Exposed person and source person are HIV-negative, where source person is from a low risk group (adolescents, ANC attender):** Stop prophylaxis
- **Exposed person and source person are HIV-negative but source person is from high HIV incidence risk group (e.g. barworker) where early HIV infection cannot be excluded:** Continue prophylaxis for a total of 4 weeks
- **Exposed person is HIV-negative and source person is either HIV-positive or cannot be tested:** Continue prophylaxis for a total of 4 weeks.

8. Documentation:

The PEP Supervisor should complete a PEP Report Form and file this in a locked cabinet. They should cover the names of both the exposed person and the source person with opaque paper and photocopy the form as soon as they have completed Section A. This will create an "anonymised" PEP Report Form. As soon as possible after the incident, the PEP Supervisor should copy the **anonymised** PEP Report Form to:

- The Programme Manager, AMREF Lake Zone
- The Director, NIMR Mwanza
- The MITU Director/LSHTM Team Leader, Mwanza
- The other PEP Supervisor

These people should then meet with the PEP Supervisor to discuss the incident (anonymously) and to make recommendations of any steps that should be taken to avoid such incidents in future.

The PEP Supervisor is encouraged to discuss the incident (anonymously) with the other PEP Supervisor if they think there is any difficulty deciding what to do: e.g. if the exposed person is pregnant.

9. Follow-up:

The exposed person should be encouraged to have further counselling and HIV testing, following Tanzanian guidelines, at 4 weeks, 3 months and 6 months after the incident to document any sero-conversion.

The exposed person should be seen by the PEP Supervisor and assessed clinically at 2 weeks and at 4 weeks if they are receiving PEP to assess their clinical and mental state, with particular assessment of potential drug-related side effects. Common drug-related side effects are: nausea, vomiting, diarrhoea, tiredness and headache. At baseline and at 2 weeks, blood should be taken for full blood count and for renal and liver function tests (if possible) to monitor for drug toxicity. The exposed person should be encouraged to abstain from sex or to use a condom consistently during the first six months after exposure. Condoms should be provided by the PEP supervisor. They should be informed that they should not act a blood donor for the first 6 months after exposure.

10. Staff working away from Mwanza:

Field Teams working outside Mwanza City that include a medically-trained person (Medical Officer, Asst Medical Officer, Clinical Officer, Clinical Asst) should always carry this policy document, PEP Report Forms, and 3 days supply of PEP. One clinician should act as the Field PEP clinician. They must account for all PEP drugs they have used on the PEP Report Forms, and should restock their supply of PEP

drugs as soon as possible from the PEP supervisors. They should complete Section A of the PEP Report Form.

Staff in such a team must report any incident to their Team Leader and the Field PEP clinician. If it is possible for this clinician to consult one of the PEP supervisors within 2 hours of the incident to seek advice (e.g. by phone), they should do so. If this is not feasible, the Field PEP clinician should make the assessment and recommend or offer prophylaxis if this is indicated (see above). They should open one of the PEP starter packs and immediately give the exposed person a stat dose of:

- **Zidovudine 250mg <u>and</u> Lamivudine 150mg (Combivir) P.O.**
- **Kaletra 2 tablets P.O.** (i.e.400mg lopinavir + 100mg ritonavir)

The Field PEP clinician and Team Leader should instruct the affected staff member to return to Mwanza immediately to seek further advice and treatment (if needed) from one of the PEP supervisors, and should make arrangements for this (e.g. by providing a project car). While they are making these arrangements, the exposed person should be continued on:

- **Zidovudine 250mg <u>and</u> Lamivudine 150mg twice daily P.O.**
- **Kaletra 2 tablets P.O. twice per day** (i.e.400mg lopinavir + 100mg ritonavir b.d.) **swallowed whole**

The exposed person should be sent to Mwanza with the blood specimen of the source person (if obtained), the blood specimen of the exposed person, and the PEP Report Form in a sealed envelope addressed to both of the PEP Supervisors by name (i.e. Dr Saidi Kapiga or J Masanja). The envelope should be clearly marked URGENT, and both the exposed person and the driver must be told that the envelope must reach one of the two people immediately on arrival in Mwanza (even if this is in the middle of the night). If neither of the PEP Supervisors is available, one of the other medical officers working for the NIMR/AMREF/LSHTM Projects should be contacted (e.g. currently Mary Rusizoka).

Staff members must NEVER act as their own clinician.

If the incident happens outside Mwanza City and the staff member is not part of a team with a clinician (see above), they must return to Mwanza immediately to report to one of the PEP supervisors. However, if the nearest PEP is with one of the other collaborative project teams out in the field, they should go to the team as soon as possible to collect the first dose of PEP before travelling onto Mwanza.

11. Other regimens:

In the event that the PEP packs provided by LSHTM have been used and it is necessary to purchase PEP locally, alternative 28 day regimens are either:

- **Zidovudine 250mg <u>and</u> Lamivudine 150mg twice daily P.O.**
- **Indinavir (IDV) 800mg three times daily P.O.**

OR

- **Zidovudine 250mg <u>and</u> Lamivudine 150mg twice daily P.O.**
- **Efavirenz (EFV) 600mg once daily P.O.**

Although IDV is recommended as the first-line protease inhibitor for PEP in Tanzania, poor tolerability has been reported.

EFV is currently listed as the first-line protease inhibitor in Tanzania for the management of HIV-infection. The most common side effects with this regimen are EFV-associated CNS effects. For this reason EFV should be taken at night before going to sleep. EFV should NOT be given in 1st trimester of pregnancy because of potential teratogenic side effects.

12. **PEP in pregnancy:**
The regimens for exposed pregnant staff members in Mwanza should be either a 28 day course of:

- **Zidovudine 250mg <u>and</u> Lamivudine 150mg twice daily P.O.**
- **Kaletra 2 tablets P.O.** (i.e.400mg lopinavir + 100mg ritonavir)

Or:

- **Zidovudine 250mg <u>and</u> Lamivudine 150mg twice daily P.O.**
- **Nevirapine (NVP) 200mg once daily for 14 days, then 200mg twice daily for 14 days P.O.**

The most common side effect with this regimen is NVP-associated rash. If mild/moderate continue tablets. If severe, stop NVP. **This will need to be purchased as the PEP coordinators do not stock NVP.**

13. Summary:

Immediately:

- Assess exposure and administer first aid
- Ask what other drugs the exposed person is taking
- Give first dose of PEP
- Encourage counselling and HIV testing (unless the exposed person agrees to testing, prophylaxis cannot be continued)
- Take blood from both the source person and the exposed person for HIV testing
- If outside Mwanza City: Send the exposed person with their own blood specimen and that of the source person (in vacutainers) and the PEP Incident Form to one of the PEP Supervisors in Mwanza.
- Ensure the HIV testing is done as quickly as possible.

Baseline:

- HIV ELISA x 2, Clinical assessment and blood for full blood count, urea & electrolytes, and liver function tests

After 2 weeks:

- Clinical assessment and blood for full blood count, urea & electrolytes, and liver function tests

After 4 weeks:

- Clinical assessment and stop PEP
- Counselling and HIV testing

After 3 months:

- Counselling and HIV testing

After 6 months:

- Counselling and HIV testing

14. References

CDC 2005, MMWR 54 1-20

HIV Post-exposure Prophylaxis: Guidance from the UK Chief Medical Officers' Expert Advisory Group on AIDS. Department of Health, Feb 2004.

National Guidelines for Clinical Management of HIV/AIDS. United Rebublic of Tanzania, Ministry of Health. April 2002.

Scaling up antiretroviral therapy in resource-limited settings. Guidelines for a public health approach. World Health Organization, April 2002.

The 2002 Abbreviated Guide to Medical Management of HIV Infection. J.G.Bartlett. Johns Hopkins School of Medicine, Division of Infectious Diseases, Baltimore. 2002.

NIMR/AMREF/LSHTM Collaborative Research Projects
Mwanza, Tanzania

Annex 2: Patient's Information Sheet

You have sustained an injury that may have exposed you to HIV infection. We are giving you medicines that will help to reduce your chances of getting HIV infection by more than 80%. It is important that you read and understand the following information.

The full course of treatment is 4 weeks. The treatment may need to be taken 2 times a day. The doctor will explain when you should take the tablets and whether you should take them with food. ***IT IS ESSENTIAL THAT YOU COMPLETE THE FULL 4 WEEK COURSE OF TREATMENT*** unless we ask you to stop taking the tablets.

Please tell the doctor if you are taking any other medicines. Some of these may not work when you are taking the medicine we give you, so it is important that we know what drugs you are taking. Do not stop them unless we tell you to. You should not take any other medicines unless you discuss them with the doctor first so that we can check that they do not interfere with the medicine we are giving you.

If you have any problems while taking the medicine we give you, please come back and see the doctor. These problems may include diarrhoea, rashes, vomiting, abdominal pain or tiredness.

IMPORTANT

You must use condoms or abstain from sexual intercourse until we have checked you for HIV infection after you have finished the medicines. This is because:

- You may be infectious to your partner
- You may catch HIV from a partner
- You may have been given medicine that may harm a baby if you get pregnant
- You may have been given a medicine can interfere with the oral contraceptive pill so that it no longer works to prevent pregnancy. The oral contraceptive pill will work again once you have finished the medicine we have given you. The doctor will advise you about this.

If you have any questions or concerns, please ask us about them now.

If you have any problems on the medication, you should contact one of the doctors listed below, either in AMREF or the following telephone numbers:

Dr Deborah Watson-Jones: 0754 056066
Joseph Masanja (clinical officer): 0754 824388

If neither available, please contact Mary Rusizoka or Abel Ngwalle via AMREF (028-2500220).

Annex 3

NIMR/AMREF/LSHTM Collaborative Projects

HIV TEST RESULT FORM

Please give patient the following information about the voluntary HIV testing service.
As you were told earlier, all the information and the results of the test on blood you have given us will be kept secret. If you decide you would like to know your HIV result, I will take a sample of blood from you. This sample will be taken back to NIMR, Mwanza, where it will be tested for HIV. Your name will not be on this sample. No one except you and myself will whose result this is. I will you an appointment to come back to collect the result. At that time I will also give you further advice and information.

1	**Requests Voluntary HIV test**	1=Yes; 2=No	|__|	**VTReq**
2	**Have you given the pre-test counselling?**	1=Yes; 2=No	|__|	**VTCouns**
3	**Have you taken and labelled the blood?**	1=Yes; 2=No	|__|	**VTBlood**
3	**VT Number**		|_V|_T||__||__|__|	*VTNo*
4	**Date for participant to return for results**		|__|__|/|__||__|/|__||__|	*vctvround*
5	**Signature of person requesting VCT** ………………………………………………………			
6	**Signature of person providing pretest counselling** ……………………………………			

NATIONAL INSTITUTE FOR MEDICAL RESEARCH
MWANZA MEDICAL RESEARCH CENTRE

Telephone:028-2500399/2503012/40723
Direct:028-2500189 Fax: 028-2500654
Telegrams: MEDSEARCH
E-mail::mwanza@nimr.co.tz

P.O. Box 1462
Mwanza
Tanzania.

Date:

To whom it may concern

Re: Agreement on conditions of use of MkV1 FS project vehicles

I have read the conditions of MkV1 FS project vehicle use specified in my standard operating procedures and I accept the conditions described therein. In particular, I accept that if I use the vehicle for non-official duties without the written agreement of the Project Co-ordinator then the following action will be taken:

1. On the first occasion, I will be given an official written warning and will be fined Tsh 50,000.
2. On the second occasion, I will be fined TSh50, 000 and dismissed.
3. The fines will be deducted from my monthly salary payments from the project.

Yours truly,

Name:

Position:

MEMA kwa Vijana Further Survey (MkV FS)

ANALYTICAL PLAN

This document should be read in conjunction with the MkV1 FS annotated CRF (English version of SukSwa30may07)

1. Summary of trial design and study population

1.1 Trial design

The trial is a community randomised controlled trial of the MEMA kwa Vijana (MkV) adolescent sexual and reproductive health intervention (ASRH). In this trial, 20 rural communities were grouped into three strata that were expected to have a high risk (6 communities), medium risk (8 communities) or low risk (6 communities) of HIV and other STIs based on an initial community-based survey. Half the communities in each stratum were randomly selected to receive the intervention, the others acting as control communities.

1.2 Hypothesis

In the longer-term, the MEMA kwa Vijana (MkV1) Intervention leads to an improvement in sexual and reproductive health.

1.3 Study population

The study population consists of consenting young adults who are *de jure* members of a household in one of the 20 trial communities in rural Mwanza AND who attended at least one year of standards 5, 6 or 7 in one of the trial primary schools between 1999 and 2002.

1.4 Exposure variable

Eligibility depends on attending an MkV study school (either Intervention or Comparison) in Standards 5-7 for at least one year during the period 1999-2002. Study arm will be assigned according to the first such school attended in Standards 5-7 during that period (Exposure 1 in Table 1.1).

A sub-analysis (Exposure 2 in Table 1.1) will look at impact of the intervention on those who were interviewed in 2007/08 in their original community ie their community for exposure 1.

Table 1.1 Exposure variables

Indicator	Variable	Comment
Exposure 1<br>***(MAIN ANALYSIS)***<br>Intervention vs Comparison community at start of intervention	Q0113c1, Q0113c2<br>Q0113d1, Q0113d2<br>Q0113e1, Q0113e2<br>Q0113f1, q0113f2<br><br>*inconsistency check with q0112*	➢ YP will be classified according to the community of the primary school where they first attended std 5, 6 or 7 from 1999 onwards eg if YP attended std 5 in school B in 2002 then school B's community will be the community for trial analysis.
Exposure 2<br>***(SUB- ANALYSIS)***<br>Intervention vs Comparison community at start of intervention & interviewed in the same community in 2007/08	Q0113c1, Q0113c2<br>Q0113d1, Q0113d2<br>Q0113e1, Q0113e2<br>Q0113f1, q0113f2<br><br>& Q0102	➢ We will carry out a sub-analysis of those who had MkV1 FS interview in their 'original' community ie community where they first went to primary school in the correct years (see exposure 1).<br>➢ Those who attended more than one school in the same community or trial arm will be included and classified by the first school attended.<br>➢ 'Crossovers' ie those who had years of schooling in both trial arms will be excluded.

1.5 Trial outcomes

The impact of the intervention on predefined primary and secondary outcomes will be examined (Tables 1.2- 1.5). The trial is powered to look at primary outcomes individually for each sex but is also likely to have adequate power to look at secondary outcomes individually for each sex.

Table 1.2 Primary Outcomes

	Indicator	Variable
1.1	HIV prevalence	hiv_fin<br><br>WB indeterminate= Negative
1.2	HSV2 prevalence	hsv_res<br><br>HSV2 indeterminate= Negative

Table 1.3 Secondary Outcomes- STI Prevalence

	Indicator	Variable
2.1	Syphilis- TPPA+, RPR- (lifetime) prevalence	tpp_res="P"
2.2	Syphilis- TPPA+, RPR+ (active) prevalence	tpp_res="P"<br>rpr_res="P"
2.3	CT prevalence	ct_res= "P"
2.4	NG prevalence	ng_res="P"<br>& confirmed by PCR<br>*(if PCR results not available then provisional results will be based on OD >= 2.0)*

Table 1.4 Secondary Outcomes- ASRH Knowledge & Attitudes

	**Indicator**	**Variable**
2.5	Knowledge on HIV acquisition	% scoring 3 ie all questions correct.<br><br>q0304=1 scores 1; q0304=2 scores 0; q0304=9 scores 0<br>q0305=1 scores 0 ; q0305=2 scores 1 ; q0305=9 scores 0<br>q0306=1 scores 1 ; q0306=2 scores 0 ; q0306=9 scores 0
2.6	Knowledge on STD acquisition	% scoring 3 ie all questions correct.<br><br>q0301=1 scores 1 ; q0301=2 scores 0 ; q0301=9 scores 0<br>q0302=1 scores 0 ; q0302=2 scores 1 ; q0302=9 scores 0<br>q0303=1 scores 1 ; q0303=2 scores 0 ; q0303=9 scores 0
2.7	Knowledge on pregnancy prevention	% scoring 3 ie all questions correct.<br><br>q0310=1 scores 1 ; q0310=2 scores 0 ; q0310=9 scores 0<br>q0311=1 scores 1 ; q0311=2 scores 0 ; q0311=9 scores 0<br>q0312=1 scores 1 ; q0312=2 scores 0 ; q0312=9 scores 0
2.8	Sexual attitudes	% scoring 3 ie all questions correct.<br><br>q0307=1 scores 1 ; q0307=2 scores 0 ; q0307=9 scores 0<br>q0308=1 scores 1 ; q0308=2 scores 0 ; q0308=9 scores 0<br>q0309=1 scores 0 ; q0309=2 scores 1 ; q0309=9 scores 0

Table 1.5 Secondary Outcomes- Reported Behaviour & Reported Clinical symptoms

	Indicator	Variable
2.9	Age at first sex	q0403a (age in years)<br>q0403b (age in age group)<br><br>(i) Binary<br>1 <16 years<br>0 >= 16 years<br>*exclude those aged < 16 yrs<br><br>(ii) Using survival analysis to calculate median age
2.10	Lifetime number of sexual partners	q0601<br>q0401(=0 partners)<br><br>BINARY (0-2, 3+)
2.11	>1 partner in last 12 months	q0407<br>q0401 (=0 partners)
2.12	Used a condom at last sex in past 12 months	q0513a = 1 or =2<br><br>(OR q0513b OR q0513c)
2.13	Used a condom at last sex with non-regular partner	q0501 (3 or 4) & q0513 (1 or 2)
2.14	Ever used modern contraceptive	Q0608a1, q0608b1, q0608c1
2.15	Use of modern contraceptive at last sex	Q0512a1, q0512a2, q0512a3
2.16	Lifetime number of pregnancies	q0602<br><br>BINARY (0-1, 2+)
2.17	Concurrent partners 1<br>(more than one partner in same time period over last 12 months)	q0508 and q0509
2.18	Concurrent partners 2<br>(made love to more than one partner in the last 4 weeks)	q0510
2.19	Timing of first pregnancy (pregnant in primary school vs not pregnant in primary school)	q0603 (females)
2.20	Unplanned pregnancy	q604 (females)
2.21	Go to hospital for treatment of STI symptoms (last 12 mths)	q0803 (also q0801, q0802)
2.22	GDS symptoms	Q801
2.23	GUS symptoms	Q802

1.6 Potential Confounding Factors and Effect Modifiers

All analysis will be stratified by sex.

We will adjust for confounders only if there is a substantial imbalance between trial arms. We will only adjust for indicators that are not on the causal pathway. A-priori we will adjust for:

- Age group
- Tribe
- Stratum

Subgroup analysis will be carried out for the following indicators which are known effect modifiers:

- Age group at further survey
- Marital Status
- Level of exposure to the MkV intervention (years in trial school, std5-7, 1999-2004)
- Years since left trial primary school

Table 1.6 Other indicators

**Indicator**	**Variable**
Sex	q0105
Age group	q0109a & q0109b<br><21 yrs, 21-22 yrs, 23-24 yrs, >=25 yrs
Tribe	q0203=1 Sukuma vs others
Marital status	q0703, q0704
Clinician observed male circumcision	q1111
Religion	Christian, Muslim, Other religion, No religion
Highest level of education	q0205
Occupation	q0206a-I Business<br>q0206j-m Fishing/Farming/Domestic<br>q0206n School/University<br>q0206o-p Other/None
Total Number of years in trial school Std 5-7 (1999-2004)	q0113<br>*Inconsistency check with q0112*
Number of years since left last year in trial primary school	q0113

2. PLAN OF ANALYSIS

2.1 **Description of census and survey recruitment**

A flow chart will be drawn to show how many of the potentially eligible young people identified during the census attended and were eligible/ non-eligible and how many attended and participated by sex (see Table 2.1).

The proportion of invited young people who attended the survey will be examined by community (census dataset: ward_name), sex (census dataset: sex) and age group (census dataset: dob, yob, age) to identify any possible bias or under representation of sub-populations.

Table 2.1

**Indicator**	**Variable**
Households interviewed during the census	*No. of household records in census dataset*
Households not interviewed (ie absent and refusing)	*Households absent and refusing from census field reports (community report form)*
Household members in households included in census	*Number (by sex) of household members in census dataset* ***n_hh***
Potentially eligible young people identified and invited to survey 'Invited Young Person (YP)'	*Number of invited young people (by sex) according to census database* ***Census_number, Appointment_date***
Invited attendees at survey (eligible, ineligible) – did they bring invitation?	*Census ID no. (Q0108) is present*<br><br>*q0106=1 if brought invitation*
Non-invited attendees at survey (eligible, ineligible)	*Q0106 = 2 or 3 AND q0107= 2*
Participants (ppts)	
Eligible Participants	
No. of participants providing biological samples (blood and/or urine)	*Number of blood/urine records on lab database*
MkV1 Recruited	YP who have MkV1 ID card or who are on a list of those recruited in the MkV1 1998 baseline survey
MkV1 Cohort	YP who has a white ID card OR their MkV1 ID number can be found in the cohort dataset.<br><br>*Q0116= 1 or 2 if have card and q0116= 3 if on list.*
No. opting for VCT	*VCT results*
No. diagnosed, treated and referred for STI during main survey and during follow-up STI treatment	*Q1112 and follow-up STI treatment forms*

2.2 Description of cross-sectional sample

This analysis will involve all those who fulfil the eligibility criteria and who participate in the survey ie Elig ppt

- The following socio-demographic variables will be tabulated by sex and Arm of trial (Intervention, Comparison): Age, Marital Status, Religion, Tribe, Highest level of Education.

- Any of these variables for which there is a substantial imbalance between arm will be noted so that final analysis can take this into account. This assessment will *not* be based on the results of significance tests, and p-values will not be shown.

- The following socio-demographic variables will be tabulated by sex and Arm of trial (Intervention, Comparison) but will not be adjusted for in analyses: Occupation, Male circumcision.

2.3 Statistical methods

- Analysis of this stratified cluster randomised trial will follow a two-stage approach:
  - In the first phase a summary measure is obtained for each cluster.
  - At the second stage the two sets of cluster-specific measures are compared using a stratified t-test with 14 degrees of freedom.
- The number of individuals excluded may differ for each analysis eg result for the test/question not available for that individual
- If a similar effect of the intervention is seen in males and females then the impact on both sexes will be examined. This will be done for primary outcomes only.

2.3.1 Unadjusted analysis

Within each sex, the overall prevalence for each community will be calculated and presented according to trial strata and arm. Risk ratios and 95% CIs will be calculated using the method described by Hayes & Moulton for stratified cluster randomised trials. For continuous outcomes eg age at first sex, the overall mean/median for each community will be calculated and community means/medians will be shown by strata and arm. As we have equal numbers of communities within strata in each arm, the log risk ratio is equal to the difference between the means of the log risk in each arm. The stratified t-test will be used to carry out a significance test of the null hypothesis of no intervention effect, and to obtain a confidence interval of the parameter of interest.

2.3.2 Adjusted analysis

Covariate adjustment will be achieved through a two-stage procedure as described by Hayes & Moulton. In the first stage, the expected number of events will be computed by fitting a logistic regression model to the individual level data. This model will include terms for stratum and pre-defined adjustment factors, but not study arm. The adjusted risk ratio (RR) and 95%CI will be obtained using methods as above, but based on the community log (O/E).

For outcomes with zero cases in some communities, or a log transformation is clearly inappropriate, unadjusted and adjusted RR will be obtained as the ratios of arithmetic mean prevalence O/E, and approximate variances and CI will be obtained

from ANOVA of untransformed community prevalence O/E on stratum and study arm.

2.3.3 Sub-group analyses

Effect-modification of intervention arm with the following factors will be assessed using the method of Cheung et al (TMIH 2008 13:2:247-255):

- Age group at further survey (categorical)
- Marital status (binary)
- Estimated potential number of years of exposure to the intervention (trend)
- Estimated number of years since leaving school (trend)

For the binary variable (marital status), we carry out a t-test to compare the difference in prevalence within each community between arms. To assess effect-modification of dose-response for the other variables, we extend Cheung's method by using linear regression to estimate the dose-response for each community, and conducting a t-test of the regression coefficients between arms. These analyses will be conducted for the adjusted RR (i.e. outcome is log O/E).

Appendix 1

MkV1 DATA AVAILABILITY

The STATA data files that will be utilised in the analysis are described below. The data-sets that will not be used in the final analysis will be described in a different document.

Cohort recruitment data (1998)

Data collected during the MkV cohort recruitment:

STATA File Name	***Content of the file***
**ENROL** (N=17085)	eligibility criteria of the pupils collected in the enrol. forms
**MEAS2**	reported behaviour and biomedical data in Main Questionnaire and Lab results (N=9283).

Lab results available at cohort recruitment:

1. HIV tested on urine using GACPAT for screening, positive confirmed by GACELISA
2. Pregnancy
3. NG
4. CT

Interim survey data (2000)

Data collected during the MkV interim survey:

STATA File Name	***Content of the file***
**MAINQ_2**	Main Questionnaires (Face to Face Questionnaires)
**SYPH_I2**	Syphilis results (tested on blood spot using Serodia TPPA test)
**UPT_INT**	Pregnancy results (tested on urine using Quickstick)
**NGCT_I**	NG and CT results
**HIV_INT**	HIV test results (tested on blood spot using Serodia for screening, positives confirmed by UniformII)

HIV was also tested on urine using GACPAT for screening and GACELISA for confirmation (file name HIV). The HIV results on urine will be compared with those on blood spot but for final analysis purpose only HIV test result on blood spot will be used.

Details of each dataset will be available before starting the final analysis (variable name, value labels, description of content of each variable)

Dataset MAINQ_ 2 all errors or discrepancies have been solved on the forms but more has to be done before the final analysis.

Final survey data (Oct 2001 – April 2002)

Data collected during the MkV final survey:

Data available

STATA File Name	***Content of the file***
**MAINQF**	Main Questionnaires (Face to Face Questionnaires)
**SYPH_F**	Syphilis results (tested on blood spot using Serodia TPPA test)
**UPT_F**	Pregnancy results (tested on urine using Quickstick)
**HIV_F**	HIV test results (tested on blood spot using Serodia for screening and Uniform II for confirmation and on serum using Uniform II and Murex)
**BV**	BV results (tested on vaginal swab). QC tests not done
**HSV2_F**	HSV2 test results
**NGCT_F**	Test completed.
**TV_F**	TV test results

All data in the main qre .were entered and all discrepancies were checked on the original form and inconsistency were solved additional inconsistency were solved later during the preliminary analysis (not by arm).

Appendix 2

MkV1 FS DATA AVAILABILITY

Further survey data (Jun 2007 – August 2008)

Data available

STATA File Name	***Content of the file***
**Mkvfs_all_1**	Main questionnaires & lab results
**Quest_abc_v2**	Main questionnaire data after cleaning by data management
**Quest_abc_v1**	Main questionnaire data before datacleaning
**Census_kaya2vijana**	Census data (household and vijana merged together)

Appendix 3: Eligibility Check
(to see if those selected as eligible by registration interviewer are actually eligible)

Young person is eligible if reported information (1) is confirmed by documentation (2) and informed consent is given (3).

1. **Reported information**

{Q0113c1= trial school code (see list) AND q0113c2= 5, 6 or 7
OR
Q0113d1= trial school code (see list) AND q0113d2= 5, 6 or 7
OR
Q0113e1= trial school code (see list) AND q0113e2= 5, 6 or 7
OR
Q0113f1= trial school code (see list) AND q0113f2= 5, 6 or 7}

2. **Verification of eligibility**

{Q0116=1, 2 or 3
OR
Q0118b=1 or 2}

3. **Informed consent**

Q0120=1 (OR =2 or 3 or missing but informed consent sheet has been submitted for this YP)

Cross-checks

(i) Check questionnaires (and registration book, informed consent sheets) for anyone who was interviewed as eligible but who does not fulfil these criteria
(ii) Check questionnaires (and registration book) for anyone who was determined to be ineligible but who is eligible according to the above criteria.

MEMA kwa Vijana Trial Further Survey 2007-08

MkV1FS: Field Team extra supplies form

Team	
Location (Ward, village)	
Requested by	
Date request sent to MkV1FS Coordination office	

Item/ Document	Number needed	Number remaining	Needed by (date)	Details (reason, specifications)	Delivered (Date/ Signature)

MEMA kwa Vijana Trial Further Survey (2007-2008)

STAFF CODE OF CONDUCT

1. You should handle and keep safely the office equipment, those in your possession and the ones being possessed by other staff individually or as a team.
2. You should not threaten to hurt yourself, your fellow staff or community member.
3. You should work safely with the equipment, which might be dangerous to you and others.
4. You should keep all drugs and sharp or dangerous equipments away from people.
5. You should not fight or get involved in any fight either with the fellow staff or community member.
6. Make sure that you don't walk in the night, all staff are required to be in the Guest House by 9:00pm.
7. You should not be involved in sexual relations with the community members as this leaves the MkV1FS with bad image in the community we are working.
8. Do not leave the work site without prior information to the PC, FWM or FS
9. You are allowed to have only one alcoholic drink per day while in the field and this should be in the evening after work.

MEMA kwa Vijana Trial Further Survey (2007-2008)

MkV1FS: Progress Report

**Date(s) of report:**	
**Author(s):**	
**Section:**	

(1) Main activities since last meeting<br>*(including progress statistics eg number of samples analysed, questionnaires entered etc)*
(2) Main activities/ Priorities for next month
(3) Staff issues (changes, travel, holidays etc)
(4) Procedures/ documents to be revised
(5) Outstanding Issues

MEMA kwa Vijana Trial Further Survey (2007-2008)

MkV1 Further Survey: Calendar of Events

(To be used by field worker in assisting participants to recall day/month and specific year of a given events/phenomena)

S/no	Date/Month/Year	EVENTS
1	April 1985	Kustaafu kwa Rais J.K. Nyerere kuwa Rais
2	1990	Ziara ya baba mtakatifu **Papa John Paul wa II** kuja Tanzania
3	Dec 1995	Uchaguzi wa kwanza wa Rais na wabunge uliyo shirikisha **vyama vingi** (mfano: Mrema, Cheyo)
4	Dec. 1995	**Mkapa** kuwa Rais kwa mara ya kwanza
5	May 1996	Meli ya **MV. Bukoba** ilizama katika ziwa Victoria na kuua watu wengi.
6	Sep.1997/April 1998	Mvua kubwa na mafuliko karibu inchi nzima - **Mvua za "Elnino"**
7	June 1998	Kufanyika kwa **kombe la dunia Ufaransa** na ufaransa kuwa Bingwa**.**
8	Oct. 1999	**Kifo cha Baba wa Taifa Mwl. J. K. Nyerere**
9	June 2002	**Ajari mbaya ya Treni** iliyotokea Dodoma na kuua watu wengi
10	June 2002	Kufanyika kwa **kombe la dunia Korea** Kusini na Japan.
11	Sept 2001	Jengo la kimataifa la biashara la marekani kushambuliwa na magaidi (OSAMA)
12	Julai 2003	Kifo cha aliyekuwa **makamu wa Rais Dr Ally Juma**
13	2003	Vita kubwa kati ya Marekani na washirika wake dhidi ya Irak.
14	2003	Marekani na Uingereza kuivamia Iraq
15	June 2004	Kifo cha Papa John Paul II
16	Dec. 2005	**Kikwete** kuwa Rais kwa mara ya kwanza
17	2006	Homa ya mafua ya ndege kuitikisa dunia
18	2006	Njaa iliyojitokeza katika baadhi ya mikoa nchini na kupelekea serikali kutoa chakula cha misaada.
19	2007	Kuingia kwa homa ya Bonde la ufa nchini na kuitikisa mikoa ya Dodoma,Arusha na Singida.

MEMA kwa Vijana Trial Further Survey (2007-2008)

Template for Main Registration Book

SN	Date	Time	Census ID no								Name	Village	Kitongoji	Head of Household	MkV1 list? (Y/N)	STICKER

Note:
1. If not-eligible then write ‘NOT ELIGIBLE’ in the space for STICKER.
2. Time should be in Swahili time

Template for counsellor record book

SN	DATE	STICKER NO	HIV TEST RESULTS			COMMENT	STAFF CODE	SIGNATURE
			BIOLINE	DETERMINE	OVERALL			

Note:
1. HIV Test Results: P=Positive, N=Negative, I=Indeterminate, X= Not done

Template for clinician treatment register book

SN	DATE	STICKER NO	DIAGNOSIS	TREATMENT	NO. OF CONTACT SLIP GIVEN	COMMENT	STAFF CODE	SIGNATURE

Template for laboratory technician register

SN	DATE	STICKER NO	No. Urine Aliquots	No. Serum Aliquots	Dipstick result	Comment	STAFF CODES	Signature

variable name in access	variable type in access	variable length in access	caption (access)	Current STATA variable name *(file from CM, Nov 08)*	New STATA variable name	Description
Team				team		CT1 or CT2
RecordId	Long Integer	4		recordid		
UnitID	Long Integer	4		unitid		
UserName	Text	255		username	pda_number	number of PDA
TimeStamp	Date/Time	8				date and time (?of start of interview)
intro	Text	64				
wilaya	Text	255		wilaya	district_name	District
h4	Text	64	Kata	h4	ward_name	Ward
h5	Text	64	Kijiji	h5	village_name	Village
h6	Text	255	Kitongoji	h6	sub_village_name	sub-village
h6a	Double	8	Code Kitongoji	h6a	sub_village_code	code of sub-village
h7	Double	8	Namba Kaya	h7	household	household number
h3a	Double	8	Latitudes	h3a	latitude	GPS latitude
h3b	Double	8	Longitude	h3b	longitude	GPS longitude
h2	Text	64	InterviewerCode	h2	interviewer	Interviewer code
h1	Date/Time	8	Tarehe	h1	date	date of interview
h8	Text	1	Ridhaa	h8	consent	consent given
h9	Text	255	Jina1Kaya	h9	hhh_first_name	first name of head of household
h10	Text	255	Jina2Kaya	h10	hhh_last_name	last name of head of household
h11	Memo	-	Jinsia MkuuKaya	h11	hhh_sex	sex of head of household
h12	Double	8	Idadi watu	h12	n_hh	total number of people in household
h13	Double	8	Idadi Vijana	h13	n_youth_hh	total number of young people in household
cont	Text	64	Gusa Vijana	cont		
end	Text	64	end	end		
InternalID	Text	255	internalID	interalid	link	link to other tables
Support	Double	8	support	support		?
h4a	Text	255	Code Kata	h4a	ward_code	code of ward
h5a	Text	255	Code Kijiji	h5a	village_code	code of village
StartTime	Date/Time	8		starttime		start time of interview
EndTime	Date/Time	8		endtime		end time of interview
TimeTaken	Date/Time	8		timetaken		duration of interview
				kayaid		unique ID for each kaya

comments (as at 21.06.07)
always = 0
always = 0
format MKV1FSnn
always = 'TOKA'
not unique within subvillage
yes/no
free text
free text
always = VIJANA
always = MALIZA
unique within PDA; based on timestamp
always null
always null
~ 1 second after timestamp
only if endtime is present

MEMA kwa Vijana Trial Further Survey (2007-2008)

HIV Follow-up Form

SECTION A			
Team	ST1		ST2
Staff code of counsellor	|__||__||__|		
Date of rapid test	|__||__|/ |__||__|/ |__||__|		
STICKER NUMBER			
Rapid Result	Overall test<br>P N D	P N<br>Determine	Bioline<br>P N
SECTION B			
Final ELISA Result	P N D		
SECTION C			
Team	Treatment 1		Treatment 2
Staff code of counsellor	|__||__||__|		
Repeat test	Y N		
Date of repeat rapid test	|__||__|/ |__||__|/ |__||__|		
Repeat Rapid Result	Bioline<br>P N		Determine<br>P N
Blood Sample Taken for analysis at NIMR	Y N		
Comments			

HERPES SIMPLEX VIRUS TYPE 2 Testing Algorithm

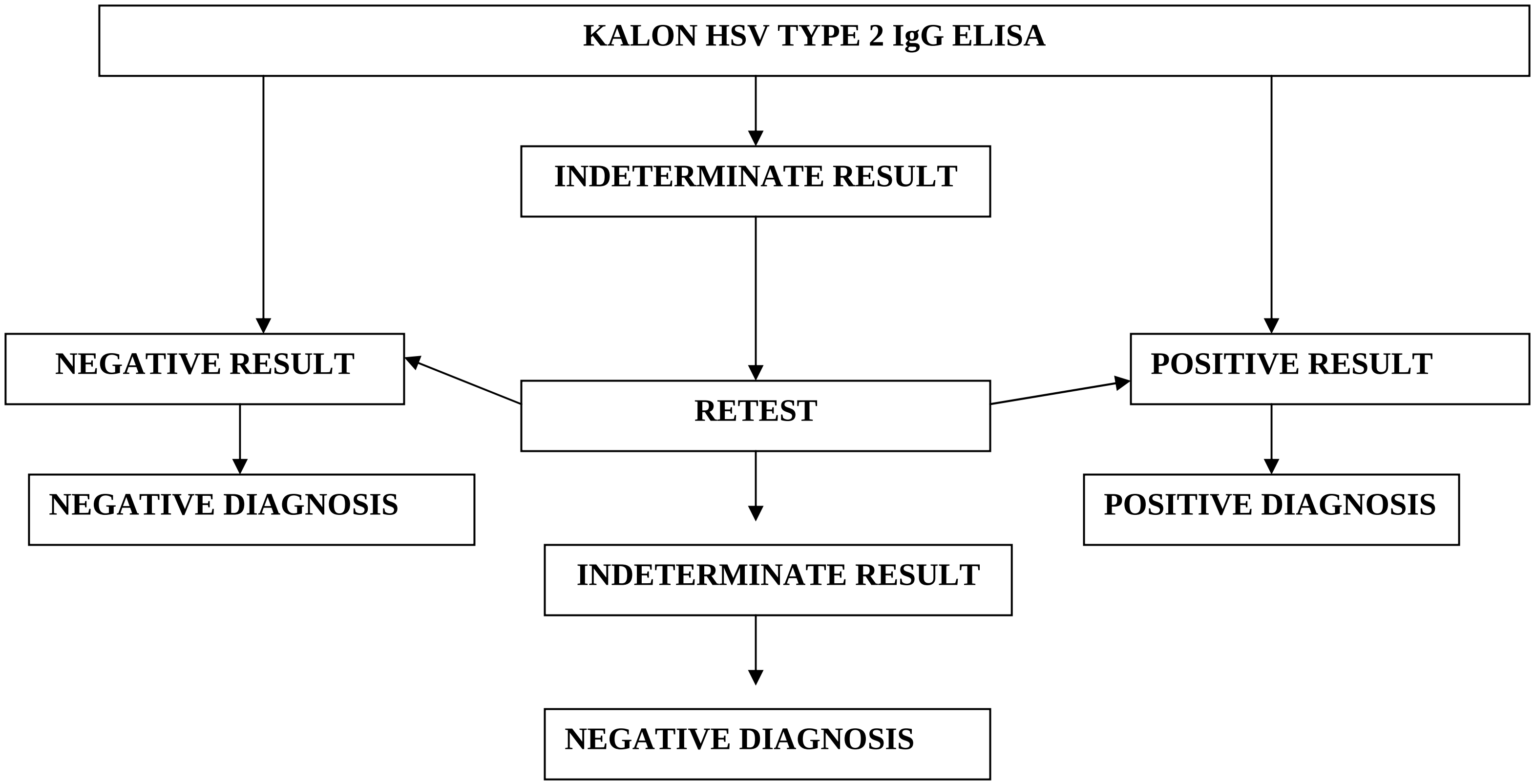

Syphilis Testing Algorithm

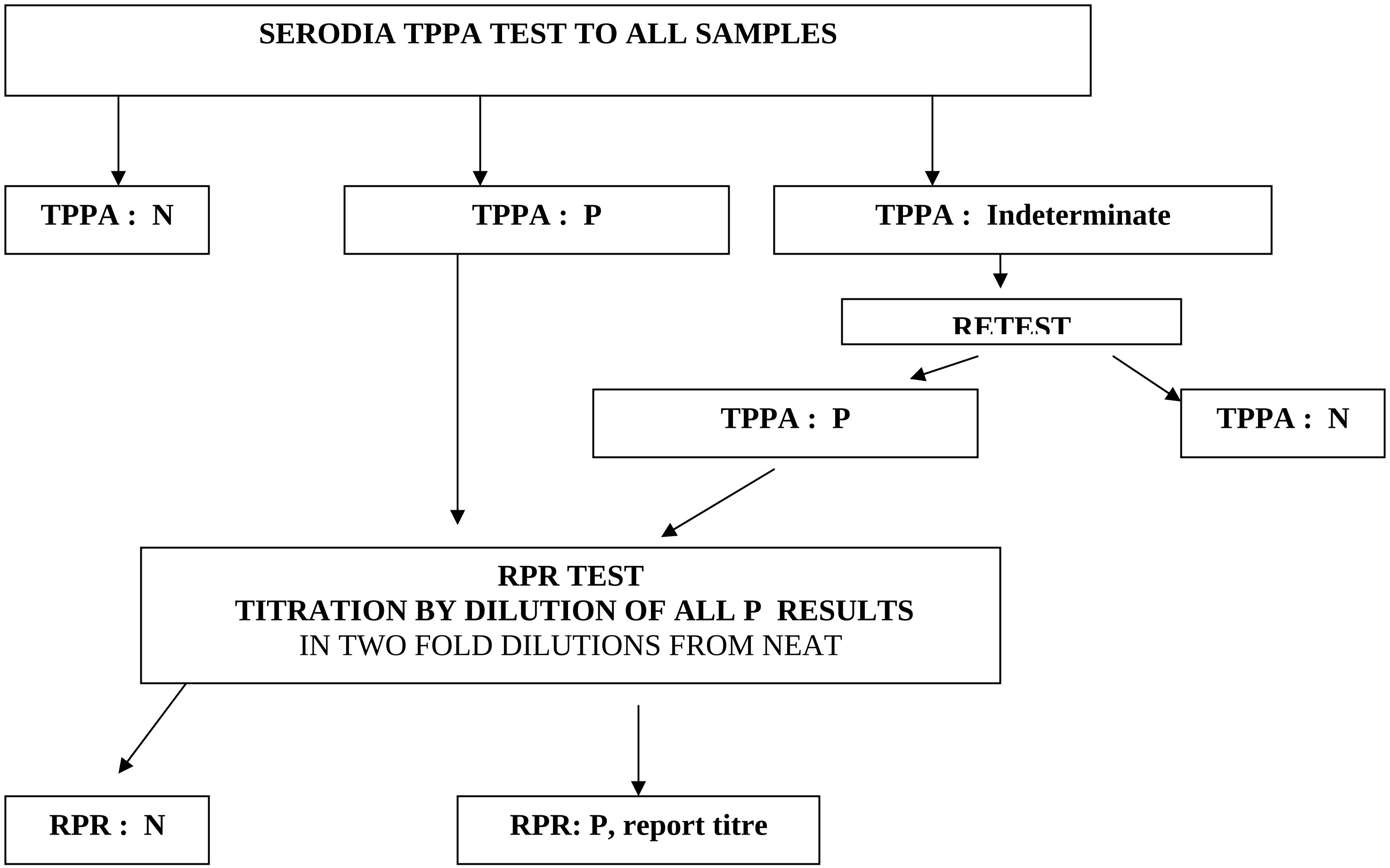

INTERPRETATION OF RESULTS:

TPPA positive RPR positive: Active syphilis
TPPA positive RPR Negative: Past/treated syphilis
TPPA Negative: Negative for syphilis

Key:

TPPA: *Treponema pallidum* Particle-Agglutination Test
RPR: Rapid Plasma Reagin Test
TP: *Treponema pallidum*
P Positive
N Negative

MkV1FS CT AND NG AMPLICOR (ROCHE) Testing Algorithm

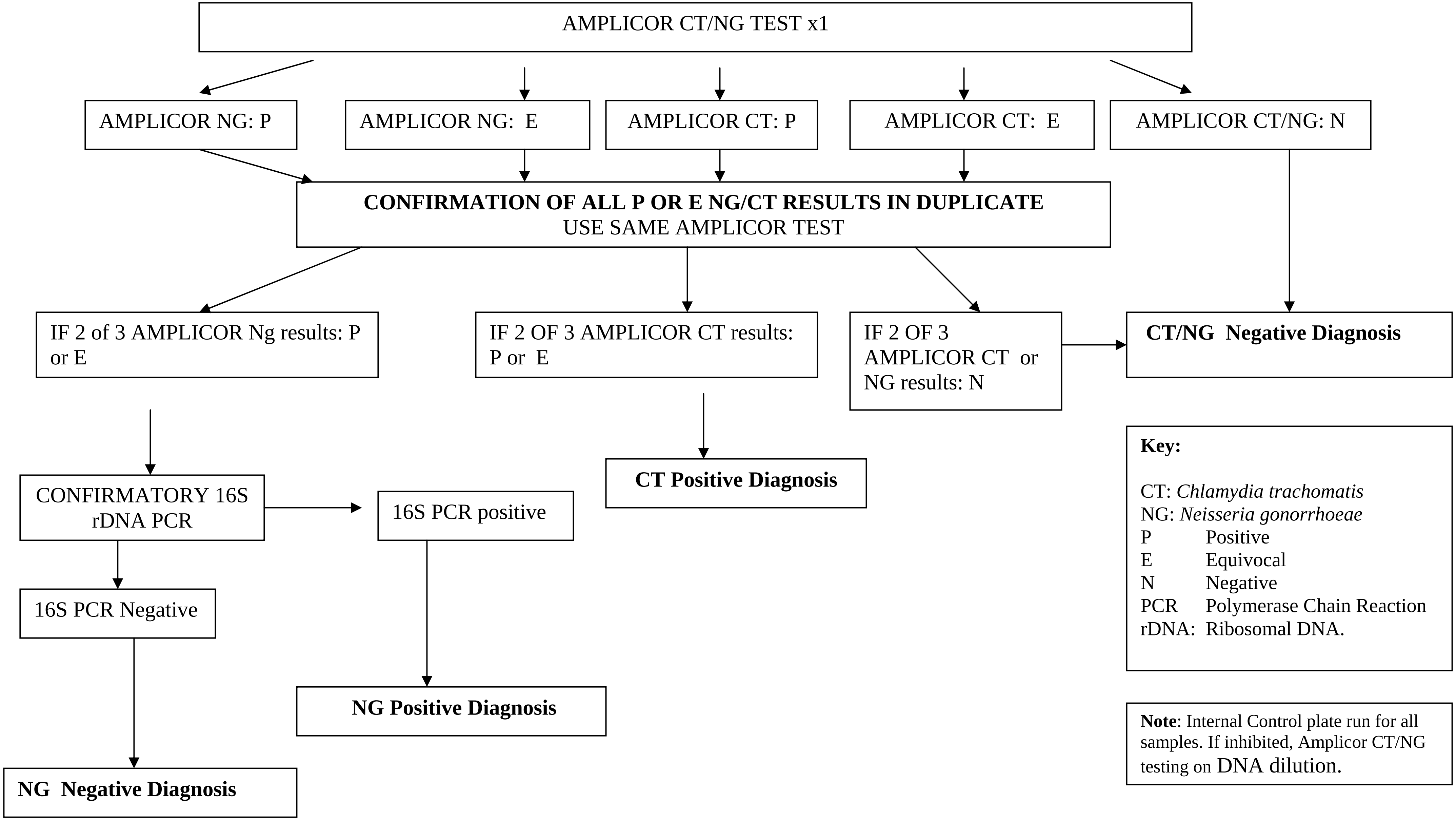

MEMA kwa Vijana Trial Further Survey (2007-2008)

MkV1FS Protocol

Data Submission Form

PART B (i) OFFICE SECTION - CENSUS

	By person who submitted data	By OfficeSection
Date of submission	|__||__|/ |__||__|/ |__||__|	|__||__|/ |__||__|/ |__||__|
Staff code of person submitting/receiving the forms	|__||__||__|	|__||__||__|
Certified correct by (Staff code)	…………	|__||__||__|
Community Code	|__||__||__|	|__||__||__|

CI FORMS

Date	TOTAL - FIELD	TOTAL - OFFICE
|__||__|/ |__||__|/ |__||__|	|__||__||__|	|__||__||__|
|__||__|/ |__||__|/ |__||__|	|__||__||__|	|__||__||__|
|__||__|/ |__||__|/ |__||__|	|__||__||__|	|__||__||__|
|__||__|/ |__||__|/ |__||__|	|__||__||__|	|__||__||__|
|__||__|/ |__||__|/ |__||__|	|__||__||__|	|__||__||__|
|__||__|/ |__||__|/ |__||__|	|__||__||__|	|__||__||__|

**HH CONSENT FORM**	**FIELD**	**OFFICE**
	|__||__||__|	|__||__||__|
**PARENT CONSENT FORM (CENSUS)**		
	|__||__||__|	|__||__||__|

HOUSE HOLD HEAD LIST

**Village(s)**	**FIELD**	**OFFICE**
	|__||__||__|	|__||__||__|
	|__||__||__|	|__||__||__|
	|__||__||__|	|__||__||__|
	|__||__||__|	|__||__||__|
	|__||__||__|	|__||__||__|
	|__||__||__|	|__||__||__|

CENSUS VILLAGE REPORT FORM

Village(s)	FIELD	OFFICE
	|__||__||__|	|__||__||__|
	|__||__||__|	|__||__||__|
	|__||__||__|	|__||__||__|
	|__||__||__|	|__||__||__|
	|__||__||__|	|__||__||__|
	|__||__||__|	|__||__||__|

CENSUS COMMUNITY REPORT FORM

Community	FIELD	OFFICE
	|__||__||__|	|__||__||__|

VILLAGE INFORMATION SHEET

Village(s)	FIELD	OFFICE
	|__||__||__|	|__||__||__|
	|__||__||__|	|__||__||__|
	|__||__||__|	|__||__||__|
	|__||__||__|	|__||__||__|
	|__||__||__|	|__||__||__|
	|__||__||__|	|__||__||__|
	|__||__||__|	|__||__||__|
	|__||__||__|	|__||__||__|

- 03 -

	LIST A 1	
**Village**	**FIELD**	**OFFICE**
	|__||__||__|	|__||__||__|
	|__||__||__|	|__||__||__|
	|__||__||__|	|__||__||__|
	|__||__||__|	|__||__||__|
	|__||__||__|	|__||__||__|
	|__||__||__|	|__||__||__|

	LIST A 2	
**Village**	**FIELD**	**OFFICE**
	|__||__||__|	|__||__||__|
	|__||__||__|	|__||__||__|
	|__||__||__|	|__||__||__|
	|__||__||__|	|__||__||__|
	|__||__||__|	|__||__||__|
	|__||__||__|	|__||__||__|

	LIST A 3	
**Village**	**FIELD**	**OFFICE**
	|__||__||__|	|__||__||__|
	|__||__||__|	|__||__||__|
	|__||__||__|	|__||__||__|
	|__||__||__|	|__||__||__|
	|__||__||__|	|__||__||__|
	|__||__||__|	|__||__||__|

- 04 -

PART B (ii) OFFICE SECTION - SURVEY

	By person who submitted data	By OfficeSection
Date of submission	|__||__|/ |__||__|/ |__||__|	|__||__|/ |__||__|/ |__||__|
Staff code of person submitting/receiving the forms	|__||__||__|	|__||__||__|
Certified correct by (Staff code)	…………	|__||__||__|
Community Code	|__||__||__|	|__||__||__|

SURVEY COMMUNITY REPORT FORM

	FIELD	OFFICE
SURVEY COMMUNITY REPORT FORM	|__||__||__|	|__||__||__|

TRACING FORM

Village(s)	FIELD	OFFICE
	|__||__||__|	|__||__||__|
	|__||__||__|	|__||__||__|
	|__||__||__|	|__||__||__|
	|__||__||__|	|__||__||__|
	|__||__||__|	|__||__||__|
	|__||__||__|	|__||__||__|

SURVEY VILLAGE REPORT FORM

Village(s)	FIELD	OFFICE
	|__||__||__|	|__||__||__|
	|__||__||__|	|__||__||__|
	|__||__||__|	|__||__||__|
	|__||__||__|	|__||__||__|
	|__||__||__|	|__||__||__|
	|__||__||__|	|__||__||__|

STI TREATMENT

Village(s)	FIELD	OFFICE
	|__||__||__|	|__||__||__|
	|__||__||__|	|__||__||__|
	|__||__||__|	|__||__||__|
	|__||__||__|	|__||__||__|
	|__||__||__|	|__||__||__|
	|__||__||__|	|__||__||__|

VCT DISCORDANT

Village(s)	FIELD	OFFICE
	|__||__||__|	|__||__||__|
	|__||__||__|	|__||__||__|
	|__||__||__|	|__||__||__|

PARTICIPANT CONSENT FORM

Village(s)	FIELD	OFFICE
	|__||__||__|	|__||__||__|
	|__||__||__|	|__||__||__|
	|__||__||__|	|__||__||__|

WALKMAN LENDING FORM

Village(s)	FIELD	OFFICE
	|__||__||__|	|__||__||__|
	|__||__||__|	|__||__||__|
	|__||__||__|	|__||__||__|

REQUEST FOR HIV TEST & RESULTS FORM

Village(s)	FIELD	OFFICE
	|__||__||__|	|__||__||__|
	|__||__||__|	|__||__||__|
	|__||__||__|	|__||__||__|

- 06 -

STI TREATMENT

Village(s)	FIELD	OFFICE
	|__||__||__|	|__||__||__|
	|__||__||__|	|__||__||__|
	|__||__||__|	|__||__||__|

HIV REFERRAL FORM

Village(s)	FIELD	OFFICE
	|__||__||__|	|__||__||__|
	|__||__||__|	|__||__||__|
	|__||__||__|	|__||__||__|

INVITATION LETTER / MWALIKO FORM

Village(s)	FIELD	OFFICE
	|__||__||__|	|__||__||__|
	|__||__||__|	|__||__||__|
	|__||__||__|	|__||__||__|
	|__||__||__|	|__||__||__|
	|__||__||__|	|__||__||__|
	|__||__||__|	|__||__||__|

MEMA kwa Vijana Trial Further Survey (2007-2008)

Data Submission Form

<table>
<tr><th></th><th>By person who submitted data</th><th>By Data Section</th></tr>
<tr><td>Date of submission</td><td>|__||__|/ |__||__|/ |__||__|</td><td>|__||__|/ |__||__|/ |__||__|</td></tr>
<tr><td>Staff code of person submitting/receiving the forms</td><td>|__||__||__|</td><td>|__||__||__|</td></tr>
<tr><td>Certified correct by (Staff code)</td><td>…………</td><td>|__||__||__|</td></tr>
<tr><td>Community Code</td><td>|__||__||__|</td><td>|__||__||__|</td></tr>
</table>

CENSUS

<table>
<tr><th colspan="4">BACKUP QUESTIONNAIRE</th></tr>
<tr><th>Date</th><th>Date</th><th>TOTAL - FIELD</th><th>TOTAL - DATA SECT.</th></tr>
<tr><td>|__||__|/ |__||__|/ |__||__|</td><td>|__||__|/ |__||__|/ |__||__|</td><td>|__||__||__|</td><td>|__||__||__|</td></tr>
<tr><td>|__||__|/ |__||__|/ |__||__|</td><td>|__||__|/ |__||__|/ |__||__|</td><td>|__||__||__|</td><td>|__||__||__|</td></tr>
<tr><td>|__||__|/ |__||__|/ |__||__|</td><td>|__||__|/ |__||__|/ |__||__|</td><td>|__||__||__|</td><td>|__||__||__|</td></tr>
<tr><td>|__||__|/ |__||__|/ |__||__|</td><td>|__||__|/ |__||__|/ |__||__|</td><td>|__||__||__|</td><td>|__||__||__|</td></tr>
<tr><td>|__||__|/ |__||__|/ |__||__|</td><td>|__||__|/ |__||__|/ |__||__|</td><td>|__||__||__|</td><td>|__||__||__|</td></tr>
<tr><td>|__||__|/ |__||__|/ |__||__|</td><td>|__||__|/ |__||__|/ |__||__|</td><td>|__||__||__|</td><td>|__||__||__|</td></tr>
</table>

<table>
<tr><th colspan="4">CD</th></tr>
<tr><th>Date</th><th>Date</th><th>TOTAL - FIELD</th><th>TOTAL - DATA SECT.</th></tr>
<tr><td>|__||__|/ |__||__|/ |__||__|</td><td>|__||__|/ |__||__|/ |__||__|</td><td>|__||__||__|</td><td>|__||__||__|</td></tr>
<tr><td>|__||__|/ |__||__|/ |__||__|</td><td>|__||__|/ |__||__|/ |__||__|</td><td>|__||__||__|</td><td>|__||__||__|</td></tr>
<tr><td>|__||__|/ |__||__|/ |__||__|</td><td>|__||__|/ |__||__|/ |__||__|</td><td>|__||__||__|</td><td>|__||__||__|</td></tr>
<tr><td>|__||__|/ |__||__|/ |__||__|</td><td>|__||__|/ |__||__|/ |__||__|</td><td>|__||__||__|</td><td>|__||__||__|</td></tr>
<tr><td>|__||__|/ |__||__|/ |__||__|</td><td>|__||__|/ |__||__|/ |__||__|</td><td>|__||__||__|</td><td>|__||__||__|</td></tr>
<tr><td>|__||__|/ |__||__|/ |__||__|</td><td>|__||__|/ |__||__|/ |__||__|</td><td>|__||__||__|</td><td>|__||__||__|</td></tr>
</table>

Signature of person submitting the Forms

Comments by Data Section

Signature of person receiving the Forms

MEMA kwa Vijana Trial Further Survey (2007-2008)

1. Use these numbers when you do not have a household number from the mobilisation list of HH
2. Make sure that you cross through the number when you use it to avoid using the same number twice

901	925	950	975
902	926	951	976
903	927	952	977
904	928	953	978
905	929	954	979
906	930	955	980
907	931	956	981
908	932	957	982
909	933	958	983
910	934	959	984
911	935	960	985
912	936	961	986
913	937	962	987
914	938	963	988
915	939	964	989
916	940	965	990
917	941	966	991
918	942	967	992
919	943	968	993
920	944	969	994
921	945	970	995
922	946	971	996
923	947	972	997
924	948	973	998
	949	974	999

Extra Numbers to be used by the CI whenever the PDAs don't work.June08

90001	90057	90113	90169
90002	90058	90114	90170
90003	90059	90115	90171
90004	90060	90116	90172
90005	90061	90117	90173
90006	90062	90118	90174
90007	90063	90119	90175
90008	90064	90120	90176
90009	90065	90121	90177
90010	90066	90122	90178
90011	90067	90123	90179
90012	90068	90124	90180
90013	90069	90125	90181
90014	90070	90126	90182
90015	90071	90127	90183
90016	90072	90128	90184
90017	90073	90129	90185
90018	90074	90130	90186
90019	90075	90131	90187
90020	90076	90132	90188
90021	90077	90133	90189
90022	90078	90134	90190
90023	90079	90135	90191
90024	90080	90136	90192
90025	90081	90137	90193
90026	90082	90138	90194
90027	90083	90139	90195
90028	90084	90140	90196
90029	90085	90141	90197
90030	90086	90142	90198
90031	90087	90143	90199
90032	90088	90144	90200
90033	90089	90145	
90034	90090	90146	
90035	90091	90147	
90036	90092	90148	
90037	90093	90149	
90038	90094	90150	
90039	90095	90151	
90040	90096	90152	
90041	90097	90153	
90042	90098	90154	
90043	90099	90155	
90044	90100	90156	
90045	90101	90157	
90046	90102	90158	
90047	90103	90159	
90048	90104	90160	
90049	90105	90161	
90050	90106	90162	
90051	90107	90163	
90052	90108	90164	
90053	90109	90165	
90054	90110	90166	

90055	90111	90167
90056	90112	90168

MEMA kwa Vijana Trial Further Survey, NIMR, PO Box 11936, Mwanza

CONTACT REFERRAL SLIP

Clinician's Name:……………………..………………………………………………………

Date of diagnosis: Date|__||__|/ Mth|__||__|/ Yr 20|__||__|

Index Sticker No.: |__||__||__||__||__||__||__||__||__||__|

Ndugu;
Kutokana na utaratibu wetu wa Utafiti inatupasa tukukaribishe tena kwenye kituo cha Afya kilicho karibu nawe.
Wafanyakazi wa kituo watakuona kwa maelezo zaidi.

Message to the Clinician at the referral health unit: Please treat this person. (S)he is a contact of someone I treated for the condition (s) ***(circle condition(s))***:

Chlamydia **Gonorrhoea** **Syphilis**

Other condition (specify): ____

………

MEMA kwa Vijana Trial Further Survey, NIMR, PO Box 11936, Mwanza

CONTACT REFERRAL SLIP

Clinician's Name:……………………..………………………………………………………

Date of diagnosis: Date|__||__|/ Mth|__||__|/ Yr 20|__||__|

Index Sticker No.: |__||__||__||__||__||__||__||__||__||__|

Ndugu;
Kutokana na utaratibu wetu wa Utafiti inatupasa tukukaribishe tena kwenye kituo cha Afya kilicho karibu nawe.
Wafanyakazi wa kituo watakuona kwa maelezo zaidi..

Message to the Clinician at the referral health unit: Please treat this person. (S)he is a contact of someone I treated for the condition (s) ***(circle condition(s))***:

Chlamydia **Gonorrhoea** **Syphilis**

Other condition (specify): ____

MEMA kwa Vijana Trial Further Survey, NIMR, PO Box 11936, Mwanza

CLIENT REFERRAL SLIP

Clinician's Name:…………………….…………………………………………………….

Date : Date|__||__|/ Mth|__||__|/ Yr 20|__||__|

Index Sticker No.: |__||__||__||__||__||__||__||__||__|__|

Ndugu;
Kutokana na utaratibu wetu wa utafiti inatupasa tukukaribishe tena kwenye kituo cha afya kilicho karibu nawe: ____
Wafanyakazi wa kituo watakuona kwa maelezo zaidi.

Message to the Clinician at the referral health unit: Please take care of this person (s)he is on the MkV1 FS list of participants given you to by a MkV1 FS staff member .

MEMA kwa Vijana Trial Further Survey, NIMR, PO Box 11936, Mwanza

CLIENT REFERRAL SLIP

Clinician's Name:…………………….…………………………………………………….

Date : Date|__||__|/ Mth|__||__|/ Yr 20|__||__|

Index Sticker No.: |__||__||__||__||__||__||__||__||__||__|

Ndugu;
Kutokana na utaratibu wetu wa utafiti inatupasa tukukaribishe tena kwenye kituo cha afya kilicho karibu nawe: ____
Wafanyakazi wa kituo watakuona kwa maelezo zaidi.

Message to the Clinician at the referral health unit: Please treat this person. (S)he is on the MkV1 FS list of participants given you to by a MkV1 FS staff member .

MEMA kwa Vijana Trial Further Survey, NIMR, PO Box 11936, Mwanza

CLIENT REFERRAL SLIP

Clinician's Name:…………………….…………………………………………………….

Date : Date|__||__|/ Mth|__||__|/ Yr 20|__||__|

Index Sticker No.: |__||__||__||__||__||__||__||__||__||__|

Ndugu;
Kutokana na utaratibu wetu wa utafiti inatupasa tukukaribishe tena kwenye kituo cha afya kilicho karibu nawe: ____
Wafanyakazi wa kituo watakuona kwa maelezo zaidi.

Message to the Clinician at the referral health unit: Please treat this person. (S)he is on the MkV1 FS list of participants given you to by a MkV1 FS staff member .

MEMA kwa Vijana Trial Further Survey (2007-2008)

FORM YA RIDHAA YA MATIBABU.

Mimi ………………………………….. ambaye ni mshiriki/si mshiriki wa mradi wa MEMA kwa vijana 1 FS. Ninakubali kwa hiari yangu kutibiwa ugonjwa uliogunduliwa na watafiti baada ya kuchukuliwa vipimo.
Tiba hii nitapata mimi mwenyewe pamoja na mume/mke wangu……………………………… Kama nilivyoshauriwa na wataalamu wa afya wa mradi.

Sahihi……………………..
Tarehe…………………………

MEMA kwa Vijana Trial Further Survey (2007-2008)

FORM YA RIDHAA YA MATIBABU.

Mimi ………………………………….. ambaye ni mshiriki/si mshiriki wa mradi wa MEMA kwa vijana 1 FS. Ninakubali kwa hiari yangu kutibiwa ugonjwa uliogunduliwa na watafiti baada ya kuchukuliwa vipimo.
Tiba hii nitapata mimi mwenyewe pamoja na mume/mke wangu……………………………… Kama nilivyoshauriwa na wataalamu wa afya wa mradi.

Sahihi……………………..
Tarehe…………………………

MEMA kwa Vijana Trial Further Survey (2007-2008)

FORM YA RIDHAA YA MATIBABU.

Mimi ………………………………….. ambaye ni mshiriki/si mshiriki wa mradi wa MEMA kwa vijana 1 FS. Ninakubali kwa hiari yangu kutibiwa ugonjwa uliogunduliwa na watafiti baada ya kuchukuliwa vipimo.
Tiba hii nitapata mimi mwenyewe pamoja na mume/mke wangu……………………………… Kama nilivyoshauriwa na wataalamu wa afya wa mradi.

Sahihi……………………..
Tarehe…………………………

(MkV1FS STI-Treatment-Consent-05, April 2008)

MEMA kwa Vijana Trial Further Survey (2007-2008)

FORM YA RIDHAA YA MATIBABU.

Mimi …………………………………….. ambaye ni mshiriki/si mshiriki wa mradi wa MEMA kwa vijana 1 FS. Ninakubali kwa hiari yangu kutibiwa ugonjwa uliogunduliwa na watafiti baada ya kuchukuliwa vipimo.
Tiba hii nitapata mimi mwenyewe pamoja na mume/mke wangu………………………………. Kama nilivyoshauriwa na wataalamu wa afya wa mradi.

Sahihi……………………..
Tarehe…………………………

MEMA kwa Vijana Trial Further Survey (2007-2008)

FORM YA RIDHAA YA MATIBABU.

Mimi …………………………………….. ambaye ni mshiriki/si mshiriki wa mradi wa MEMA kwa vijana 1 FS. Ninakubali kwa hiari yangu kutibiwa ugonjwa uliogunduliwa na watafiti baada ya kuchukuliwa vipimo.
Tiba hii nitapata mimi mwenyewe pamoja na mume/mke wangu………………………………. Kama nilivyoshauriwa na wataalamu wa afya wa mradi.

Sahihi……………………..
Tarehe…………………………

MEMA kwa Vijana Trial Further Survey (2007-2008)

FORM YA RIDHAA YA MATIBABU.

Mimi …………………………………….. ambaye ni mshiriki/si mshiriki wa mradi wa MEMA kwa vijana 1 FS. Ninakubali kwa hiari yangu kutibiwa ugonjwa uliogunduliwa na watafiti baada ya kuchukuliwa vipimo.
Tiba hii nitapata mimi mwenyewe pamoja na mume/mke wangu………………………………. Kama nilivyoshauriwa na wataalamu wa afya wa mradi.

Sahihi……………………..
Tarehe…………………………

(MkV1FS STI-Treatment-Consent-05, April 2008)

MEMA kwa Vijana Trial Further Survey (2007-2008)

FORM YA RIDHAA YA MATIBABU.

Mimi …………………………………….. ambaye ni mshiriki/si mshiriki wa mradi wa MEMA kwa vijana 1 FS. Ninakubali kwa hiari yangu kutibiwa ugonjwa uliogunduliwa na watafiti baada ya kuchukuliwa vipimo.
Tiba hii nitapata mimi mwenyewe pamoja na mume/mke wangu………………………………. Kama nilivyoshauriwa na wataalamu wa afya wa mradi.

Sahihi……………………..
Tarehe…………………………

MEMA kwa Vijana Trial Further Survey (2007-2008)

FORM YA RIDHAA YA MATIBABU.

Mimi …………………………………….. ambaye ni mshiriki/si mshiriki wa mradi wa MEMA kwa vijana 1 FS. Ninakubali kwa hiari yangu kutibiwa ugonjwa uliogunduliwa na watafiti baada ya kuchukuliwa vipimo.
Tiba hii nitapata mimi mwenyewe pamoja na mume/mke wangu………………………………. Kama nilivyoshauriwa na wataalamu wa afya wa mradi.

Sahihi……………………..
Tarehe…………………………

MEMA kwa Vijana Trial Further Survey (2007-2008)

FORM YA RIDHAA YA MATIBABU.

Mimi …………………………………….. ambaye ni mshiriki/si mshiriki wa mradi wa MEMA kwa vijana 1 FS. Ninakubali kwa hiari yangu kutibiwa ugonjwa uliogunduliwa na watafiti baada ya kuchukuliwa vipimo.
Tiba hii nitapata mimi mwenyewe pamoja na mume/mke wangu………………………………. Kama nilivyoshauriwa na wataalamu wa afya wa mradi.

Sahihi……………………..
Tarehe…………………………

(MkV1FS STI-Treatment-Consent-05, April 2008)

MEMA kwa Vijana Trial Further Survey (2007-2008)

FORM YA RIDHAA YA MATIBABU.

Mimi …………………………………….. ambaye ni mshiriki/si mshiriki wa mradi wa MEMA kwa vijana 1 FS. Ninakubali kwa hiari yangu kutibiwa ugonjwa uliogunduliwa na watafiti baada ya kuchukuliwa vipimo.
Tiba hii nitapata mimi mwenyewe pamoja na mume/mke wangu………………………………. Kama nilivyoshauriwa na wataalamu wa afya wa mradi.

Sahihi……………………..
Tarehe…………………………

MEMA kwa Vijana Trial Further Survey (2007-2008)

FORM YA RIDHAA YA MATIBABU.

Mimi …………………………………….. ambaye ni mshiriki/si mshiriki wa mradi wa MEMA kwa vijana 1 FS. Ninakubali kwa hiari yangu kutibiwa ugonjwa uliogunduliwa na watafiti baada ya kuchukuliwa vipimo.
Tiba hii nitapata mimi mwenyewe pamoja na mume/mke wangu………………………………. Kama nilivyoshauriwa na wataalamu wa afya wa mradi.

Sahihi……………………..
Tarehe…………………………

MEMA kwa Vijana Trial Further Survey (2007-2008)

FORM YA RIDHAA YA MATIBABU.

Mimi …………………………………….. ambaye ni mshiriki/si mshiriki wa mradi wa MEMA kwa vijana 1 FS. Ninakubali kwa hiari yangu kutibiwa ugonjwa uliogunduliwa na watafiti baada ya kuchukuliwa vipimo.
Tiba hii nitapata mimi mwenyewe pamoja na mume/mke wangu………………………………. Kama nilivyoshauriwa na wataalamu wa afya wa mradi.

Sahihi……………………..
Tarehe…………………………

(MkV1FS STI-Treatment-Consent-05, April 2008)

MEMA kwa Vijana Trial Further Survey (2007-2008)

FORM YA RIDHAA YA MATIBABU.

Mimi …………………………………….. ambaye ni mshiriki/si mshiriki wa mradi wa MEMA kwa vijana 1 FS. Ninakubali kwa hiari yangu kutibiwa ugonjwa uliogunduliwa na watafiti baada ya kuchukuliwa vipimo.
Tiba hii nitapata mimi mwenyewe pamoja na mume/mke wangu………………………………. Kama nilivyoshauriwa na wataalamu wa afya wa mradi.

Sahihi……………………..
Tarehe…………………………

MEMA kwa Vijana Trial Further Survey (2007-2008)

FORM YA RIDHAA YA MATIBABU.

Mimi …………………………………….. ambaye ni mshiriki/si mshiriki wa mradi wa MEMA kwa vijana 1 FS. Ninakubali kwa hiari yangu kutibiwa ugonjwa uliogunduliwa na watafiti baada ya kuchukuliwa vipimo.
Tiba hii nitapata mimi mwenyewe pamoja na mume/mke wangu………………………………. Kama nilivyoshauriwa na wataalamu wa afya wa mradi.

Sahihi……………………..
Tarehe…………………………

MEMA kwa Vijana Trial Further Survey (2007-2008)

FORM YA RIDHAA YA MATIBABU.

Mimi …………………………………….. ambaye ni mshiriki/si mshiriki wa mradi wa MEMA kwa vijana 1 FS. Ninakubali kwa hiari yangu kutibiwa ugonjwa uliogunduliwa na watafiti baada ya kuchukuliwa vipimo.
Tiba hii nitapata mimi mwenyewe pamoja na mume/mke wangu………………………………. Kama nilivyoshauriwa na wataalamu wa afya wa mradi.

Sahihi……………………..
Tarehe…………………………

(MkV1FS STI-Treatment-Consent-05, April 2008)

MEMA kwa Vijana Trial Further Survey (2007-2008)

FORM YA RIDHAA YA MATIBABU.

Mimi …………………………………….. ambaye ni mshiriki/si mshiriki wa mradi wa MEMA kwa vijana 1 FS. Ninakubali kwa hiari yangu kutibiwa ugonjwa uliogunduliwa na watafiti baada ya kuchukuliwa vipimo.
Tiba hii nitapata mimi mwenyewe pamoja na mume/mke wangu………………………………. Kama nilivyoshauriwa na wataalamu wa afya wa mradi.

Sahihi……………………..
Tarehe…………………………

MEMA kwa Vijana Trial Further Survey (2007-2008)

FORM YA RIDHAA YA MATIBABU.

Mimi …………………………………….. ambaye ni mshiriki/si mshiriki wa mradi wa MEMA kwa vijana 1 FS. Ninakubali kwa hiari yangu kutibiwa ugonjwa uliogunduliwa na watafiti baada ya kuchukuliwa vipimo.
Tiba hii nitapata mimi mwenyewe pamoja na mume/mke wangu………………………………. Kama nilivyoshauriwa na wataalamu wa afya wa mradi.

Sahihi……………………..
Tarehe…………………………

MEMA kwa Vijana Trial Further Survey (2007-2008)

FORM YA RIDHAA YA MATIBABU.

Mimi …………………………………….. ambaye ni mshiriki/si mshiriki wa mradi wa MEMA kwa vijana 1 FS. Ninakubali kwa hiari yangu kutibiwa ugonjwa uliogunduliwa na watafiti baada ya kuchukuliwa vipimo.
Tiba hii nitapata mimi mwenyewe pamoja na mume/mke wangu………………………………. Kama nilivyoshauriwa na wataalamu wa afya wa mradi.

Sahihi……………………..
Tarehe…………………………

(MkV1FS STI-Treatment-Consent-05, April 2008)

MEMA kwa Vijana Trial Further Survey (2007-2008)

FORM YA RIDHAA YA MATIBABU.

Mimi …………………………………….. ambaye ni mshiriki/si mshiriki wa mradi wa MEMA kwa vijana 1 FS. Ninakubali kwa hiari yangu kutibiwa ugonjwa uliogunduliwa na watafiti baada ya kuchukuliwa vipimo.
Tiba hii nitapata mimi mwenyewe pamoja na mume/mke wangu……………………………… Kama nilivyoshauriwa na wataalamu wa afya wa mradi.

Sahihi……………………..
Tarehe…………………………

MEMA kwa Vijana Trial Further Survey (2007-2008)

FORM YA RIDHAA YA MATIBABU.

Mimi …………………………………….. ambaye ni mshiriki/si mshiriki wa mradi wa MEMA kwa vijana 1 FS. Ninakubali kwa hiari yangu kutibiwa ugonjwa uliogunduliwa na watafiti baada ya kuchukuliwa vipimo.
Tiba hii nitapata mimi mwenyewe pamoja na mume/mke wangu……………………………… Kama nilivyoshauriwa na wataalamu wa afya wa mradi.

Sahihi……………………..
Tarehe…………………………

MEMA kwa Vijana Trial Further Survey (2007-2008)

FORM YA RIDHAA YA MATIBABU.

Mimi …………………………………….. ambaye ni mshiriki/si mshiriki wa mradi wa MEMA kwa vijana 1 FS. Ninakubali kwa hiari yangu kutibiwa ugonjwa uliogunduliwa na watafiti baada ya kuchukuliwa vipimo.
Tiba hii nitapata mimi mwenyewe pamoja na mume/mke wangu……………………………… Kama nilivyoshauriwa na wataalamu wa afya wa mradi.

Sahihi……………………..
Tarehe…………………………

(MkV1FS STI-Treatment-Consent-05, April 2008)

MEMA kwa Vijana Trial Further Survey (2007-2008)

FORM YA RIDHAA YA MATIBABU.

Mimi …………………………………….. ambaye ni mshiriki/si mshiriki wa mradi wa MEMA kwa vijana 1 FS. Ninakubali kwa hiari yangu kutibiwa ugonjwa uliogunduliwa na watafiti baada ya kuchukuliwa vipimo.
Tiba hii nitapata mimi mwenyewe pamoja na mume/mke wangu………………………………. Kama nilivyoshauriwa na wataalamu wa afya wa mradi.

Sahihi……………………..
Tarehe…………………………

MEMA kwa Vijana Trial Further Survey (2007-2008)

FORM YA RIDHAA YA MATIBABU.

Mimi …………………………………….. ambaye ni mshiriki/si mshiriki wa mradi wa MEMA kwa vijana 1 FS. Ninakubali kwa hiari yangu kutibiwa ugonjwa uliogunduliwa na watafiti baada ya kuchukuliwa vipimo.
Tiba hii nitapata mimi mwenyewe pamoja na mume/mke wangu………………………………. Kama nilivyoshauriwa na wataalamu wa afya wa mradi.

Sahihi……………………..
Tarehe…………………………

MEMA kwa Vijana Trial Further Survey (2007-2008)

FORM YA RIDHAA YA MATIBABU.

Mimi …………………………………….. ambaye ni mshiriki/si mshiriki wa mradi wa MEMA kwa vijana 1 FS. Ninakubali kwa hiari yangu kutibiwa ugonjwa uliogunduliwa na watafiti baada ya kuchukuliwa vipimo.
Tiba hii nitapata mimi mwenyewe pamoja na mume/mke wangu………………………………. Kama nilivyoshauriwa na wataalamu wa afya wa mradi.

Sahihi……………………..
Tarehe…………………………

(MkV1FS STI-Treatment-Consent-05, April 2008)

MEMA kwa Vijana Trial Further Survey, NIMR, PO Box 11936, Mwanza

Huu ni mualiko wa <Jina> ________________________________

Tafadhali njoo kwenye <*eneo*> ____________________ **katika <*kijiji*>** ______

<*siku*> _______________________ **<*Tarehe*>** ________________**200**___ **<saa>**___________

Study ID: FS

MEMA kwa Vijana Trial Further Survey, NIMR, PO Box 11936, Mwanza

Huu ni mualiko wa <Jina> ________________________________

Tafadhali njoo kwenye <*eneo*> ____________________ **katika <*kijiji*>** ______

<*siku*> _______________________ **<*Tarehe*>** ________________**200**___ **<saa>** ________

Study ID: FS

MEMA kwa Vijana Trial Further Survey, NIMR, PO Box 11936, Mwanza

Huu ni mualiko wa <Jina> ________________________________

Tafadhali njoo kwenye <*eneo*> ____________________ **katika <*kijiji*>** ______

<*siku*> _______________________ **<*Tarehe*>** ________________**200**___ **<saa>** ________

Study ID: FS

MkV1 FS – Mop-up Protocol

1. Background:

Our power calculations were based on an expected HIV prevalence in the comparison communities of 2.0% in males and 5.0% in females, and 365 males and the same number of females seen in each community.

During the first sweep through the first 18 communities (excluding one community (Katoro) where massive in-migration to the main village in the community meant that the time allotted was grossly inadequate), the survey teams have seen an average of 340 males (93% of expected) and 273 females (76% of expected) per community (Table 1). Initial data collection will be completed in all of the 20 trial communities (including Katoro) by the end of May 2008.

Table 1 Recruitment of eligible young people during MkV1 FS

Order of visiting community	Team	Community (cmty)	Interv(I) Comp(C) & stratum no.	No. Hholds	YP Invited by census	Participants (expect 730 per cmty)				
						M*	F*	TOTAL	% of expected	Moved Away Form
								n		
1	T1	Katunguru (5)	I1	3301	?	293	222	516	70.7	0
2	T2	Koromije (22)	C1	2359	787	314	318	628	86.0	13
3	T1	Kasamwa (11)	I1	4292	888	417	314	762	104.4	45
4	T2	Mwagi (23)	C1	3252	763	307	325	632	86.6	46
5	T1	Nyangw'hale (14)	C2	2644	786	392	270	639	87.5	16
6	T2	Malya (24)	I2	3847	908	378	334	701	96.0	77
7	T1	Bukoli (13)	C1	2965	688	316	200	516	70.7	115
8	T2	Misasi (18)	I1	2473	703	339	250	588	80.5	55
9	T1	Katoro (8)	I2	3069	524	247	159	406	55.6	88
10	T2	Usagara (21)	C2	3483	799	329	289	618	84.7	102
11	T1	Kagu (9)	I3	6518	815	367	227	594	81.4	99
12	T2	Nyakalilo(4)	C3	3814	856	363	283	646	88.5	146
13	T1	Lubanga (10)	C3	3242	686	270	264	535	73.3	150
14	T2	Katwe (2)	I3	3679	911	263	237	559	76.6	252
15	T1	Busisi (6)	C2	2873	712	282	215	497	68.1	245
16	T2	Nyehunge (3)	I2	4654	1005	358	334	692	94.8	218
17	T1	Nkome (1)	C3	5086	888	337	264	601	82.3	
18	T2	Fukalo (17)	I3	3459	1029	467	354	839	114.9	
19	T1	Ihanamilo (12)	I2	2619	739	322	214	536	73.4	
20	T2	Hungumalwa (16)	C2	4004						
TOTAL				71,633		6361	5073	11505		
Average				3,582		340	273	617		
% of expect						93.1	74.8	84.5		

** NB The numbers of males and females interviewed in a community do not always add up to the total number in the community. This is due to errors in reporting from the field. The total numbers interviewed are more reliable but I have left in the numbers of each sex as they are indicative of the real numbers interviewed.*

Table 2 shows that the provisional prevalence of HIV in the first eight comparison communities is slightly lower than expected in both males (1.6% vs 2.0%) and females (3.7% vs 5.0%), whereas the prevalence of HSV2 has been roughly as expected (23% vs 25% in males, 41% vs 35% in females). Active syphilis prevalence is also much lower than estimated (2.7% vs 6.5% in males, and 4.5% vs 10.0% in females). There were no specific estimates for CT and NG prevalence, but CT was expected to be much lower than syphilis and roughly similar to HIV, with NG lower than this.

Table 2 Provisional prevalence of biological outcomes in first eight comparison communities (* CT/NG in first 6 comparison communities only)

	**Male**		**Female**	
	No.	*%*	*No.*	*%*
**HIV**	39	1.6	79	3.7
**HSV2**	594	23.4	874	40.9
**Syphilis (active)**	67	2.7	94	4.5
**CT***	35	1.8	35	2.1
**NG***	18	0.9	21	1.3

NOTE:

- Each sample is tested for HIV by two independent ELISA tests. Unresolved HIV discrepant samples (ie. one test positive, the other negative; about 8.5% of all samples) have been counted as negative. It is possible that some of these discrepant samples will eventually prove to have been HIV positive.
- All NG positives will need to be confirmed by a second, independent PCR test, and some may end up with a final result of being negative
- **Overall, all these results should be considered highly provisional, and confidential**

A simple analysis of the MkV1 2001-02 mopup data shows that a higher proportion of females and ever married were found during mop-up when compared to the main survey (Table 4). There is some indication for both males and females that those interviewed during mop-up were at higher risk of STIs, but in general numbers are too small to draw any definite conclusions.

Table 3: Quick analysis of MkV1 2001-02 Data using variable 'Mopup' created by AD based on date of survey

**Variable**	**Survey**	**Mop-up**	**Total**
Interviewed	6435 (91.4%)	605 (8.6%)	7040
Male	3812 (59.2%)	288 (47.6%)	4100
Female	2623 (40.8%)	317 (52.4%)	2940
Ever married at follow-up	822 (12.8%)	179 (29.6%)	1001 (14.2%)
HIV males	4 (0.11%)	2 (0.71%)	6 (0.15%)
HIV females	36 (1.37%)	7 (2.21%)	43 (1.47%)
HSV2 males	440 (11.6%)	45 (15.9%)	485 (11.9%)
HSV2 females	516 (19.8%)	98 (30.9%)	614 (21.1%)

Active Syphilis males	63 (1.7%)	2 (0.7%)	65 (1.6%)
Active Syphilis females	100 (3.8%)	1 (0.3%)	101 (3.4%)
CT males	21 (0.6%)	1 (0.4%)	22 (0.5%)
CT females	111 (4.2%)	14 (4.4%)	125 (4.3%)
NG males	7 (0.18%)	3 (1.04%)	10 (0.24%)
NG females	49 (1.87%)	4 (1.26%)	53 (1.80%)

2. Aim of Mop-up

To increase the number of eligible females interviewed during MkV1 FS in order to increase the power of our study to detect a difference in HIV prevalence and to decrease potential bias in our sample selection.

3. Methods

(i) Reminder of relevant protocol procedures

Census: House to house census was carried out to identify young people (YP) who might be eligible to participate in MkV1 FS (700-900 YP invited per community). These potentially eligible YP are all invited to attend the survey, irrespective of whether they are currently present or not. If the household head reports a young person who is eligible to be invited to survey but who has moved away, then the census interviewer leaves an invitation for that YP but also fills out a Moved-Away Form (recording information on whereabouts of YP and their contact details).

Survey: The eligibility of YP is confirmed at the survey site using a list of those enrolled in MkV1 in 1998 or a list of Std 7 students for trial schools in the subsequent years (2002, 2003 and 2004). Tracers go back to the households to try to find invited YP who did not attend the survey. If the household head or other community member reports that the YP has moved away then the tracer will fill out a moved-away form. In the most recent communities, the survey team leader has been visiting secondary schools to see if there are any YP there who might be eligible (ie any YP in a secondary school who did not receive an invitation from census team). During tracing, tracers occasionally find non-invited potentially eligible and they invite these YP to the survey site.

(ii) Target population for mop-up

During the mop-up we will primarily target females but if we meet males who are eligible then they will also be interviewed. The females that we did not interview fall into three target populations (Figure 1, Table 4):

1. Females invited <u>without</u> moved-away form.
2. Females invited <u>with</u> moved-away form.
3. Females never identified during census or survey.

Figure 1 Target populations for mop-up

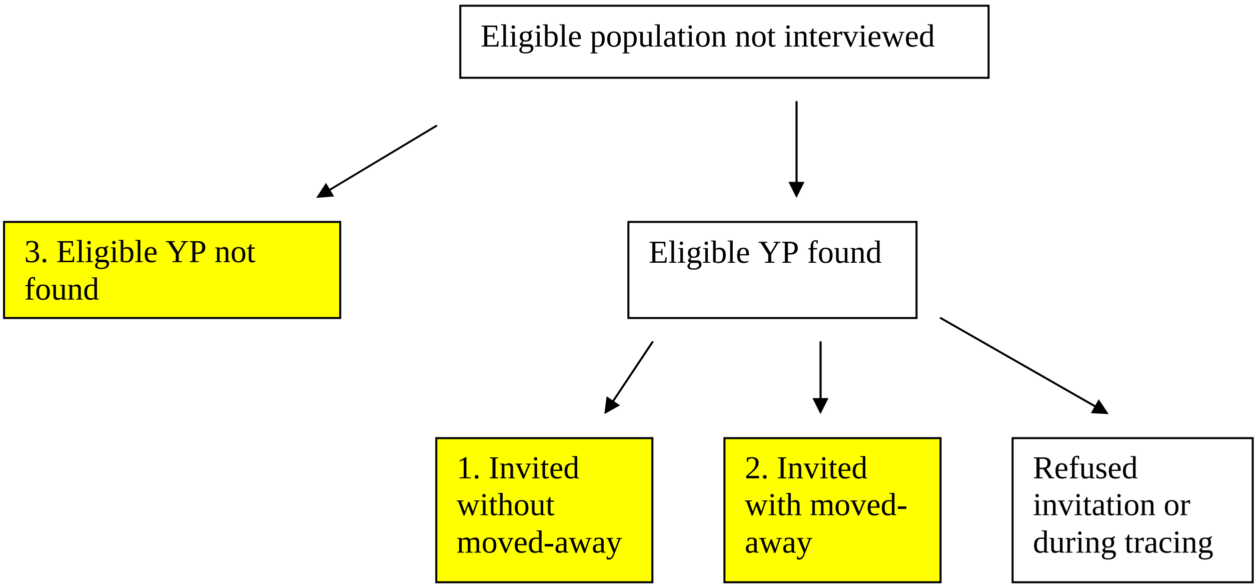

(iii) Target sample size

Given our current recruitment for females we would need to find another 1840 females to reach our target of 365 females/ community.

1. **Invited (no moved away form):** estimate 50% success in interviewing the ~50 females/ community → **25 females interviewed / cmty**

2**. Invited (with moved away form):** estimate total of ~60 females/ community with ~10 living in the community and ~ 50 at a migration point. In the community estimate will interview ~ 5 females and at the migration point estimate will interview ~ 15 females → **20 females interviewed / cmty**

3. **Never identified:** estimate total of ~ 100 females/ cmty with 20-30 living in community and 70-80 at a migration point. In the community estimate will interview ~8 females and at the migration point estimate will interview 2 females → **10 females interviewed / cmty**

Total: 55 interviewed / community
(~ 38 in the community, ~17 at migration points)

1100 additional females
(~760 in trial communities, ~340 at migration points)

In the above estimations we have included those identified during mop-up at the migration points as they will be identified by their community of origin. It is anticipated that both the target number of females and the number of females interviewed will vary between communities.

AD: The above figures are just rough estimations and ongoing analysis of existing census/ survey data will improve estimations of numbers of females in each of populations 1 and 2 in each community.

Table 4: Summary of current ideas re mop-up strategies

**Target Population**	**Likely size of this population**	**Methods of tracing**	**Information available on YP**	**Likelihood of success for males/ females**
1. Invited, no M-away	**50-150 per community** (likely that at least 50% are male)	(i) Return to HH	Name of YP and location of HH	Might get 50% (?) of those who were invited and did not attend but will depend on proportion of YP who did not attend because they were really passive refusers vs those who were busy/forgot.<br><br>Likely to be more males than females in this population (more females have MA form, more males who are invited attend)
2. Invited, with M-away	50-250 moved-away/ cmty but some of these may have attended so estimate **~ 100-150 per community and 2000- 2500 for all cmtys** (likely at least 50% are female)	(i) Migration points with/without return to HH to collect more information	Name of YP, location depends on moved-away form/ additional info collected at HH	Expect <50% of those who we attempt to trace and expect that will only try to trace ~50% because we would need to restrict this to major migration points where we expect several YP to be based on Moved-Away Forms.
3. Never found	➢ 1500-2000 potentially eligible attended school in cmty<br>➢ probably 1000-1500 living or recently lived in cmty.<br>➢ have invited 700-1000 per cmty<br>➢ expect maximum of **300-500 per community** who were never found- likely higher number of females?	(i) Return to HH not visited or not interviewed (HH absent)<br>eg in Misasi 48 HH absent<br><br>(ii) Return to community and ask trial school teachers using master list of potentially eligible<br><br>(iii) Secondary schools with master list of potentially eligible	(i) name of HH head and location of HH<br><br>(ii) Master list (ie MkV enrolment list and Std 7 lists)- information provided by community members to allow tracing at migration points<br><br>(iii) Master list (ie MkV enrolment list and Std 7 lists)	(i) Could be quite successful for HH never visited or visited only once but if HH ‘absent’ after 3 visits then less likely will find anyone there during mop-up visit- likely to find slightly more males<br><br>(ii) If most eligible YP living in community have already been found then would at best get information on current location of YP ie moved-away form- more females moved away?<br><br>(iii) High if trial villages in catchment area for secondary school that has not already visited- low probability of finding in other migration points- likely slightly more males than females in secondary schools

(iii) Preparation of documents for mop up in Mwanza

Prior to mop-up a number of lists and forms need to be produced to help during the mop-up activities. Annex 1 lists all potential sources of information available during preparations.

<u>Household head list (by kitongoji)</u>
Variables: Name of HH head, Kitongoji, no. times visited

This list will contain names of all households that were not visited or visited but found absent on all occasions. This list can be produced by cross checking the master household head list with the CI form (and household head consent form if necessary). The list will be ordered by village and will indicate the number of times the household has already been visited.

<u>Master list of potentially eligible YP (by school and separated by sex)</u>
Electronic variables: Name, school, year std 7 (or year would have reached std 7), MkV1 ID no.
Manually entered variables: invited (yes/no), attended (yes/no), interviewed (yes/no), moved away (yes/no), sufficient moved away info (yes/no), Traced and refused to attend (yes/no)

This list will be produced by entering the Std 7 lists and electronically appending this list to the list of MkV1 trial recruited YP. This list can be merged with the survey database by MkV1 ID number in order to identify MkV1 cohort members who attended the survey.

Members of the MkV1 FS team will then manually compare the master list to
- (i) the registration book and print out from survey database (including MkV1 ID no, school & years attended) to identify those who attended
- (ii) the census database and/or list A1/A2 to identify those who were invited and did not attend
- (iii) the moved away forms (or moved away form database) to see if a form is available for the young person.
- (iv) The tracing forms to see if invited YP was traced and refused to attend
- (v) List of YP who attended survey in village different from their original village to double-check that these YP have been noted on master list

The master list will be annotated to indicate the status of each young person (see list of variable above) and can be colour coded for ease of use during mop-up eg

Green: interviewed OR attended and not eligible
Yellow: invited no moved away form
Orange: invited with moved away form
Pink: invited with moved away form with insufficient information
Red: refused to attend/ accept invitation
No colour: not identified

<u>List of moved away forms needing further information (by kitongoji)</u>
Variables: name of HH head, Kitongoji, Name of YP

The existing moved away forms will be summarised by migration point to identify likely destinations for the mop-up teams. Moved away forms with insufficient information will be identified electronically and a list of households to be revisited to improve moved away form information will be generated. The original moved away

forms will be used by the fieldworker during the mop-up and annotated in a different colour pen with additional information provided at the household.

List of YP to be invited to the survey site to visit the clinician (by kitongoji)
This list will be generated from the survey database and will include those who need to be treated for CT, NG and Syphilis and an additional 20% of young people who will be interviewed by the clinician about their experience of the trial eg if 30 YP in the community to be treated then 36 YP will be invited.

The standard operating procedures (SOP) will be similar to those the teams have been using in the further survey. However, revised moved away forms, tracing forms and revised SOP for registration interviewer and tracers will be produced for use by the mop-up teams.

(vi) Official permissions and clearances

Official permissions have already been obtained to work in the 4 original trial districts (Geita, Sengerema, Missungwi and Kwimba).

MkV1FS senior staff will visit the RAS office to talk about the Mop-up and the need for further work in non-trial districts. They will request the RAS to write a letter introducing MkV1 FS to the District Executive Directors (DED), District Medical Officer (DMO) and the District Education Officers (DEO) in the districts that did not participate in the trial ie districts where migration points are situated.

(vii) Pilot study

There will be no formal piloting of the study procedures as they have already been tested in the previous sweep of the communities. However, during the last village of the main survey (Katoro village) the team will test the use of the master list to identify YP and investigate the success of revisiting HH to collect improved moved away form information.

Before leaving Mwanza for mop-up the newly formed mop-up teams will receive training on the new SOPs and documents to be used during mop-up. The first mop-up team will leave a few days before the other teams and will be given extra time in the first community so as to learn the best tracing techniques. Lessons learnt will be shared between teams.

(viii) Mobilisation

A mobilisation officer will travel to the first mop-up community of each team a few days before they arrive to inform the WEO, WEC and Village leaders that the MkV1 FS team are returning.
Mobilisation will not involve any formal meetings as this is a continuation of work carried out previously. The mobilisation officer will also organise accommodation for the teams in the first community. For all subsequent communities the teams will ensure that at least one team member visits the next community to meet with the ward and village leaders and organise accommodation. The teams can use previously collected telephone contact details for local leaders to help them in their arrangements.

The mobilisation officer with the assistance of MkV1 FS senior staff will then start mobilisation at the major migration points. One or two MkV1 FS senior staff will visit all the districts where the mop-up will be done to explain to the DAS, DED, DEO and DMO the rationale of doing Mop-up. If that District wasn't among the four MkV1 FS was done explain to them that that is one of the districts with migration points where the field staff will be visiting selected villages, centres and/or towns to get YP. For the new Ward the staff will give a brief introduction in addition to the letter of introduction to the WEO. The senior staff in the team will visit the village leaders and explain the aim of the mop-up and the time expected to stay in the village. For the new village explanations about MkV1FS will be given

(ix) Methods for Mop-up

Phase 1 of Mop-up

Three mop-up teams will revisit each of the 20 trial communities to try to find eligible females and gather more moved away forms and/or improve the quality of information on the existing moved away forms.

When a mop-up team reaches the community they will set up a survey centre in the main village in that community and organise themselves to complete the following tasks:

1. Revisit all households which were not interviewed during the census
2. Revisit all hholds with a female who was invited to the survey, did not actively refuse to attend, but did not actually attend. These should be revisited whether or not they had a Moved Away Form, but with highest priority being those who did not have a Moved Away Form, then those with an incomplete Moved Away Form, then those with a complete Moved Away Form.
3. Visit households where moved away form information for a female is incomplete and seek further information/ contact details for moved away female.
4. Visit all trial schools with Master list of eligible students and with the help of the teachers try to locate females who were not identified during the census/survey.
5. Visit any secondary school in that community to try to find additional eligible females.

If an eligible female is found the fieldworker will invite the female to the survey site or, preferably, accompany her to the survey site.

Phase 2 of Mop-up

Following phase 1 the moved away form information will be summarised and locations with high numbers (eg 50 +) of potentially eligible females will be selected for phase 2. The three mop-up teams will then go to these major migration points outside the trial communities.

When a mop-up team arrives in the major migration point they will set up a survey centre. Team members will, using the moved away form information, try to contact potentially eligible females. If an eligible female is found the fieldworker will invite the female to the survey site or, preferably, accompany her to the survey site.

(viii) Team structure

There will be three survey teams with 15 members each.

Team Leader (1)
Clinical Officer (1)
Counsellors (2)
Tracers (4)
Female registration interviewer (1)
Interviewers (1 male, 2 female)
Lab worker (1)
Driver (2)

Table 5: Proposed structure of mop-up teams

S/N	Core Survey (Day 1-3)	Survey (Day 2-3) /Tracing (Day 1)	Tracing (Day 1-3)
1.	Clinician (Lab)	Laboratory tech.	Tracer (lab)
2.	Counsellor (Lab)	Female SI	Tracer (male SI)
3.	Female (RI/SI)	Counsellor	Tracer (CI)
4.	Male (RI/SI/Data checker)	Female RI	Tracer (CI)
5.		TL	Driver
			Driver

(ix) Survey site procedures

The procedures at the survey site during the mop-up will be similar to those used during main survey. However, most of the team will work as tracers during the first day of the survey leaving only the ‘core survey team’ at the survey site. On subsequent days, depending on workload, the ‘survey/ tracing team’ will either work at the survey site or work as tracers. The best tracers and drivers will spend all their time tracing.

Table 6: Estimated time taken for mop-up in each community

	Estimated number of females to trace	Estimated number of males* and females interviewed	Days taken by survey team	Estimated number of extra YP to see clinician (STI Rx)
			10 people interviewed on day1<br><br>25 people interviewed each day on days 2 & 3	
**Community**	**350**	**60**	**3 days**	**30-40**
**Village**	~58	10	0.5 days	5-7

** The teams will prioritise the tracing of females but if they meet an eligible male then he will also be interviewed*

At the migration points it is estimated that the teams will target ~ 2000 females over a 10 day period. Each team will trace ~ 70 females per day and will expect to interview ~ 30 females/ day.

4. Timetable and Logisitcs

Each team will have 2 landcruisers to transport them from community to community and to assist with tracing (6 vehicles in total). Sample collection and supervision will be done with another borrowed/hired vehicle or using the existing team vehicles.

Table 7: Proposed timetable for mop-up teams

Order of communities	Community name & number			Community intervention status & stratum		
	Team 1	Team 2	Team 3	Team 1	Team 2	Team 3
1.	Katunguru (5)	Lubanga (10)	Koromije (22)	I1	C3	C1
2.	Kasamwa (11)	Busisi (6)	Mwagi (23)	I1	C2	C1
3.	Nyang'whale (14)	NKome (1)	Malya (24)	C2	C3	I2
4.	Bukoli (13)	Katwe (2)	Misasi (18)	C1	I3	I1
5.	Katoro (8)	Nyehunge (3)	Usagara (21)	I2	I2	C2
6.	Kagu (9)	Ihanamilo (12)	Fukalo (17)	I3	I2	I3
7.	Nyakaliro (4)		Hung'malwa (16)	C3		C2

Table 8: Timeline for Mop-up

	All teams			
**Week**				
**12-16 May 08**	Finalise mop-up protocol<br>Prep materials for Katoro			
**19-23 May 08**	Pilot testing of mop-up procedures in Katoro<br>Prep materials for other communities			
**26 May -03 Jun 08**	Training of mop-up teams<br>Prep materials for other communities			
	**Team 1**	**Team 2**	**Team 3**	**Sample collection**
**4-7 June 08**	Katunguru	Prep in Mza	Prep in Mza	
**9-14 June 08**	Katunguru	Prep in Mza	Koromije	Katung, Korom
**16-21 June 08**	Kasam/Nyang	Lub/ Busi	Mwag/Malya	Geita, Malya
**23- 28 June 08**	Buk/Katoro	Nkome/ Kat	Mis/Usag	Geita, Usag
**30 Jun – 4 July 08**	Kagu/Nyak	Nyeh/ Ihan	Fuk/Hung	Geita, Hung
**6 Jul- 11 Jul 08**	Migr point	Migr point	Migr point	Migr points
**13 Jul- 20 Jul 08**	Migr point	Migr point	Migr point	Migr points

5. Anticipated challenges

The registration interviewers will have to take care to check that a previously interviewed eligible YP is not interviewed again.

Poor documentation for first communities visited eg some households not on household head list.

Multiple copies of census ID number making merging with survey database difficult.

Logistics – especially sample collection

6. Advantages of mop-up

The mop-up will take place during the school holidays (except for Form 2 students who are preparing for exams) and this may mean that we find more eligible people in their home villages.

Some young people leave the villages during the farming season and may have now returned to their home village.

Young people who were afraid to participate in the survey/ to be identified may be willing to participate now as the community understand what MkV1 FS is doing and no longer fear?

Reports from the field suggest that in some communities eg Bukoli not all households were visited as households were very far from each other. The work of the census checker suggests that even where households are visited potentially eligible young people are missed.

Annex 1: Sources of information

	Source	Format	Useful information	Potential uses
A	Household Head List	A4 form completed by hand	Names of all HH heads by kitongoji	Cross check with CI form to see if all HH were visited
B	Census Interviewer (CI) Form	A4 form completed by hand	Names of all HH and whether visited or not	Identify HH where upon last visit the HH was '2- absent'
C	Household head consent form	A4 form completed by hand	Names and signatures of all HH heads interviewed	To cross check with Household head list and CI form
D	Census Database	Access file	All variables collected during census	To create lists of invited, source of GPS codes
E	List A1	List produced from census Database with participant stickers attached	Names of all invited males per village and stickers for interviewed participants	To create list of those invited who did not attend
F	List A2	As A1 above	As A1 above for females	To create list of those invited who did not attend
G	List A3	List produced from census database for tracers	Details on households with invited YP including GPS code	
H	Survey invitation	A4 form completed by hand	Names and invitation number for invited YP who attended	To cross check against registration book and list A1/A2 to see who was invited
I	Survey Registration book	Handwritten registration book	Names, invitation no. for all interviewed YP	To list YP who attended (invited and not invited)
J	Survey database	Dbase/ STATA file	All variables collected during survey including (if applicable) invitation no and MkV1 ID no	To list YP who attended (invited and not invited)
K	Tracing forms	A4 form completed by hand		Provide information on those who were traced and eg refused
L	Moved away forms	A4 form completed by hand- Entered into Access		Compare to list A1/A2 or census database to identify those invited with/without moved away
M	MkV1 Database	STATA file	List of names of MkV1 recruited incl MkV1 ID no	Combine with Std 7 list to produce Master List of those eligible for MkV1 FS
N	Std 7 lists	Handwritten or typed A4- propose to enter into Access	List of names of those in MkV trial schools 2002, 2003, 2004	Combine with MkV1 database to produce Master List of those eligible for MkV1 FS

MEMA kwa Vijana Trial Further Survey (2007-2008)

MkV1 FS: List of Household heads for Mop-up

Jina la Kijiji: ___________________________

Jina la Kitongoji: ______________________________________

S/N ya Kaya	Jina kamili la mkuu wa KAYA	Jinsia ya Mkuu wa kaya	How many times visited and absent during main survey

Order of communities	Community name & number			Community intervention status & stratum		
	Team 3	Team 2	Team 1	Team 3	Team 2	Team 1
1.	Kasamwa (11)	Lubanga (10)	Mwagi (23)	I1	C3	C1
2.	Nyang'whale (14)	Busisi (6)	Malya (24)	C2	C2	I2
3.	Bukoli (13)	NKome (1)	Misasi (18)	C1	C3	I1
4.	Katoro (8)	Katwe (2)	Usagara (21)	I2	I3	C2
5.	Kagu (9)	Nyehunge (3)	Fukalo (17)	I3	I2	I3
6.	Nyakaliro (4)	Ihanamilo (12)	Hung'malwa (16)	C3	I2	C2
7.		Katunguru (5)	Koromije (22)		I1	C1

Table 8: Timeline for Mop-up

	All teams			
**Week**				
**12-16 May 08**	Finalise mop-up protocol<br>Prep materials for Katoro			
**19-23 May 08**	Pilot testing of mop-up procedures in Katoro<br>Prep materials for other communities			
**26 May -03 Jun 08**	Training of mop-up teams<br>Prep materials for other communities			
	**Team 1**	**Team 2**	**Team 3**	**Sample collection**
**3-7 June 08**	Malya	Prep in Mza	Prep in Mza	
**9-14 June 08**	Mwagi/Misasi	Lubanga	Koromije	Katung, Korom
**16-21 June 08**	Usag/Fuk	Busi/ Nkome	Mwag/Malya	Geita, Malya
**23- 28 June 08**	Hung/Korom	Nkome/ Kat	Mis/Usag	Geita, Usag
**30 Jun – 4 July 08**		Nyeh/ Ihan	Fuk/Hung	Geita, Hung
**6 Jul- 11 Jul 08**	Migr point	Migr point	Migr point	Migr points
**13 Jul- 20 Jul 08**	Migr point	Migr point	Migr point	Migr points

MEMA Kwa Vijana Further Survey (2007 – 2008)

MEMA KWA VIJANA,
P.O. BOX 11936,
MWANZA

TAREHE……………….

Ndugu…………………………………………..

YAH: MWALIKO WA KUHUDHURIA KWENYE UTAFITI

Mimi ni Mjumbe wa Mema Kwa Vijana Mwanza, nimefika kwenye Kaya yako lakini sikukukuta. Hivyo nitarudi siku ya ……………………Tarehe……………………….
Katika Kaya yako nitahitaji kuonana na wafuatao:-

……………………………………………. ………………………………………………

…………………………………………… ………………………………………………

Ambaye/ambao amepewa/wamepewa mwaliko wa Utafiti wa Mema Kwa Vijana.

Upatapo ujumbe huu naomba unisubiri au mjulishe/wajulishe; mlengwa/walengwa ahudhurie/wahudhurie katika Kituo chetu cha Utafiti kilichopo…………………………………………………………………………

Tafadhali mwambie/waambie afike/wafike na Kadi yake/zao za Mwaliko (Rangi ya Kijani) na Kitambulisho chochote.

Natanguliza shukrani zangu za dhati kwa ushirikiano wako.

Wako katika kuboresha Afya za Vijana,

………………………………

………………………………

MEMA KWA VIJANA (MKV1 FS)

MKV_REMINDER INV FORM_SWAHILI_22/8/08

MEMA kwa Vijana Trial Further Survey (2007-2008)

Moved Away Form (Swahili) For Mop-up

Village: ____________________, Kitongoji: _____________________

1.1	Namba ya siri ya mtumishi	|__||__||__|
1.2	Tarehe	|__||__|/|__||__|/200|__|
1.3	Namba ya siri ya sensa (Mwaliko)	⊗ ⊗
1.4	Jina la Kijana:	
1.5	Majina mengine (kama yapo)	
1.6	Nani aliyetoa taarifa hizi?<br>01=Mzazi/mlezi; 02= Ndugu wa karibu; 03= Jirani; 05= kiongozi wa jamii; 05= mwingine	|__||__|
1.7	Kijana ameondoka kwa sababu gani?<br>01= kikazi; 02= kuishi na ndugu; 03= masomo; 04= kuishi na mme/mke au mpenzi; 05= Nyingine	|__||__|
1.8	Je, Kijana ameondoka kimoja? (zungushia moja)<br><br>Kama HAPANA→ Anategemewa kurudi lini?<br>01= Ndani ya wiki 1; 02= ndani ya wiki 3; 03= zaidi ya wiki 3; 99= haifahamiki	Yes 1<br>No 2<br>|__||__|

2 Sehemu aliyohamia

2.1	Wilaya ________________________________	
2.2	Kata ___________________________________	
2.3	Kijiji _______________________________	
2.4	Kitongoji/mtaa/barabara ______________________________	
2.5	Jina la mkuu wa kaya <anakoishi kwa sasa>___________________________________	
2.6	Jina la mke au mme *<kama ameoa/ameolewa>*<br>____	
2.7	Jina la mtu mwingine wa kumuuliza <sehemu ya sasa> ______________________________	
2.8	*Tafadhari andika taarifa zingine zitakazo weza kusaidia kumpata huyu kijana (mf: annuani nyingine; maelezo ya sehemu nyumba anayoishi ilipo; shule anayo soma; kazi anazofanya na eneo zilizopo.*<br>____________________<br>____________________<br>____________________<br>____________________<br>____________________<br>____________________<br>____________________	
2.9	Jinsia	
2.10	Jina la mototo wa kwanza (kama ana mototo/watoto)	

MkV1 FS Mop-up- List of Documents needed for each community

Original forms:
Registration Book
List A1/ A2
Tracing Forms
CI Forms & Master List of HH heads **OR** Mop-up list of HH heads

New forms:
Master List of potentially eligible YP
List L1/L2
List L3/L4
List L5/L6
List L9/L10 (one list for all communities)
List L11/L12 (one list for all communities)

Moved into community list
Moved away from community list

List for STI treatment

MEMA kwa Vijana Trial Further Survey (2007-2008)

MKV1 FS: CI FORM FOR MOP-UP

VILLAGE NAME: ___________________ ***Code*** **:_____ Kitongoji: (NAME):** _________________________________

S/NO	NAME OF HH HEAD	SEX	CENSUS STATUS			
		M	**Date & Time**			
		F	**Outcome** *1=Inter, 2= Absent, 3= Refused 4= Repeat HH, 5= HH unknown, 6= HH shifted*			
		M	**Date & Time**			
		F	**Outcome** *1=Inter, 2= Absent, 3= Refused 4= Repeat HH, 5= HH unknown, 6= HH shifted*			
		M	**Date & Time**			
		F	**Outcome** *1=Inter, 2= Absent, 3= Refused 4= Repeat HH, 5= HH unknown, 6= HH shifted*			
		M	**Date & Time**			
		F	**Outcome** *1=Inter, 2= Absent, 3= Refused 4= Repeat HH, 5= HH unknown, 6= HH shifted*			
		M	**Date & Time**			
		F	**Outcome** *1=Inter, 2= Absent, 3= Refused 4= Repeat HH, 5= HH unknown, 6= HH shifted*			
		M	**Date & Time**			
		F	**Outcome** *1=Inter, 2= Absent, 3= Refused 4= Repeat HH, 5= HH unknown, 6= HH shifted*			
		M	**Date & Time**			
		F	**Outcome** *1=Inter, 2= Absent, 3= Refused 4= Repeat HH, 5= HH unknown, 6= HH shifted*			
		M	**Date & Time**			
		F	**Outcome** *1=Inter, 2= Absent, 3= Refused 4= Repeat HH, 5= HH unknown, 6= HH shifted*			
		M	**Date & Time**			
		F	**Outcome** *1=Inter, 2= Absent, 3= Refused 4= Repeat HH, 5= HH unknown, 6= HH shifted*			
		M	**Date & Time**			
		F	**Outcome** *1=Inter, 2= Absent, 3= Refused 4= Repeat HH, 5= HH unknown, 6= HH shifted*			
		M	**Date & Time**			
		F	**Outcome** *1=Inter, 2= Absent, 3= Refused 4= Repeat HH, 5= HH unknown, 6= HH shifted*			
		M	**Date & Time**			
		F	**Outcome** *1=Inter, 2= Absent, 3= Refused 4= Repeat HH, 5= HH unknown, 6= HH shifted*			

MEMA kwa Vijana Trial Further Survey (2007-2008)

S/NO	NAME OF HH HEAD	SEX	CENSUS STATUS			
		M	**Date & Time**			
		F	**Outcome** *1=Inter, 2= Absent, 3= Refused*<br>*4= Repeat HH, 5= HH unknown, 6= HH shifted*			
		M	**Date & Time**			
		F	**Outcome** *1=Inter, 2= Absent, 3= Refused*<br>*4= Repeat HH, 5= HH unknown, 6= HH shifted*			
		M	**Date & Time**			
		F	**Outcome** *1=Inter, 2= Absent, 3= Refused*<br>*4= Repeat HH, 5= HH unknown, 6= HH shifted*			
		M	**Date & Time**			
		F	**Outcome** *1=Inter, 2= Absent, 3= Refused*<br>*4= Repeat HH, 5= HH unknown, 6= HH shifted*			
		M	**Date & Time**			
		F	**Outcome** *1=Inter, 2= Absent, 3= Refused*<br>*4= Repeat HH, 5= HH unknown, 6= HH shifted*			
		M	**Date & Time**			
		F	**Outcome** *1=Inter, 2= Absent, 3= Refused*<br>*4= Repeat HH, 5= HH unknown, 6= HH shifted*			
		M	**Date & Time**			
		F	**Outcome** *1=Inter, 2= Absent, 3= Refused*<br>*4= Repeat HH, 5= HH unknown, 6= HH shifted*			
		M	**Date & Time**			
		F	**Outcome** *1=Inter, 2= Absent, 3= Refused*<br>*4= Repeat HH, 5= HH unknown, 6= HH shifted*			
		M	**Date & Time**			
		F	**Outcome** *1=Inter, 2= Absent, 3= Refused*<br>*4= Repeat HH, 5= HH unknown, 6= HH shifted*			
		M	**Date & Time**			
		F	**Outcome** *1=Inter, 2= Absent, 3= Refused*<br>*4= Repeat HH, 5= HH unknown, 6= HH shifted*			
		M	**Date & Time**			
		F	**Outcome** *1=Inter, 2= Absent, 3= Refused*<br>*4= Repeat HH, 5= HH unknown, 6= HH shifted*			
		M	**Date & Time**			
		F	**Outcome** *1=Inter, 2= Absent, 3= Refused*<br>*4= Repeat HH, 5= HH unknown, 6= HH shifted*			
		M	**Date & Time**			
		F	**Outcome** *1=Inter, 2= Absent, 3= Refused*<br>*4= Repeat HH, 5= HH unknown, 6= HH shifted*			
